# A subset of chemosensory genes differs between two populations of a specialized leaf beetle after host plant shift

**DOI:** 10.1002/ece3.4246

**Published:** 2018-07-20

**Authors:** Ding Wang, Stefan Pentzold, Maritta Kunert, Marco Groth, Wolfgang Brandt, Jacques M. Pasteels, Wilhelm Boland, Antje Burse

**Affiliations:** ^1^ Department of Bioorganic Chemistry Max Planck Institute for Chemical Ecology Jena Germany; ^2^ Leibniz Institute on Aging – Fritz Lipmann Institute Jena Germany; ^3^ Leibniz Institute of Plant Biochemistry Halle (Saale) Germany; ^4^ Department of Biology Université Libre de Bruxelles Brussels Belgium

**Keywords:** chemosensory genes, *Chrysomela lapponica*, host plant shift, leaf volatile analysis, structural protein modeling, transcriptomics

## Abstract

Due to its fundamental role in shaping host selection behavior, we have analyzed the chemosensory repertoire of *Chrysomela lapponica*. This specialized leaf beetle evolved distinct populations which shifted from the ancestral host plant, willow (*Salix* sp., Salicaceae), to birch (*Betula rotundifolia*, Betulaceae). We identified 114 chemosensory candidate genes in adult *C. lapponica*: 41 olfactory receptors (ORs), eight gustatory receptors, 17 ionotropic receptors, four sensory neuron membrane proteins, 32 odorant binding proteins (OBPs), and 12 chemosensory proteins (CSP) by RNA‐seq. Differential expression analyses in the antennae revealed significant upregulation of one minus‐C OBP (*Clap* OBP27) and one CSP (*Clap* CSP12) in the willow feeders. In contrast, one OR (*Clap* OR17), four minus‐C OBPs (*Clap* OBP02, 07, 13, 20), and one plus‐C OBP (*Clap* OBP32) were significantly upregulated in birch feeders. The differential expression pattern in the legs was more complex. To narrow down putative ligands acting as cues for host discrimination, the relative abundance and diversity of volatiles of the two host plant species were analyzed. In addition to salicylaldehyde (willow‐specific), both plant species differed mainly in their emission rate of terpenoids such as (*E*,*E*)‐α‐farnesene (high in willow) or 4,8‐dimethylnona‐1,3,7‐triene (high in birch). Qualitatively, the volatiles were similar between willow and birch leaves constituting an “olfactory bridge” for the beetles. Subsequent structural modeling of the three most differentially expressed OBPs and docking studies using 22 host volatiles indicated that ligands bind with varying affinity. We suggest that the evolution of particularly minus‐C OBPs and ORs in *C. lapponica* facilitated its host plant shift via chemosensation of the phytochemicals from birch as novel host plant.

## 1 INTRODUCTION

Phytophagous beetles have undergone a 140 million years lasting period of coevolution and coadaptation with their host plants (Labandeira & Currano, 2013; Wang, Zhang, & Jarzembowski, 2013). Currently, it is debated how such an interaction diversifies during evolution and how the interaction affects the modes and rates of the speciation of beetles and plants (Barrett & Heil, 2012; Futuyma & Agrawal, 2009; Tilmon, 2008). The most successful herbivorous beetle lineages (Curculionoidea and Chrysomeloidea), forming the clade “Phytophaga,” have developed different patterns of life history strategies to use plants as efficient food source (Farrell, 1998; Farrell & Sequeira, 2004; Fernandez & Hilker, 2007; Gómez‐Zurita, Hunt, Kopliku, & Vogler, 2007). Plant secondary metabolites are often key players in this relationship as they can deter generalist herbivores, but attract specialized and adapted herbivores. Thus, plant secondary metabolites contribute to host specialization of phytophagous beetles (Mithofer & Boland, 2012). Although the adaptation to plant metabolites promotes diet specialization, it does not inevitably lead to evolutionary “dead ends” (Day, Hua, & Bromham, 2016; Termonia, Hsiao, Pasteels, & Milinkovitch, 2001). Over ecological and evolutionary timescales, both plant and insect herbivores may change their geographic range generating novel plant–herbivore interactions often including host plant shifts.

In the affiliations of novel host plants, the insect chemosensory system represents the first barrier to be overcome (del Campo et al., 2001). Based on nutritional and secondary metabolites, this system discriminates among a chemical mosaic of different plant species and triggers physiological processes and an appropriate feeding behavior (Dahanukar, Hallem, & Carlson, 2005). The evolution of the sensory repertoire could provoke and reinforce adaptations of insects to new hosts. Such host plant shifts have also occurred during the evolutionary history of the leaf beetle subtribe Chrysomelina (Chrysomelidae, Chrysomeloidea). Some species of the monophyletic *interrupta* group escaped plant constraints by shifting host families (Termonia et al., 2001). In the species *Chrysomela lapponica*, for example, geographically separated populations in the Eurasian Palearctic have evolved that colonize and eat either willow (*Salix* sp.; Salicaceae) or birch leaves (*Betula* sp.; Betulaceae) (Geiselhardt, Hilker, Muller, Kozlov, & Zvereva, 2015; Zvereva, Hunter, Zverev, & Kozlov, 2016). Reconstructions of the host shift history of allopatric *C. lapponica* populations disentangled that willow is the ancestral feeding niche and that the transition to Betulaceae occurred several times independently, possibly after the last glacial episode during the last 10,000 years (Mardulyn, Othmezouri, Mikhailov, & Pasteels, 2011). Besides the resistance traits of host plants, further biotic factors may determine host affiliation. For example, ecological studies carried out on different *C. lapponica* populations revealed that juvenile willow feeders are frequently exposed to specialized parasitoids and predators, while birch feeders escaped this top‐down pressure and occupied thus an enemy‐free space (Gross, Fatouros, Neuvonen, & Hilker, 2004; Zvereva & Rank, 2003, 2004). The populations of *C. lapponica* selecting different host plant families represent an excellent model system to investigate the role of the chemosensory system during and after host plant shifts by herbivorous insects.

In insect herbivores, two major chemosensory mechanisms, the sense of taste and smell, largely contribute to selecting appropriate host plants (Pentzold, Burse, & Boland, 2017; Suh, Bohbot, & Zwiebel, 2014). While smell (olfaction) is a sense enabling insects to detect and discriminate between numerous volatile molecules, taste (gustation) is the sensory impression of mainly nonvolatile substances (Van Naters & Carlson, 2006). The reception of chemical cues from the environment is mediated by receptor neurons residing in peripheral organs such as antennae, palps, or legs which are covered by hair‐like sensilla (Hallem, Dahanukar, & Carlson, 2006). The sensilla house the dendrites of a varying number of these neurons which project into the central nerve system (Yarmolinsky, Zuker, & Ryba, 2009). In smell and taste, the receptor proteins of peripheral neurons play a pivotal role as biological transducers that convert external chemical signals into a sensory input. These receptors operate as ligand‐gated ion channels and a potential modulatory role for G proteins, and second messenger downstream of the receptor is suggested (Fleischer, Pregitzer, Breer, & Krieger, 2017; Sato, Tanaka, & Touhara, 2011; Sato et al., 2008; Wicher et al., 2008; Zhang et al., 2011).

To facilitate olfaction, members of the olfactory receptor (OR) family are composed of heteromeric complexes of two subunits: a highly conserved OR coreceptor (ORco) and the other highly divergent OR subunit(s) (Leal, 2013; Suh et al., 2014). In order to sense nonvolatile tastants, and also CO_2_, insects possess gustatory receptors (GRs). GRs share motifs with ORs in their transmembrane domains, and phylogenetically, they are suggested to predate the expansion of the insect ORs (Missbach et al., 2014; Robertson, Warr, & Carlson, 2003). Gustatory receptor neurons (GRNs) express a manifold subset of GRs; however, the design principles of taste are much less understood to date than olfaction (Karner, Kellner, Schultze, Breer, & Krieger, 2015; Koutroumpa, Kárpáti, Monsempes, Hill, & Hansson, 2014).

Further classes of membrane receptors involved in chemosensation comprise the ionotropic receptors (IRs) (Benton, Sachse, Michnick, & Vosshall, 2006; Rytz, Croset, & Benton, 2013) and sensory neuron membrane proteins (SNMPs) (Vogt et al., 2009). IRs seem to act as ligand‐gated ion channels for odor coding of ligands not bound by ORs (Suh et al., 2014). SNMP recognize fatty acid‐derived odorants in trichoid sensilla (Leal, 2013). Recently, it has been shown for *Drosophila* that the ectodomain tunnel in SNMP1 allows the transfer of hydrophobic pheromones from the extracellular fluid to integral membrane receptors (Gomez‐Diaz et al., 2016).

Besides membrane proteins, also soluble proteins seem to contribute to the process of chemosensation in insects (Leal, 2013). The soluble odorant binding proteins (OBPs) and chemosensory proteins (CSPs) have the ability to bind and solubilize small hydrophobic compounds critical for various physiological processes such as nutrition, development and regeneration, vision, or chemosensation (Pelosi, Iovinella, Zhu, Wang, & Dani, 2018). OBPs and CSPs are remarkable in their number, diversity, and abundance (Brito, Moreira, & Melo, 2016; Pelosi, Iovinella, Felicioli, & Dani, 2014). OBPs have been proposed being involved in the protection of odorant molecules from the action of odorant‐degrading enzymes (Suh et al., 2014), the delivery of semiochemicals to the odorant receptors (Laughlin, Ha, Jones, & Smith, 2008), the buffering of effects of sudden changes in the level of an odorant in the environment (Larter, Sun, & Carlson, 2016), or the mediation of tolerance toward plant toxins has been suggested for OBPs of *Drosophila sechellia* (Hungate et al., 2013). The CSPs are also believed to play a role in insect olfaction, although there is still no direct evidence of this (Ozaki et al., 2005; Pelosi et al., 2018).

In this study, we test the hypothesis that the populations of *C. lapponica* which shifted their host plant species from willow to birch changed expression of components of the chemosensory repertoire in comparison to populations which kept their original host plant species, that is, willow. For this reason, we present a comparative inventory of the chemosensory systems based on transcriptome sequences from two *C. lapponica* populations that differ in their host plant preference for either willow or birch. Using RNA sequencing and qRT‐PCR, we studied the expression profiles of the chemosensory components in antennae and legs in males and females of the different populations. Our results indicate that among the chemosensory gene families, changes in expression of mainly OBPs and ORs are associated with the host shift. *C. lapponica* from a birch‐feeding population (BFP) were collected as pupae from *Betula rotundifolia* in the Altai Mountains in East Kazakhstan, close to Uryl, near the Burkhat Pass (2,130 m altitude, 49°07.438′N 86°01.365′E). Circa 60 km distant, *C. lapponica* pupae from a willow‐feeding population (WFP) were also collected in the Altai Mountains, near Katon‐Karagay, from *Salix* (most likely *S. karelinii*) (2,207 m altitude, 49°02.573′N 85°39.209′E).

## 2 METHODS

### 2.1 Collection and rearing of *C. lapponica*

*Chrysomela lapponica* (L.) were collected from the end of July to the middle of August in 2014. *C. lapponica* from a birch‐feeding population (BFP) were collected as pupae from *Betula rotundifolia* in the Altai Mountains in East Kazakhstan, close to Uryl, near the Burkhat Pass (2,130 m altitude, 49°07.438′N 86°01.365′E). Circa 60 km distant, *C. lapponica* pupae from a willow‐feeding population (WFP) were also collected in the Altai Mountains, near Katon‐Karagay, from *Salix* (most likely *S. karelinii*) (2,207 m altitude, 49°02.573′N 85°39.209′E). Circa 60 km distant, *C. lapponica* pupae from a willow‐feeding population (WFP) were also collected in the Altai Mountains, near Katon‐Karagay, from *Salix* (most likely *S. karelinii*) (2,207 m altitude, 49°02.573′N 85°39.209′E). *Chrysomela lapponica* pupae were kept in plastic boxes at local environmental temperature and light–dark cycle until adult emergence (“field laboratory” (wooden house), Uryl, 1,107 m altitude, 49°13.945′N 86°20.569′E). Adults were reared for 2 days on their corresponding host plant twigs which were collected at the beetles’ field site and kept alive for several days in tap water. After these 2 days, individuals from *C. lapponica* were dissected and the organs were stored in RNA stabilization solution (RNAlater, Qiagen, Hilden, Germany) for transport to Germany where RNA isolation took place in the laboratory. Pupae from a WFP in Finland have been collected near Utsjoki (Kevo Subarctic Research Station, 69°45′N 27°01′E). After arrival in Germany, already emerged adults have been dissected immediately.

### 2.2 Host preference assays

Individual *C. lapponica* beetles from either the BFP (*N* = 58) or the WFP (*N* = 14) were tested during daytime in the “field laboratory” for their feeding preference toward host and nonhost leaves using short‐distance two‐choice assays from the end of July to the middle of August in 2014. Undamaged twigs were cut off from the trees, in the immediate neighborhood of which also the beetles were collected. Plant twigs were taken from the sampling site to the “field laboratory” and kept in tap water until needed. In order to have comparable conditions in each choice experiment, we used only well‐developed young leaves of similar size from undamaged twigs. During the experiment, leaves were kept on moisturized filter paper in order to preserve the physiological status of the plant tissue. One beetle from either population was placed in a plastic box (16 × 12 cm) and offered one fresh young leaf of birch and one fresh young leaf of willow separated by ca. 7 cm. Beetles were allowed to move freely between the two host plant species in the box. During the 30 min of the experiment, host plant preference was tested by assigning one point to the plant species that was damaged by feeding. The chi‐square test was used to test for significant differences between responses of the individuals from both populations.

### 2.3 RNA preparing, RNA library construction, and sequencing

RNA sequencing (RNA‐seq) was carried out using Illumina HiSeq2000 (Illumina, Inc., San Diego, California) (Bentley et al., 2008). For creating transcriptome reference libraries, total RNA was pooled from entire individuals collected from all developmental stages including male and female adults, pupae, and first‐ to third‐instar larvae each of either the BFP or the WFP. For differential expression analyses, entire legs and entire antennae dissected from 20 adult females or 20 adult males per biological replicate from either population were used for total RNA isolation (two biological replicates per prepared tissue, sex, and host plant specificity, i.e., 16 sequenced samples). All total RNA samples were prepared according to Bodemann et al., 2012. Around 2.5 μg total RNA of each sample was used for the library preparation with the TruSeq RNA Sample Prep Kit v2 (Illumina Inc., San Diego, USA), according to the manufacturer's description. In order to obtain longer fragments for the transcriptome reference libraries, the fragmentation step during the preparation procedure was reduced to four minutes.

The libraries of pooled samples either from the BFP or from the WFP for the reference transcriptome were sequenced using a Genome Analyzer IIx (GAIIx, Illumina Inc., San Diego, USA) in 100‐bp paired‐end mode. The two libraries were pooled in one lane. The eight libraries of the tissue samples were sequenced using a HiSeq2000 in a 50‐bp single‐end mode by pooling four libraries per lane. All reads were extracted in FastQ format using CASAVA v1.8 (GAIIx) or v1.8.2 (HiSeq) (Illumina Inc., San Diego, USA).

### 2.4 De novo assembly of transcriptomes from *C. lapponica*

Transcriptome reference libraries were created from each population separately. The raw RNA‐seq reads were subject to adapter removal and to trimming of low‐quality regions from the 3′‐ and 5′‐ends with a minimum Phred score threshold of 20 using the tool cutadapt v1.8.1 (Martin, 2011). Afterward, the trimmed paired‐end reads of pooled samples and the trimmed single‐end reads of tissue samples were de novo‐assembled using Trinity v2012–03–17 (Grabherr et al., 2011) with a minimal contig length of 300 bp. In order to reconstruct the full‐length transcriptomes, the above de novo‐assembled transcripts were reassembled using TGI Clustering tool (v January 2009) (Pertea et al., 2003) with a minimum overlap length of 100 bp and sequence similarity of 90%.

### 2.5 Annotation of assembled transcript libraries and identification of chemosensory proteins

The assembled transcripts were translated into six possible frames using EMBOSS “transeq” v6.3.1. The BLAST2GO step was performed with an e‐value cutoff of 1e‐1 and GO Slim was not used. The remaining process was performed with default parameters.

To identify chemosensory genes from *C. lapponica* such as OBPs, CSPs, SNMPs, IRs, ORs, and GRs, we created custom reference databases of receptors described from other insect species including *Tribolium castaneum*,* Manduca sexta*,* Bombyx mori*, two bark beetles (*Dendroctonus ponderosae* and *Ips typographus*), and *Drosophila melanogaster*, whose sequences were deposited in GenBank (NCBI). The sequences of *Anoplophora glabripennis* were provided by Robert F. Mitchell (McKenna et al., 2016). All protein sequences from *C. lapponica* transcriptome libraries were searched via blastp (v2.2.29+) with an e‐value 1e‐1 against the custom databases to identify chemosensory genes.

To verify the chemosensory proteins identified by comparison with our custom databases, all the sequences were subsequently searched via blastp (e‐value 1e−3) approach against the NCBI nonredundant database (updated June 2017) (Camacho et al., 2009). The top ten hits were inspected manually, and sequences homologous to known chemosensory proteins of *C. lapponica* were identified. The species‐specific sequences of *C. lapponica* were given temporary designations as numbered series in the form of ClapXXyy (XX: chemosensory transcript; yy: number). In addition, the population‐specific sequences (i.e., sequences assembled in only one of the two populations) of *C. lapponica* are named ClapXX‐Wyy and ClapXX‐Byy for willow‐feeding and birch‐feeding beetles, respectively.

To identify the longest ORFs in all transcripts, derived protein sequences were aligned with their corresponding custom reference databases using MAFFT version 7 (option E‐INS‐I with default parameters) (Katoh & Standley, 2013). The full‐length ORFs and the incomplete sequences with more than 100 amino acids were selected for further analyses.

### 2.6 Phylogenetic analyses

The population‐specific and the longest chemosensory protein sequences between *C. lapponica* feeding on willow or birch were aligned with the homologous protein sequences derived from other insect species (Fasta dataset, Supporting Information) (Andersson et al., 2013; Attrill et al., 2016; Croset et al., 2010; Dippel et al., 2014; Engsontia et al., 2008; Grosse‐Wilde et al., 2011) by applying the E‐INS‐i methods from MAFFT with default parameters. To calculate phylogenetic trees, RAxML v7.2.8 (Stamatakis, 2006), a program based on the maximum‐likelihood inference, was used. For phylogenetic analysis of the chemosensory transcripts of *C. lapponica*, the best‐fitted model of protein evolution was chosen using Perl script ProteinModelSelection.pl (http://sco.h-its.org/exelixis/web/software/raxml/). The maximum‐likelihood phylogenetic tree was reconstructed with a bootstrap test of 100 replicates in RAxML.

### 2.7 Differential expression analysis

To find identical chemosensory transcripts in both populations, we compared sequences of BFP and WFP using blastp with an e‐value cutoff of 1e−3. As we analyzed population‐specific differences, we included datasets from males and females in the same ratio per population (e.g., for the analysis of the WFP: two biological replicates each from females’ antennae and legs, and two biological replicates each from males’ antennae and legs). To compare the transcript expression levels of the antennae (*N* = 4 per population) and legs (*N* = 4 per population) from both populations of *C. lapponica*, we mapped tissue RNA‐seq reads onto the WFP transcriptome library including BFP specific chemosensory transcripts using Bowtie2 v2.2.9 (Langmead & Salzberg, 2012) using default parameters. EdgeR (Robinson, McCarthy, & Smyth, 2010) was used to estimate abundance and detect differentially expressed transcripts in the two different tissues. To remove very low counts across all tissue libraries, we selected transcripts that were expressed in two or more libraries with counts per million (CPM) mapped reads ≥1. Trimmed mean of M‐value normalization (edgeR default normalization method) was applied to remove technical variability (accounting for compositional difference between the libraries). Using the Cox–Reid profile‐adjusted likelihood method to estimate dispersions, the generalized linear model according to Lu, Tomfohr, & Kepler, 2005 was selected to test for significant different expression of transcripts with a log_2_fold ≥ 1, a *p*‐value cutoff 0.05, and false discovery rate (FDR) cutoff 0.05 (Supporting Information Table S9). To avoid differential expression caused by low expression levels among samples, we focused on transcripts that had at least 10 CPM in one or both comparable samples. Blast2GO V4.1.9 was used to annotate molecular function, biological process, and cellular component for significantly differentially expressed transcripts when comparing antennae and legs of BFP and WFP (Götz et al., 2008).

### 2.8 qRT‐PCR validation

Chemosensory gene expression was analyzed via quantitative real‐time (qRT)‐PCR from the antennae and legs of further Kazakh *C. lapponica* individuals feeding on willow or birch (four to seven biological replicates for each organ) and from an additional WFP from Finland (four biological replicates for each organ). After homogenizing antennae and legs in liquid nitrogen, RNA was purified using RNAqueous^™^ Total RNA Isolation Kit (Ambion) including DNase treatment, following the manufacturer's instruction. Synthesis of cDNA was carried out using SuperScript III Reverse Transcriptase and oligo(dT)20 (Invitrogen), following the manufacturer's protocol. Chemosensory genes that either differed significantly (ClapOBP02, 20, 27 and OR17) or did not differ (e.g., ClapGR06 as control) between WFP and BFP according to the RNA‐seq data were used as targets for validation by qRT‐PCR. Relative gene expression (Livak & Schmittgen, 2001) using the housekeeping genes *ClEF1a* and *CleIF4A* as reference was acquired on a CFX‐96 Touch^™^ Real‐Time PCR Detection System (Bio‐Rad) using cDNA as template or distilled water as negative control. Reactions were run in a thin‐walled 96‐well Hard‐Shell PCR plate sealed with Microseal (Bio‐Rad Laboratories GmbH, Munich, Germany). Two technical replicates were analyzed; those with a Ct difference of >0.5 were repeated. For primers used, see Supporting Information Table S10. Specificity of each primer set was determined by melting curve assays as final step in the cycle program. For all genes, curves showed single sharp peak indicating specific amplifications without nonspecific PCR product formation. No signals were detected for the negative controls.

### 2.9 Protein structure modeling

Protein modeling of the three highest expressed OBPs was performed with YASARA (Krieger et al., 2009). The resulting models were evaluated by YASARA, and if appropriate, a final model was created by merging the best‐folded fragments from different models, followed by energy minimization. The quality of all models was checked for native folding by energy calculations with PROSA II (Sippl, 1990) and for stereochemical quality by PROCHECK (Laskowski, Macarthur, Moss, & Thornton, 1993). Only models of excellent quality, as indicated by a Ramachandran plot with more than 90% of all amino acid residues in the most favored regions not containing outliers, were used. For ClapOBP27, YASARA created 16 homology models based on alignments with several already crystallized odorant binding proteins (PDB codes: 2ERB (Wogulis, Morgan, Ishida, Leal, & Wilson, 2006), 4PT1 (Zheng et al., 2015), 3OGN (Mao et al., 2010), 3CZ0 and 3D73 (Pesenti et al., 2009), 3R72, 3Q8I, 3K1E). A final hybrid model was formed based on the 3R72 model template (sequence identity 19.8%, sequence similarity 47.2%) with the inclusion of short template fragments from 3K1E and 4PT1. For ClapOBP02, YASARA created 20 models (PDB codes: 2JPO (Damberger, Ishida, Leal, & Wuthrich, 2007); 3D78, 3D73and 3CZ0 (Pesenti et al., 2009); 2QEB (Mans, Calvo, Ribeiro, & Andersen, 2007); 3VB1 and 3V2L (Ziemba, Murphy, Edlin, & Jones, 2013); 4PT1, 3OGN, 3K1E). A final hybrid model was formed based on 3VB1 (sequence identity 14.4%, sequence similarity 35.1%) with the inclusion of short template fragments from 2JPO and 4PT1. For ClapOBP20, YASARA created 22 structural models (PDB codes: 3S0D and 3S0G (Spinelli et al., 2012), 3R1P (Lagarde et al., 2011), 1C3Z (Rothemund, Liou, Davies, Krause, & Sonnichsen, 1999), 3DYE (Calvo, Mans, Ribeiro, & Andersen, 2009), 1DQE (Sandler, Nikonova, Leal, & Clardy, 2000), 3D78, 3R72, 3V2L, 3CZ0). A final hybrid model was formed based on 3V2L (sequence identity 20.0%, sequence similarity 38.0%) with the inclusion of short template fragments from 3R1P and 3DYE. The 3D structures of all the ligands were constructed with MOE (Molecular Operating Environment, 2013.08 (2016); Chemical Computing Group Inc., Montreal, QC, Canada). The putative binding sites were identified based on the structure of *Anopheles gambiae* odorant binding protein 20 with bound polyethylene glycol (PDB 3V2L (Ziemba et al., 2013)). In all three cases, a radius of 15 Å was applied to define the active site for docking using the coordinates of the following atoms as origin: ClapOBP20: L73‐CD1, ClapOBP02: L125‐CG, and ClapOBP27: L131‐CD1. Two side chains of each protein were considered to be flexible (ClapOBP27: F142, M34, ClapOBP02: Y70, Y126, ClapOBP20: F124, Y133). Docking studies were performed with GOLD using the ChemPLP scoring functions. For all other options, standard settings were applied.

### 2.10 Volatile analysis using GC‐MS

Freshly collected tree branches of either *B*. *rotundifolia* or *Salix* sp. without any treatment (*n* = 3), coronalon (0.1 mmol/L)‐treated plants (sprayed two times the evening before collection and let the leaves dry; n (birch) = 6; n (willow) = 5), and mechanically wounded leaves (scratched by a pattern wheel; *n* = 2) were sampled in the “field laboratory”. Coronalon is a synthetic 6‐ethyl indanoyl isoleucine conjugate that induces various plant stress responses including the induction of volatiles against herbivore attack (Schüler et al., 2004). The 25‐cm‐long treated or untreated branches of birch or willow were enclosed with polyethylene terephthalate (PET) foil (Toppits Bratschlauch, Minden, Germany). The volatile collection time was 6 hours. For volatile collection, push–pull systems were used. One system was equipped with two rotary vane pumps (model G 12/02 EB, Gardner Denver Thomas GmbH, Fürstenfeldbruck, Germany), one for providing fresh charcoal‐cleaned air (flow 1.0 L/min) and one for volatile collection (flow 0.9 L/min). The volatiles were collected on Porapack‐Q 80/100 mesh (20 mg, Supelco, Bellefonte, PA, USA) and eluted with 90 μl dichloromethane containing an internal standard (1‐bromodecane, 50 ng/μl, Fluka, Germany). Samples were kept at 4°C sealed in glass capillaries (capillary tubes for the determination of melting point, one end closed, Marienfeld GmbH & Co. KG, Lauda‐Königshofen, Germany) until measurement. The volatile bouquet was analyzed by GC‐MS. Therefore, a TRACE MS (Thermo Finnigan) device equipped with a ZB5 column (15 m, 0.25 mm I.D, 0.25 μm film thickness) was used with a 10‐m guard column (Phenomenex, Aschaffenburg, Germany). Mass spectra were measured in electron impact (EI) mode at 70 eV, 33–450 *m*/*z*. Volatiles were eluted under programmed conditions: 40°C (2 min isotherm), followed by heating at 10°C/min to 220°C and at 30°C/min to 280°C, using helium (1.5 ml/min) as the carrier gas. The GC injector (split ratio 1:7), transfer line, and ion source were set at 220, 280, and 200°C, respectively. The compounds were identified using authentical standards. The van den Dool and Kratz RI (Van den Dool & Kratz, 1963) calculated by MassFinder 4 software (Dr. Hochmuth (scientific consulting), 1999–2010, Hamburg, Germany, http://www.massfinder.com) was used to identify β‐bourbonene (RI 1384; ZB‐5 column). The values were compared with the literature values (RI 1384; DB5‐column (Telascrea et al., 2007) and RI 1385, HP‐5 column (Flamini, Cioni, Morelli, & Bader, 2007)). Additionally, β‐bourbonene is described for oils of *Mentha longifolia* (Adams, 2007) and *Mentha piperita* (Yadegarinia et al., 2006). The analysis of both essential oils and the coronalon‐treated willow sample resulted in identical mass spectra and retention times. Rate calculation was carried out using the peak areas of the volatiles relative to the peak area of the internal standard 1‐bromodecane. Average and standard error of the analyzed volatile composition of coronalon‐treated willow and birch branches were calculated. A Wilcoxon's rank‐sum test was used to compare the emission of volatiles between coronalon‐treated willow (*n* = 5) and birch (*n* = 6) branches (Supporting Information Table S7).

## 3 RESULTS

### 3.1 Host plant preference of Kazakh *C. lapponica*

Two‐choice assays were carried out to test adult beetles for their attraction and feeding preference toward host and nonhost leaves directly at the field site in Kazakhstan. As expected, beetles of the willow‐feeding population (WFP) significantly preferred willow over birch, whereas members of the birch‐feeding population (BFP) significantly preferred birch over willow leaves (*p* < 0.001; Table 1). To identify differences in the chemosensory repertoire at a transcriptional level, we have performed RNA‐seq experiments and differential expression analysis on the two *C. lapponica* populations.

**Table 1:** Feeding tests of adult *C. lapponica* beetles from either birch‐adapted (*N* = 58) or willow‐adapted (*N* = 14) individuals using host and nonhost leaves in two‐choice assays. *p*‐Values for significant differences between BFP and WFP were analyzed by chi‐square test

Leaves tested	Willow‐adapted *C. lapponica* beetles (*N* = 14)		Birch‐adapted *C. lapponica* beetles (*N* = 58)	
	Willow	Birch	Willow	Birch
Number of beetles that were attracted and fed	14	0	16	42
*p*‐Value	<0.001		<0.001	

### 3.2 Transcriptome library generation of Kazakh *C. lapponica*

For creating a catalog of chemosensory genes, we have sequenced cDNA derived from pooled *C. lapponica* individuals from different developmental stages as well as from antennae and legs of adult beetles of the WFP and BFP (for the external morphology of the chemosensory organs of the species, see Supporting Information Figure S1). The resulting raw sequence data are listed in Supporting Information Table S1. For our transcriptome reference libraries, we obtained 31,612 assembled cDNAs (contigs) with an average length of approx. 1,260 bp and an N50 length of 2,048 bp from the WFP and 34,154 contigs with an average length of approx. 1,166 bp and an N50 length of 1,904 bp from the BFP.

### 3.3 Identification of putative chemosensory receptor proteins in WFP and BFP of *C. lapponica*

The 114 identified putative binding proteins and receptors of the chemosensory system are listed in Table 2 for both *C. lapponica* populations.

**Table 2:** Number of identified chemosensory protein candidates from willow‐ or birch‐feeding *C. lapponica* beetles

	ORs	GRs	IRs	SNMPs	OBPs	CSPs
Species specific	31	8	12	4	32	12
Willow specific	7	—	2	—	—	—
Birch specific	3	—	3	—	—	—

We identified 38 ORs of *C. lapponica* in the WFP and 34 ORs in the BFP. Comparing the OR sequences of both populations, 31 ORs share high amino acid identities (30 sequences share ≥93% identity; due to a gap, *Clap*OR14 shares 82% identity) with a counterpart in the other population. Among them was also the universal odorant coreceptor, *Clap*ORco with 480 amino acids. Three ORs appeared to be BFP specific, and seven ORs were WFP specific with ≤55% identities (Table 2). Among all the total identified 41 ORs, eight ORs (ORco, 02, 04, 05, 12, 15, 16, and 19) were likely represented by full‐length proteins composed of 373 to 480 amino acids with 4–7 transmembrane domains (TMDs).

By sequence alignments, we observed that the region at the C‐terminus of *C. lapponica* ORs was more conserved than that at the N‐terminus. This conserved region is a loop of roughly 50 amino acids between the sixth and seventh alpha helix and contains three conspicuous motifs (Supporting Information Figure S2). These motifs are also known from other insect ORs presumably involved in protein–protein interactions (Benton et al., 2006; Miller & Tu, 2008).

**Figure 1:**
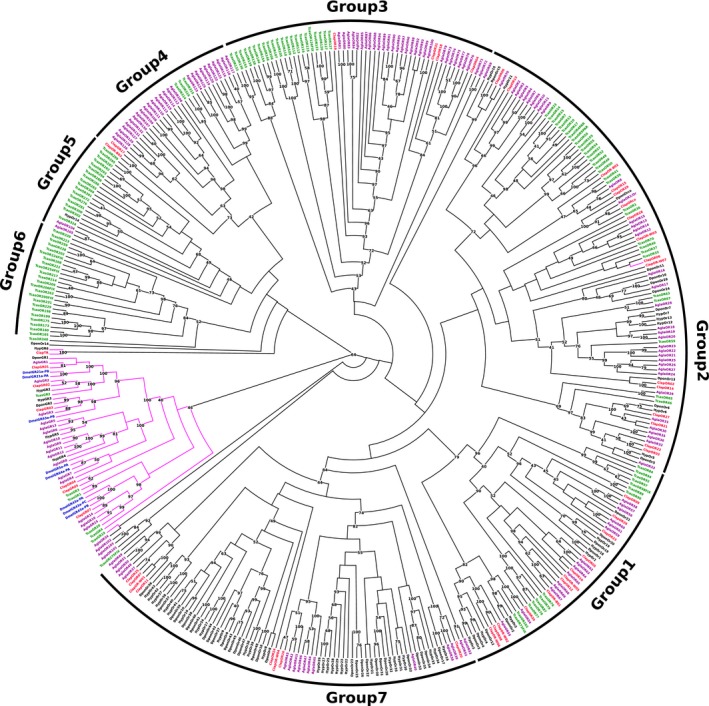
Phylogenetic tree of ORs and GRs. Blue: *D. melanogaster* (Dmel); green: *T*. *castaneum* (Tcas); black: *D. ponderosae* (Dpon) and *I. typographus* (Ityp); red: *C. lapponica* (Clap); purple: *A. glabripennis* (Agla). Seven subgroups 1–7 of ORs are identified. Numbers at nodes represent bootstrap values based on 100 replicates, which are shown when ≥40%

We identified eight GRs including one trehalose receptor, *Clap*TR. All of them were detected in both *C. lapponica* populations with high amino acid similarities (seven sequences share ≥96% identity; due to a gap, *Clap*GR05 shares 82% identity). Three candidates were represented as full length: *Clap*GR01 with 440 amino acids and eight predicted TMDs and *Clap*GR02 and *Clap*TR possess 385 (eight predicted TMDs) and 299 (seven predicted TMDs) amino acids, respectively. As insect ORs and GRs belong to one chemoreceptor superfamily (Robertson et al., 2003), the *C. lapponica* ORs and GRs were combined in our phylogenetic analysis (Figure 1). Except *Clap*GR06, *Clap*TR, and *Ityp*GR6, all GRs were grouped together, but only with a bootstrap value of 45%. *Clap*GR01, 05, and 07 clustered into a CO_2_ clade characterized by *Dmel*GR21a and *Dmel*GR63a (Kwon, Dahanukar, Weiss, & Carlson, 2007). *Clap*GR03 clustered with *Dmel*GR43a group. *Clap*TR and *Ityp*GR6 clustered together in one clade. *Clap*GR06 clustered next to the GR group, but in the OR group. As described in previous studies (Andersson et al., 2013; Engsontia et al., 2008), seven subgroups (named 1 to 7) of beetle ORs could be found.

**Figure 2:**
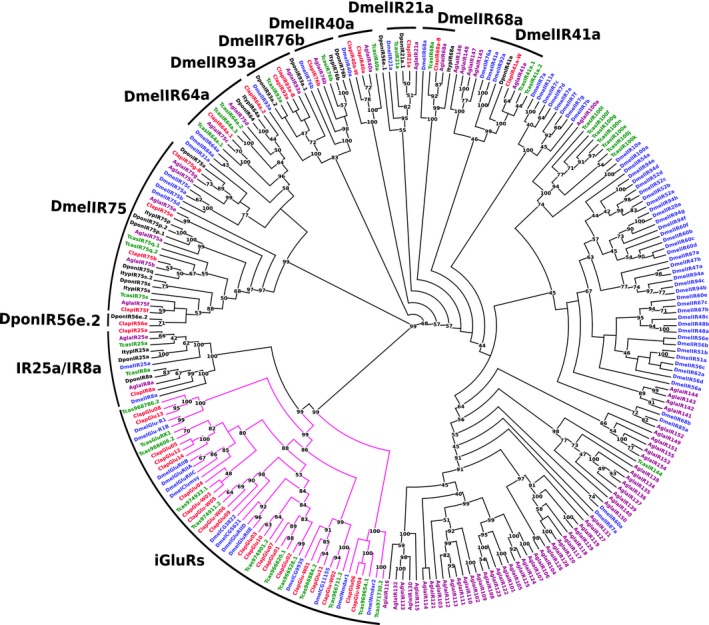
Phylogenetic tree of IRs and iGluRs. Blue: *D. melanogaster* (Dmel); green: *T*. *castaneum* (Tcas); black: *D. ponderosae* (Dpon) and *I. typographus* (Ityp); red: *C. lapponica* (Clap); purple: *A. glabripennis* (Agla). Magenta edges: iGluRs subgroup. Numbers at nodes represent bootstrap values based on 100 replicates, which are shown when ≥40%

IRs represent also membrane proteins, but unlike ORs and GRs, they include only three transmembrane domains (Silbering & Benton, 2010; Wicher, 2015). They are more closely related to ionotropic glutamate receptors (iGluRs) (Croset et al., 2010; Rytz et al., 2013). In order to distinguish IRs from iGluRs, we carried out phylogenetic analyses. Twenty putative receptors of *C. lapponica* clustered distinctly into the family of iGluRs and 17 into the family of IRs with a bootstrap value of 92% (Figure 2). Twelve of the 17 IRs share high amino acid identities (≥96%) in the coding sequence in both *C. lapponica* populations. The remaining IRs possessed low amino acid identities with ≤39% between the two populations and were therefore considered as population‐specific IRs (Table 2). *Clap*IR25a (924 amino acids) and *Clap*IR75b (626 amino acids) were full‐length proteins. Our phylogenetic analysis revealed that the IRs from *C. lapponica* could be divided into two general subgroups: coreceptor IRs and antennal IRs (Abuin et al., 2011; Croset et al., 2010). The candidates *Clap*IR25a and *Clap*IR8a clustered into coreceptor *Dmel*IR25a orthologs and *Dmel*IR8a orthologs, respectively, that are located in the clade of iGluRs (Rytz et al., 2013). The remaining 15 IRs of *C. lapponica* formed ten orthologous groups with other insect species in the subgroup of antennal IRs.

We identified four SNMPs. Except *Clap*SNMP2b, all of them represented full‐length proteins with 515–534 amino acids in each of the two *C. lapponica* populations. The amino acid identity of each matched candidate was very high (99%) between both populations. Our phylogenetic analysis revealed that the four *Clap*SNMPs were divided into two subgroups, SNMP group 1 and SNMP group 2 (Supporting Information Figure S3).

### 3.4 Identification of putative soluble proteins in WFP and BFP of *C. lapponica*

Based on our analysis, we identified a total of 32 OBPs in the sequence library of each of the *C. lapponica* populations. Sequence comparison showed that the putative OBPs from the two populations shared a sequence homology of more than 96%. Except *Clap*OBP12, all of the OBPs represented full‐length proteins. Despite conserved protein features, including a signal peptide, a six α‐helix domain and cysteine motifs, the *C. lapponica* OBP family members were divergent in terms of length (131–263 amino acids) and cysteine profiles.

**Figure 3:**
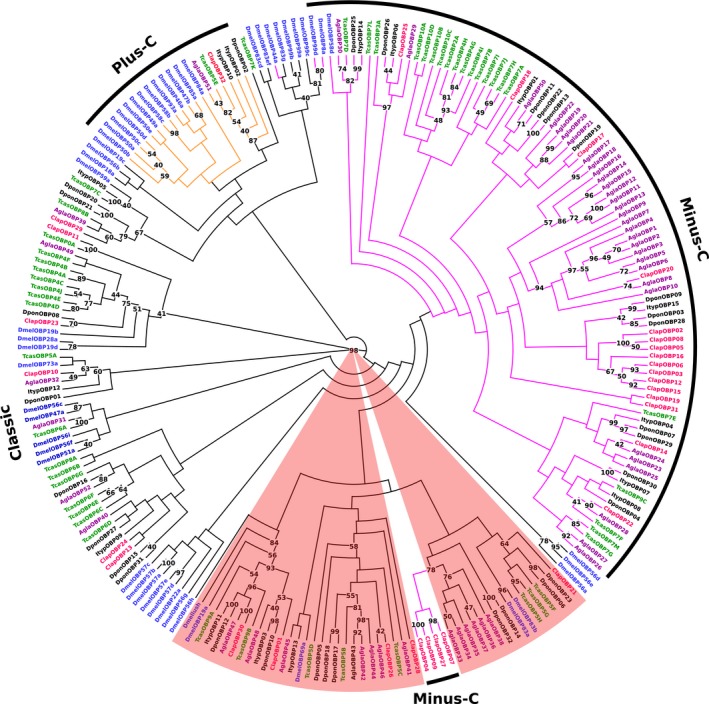
Phylogenetic tree of OBPs. Blue: *D. melanogaster* (Dmel); green: *T*. *castaneum* (Tcas); black: *D. ponderosae* (Dpon) and *I. typographus* (Ityp); red: *C. lapponica* (Clap); purple: *A. glabripennis* (Agla). Four subgroups of OBPs: classic (black edges), minus‐C (magenta edges), plus‐C (orange edges), and ABPII (shaded in brown) are identified. Numbers at nodes represent bootstrap values based on 100 replicates, which are shown when ≥40%

On the basis of distinctive structural features and phylogenetic relationships, we identified four main subgroups of OBPs: classic, antenna binding protein II (ABPII), plus‐C, and minus‐C (Figure 3). In accordance with previous phylogenetic analyses (Andersson et al., 2013; Dippel et al., 2014), we could show that the basal OBP group seems to be the classic, whereas all other groups were internal clades of this subfamily. In our tree, the subgroups ABPIIs and minus‐C OPBs appeared to have independent origins. Further, we found mostly lineage‐specific expansions, particularly in minus‐C and plus‐C subgroups. Only two classic OBP genes were found with clear orthologous relationships across the insects tested: *Obp29* and *Obp10*, a finding which is similar to (Sanchez‐Gracia, Vieira, & Rozas, 2009) that may indicate a conserved function for these genes.

In each of the two *C. lapponica* populations, we found six classic OBPs and five ABPIIs. The characteristic hallmarks of these proteins are six cysteine residues at conserved positions with a C‐pattern of C1X_23‐40_C2X_3_C3X_38‐44_C4X_8‐21_C5X_8_C6 (Supporting Information Figure S4A) (Xu et al., 2009). Unlike in *T. castaneum* or in *D. melanogaster*, we could not find an expansion of *C. lapponica* classic OBPs. Among the classic OBPs, the 243‐amino‐acid‐long *Clap*OBP29 featured a modified C‐pattern that had three additional cysteine residues instead of C1 in the above‐mentioned C‐pattern.

The subgroup of ABPIIs formed three clades. In one clade, the characterized OBP LUSH of *D. melanogaster*, crucial for binding pheromones and short‐chain alcohols among other compounds (Ader, Jones, & Lin, 2010; Laughlin et al., 2008; Xu, Atkinson, Jones, & Smith, 2005), was localized together with *Clap*OBP01 and *Clap*OBP30 (Figure 3, bootstrap value of 84%). Another ABPII clade contained four *C. lapponica* minus‐C OBPs.

In contrast to the classic OBPs, minus‐C OBPs lack the second and the fifth conserved cysteine residues (Fan, Francis, Liu, Chen, & Cheng, 2011). Twenty of all the predicted OBPs in *C. lapponica* comprised a motif of the minus‐C OBPs, C1X_28‐34_C2X_35‐39_C3X_16‐22_C_4_ (Supporting Information Figure S4B). Two of the minus‐C OBPs (*Clap*OBP06 262 amino acids, *Clap*OBP19 263 amino acids) contained a dimer minus‐C pattern. Most of the minus‐C OBPs were localized in two distinct clusters, and only a few were scattered across the phylogeny. Compared to the many minus‐C OBPs from *C. lapponica*,* D. melanogaster* possesses only a few members that fell mainly into a branch separated from the beetle sequences. Plus‐C OBPs, however, appeared to be more diverse in fruit fly, while in *C. lapponica*, only one sequence (*Clap*OBP32) was identified, which clustered together with other beetle sequences.

**Figure 4:**
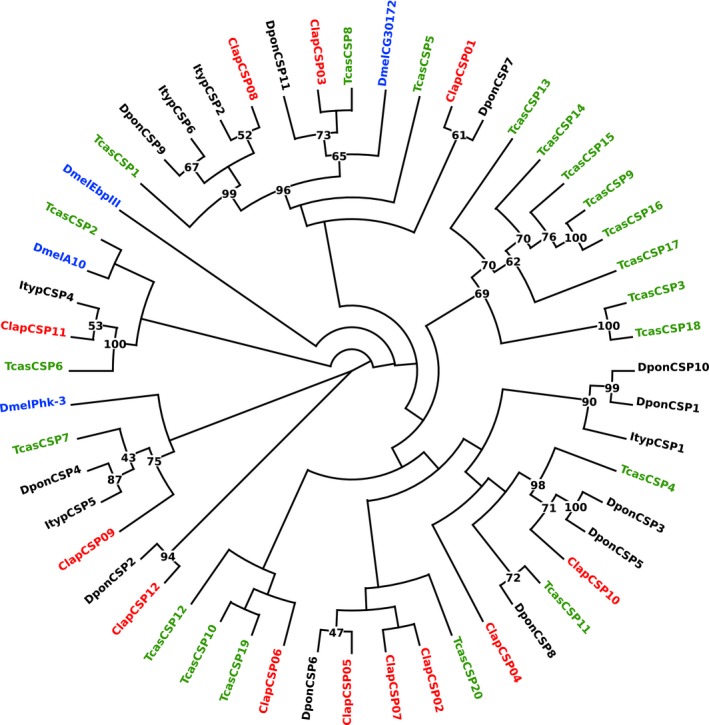
Phylogenetic tree of CSPs. Blue: *D. melanogaster* (Dmel); green: *T*. *castaneum* (Tcas); black: *D. ponderosae* (Dpon) and *I. typographus* (Ityp); red: *C. lapponica* (Clap). Numbers at nodes represent bootstrap values based on 100 replicates, which are shown when ≥40%

In each of the two *C. lapponica* populations, we identified 12 CSPs. The sequence comparison of both CSP sets revealed that the CSP pairs shared at least 93% amino acid identity. All CSP candidates represented full‐length proteins showing the conserved C‐pattern, C1X_6_C2X_18_C3X_2_C4 (Supporting Information Figure S4C) (Xu et al., 2009). Among all the *C. lapponica* CSPs, the candidate *Clap*CSP11 contained with 283 amino acids the longest amino acid sequence. Bootstrapping (Figure 4) revealed a clade of CSPs with a value of 100% that included only the longest CSPs, *Clap*CSP11, *Tcas*CSP6 (251 amino acids), and *Ityp*CSP4 (214 amino acids).

### 3.5 Differential expression of chemosensory genes in the antennae and legs of birch‐ and willow‐adapted *C. lapponica*

In total, we have identified 114 unique sequences encoding putative members of six chemosensory protein families from both populations of *C. lapponica*. We have filtered out 80 from the total of 114 sequences that were expressed in least one library with a CPM ≥ 1 for the following analyses (Supporting Information Table S2). Among the 33 discarded sequences were also those putative ORs and IRs that were initially identified as population specific. As they exhibited CPM < 1, they may not play a role in the adult but in other developmental stages and/or could also be expressed in internal body tissues such as gut or fat body as proposed from other studies (Engsontia et al., 2008; Koenig et al., 2015).

In order to obtain a general overview of the differential expression in the antennae and legs of beetles from WFP and BFP, respectively, we first compared the CPM of all sequences among all RNA‐seq libraries. We identified 238 contigs as significantly differentially expressed in antennae and 374 contigs as significantly differentially expressed in legs between WFP and BFP (Supporting Information Tables [Link], [Link], [Link], [Link]). Among these sequences, we found candidates of our already annotated chemosensory genes. In addition, GO annotation indicated also contigs with GO terms related to enzymatic activity, such as oxidoreductase activity (e.g., by cytochrome P450s), hydrolase activity (e.g., by esterases), and transferase activity (e.g., by glutathione S‐transferases) (Supporting Information Figures S5 and S6).

**Figure 5:**
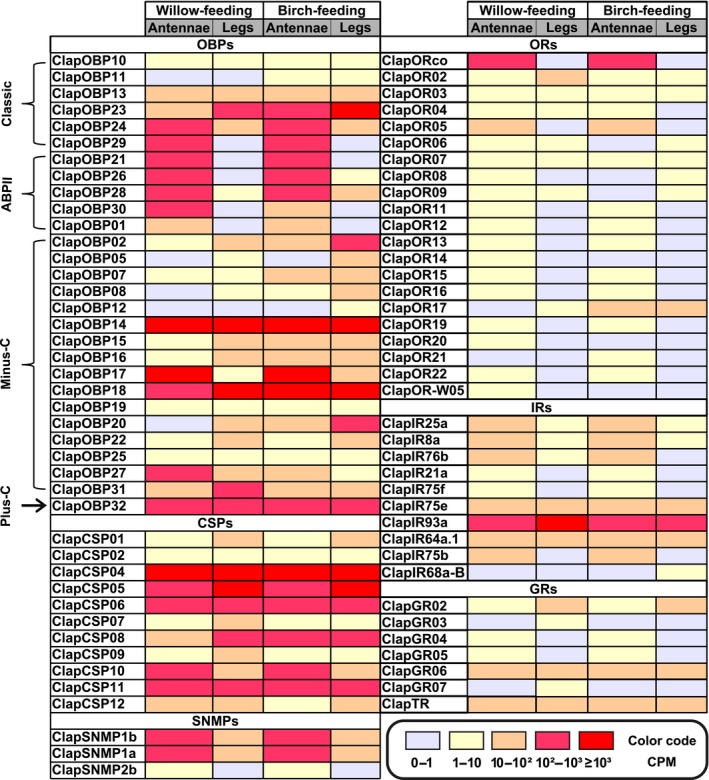
Expression profiles of 80 unique genes from six chemoreception families: OBPs, SNMPs, CSPs, ORs, IRs, and GRs from WFP or BFP *C. lapponica* in antennae and legs based on CPM values. RNA‐seq reads were normalized to the effective library size. The CPM value of each tissue is derived from four replicates: two in male and two in female, respectively. Candidate chemosensory genes were chosen according to their CPM values of ≥1 in at least one of the examined tissues. OBPs are divided into four subgroups: classic OBPs, ABPIIs, minus‐C OBPs, and plus‐C OBPs

**Figure 6:**
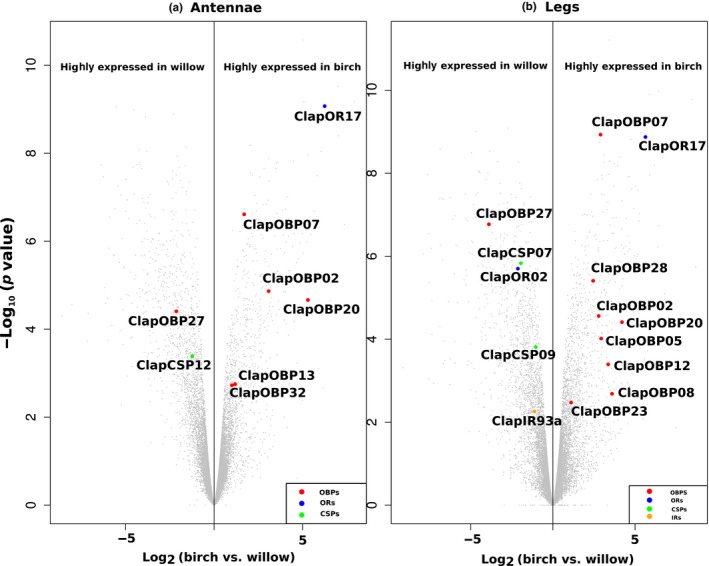
Volcano plot showing significant differences in the expression level of all chemoreception genes of *C. lapponica* when comparing willow and birch populations. Gray points: differently expressed genes between two populations of *C. lapponica*. Significantly different: log_2_fold ≥ 1, *p*‐value ≤ 0.05, and FDR ≤ 0.05

Focusing on the chemosensory genes, genes encoding OBPs, CSPs, and SNMPs were in general higher expressed than the receptor genes for ORs, GRs, and IRs (Figure 5). In detail, we detected in the antennae of the WFP for *Clap*OBP27 and *Clap*CSP12 higher transcript levels than in the antennae of the BFP (Figure 6a). In the antennae of the BFP, *Clap*OR17, and five OBPs (*Clap*OBP02, 07, 13, 20, 32) exhibited higher mRNA levels compared to the antennae of the WFP.

The comparative analysis of legs between the two populations revealed that *Clap*OBP13, 32 and *Clap*CSP12 were differentially expressed only in antennae but not in the legs, while all other chemosensory genes exhibited a differential mRNA level in both organs (Figure 6b). In addition, we observed a significantly higher expression of one OR, one IR, and two CSPs in the legs of WFP compared to the samples of the BFP. In the legs of the BFP, additionally five OBPs were higher expressed compared to the legs of the WFP (Figure 6b).

Analyzing the selected chemosensory genes in antennae and legs of additional individuals from BFP and WFP via qRT‐PCR confirmed the differential expression of *Clap*OR17 and OBP02, 20, and 27 as found by RNA‐seq (Supporting Information Figure S7A,B). Including a geographically distant WFP from northern Finland, we confirmed reduced expression for *Clap*OR17 (in antennae and legs) and *Clap*OBP02 (in legs) as well as increased expression of *Clap*OBP27 in comparison with Kazakh BFP (Supporting Information Figure S7C). Interestingly, the mRNA level of *Clap*OBP20 in both organs was similar to the expression pattern of Kazakh BFP.

### 3.6 Characterization of the differentially expressed genes

To further characterize the chemosensory genes which displayed a differential expression associated with the scenario of host plant shift, we analyzed their phylogenetic relationships. Among all tissue samples, *Clap*OR02 displayed the highest expression in the legs of the WFP (Figure 5; Supporting Information Table S2). Phylogenetically, *Clap*OR02 is a member of the OR group 2. *Clap*OR17 was significantly higher expressed in the BFP, in both antennae and legs, compared to samples from the WFP. *Clap*OR17 had similar CPM values in the antennae and legs of the BFP and exhibited the second highest expression level among all identified ORs after *Clap*ORco (Figure 5; Supporting Information Table S2). Our phylogenetic analysis revealed the clustering of *Clap*OR17 into the subgroup 4 with relationship to several *Agla*ORs (Figure 1).

The only differentially expressed IR in our study was *Clap*IR93a. Among all identified IR genes, *Clap*IR93a exhibited the highest expression in both legs and antennae of *C. lapponica* (Figure 5). As it clustered together with IR93a from *D. melanogaster* (Figure 2) that has been shown to mediate both humidity and temperature preference in the flies (Enjin et al., 2016), we propose a similar function in *C. lapponica*.

In the antennae, we found OBP genes with a significant differential expression: one plus‐C OBP, *Clap*OBP32, one classic OBP, *Clap*OBP13, and four minus‐C OBPs (*Clap*OBP02, 07, 20, 27) (Figure 6a). Differential expression between antennae and legs within one population showed that most of these OBPs were also expressed in the legs (Figure 6b; Supporting Information Figure S8). *Clap*OBP02 and 20, for example, were at least six times higher expressed in the legs than in the antennae of the beetles from both populations (Supporting Information Figure S8; Supporting Information Table S2). In contrast, *Clap*OBP27 showed higher expression in the antennae in both populations (Figure 5; Supporting Information Table S2). This minus‐C OBP formed together with three more *Clap*OBPs a cluster within the ABPII group (Figure 3, bootstrap value of 78%).

The OBPs particularly upregulated in the legs of the BFP were minus‐C OBPs, with the exception of *Clap*OBP28. *Clap*OBP28 has been classified as a candidate of the subfamily of ABPIIs found to be highly expressed in antennae in other insects, for example, in *Phyllotreta striolata* (Wu et al., 2016). Accordingly, although *ClapOBP28* showed a differential expression in the legs of the two populations, its highest expression has been detected in the antennae with CPM of 145 and 169 in BFP or WFP, respectively (Figure 5; Supporting Information Table S2). *Clap*CSP12, upregulated in the antennae of WFP, seems to have an ortholog in *D. ponderosae*, which functionally has not yet been characterized (Figure 4).

### 3.7 Volatile composition of willow and birch

**Figure 7:**
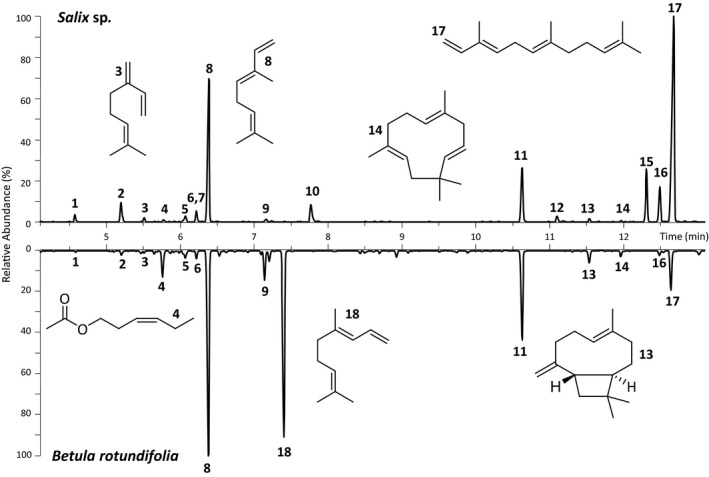
Gas chromatograms of the volatile composition of Kazakh *Salix* sp. and *Betula rotundifolia* colonized *C. lapponica*. The plants were treated with coronalon (0.1 mmol/L). The volatiles were collected for 6 hr on Porapack‐Q using a push–pull system. 1, α‐pinene; 2, sabinene; 3, myrcene; 4, *cis*‐3‐hexenyl acetate; 5, eucalyptol; 6, (*Z*)‐β‐ocimene; 7, salicylaldehyde; 8, (*E*)‐β‐ocimene; 9, linalool; 10, (*E*)‐myroxide; 11, 1‐bromodecane (internal standard); 12, β‐bourbonene; 13, β‐caryophyllene; 14, α‐humulene; 15, germacrene D; 16, (*Z,E*)‐α‐farnesene; 17, (*E,E*)‐α‐farnesene; 18, DMNT (4,8‐dimethylnona‐1,3,7‐triene) (see also Supporting Information Table S7)

Volatiles were identified from the two host plants via GC‐MS to narrow down the number of putative ligands for differentially expressed chemosensory proteins. We compared the volatile bouquet of untreated leaves with the bouquet of leaves treated with coronalon (induces various plant stress responses, e.g., the induction of volatiles against herbivore attack) and with the bouquet of mechanically wounded leaves. The volatile emission was much lower in the untreated than in the coronalon‐treated or wounded leaf material of both plant species (Supporting Information Table S7). With one exception, both coronalon‐treated plant species shared a qualitatively similar volatile pattern. However, they appeared to differ in the quantity of their emitted compounds. Willow leaves released as major volatile compound more (*E,E*)‐α‐farnesene (17), and as minor components e.g., more (*Z,E*)‐α‐farnesene (16) and *E*‐myroxide (10) than the birch leaves (Figure 7). A minor amount of salicylaldehyde has been detected exclusively in willow. By analyzing birch volatile emission, we found an increased release of DMNT (4,8‐dimethylnona‐1,3,7‐triene) (18) as a major component, as well as of linalool (9), β‐caryophyllene (13) and α‐humulene (14) as minor components in comparison with the volatile emission from willow.

Artificially wounded leaves of the two plant species produced more green leaf volatiles than the coronalon‐treated or untreated leaf samples. Both species had this pattern in common, but willow released higher amounts of green leaf volatiles than birch (Supporting Information Table S7). The identified volatiles represent a basis to conduct further physiological and biochemical experiments or to compute ligand binding abilities of chemosensory molecules.

### 3.8 Structural modeling of ClapOBP02, 20, and 27 and ligand docking of selected host plant volatiles

**Figure 8:**
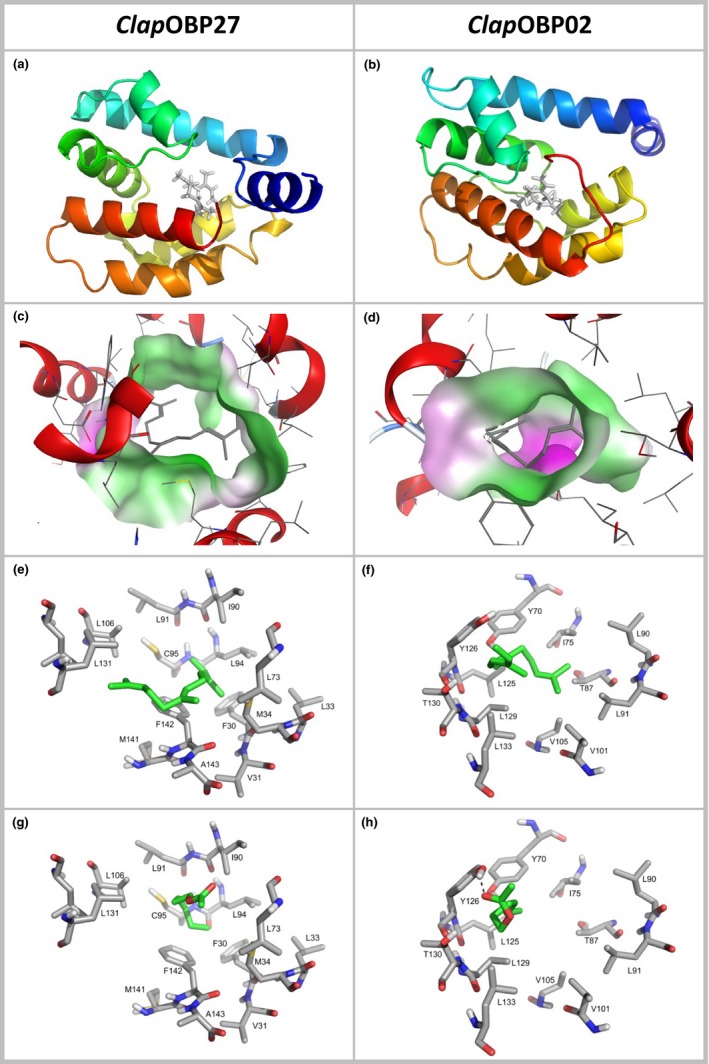
Comparison between tertiary structure models and docking studies of the minus‐C OBPs, *Clap* OBP27 (upregulated in willow feeders), and *Clap* OBP02 (upregulated in birch feeders). (a,b) Rainbow representation of the 3D models (N‐terminus dark blue, C‐terminus red); (c,d) graphical representation of the lipophilic (green) and hydrophilic (red) potential of the binding site of the ligands with docked (*E,E*)‐α‐farnesene; (e,f) details of the interactions of (*E,E*)‐α‐farnesene in the binding site for each protein; (g,h) details of the interactions of *cis*‐3‐hexenyl acetate in the binding site for each protein. Ligands are highlighted by green carbon atoms

To analyze the ligand binding properties of the predicted chemosensory proteins, we have computed binding abilities of the three most differentially expressed OBPs in antennae. The structural models revealed that all three OBPs formed at least seven α‐helices which define the internal ligand binding pocket of each protein (Figure 8a,b; Supporting Information Figure S9). In order to evaluate the ligand binding affinities, we have calculated fitness score values for selected willow and birch volatiles which are listed for the most favored docking poses of each ligand in all three proteins (Supporting Information Table S8). These fitness score values are based on empirical functions and approximately reflect the interaction energies between the ligands and the protein. In general, the more positive these values are, the higher should be the affinity. From all three binding proteins, *Clap*OBP27 seems to be favored to bind all the odorants tested, especially the hydrophobic terpenoids.

Even though the three proteins had a folding pattern in the central core in common, they differed from each other regarding size and lipophilic/hydrophilic surface potential of the ligand binding cavities (Figure 8c,d; Supporting Information Figure S9). *Clap*OBP27 possessed the most distinct hydrophobic ligand binding pocket. This was also reflected by the putative binding of the hydrophobic ligands, such as (*E,E*)‐α‐farnesene or (*Z,E*)‐α‐farnesene, showing the highest affinity (Figure 8e,f; Supporting Information Figure S9; Supporting Information Table S8). In comparison, the two other OBPs contained more amino residues whose side chains were capable of contributing to hydrogen bonds in the ligand cavity, with consequences for the ligand binding abilities of the proteins. In the structure of *Clap*OBP02, the carbonyl group of *cis*‐3‐hexenyl acetate, for example, forms hydrogen bonds to the hydroxyl groups of the tyrosine side chains of Y70 and 126 and in the structure of *Clap*OBP20 with H32 (Figure 8g,h; Supporting Information Figure S9). Similar results were obtained for other ligands with hydrophilic moieties like salicylaldehyde or methyl salicylate. In summary, the 3D protein models and docking studies demonstrated that all tested volatiles may bind to the OBPs with varying affinity due to individual differences in polarity and the architecture of the ligand binding cavities.

## 4 DISCUSSION

In our study, we highlight the involvement of olfactory related genes (OR, OBP) underlying host plant shifts and thus exposure to different odor environments in a specialized herbivorous beetle. Within the peripheral chemosensory system, relatively minor and nonrandom changes in a subset of chemosensory genes contribute to population divergence in *C. lapponica* with respect to their two different hosts.

In the context of olfactory processing, ORs residing in the peripheral neuronal membranes are crucial to transduce ligand binding into a signaling cascade to the central nerve system of an insect. By comparing the antennae of the two populations, we found that only one OR, namely *Clap*OR17, was significantly higher expressed (75 times) in the BFP than in the WFP. Even though a function cannot be predicted from our studies, it is likely that this OR contributes to the attraction of birch as novel host plant.

Among the OBPs upregulated in the antennae of the BFP compared to the antennae of the WFP, we found one classic, one plus‐C, and three minus‐C OBPs. The differences were most clearly seen in the mRNA levels of the minus‐C *Clap*OBP02 and *Clap*OBP20 with an upregulation of eight times and 28 times, respectively. From the structural modeling, we could infer differences in the ligand binding abilities among the binding proteins. Due to the higher polarity of their cavities, *Clap*OBP02 and *Clap*OBP20 could facilitate the interaction with more hydrophilic compounds. With the exception of the classic and the plus‐C OBP, all antennal upregulated minus‐C OBP genes displayed also significant upregulation in the legs. Even five more minus‐C OBP genes were found being upregulated in the birch feeders’ legs compared to the willow feeders’ legs. Although OBPs are also found in the legs and antennae of other beetles (Dippel et al., 2014), they could fulfill different functions in the two organs. This scenario could also be true for the high expression of *Clap*OBP20 found in the Finnish WFP, a result opposing the data from the Kazakh WFP. Variability in the host chemistry existing among species within the genus *Salix* (Nyman & Julkunen‐Tiitto, 2005) could contribute to the differences in the gene expression among WFPs. In combination with the beetles’ genetic distinctiveness over wide geographic distances (Mardulyn et al., 2011), it is not surprising that the expression of a few chemosensory genes differs between populations over such a large geographic distance, even if they are adapted to the same host genus (but not species). Therefore, future studies are encouraged to construct large‐scale phylogeographic analyses of chemosensory gene expression in relation to host chemistry.

Among all binding proteins, in particular, the minus‐C OBP genes in *C. lapponica* have experienced an expansion, presumably due to gene duplication events, compared to the four minus‐C OBP genes in *D. melanogaster*. Expansion of the minus‐C OBP subfamily has been described from several other herbivorous beetle species (Andersson et al., 2013; Li et al., 2015; McKenna et al., 2016; Wu et al., 2016; Zhang et al., 2016). Furthermore, due to the lack of a third disulfide bridge stabilizing the 3D structure, the minus‐C OBP may have the capability to bind different compounds with various functional groups (Schwaighofer et al., 2014). Based on our results, we speculate that OBPs could contribute to an escape of herbivorous insects from chemical host constraints, mainly due to the large number of different candidates in *C. lapponica* as well as the structural properties of these proteins.

OBPs might, however, also contribute to diet conservatism. For example, in the antennae of the WFP, the minus‐C *Clap*OBP27 was four times higher expressed than in the same organ of BFP. Phylogenetically, *Clap*OBP27 clusters within the group of ABPII together with *Dmel*OBP83a and *Dmel*OBP83b, whose ligands have not been identified. However, a homologous protein, *Ccap*OBP83a‐2 from the Mediterranean fruit fly, *Ceratitis capitate*, displays a high affinity toward (*E,E*)‐α‐farnesene (Siciliano et al., 2014). Together with the high binding affinity calculated from our modeling, (*E,E*)‐α‐farnesene may be anticipated as one potential ligand for *Clap*OBP27, which has to be experimentally corroborated.

In order to identify potential ligands for the chemosensory proteins of adult *C. lapponica*, we have analyzed volatiles emitted from either *Salix* sp. or *Betula rotundifolia* leaves. Although both species have most components of the measured volatile bouquet in common (with the exception of salicylaldehyde), the quantitative ratio between the components was characteristic of each host plant. Among all the identified volatiles, willow produced, for example, higher amounts of (*E,E*)‐α‐farnesene, while birch released more DMNT. These are possible olfactory ligands that can be used by *C. lapponica* beetles to discriminate between the two different host plant species. The similarity in volatile composition and the fact that both willows and birches occur frequently together in the same habitat (Fatouros, Hilker, & Gross, 2006; Gross, Fatouros, & Hilker, 2004) may have favored initial host plant shift from Salicaceae to Betulaceae accompanied by the change in the expression of chemosensory genes. Thus, when comparing the volatile composition of the host plants, it seems reasonable to assume that *C. lapponica* shifted host to a chemically similar plant species—via an “olfactory bridge”.

Interpopulation variations in olfactory genes that modulate phenotypic plasticity in host plant use are known from the stem borer *Sesamia nonagrioides* (Glaser et al., 2015) and *Drosophila* flies (Crowley‐Gall et al., 2016). In the latter species, RNA‐seq analyses comparing different cacti‐adapted populations of *Drosophila mojavensis* demonstrated that changes in host use were accompanied by changes in the olfactory system including the expression profile of ORs in adult heads. Here, we add an example from the most diversified insect class, the beetles (Coleoptera). Given the selective feeding choice and differential expression of chemosensory genes, we suggest that the host plant shift of *C. lapponica* seems to have occurred through a loss of host preference, or alternatively a biotic selection pressure to colonize other hosts and an obvious tolerance to the phytochemicals of birch accompanied by the modulation of mainly ORs and OBPs.

In addition to the ORs, other peripherally localized chemoreceptor families could also influence host plant choice of *C. lapponica*. However, we did not detect significant differences in the expression of, for example, GRs when comparing WFPs and BFPs. This lack is surprising as many nonvolatile compounds, such as salicin and other salicinoids, do differ among willow species and are absent in birch species (Zverev, Kozlov, & Zvereva, 2017). The following reasons may explain this expression pattern: (a) Subtle differences in GR expression seem sufficient for *C. lapponica* to distinguish willow from birch based on nonvolatile compounds; the generally very low expression levels of insect GRs as found here and elsewhere (Missbach et al., 2014) may support this notion; alternatively, changes in the GR structure may modulate ligand binding properties and efficacy; (b) the same GRs may act together in different combinations to modulate ligand specificity; and (c) differential signaling cascades activated by the same GR(s) and/or differential downstream processes in GRNs to higher brain centers influence whether ligands are perceived as stimulant or deterrent (Wright, 2016). These aspects should be examined in the future.

When we compared differentially expressed genes between WFP and BFP, cytochrome P450s, esterases and glutathione S‐transferases were also differentially expressed in antennae and legs. These typical detoxification enzymes are also known to contribute to odorant modification and/or odorant degradation (Chertemps et al., 2012; Maibeche‐Coisne, Nikonov, Ishida, Jacquin‐Joly, & Leal, 2004; Mamidala et al., 2013; Pottier et al., 2012; Younus et al., 2017). For example, the P450 CYP345E2 highly expressed in the antennae of *D. ponderosae* catalyzed the epoxidation or hydroxylation of several pine host monoterpene volatiles (Keeling et al., 2013). Hence, these proteins might modulate ligand availability and the saturation of chemoreceptors and could thus represent additional candidates influencing host plant selection by insects.

## CONFLICT OF INTEREST

None declared.

## AUTHOR CONTRIBUTIONS

DW, JMP, WBo, and AB designed the research; DW, SP, MK, MG, WBr, JMP, and AB performed the research; DW, SP, and WBr analyzed the data; DW, SP, WBo, and AB wrote the manuscript.

## DATA ACCESSIBILITY

The raw sequence data are stored in the Sequence Read Archive (SRA) of the National Center for Biotechnology Information (NCBI) with the Accession Number SUB3612888. The corresponding BioProject is PRJNA418071. This Transcriptome Shotgun Assembly project has been deposited at DDBJ/EMBL/GenBankunder the accession GGOB00000000. The version described in this paper is the first version, GGOB01000000.
