# Supplementary material for: A subset of chemosensory genes differs between two populations of a specialized leaf beetle after host plant shift
Source: Ecol Evol. 2018 Jul 20;8(16):8055–75. doi: 10.1002/ece3.4246 (PMC6145003; doi:10.1002/ece3.4246)
Supplement: Supplementary file 1 [file ECE3-8-8055-s001.docx]

Supporting information: Figures

**A subset of chemosensory genes differs between two populations of a specialized leaf beetle after host plant shift**

Ding Wang^1^, Stefan Pentzold^1^, Maritta Kunert^1^, Marco Groth^2^, Wolfgang Brandt^3^, Jacques M. Pasteels^4^, Wilhelm Boland^1^, Antje Burse^1*^

^1^Max Planck Institute for Chemical Ecology, Department of Bioorganic Chemistry, Hans-Knöll-Str. 8, D-07745 Jena, Germany

^2^ Leibniz Institute on Aging – Fritz Lipmann Institute, Beutenbergstraße 11, D-07745 Jena, Germany

^3^ Leibniz Institute of Plant Biochemistry, Weinberg 3, D-06120 Halle (Saale), Germany

^4^ Université Libre de Bruxelles, Department of Biology, Av. F.D. Roosevelt, 50, B-1050 Brussels, Belgium

**SUPPORTING INFORMATION**

**Table S1.** Raw RNA-seq data information. The table shows RNA-seq samples, number of reads and sequencing information.

**Table S2.** The expression profiles of 114 unique chemoreception genes from males and females, antennae and legs of willow-feeding and birch-feeding *C. lapponica.* The table shows the expression level of CPM values using R package edgeR, detailed description in method.

**Table S3.** Gene ontology results of significant (log2fold≥1, P-value≤0.05 and FDR≤0.05) differentially expressed genes in the antennae. The table shows the annotation of higher expressed genes in the antennae of the willow-feeding population.

**Table S4.** Gene ontology results of significant (log2fold≥1, P-value≤0.05 and FDR≤0.05) differentially expressed genes in the antennae. The table shows the annotation of higher expressed genes in the antennae of the birch-feeding population.

**Table S5.** Gene ontology results of significant (log2fold≥1, P-value≤0.05 and FDR≤0.05) differentially expressed genes in the legs. The table shows the annotation of higher expressed genes in the legs of the willow-feeding population.

**Table S6.** Gene ontology results of significant (log2fold≥1, P-value≤0.05 and FDR≤0.05) differentially expressed genes in the legs. The table shows the annotation of higher expressed genes in the legs of the birch-feeding population.

**Table S7.** Comparison of the volatile composition measured by GC-MS of the host plants colonized by the two *C. lapponica* populations. Untreated control plants, coronalon (0.1 mmol/l) treated and mechanical wounded (pattern wheel) plants were analyzed. The volatile content was divided into three groups: main components, minor components and traces. The ratio to the internal standard 1-Bromodecane (50 ng/µl) was used for calculation (main component > 1.5; minor component ≥ 0.15; trace < 0.15). Detailed statistic values for coronalon treated plants were given in worksheet 2-5.

**Table S8.** Affinities of minus-C OBPs for putative plant-derived ligands after docking studies.

**Table S9.** Output tables of EdgeR including p-values, false discovery rate (FDR) and log2-fold change.

**Table S10**. Primers for qRT-PCR analysis used in this study.

**Supporting Figures.** The file contains scanning electron micrographs of the external morphology of the chemosensory organs (**S1**), the motif analysis of C-termini of ORs identified from *C. lapponica* (**S2**), a phylogenetic tree of SNMPs (**S3**), multiple protein sequence alignments of OBPs and CSPs (**S4**), Gene ontology results in molecular function GO categories of antennae (**S5**) or legs (**S6**), differential expression of selected chemosensory genes from *C. lapponica* Kazakh and Finish populations via qRT-PCR (**S7**), significant differential expression of chemosensory genes in legs compared to antennae from birch or willow-feeding populations of *C. lapponica* (**S8**), and the tertiary structure model and docking studies of the minus-C OBP *Clap*OBP20 (**S9**).

**Supporting Data set.** The fasta-file contains the sequences of OBPs, CSPs, SNMPs, ORs, GRs, IRs and iGluRs from four insect species used for generating phylogenetic trees.

**
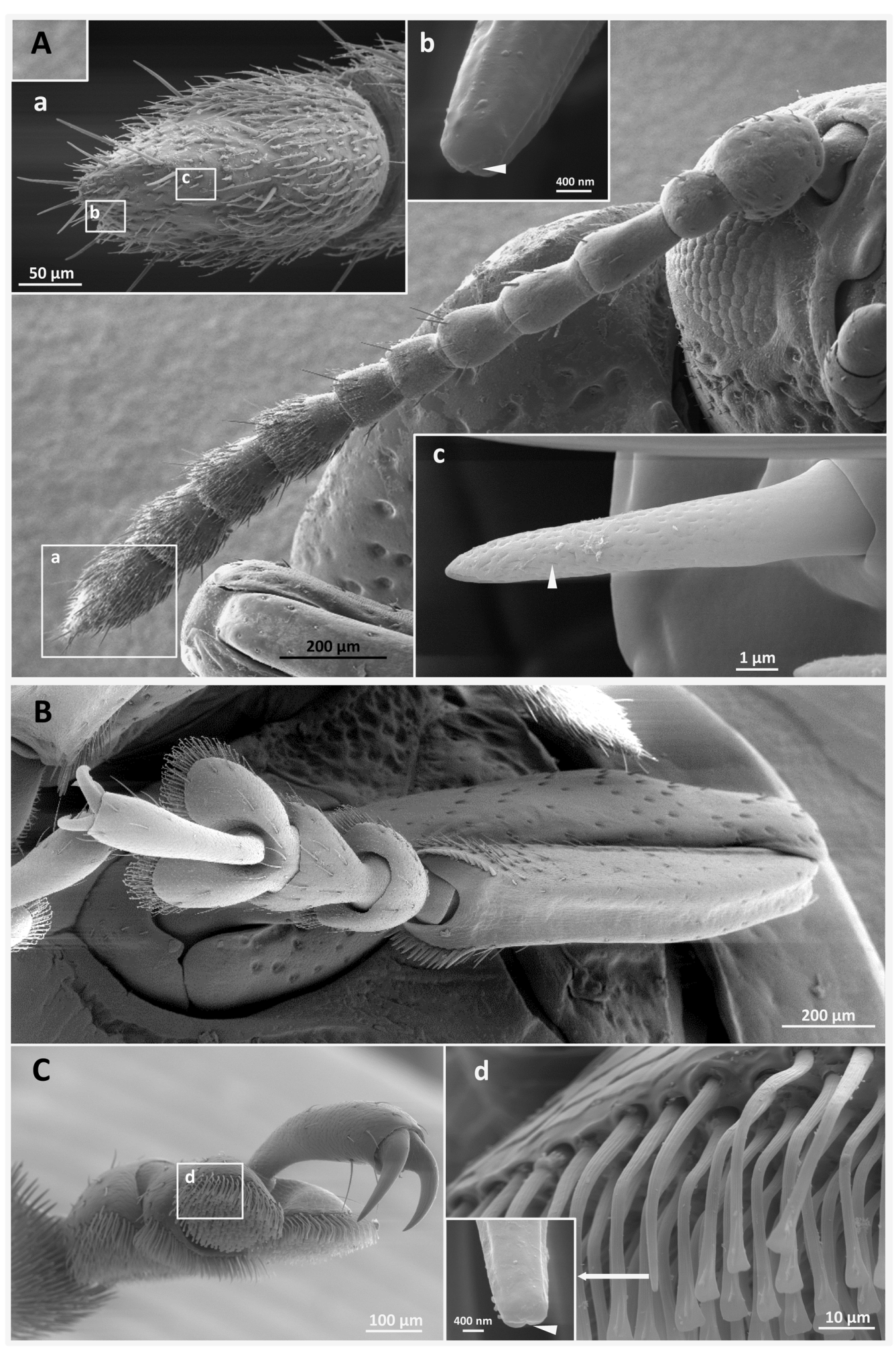
**

**Figure S1. Scanning electron micrographs of the external morphology of the chemosensory organs of a birch-feeding *C. lapponica* female** (three days after eclosion). **A**, Filiforme antenna; **a**, tip of the ninth flagellomer showing diversity of sensilla; **b**, tip of an antennal sensillum showing terminal pore (triangle); **c**, multiporous sensillum basiconicum; **B**, dorsal view of a leg; **C**, ventral view of the tarsus, **d**, tip of a tarsal sensillum showing terminal pore (triangle). The animals were fixed with 2.5 % (v/v) glutaraldehyde in cacodylate buffer for 60 min. Afterwards the samples were washed three times for 10 min with cacodylate buffer and dehydrated in ascending ethanol concentrations (30, 50, 70, 90 and 100%) for 20 min each. Subsequently, the samples were critical-point dried using liquid CO_2_ and sputter coated with gold (thickness approx. 4 nm) using a SCD005 sputter coater (BAL-TEC, Liechtenstein) to avoid surface charging. Finally the specimens were investigated with a field emission scanning electron microscope LEO-1530 Gemini (Carl Zeiss NTS GmbH, Oberkochen, Germany).


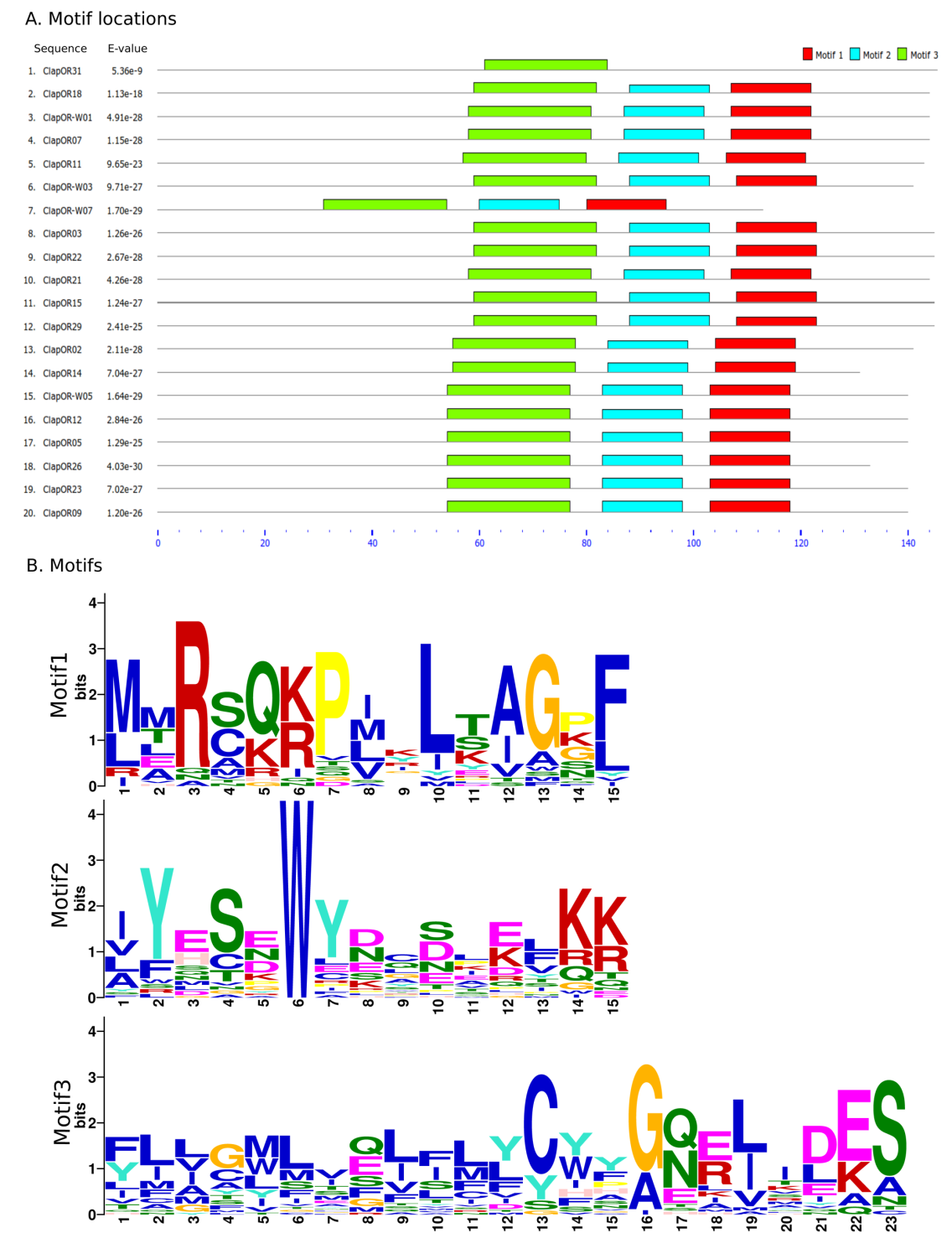


**Figure S2. Motif analysis of C-termini of ORs from *C. lapponica.*** Only the last 140 amino acids from C-terminus of population-specific ORs and longest ORs between willow-feeding and birch-feeding *C. lapponica* were analysed by applying of MEME 4.11.2 online software (<http://meme-suite.org/tools/meme>) with default parameter.


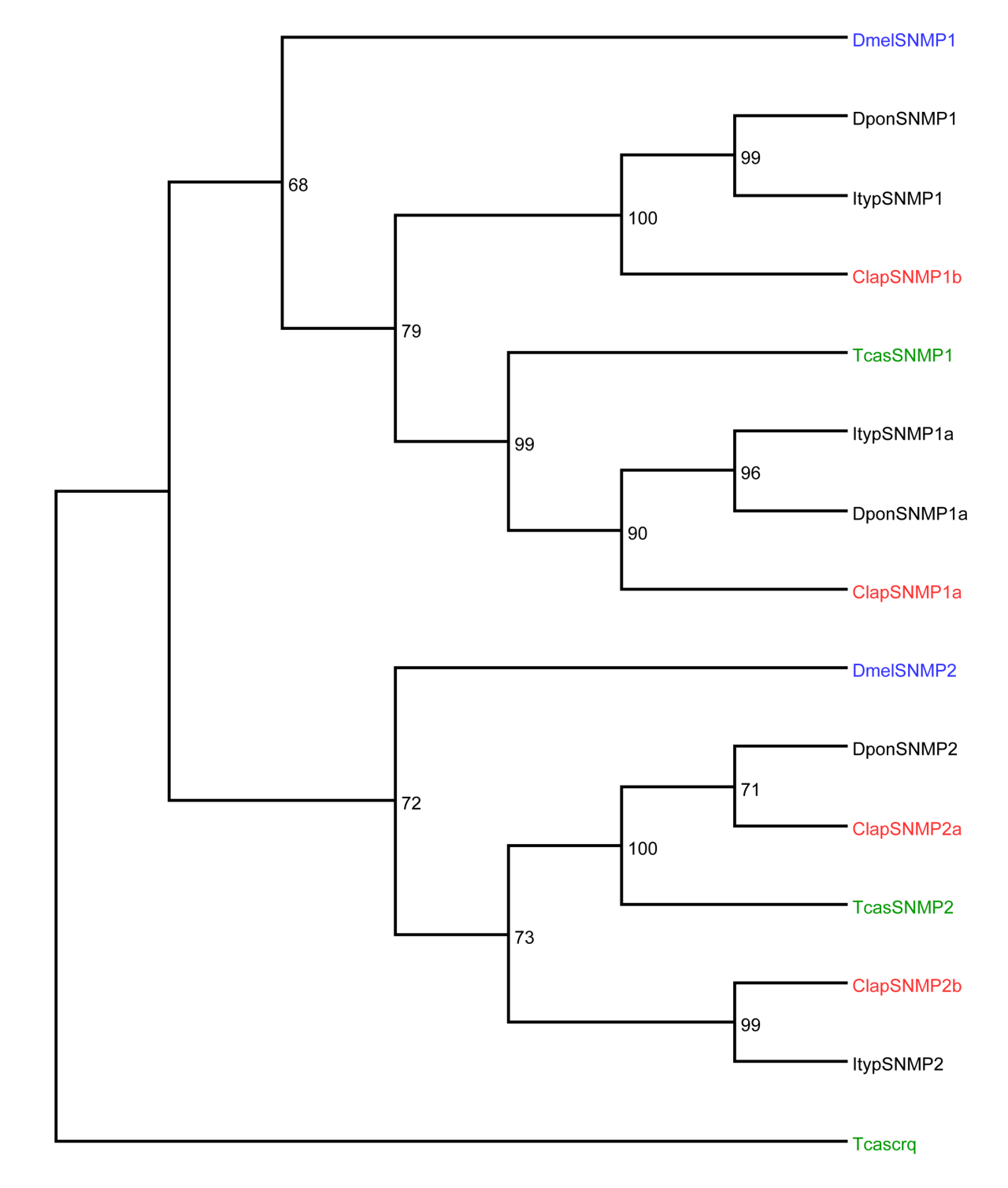


**Figure S3**. **Phylogenetic tree of SNMPs.** Blue: *D. melanogaster* (Dmel); Green: *T.* *castaneum* (Tcas); Black: *D. ponderosae* (Dpon) and *I. typographus* (Ityp); Red: *C. lapponica* (Clap). Two subgroups of SNMPs are identified. Numbers at nodes represent bootstrap values based on 100 replicates, which are shown when the value ≥ 40%.


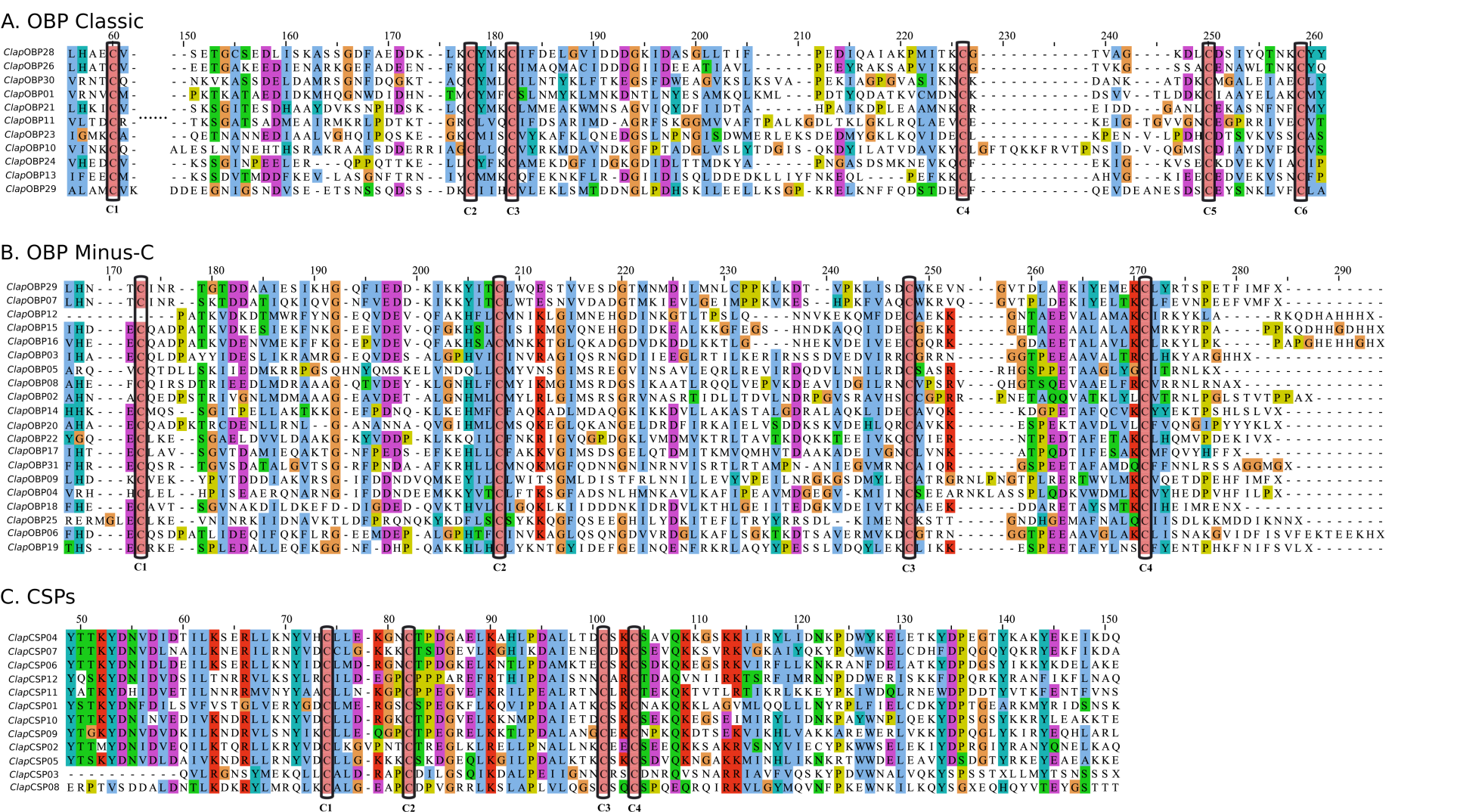


**Figure S4. Multiple protein sequence alignments of classic OBPs, minus-C OBPs and CSPs from *C. lapponica* (Clap) using the program MAFFT.** Conserved cysteines are framed in black.


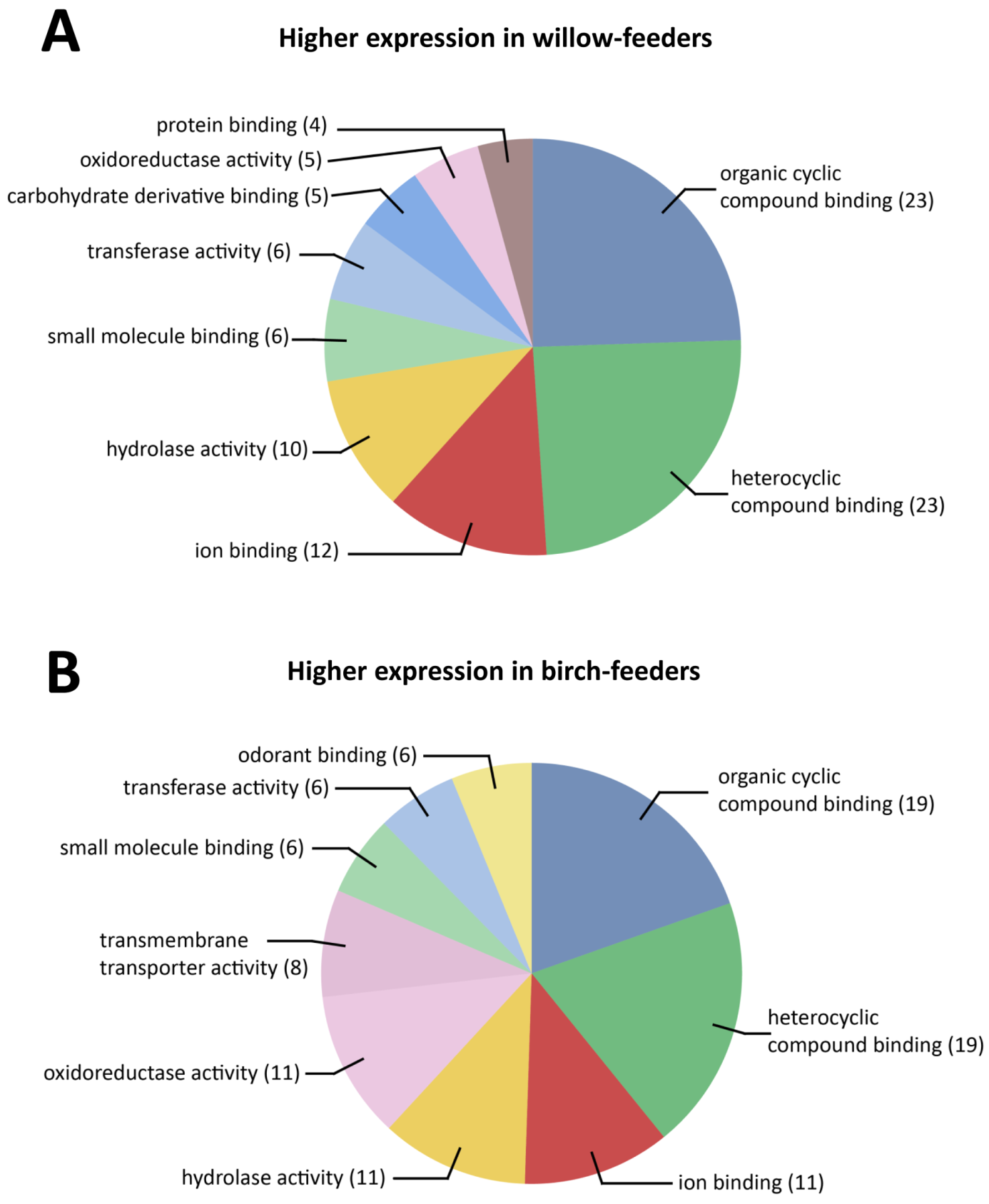


**Figure S5. Gene ontology results in molecular function GO categories, graph level 3.** GO Combined Graphs display annotation results of significant differentially expressed transcripts from **antennae** between *C. lapponica*'s populations.


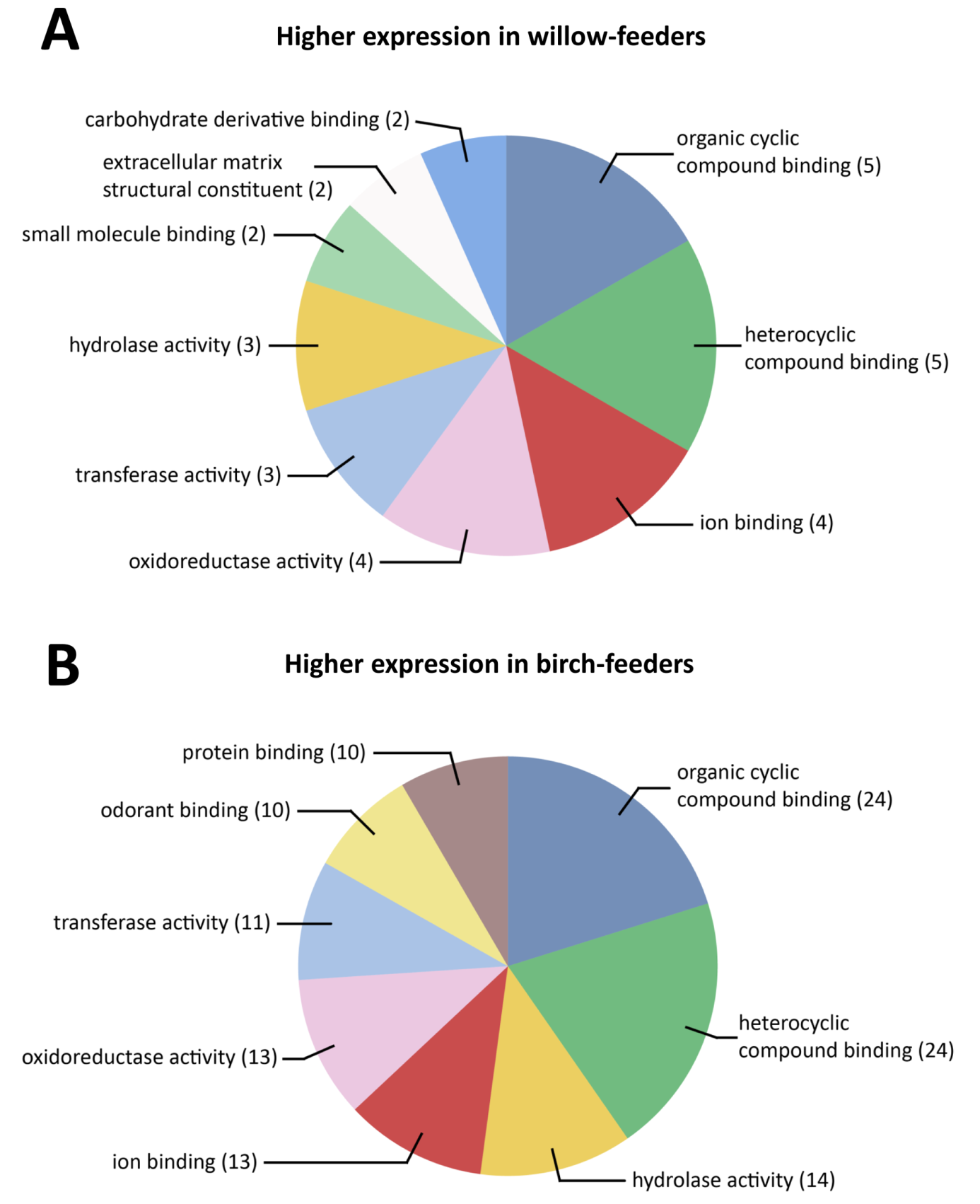


**Figure S6. Gene ontology results in molecular function GO categories, graph level 3.** GO Combined Graphs display annotation results of significant differentially expressed transcripts from **legs** between *C. lapponica*'s populations.


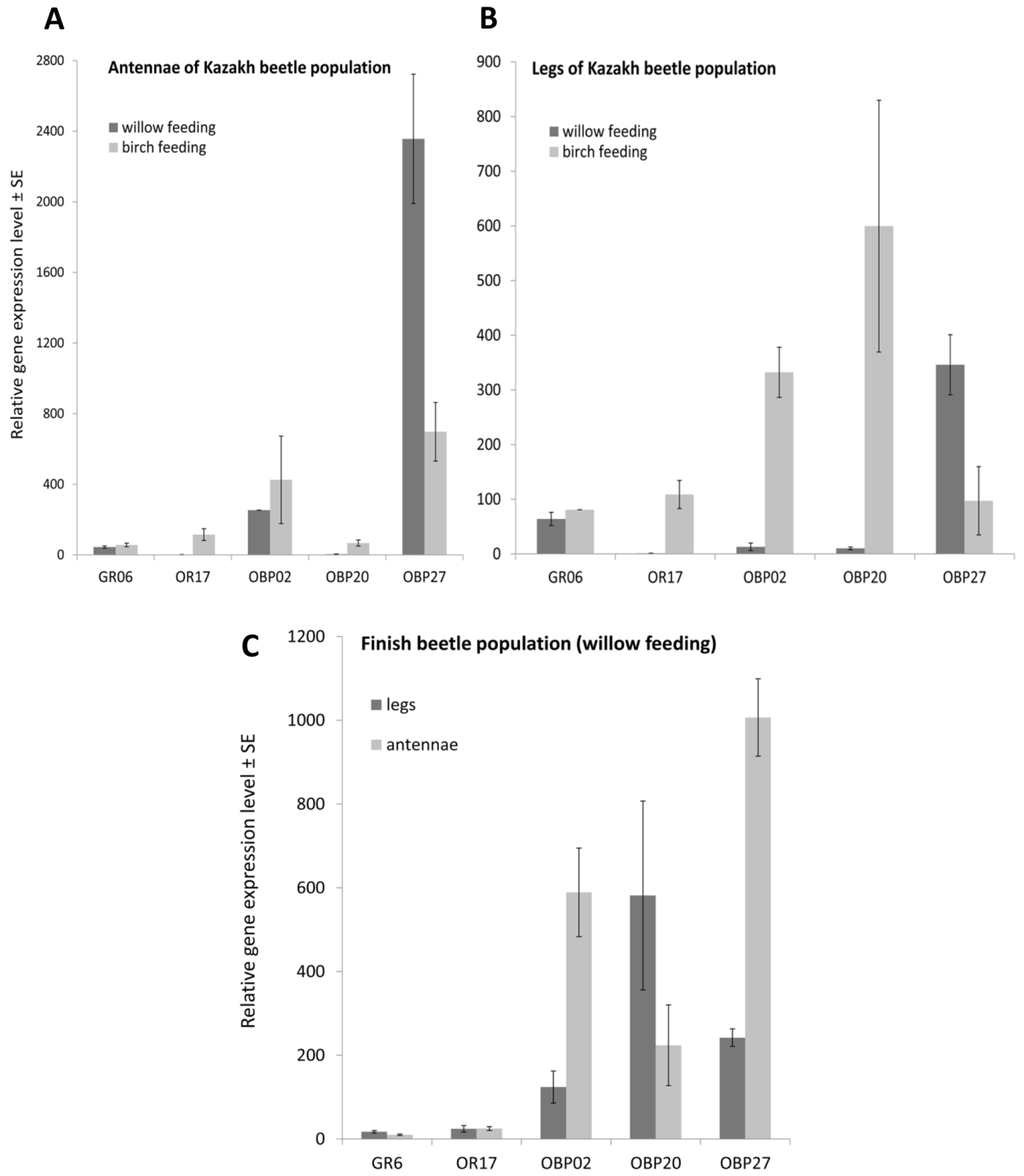


**Figure S7**. **Differential expression of selected chemosensory genes between willow- and birch-feeding *C. lapponica* populations validated by qRT-PCR.** Samples are derived from additional individuals in comparison to expression analysis by RNA-seq data, but show the same overall results of differential expression. Four to seven biological replicates were analysed.


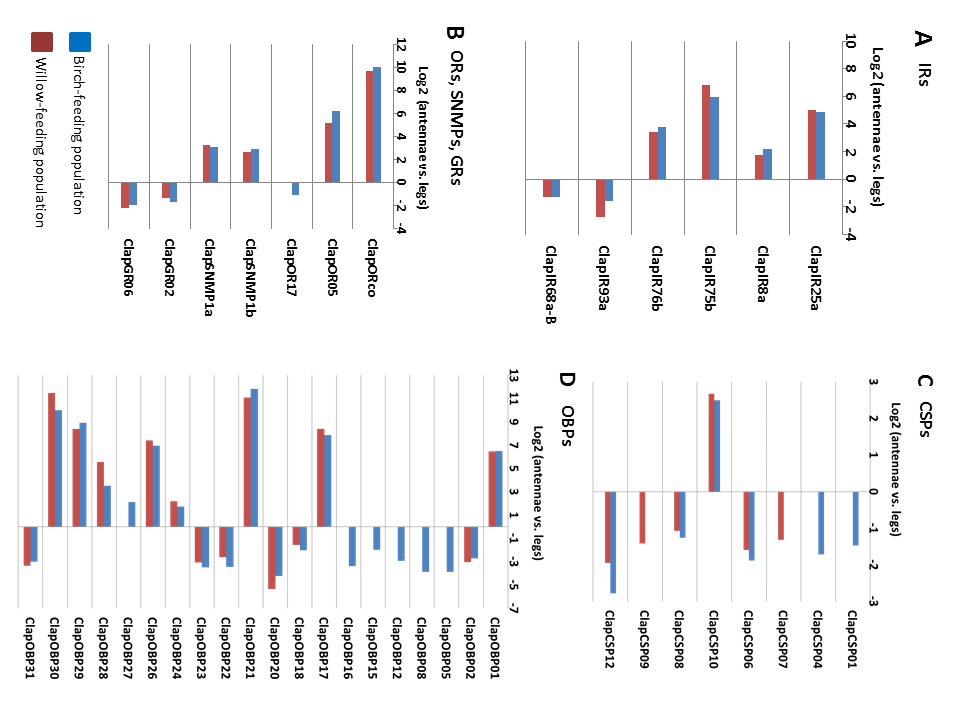


**Figure S8. Significant differential expression of chemosensory genes in antennae compared to legs from birch or willow-feeding populations of C. lapponica.** Significantly different: log2fold≥1, P-value≤0.05 and FDR≤0.05 (antennae positive values, legs, negative values).

**
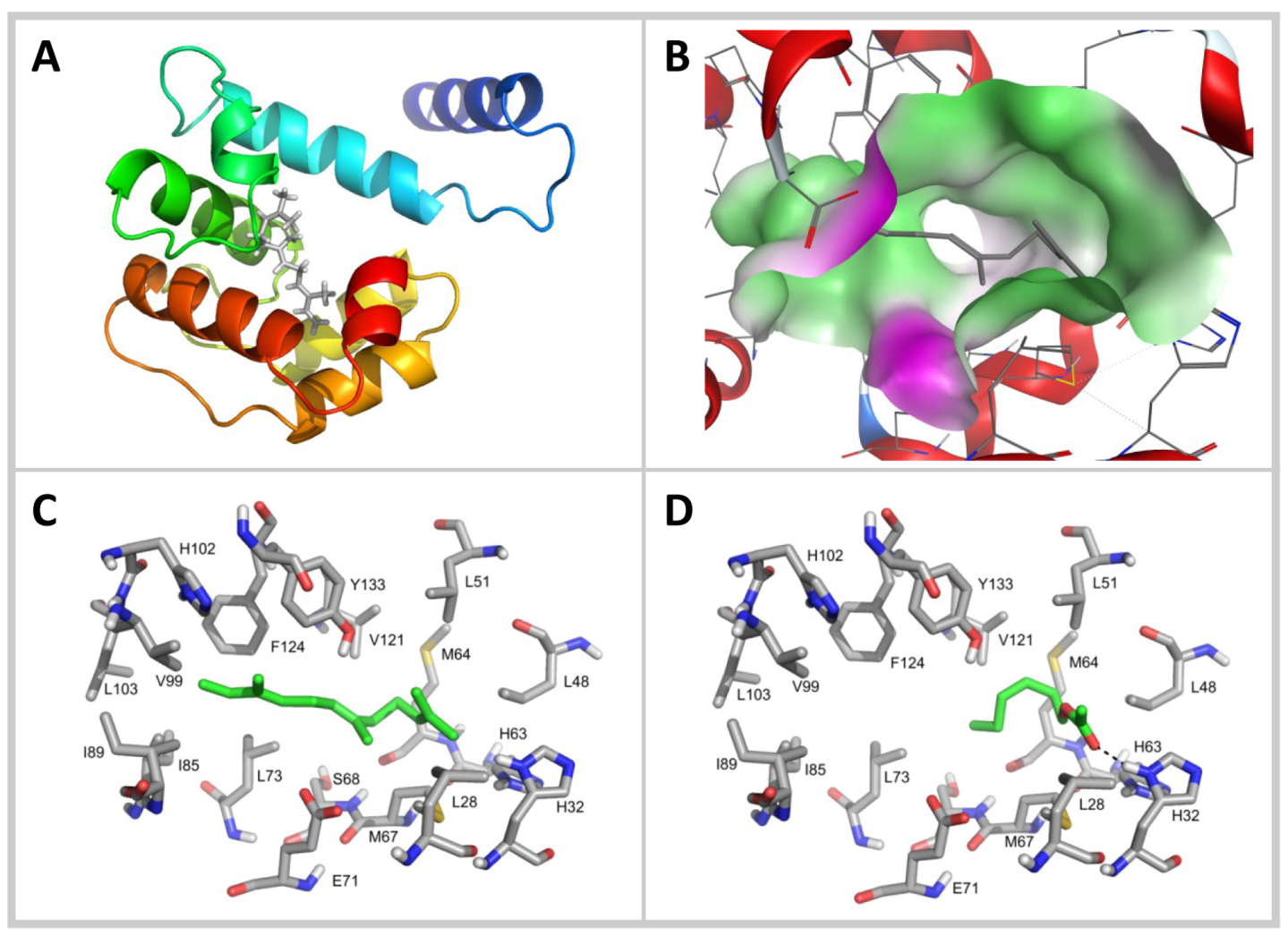
**

**Figure S9. Tertiary structure model and docking studies of the minus-C OBP *Clap*OBP20 (upregulated in birch-feeders).** A, rainbow representation of the 3-D model (N-terminus dark blue, C-terminus red); B, graphical representation of the lipophilic (green) and hydrophilic (red) potential of the binding site of the ligands with docked (*E,E*)-α-farnesene; C, details of the interactions of (*E,E*)-α-farnesene in the binding site; D, details of the interactions of cis-3-hexenylacetat in the binding site. Ligands are highlighted by green carbon atoms.
