# Supplementary material for: A subset of chemosensory genes differs between two populations of a specialized leaf beetle after host plant shift
Source: Ecol Evol. 2018 Jul 20;8(16):8055–75. doi: 10.1002/ece3.4246 (PMC6145003; doi:10.1002/ece3.4246)
Supplement: Supplementary file 12 [file ECE3-8-8055-s012.docx]

**OBPs**

>AglaOBP1

MKTALVFLCVIVGTLAASLSEEEKKLKEIHDKCQADPATYVDHELLHHLAENINNPKVGAHMLCESKAVGLQKPNGELDLNVIKQKISLTVSDKAKVERLVRECAVKKQTPEKTAVNLFMCLDKDGVTYFHEF

>AglaOBP2

MKTALVLLSVIAGTLAASLSEEEKKLQEIHDRCQADPATYVDHELLHHLAENINNPKVGAHMLCESKAVGLQKPNGELDLSVIKQKISLTVSDKAKVERLVKECAVKKETPEKTAVNLFMCLDKDGVTYFHEF

>AglaOBP3

MKTAFIFVCVIAVALSASLSEEEKKLQEIHDKCQADPATYVDHELLHHLADNIDNPKVGSHMLCESKAVGLQKPNGELDLSVIKQKISLTVSDKAKVERLVKECAVKKETPEKTAVNLFMCLDKDGVTYFHEF

>AglaOBP4

MKIALVLSCVVLGALSASLSEEEKKLQEIHDKCQADPATYVDHELLHHLADNIDNPKVGAHMLCESKAVGLQKPNGELDLTVIKQKITLTVDDKDKVERLVRECAVKKETPEKTAVNLFMCLDKDGVTYFHEF

>AglaOBP5

MKPVIVFACVILGAWAANVLVVEEQNLHHLHDACQSNPATYADHELLHHLAENIDNPQVGAHMLCESTKVGLQKENGELDLETIKSKVALSVTDSDKVDRLVQECAIKKKSPEKTAIHLFMCLDKNGVTYFHEF

>AglaOBP6

MKTVFVFLCVIVGILAQDKPKLIAEEQMLEHIHDECQADPKTFADHDLLHNLAANIDNPQVGAHMLCESTKVGLQKENGELDIETIKSKIGLSVDDPNRVEFLVKECAIKKNTPEKTAINLFMCLDKNGVTYFHEF

>AglaOBP7

MKTALVFVCAILGALAKTHLEEEKEKLHKIHEECQADPATYADDKLLENLSQNLDNPQVEAHMLCESTKVGLQCKDGHLNIKTIKEKVALCVKDEKEVDRLVKKCAIQRETPGKTAVYLFMCLDKNGVTYFHEF

>AglaOBP8

MKAFAVILCSVFAVALAQLPDSERIHLKQVHDSCQADPKTYADESRLKQLNKYIDDNVVGTHMLCMSRKAGLQKANGDLDVGVIRQKIDLVTADKSKVDGLVKKCAVVNGNPKRTANLLWLCFVQNNIDYLHRL

>AglaOBP9

MKTLFVFLCVAILGALAHHLPETEMRKLKEVHDSCQANPTTHVDEKLLKKLKENVEDKKVGTHLLCMAVKVGLLTQDGDLMKNVIRDKIVIATHDVSKVDEVLKKCAVKRETPEKTAIQMLVCFIDNGVHYHHNL

>AglaOBP10NTE

AQLPPEERQTLKKVHASCQSDLATRVEKEKLKNMDKYFNDIQVGAHMRCMATKLGFISEQGLMNREVVRSKIALVTPDESKVDVVMEKCAKQQKTPEKTAVLLSLCFYQNGVQYHHGI

>AglaOBP11

MRTCAILVFIATLVVSIHCATDEEREKFKALHNECQADPKSHIDEESLKKYIKGELLDKSVVGSYAFCISKKVGFLNEEGKINKENLKKALGRNISDEAKLNEAVNKCAVEKDDPQDTAIAIGKCFREQAGLAQS

>AglaOBP12

MRTCAVVVFIATLVVSIHCITDEEKERLRTLHNECKSHIDEETARKFYKGELLDKSVIGSYALCISKKSGFLNEDGKLNKDNLKKALGRAISDEAKLNEVINKCAVEKDDPQDTAISLGNCFRREAGAPHH

>AglaOBP13

MKTCIAFICVATLVVSIRGLKASAHLECQADPKTRVNEISIKRFNDGEDIDKSAIGVYALCLSTKLNYINEEGKINIPIVKKFFSYLFLNETVVNQAIERCSNAQKDDPQDTAMALAKCFRESYNDFHKGNNLNH

>AglaOBP14

MRTCAIVVCIVTLVVSIRCTNEGQKKRVEKIHIECQADPKTQVDEELLKRYYKGEQFDKSLLGAHMFCMVTKFGVLDEDGKIIKSTLKTTFSRFISDETKLNEAMEKCAVEKNDHKDTALDLVKCFREESGLSGPKHIHDRF

>AglaOBP15

MRTCAIVVCIATLVVSIHCASEEQHERVKKIHSECQADPKTHADDELLKKYHKGEEVDKSIVGAHMLCMSTKFGVIQEDGKINKSALKTSLSRLISDETKLNEAIEKCAVEKDDPKDTALALGKCFREQGGLRGHEHIHNRL

>AglaOBP16

MRTFVVIISISALVLGRPDENLATINMAHNECQSNPKTYVDEDILDRISGGEKIDNPSVRAHILCVTTKLGVLNEDGEVNRTNLRTVLSRVILNEEKLEENLEKCAVEETDAEEVALVLDKCFWNNLDHDHNSHIHYHHQKT

>AglaOBP17NTE

VTGTAINRDLHKECQADKATHLDENIMKALDEGEITDRAKVGAHLLCISTKAGVINQDGSINKNMVKEKFSRYYSDAAELERITNKCSQQGTTPVETALKLAECINEYSLEQ

>AglaOBP18

MKIFASFLCVFITCALVYGAPSIRDIHGECQSDPATRLDHDEFKAVRTGESFDRTKVGAHMLCMNKKFGTQNADGTVNRNAVKEVLAQDITDETKLEEITNKCVEEGSTPSETALKLSKCVSENTKGGRHGHGHEHHHGHHHEHHHDH

>AglaOBP19

MNTAAILCLCVIFFSAVQGFSKEQREATHNECLAQTGADEETVLKAMDGEFADDPKFKSYLLCFGKKEGFHNDAGELQKDFMRAKLLEMFGDDATVDEMMKCAVEKATPEETAFEGCKCMYAYKNKFVDYF

>AglaOBP20

MNTVVLFLFLCIISFSAVQCSIEKEQEAYHYECVAQTGVDQLIIMKAMGGNFIDDSKFKSYLLCFGKKMGVHNNAGDIQRDVMRTRLMEMVPEEAIVDKMMECAVNIGTPEDTAFEGCKCLYAIKQQLVN

>AglaOBP21

MKTTTVLLCVTFLSIAHTMTNAQKQTMYEECVSETGVNEEIIENAMAGEFADDPKFRLFLSCFAKKRGVLNDAGEIQKHTLRAELSSMISDEALVEDIMARCVVQSGTPEETAFQISKCIHARKQS

>AglaOBP22

MSVKVVFVLTSVLAVVWAQELSTTQIQVIMNHHKECKAETNIDDSLASGVLAGNFSDDPVLKQYLFCMNKRLGILNDSGEIQKDAMKEKLGKLITDEAKIEELTNSCSVEKSSPEDTALELTKCIYQGTHP

>AglaOBP23

MKLVIAAVVLTLFIVVKAELTLEQLKKLKDHRENCIKESGADPEVVAKARTGNIAEDPKLQEHLLCMFKRIGFMSEEGKIQRDVLRRKLVDVIKDEELADKLIGECVVEASTSQLTALESFKCFFTKTGLPIV

>AglaOBP24

MKFAVAIVCLVVVAVARASLTEEQIQKLKGYHKDCAAETGVDTELVTKARKGEFSEDPKFKDHLFCVAKKIGFMTADGEIHRDVLKEKLGSAINDDAAAQKLIDECAVKKDTPQATAFDTIQCYYVKTPTHISIV

>AglaOBP25

MKSLLLLIVLGVLSVQADIQLDEEHVEHVRNHREVCSKKSNVDPMLLEKARKGEFSDNADFRYHVFCVSQEIGYIDDSGKVLRDVLKLKTTEIFNDKADEIIEMCLSKSEDPIQLAIETMKCYYDKKGLVLL

>AglaOBP26

MKVLAALIAVCVVASLVQAELIITEEQKSKLIGHHKVCSEQVSVQDDVIEKLLEGVFSEDQSFKNYLFCLSKRIGFQNEAGEVQKEVIITKLRDSIEDPSKAEEYTEKCLVKEGKPADVVYKVVTCLQESTPNVLFA

>AglaOBP27

MKTSVAIIVTCVIFSTAKATLDITSEQKAKLDEYKDECKKQSGVNVEILKKLPEGEFPEDPKLKEFLFCVSKKTGFQNDAGEIQQAVIIEKLGKALKDPAKAKELTEKCTNQEGSPSEITYKFVICFYNHSSKHVVIV

>AglaOBP28

MKYFVLSVVCLISVVQGGLLTDEQKQKIMQIHAECLKETGVDEAITLQAQKGEYVDDPKLKKQIFCFNKKAGFQNEAGELQIDKIKANLMEMIKDSEKADELIKQCNIKKENGEETAFAVTKCFHNLAPNKDFLA

>AglaOBP29

MWGSVILIICVFLMTTQAAPTISDPYAEDMKNRNQIALECINQVNIDITIVRNAVSNPDKIPTDDKYKKFLACSYKKQKFQSENGEMLYGNINDFLARFYTRDQLKILNQCKSITAQNDAEMAYASLQCILKCLHDIEETER

>AglaOBP30

MKVFATVAVVVVVLVVCCTGQTYTEEERERIRKNREDCITETKVDPNLITRADAGDFVDDDKLKCFTKCFYQKAGFVTEDGELLLDVIKAKIPASVDKERAVKVVENCKQTGKDACETVYLIHKCYFEYTQRNNLNKTPENAETATEAQTEAKTEAQKESTTKSKA

>AglaOBP31NTE

MKVLAYMAEDINMDLKFIKICNKTSPISIHTMNEVLVNKKLMKGESSSFKCFLHCLFAKYGWMDEEGGFLLHDIKDTLEQADVEIASLEYILYKCTALESVDRCERSYMFTECFWEKMSEQQPSEDQLFYSIEDYK

>AglaOBP32

MFPSVVCAVGSYLIFLTIIQAKEISSGRCDIPPSAPKKVEEIINQCQDEIKLAILSEALQAFNVNEHSRSRAKRATFSEDEKRIAGCLLQCVYRKMDAVNEKGFPTVQGLVSLYTEGISQKDYILATVQAVNTCLIKSQKKYLITPQSIDENGKTCDIAYDVFDCVSDEIGKYCGQRP

>AglaOBP33

MNNLLLLIVVASVLGAFRVQATLDQSEFGPNILKLVETLHSVCVLMSGTDESSIAKVIQGEFTDEPKIKAYMKCLFIETGVIDEKGNFNTEVLIELLPPKILDEAVNIFKNCATRTKGIAREEDRIFALLKCCYDQNPDIFIFF

>AglaOBP34

MDSSLFFILVCSLLAVSEVQATVAIADLGPKIKQLVETLHSVCIPRSGTSEEAIQKVINGEFTDEPKIKAYMRCLLEESGVVDENLAFNNDVAAALIPPKLLNDVLENIKICEPKKAGFQSFDDKAFVFVKCAYEQNPDIFIFF

>AglaOBP35

MINFKFLLAVSSVLAVSTVEARLRASEFGPRIQSIAPRLHSICKFRTGVLDYSINSVINGVFIDDPLIKSYAACLFRESNLMSARGEFNYELGAYLLPADIKDEAISNAKYCDTEIRGSAKVEDRLYELLKCFYSLDPDIFIFF

>AglaOBP36

MVSLNFFVTVSSLFAASVVQAALPQSEYGPQLEALQKNVRAACISTSGVDETAISNVGNGVFTDEPKIKHYLTCVLKEGKLVNEKGVFSEKNIAQLFPDKYKEESLTNIKSCIVKVNDITNLEDKIFALFKCYYHQNPDLFVFF

>AglaOBP37

MGTAIFSVVVTAILAISSVQAILKQSDFTPELKKLAANLHVTCVSKTGIDEALIDKVLNGEFVEEPKMKAYMTCLLLEGTLIDEKGTPNLEFGATLIPENIREESVKNIKHCYAVNNDVTDLEEKIFRVFKCYYYINSDIFIFF

>AglaOBP38

MDNLLFVVVMFTLLTLSMVQAVLDESEFTPKLLEQVKALHDTCASQSGADDGLIGKIKKGDFVEDPKIKSYMKCGLTELGVMDDKGEISVDMVPELVPAKYVSESIANTKTCTGKTKDIANLEDRVFAFFKCYHDLNPEIFIFF

>AglaOBP39

MRGSTLFLVVFCSVGYSKALECGIGKVNSEDVKKTLSTCIKNNATLEKIWEMASSSATPASDSSESSSMEDDQPQTQNTCIVQCVFKQLGMTDDNGLPDHSKIVEGLLKNVTERELSIFLQEAAGDCFQQMDQDKNMDSCAYSTQLVSCLANKGRMNCEDWPAGNLPF

>AglaOBP40

MIVPTIVILVAFILVMDVDADLTDKLRQKLDLPEVQKCITSTGYIPKGPPEGPSQEFTPEQLCFFKCIMEEKGLLDSTGNIIQDELNNVPLPIPDDKKNEIKKCAAGAGKIESCEDIQKLLSCLPM

>AglaOBP41

MNIVKMKAAREIFFIVGVCVALISGMSDEMKDLLDSLHAQCLSDSGVNEELISKAQKGEFEEDEKLKCYMRCIFDETGLFGDDGKIDVEGMVSMLPDEIKEPFAPTVRKCGGLVSGSPCEQSFKMYKCCFDEAPQYYFLP

>AglaOBP42PSE

CASVDMRLVALRKTCVAETGIDEAFIEKVDAEKVLVDDKTLKCYIMCKMKHCGAEMKETAEPIVRACGVTVGVDDCQTDWLTEKCFLENGPD

>AglaOBP43

MKVCALFVLCMGLSAVMCASMDKRVAELHRTCVAESGVDEALIEKANAEKVLEDDQKLKCYIMCIMKHSGSMSDDGTVDVEAIVANLPDEIKNNGASVVRACGTKVGANPCENAWLTHKCYLQTGPNEYILM

>AglaOBP44

MKICVLFVLCVGFPASMCSLEEWRAMGAPIHKTCVAESGVDEVLIEKANSEKVLEDDEKLKCYILCLMKQSGWMVDDKTIDVETVVMVLPDEMKEKATPVVRACGTKVGANPCETAWLTHKCYAENGPNAYVLI

>AglaOBP45

MNLSVTLLYVLFCLTSIKGLSESKFIAAKAEARAACLASTGVSEDLVMDINRDGKFADDENLKCYVKCVHEYLGLMAEDGTMDYEKLIENIPEEFRIKYASRIRACGTIYGSDVCDTAWLTIKCYGENIPKLPHP

>AglaOBP46

MLIRLGAACGCVLLVLPLVLSISEELQELVDMLHNTCVGETGTSEEAIENAKKGDFADDEKFKCYLMCIMVQMACIDEDGIVDVEATIAVIPEEFQDLAAPIIRKCDTQKGSTPCESAWLTHKCYYNENPDAYFLV

>AglaOBP47

MKFCFFLLCSCLAAPYVYSAMTEKQLNAAKKLMRNTCQNKAKPTSEQIDAMQKGDFNGDRNAQCYLLCILSTYKLLTKENTFDWENGIKALAANAPASVAGPGSATLKNCKDAVKTPSDRCVASMEIAKCIYDDNPSNYFLP

>AglaOBP48

MWRKYFLASILTCLLSVYQVGSLTEKQVQATVKMVRNTCQSKTKASTDDIEKMHLGDWNIDREAMCYMQCALNMHKLLNKENKFDYESGMNQLKILPESFRGHTLECMNQCKDAVVTPNDKCIAAYEFAKCFYFCNPEKYFLP

>AglaOBP49

MKILVVLACFIVLSSALDQDFKEKLMQQMEEYGTKCIDEVHATDADIAELVAHKVPPTSHEAKCLIFCIHKSFKVMTEDGNPNTEGVLQLMAPLKESDSDIYEKFLKITEKCSDSLEKDDDHCVTSSNWAACGINEAKAMGMPDDLFQM

>AglaOBP50

MKTVFVISMVVIMAISHAEMDKEHQECLDETELTEAEVSEFLLGDDAENDAKATKFLMCIFKKNEAVNDEGHFDVTKAQEVVKHYMTEVVGSEDQQALDCVQEKDTTEQTVLALGKCVEKRKVELSSSK*

>AglaOBP51

MNNLVAFCALLVAAVSAYNFEDPDFNILLSDDLEELSSGVASFSHPRSRRDDEAVNDKDKCHHRKRWGELCCAEDVMAKMRDVEKDLKRECFKEVVGKDKHEKFDPFNCETMDQRKKQIVCVIQCVGQKKDLLDTEGNPKEEEFRSFLKESFSSESWLAALQDKVISTCLDEGKNATANRDASDSTSCNPAGIKIAHCLHREIQLNCPADQIKDEKSCARLQERLKRRDFFHPPPPPGAFDEPDN

>AglaOBP52

MIAYALFFFVFASAVLNVHCDILEKNITLAAQCMEETKVTREVVDSYIEDPEKEPTEDFYCYLQCVYTGVGLIDENGDLDIDLCKSMFVVDVDCLKDMPKIVKCTDMEALSECVKD

>TcOBP0A

MNCFVIFALSLSATVFGQSLSEDEMRENARKLMTSCKDKVGASDADVEALKMHQMPESRE

GFCMLECVFDSAKIMQDGKFSKSGMIEGFKPLIGDDKAKLESLEKLSATCESELGDGEDK

CETAKRLVECVIKNGKTHGFEVPPPRE

>TcOBP4A

MKLFILAGILFTGVCAVDQEFVEKFLQKMEKIGEECAEETHATSDDIADLIEQRDPKTHE

GKCLIFCYHKKFNTMKEDGSLDKVGSVLALEEVRDADFELYKNILTIFVTCGDKAKIYDD

PCETATALTMCGRDEAKALGLQDAIFG

>TcOBP4B

MKSTWFFLLLACSLTCALDQEFVDEFLEKMQEFGAQCAEETDATSDDIAELIARKLPPST

HEGKCMIFCMQKKFNMMKENGGIDRAGAIAALKPLQKADPELHQKVLKIFVTCGMRVKPS

PDPCDTATELALCGKKEAEAIGLEDALLT

>TcOBP4C

MKYFVVFASLFLATNALSQDFIDKFVAKVKSIGETCVPETNASKDDISSLLAHKMPDSHE

GKCLIFCFHKQFQIQNDDGSINREGAIKALEPLKADDAELYEKVISIFKKCESTPVDGDS

CLYAASLAECAVKEGRAMGLDNLIVLEIE

>TcOBP4D

MKFLLVFLSVAILCTFAMDESFLQQTRDRVKAIVKECVTEEKATDSDFDDIMALKIPTSH

EGKCVFFCSHKKFNMQHPDGSINKEGALDTFEVVKDVDAEFHDKVITVYNHCLSTPVDPD

PCVYSVNLFQCFMKEAKAAGIHELIIK

>TcOBP4E

MKLLITLATLVVATYAIDKEFVQELRQKLRSHVEACAKEVNAGPDDVSAIFAHKLPATHE

GKCIFFCMHKLYNAQNEDGSLNMAGALANLELIKDMDPDVYTKVSTSFKNCESAPFDSDP

CLYAANLVTCIVKEGRAVGLDEVLVE

>TcOBP4F

MQLLVVVLAVCVLGANAGLDPKFLEKLTQEVQAVGTSCGEKEHATADDMIEIMEEKFPPT

SHEAKCVVACFYKHYKMMKEDGTFDKDAAVKAFDEIKAQDAEIHAKILKVIDACDAKKQM

SDDHCVSAASMAGCVKTEAIANGLTKEAFMAS

>TcOBP4J

MNSVLFLLVCALVACSGELDKEFLMQFLQKIKKVSEDCIAETQATKNDIKTLLEHKIPDS

HEGKCMIFCFHKHFQIQNEDGSLNKVAAISLLEPIKDHSQDIYDKVVKIFNTCFDSAERD

DDSCIYASNLAECAIRESKSLGLDDLLVIE

>TcOBP5A

MCPKFCILVLSIIVIVQAEDAKEKKCDIPPTAPKKIEDVINQCQDEIKLAILTEALEALN

INEHTKSRAKRDTFSDDEKRIAGCLLQCVYRKMKAVNEKGFPTVEGLVALYSEGVTQKEY

IIATLQAVNVCLNKAQKKHLTKPQSLEAEHGKTCDIAYDVFDCVSERIGEYCGQTP

>TcOBP5B

MIRYYIVLLLYFFAPPVLGISEEMQELVNQLHSTCVAETGVSEDLINKVNSDKVMIDDEK

LKCYIKCLLTETGCISDDGVVDVEATIALLPEDMKAKTTPVIRSCGAKMGANPCESAWLT

HKCYLETSPADYVLI

>TcOBP5C

MSPLLLIFISCLFPRVFGISEEMQELANTLHATCVDETGVSEDAIESARKGNFAPDDKLK

CYMKCIMEQMACIDDEGIIDVEATIAVLPEEYQAKAEPIVRKCGTKIGANACDNAFLTNK

CWYEEDPEDYFLV

>TcOBP5D

MYKTRVIYVLFALCLVEIFAIEMDDDMKELINNLHNTCTGETGATDDQIENARKGNFAED

DSFKCYFKCVFDQMGCMTDDGKVDSEAVIAVMPPELADKIASTVRGCTEVGANPCETAWL

ANKCYQKSNPDMYFVP

>TcOBP5E

MNKQIAVVTFLIAVAALTAAYEFNDPLFNQILANELVELESSAYPHHRSRRDEDAVTEKC

RPFRKKKLCCAEETFDELHDKDRDFKRECFKQVVGSKDGPREFDPFRCDKVDKHRRDMTC

VSQCVGQKKDVLDKDGNVKEAEFGEFVKETMAKESWFVSIQDKVVSTCLAEARNATANRD

TSDTESCNPAGVKLMHCMFREIQLGCPTEQIKDQKACARARDKIKRHNEFLPPPPQFLND

E

>TcOBP5F

MLPAALFVVLATLTFATAEIVVPDDLKDYINELHDHCLKEMGLTEGDHKNYNIHVKDPKM

MCYMKCLMTTSKWMNMDESIQYDFILSSVHPAVKNILLPALDKCRDIPKGTMECEKAYNF

NMCLFNADPENWFFI

>TcOBP5G

MMPLKNLIILIVCPLFVFAKVDIPPDLQAEIDGYYDICYKQIGLTKDDLKAYKIGDRDPK

IMCFMKCVFVEAKWMDENENLQYDYIKNTIHHSIRHITLPELENCGKKAEGDKCEKSFSF

FNCMNKAEPEDWVLIQ

>TcOBP5H

MMHLKNFVVLVVCPLFVFAKVEIPPDLEAEIDEYFEQCFEPNGVTMDDIKAYKMGDKDPK

IMCFMRCLFVSGKWMDENENMQYDYIKETIHHAIRHITIPELENCGKEAQTGDKCEKSFN

FFMCMNRAEPEDWILDYKS

>TcOBP6A

MSFLILLICVIPAIFCRSFSHDELDTDLSFIKTCNRTSPISMRTMNEVLINKKLGHGESS

AFKCFLHCLFMKYGWMDSDGGFLLHDIKQTLEESDVEIASLEFILYKCTATESNNRCERA

FVFTQCFWDKMAEQQPSEDQFFYNIEDKK

>TcOBP6B

MILKASIFLILAVATFGAILEDSELMKVVENCVKKTNANESEFSSPNFLETTPSQPALCT

AKCLLESLEIVNSEGNINMETLKEYAQPFESPAREAVATCGEEIKSVTTCDDMEKYRKCV

EPLIKNS

>TcOBP6C

MAKKQLVLFFLAFIFLQSSWAYFFMSQKFAEVREECLSENSMTMDELHEGWKMENLPESH

LCFLKCLLEKREVIDENGVPQKEKIDEILTVKQLSDEKREEISTCITNVEKIENCETMSE

IMRCFPKKRRD

>TcOBP6D

MCRLFVVLSLFVASQALDVEKIRNELMADKNFVELRNKCLDKLGLKEEDLRDLKFDGDVS

EDLMCFGKCIQEEDGLLDSEGNLNEEKLEKKIETMPFLSRVSDDTKNNIMECLKEIGKIE

TCQDFGKQRDCIHKYV

>TcOBP6E

MNPITSVILTFLFVFSFGEKESEEALQQIFTELDGPAAELRDQCLEKNSMKVTDLKTYNT

SNDIPEKELCFYKCFYEGVEFIDANGNLNVNNMKEIPAISELGDEVLNEITACVEKIGKI

RCCGDLRKIEQCYQNITM

>TcOBP6F

MWSFVTLLFSFLVLASAQKKGKYWTTISECLTEHSMGVEDMKKFDLPAEKMSEEMLCFNK

CFYDKLLITDENGEINTDNLMSIPLVNAIDASKHDDLVTCLKKVGKIEECDGVKKIEQCF

VEFI

>TcOBP6G

MATRFCFGLLILFVGTVLVFAENEHEILEVRALCMNETGVSEETARNYKPAEDPASEEIL

CMVKCIFEKIGCLKDDGSFCVDTMKKKNYIMDVINEENEEKIYECLRGVGKITNCRDMAA

VEECFVKNDSK

>TcOBP7C

MNRYVLCVILVLQIYPKLSQTLENNNGNKLGDVLKNHTKSIYNSTKNQNEDENIMKQETH

KLLSECGVESDNSYDDDDSYGTRRGNKRRGNNYDNFGNRKRPNRRYSGNESNNYNRYGNR

NRGNRYSGNDDENNSNESTNRGNRRNNGNRNRYNNENYSDEEDSNESNQSNNQRRYNRTM

SNTGYYYGDGGYHNECNDNHGYRNQWRPFGGNVGYDYGNMRRGYGRNLDMNLRAKRSNDN

DDSQCVSQCVFGYLEVLDDNRVPSETLVIKWLQDHLSNDMKRIRALREARRCFARLSTSD

TEDGCEFSQSLSKCLNLELE

>TcOBP7D

MKTVAVLLFLALAACTKQEDDDRQETIRQYRDDCIAETKVDPALIDRADNGDFTDDAKLQ

CFSKCFYQKAGFVSETGDLLFDVIKDKIPKEANREKALAIIDKCKELKGADSCETVYLVH

KCYFLHSYGTDKKTE

>TcOBP8A

MLRLVSLLCLFLLVQGENLDMFDPAGLQACMKKLSVGETELAKALEDKSKDPPEKIMCLF

KCALEDSGFLQDGVVDKSKWPMPECVQDVVKITNCNDMVALKHCFD

>TcOBP8B

MKPIFAIITLTLCTTVHALDCGIHINKNDALKATINKCLISNKTLEDLWDMAPMSSESDS

SSEEVPPVDGKMLQNFRIKRASVRLTNTETNETTPEPKAVSSEAQATENCIIQCIFDNLQ

MTDSTGYPVHTKILDGLLKNTTNRELRDFLQDTTDECFQVMDKEDTMDPCSYSNKLVTCL

AEKGRSNCADWPVGELPFKP

>TcOBP9A

MKYFPHLCLCLIFFELSEAAMSEAQLKAAVKLVRNMCQPKSKATNEDIEKMHHGDWNIDR

TAMCYMHCALNSNKLITKENVFNRDYAITLAEKNLPTALKTASIEAANLCKDSAKTLDDK

CVAAYEISKCLYESNPEKYFLP

>TcOBP9B

MKAILLLLVATLSFYHVYCAMSEAQMKAALKLVRNVCQPKTKATNEQIEAMHTGNWDLDK

NGKCYMWCILNMYKLIGKDNSFDWEAGIATLKAQAPESVRDPAIASVNNCKDAVKTTSDK

CEAAYEIAHCMYLDNPEKYFLP

>TcOBP2A

MNFFAICLCFVASTVGVSSEENDINEIRSVEENCQKQTGVSVEKVNNFELVDDPLVKENA

LCILKAYGIMDEDGNIYEDKLKEQITSELGEKNAEQVAKKCTIKKESPQETAHESLWCVG

EQKPIPGASPDEKN

>TcOBP3A

MKKTILLCLLSQLLLLKAAELQPEDRHQIALQCIDIVGIDQKVVEDAINIEIPKNNPKYK

EFLACSYKKQGYQNENGEILMENIKKFLQKFYHPSDLQELNSCSGHNGTNHAENAYQALQ

CIYNRLSNMTVVGN

>TcOBP4G

MKMCVIFTLLLLVVLASAEEDNVGKIESVEKKCQEKTGVSEESLQKIMRLEEVDDPLVKE

NALCTLKAYGVMDDDGNIFPDKFEEKLKPEIGADEAKRVAEKCAVKKDSPEETAHQTLWC

ATEENALTDTSQEQ

>TcOBP4H

MKISTLVAILVLAGSAVCADEDNLNTENVQSIEEDCQKETGVSDESLQELSETGDSDDPL

VKKNALCILKAYGVIDDQGEISEDKLEEKLEPDRGKEEAEKVAKSCAVKKDSPEETAHEA

LLCMQQKSQK

>TcOBP4I

MKSFVIFVLIIVITGQINATPSLDDFKKVQKDCQKKTGVSDESINKVNNLEPVCDDLLLQ

ENALCILKTYEVMDEEGKICPDKLMEVLEPKFGKEKAEKLIEKCTLEKDTPQLLAHATLF

CLSVQKYVV

>TcOBP7A

MKQIYCLITVVVLIPTLTALENEGQNPDTANCVALGGQRIKDSEIAKMAHCILTKTNLMT

DKGTFNSNLLKERLRQSVHSDELVDKVVMMCTVEKETPLKSAFSGYKCLRYLVPWFPLD

>TcOBP7B

MNIYTCLVLVVIAASAQHLTEEQKNNWRKWSNECKVLIGVSQEAINKIRNNEFDSVDDKI

KKHGLCFAKKASLADSSGNIIINQIKIKLKRVIEDDEEVDRIVTKCTIRKNTPEETTFET

FRCLRENSSKFVPV

>TcOBP7E

MKAFIVLVAVAVCAQALTDEQKEKIKNYHKECSAVSGVSQDVITKARKGEFIEDPKFKEH

LFCFSKKAGFQNEAGDFQEEVIRKKLNAELNDLDATNKLIAKCAVKKDSPQQTAFETIKC

YYENTPTHVSLA

>TcOBP7F

MKFLVVISTVLMANIVQGLTDEQKSKLEEYSKECLKESKVDESVLKEAEKGVYLDDPKLM

NHVYCLVKKINSQKDKGELEVTQIKEKLMMQINDEKEVDKLIQLCLVQEKSARYSLGKCE

VSS

>TcOBP7G

MRASAVFLSSFIISIQAAAFNNPEDELRRSAACLEQSKVSSESIKNLQIGNFDDDERLKE

YLFCVSKNAGYQDPAGHLQHEMIRLRFKGGRYSDDTINEVLQQCGHQKDTPQETAFQFMK

CAYQNAFPRNYK

>TcOBP7H

MKTIICFVFVLAGAWALTKEQIDKLEPISKECRELNGISEDTILKVRRGEAVNEPKLKNH

VLCVSKKTGLASETGETNVEVLRTKLRKVSENDDEVNSIIQKCVVKKSTPEETAFEIFVC

LRKVKPNFSPAN

>TcOBP7I

MNFVCVIFILVAIIGAHGLSEQQTEKLNQLSKECRALTGVSQETITNARNGNFEEDPKLK

LQVLCIGKKVGIMNESSQIDENVLKAKLRKVSDNDEEVNKIYNKCAVKKPAPEETAFETI

KCVMKNKPKFSPVE

>TcOBP7J

MKFLVCLLFVIVAANALTKEQKEKLDKISKECKNQSGVSQELIDKARTGELINDPKLKAQ

IYCVSKKAGLATEAGEINMDNLKTKLKKVAANDDEVNKIIQKCVVKKPTPEETAFEVYKC

LHANKPNFSVVD

>TcOBP7K

MQKILFLGLFVSSSLSTRFFNHDEIQKLECFPDIGGGLKAELWPNVVSCVFHRKGFTDDK

GEFKIDVLKQKLSKFQDDKYLVNEIAELCVDKHDFTMTAAMKSALCLNKHAPWFSPYQD

>TcOBP7L

MENRLVLLIVINTLLLAQAAAKQDFHKKCLASSGANADTIAKVRNGKFSNDPQTQKYFGC

MLRSVGVVNQAGQLQVAALRKQVPKDMKRDEAMKIYMSCKDKKGANNDETAYLLYKCFWE

ASPRHVKIDGQ

>TcOBP7M

MKYLLFLTVITLTCGIFAFSLSNREQAIFLSTYSTCLETSKVDSERALRTASGIIDDEPK

LKEFLFCINKQNGVQDDAGNFVKDAVRKRIEHPLLTDKTMEIIVNKCTRKRETGEETAYQ

FLKCSYFTIMNEKHQ

>TcOBP9C

MSKTVFIFVIFFYLDFYATGDESVYLSNHEACVKLSGVDETLLETIYEGDVFEDMKFKTY

IHCFFKKSGFQDENGVMHFDAIKSSFHKDFSQTENIDKTITECEEKKLNGESALETAFLH

FKCFMGEL

>TcOBP10A

MKIVLCLLALATVALAKKCFLAEDTDKLEVMINECKTKTGVPDDILQKARNGEKIDDPKL

REHALCMMKKSEMMNDAGEMQMDKIRARIKHAVSNEAEGTRIMNECAVKKDTPLATAYEM

ICCLIRNKNSVDE

>TcOBP10B

MKVFVCLAVFALVAAAQAETAKEKLRKYSDECKSVSGVSEELLNKVRNHEDVHDPKLDEH

GFCILKKAGFMNEAGDILADTIKTKLKENSEHPDTVDALVEKCNEKKDTPQHTASHLFTC

LVDKKVHSH

>TcOBP10C

MKLFILLSLLSVCYARKEWFDKDPQDVAKWQKECFEASGVSMESMNKLPNITLSEDPKLG

ENAFCLLKKLGFISEDGTLLIEKLRTSLKNQWGDEIANKLVNECARQKSTPQETAHEMFL

CIPAKLK

>TcOBP10D

MKIVLICVLIGLVVAKQQKQDTLDEEKEKMKKWTQECIQESGVTSEILQQLRNQKRVEDP

KLKEYTFCTFKKNGFMNEDGKLQYDVIKSTLMKVSGSEEEANKVVKDCVVEKSTPQDTAF

ETVDCWYRYKKN

>Ityp10

MNAFICMFLVFGVVKAYDFSDSIFNDHLNQIYYTLDNWQHERIRRNAEDVELKCRKPPPP

MPKPCCAQDSFRDLMDKEREVLRDCFKEVVGEEHHPGRSNHPNKFDMFSCEAVEKRKNDI

ICIKQCLGSKLGLVNKDGKLDQAQIGNYVKSTFKNEAWLSPLADQIIGKCLVEAESVAPP

KFHIEKLKPCKPSVITFKHCLDREIQLNCPADQIHNQESCERFRNHLNHKNDFDEDQPMM

GPPDDD

>Ityp11

STGDSTMKFILLVIVVGQMGCVFGAMTESQMKAAFKLIRNVCQPKNKATDAQIEAMHKGD

WNQNKNGMCYMNCVLNYYKLQLPDNSFDW

>Ityp12

MLTVGKLVLVLVLVLIETSALQKTNNKCEIPTAAPKKIEDVINTCQDEIKIAILSEALEA

LNINEHKVSRKRRSTFNDDEKKIAGCLLQCVYRKMNAVNQYGFPTVDGLVSLYTEGITQK

EYVLATLQSVTKCLGKAQKTYDIPAQNGTASTACDVAYGVFDCVSEEVAKYCGQTP

>Ityp13

KVEMLTDKNEIIKCAMECMQAEIDRLTEERNEHLKKLFESHNAQISETKKKQWCYNCEQD

AIYHCCWNTAYCSQTCQQQHWQAEHKKVCRRKRQT

>Ityp14

MYGSVLKVSLVFAVISVISCQDFTEEQRKKIIQNRQDCIQETKVNPELIEKADQGEFIDD

QALKCFTKCFYLKAGFVNDEGEVQKDVVEAKLPPQADKKKALEIVDKCAVKGKDACETVY

LIHKCYFEHTHPDLPAKAEEEKKA

>Ityp15

MIQHHRTNSDLTAVKMKILAVLFVICVLFQFTIARNGGNLHYSKISMKKVQKRCQKNEES

RIDPDVLKKLRKGEEVVQLPDNFPDHVTCLMKGMEYLNDDNTVNEEKVRNMVQRRVTDDQ

DVDAIVGECKAVKTALKETALNLINCLRKHELLWNHNFHD

>Ityp01

HGPPVELLSCVAKELSLIDADGNVSEAGIKQHVEHVESDAGKAEEIVKACAVNQASADET

VRQLWKCLHDNNIVQTPKHNHGSNESSSSESEEHSHRHRH

>Ityp02

MNSVAVFAVLALGAVCVIDAYNFQDEDFXSAVVVRDGRIVDSIDSGPVHPRVRRDQEAAT

VAEEKCPKRHRRPKLCCAEETLDALHAKKKEITKACFKEVTGLEKQDRHDHGPHFKRFDL

FNCKEVEKRKSDMICIDQCVGQKKGLLDDSGAPIRDQLIQHLKQHFSNESWFDQTVVEKI

TSNCLAAAKNATETPIKFSTEGLKACNPSGITLKHCLFREIQLSCPADQIKDKTACDRFQ

DRIQKEIEIDDLRLAPDDQQ

>Ityp03

MATGKVFVYFFVVLFLSEQSVSRMTEKQLAAAVKLVRNMCLSKEKAKLEEVDKMHEGNWD

IDHKTQCYMWCVLSQYKLIGKPNHFDRESANIQVDTLLPESMHDYVVGCLDKCENAATNF

DDKCVAAYEYAKCLYFCNPKEYFLP

>Ityp04

MISAVIFFALVGTIFCADADLTQQQKDKLLADGKACVAETGVSTDLIQAARQGKFTEDDK

LKAFSFCMSKRLGFQNDAGDIQTEVVKQKLGGALGDLGVAAQLVTKCLVPKATPQETAFE

SFRCYYQNTPTHLTVF

>Ityp05

MRQPGGNNKNTQQDYEMWTPSTGYQPSGSNNDFNVRTRYDGNTRFNRPSSEECRDQGNGN

IPRSPFGSSNLPRRQRSSYFNREDDDNDNDCISQCVLGYMQLLDTDRSPSETLIIKWLQE

HVTRNEMDRIKALRDTRKCFGKLVTTDIEDGCEYAKELSKCLELDLE

>Ityp06

MVLYLCDLVQLAPTNQTEEVTTSTKRQLTREKKKKIGKTCMLETGVRIETILRAIKEDIP

KNDEKYKSYLVCSYKKQGYLSEDGGTMLYDNLYSFLQESAGYAKEDLHYIDDCKTITAET

PGDLCLKKLVGILDGLHKVEKNREIDTNTIES

>Ityp07

MKVLFAFVCLVVLIQVNSQTDKQKELLAQHYKECLAKSKVNEATLQKARIGQFADDDKLK

EHILCVAQKIGFQNSAGQFQNQVIETKLREALKGDAAKTKKLISDCAITNPDPKLQAFNA

FKCVYQKASINLL

>Ityp08

MKYLFLVIVSLLLAVQGEVSKEELEKLKEIHDTCLTESGVDQSMPEKAFKGEFTDDPKFK

EHLLCFHKK

>Ityp09

MTKLQIVLLLTTLMGSFNIITGEKKCNSSNCMYDRMLETVGKEFIEQCFKETGVTPEDIR

SVMEQNGYGEKQIVFPKMLDKENWYFGKRWSNQYRLY

>DponOBP03

MSINEIFVWRKLNIDLKMKSLVALFVCAFTATAMADAEINQSTFEAGRNRIMEMSRTCDE

NPATAVDQKALENYLESNGPAPANAGVHALCITKNLGWQNEDGSVNKPLITEKVKAIFGS

VDAKIERYIEDCTEAKAKPEDTAEQLLNCYRKHSPKTE

>DponOBP04

MGKQAGFINEAGDVLKDVLKEKSLKLFNDPALVQKLIDQCIVKKETPQETSYHAHVCLYK

NSPGHLALTQFGAISQEKKEKKVQIIKECAEESGVSRSAVLSARKGDFQDEPLLKQYFFC

INKKSQIQNEAGEYKTDVIRKGLTELFNAEEANRIIEKCARIQDSALNTAFQSFKCFYNE

APEITGVF

>DponOBP07

MKVFIVAFVVLGVVFAVNADLTEEQKQKIVANGKACVADTGADPELIKAARQGKFADDAK

LKAFALCMSKKIGFQNEAGEIQSDVVQQKLGSAIGDNEAAKKLVEKCLVSKGSGEETAIQ

SFKCYYENTPTHIAVF

>DponOBP09

MKVLVVLCIVLIAFTLIVSAKKNKSNDEEKPKSYKKVFKECQKKDETRVDASIIRKLKKH

KQVDLPANFGDHKLCVFKGIGLLKADNTVDEDKLKKKISSAKPQKDNVDSIFTECKSSKS

TLQETALNLDRCLTTNSIEF

>DponOBP11

MKSLVVFCALLVMALAHDPHGLDSVHKECHNEVASQHYLCMAKGLHLVTPEGKVNVNGVK

THAGHVVSESAKIDQIAKECAVDHASTEETVNHLFKCLEEKHVLSLAGHVAPQHHH

>DponOBP13

MFPAIKFLIVLGVVAVATRADRQQVVDFHRPCLDHHEIEDDDLHFALDKIKMRDDDEFYL

HFFCVAKQGQLMTEDGTVNTDNFETNMKGIIDEDNMENVAAIVRLCLIQKDTVLQTIRNA

VDCFMGKDHKL

>DponOBP19

MNGLCVFFLLLLAAVVKSDFDFSNYKEFEHLAGDQREKAISIFKECMAETGATHEMMEKS

VEGDIPDDIVFKNHLVCIGKKSGFIDENGLHSKEKLKEKLTLLLGDEGLVDKILDKCFME

KGTPQDTAFELAKCCHKEYHN

>DponOBP22

MSQKIHFALVAVFLTFLVNIIEADQREKAVEFQRGCMEAHGLLEDELHEIMDGKPIQNEA

FYFHFFCVVKKAKLISDNGIVNTDHFEENLKDVIDEEHMAHVAALTRKCLIQRDDIFTTI

KMAIDCFYSSEHKL

>DponOBP28

MKYLVVLSLCLAVVSAAALTKEEIKERLKAAHDKCQADPQTAIDEAALKAFKDSKGKGQL

PANMGPHDLCISKALKWQNADGKVNKELIKERITDNVADASKVDAIVNECAVDKENEIAT

AENLFKCLLKHHATAVHGH

>DponOBP26

MAKTTIVLCLMGIFCMRSVQPNPVRTHKLLSKSELHEIATSCLEEVQLSGSIVYNILKTE

IFPRDNNKYRDFLACSYKKQGFLSEDGTKLLYDNLFHFISHFYGPTEVQALKHCNLIRRE

DPGFLCFDTMKCIIDALKQLEFDANADIGIETNQVV

>DponOBP29

MKAMFVTLTVATVVVFASADLTEEQKQKIVANGKACVADTGADPELIKAARQGKFADDAK

LKAFALCMSKKIGFQNEAGEIQSDVVQQKLGSAIGDNEAAKKLVEKCLVSKGSGEETAIQ

SFKCYYENTPTHIAVF

>DponOBP30

MQLLFAAVLVIALVQVNSLTDKQKELLTQHYNQCVAISKVDQAVLQKARAGDFANDPNLK

THIKCISEKIGFQGTDGKFRRDVIEKKLKETIPGDNAKNAKLIETCVVANKDPKLQAFNA

FKCLYTNAKINLL

>DponOBP17

MALTTWVVSIMLILPAIRALSDEMKELAQMLHNTCVAETGVNEDFIRKVNAEKIFADDEN

LKCYIKCLMAQMACIDDDGIIDEEATIAILPEEYQALAAPVIRACGTKHGANPCENAWLS

HRCYAEMEPSVSG

>DponOBP32

MNQGCFLLVVSAVLVFAELDQTSLPPETKELMAALHKNCIEQIGVSEADVDQLRAANFEE

DAKLKCYTRCLMAESGVMDENGAIDVEAFAEILPEDIRGNIQTIFRRCSLTNKDIEDQCV

KAYEMVKCWHKEDPESYFMI

>DponOBP01

MFGFRKIFLVLVLVVFESLALQKNNKCDIPLSAPKRIEEVINTCQDEIKIAILSEALEAF

KVNEHKVVSRAKRSAFNEDEKKIAGCLLQCVYRKLNAVNEYGFPTVEGLVSLYTEGVTQK

EYVVATRQAVTKCLENAQKTHEISTKTVEASKSCEVAYEVFDCVSLEVAKYCGQTP

>DponOBP05

MKTALKVFLVALAIPTIMGMSDEMQELANQLHTTCIGETGAAEDAITNARNGDFSEADSF

KCYIKCLLSQMAIIDDNDGTIDVDAMVAVLPEEIQEATEPIIRKCGSIIGANPCDSAWLT

HKCYYKEGPEHYFLI

>DponOBP06

MFKSVSFLLLILALGSLDAKITLPPELQEYVDDLHKLCLEKGGLTENDHQTYNINDKNEK

MMCYMKCLMLESKWMKSGGEIDYDFIETQAYPEVRDLLLSALNKCRTIEEGADLCEKSYN

FNKCMYEADPVNWFFV

>DponOBP08

MDEPTFNRFAYLKANFPIGTQLHNTSPRICILTMKLLIFASILVCASALDQAWRDHMKEK

LTEFGLECAESEQATSEDIEALHNHKPPVTHAGRCVIFCVSKKLNLMNADGTLNVTPQSD

WIEKVKETDSEAFEKMKTVYHHCADTVEVEADACDTSLSYAHCIKEEGHKVGLYTVSAD

>DponOBP10

MLTKTILIWAAILLTVFISTGNCRLTEKQVAAAVKLVRNMCMGKSKVNPEDIDKMHQGNW

DVDYEAQCYMWCGFNMYKMLDKENHFDKKSALQQMEQLPTDLQDYVIKCMGQCENAVTNF

DDKCVVAFEYSKCLYFCDPEKYFLP

>DponOBP12

MKLMWILVLGAALKKADGAMTEAQMKAALKLIRNVCQPKNKATDAQIAAMHNGDWNQDKN

GMCYMNCVLNYYKLQLPDNSFDWETGLKVVESQAPPSMAGFIMETITGCKDAVKTRDDKC

KAAVEITKCLYDQNPEKYFLP

>DponOBP14

MPVTMNQGCFLLVVSAVLVFAELDQTSLPPETKELMAALHKNCIEQIGVSEADVDQLRAA

NFEEDAKLKCYTRCLMAESGVMDENGAIDVEAFAEILPEAVRGNIQTIFRRCSLTNKDIE

DQCVKAYEMVKCWHKEDPESYFMI

>DponOBP15

MMTAFLLCVLMAVVNQQVMGHPPRGPPGPPPFLGHPDPESANECRTEVGLTSEDRETKKN

GELTEKELCFIRCLGQKNGALSDAGALNIETIKNDLPDHLEDSEAVIACLKKVGTVTTCQ

HIKKVAKCYPEPKEPMDRT

>DponOBP16

MGPTILLLVGLVMMTNAYVPNVNNKIRDFCIDDSGVSIEMVENLLANPEKQLIDVESCYL

HCIFTEMGLLSENGNVEVEKFKSLKASEAPYIDLTCLEEIKSIDHCSEMMILRACHV

>DponOBP18

MALTTWVVGIMLILPAIRALSDEMKELAQMLHNTCVAETGVNEDFIRKVNAEKIFADDEN

LKCYIKCLMAQMACIDDNGIIDEEATIAILPEEYQALAAPVIRACGTKHGANPCENAWLS

HRCYAEMEPSAYMLI

>DponOBP20

MKQLFMVVLTALCAVHCKGLECGLSKISSEHFRKIASECVKDNETLNRIWELTSEASMDD

ESASSDEEVPITQGKEAPNLDLGSSPHKSMKMSRASRTKRSRKIFNNESPMSQRKPSPAS

TTTEQTTTVQSEENEDIADANNVEESGEVCLLQCIFEKLEMTDTNGLPDHKKFAAALVES

ATGRETRDFLKDSVDECFQETEEGDFEDSCEYSTKLVTCLAGRGKSNCADWPVGDLPF

>DponOBP21

MKQLFMVVLTALCAVHCKGLECGLSKISSEHFRKIASECVKDNETLNRIWELTSEASMDD

ESASSDEEVPITQGKEAPNLDLGSSAQKSMKMSRASRTKRSRKSFNNESPMSQRKPSPTS

TTTEQTTTIQSEENEDNADANNVEESGEVCILQCIFEKLEMTDTNGLPDHKKFAAALVES

ATGRETRDFLQDSVDECFQETEEGDFENSCEYSTKLVTCLAGRGKSNCADWPVGDLPF

>DponOBP23

MHCLRVCLIVFFSICGFSSSLKITLPPELQEYVDDLHKLCLEKGGLTENDHQTYNINDKN

EKMMCYMKCLMLESKWMKSGGEIDYDFIETQAYPEVRDLLLSALNKCRTIEEGADLCEKS

YNFNKCMYEADPVNWFFV

>DponOBP25

MSNLLKLSIAFAVVSVISCQDFTEEQRKKIIENRQQCIEETKVNPDLIEKADLGDFAEDQ

ALKCFTKCFYQKAGFVNDKGEVQKDVVEAKLPPQADKKRALEIVDKCALKGKDACETVYL

IHKCYFEHTHPEADEKTAKDGKSEEKKA

>DponOBP27

MNFSFIVALTLYLPISNGFLTVPKCLISTGARIKDLHNLATGDSLPESSRCFVKCVGEES

GLILDGTLHSEHFEALPMVSRLKADVFVDARRCIESVQGIKIESCKDIDNLNDCMKIVYR

QKYSDSK

>DponOBP31

MTFQGGVSVFLCILGVAQLVAAGNSDDLFARIAPADVEMCGKDTGVDRKEFEDAREKRAL

NHSMLCFLKCAMEKVGFLKDGHLEIDQAKGSLPDKMMEPVVECFKAVGPISTCDDIQKVE

DCLPSS

>DponOBP02

MNKLVTLYAVLLGAACHLVQTYDFQDATFNEILSSDFEDIFDTLDNTYLHPRAKRNEEAV

NSDEKCRRRHHRKPKLCCGEDVLDSLQEKEKEIVRLCFKDITGGVKESKPDRGFGNHRNF

DLFSCEAVEKRKSDMICVEQCKLQKQGLVSDDGSPKPEQISTYLKEAFTTQTWFEKVSQG

IVEKCVNEAINATKNPVKFYTEGNKLCSRSGIVLKHCLFNSIQLSCPAGQIKDKNACERF

QERAKKGKDLFDQPPGPPPFDDNREEQI

>DmelLush

MKHWKRRSSAVFAIVLQVLVLLLPDPAVAMTMEQFLTSLDMIRSGCAPKFKLKTEDLDRL

RVGDFNFPPSQDLMCYTKCVSLMAGTVNKKGEFNAPKALAQLPHLVPPEMMEMSRKSVEA

CRDTHKQFKESCERVYQTAKCFSENADGQFMWPX

>DmelOBP18a

MKVVCSIAVLWICLITMWQSAGRVNAEGCLKHHNLTSAQVQAVAPSTPVADVPVAVKCYS

RCLIQDYFGDDGKIDLQKVGKRGSQEDHVILSQCKQQFDGVTNLDTCDYPYLILQCYFKG

KQSGTIASX

>DmelOBP19a

MKFHLLLVCVAISLGPIPQSEAGVTEEQMWSAGKLMRDVCLPKYPKVSVEVADNIRNGDI

PNSKDTNCYINCILEMMQAIKKGKFQLESTLKQMDIMLPDSYKDEYRKGINLCKDSTVGL

KNAPNCDPAHALLSCLKNNIKVFVFPX

>DmelOBP19b

MMQCSRMTTTLKMTNLLLAVACAAVLMGSATADEEEGSMTVDEVVELIEPFGDACTPKPS

RENIVEMVLNKEDAKHETKCFRHCMLEQFELMPEDQLQYNEDKTVDMINMMFPDREDDGR

RIVKTCNEELKAEQDKCEAAHGIAMCMLREMRSSGFKIPEIKEX

>DmelOBP19c

MKPSTPVAAIPLMTIVVAVLLQTHCVRGQTQAFDLAKLLPKTGTEPIWAVIDRNLPQVQE

LVTAARMECIQKLQLPRDQRPLGKVTNPSEKEKCLVECVLKKIKLMDADNKLNVGQVEKL

TSLVTQDNKMAIAVSSSMAQACSRGISSKNPCEVAHLFNQCISRQLERNNVKLVWX

>DmelOBP19d

MSHLVHLTVLLLVGILCLGATSAKPHEEINRDHAAELANECKAETGATDEDVEQLMSHDL

PERHEAKCLRACVMKKLQIMDESGKLNKEHAIELVKVMSKHDAEKEDAPAEVVAKCEAIE

TPEDHCDAAFAYEECIYEQMKEHGLELEEHX

>DmelOBP22a

MRVLLAFVLLLGLSVLATKEPEEVKIVSECAKENNVHRKKALDLLMSYRLKKKTHNVMCF

INCIFERTNILQKVKEKVVKENHNCDSIKDADKCAESFQKFQCLVKIEMKVRGIDRGX

>DmelOBP28a

MQSTPIILVAIVLLGAALVRAFDEKEALAKLMESAESCMPEVGATDADLQEMVKKQPAST

YAGKCLRACVMKNIGILDANGKLDTEAGHEKAKQYTGNDPAKLKIALEIGDTCAAITVPD

DHCEAAEAYGTCFRGEAKKHGLLX

>DmelOBP44a

MKNAVAILLCALLGLASASDYKLRTAEDLQSARKECAASSKVTEALIAKYKTFDYPDDDI

TRNYIQCIFVKFDLFDEAKGFKVENLVAQLGQGKEDKAALKADIEKCADKNEQKSPANEW

AFRGFKCFLGKNLPLVQAAVQKNX

>DmelOBP46a

MCSQLFAFLLLLLTAFVTGRSTPPALDEDCELNSVDTMHDFCCDLHDESPQFSDCQMEWH

EKIPYETDEEEQTYMFCTAECSFNSTNFLGRDRRSLNLNEVKEHLESDLVNDADIKLLYD

TYVKCDKHALSLMPHKGVKQLSKRLSRLGCHPYPGLVLECVANEMILHCPTKRFRQTAQC

EETRNHLKQCMQYLKYKSX

>DmelOBP47a

MNRVLVLLLVLKMFALSESRFAKININLGLTVADESPKTITEEMIRLCGDQTDISLRELN

KLQREDFSDPSESVQCFTHCLYEQMGLMHDGVFVERDLFGLLSDVSNTDYWPERQCHAIR

GNNKCETAYRIHQCQQQLKQQQQNLLATKEVEVTTTPAGSDETKPX

>DmelOBP47b

MSPSQLLVIFASLALNTRLVFGQATIDCQRPPQLVDPALCCKDGGRDQVAEQCAQRILGT

ANGQKAGGPPSLDTAACLAECILTSSKYIDEPQKLNLANIRSDLSAKFSNDTLYVETMTM

AFSKCEPQSQRRLAMIMQQQQQVQQQKTQQQQPRCSPFSAIVLGCTYMEYFKNCPDHRWT

PNAQCTLAKAYVTQCGLGAX

>DmelOBP49a

MLSKSQLLLLVVGFCLNAAVSADVDCSKRPSFVNPKTCCPMPDFVTAELKQKCIKFDMTP

PPPPDGEASGSFESKRRHHHPHPPPCFFSCIFNETGIYQNRKLDEAKLNAYLQEVFEDSS

DLQTTATQAFTTCATKVADFEANLPPRPAPSPPPGFPMCPHDAGHLMGCVFRNMMKNCPD

SIRNDSQQCTDMKEFFTKCKPPRGPPPSAEDMX

>DmelOBP50a

MRTGRILVALIFLGLIIPFRAAKCRAAPKSVQNVHVCCSAPLPNWGVFNRECHKSAIQAS

CRLDCDFNASSVLQGNRLIQAKVRPMLERAFSNEPTIDAYESNFAKCSTVVRSKYQELSP

LSRQSDACDRHALFYSLCAYARLIFTCPDKMWQRNNRMCQEAKAYAKKCPWPALKMFMRN

TX

>DmelOBP50b

MSSVLHLLGFLWLPLLVYSVSNDMGGLQKCTELLNTHKLVYCCGKSFLDKFPFVGSNCTP

FWDDYGPCRYECLYRHWDLLDQDNKIKKPELYLMITSLYSPLNGYDKYGAAFKAAHETCE

ALGSRHADFLLLYSNQVADKMGMASSTCLPYAMLHAQCTMVYLTANCPRENWIDDPKCNS

LQKLLSSCTKKLDEKTNALKGKDEELTDNGCGHIDSEGSNLLMACFLTLMIAKFISDHX

>DmelOBP50c

MARHIALLICSLLAMAGCDPIDVDCTRRQDFNIVKDCCVYPTFRFDQFKSQCGKYMPVGA

PRISPCLYECIFNKTNTVVDGAIHPDNARLMLEKLFGNQDFEEAYFNGLMGCSDSVQEMI

SNRRSRPQRKTEQCSPFSLFYGICAQRYVFNHCPSSSWSGTESCEMARLQNMNCSKPSRG

SSHRLX

>DmelOBP50d

MLHKLTWVLIFIPAFRAADPICSQRPDVTALRNCCKLPNLDFSSFNSKCSQYLVNGVHIS

PCSFECIFRAANALNGTHLVMENIEKMMKTILGSDEFVHVYLDGFRSCGNQEKVLIKAMK

RRRVPITGKCGSMAIMYGLCAHRYVYRNCPESVWSKSATCNEAREYSIRCDDMX

>DmelOBP50e

MHKYIICFGFLLIILECSLASFNCSAPPNFNNFDINTCCRTPELDMGDVPQKCHKYVSGL

KSANSKYPSYAHLCYPDCIYRETGAMVNGKIKVNRVKQYLEEHVHRRDQEIVSHIVQSFE

SCLSNVKGHMKSLNIESYKVLPHGCSPFAGIIYSCVNAETFLNCPQQMWKNEKPCNLAKQ

FAEQCNPLPHVPLPSSX

>DmelOBP51a

MKVFIGLVLLLAVTTLSSALFESEANECAKKLGITPDYFENFPHSSRVKCFYHCQMEKLE

IIANGVVTPFDLKVLNISPESYDKYGVKVKPCLKLSHRDKCELGYLVFQCLKREFNLX

>DmelOBP56a

MNSYFVIALSALFVTLAVGSSLNLSDEQKDLAKQHREQCAEEVKLTEEEKAKVNAKDFNN

PTENIKCFANCFFEKVGTLKDGELQESVVLEKLGALIGEEKTKAALEKCRTIKGENKCDT

ASKLYDCFESFKPAPEAKAX

>DmelOBP56b

MKLIYLLVVFLIFALSELVAGQSAAELAAYKQIQQACIKELNIAASDANLLTTDKEVANP

SESVKCYHSCVYKKLGLLGDDGKPNTDKIVKLAQIRFSSLPVDKLKSLLTSCGTTKSAAT

CDFVYNYEKCVVKGISAX

>DmelOBP56c

MYFRASLMALLCLTLSEFVSKAWVMFFIFYISFTRSLSVSLNMSMTRTLVPDPPNGTENK

LSQEMLRACMRRTEISMSQLKLFHMSLMNSDYNNDNDIAPTPVQSIGDVNNLGDLDFNGN

SQMPYLDLKHNEPLQCFVSCLYETLDLDRYNVLLEEAFKNQVQTIIQHEKAEIKECSDLQ

GKTRCEAAYKLHLCYNHLKTLEAEQRIREILERTEAENEGFGPEGSDFIDGIQHSGEAMT

TAKSEX

>DmelOBP56d

MKFLIVLSVILAISAAELQLSDEQKAVAHANGALCAQQEGITKDQAIALRNGNFDDSDPK

VKCFANCFLEKIGFLINGEVQPDVVLAKLGPLAGEDAVKAVQAKCDATKGADKCDTAYQL

FECYYKNRAHIX

>DmelOBP56e

MKVFFVFAALAALSLASAVGLTDSQKAEAKQRAKACVKQEGITKEQAIALRSGNFADSDP

KVKCFANCFLEQTGLVANGQIKPDVVLAKLGPIAGEANVKEVQAKCDSTKGADKCDTSYL

LYKCYYENHAQFX

>DmelOBP56f

MKVFLLFIFISAIWLQAFCMKSSEKIKACLKRQLGYTITENTKFDAKEDSLQSKCFYHCL

LEVKGVIANDAISSEQPRKVLEKKYGITDTDELEKAEEKCHSIKASGKCELGYEILKCYQ

SITKHX

>DmelOBP56g

MRATFALTLLLGCLSGILAQANIDSSVSKELVTDCLKENGVTPQDLADLQSGKVKAEDAK

DNVKCSSQCILVKSGFMDSTGKLLTDKIKSYYANSNFKDVIEKDLDRCSAVKGANACDTA

FKILSCFQAANX

>DmelOBP56h

MKFTLFCIALAAFLSMGQCNPDFRQIMQQCMETNQVTEADLKEFMASGMQSSAKENLKCY

TKCLMEKQGHLTNGQFNAQAMLDTLKNVPQIKDKMDEISSGVNACKDIKGTNDCDTAFKV

TMCLKEHKAIPGHHX

>DmelOBP56i

MHFFTCCALLLVVVTLPTCFVQAGPIKDQCMAAAGITAQDVANRHETDDPGHSVKCFFRC

FLENIGIIADNQIIPGAFDRVLGHIVTAEAVERMEATCNMIKSETSHDESCEFAWQISEC

YEGVRLSDVKKGQRTRNHRGX

>DmelOBP57a

MFNTRLAIFLLLIVVSLSQAKESQPFDFFEGTYDDFIDCLRINNITIEEYEKFDDTDNLD

NVLKENVELKHKCNIKCQLEREPTKWLNARGEVDLKSMKATSETAVSISKCMEKAPQETC

AYVYKLVICAFKSGHSVIKFDSYEQIQEETAGLIAEQQADLFDYDTIDLX

>DmelOBP57b

MFIYRLVFIAPLILLLFSLAKARHPFDIFHWNWQDFQECLQVNNITIGEYEKYARHETLD

YLLNEKVDLRYKCNIKCQLERDSTKWLNAQGRMDLDLMNTTDKASKSITKCMEKAPEELC

AYSFRLVMCAFKAGHPVIDSEX

>DmelOBP57c

MLKLWLICILTVSVVSIQSLSLLEETNYVSDCLASNNISQAEFQELIDRNSSEEDDLENT

DRRYKCFIHCLAEKGNLLDTNGYLDVDKIDQIEPVSDELREILYDCKKIYDEEEDHCEYA

FKMVTCLTESFEQSDEVTEAGKNTNKLNEX

>DmelOBP57d

MPEKMSLRLVPHLACIIFILEIQFRIADSNDPCPHNQGIDEDIAESILGDWPANVDLTSV

KRSHKCYVTCILQYYNIVTASGEIFLDKYYDTGVIDELAVAPKINRCRYEFRMETDYCSR

IFAIFNCLRQEILTKSX

>DmelOBP57e

MLDQLTLCLLLNFLCANVLANTSVFNPCVSQNELSEYEAHQVMENWPVPPIDRAYKCFLT

CVLLDLGLIDERGNVQIDKYMKSGVVDWQWVAIELVTCRIEFSDERDLCELSYGIFNCFK

DVKLAAEKYVSISNAKX

>DmelOBP58b

MLRIGFVICVIISLRLNGLVAVRVHCRHMERIHEENIHHCCKHQDGHDDVTESCAKQTNF

RLPSPNEEAIVDVTVDQAMVGTCWAKCVFDHYNLMENNTLDMDKVRSYYKRYHQTDPEYA

TEMLNAYEKCHTQSEEATEKFLSLPIVRAFSTAKFCKPTSSIIMSCVIYNFFHNCPASRW

SNTTECVETLAFARKCKDVLTTMX

>DmelOBP58c

MKCTILLSFFSLIWFAGGIKIDCENTEAINEDHIHYCCKHPDGHNDLIEGCARETNFTLP

NQNEEALVDITADRAIRGTCFGKCVFSKLNLMKDNNLDMDAVRSLFTERFPDDPEYAKEM

INAFDHCHGKSEENTSMFLSKPLFKQMSKQFCDPKSSVVLACVIRQFFHNCPADRWSKTK

ECEDTLAFSKKCQDSLATLX

>DmelOBP58d

MVNIVCYWTFLILVAVSKAQDNEETTAVAISSGDLTEDKCNTSRAGCCSELYIGEEEDLV

KCFVIHSPKLPVDGDADIGKTLRFLSCFVECLYKQKKYIGKSDTINMKMVKLDAEKTFVD

RPKEKDYHIAMFEFCRKDAVGVYNLLKASPGAKVLLKGACRPYLLMVFMCISDYHQKHEC

PYFRWEGTAKAGTKDMCENAKAECYQIDGITLPTKSPAX

>DmelOBP59a

MKQLIFLLICLSCGTCSIYALKCRSQEGLSEAELKRTVRNCMHRQDEDEDRGRGGQGRQG

NGYEYGYGMDHDQEEQDRNPGNRGGYGNRRQRGLRQSDGRNHTSNDGGQCVAQCFFEEMN

MVDGNGMPDRRKVSYLLTKDLRDRELRNFFTDTVQQCFRYLESNGRGRHHKCSAARELVK

CMSEYAKAQCEDWEEHGNMLFNX

>DmelOBP69a

MVARHFSFFLALLILYDLIPSNQGVEINPTIIKQVRKLRMRCLNQTGASVDVIDKSVKNR

ILPTDPEIKCFLYCMFDMFGLIDSQNIMHLEALLEVLPEEIHKTINGLVSSCGTQKGKDG

CDTAYETVKCYIAVNGKFIWEEIIVLLGX

>DmelOBP73a

MRITQLLCISCMVITSIDAVEYLIRFETKKAKCLNPPRTARKVESVIRECQDEVRNKLVN

EAYEILKEQVSQNQPPIDPNDDSIDFIWPSVPEAPSLDHSPNISQYEYIVYDEPEPQRHV

ARLMRNIRRLDVASSGIYHPTLVPLEDKRIAGCLLHCVYAKNNAIDQRGWPTLDGLVHFY

SEGVHEHGFFMATLRSVNLCLRTMTARYGVNRKELPKKGESCDLAFDVCSHMNTNIFKDL

QFWAPFISYVNESRAINYIX

>DmelOBP83a

MALNGFGRRVSASVLLIALSLLSGALILPPAAAQRDENYPPPGILKMAKPFHDACVEKTG

VTEAAIKEFSDGEIHEDEKLKCYMNCFFHEIEVVDDNGDVHLEKLFATVPLSMRDKLMEM

SKGCVHPEGDTLCHKAWWFHQCWKKADPKHYFLPX

>DmelOBP83b

MVKYPLILLLIGCAAAQEPRRDGEWPPPAILKLGKHFHDICAPKTGVTDEAIKEFSDGQI

HEDEALKCYMNCLFHEFEVVDDNGDVHMEKVLNAIPGEKLRNIMMEASKGCIHPEGDTLC

HKAWWFHQCWKKADPVHYFLVX

>DmelOBP83cd

MQMKSGILIALCLCLSLNEGLALLEHEGETINRCIQNYGGLTAENAERLERFKEWSDSYE

EIPCFTRCYLSEMFDFYNNLTGFNKDGIVGVFGRPVYEACRKKLELPFESGESSCKHAYE

GFHCITNMESHPFTVIDNMPNISPSAKDAMKDCLQDVHQDEWKSFDAFAYYPVNEPIPCF

TRCFVDKLHIFEEKTRLWKLEAMKQNLGIPAKGARIRTCHRHRGRDRCATYYKQFTCYAM

AVX

>DmelOBP83ef

MSSPRAVLVSLFLICSQALADLSGDAQTLEKCLRQLSSPESIAGDLRKLERYSSWTREEV

PCLMRCLAREKGWFDVEENKWRLKQLTEDLGADVYNYCRFELRRMGSDGCSFAYRGLRCL

KQAEMHAGTSLSTLLQCSRQLNATNVELLQYSKLKSKEPIPCLFQCFADAMGFYDPDGNW

RLENWKQAFGPSGNEDQSSGADYSGCRLSGTQREVALSKCSWMYHEYKCWERVNGNKLVE

DNEEQX

>DmelOBP83g

MQSQSLLLIVAAVATFLVAQTTAKFLLKDHADAEKAFEECREDYYVPDDIYEKYLNYEFP

AHRRTSCFVKCFLEKLELFSEKKGFDERAMIAQFTSKSSKDLSTVQHGLEKCIDHNEAES

DVCTWANRVFSCWLPINRHVVRKVFAX

>DmelOBP84a

MYSALVRACAVIAFLILSPNCARALQDHAKDNGDIFIINYDSFDGDVDDISTTTSAPREA

DYVDFDEVNRNCNASFITSMTNVLQFNNTGDLPDDKDKVTSMCYFHCFFEKSGLMTDYKL

NTDLVRKYVWPATGDSVEACEAEGKDETNACMRGYAIVKCVFTRALTDARNKPTVX

>DmelOBP85a

MSPGSVVFSMFLTRPSLDKGNSECRKSLNLPAHRKFNFAELYTINMCIEECNFIGCGYIE

IDPPFRLDLANIRTNLQTIAPQPQNESIPFLVDAYRKCELFRSSHGRRFTLHLPDIEFIE

EPCNPFALQITICVRIHAMQKCPSEFYVDSDECRLAREYFTQCVGDIETNLAX

>DmelOBP8a

MMRRSQIGLLSRLLLLLLVVELTPPAIPVPMRSSPQSLALLRARDQCGRELTAAQRLQLD

RMQFEDAAHVRHYLHCFWSRLQLWLDETGFQAQRIVQSFGGERRLNVEQALPAINGCNAK

TSSRGSGAQTVVDWCFRAFVCVLATPVGEWYKRHMSDVINGNAX

>DmelOBP93a

MYVYNLLFVVIVFSYCAKSFNYTSCDHAKQPKFLSSCCDVQKNDKAINSCRKSLLGNNST

NSNGEVRNLKSDKVALHACIAECSFRTNGFLLSNGTVNTQALQKSYQQRYKNDPNMSQLM

LKSLNSCTDYARKRVQEFQWMPKKGDCDFYPATLLACVMEKVYINCPTSKWKNTSDCTAM

WKYLVACDDVASNKKKX

>DmelOBP99a

MKVFVAICVLIGLASADYVVKNRHDMLAYRDECVKELAVPVDLVEKYQKWEYPNDAKTQC

YIKCVFTKWGLFDVQSGFNVENIHQQLVGNHADHNEAFHASLAACVDKNEQGSNACEWAY

RGATCLLKENLAQIQKSLAPKAX

>DmelOBP99b

MKVLIVLLLGLAFVLADHHHHHHDYVVKTHEDLTNYRTQCVEKVHASEELVEKYKKWQYP

DDAVTHCYLECIFQKFGFYDTEHGFDVHKIHIQLAGPGVEVHESDEVHQKIAHCAETHSK

EGDSCSKAYHAGMCFMNSNLQLVQHSVKVX

>DmelOBP99c

MLKYLIVALALCAVAHADDWTPKTGEEIRKIRVDCLKENPLSNDQISQLKNLIFPNEPDV

RQYLTCSAIKLGIFCDQQGYHADRLAKQFKMDLSEEEALQIAQSCVDDNAQKNPTDVWAF

RGHQCMMASKIGDKVRAFVKAKAEEAKKKAAX

>DmelOBP99d

MNHLRLEIICWSCLLIAMAVSTEAASVWKLPTAQMVYEDLEKCRQESQEEDAATLRCLVK

KLGLWTDESGYNARRIAKIFAGHNQMEELMLVVEHCNRMEQDTSHLDDWAFLAYRCATSG

QFGHWVKDFMSQKEVERX

**CSPs**

>DmelPhk-3

MKASLALVFCVCVGLAAAAPEKTYTNKYDSVNVDEVLGNNRVLGNYLKCLMDKGPCTAEG

RELKRLLPDALHSDCSKCTEVQRKNSQKVINYLRANKAGEWKLLLNKYDPQGIYRAKHEG

H

>DmelEbpIII

MKMILALVVLGLVLVAAEDKYTTKYDNIDVDEILKSDRLFGNYFKCLVDNGKCTPEGREL

KKSLPDALKTECSKCSEKQRQNTDKVIRYIIENKPEEWKQLQAKYDPDEIYIKRYRATAE

ASGIKV

>DmelCG30172

MLLLNKNRVISLVVNFIFLIILISSSVQADERNINKLLNNQVVVSRQIMCILGKSECDQL

GLQLKAALPEVITRKCRNCSPQQAQKAQKLTTFLQTRYPDVWAMLLRKYDSA

>DmelA10

MGQPGFRRAIGHVSLVVALMCTTCFQVEGLPHPPATSPSPMMERMVEQAYDDKFDNVDLD

EILNQERLLINYIKCLEGTGPCTPDAKMLKEILPDAIQTDCTKCTEKQRYGAEKVTRHLI

DNRPTDWERLEKIYDPEGTYRIKYQEMKSKANEEP

>TcasCSP20

MRFFVIFFVACVSVALARPEDQYTIKYDNVNLKEILQSDRLTENYVNCLLEKKPCTPDGE

ELKRVLPDALKTSCAKCTDKQKQGAKTVIQHLYKNKQDWWKQLEAKYDPEHTYVKAHEDE

LKAL

>TcasCSP19

MKFFIAFLMLLGAVWCEQYTTKYDNINVDEILASERLLKNYFNCIMDRGACTPDADELKR

VLPDALKSDCAKCSEKQKEMTKKVIHFLSHNKQQMWKELTAKYDPDGIYFEKYKDKFDS

>TcasCSP18

MLFTVFLVLTCAHVVFLEEYVIPDNIDIDDILSNERLLKNYVNCLLDKGRCTPEGKKLKS

TIPEALSTDCAKCNEKVKANVRKVLHHLIDNKPDMWKQLEAKYDPSGEYRSKYKDELEKN

GIHV

>TcasCSP17

MFKVLFVVFACVQAYVYAEEYTVPQNIDIDEILKNDRLTKNYLDCILEKGKCTPEGEELK

KDIPDALQNECAKCNEKHKEGVRKVIRHLIKNKPSWWQELQEKYDPKGEYKSRYNHFLEE

EGLN

>TcasCSP16

MTAIVFLLALACLKTYVSSQEYLVPQNIDVDEILKNDRLTRNYLDCVLGKGKCTPEGEEL

KKDIPEALQNGCAKCNEKHKEGVRKVIHHLIENKPNWWQELESKFDPQGEYKKKYDELLK

KEGLAN

>TcasCSP15

MIFKIHFLVFGALLTYVSSVEYLILREIDTILKNDQMTRNYLDCVLDKGKCTKEAEKLKK

GITETMKNGCVKCEQKQKEDVHKVFQHLMIHRPNWWHELETKFNPHHEIKLQHLHQSKFN

PHEEVKLQHLHQFPHHDFLEREGFIR

>TcasCSP14

MFATSALFAFICIQGLVSAEEYLVPQNIDLDEILKNDRLTRNYIDCILGKGKCTPEGEEL

KRDIPEALQNECAKCNEKHKEGVRKVLHHLIKNKPNWWQELEAKFDPKGEYKQKYNKLLE

KEGLQA

>TcasCSP13

MFLAIVLVVCACTNVLSEEYTNQYNDELDAALKSERLMKSYFECLLGTGKCTPSGEELKK

DIPDALKNECAKCNDKHKEGIRKVIHYLVKQKPEWWEQLQKKFDPQGIYKKRYQNYLDKE

GLKA

>TcasCSP12

MKTLVLVLFVAVLSVVFAADKYTTKYDNIDLNQILKSDRLLKNYVNCLLDRGKCSPDGQE

LKNNLADALQTSCSKCSQRQKDGSRTIIRYLIKNKRDWWNELEAKYDPTGIYKNKYADEL

KAEGIVL

>TcasCSP11

MKTLVPLLFFVIAIASSLAENSKYTTKYDNVDLDEIIKSDRLLKNYVNCLLEKGKCTPDG

AELKRHLPDALHTECSKCSETQKNGSKKIMRHLIDHKRDWWNELEEKYDKEGEYRKKYEA

EIKGKKD

>TcasCSP10

MKTFVLVAFAAVLGLALARPQEKYTTKYDNIDLEEILKSDRLLKNYFNCLMERGTCSPDG

EELKKALPDALHSGCSKCTEKQKEGSRKIIHYLIDNKRDWWNELEAKYDKDGVYRQKYKD

VIEKEGIKL

>TcasCSP9

MTAIVFLLALACLKTYVSSQEYLVPQNIDVDEILKNDRLTRNYLDCVLGKGKCTPEGEEL

KKDIPEALQNGCAKCNEKHKEGVRKVIHHLIENKPNWWQELESKFDPQGEYKKKYDELLK

KEGLAN

>TcasCSP8

MPLVKSLVVVVLLIGVVYQVQGQLGLAGNNYIEKQLLCALDKAPCDALGNQIKGALPEII

GKNCERCDSRQVANARRIARYVQTKHPDVWNALVKKYSV

>TcasCSP7

MKLISAVILCAFLVAVSAAENKYTNKYDNVDVDKILNNDRVLTNYIKCLMDEGPCTSEGR

ELKKTLPDALSSGCTKCNQKQKETAEKVIRHLTQKRARDWERLSKKYDPQGQYKKRYEEH

VATSRAA

>TcasCSP6

MIPLIAIAGILAVSAAPAEFYESRYDHLDVESILNNRRMVNYYAACLLSKGPCPPQGVDL

KRVLPEALQTNCAKCTEKQRTAAYRSIKRLKKEYPKIWEQLRAVWDPDDVFIRKFETSFE

SGKPSGVISTNTSPPSPILSNRFGENEEADAASNVISSTPLPPTTSTTTRTTLTTKFTTK

PSTKPTNKPVVVTKPPQAPPFATVGANLQATVSFGTNLVGGIVRSLGTLGSRVVESGTKL

ANMVISAAIRP

>TcasCSP5

MKTFVILFFGVFFIIFSDFVNGKTLHRSTRDDKYTTRYDNVDVDRILHSKRLLLNYINCL

LEKGPCSPEGRELKKILPDALVTNCSKCSEVQKKQAGKILTFVLLNYRNEWNQLVAKYDP

DGIYRKQYEIDDDYDYSELDSAKK

>TcasCSP4

MYSYLIPLYLFLFVHYGWSEDTTHKYTTKYDNIDLENVVKNERLLKSYVDCLLEKGRCSP

DGLELKKNMPDAIETDCSKCSEKQKEGSDFIMRYLIDNKPDYWKALEAKYDPDGTYKKRY

FESQKDEVSKVEA

>TcasCSP3

MLFTVFLVLTCAHVVFLEEYVIPDNIDIDDILSNERLLKNYVNCLLDKGRCTPEGKKLKS

TIPEALSTDCAKCNEKVKANVRKVLHHLIDNKPDMWKQLEAKYDPSGEYRSKYKDELEKN

GIHV

>TcasCSP2

MKIIILAVLIATAVAATYDVYPTKYDNVDIDAILHNKRLFDNYLQCLLKKGKCNEEAAIL

RDVIPDALITGCRKCNDHQKVSVEKVIRFLIKERNSDWQQLISVYDPKGEYQTQYAHYLE

KI

>TcasCSP1

MLILQIAHLCAQFCLLAAIFTCVKPQLTRISDEAIESTLNDRRYLLRQLKCATGEAPCDP

VGRRLKSLAPLVLRGSCPQCTPQEMKQIQKVLAFVQKNYPKEWNKILHQYAG

>ItypCSP1

MKLIISFLLIAVAALSYADKYTSKYDDVDIDQILQSERLLRNYLNCLLDKGRCTPDGAEL

KKNLPDALENECSKCNESQXKGASKVIRYLIDNKRQYWDELAAKYDPEGVFFKKYEAEAK

KDLLDQIGRA

>ItypCSP2

MGGHRKSYLVLALVLVNLVSLNRAAESTTRAPISDDALEKTLSDKRYLTRQLKCALGEAP

CDPVGRRLKS

>ItypCSP4

MALLIFFVVILTVGLASAKPAVKHYASKYDHIDVETILNNPRMVKYYSACLLSQGPCPPE

GVEFKRILPEALHTNCHRCTEKQATVTLRAIKRLKKEYPKIWSQLSQMWDPDDVYVRKFE

STFGNRNKIPSVVVNNGWDLGSSTTSNADEPRPDTTTHQIITSPNIMSFTTSKTSSTPIT

TSSTANPSTKTSTTTVGTTTKPPSRPAPIPGLLP

>ItypCSP5

MQCLGLFVVLVLGCSLVAAQSPYTSKYDNVDVDKILKNERVLTNYIKCLMEEGPCTPEGR

ELRKTLPDALASGCSKCNEKQKDTTEKVIRHLMDKRTKDWDRLSKKYDPQGVYKQRFEKE

LSARKLA

>ItypCSP6

LIGLAPLVLRGSCPQCTEQEKKQIKKVLAYVQVNFPKEWNKMLQTYASG

>DponCSP1

MKVVLLLVVVVGVAFGEEYTSKFDNVDLDQILSSDRLLRNYINCLLEKGKCTPDGTELKK

NLPDALENECSKCTPKQRDGAKKVIRYLIENKRDYWDEVAAKYDPEGTYYKKYQEQAKKE

NIKL

>DponCSP2

MKFCVVLVLVLQIAICLGQTYTSRFDNINIDEILSNKRVLNNYVRCVLDEGPCTAEGREL

RTHIPEALRTSCAKCTPSQQKFVRKGANFLIKNDPDQWKRIAKKFDPEGKFAPQFRQFLN

A

>DponCSP3

MWKLVLLGSLLICIGQTLAEVTEKSQYTTKYDNVDINEVVHNERLLKNYVNCLLDRGPCS

PDGLELKKNMPDAIETDCSKCSDKQREGLEAMMRFLIDNKPEYWNPLQEKYDPTGSYKKR

YLDAKRAEVAIQPAEKTP

>DponCSP4

MHCASVFFVVSALLVLISAQSSPYTSKYDNVDVDKILKNDRVLTNYIKCLMEEGPCTPEG

RELRKTLPDALASGCSKCNEKQKSTTEKVIRHLQTRRAKDWDRLSKKYDPEGVYKQKYTA

ELKTETTA

>DponCSP5

MWKLVLLGSLLICIGQTLAEVTEKSQYTTKYDNVDINEVVHNERLLKNYVNCLLDRGPCS

PDGLELKKNMPDAIETDCSKCSDKQREGSEAMMRFLIDNKPEYWNPLQEKYDPTGSYKKR

YLDAKKAEVAIQPAEKTP

>DponCSP6

MKTIIFLVVVASFYGLSSCKPQEKYTTKYDNIDLDAIIRNDRLLRNYIDCVLGKKKCTKD

GEELKVHLPDALQSDCSKCSEAQRNGSRKIITHLLKNKRGWFNELQAKYDPAGNYLSKYS

EELRKEGIVI

>DponCSP7

MVPPGSFVLIAVLTVLMMDEENGANARRVKRSAQTYTTKYDNIDIDQILASNRLLKNYVN

CLLDKGGCTQEGKELKKYLPDAIATECSKCSQTQKKIAGRVFQALLLNHRDDWELLTNKY

DPEGNFQKKYLQEDEDYSDLEEA

>DponCSP8

MKIFIVVCCAFIGLVLADTPKYTTKYDNVDLEEIIKSDRLMKNYVNCLLEKGKCTPDGAE

LKRVLPDALHTECSKCSDSQKKGSRKIMRHLIDNKPEWWTELENKYDKEGAYKKQYREEL

KKDGIKL

>DponCSP9

MNSHCFQLQLIALLVLVALVSLVRNETTERPAISDEALEKTLSDKRYLQRQLKCAVGEAP

CDPVGRRLKSLAPLVLRGSCPQCTEQEKKQIKKVLAYVQVNFPKEWNKMLQTYAG

>DponCSP10

MKVVLLLVVVVGVAFGEEYTSKFDNVDLDQILSSDRLLRNYINCLLDKGKCTPDGIELKK

NLPDALENECSKCTPKQRDGAKKVIRYLIENKRDYWDEVAAKYDPEGTYYKKYQEQAKKE

NIKL

>DponCSP11

MAPFPQSWLQFGALLLLLALVQGQILNGNVYVEKQLLCALDRAPCDNLGRQIKDALPEII

GKNCKACDNKQLSNAKRIARFVQNKYPNVWNDLVRKYGNPTN

**SNMPs**

>TcasSNMP2

MGCSCCTIKVLLVCVVISVALLIVSLALAFKVFPDLLESEVNKAVRLEDGTKQYDRFVEL

PFPVDFKVYLFNVSNPQQVLDGTEKPKLEEIGPFVYKQYRKKTILGKNEEEDTISYTQKE

TFEFDAEASKPLTEESVVTVLNPALMSIYQLAEDLHLAGAADTCIKQTFENNQGKVFIEA

NVRKLLFDGFSFCKNTSPGICGLVNDLICAIAATKRNSDLVLPDYSLIFSYLNYKRKPDD

GKYTVKRGLTNIEKLGHIVAWNDSLYTKFWGEGTTCSEVKGTDSTLYPPRVTTDSAFYIY

STDICRFVKINYKGEESYKGIDGYLFETSEDTLRSSAPEEDCYCSKLSRDMEGKKSCFLD

GVIDMQTCFGVPVLFSFPHFLWADNKYLSAVEGLNPVEEKHKTYLVVEPNTGTPLKGMKR

IQLNGVIRPIVGIKSMLQTKRALLPLLWIEEGVSLPQKYVDELKSSYFDKVQIVDGVRYA

LIVISAILVGAFGIIILRKRSHAKHHV

>ItypSNMP2

MRFLQRVKFNLKTVFLCGISGVSLLVVALFLGFIIFPKVVNDQLLETKILREDTEQWAIF

KKIPFAFTFNVYLFTVENPEEILKGAKPVVKEKGPYVYKLYKWKEDIIWNYTTDEISYYE

YEKYVFDQEASGSLTEHDKVTLLNLPYLTFLYTAEANEATSGFLPLIDEALEFIFSGHNS

PFLVNVTVRDYLFEGVEICKNGCEDDGFVAKMACGKIKDNLKVAKQMRLHHKDILFATFH

YRNNTHQKYLTVNSGRQNHLEIGAITQLDNSSTMNVWNQFGCNQVSGLTGIFPINLGFKT

TFQSFSAEICRPVKLHFSTIKPFGSIKGYKYVALNTTFNTSMVENQCYCTGKIPNLDGNL

GCLYDGVLDLSTCLGAPIVVSFPHFLYADWRYVNNVKGLSPNETNHQIFVNLEPISGTPL

EAATRIQFNLFLRPVRNITSLDSVADALVPLFWIEELTYLPQKYQDVITGKLYRSIFILN

AIKYVLLAIALVIITVCILIFLYTD

>DmelSNMP2

MIHWSLIVSALGVCVAVLGGYCGWILFPNMVHKKVEQSVVIQDGSEQFKRFVNLPQPLNF

KVYIFNVTNSDRIQQGAIPIVEEIGPYVYKQFRQKKVKHFSRDGSKISYVQNVHFDFDAA

ASAPYTQDDRIVALNMHMNAFLQVFEREITDIFQGFANRLNSRLNQTPGVRVLKRLMERI

RGKRKSVLQISENDPGLALLLVHLNANLKAVFNDPRSMSVSTSVREYLFDGVRFCINPQG

IAKAICNQIKESGSKTIREKSDGSLAFSFFGHKNGSGHEVYEVHTGKGDPMRVLEIQKLD

DSHNLQVWLNASSEGETSVCNQINGTDASAYPPFRQRGDSMYIFSADICRSVQLFYQTDI

QYQGIPGYRYSIGENFINDIGPEHDNECFCVDKLANVIKRKNGCLYAGALDLTTCLDAPV

ILTLPHMLGASNEYRKMIRGLKPDAKKHQTFVDVQSLTGTPLQGGKRVQFNMFLKSINRI

GITENLPTVLMPAIWVEEGIQLNGEMVAFFKKKLISTLKTLNIVHWATLCGGIGVAVACL

IYYIYQRGRVVEPPVK

>DmelSNMP1

MQVPRVKLLMGSGAMFVFAIIYGWVIFPKILKFMISKQVTLKPGSDVRELWSNTPFPLHF

YIYVFNVTNPDEVSEGAKPRLQEVGPFVFDEWKDKYDLEDDVVEDTVSFTMRNTFIFNPK

ESLPLTGEEEIILPHPIMLPGGISVQREKAAMMELVSKGLSIVFPDAKAFLKAKFMDLFF

RGINVDCSSEEFSAKALCTVFYTGEIKQAKQVNQTHFLFSFMGQANHSDSGRFTVCRGVK

NNKKLGKVVKFADEPEQDIWPDGECNTFVGTDSTVFAPGLKKEDGLWAFTPDLCRSLGAY

YQHKSSYHGMPSMRYTLDLGDIRADEKLHCFCEDPEDLDTCPPKGTMNLAACVGGPLMAS

MPHFYLGDPKLVADVDGLNPNEKDHAVYIDFELMSGTPFQAAKRLQFNLDMEPVEGIEPM

KNLPKLILPMFWVEEGVQLNKTYTNLVKYTLFLGLKINSVLRWSLITFSLVGLMFSAYLF

YHKSDSLDINSILKDNNKVDDVASTKEPLPSANPKQSSTVHPVQLPNTLIPGTNPATNPA

THHKMEHRERY

>ItypSNMP1

MPHPKNIAWAGGALAFGGVLFKVWLFDVLVRFGVKDQTALRYRNEVRGIYLKIPFPLNFK

IYFFNVTNPEEIQNGAKPVLNEVGPYWYDEYKERVDVIDNDTEDSLTYTPYDLFKFNPNM

STPLSDNDYVTIIHPVIVGMVNLLLRDSPMLLKVVSKAIPFIFNDPKTIFLTGRVKDILF

DGVVLNCTSKEFASTAVCGQMKGQVPGLKPTPGQPNLLLFSLLGPRNATRTGSLKVLRGI

KHFQDLGRLLEVNGRKSIGIWAGDQCNRYDGTDSWIFPPLIQPESGLKSFSTDLCRNIKM

KLVNETVVKKIPVGVFEPTWGVKVVTRRKSATVPTLPVXXXXXVFDLTKCMGVPLYATLP

HFLDTDPNYLKLVDGLKPDHEKHRIVVFFETMTGTPLKAAKRMQFNLELQQTNKLELFSK

LPAALFPIFWLEEGMELEGYFLKKIQTVFMLLLFADVTIYVTIATGLSVCGAGFYQYWKN

TKSLSITPLTKNNNGLSEPKLN

>DponSNMP1

MMLSNKIWSSSRFLYGSVILLVSSVLLKLWLFESMVKFVIRDQTALRKRNQVREVYLKIP

FPLNFKLYFFNVTNPEEIQTGSKPKLKEVGPFWYDEIKEKVQIIDNDTEDSLTYTPYDLF

EYNQNKSNQLREDDYVTIIHPAIVGMVNLVLRDSPVFLSIVSKAIPSIFNNPQTIFLTAK

VKDILFDGVELNCLGKDFGTTAVCSQMKSQIPGLKFKKDNENIFLFSLLGSRNGTLTRRL

KVHRGIAHAKDLGRLVELDGKKEINIWRQAECNRFHGTDGWIFPALSTPEEGLPSFSTDL

CRSVNLRYINDTVLKKIPVRIYETDLGDQMTDENEKCYCRSADSCLKKGVFDLSKCMGVP

IYATLPHFLRTDPSYINLVDGLAPSELLHAIRVYFEPMTGTPLFAAKRMQFNLDLKPTNK

IPLFSHLPTALFPMFWLEESVDLDGYLLKKVQTVFLLLHAVDIIQYLMIVIGCGCVTISM

YFRLKNRKSVTITPATGSKKSAPPKPIDEMDVSHLSIAGILGDRPQKKAVVSQVMSGHEF

DKY

>ItypSNMP1a

DLFKCAGVPLYASAPHFYDCHVSYLKGVRGLHPDEQKHAIKILFESLTGSPVYAKKRLQF

NMPLEPNQKIDMFKNITPTVLPLFWIEEGVKLNNTYTKPLKSLFMMKKIVGVVKYLILLG

SIAGVTVGVYLYFKSGDTVNVQ

>DponSNMP1a

MNFPMRLAIGSACSLLFIILVGFVGFPKMIKGKVKDMVNLKPGMEIREMFVKVPFPLSFN

VYIFSVLNPAEVQGGAKPHLKEMGPFCYNEWKTKINVEDNEGDDTISYDPVDTFENAKRP

KCLSVDTLVTIPHPMILGMVNTILRQKPGALTLANKAIKSIWSNPSSLFITVKAQDLLFD

GVVIHCGVSDFAGKAICTNLKAEPSLTHLGEDDLGFSLMGPKNGTAGKRIKAFRGTQDFH

KVGRIIEFDGKSKLDVWNNSKCDTIVGTDGTIFPPMLKKEEGLASFAPDLCRSLIAQFDK

HDKYDGIPVSSFFASLGDQSKNPAEKCFCTTPETCLKRGLMDLYRCAKIPLYVSLPHFYD

SHESYLKGVKGLKPDVEKHGIRIMFELLTGSPLSARKRLQFNMPLEPNPKVELFHNFTPT

VLPIFWVEEAVDLNSTFTKPLKTLFLTKKLVNIVKYLVLLMSIGGFCAAVYLYFKSDDSM

NVTSVQKVQPDQNGHRNIISTVFNGNHTAGQDNEAYEDKY

>TcasSNMP1

MRLPVKIAIGCAIGLVVIIVFGFIAFPKMIKGKVKSMINLNKGSEIRQMFVKVPFALDFK

IYMFNVTNPMDVQKGALPVLKEVGPFCFEEWKEKVDLDDNDDEDVMFYNPKDTFYKANGP

GCLDGSQMITMAHPLILGMVNTVVRTKPGAISLISKAINSIYGNPDSIFMTASAMDILFD

GVVIKCGVKDFAGKAVCSQLKEAPDLRHVDENDLAFSFIGPKNATPGKRFKVLRGVKESH

DVGRILEYDNKKEMEVWPTKECNQYKGTDGTVFPPYLTKEEGLASYAPDLCRSLVAVYSG

DTKYDGIPVRIYTATLGDMSKNADEKCYCPTPDTCLKKGMMDLFKCAGVPVYVSLPHFYE

SDESYVKGVVGLNPNKKDHGIQILFESTTGGPVKAAKRLQFNMPLEPNPKLPIFANLPNT

VLPLFWVEEGVALNNTFTKPLKDLFKIMKIVKIAKWLIMLGCLGGLGAAGYLYFSKKGEA

NITPVHKVKPAENGVSTLGGEVNHAMSDNEIEKY

>DponSNMP2

MFRNCCSPRLVFLYNLLAVLLLIASLVLAFWGLPQIISKQIHKQTELTENTDQWDRFKEL

PFPMEFNIRFFLVTNPADVLNGSMPILKESEPYKYKSTIKRTDIRFDDIEEDSVTYRRSF

SFEFDGSGTTREDDSITVINPLLMASFQLTNDIQRLAMAGCRKYILEPAGLDQVFLTTTV

RKLLFDGIYFGFQNATGKGVACEMVRKELGKIVANVRVVEHLNDTDCYRLAIFNYKTDNF

LKNSPDGIYTINRGRNNATALGSIMRWNGATTSTTYGTSTSINNLTCHSIKGTDSTIYSP

ELKAGENLMIFNTDLCRTIQLVQVSSNEVFNGINAFRYSTGYTLFRPETILKENDCYCSH

GTKGADGKPSCFLDGLLDFRPCLGAPVLISQPHFLHADVKYIRAVSGLSPDEDKHDIYLL

LEPNTGTPLEGRKRVQMNSVLRRQPLLSMITPPNMYEAVVPLLWLDEGFTLPQKYLDDLN

AKYFKTVRIATGFKFGFIAVALALLVGCLFVACRKMYFRNAK

**ORs and GRs**

>AglaGR1

MHGFDPLPQEGRHRRHPGQDQHLKVSEVIQVSGTPASGIRVSNADPELLHQYDNFYHTTKSLLVLFQIMGVMPIERDIGKTTYRWTSATNIWAYFVYIVETIFVTIVFKERLELVLLPGKRFDEYIYAIIFLSILIPHFLLPIGAWTNGHEVAKFKNMWTKFQYKYFTVTGTPVVFHNLTLISYSLCVLSWVIGIVVMLAQYYLQPDMLLWHTFGYYHILAMLNCLCSLWFINCTAKGRVAGWLAENLHNALQSADAANKLAEYRDLWVDLSHMMQQLGTAYSGLYGMYCVLILLTTIVASYGCLTEILDHGLSFKEAGLFLISFYCMSLLYIICNQAHYASSKMGPEFKERLLNVNLAAVDSRTRQEVNMFLTAIDKNPPVMTLNGYANVNRKLISSTVTSMATYLVMLMQFRLSLMRNAAIAARRSAAANATSFGNATGH

>AglaGR2

MEIKDLADLYGNELHIRQLKKLIRGSARAQEIEKRSKLDSSDGHVIDEHDQFYRDHKLLLALFRVLGVMPVQRGKIGKITFSWTSLPMIYAYIFYMITTTIVLMVGYERVDILLNKSKKFDEYIYSIIFIIFLVPHFWIPFVGWGVAYEVCDYKNGWGTFQLHYYKIAGKNLEFPHLSTLIVILSIGCLLLAVGFLLILSTLLDGFTLYHTTAYYHIITMINMNCALWYINCRAISNASEALAKNFQRDIDKYCTSYIVAHYRVLWLELSELLQAIGNAYSRTYSTYSLFMMTNVTIGIYGFTSEVLDHGITFSFKEMGLLVDSIYCMVLLYVFCDCSHQTSANIAEGVQLSLMNIKLNSVDVATTREVELFLKAIHLNPPKVSLQGYSVVNRELISSSVGTIAIYLIVLLQFKISLVNLRG

>AglaGR3INT

MYNRNLNHLSIIGDSVTEPARKRSVYLEGAQPFYHTSLSKITKVAPAPGDYANGAPNAFQKSALDSALFDTLKPVLTLMRVMGIFPITNQGQIFQVTPQWMIYSIVICFLILGYIGYIRWDRVQMVRSAEGRFEEAVIDYLFTVYLIPIVMNPIAWYEARKQAAVLTNLVAFEKMYRKISKKKIYVFLGNRPLITAIGLPVLSVATMIVTHITMVHFRFLQVVPYCYINTITYLIGGAWYIYCDLIGKVATLIASDFQQALRNIGPSARVADYRSLWMLLSKIIRDVGNAFGYTVTFLCLYLFLVITLTIYGLMSQIQEGLGVKDIGLAITAGYAIALLFFICDEAHYASNCVRVQFQKKLLLVELNWMNDDAQQELIATMVTYLVVLLQFQISIPEDSSGGETTASGVTEKTTTSN

>AglaGR4

MKVIKKVKSEVSVNLELDKPLAFKHKGIHENMKFLISMAQFFGVMPLHNVRKDVQDVKFKWKSFGLFYSVYNASATFVSSIFWFAKFAIHGVVVDETAQMSFYVCTCFTSIQLIQIARHWSHILREWSFVEMSMRSYSYQVNLKKTFVILTSLFMGVGLVEHLFFILNSVYQAKHCTGFAEAPLKYYSGVSFPNFFTLIAYTHWAGAIIKFSNTISTFAWIFTDLLITLISIALTERFKQIVRRLENNKVMHEKFWKEIRQDYQRLYHLSKTVEKHMALLVLISYIHNIFFLCIQLYNSIRERTGIIESVYFFYSFGFLVLRLLVISMYGAWLNDEARKPLDILYAVPSEHYCTEINRLIEQIYTNPVGITGSGFFLVTKNFLLQMAGTIVTFELMIFQFAQPVKTTSTTSTNITYNCNVI

>AglaGR5PSE

MEKRNTENSHVLLKDILLVAQLCGLFPINNIRNPIPDGIRFKWVSWKILYCAVLWLLSLFCGLMALIKALSYGSVISQLIIPLYYCCTSLTIIAFIQIARNWAIFIQMLCKIEEELLIKYNNAINLKGKLRKTAIALYLFGICEQCLATLNFIFSYKCESNAHGWEQFFKKHFHHIFYFIPYHHVLGIFSLIICWLNLIIWNFGDIFIVYLSIIMAARFRQISDTLQLYLSTQKGNNAFFGEIREDYNKLALIIKKLNRLLSNLIVLCYAFNLYFILIQLFNSIREMEPYTERIYFFYSFGFVITRTIIVSIYAASVNEESKRFLPILNSAPSSFYCLEIQRLITQIHNDSSALTGHNFFTITRGSVLNIAAAVITYELVLIQFNQNTMEFYKSANSTICFDAVD

>AglaGR6

MVYVIPQENVFNVNIKPKYVKESRENILNSLKFFVICSQFLGILPVENITVPNKLRFRWKSWKVLYTLLFIGMTSFASTLCLLDWFYAGYVFNSLGITIFYSSALVTLILYVDLARSWPKLMTLWCRIDKIMNNSYGYPKSLNRRFYIVSITYSILALGHFLINTVYKSLSIKESTGNEYNFNKYYFKSFPQMFRFIPFSAVSATFCFIVHIHGFLTWLTNDLFIILISIALALRFKQISERLVRNQRTNKSLHFWKEIREDYDRLSLLCKELDKHLSYMILLSFSMNIFFLLVKLYNSLEGFADVSGQVYFVYSFMYLIIKIVSVSLYAAWINDESVGPASILNSVPASSYNIEIRRLLTQISFDNVALTGCRMFKVTRGIILSIAGAVVTYELVLIQFNAATGLNTTE

>AglaGR7

MVRGRKIHSTQTVLYPFLALAQCFAVFPVSGLKGNDFRTIRFSWRSRQSIYALLYLIFSLINLCFYLMKHLNSLESLSSFVGLIYFTTTLLIQIFFIDLARQWPCLMKKWAEVDSSMRSYAYSSNLYKKLKITSIFVMLAAFGEHGLFVANKIAVIETNVTKAEMIEIYFRRNYNQIFKIIPYNLWFGIFVEIFEVYSVFTWNYNDLFIMLISITLSERYSQIGNKIKLMSKYKIIDRSHWQSVREDYIRLNALCKLTNHLMTNIIVLSFMHNLFVVLIQFMYTLHYESNQMFIRRVYFIYSFGFLIIRTICVCLFAAEIDCESRNPLDVLTSLPSKIYNIEIERFIVHIAKDPAHLSGGGFFIISRSLLLRIAGAIVSYELVLIQMYTIK

>AglaGR8

MWSVFYKMKRQNKIAAAISRNTAQKNMSFHVSIRNILLTAQILGYMPVKGIYYPQISGVTFSWLSRRMLYSLLTLAGAIFLTTIQLKKSFYVKIKMPDIHKLCLYLMEVCSSILFIKLAREWPQFIKTWSRMENSMALYGWPPKLNKKINSIIVVFIVLLLIEYGLMQAVEFSWVNYCEGYLNKNNFCEVYLTKNFFSHSFFSYNIISTIFILYANFQAFFSCTIVDVIIMVLSISLAARLKQIKKRIKTVISLQSTEENIWREIREDYDRLEHLCKSVNSTFSWFIVLSFVGNLSLILIQLFITLQLNSSLEKICLYYSFGYLIARTACVCIYGAAVNDECRKALVHLYNLPDSIYNREIERMIHQISSNNMALTGLNFFVVKRDLTLKIAGALVAYELIIIQIFGDLLQLDYINGSLCISPHK

>AglaGR9

MKNKSSLKSIKSIKVNMDTKVILESQILSSSFYVAMRFPLQLAQCFGLFPINVGGTVPKSNCLKIKWFHWRMVYCLLTFGFAFFDFLAQLFQLIKSTNQSLGIIFSIDCVTKYILFFKLTLDWPNYLKEWQNIEDNMKKYELCSHLQKSLCRITVILMCLAAVEHAAINGFLLKDCIDKEPTVLKAFRKYFVKENYSAVFELVEYSLLTGIIFEFINLQMTFIWNYGDIFIILIGTALQCKLNQITKRLQSICRLKVRDMQVWNVVRKDYLELSKLCHSTNKKISLLIIVSFSSNLYFLLKQMFGSLRPMESTLQKFYFFISLGLLLLRVICVCIFGSSLHVEWENISYVLQSVHSSAYNIEVHRFVENVSTSELVLTGKNFFKITRGLILKIAGAIVTYELVVIQYNMETLKSKPY

>AglaGR10CTE

MNNIAMARRSSNTFYVAIRFPLQLGQWLGIFPIYVGGPTYTFLQIKWFHWRMLYSLVTFLLTLITLIAELLLLMKGTKKKLTILFYLNTVVELLLFFKLTLEWPDYMKEWYKMEKSMKGYEVSGKLRKTLITITLSTIIIGLVEHFLIKGYFFQLCLRVEPTFSEAIRTYFTEKIHDAVFQLLQYSPYMGIIFEISSIQSALLWTFTDVFIMLIGVSIKSKLEQITKRVCCLGSLK

>AglaGR11INT

MKKRRTEFDKPVEKIILGQRMASSTFHVAMKSSIQLAQWLSFFPIDVKVKTEKDLKFKWLHWRVFYSMFTFVLFVFEFVLQIYQHYMSSNEQTDMNYAAVIVFFLGAIVQYILFFKFTKEYPDFVKEWKRVEDRMRHYKIFCNLRVNLNCITASIMLIAAESSNNKFRTNYIFVTKKMSRVFVEMEVFLSIYSNKIIKMMTNVINFFIRLNNQIISVCHIKDERVWNSTRNDYLQLSNLCLSSNNKISLLIMVSFTSNLYFVVKQIYESLKPSGNTIRSLYFCISVGLVLLRNIGVCIFGSSLHVEWENLLSALQSTHSDAYNIEVDRFIENVTTCEMVLTVKNFFRLTRGLMLNIAGAVVTYELVVIQFNINSFQHSLHN

>AglaGR12INT

MKKRRTEFDKPVEKIILGQRMASSTFHVAMKSSIQLAQWLSFFPIDVKVKTEKDLKFKWLHWRVFYSMFTFVLFVFEFVLQIYQHYMSSNEQTDMNYAAVIVFFLGAIVQYILFFKFTKEYPDFVKEWKRVEDRMRHYKIFCNLRVNLNCITASIMLIAAFVNLQRTFNWNFADVFVMLISMAFKFRLKQMTNRVKALSRIQVMDVNLFRSVRRDYEKLSDLCSTINKRISLIVIVCFSLNMFFILLQLFNSLKKIDNTTEKVYFYMSFGLILLRATCLCILGGEMYEEWKNIAIYLFSIQSSAYNMEAERFITEVISNKLVLSGKNFFHVTRNLILQIAGATVTYELVLIQFYSESLLQVNY

>AglaGR13

MKYITKIKVLPKHQKGKEYVPQTFYQSCKDLLTFAQCLGLFPLSEIRQAEPKKLKSKWLSFRVLYSVVLFVLIFLCSATSIYKALNRDAVFYSLVSPLFNLHVACTLILFIRMSKKFPTFLSKIDALDKSFVKKYKSSTNVRRNVVVFAIIANIIVTVEHSLQALTFMLTKDCDNNSTGLEYYFKKHFHYIFDFFPYNTAFAFILLLANWIGAYIWNYGDVFIIAMSMILTARFHEIREKICIYTSAMPTSDKKFSKPLSNDLQKTNFMFWKEIRRDYIMMSELCRKLNDLISDVILFDFASNLSFILFQLFAGLSHKEKFAKILYFYFSFGYVVLRICLLSIFGGWLYEAGRASLPILNAVPTEIYNIEITRLIHQMHYNTPCFTGNNFFAVTKGLTLSVAAAVITYELVLIQFSPDIHADDHSTCL

>AglaGR14

MNRNLCVYLFGIVVYCCFALKMRLETIWKSKNQGMDLQDINISQENFELLQPIILVARFLGILPVRYDKHGGHFKLKMSLVYSIYSYVLTIFLTVATVLGIVNDLEKDTNHSVRMVDQKARYVTSCDISIVIIIVFFSAVTIPQKMRKLWKLLHYLNQTDSIIPLTKQSQFRQSSLFFMATTFVVAVLLFTFDIVVWTNSTTKRMKDATSFFRNYTTFYVLYLIVVIHEIFYWHLVLFIKIRISALNRYLHSIGKEDRRKKIPVQTGGRQVVVGGALENIEVCKESYKKKNFENMSLVERITVLATFQERISIAVQVLNNDGAFGIHLITLSCLLHLIVTPYFLLAEIIKPNGNVMFTYLQAAWLLAHIGRLLIIVEPCQLCLDEHRRTSMLLCELLTKDFDENVRNSLIIFSMQLNYCKIKFSPCGFFKIDRSLITSVTAAVTTYLVILFQFNTN

>AglaGR15

MLEKITTSTATYSVFSPILFLTRTCCFLPVRIRSLGHNTVISWSWRYYATSVGFFAGIMAWIGFGLSRSYSIQDVYDIRLYQMSAMFITILDDTYLLCTFTLATLISPLTFRHFCTYFRCLNKADYLLKRQVKNEKKKYSIVLLLILTLTVFILGLDMGMWWQHVFSKHINLGLYLLYNLPFYYTYFLVMVIEMNYWYLVQMVNVRLANVNENLTEEYNQYKSVRTKKKAAPMLRSEIGSTVSVLKEAPTDPFVRNISTTIKVYQLLYEACQAVNNFYGLNILLVVIGCFIHLFVTPYDLYLELKFKNYTFIFPQICWMISHILRLFLVVEPCHTAVQGVKGASYLVCQFLSLDLSEDLHLQLEILSTQLFQCPIQYTAYDLVILDRSFLTKITGGVTTYLVILFQFEN

>AglaGR16

MSVLISRDIYGSFSAIFRVSRVGGICPYVVLSSVSGFTLVWSKKMLACSYLSIGLLACCSVYGLVQNFKINEIHNIKSRTVAGLVCTFSEAASLLFSCILGTILTPFCRRHFVKYLKCLHQVDLLLVPRNPNQDKKCSRVTIIVLTITFVILTIDVTLWTSIIRKDTPLALFLLHTLPSYMTYICQTIVELHFWMLVHLVKIRLASLNEDLLALLQEVEICDSFLKNNDYRNKKVFSLISHFDDSRRKDMIKKENISKRVKNITHVYNLLVKAVTATNNYFGINVLVLLIGCLMHLLVTPYILHLQIKETKNYIFVGSQLMWLICHMSKLMFIIEPCQECKAQVQSAVFTICKLMHFEMCKETTTQLQSLLVQIERCKIQFTACNLVTIDRTLLSTVTGTVTTYLVILFQFR

>AglaOR1/Orco

MMKFKVSGLVADLMPNIRLIQASGHFMFNYHADNSGALHALRLGYSCAHLLFCLFQYGCIFGNLVVEKDDVNYLAANTITVLFFTHCITKFVYFALRSKLFYRTLGIWNQSNSHPLFVESNNRYHALALKKMRTLLICVTATTVLSAAAWTGITFVEESVHNIKDPDNENETITEEIPRLLIKSWYPWDAMSGMAYYGSLIFQIYYVLFSLAHANLMDSLFCSWLIFACEQLQHLKEIMKPLMELSASLDTYVPKSADLFRAPSAKSQDNYIENDYNAKNEELNLKGIYNTRQELGGNFRSGALQTFGQGGVGPNGLTKKQELMVRSAIKYWVERHKHVVRLVTAIGDAYGVALLLHMLTSTVMLTLLAYQATKINGVNTYAATTIGYLVYSLAQVFHFCIFGNRLIEESSSVMEAAYSCHWYDGSEEAKTFVQIVCQQCQKAMQISGAKFFTISLDLFASVLGAVVTYFMVLVQLK

>AglaOR2

MKQNYDFYGYFTSNIMIYKIVGFWRPDEDMKFKKLYNCYTAFCTLAWMAFLLSEIIYITNNRKNVQEITAALYVTGTFTIDFIQMIFTYKNMNHLKILMKEMNRTLFQVKCREHYRIAENTKRTYNVLFKSCLYLALLTAVFVMIVPLVGKERATSIKGWFPYDWTKPLYFALTYIFQNLVFIWNALICSNFATFTSALLMQVGLQCDLLCCTLDSLDDFYTEDDVLYEISLENKKKLRKDEERFSEAMTKNLVVCIEHHRQIMRVTKNIEQICGTSIFILFIGGTLILCSSLFQLSVVKVGSVESVMLLLYLICMIVDQLCYSWFGNEVICKSSLILQSAYKTPWVDCNIKFRKILLQFMTQTCQPISILTGGLFTMSVQVFVSIMRTAYSYFTLLKNIQ

>AglaOR3

MQQNYDFSSYFRPSIIMLKLFGFWRPDRNMKFKGIYNCYTCLCSSIWVAFLLSQIIYIINNRNDVQEITAALSVTVTFTVDLIVMMFTYKNMNCLKILIKEMNRPLFQVKCQKHYHIAKNTERMYKLMFKSCLYLATLTDVFVMVVPLMGKEKMSSVKGWFPYDYTKPLYFILSYIFQKLVFIWNTFISFNIGMIILALLIQVGLQCDLLCCTLDSLDDFYTEGNVLYEISLEDKLKLTKDREIFSKEMIKNLVICIKHHRQILRVVKDVERISGTGLFILFVGGGLILCSSLFPLSIVEIGSIEFIMLLFYLICMLVEQFCYCWFGNEIIFKSSLILQSAFNTPWIGCNVKFQKILLVFMMNTKPMSILTGGLFTMSVPVFVSILRTAYSYFTLLQNIQ

>AglaOR4

MQQNYDFSSYFRPSIIMLKIFGFWRPERNMKFKGIYNCYTALCSLIWMAFLLSQIIYIINNWNDVQEVTAALSVTVTFTIILIQMMFFYKNTNYLKILIKEMNRPLFQVKCQKHYHIAKNTERMYKLMFKSCLYLSVLTDVLVTVVPLMGKEKKSSIKGWFPYDYTKPLYFILTYIFQNLVFIWNTFVNLSIFMIILALLIQVGLQCDLLCCTLDSLDDFCTKDNVLYEIGLEDKLKLRKDRERFSEEMTKNLVICVELHRQVIRVIKDVERINGTGIFILFVGGGLILCSSLFSVSVVKIGSLEFIMLLFYLICMLIEQFFYCWFGNEIIFKSSLILQSAFNTPWVSCNVKFQKILLVFMLKTSKPISILTGGLFTMSVPVFVSILRTTYSYFTLLKNIQ

>AglaOR5INT

MQQNYDFSSYFRPSIIILKILGFWRPERNMKFKGIYNCYTALCSLIWVQFLLSQIIYIINNRNDVQEVTAVLSVTVTFTINLIQMMFFYKNTNYLKILIKEMNRPLFQVKCQKHYHIAKNTERMYKLMFKSCLYLAILTDALVTVVPLMGKEKKSSIKGWFPYDYTKPLYFILTYIFQKLVFIWNTFICLNTAMTIIGLLTQFGLQCDLLCCTLDSLDDFYTEGNVLYEISLEDKLKLTKDRERFSIEMTKNLVICVEHHRQIIRVVKDVERISGTGLFILFVGGGLILCSSLFPLSVSSLILQSAFNTPWIGCNVKFQKIMLLYMMKTSKPMSILTGGLFTMSVPVFVSTLRTAYSYFTLLQNIQ

>AglaOR6

MKKNKTFDYSQLMAHIFKIYKILGFWRPDPDMKHKNLYHCYTAFWLSLSITFMSSQVIYMYNNRKSLKEVLAALYITLTFVSILARQLTTYKAMNELKEIIKQLNRPLFQVKCQKHYEIAEETNRNQRLLYNICLFLGVSTDIFAALFPLFSKEKVILAKAWFPYDWTKPFNYFMTYIFQNAVLIWHTFVCYSIDIFTFILLVQIGIQCDILCYTLNHLDDFYFKDGILHEISLRDKLELRKDMEKFSKAMVKNLVVCVQHHREIIKLAKDIQRIKEIGIFVLFASGALVLCTCLFQLSMVQFGSVESMMLLFFSICMLTEQFLYCWFGSDVIYKGSLILQAAYNTPWTDCNSKFRKILLQLTTQACCPLNILAGGLFIMSVPVFISVLQTSYSYLTLLHSIQ

>AglaOR7

MKKNKTFDYTQLMAHTIKIYKFLGFWRPDPDMKHKNLYHCYTAFWLSLSLIFISSQVIYMYNSRKSLKEVLAALYITLTFVSILARQLTTYQSMNELKEIIKDLNRPLFQVKCQKHYEIADKTTRNQRLLYNICLFLGLSTDIFAAIFPLLANEKVILAKAWFPYDWTKPFNYFMTYIFQNAVLIWHTLVCYSGDMSTFTLLLHIGIQCDILCYTLNHLDDFYFKDGMLHEITPRDKLEFRKDMEKFSQAMAKNLVVCVRHHREIIKFAKDIQRIKEVDIFILFASRALILCTCLFQLSMVQFGSVESMMLLFFSMCMLTEQFVFCWFGSDIIYKGSLILQAAYNTPWTDCNNRFRKILLQFMTQACCPLNIPVGGLFVMSLPIFISVLQTAYSFLTLLHSIQ

>AglaOR8

MKETLNFKEIITLNVEVLNGFGYIAPEFQHKIEAVWFGIRSVVFIGFIYLGMLTSEIVNMILVSGDMEKMIEATFLTLTHLVEIRKVYAVIRYRDRLKKLLNSINRKEFLPKTTTQAKALQNYVQDSKVISKVFLGACVATCSFWGIYPFVDDGDLRLPLGGWFPFDTRYSPWFELAYVYQVIGSTVNGLVNVSLDTFMSGLIMVVCAQLNILNDSLKNMREQAETELKGVGIEVGQYMTNTLQEKMNEKLLNCVNHHRCIIEFANELTFLFTTSILGQFIVSVVIICITLFEITLLPALSIKFFSLILYQFCMLLEIFLLCYYGNEVIRESAELTKFAFCSDWMDCSPEFKRNLVFFMTRSQMALKLYAGGFFTLSLETFVKILKSSWSYFAVLNSVHTDD

>AglaOR9

MNFNFQLYFQIDFTRLKFFGVWINDGKKKPRLYPQYFCVINIFFIFLFNILQFIHLSYTSDSLRSIASSGYIIATCFMSNVKSFFMFRNRHRFEKLSSALNENVFQPKNNFQLLLTKKVLSLYSHVKTSLLVFCSVAVFSSMVNPLFHKNNDQDLVMPAWYPFDISTFLVHNVVYIHQCIAVFYTSYINIYVDIMLAGFATFIALQCDLLCDSLRKIPKEKYVLNKYVEHHWKILSFAKDTETLFSQIYFGQFINSTLAYCMTLFLLTLVDRTSFEFFYLVFYQTSMFCLLLVPCWFSSEITTKSKNIPIAAYECFWTDKPNYFKKDLLFFIHMSQEPIKLYAIGFFHISVEIFVKLLQSLIYSFSYL

>AglaOR10

MKRFQLKEYLHYNLVVMKYLGLWPKKDFTSDKSYLIYTIIVNGFFNFTMSVGLTGYILTSSNLLEDVIGAGYIILGIMATAKTFFIMKYSKMFKLLVDAEIHRSVNVELNEEQTKILCDYVGFWKKVHLIYSYLSVLVFFNYVTLPIFSKTPYTLPLNCWYPFDYKRPVVYEIVYFHQSVSALIDIFVNLNGATLAAGFLAYLSAECDVLVDLLKNLNNGSAMNEINLDTEMLKICVQYHKKIVEYSKLVESYFSKYLLIQFTSSCVSTALIMTSMSMNHDVNADFWFLSVLQLGVWADLYIYCWYGNEVTEKSKKIPYAAFESNWVPASKGYKKDLLFFICRTQKPIKLYAVNFFELSLSTFIGILRMAYSYYMLLSQLSKDE

>AglaOR11

MKKFQLKEYLHYNLVVMKYLGLWPKKDFTSDKSYLIYTIIVNGFFNFTTSVGLTGYILTSSNLLEDVIGAGYIILAIMATAKTFFIMKYSKMFKLLVDAEIHRSVNVELNEEQTKILCDYVGFWKKVHLIYSYLSVLVFFSYVALPIFSKTPYTLPLNCWYPFDYERPVVYEIVYFHQSVSALIDIFVNLNGATLAAGFLAYLSAECDVLVNLLKNLTNGSAMNEINLDMEMLKICVEYHKKIVEYSKLVESYFSKYLLIQFTSSCMSTALIMTSMSMNHDDNADIWFLSVSQLGVWADLYIYCWYGNEVTEKSKKIPYAAFESNWVTASKGYKKNLLIFICRTQTPIKLYAVDFFELSLNIFISILRTAYSYYMLLRQLSADE

>AglaOR12CTE

MPPRDLVQSSFKYHLLILKIFGLYPYDSFPKLYKPYAFFFYIAFTVITPILALVAIIVSEDHDIAAISQKGFMIVELNAMIVKLLPCKINPEGTRRTVFALNKKIFNSQLPEQDHILSEAVRNIKYVLLIFSTTCTCAVMTWASLPLMYNDRRFPFEVWLPFDPFQNTAVYLFLYLFVFLCTMNGGVDNAVLDTMVASLIYHAACQIRVLKDTLLHLDRRIEDQISKEGKSLSTEEREQLKNKVIYKKICDCIDHYDAIYEFVQDLERTYTVVVFSQLLSSVIIXXXXXXXXXXAVPLTIPFFSTSSFTVAILMEQFLYCYSGALLNEE

>AglaOR13

MSSEDLVQRTFKYNIAIMKIFGLYPFDSWPEIYKLYSFIFYLVFTFITPALIVVLLVVRAEESIQIIRQDGFMMVELITLTMKMLPCKVNPEGTRRTMYALKQRIFNSQLPEQDWILAETVDNCRFVFLTYCTSCVFTVLCWACVPLVYEVRRFPITVWLPFDPFENTPIYFSLYIFLVLVVVNAGVDNICVDTLLAFQVYHAASQIKILKDTLAHLGERAEEQILKEGKSLSLEDKDNLKNNIIYKKICHCVDHYEAIYKFVEDLETTYSFIVFSQMIATIVVICICCLRFTVDIPFTMPFFGTATFTAAALIEIFLYCYSGTLLYEESNSVINAIYMSEWYTYDEKSKKALLTLMERAKRPIKVTAGKLLDLSLATFTAIIRRSYSLLAVLKNY

>AglaOR14

MFSRDLTESTFKYNIAIMKIFGLYPFDNWPKVSILYGFISYVVLTFITAVLVVVLLIVRIKDSVQILSEDGFIMVELIVLSVKILPCKLNIKGIKRTMHALKQEIFNSQLPEQDRILAETIDNCIFIFLTFCTCSVITVSLWACVPLAYEARRLPIAIWLPFDPFEDTAIYISLYIFLIYVVVNGGVENVCIDTLLAFQVYHAASQIKILKDTLAHLGERAEEQILKEDKSLSLEDKDNLKNNIIYKKICHCVDHYEAIYRFVEDLETTYSFIVFSQMIATIILICVCCLRFTVDIPFTMPFFGTATFTAAALIEIFLYCYSGMLLYEESNSIINDIYMSEWYTYDEKSKKALLTLMERAKRPIKVTAGKLLDLSLATFATIIRRSYSLLAVLKNY

>AglaOR15CTE

MPSNDLVQRSFKYHLIIMKIFGLYPFDSWPQYFTPYALFLYVIFTIATPILAVIHLIVGEKPIVDVITENGFMIVELIALIAKFLPFKMYPERTKKAFSALNKEIFNNHLPEQEAVLDETVENCRFIFRIFCMSCAFAVLSWASLPLMYEDRRFPIDVWLPFEPFENTAVYLSVYLFVCLSGVHAGFDNATVDSIVALLIYNASSQVIILKDTLMYLSKRTEDEISKENRSLSTEEKENLKSNIIYKKICHCVDHYNAIYQFVEDLEDIFSMVVFSQLIASIIIICICCLQLSVAVPFTIPFFGAVSFLTAALLELFLYCYSGTLLFEESGTIVTAIYMSNWYNYDKKSKKALLTMMERAKRPMMVTAGKLMNFSLETFST

>AglaOR16

MVLYEIVNAIEVPKNMLIVSGVWPHDGPTFWYRLRKLMSYGGTMLLLALMFLEVVVSISNILNLSYVIPLSFEYFSYAVRLMVFRLEKVNVRRLSERLKDPLLNSYGEEHNEFVKSAIARTNRIATVFKVNCLLTTSFLALTPFIADAMFPIPFSFFQEGSMWYVVYLVQVNGLAVGIWNNAGLDTLNIGYMGVALAELDILIYNIEHSAEASASGVTKSKSDVDEYLFTCYKHHNKIMEFIKNIEAVCTKSILVHYVSSIVVICNSGFHLIMTPLFSGQFIILIVFFSAIMIELAMYCWIGNEIILKSLQIGDACYMSKWYEFSPRTNKILFLIMERSKRPLTISAYKFSVLSMSAYLKIIQCSYSYFTVLRRVYMKD

>AglaOR17

MLKNTSNAIKIQKFILTLTGLWPEEHPTLYGKISGRISVVTAIIFTATLIAEAIKQIGNYVVLIEHLSLIISPTSFLIKLIMFLRKTGQFVRLYRNLDMDIFNKHPDQFNTIKRKSETTSAVIGLSYMFSCFVITFFFCARPLYTSANMPVRFSFEMGQYKPIVAVFQIFCMFNAALSNSCLDVIAMTLMGIASVQIDILNRNITNFKKECDESATGTDGYIRYLNHCVKHHNEIIRYIGDIEEVFSLVFLAQYLTSGALICNIGFLLVHIRGLNLQFFNTVFYFAAMMCQLGMYCWFGNEIIVKSSDTKTACYESDWIDCEVKVRKILIIIMERSKRPLFLTAGKFSVLSLNSFTTVVNSSYTYFALMQKLYSKTNN

>AglaOR18NTE

HADLLGFLNTEMVALKIAGFYPLRGNKYKHLHTISATYFLSITILYIILACIHSFLNLTNITELSETVTFLMTILAYLGKILNLFFYRKNLIKLEDMLQNPILTKLETEEEENILKRNFRYSRLFTNIFKAKSATAASVHAVYPLLGDYGSKNFLFLLWFPFDPKDYYIPVYFFEMTLLLSSTWFDITMDTLNILMMDLCATQFEILKKRLIRIGTSFTGDEAIDDKLRLRKLRKYIIHHNYIYSCSELVRDTYSIGEFCQVGCSVMVVCFGLFKLLIIPLKSAQFLMLVTYSTTMIYQISLYCCYGQKLLNASDTVTEACYMSRWNDCSTQVQKYLAMIMNRANTPFLMKAGGIFSLTLETLMTIYTSAYSFFAILWKVYHSEDQAA

>AglaOR19

MKRHIDLLKFIKEERTCMVFGGFYSIEKYKVLHNLSAILIMTVVSLYNLLGLVHGFQHISNVAVFSQSIAYLLTGISFSCKMINLIMHKNNLLLLDEILQNPIFTELETTEEEVVLKNTLKFGQTLKKTYKLYTSATVTVQVLYPMINNPGHKNFPLLFWFPFNPEDHYYKVYFAEILMIFCICTFNVTVDLLNVLFMDLCAAQFELLKYRLKHFGREFHGGEAVNDRTLYEKLNKIIVHQNLVYRFSKLTEETFSAGVFCHLACTVIVLCCAIFKAVITPINSMQFLMMATYSFCMTLEVSLYCCYGQKVLDSSSTITEACFMANWYNCNVKVQEDLVIIMNRANKFVTMKAGGMFPLTLETLMRIWSSAYSFLTLLMQIYNENY

>AglaOR20NTE

HIDLLDYIKAEKKTLAFIGYYPIKEKMYKTLHTLSAIFFGFFPMMYSVLGIMYGFQHMSNLEEVSELIGYLLTGMSIVVKMMNLIWYKKNILELDDMLQNPILTKLESEEEEILLKNKLKLGQILKKAFKIFIAVTAILHTHILYLLVINIDPNAITLLLWFPFNIDDYLYEVYISEFFLVPSIAVLDAILDILNIIFIDLCYVQFELLKHRLKHFGRTSHEEEAVDDTVVYDKLGKIIIHQNYAYRFSEIVEETFSVSIFCQLAVTVAVLCCAICKVVITPISMQFLIRTFYTFTIVLEIAAYCCYGQKVLDA??TIAEACFMSNWYNCSVKVQKNMVMVLNRANRPVTMKAGGMFPLTLETLMSIWRSAYSFLTFLMQVYKEDN

>AglaOR21

MQKFDLLASFDTEKYHLTLGGFYPSTHIKYKYLYVLSCIFNLFISWSQFLSMATFSYFNSSNLEKLSEILLFCMTQFAFLNKLTNFVAQKTSLMKLEVMLQSYLLTNVTQKEIDVLENHIKEGRLLAKVYRVLCFLVVLFYALFPFLDDRSGESHKFPLPCWFPFDEIKYYYQVFFMEILSIAVGAWINSNIDILTVMMCVLATAEFEVLRNRLATILQPLSTSASIAEGDALVKTKLRDCVNQYDELLCLVNQIEITFSKGIFVQFFCSVIVICLTGFQMIVISFNSMQFFLLIVYFSCMMCQVAMYCWYGHTVMESSDKIRDACYLADWNESDLIVQKSLLMIMERAKRPAILRAGNFFELNIPTLMKILRSSYSYFAVLQRLYGKK

>AglaOR22PSE

KFDLLASFDTEKYHLTLGGFYLGTHIKYKHVYVLSCIFNLFISWSQFLSMATFSYFNSSNLEKLSEILLFCMTQFAFLNKLTNFVAQKTNLMQLEVMLLSYLLTNVTQKEIDVLENHIKEGRLLVKIYRALCFLVVLFYALFPFLDDRSGESHKFPLPCWFPFDETKYYYQVFFLEILSIAVGTWINSNIDLLTVMMCVLATAEFEVLRNRLATILQPLSTSASVDGDAVVKIKLRDCVSYFGKPICWILVNQIEVTFSKGIFVQFFCSVIVICLTGFQMIISFSSMQFLLLIVYFSCMMCQVAMYCWYGHTVMESKSLVMIMERAKRPAILRAGTFFELNIPTLMKILR*TYSYFAVLQRLYPLNI

>AglaOR23PSE

EKLSEILLFCMTQFAFLNKLTNFVAQKTNLMKEVMLQSYLLTNVTQKEIDVLENHMKEGRLLAKVYRALYFLVVLFYALFPFLDNRSGESHKFPLTCWFPFDETKYYYQVFFLEILSIAVGAWINFNSDKIRDACYLAEWNGSDLTVQKSLVMIMEMAKRPAILRAGNFFELNIPTLMKILRSSYSYFVVLQRLY

>AglaOR24CTE

MENFNLLDFFATEKLYLTVAGFYPAELSLSRYLYILSALVNLSTSWLQFLSLITFSYFSLNDLRKLSEILLFCMTQFAFLNKLTNLIWHKSSLKELEAMLQKTVFTSVRIEEKHILTNHLQGGKLLAKMYRVLCFLVVLFYALFPFLDERSDKGHTFPLPCWFPFNEGDYYYPVFFFEVWSIAISATVNSSIDVLTIMTMILATAEFKILNRKLTNIASYSGVDGDYDDDVEVRSRLGECIIHYDEALNLVRHIELTFSKGIFVQFFCSVMVICLTGFQMLV

>AglaOR25INT

MMETFNTMDFIRAEKLYLTLTGFYPAEPGLRRYLFILSALVNLSISLLQFLSLITFAYFNLNDLRKVTDILVFCVTQFAFLNKLTNLIWHQSSLKELEALLQKPVFTSVRSEEKHIKTSHLQGGKLLGKMYRGMCFLGVVLYTLFPLLDKRSDKEPKFPLPGWFPFNEVDYYYPVFFFEILSIAILSVINSSIDLLTIMTMILATAEFKILNRKLTNISTSSGADGDYDDDDEVRSRMGECIIHYDEALNLVKHIELTFSKGIFVQFFCSVMAICFTGFQIIVCYKTRWDKCSVEVRKSLMIVMERAKKLATIRAGNFFTLNIPTLMTILRSSYSYFALLQRVYGNN

>AglaOR26NC

YYYPVFFFEIWSIAISAVLNSSIDLLTIMTMVLATAEFEVLNRKLTNISTSSGVGGDYDDDDEVRSRLEECIIHYDEALNLVKHIELTFSKGIFVQFFCSVMAICLTGFQMLV

>AglaOR27CTE

TFNTLDFFAAEKLYLTLAGFYPAEPGLRRHLFILSALVNLSISWLQFLSLVTFSYFNLDDLRKLTDILVFCVTQFAFLNKLTNLIWHKSSLKELEALLQKPVFTSVRSEEKHILTSHLQGGKLLAKMYRVMCFLGVVLYALFPFLDERSDKEPKFPLPCWFPFNEGDYYYPVFFFEIWSIAISAVLNSSIDLLTIMTMILATAEFKILNRKLTNISMSSGVGGDYDDDDEVRSRLGECIIHYDEALNLVRHIELTFSKGIFVQFFCSVMAICLTGFQMLVISFSSMRFALLVVYLLVMMCQVAMYCWYGHNVMDSSDEVTSACYMSRWDKCSVEVRKSLMIVMERAKKPATIRAGNFFTLNIPTLMT

>AglaOR28

MSTIEDYDLRNAFKIERKLLLLCGIYPNEGRINKKLYNLSAFCHISFSLLITLSMVIFLAMNMKNILSVVEALLFLATQMAFLCKLFNVLNKKHKLLEIEDILANPAFYGYPKEKRHLIEDSVRFTKIFGMCYRSICTVVSITYAIFPLMDDDEWALPLSGWNPIQIDTKFKYWTIFTFQWVSYYMSVYINSGIDILIYILITVVTSQFEILKDNLTNIRYETDTAKRDFAKNVVLHYGILKLVRVIEDTFSYATFFQFFSSVVVICFTGFEMMIVPPNSIQFISMCTYFNAMIFQVAMYCWFGHSIIASSDKINDAIYMSNWYEADLSLKKSIMIFMEKCKKPVVLTAGKIFPLSLVTFTSIMRSSYSYLAVLQSMYGQE

>AglaOR29

MELKTPYFKKHLKWLLVLGVDIIPVKNVWYKYFYKLWSLFIIGFVVLYTLLEVIDILNTSDFNSMTFGLCYSATHLLGLAKIIILIVKKKKVREMLNELESGDFLPKIERGGEEEIRLINIAVTRCARHAEIFNLIVYSIVSIRCLYALFDTGYNDELFDEQLNTTTPIHTRILPYRIWLPIETTKSPIFEIVFFFQAFTLTLYGYYIGMMDSMVYGMMIHMNTQYLILKRVLERYVSIATNMVSKNLLDKGVKDIRNGVISLPVGYERIDFLSEPVQEKVREIVHNCAKHHVHILEFCEKVEKEFSYLMLSQFLFSLYTLCFQLYQLSLMANVLSFDFISMCCYLTLLMYQLFCYCFYGNEIMVQSEKFSEALYNSDWLVLDNSTKKSLLLMMMRAQRPIRFTAGKFALLSLQTFMAIVRGSASYFMVLRQMNR

>AglaOR30

MEEQYAEDFFVANRWILQCAGLWSPDGQHKVLRMLYKLYSIGIFLFVNIFFTATEFISLYYTYGNEYDLIKNISFALTHLMGAIKVVFFYFQKHKLLAIMDTLENKEFRYDSCTEKSFFPGLISRRYKNIGVKYTILFFILAHATLFSSYLPPTISTLMNSSRRGAEGQGLYVLPYYSWMPFKYNTDDSFLLALGYQAIPMFSYAYSIVGMDTLFMNILNSIGFNLEMIQGAFLTIRNRIIKQTGKSIYKSSILLDSEELKLKLCNEMKKICHHLQIIYKVCEDLENVHKYLTLAQMTATLFILCSCLYLVSSTPIASKQFYAEIVYMVAMGFQLTLYCWFGNEVTLKAAELPLYIWQCDWLTADNNFKASMILTMARARKPLYLTAGNFAPLTLSTFVSIIKGSYSFFTVLKSTNE

>AglaOR31CTE

MDSYAEDNFFHVRWILRCAGLWPPATKNKAVQALYRAYAVFIFLFVNVYFTATELVSLLYTYGNEDDLIKNLSFALTHAMGTVKAVFFYVKGDKLIELMKVLESQELRYEPCEERNFFPGLISRKTKAAMKKYTLICFALPHLTLFASYIPPAATAILVMFEGNGTRQLPERLPYYSWVPFGFDTGPSYLIALGYQAGPMFSYGYSISGMDALCYGLMLCIAGNLAIIQGAFLTIRERSLKRIKGPEWAADGLYNSACLNAAMNGEMRSICRNLQTVLNSCKDLEHLQKYVILAQVSTIQMIICSCLYLVSTVPINSKQFYAEIVYLVAMGIQPFFLCLFGNEVTFQAKYMPDYLWQCDWLAADKKFKKSMIITMARLSKPVSLTAGNFASLTLTTFVS

>AglaOR32

MLSEDISYPSNFFHVNEIILLISGLRLPNKHDSLLKKCFYYVYAIFLYGASITFFVLEILKLEDTVKDPTKFFSHIGLLLTHLVGILKACLLLFKYDEIQKVMDLLQDKGFRYETSDGFQPGLMVREAKRKSFKVSVLVFSLYTLAGVLAFASAWTSIIVKVKGQQFEVNITCYDFLPFYFHIPFPVETKSQCENVFTFMGIALCAFAWFIASEDAIFCALLNCLKTQLDVVGEALLTIRLRTLRKIDFPPGYAGLRDDDCPELERSLYSELNGCIKHLTSLLQARDELEGIFTYIILAQTICSILIISSCLFVAAMVPISSPTFFSQGEYFLCILLQLWLICNFGNEITFSSSEIIRSLYESDWFSCSLRYKKSMIMIMCRMQRPFYLSIGKFTPLTLITFMAVCRGSFSYFAVFRSM

>AglaOR33

MSGMNYPKDFFYVNKILQRVTGFWLPGKEIHLILRVLFYLYITLAYSVSIFFFICEIIIVSETVKDLNKFVRQFGMLFTHVVGILKFYILVFRRKSLQKIMDMLQDKDYVYEPQGTFQPGLMLSKAKRLTSFVSVMVFILYNFVGMSAQISSTITLNEVVKDDHLPGNLTCYDFLPYYFYIPFNAVTKFQCHVAFTFMNVSLDVFAWIIGTHDGFFVTLMNCLKTQLEIVQCAFRTIRERCEKRLDLPENYKIFVDESNPTLEKTLYDELTHCTKHLNILLQVRDDIENVFTYVTLAQTLASLIIFASCLYVASTVSMTSPEFFAQVEYFLCVLVQLSVICFFGNEITTASAQTGVSLYECDWFSSSLRFKRSMILTMSRIQRPVYVSIGKFSPLTLATLVAVCRGSFSYFTLFKSVQ

>AglaOR34PSE

FIHLTTFLRLNILAWIKCYLKHLYNK*LFYYYRDTKKIINNCYVTPKQQSAMHVHIIFFYKNVYFIFEIYSIKNLIFLRYLLNIILPLTSIQLKYNRCSNTVNFFFQILT

>AglaOR35

MKQHSSYPKNFFETNEILSYLTGLWLCKSNNISKWKKYLQIIWILIEYTVGTIFLILQFLILSETSKDINKFFSHIGLLFSNALGTVKIAFIILRRNKFKKFMDILHDENYQYESLDEFKPSLIFLKEKRFSKVISISVLCTFTLVGVSAHASAVRVIRKEINGTIFEGDYNCQDFVPYYFLIPFEVDTTTKCEYVLFFMDISVCAFAWLVGCYDGFIAALLNCVKTHLVILKGAIRTIRKRVMRRLDLDENLEIFHDEHLPLFEEELYKEFKHLVMHLNIILNVTEEIEKMFNLMILAQTLSSLFNIASCLFTAAKEPVGSVMFFATLTYFLSMLIEFALVCWFGSEITTASEELMFALYDIDWFSASHRFKKSLLLTMSRMQRPIYLSIGKFFPLTLKATVSVCKASFSYYTVFRRVED

>AglaOR36

MMEHSGYLNNFFKTNEILCKIMGLWLYDSNGESRWKRYLQICFASFEYTTAGIFLTLQFLILNETSKDINKFFSHIGLLLSNVLGAAKVAVMVLQYSRFKNIMDILHDKNYQYEYTKKFNPSLIFRKEKRFSQLCSISMYCIYAFVGLSAHVSVAATIRNKVNGTKFEGEYSCQGFVPYYFLIPFEVDTTTKCDYVLFFMDISVLFCGTLVGCYDGFLAALLNCVKTQLLILQDAIRSIRERVMQRLALDERLDIFHDETLPKLEEELYKEFKHLVKHLTIILRVADELESMYNLMILAQTLTSLFNVASCLFSASREPAGSAVFFATLVYFTSILIELGVVCWFGGEITTASEDIMFALYEVDWFSSSQRFKHSLVLTMCRMQRPIYLSIGKFFPLTLSAMVSVCKASFSYYTVFRRTDE

>AglaOR37

MEYDSNFLKLVKKVLIVIGIWPMNLSIYANLAYRIYHGMVLTYFTIFVVTLAIATKDILHVNPEKQAYVIQYGFSYALFLWKVILCQKESIRKLMKIISTKERLITNSNDNDIKNIYRICLQHNTTVFYIALLVIVAVVVLFAVTIYFEVKSWPAPEGISNCSVSARELPLPQAVPFGIDKYFKLVYIFQVSSRVFAAFIFLGCDTLLNYLIYFPAAYIKVLGHKFEHIGDQNGNRLYSSEAFLKKLILEHKEMIEYVIHLNDILQWLLSMDFFILSYHISLTLMGIMSLLESPVFNMDFMITLFFSVSYLMGVFFQMCLLYYNCNELIVESLEIPNSIFRSNWYELPQAVQRSLLIVIMRTQKPLEMKIGKLYAMSNDLIVGFVKAGFTYVLLSHLDFQQA

>AglaOR38

MEDNIHMKFAKILMIILGIWPVKLTGWKLILYNAYFYASYVYYIMYDISQGAIIFVARGTFLQTVGNLGVTIVYIINIYKVFICRSTSVKKILQEIEAKERLILENDDDTIKKIYYDHVQSSMLAMKYYVSLGSVGISLYFIAPIVRNLVEEDDHKYLIFTSWFPFDSDKHYAVAYLIQFFGGFYGYAYIVYCGSFFFCMLKYCVGQIKILQHIFRNLRQYTVKYSRNNDLDEKCSEEIFVKLCIREHQYIISLVKRLDDCIKTLMLLEFQISSFQLSLVVYQILQSSGWTIERTAVLSYLVTLCSQLFIFYWNAHEIIIESTELAQAVFENEWYTFDRNIQNLLIFVILRAQKPVGISIGPIYKVKMDALIGIFKAIYSYVAVIQK

>AglaOR39NTE

RTAVLSYLVTLCSQLFIFYWNAHEIIIESTEMAQAVFESEWYTFDRKVQNLLNFVILRAQKPVGISIGPIYKVKMDALIGIFKAIYSYVAVIQK

>AglaOR40

MGFLPQVLHFKVAMYFSVCIGLWPLITTKNKTVRKLYEIMSKLHYIYFNLFCFTQLVQLVVLLMEDQVNYTEVVNNLCILFIYFNSALRCKSIKGKAIRNVIKDVGKSEKKLWESMDENLKKIYNENVKRNNLFCKIFAGNTLLTNVGYAIWPLFMEKQTVEINNTTMEVKYLPISAWMPFDVQKHYVSAYLLLLFNCNYLCTMFFISTEALAFGLMTYPLGQIHMLNHILRNFRKYKDSLQRSMNLKEEAASETQLRECIVKHQDIIKYVDIFNAEMKHTYLLDFLQCSLQLTCVLYRFMTQRNLVLILINLQFILVMLIRLFITYWTANEVTLQSLAVSDELYSMEWLDETQKVKKMILFMIMRGQRPLILFIGIFKAMSLEVFVTIIKVTYSYISLFYRHN

>AglaOR41NI

DIFAFSLIIFGVGQIKILKLILSNFQEFAMNIKDQLHCSQEEASYITLRECILKHQEIIEYIKEYNLVMKNIMVLDFLLSSVELASGVLTLLVVLKATYSYITVIY

>AglaOR42

MIFPKNEHLKITIYISAVLGVWPFIFEDNPVLRKIYDVYSKCIFCYYLLYVLTAIIQLFIIVTDEVLDVDEIVANLCITLLNFVAILRVNAIKTERVKNIIQNVFKLEEKMMNSGNDEIIEIYNRHARQNQTCNKIFLVNVYLVAILYFIHPLYVEDTIKYYPNRNETVVIKALPLSTWYPFDQQKHYVLTYIWEQIDAFMATTFIASSDIFAFSLIIFGVGQIKILKLILSNFQEFAMNIKDQLHCSQEEASYITLRECILKHQEIIEYINEYNLIMKNIMVLDFLLSSVELASGVLTLLVTEMTLPNTIYSSQLAFSLFLRVLVYYWYANEIMVHGSEIGMALCNSNWYEESERVQKMMVIMLMRCNRELYLEIGPFAAMTLRTFLGVLKATY

>AglaOR43CTE

MIFPKNEHLKITMYISAVLGVWPFIFEDKPVLRKIYDVYSKCIFCYYLLYVLAAIIQLFIIVTDEVLDVDEIVANLCITLLNFVAILRVKAIKTERVKNIIQNVFKLEEKMMNSGNDEIIEIYNRHARQNQTCNKIFLVNLYLVDILYFIHPLYVEDTIKYYPNRNETVVIKALPLSTWYPFDQQKHYVLTYLWEEMDVFMATTFIGCSDIFAFSLIIFGVGQIKILKLILSNFQEFAMNIKDQLHCSQEEASYITLRECILKHQEIIEYINEYNLIMKNIMVLDFLLSSVELASGVLTLLVTEMTLPNTIYSSQLAFSLFLRVLVYYWYANEIMVHGSEIGMALCNSNWYEESERVQKMMVIMLMRCNRELYLEIGPFAAMTLRTFL

>AglaOR44

MIFPKNEHLKVTMYANALLGVWPYIFQDNPCLRKLYNVYSRFTFYYFILFIISAIMELFILVADDENRTEEIVANLCITLIYIITAVRVYVMRSTTIRNLIKQILATEDAILKNDDEEIMEIYKFHARQSQITNLIFIVNITVETIFFFTHPLYVDEKIKFNKATNETKVIKALPLSSWFPYDPQDHYLASYMWHIFDGTVGASYVMYTDAYNFSLIIFPLGQIRILTHVLSNFPRYVLKVKDQLQCSRDEASFITLRECILKHKEIMRYLQEYNDSMKNIMLLDFLQSSLQLASVVIQLFVTKATVFNIIFHGEFTICMLIRLLVYYWYANEIMLESLNVSTAVYECGWYDEPQNVKQMMLLVIQRANKALELDIGPFTTMTLRSFLGIIKATYSYLMVMYR

>AglaOR45

MIFPINDHLKITMYANSVLGVWPYIFQGNPKLRKLYNIYSKFMLSYFLFFLLTALIQLYILITDDELKVEEIGANLSITLLYPITVRRVLVIRSTKMKKLIKKILAAEEAILGGKDEEVLKIYKFHAQQSQFTNLLFIVGIAVQAVFYFTHPLSLDDVVKFNNVTNETTIIKALPMSSWLPYDPQKHYLISYLWHMFNGLIGASYVTVADAFSFSMIIFPLGQIRILLHVLTNFSEYVGKVMEQNQCDRDQASFLTLRECIIKHKEIISYLQDYNHSMKNIMVFDFLQSSLQLASVVIQLIVTTPTFFNVVFYGEFAICMVIRLLVYYWYANEIMLESINISTTVYECGWYDEPRKVKQMMVMVILRANKALGLDIGPFTTMTLNTFLGIIKTTYSYMMIMYR

>AglaOR46NTE

FPKSEHLKISIYTSSMAGIWPFVFQENSTERRLYDIYFRYLFYYFILFIFSAIVQFFIIIQDYETGVQQVMNNLCVTLPHSICAMRLWSIKGDTIQNLIKGILATEKGILESNDEGTIKIYKFHARKSYITILLYTSNLTMAAILIFIHSLHDENKIKFDKATNETKIIKSLPLSSWLPYDPQDHYLISYLWLTFDGMVGAFYMMYTDAYNFNLIIFPLGQIRILTHVLSNFPRYVLKVKDQLQCSRDEASFVTLRECILKHKGIIRYLEEYNDTMKNIMLLDFLQSSLQLASIMIQFVLTKATVFNIIFHAEFVFSMLLRLLVYYWYANEIMLESINVSTAIYECGWYDEPKKVKQMMLLMIQRANKVLKLDIGPFTTMTLGTFLSILKASYSYLTVMYR

>AglaOR47

MIFPKNEHLKISIYTSSIVGIWPFVFQENSSRRRLYDTYSKFLFYYFIIFILSAIIQLFILIQDDETEVQQIMANLCITLLHVITAMRVWSVKGETIKNLIKDILATEEDILNSNDEEVIEIYKFHVRQSQITNLLFIGNIMLETVCYLINSMYAEAVVKFDAATNETILVKALPLSSWFPYDTQEHYLMSFMFHVFDGSVGACYVMFTDAFFFSMIIFPLGQIRILQHVLSNFSEYVAKVKNQMQCTRDKASFLTLRECILKHKKIIGYLKDYNNAVKNIMLLDFLQSSLQLASLAVQLLLSKPTIFNIIIYGQFIICMLLRLTVYYWYANEIMVESIEISSAVYECEWYNEPHQVKQLMSLVILRANRPLGLDIGPFSTMTLNTFLGIIKTTYSYMTMMIVYR

>AglaOR48

MGVTFLHYLKHTLLITGIVPYDMLTCNKTRYKLFRNTSHVFYLFLLTFMYLEFPYIWNDSEKFIESLNLITTYTVLAIKMFICRNTNFGGLMCDMVAVEQQTVASRDDKVLNIYKRYVKRAKYIQLFNIFVYVASAVVYSAPYILKYLILYKIKEQVDGEERKLPFYMWMPFDKKNHYLLAFAINLYFLFITCIYYSTVPVVIIVLLLYTSFRLKVLGCLLKNIKIYMSKFKENDENITVEAVIRYCVLEHLKIMQITNTLNNCLRNVTLLEFIVLSAQIALIAFEGFTSRSANTVVVCIVHVLLLLVHMLLFYWHADEIRHESMAISEALYESDWYEYSRTTSSTIHIMMMRSQRPLSLSVGPFGEMSLTMALKILKGVYTYMTFLQHSYGQTSSLGTNKYPFR

>AglaOR49NTE

TGNISLLCYVKPIMIFLGALPYQLVDINKRFYTSYKYVLQSTFFLVLIFFYLETPNFHGPMEKLFQNVSILSTYTILYVKGLVCMSKNMSKLLLDMVYYESNIKASKSEEIFNIYLSAVKRSKIVQVFYIILTYLTGISFFQKALNYAMSSDEIKFNSTLEAHESLPYAMWLPLDEKEHYLAALCIQTLCALLAINYYCFVQCILVILPLSVTLRLKVLGNHLENIKYSRNNSLQTSEADDLDCIKDMVVLCIKEHTNIIEICSILNNTVKYLMLLEFLMSSAQISLALVLLTTTHETNEQLFALIYLFQLLTQLLILYWHGDEIREQSVAISNVLYGMQWYEYNKSINTSIHIMMIRSQKPLSITVGPFGEISLEMAVKIMKAAYTYVMFMKQVYE

>AglaOR50

MDSPFLHYVQYLMVVGGIFPVGLINSHKRIYILYRYVTHALLFLFLFCLFDGNKDFHGSKEMLFKYINDRILVIVIYINLVTSMSENFDELIFEMIAYEAELADVGTEKHLKINEQIVTKTRRVQVFYMSNILACYVALVVPAFVEYAVVQASEKSTNITINTYTATPYEFWVPFEESKHYLALLLIQAYYFALMGVSIYCPFQIILINLFLTVILRLKMLRANIESMNDIDTLDVNKFIRIYAKEHIDIMRNCRFLNDTMKYAMLLEFLITSGELSMFIFIVIMTNSPNSKMFSGACIVNLLVKILILCWHADQIREESIGISDAVYELPWFEYDKSDVTSLHIIMMRSQKPLTLTIGPFGTMTLELAVKIFKATYTYVTFMQNVYGKLD

>AglaOR51

MDSPFLLYIKHLMIIGGTLPVDLINSRKRIYIFYRYVTHTFVSLAGIYWLGGNMNIDGFDRTLFNYIYDRIFVMISYVNLAITTSGNFQKLIYEMIAYEAEVLDVGTEKHLKIHEQVVKKCRTLQVYYISILSSCSIAFVVPAFVEYALVESSEESTNSTINTHNYDLWIPFEESKQYLVWLLVQSCYTLIVTCIYCSYQTILINLLLVVILRLKILRARIENMKDIETLDAKRFIRIYAKEHIDLIRNCKHVDDTVKYVMLMEFLFASLRLALSIFLLVTTNTPNSKIYFGSVTVNVLINILILCWNADQIREESIGISDSIYQLPWFEYDKSDVISLHIMMIRSQTPLTLTTGPFGTVTLDLAGKILKATYTYATFMHQMYEN

>AglaOR52

MSQTIHLKLQKYIMLLIGTWNFKSYNNAFLVMYKAYSYFIFWHYVVMTQLVMVSIPIQWDCKSRVIELICFYIQYTNNIIMMVLSKYSNKMQKVFDHILDYEDIKLRSTTDVEQKIYFKYAKLNNRVSLFVTIVLLGTSVYWYTTSVRYSLFGQPTDVCPLTKGTIYQIWLPSIIRKYHWLMILNDIVFFMSVINITLYREIIMFAICIFMLGQIKILQQNVRDLEKNSEVVRKSRNVSYDDGLLISVVKCAQEHQQVTKLMEIVQSATSIFILVLYFSNTFEMAAYLFQLISEKSIYNILRPLYVFTLMISQLYIFYKYTNEILVESTALCDVIYNETNWIDYNQAVKKNLLLMMRRSQKQLSFKAAGLGDMSLQTFTNLIKLCYSIVACLKTAYDV

>AglaOR53NTE

VKHDAIYLNIHKYIMIIVGKWYVGFQNKALDKFYRLYSMFFETFVILITQQIFVSIIVYRSCPAERVIELICYYIQYTNMYVSSLLCRRASMRRVYEYIFDYEKTKLHKENDVSVNIYFKYTTLNRRMLVFFALLTNITGAIWYILVVRHTLTEHDTTLCALKRGLNFQIWYPFDMYNKCYFLTVLTDLMFYCSVVSIHIYNKLTPVTFMLFELGQIKILQNMIRCIDKNASELASFEDIDSDEAVLITMRGCVKKHQEIIRLLHMMDNACKKIVLIIFFSNSVELAAFIVKLLTETNTLDIFRTFGILIMLITQIFMFFWYANEIQVQSTAISDILYNNTDWINYNKPARMRMLLMMIRSQKPLTFNAAAIGDMSIDTFKRIMKLCYSITAFVKTVFNSRTEM

>AglaOR54

MGDDHTYLPFTIKILNMINMWPNEAKKCDFIKKYYILFGILLSITAIMADFILQFYDDDFSFTSLVDSLIGLSALLSVIYISVCFIVKDQEIKRLTENLKIFEEYIPKVKIEETEDKAKFHTKIFIIYGIMGNIIYDCLPLLTYEDCHKNRSKHMIKYGIPCGGIVHYVLPYKQDTFPLAEITIVNQMLISTVGTLIVMTVTMLVCGILIHVSANLKYLRSMISNLTYTPNNRIMQHVNSCVKYHTMICDTMMLVHITWTSFIISVLGFEIIMDTNYSNSIRFSLHLGGWLGMLFLICFYGQILMDDSFAVSEALYEAEWYEKPPIVRKSLVLILLRSQRPLVLKAAGVNVMSLATFLGVLYNAYSYFTLLLKIKP

>AglaOR55

MDILRVNIKTLKLFLLWPSDNMSWVTNIVLSVIHQTFIFLSSTPTFSAVGYQIAVGIDDINIIIESIIGCGDYLGFLFVYICFRVNVPKIKKTIKRSSVFLEYCGNSIVEEAEKKVRFYTLGLMIYFFIGLTINTILPLLDYNNCNERRLSDYYRLHDPCGMPTRVWVPFEINTPTLFYLLIFLHNDACVHISYTVLCITMILIGLLIHITAQIKNLRKHLIDIFNTEGSDSESDELIENTEEKLKFCIKYHNTIIGYTNEVFEAFNITLIVHVCLTSMVVAVIGYQIISADNLMDKLRYFLHLMGWIGMLFITCFYGQMILDESITIADAAYQSEWYNGPEHFKKMICLIIVRSQRPLLLRVASIGVVSLETFVSVIKTAYSYFALLLTIAK

>AglaOR56

MYKAEREEPFYTSLNYLSRMRQIPVSMKKSYMRKFITLFLMSKVVGLFCTAIGPILHLIISIMRNSGTNVTEDISIIFGASGGGIVSLLFTLKYKNWSNFFKDLVDTRQYTRPRNLQARATRLNHLGKICFFCAWWVTITYAIAAWFSSKGCQDTNDPGKFGEICPTFIPIWLPFGTNDVAAGTVVFVLQTFFVGCLIAQSFLAECFVMYESTEIFICHISELKSQLKETLDAPFSVETRDKLRLCVDYHVYILNLGYRLSSLIKYTAGHMSLLSALVCGCVANQMLNTKPMGSLFFLAAWLFALFCFCHSGQRIKDKTLSIADAIWNTKWYKADVKTLKDVQFILLRCQKPICFEALPMGVTDYAFYFMMIKTGYSFFALFRQSL

>AglaOR57CTE

MYKIEKNGAFSNSLNYLKMTYQIPVSMDKSYLKKFVAIYLLSKVVSFFFTTIGPLLFLIVIIVTHSETDISEAASIIIAGTAAFLVNVLFTLKYKHWSNIFKDLEDLKKFGKPASYQKTVNFLNRLGHISIFGSSMVIFIYGLVAWYNTKFNKEFKEDFNQISPVVVPIWLPFGANNLTAGTIIFTMQFIFSGILTCQTYLIQSFIIYESTEILICYISHLNDNFKETFGASSRVEMRNRLRFCIGYHVHILKLGYRLRSLVKYTVGHVSLLSALVCGCMANQMLTTKPIGSFILLIAWLIAVFSYCHAGQRIKDKSMSIGNALYESKWYNADNETMKDVQFILMRCQKPICYEAIPLGIIDYPFYFM

>AglaOR58

MYKIEKNGAFSYSLNYLKMTYQIPLSMDKSYLKKFVAIYLLSKVVSFFFTIIGPLLFLIVIIGTHSETDISEATSIALGGTAAFLVNVLFTLKYKQWSDFFKDLEDLKKFGKPASYQKTVNFLNRFGYISIFGSFMVIIIYGLTAWYNTKLIKEINEDFDQISPVLVPIWLPFGANNLTAGTIIFTMQIIFTGLLTGQPLLIQSFIIYESTEILICYINHLNDNFKKTFEASSRVEMRNRLRFCIGYHVHLLKLGYRLSSLVKYTVGHMSLLSALVCGCMANQMLTTKPIGSFTFLIAWLIAVFSYCHAGQRIKDKTISIGDALYESKWYNADNETMKDVQFILMRCRKPICYEAIPLGITDYPFYFMMIKTGYSYFTLLNQST

>AglaOR59

MYQVQRNKPFYWTIKALHIVMAIPVSFEKSYLRRFYIKNVVVRFGAFACLAILPSCHLISSIKEHNGADISEDISMILGGVGTVVTNTLLAFKYDLWSTFFMDLENCGHLFGEPSTLKKRKKELNIYSLTYTIYTYFGQFVYGLEAALKWSDCKKLNEEKGLKEICGAFFPLKLPLDVTSPLGSAAIFALQFSFTIFSIPSGGVICFMVLEVTEVVICYYKELKSNFERVFDVPDEEKRKERLRFCIRYHKHMLGLTRRLQKMVKYTAGHLSLISAIVFGTLGNQAFKSKPLGAMIFLVGYIIALFSLCRAGQRLMDETQAVSDAAYNSKWYDADPKIMKNIVLIIARAQEPEMLDALPLGSFCYPLFLLIVKTSYTYLTLLQQTT

>AglaOR60

MYEISKEKSFYSSLTILRYTYAIPSATEKSGLTWFRFIFIVIRLASLLNFVILPCLHLISTVRAKTGVDISEDLSTMFGAFGFITNAVTFLILYEKWSQFFIDVEDCNRFEIPEGIEERKKKLNLYSLLYALYAICGVLIYGVIASSETSYCKRMNEEHGLSETCGMFTPIVLPFDGSNVYIRSIIFGIQMLLGMFTLPSTALISFLLYEGVETLILHITQLKKSFIEVFDVASNAERKKRLRFCVYYHINILSLCQRLSTLGKYTSGFLCMACALVFGCIGNLILKSKPIAGISYLLEYVFALFLLCHGGQRLLDETLSVADVIYDTKWYDGDVQIMRDIRFILARSQIPVMIGALPLGCMNYALFLMIMKTSYSYLTLLTQNT

>AglaOR61NTE

NTGVDVSEDFSNMFGTFGANVVTISFLLLYDKWSQFFIDLENCNRFGIPTDIEALRKNLNLYSVLYALYTVVGVAIYGVIAIAETSHCKKFNEEHGSNEICGTFTPLVLPFDGNPIFVRSIIFVIQAFIAMTTFSAPAVVCFMLYEAVETLVAYITLLKQSFINVFETTSEAERKDKLRFCVYFHDNMLSLCQRLSTLGKHSAGFLCMTCAVVFGCIGNFILKSKPVAGICYFLGYVAALFLLCHGGQRILDETLSVADALYDSKWYDGDVQIMRDICFILARAQIPATLGALPLGVMNYPLFLMIMKTSYSYLTLLSQNT

>AglaOR62

MEAEDIVIMENSLRYLSRNLVFPRKKDVNNPGVSFHLKFVALNLSTVMFLTGNILHLMINIKRKTYINLDLDLALAISLLGSYYFNFSYIRQVKNTINIYKQLSDLRSYGIPKDFYATNKKLNNYSKYHYIYIVSAVLGLSVAPLLEYKKCRKENVVKNINEICGLIGSIWLPIDLDSTPYKQMYYIFQVYSSFVIYQTSSLISFSIMETVEHLILRLNHVKNVFLEALMEREHGVRREKFRRAVKYHVNIMRISKLVNTSFNMCMFVHVLLTGAILGCVGYRLLKSYSLGAICLFVGWIVSMIMVCISGQRLRDQSLSIGDAICRSNWLDLNKELQKDLVLVLLRCQKPIFLNAGPFGYMTYAMILTVLKTSYSYLTLLSSTS

>AglaOR63CTE

MDADDIVIMENSLRYLSRNLIFPRKKDVNKPGVIFHLKFIALNLSTVMFLTGNILHLTINIKRKTYINLDLDLALAISLLGSYYFNFSYIRQVKNTINIYKQLSDLRSYGIPKDFYATNKKLNNYSKCHYIYIVCAVLGLSVAPLLEYKKCQKENIVKNINEICGLIGSIWLPIDLDSIPYKQMYYIFQVYSSFVIYQTSSLISFSIMETAEHLILRLNHVKDGFLDALMEREHGVRREKFSRAVKYHVNIIRISKLLNTSVSTIMFGHVLLTGAILGCIEYRLLKSYSLGAICLFVGWLISMSMVCIGGQRLRDQSVSIGDAICRSNWFDVNKELQRDLVLVILRCQKPIFIDAGPFGYMTYAMILT

>AglaOR64

MEEENTQVLENTLKYLRFFLIWPQEGTNFNLGKAFYLKFVLLTISTSFILYGSVAHMIVTLYEKNGAQIDLDIAVTISVIGSYYFNFSYLSNIKEIADIFKDLANFKTFEMPSGYKHFLKKWNELSTFHFIYYVILCSFCLVVALLESRRCLAKNALKGTHEICGLALNLWLPFDIDYFPLKQIICCYQIYSVFFSYVLSSTVSFSIMESMEHVIFRLDYVKQLFVEALRQEEQVIRKHIFKHSVQYHNFVIRLAMRFNNCYSPCIFIHVLLTSVILGCVGFHLTQSPHLLTIGIFVGWYMALIMVASGGQRLIDEATSIGDAIYSTRWYDAENEIQKNVVLVLVRCQKPIVLRAGYFSFMSFQMIVTILKTSYSIITVMKGIDLN

>AglaOR65

MVTSIVYFATTLKIFRIYQVFPAEGHELKPGKMFYIKFGFTCLFASAIITGSFMHFVYSLKSRNNIYIYKDICYVLSMLTGYSFFVLFSFNIKSAAKLFILLSDFNKFGKPPKFDERNEQLNRFSRYHYIYINLASFAFLSVTNIFKSHQCIKENQEYNFNEVCGLITNAWLPFDIDYFPLKQIYATLQVFSIFYVYVTAGTVTWLVVEVVEHISVRIDHIKHLFTSALKEKDPEEMKRNFRFAVQYHSWFLELEDELNKSFSIPMFSMMLLGAPILGCAAFGYMETGANSLLVICLSWFNALALVSFGGQRLINENLTIAEEIYNSKWVDVAPILGKDIIIILTRCQKPMKLRAAAFGVMNHAMIVSVSRAAYSYLNILRASSK

>AglaOR66

MSKPQIVCLQNTLKIFRFYAMFPEKDKELNPGKYHYVKFAILACFSSVVLIGSFLHLIKSVNANNYLSLDTDLIYTLSLGITFAFSIGFLYKTEALVEFFLFLSDFEEFGKPLDFDRDNTFYDRLSIYHYIYLETLVSGIVIISTYLNMNKCQKLNLEYGLDEICGLFSYTWMPFEIDYFPMRQIFLVVQLFGAHHFYMIAGMCAWFVFETVQHFRIRIRHVNFLFQEAIKEEDPQRCREKFNHAARYHASLLGLEDKMNGAFGTFMFTHMVITAPIIGVGVFAIVSGGSVSSFLVCLGWFGGLAMACFGGQWLQDECFAVGTDLYEADWLHCPEDIKKDIMIVIQRSWTPMYLRASSFGIMNYRMFLGVLKASYSYITLLTQT

>AglaOR67PSE

MAKLQIVCLQNTLKMLRFYTMFPEKGKELNLGKYQYVKFAVLAFFFVSVILIGSFLHLIKCVSVAFSLSFLHKTEALVELFLFLSDFEEFEKPVDFDRDNTFYDRLSVYHYIYLEILVSGIVIISTYLNMNKYQKLNLEYSLEEICLFSYTWMPFEIDYFLMRQIFLVVQLFGAHFYMIAGMCAWFLFQEAINEENPQSCMEKLKYTIKTRIRKKLEDIINGAFGTVMFSHIATTALVIGIGVFAIVSSREAILVHFWYVWAGLVDCFVGQWLQDECFAVGTDLYEADWLHCPEDIKKDIMVVIQRSWTPIYLRGSSFGIMDYRMFLGVLKASYSYVNLLTQA

>AglaOR68

MAKPQIVCLQNTLKIFRFYAMFPEKDKELNHGKNHYVKFAVLACFSSVVLIGSFLHLIKSMSDNNYLNLDTDLMYTLSLGITFAFSIGFLYKTEALVEIFLFLSDFEEFGKPVDFDRDNTFYDRLSVYHYIYLETLVSGIVIISTYLNMTKCQKLNLEYGLDEICGLFSYTWMPFEIDYFPMRQILLVVQMFGAHQFYMIAGMCAWFVFETFQHFRIRIRHVNFLFQEAIKEEDPQRCREKFHHAARYHALLLGLGDRMNGAFGIFMFTHMAITAPIIGIGVFAIVSGGSVSSFLLCLGWFDGLAMVCFAGQWLQDECIAVGIDLYDADWLHCPEDIKKDMMVVIQRSWTPICLRASSFGIMDYRMFLGVLKASYSYITLLKQT

>AglaOR69

MWQNLSKFAPQNFYTWMDFFFKILGLQPPKDKKGKLLYLLVAVPHISMSAFLVTGLEWSKLFRDFDKDFKVSMLTLSVCTLHSVFIFRIIVWFFTREKLAGVARVVQRDSFEFNCFSIYKIKNVIERFRREKMVHTRAICCLIYLSINFISLFTINVSYINVLSTETYEGFNERYNTTSTYRVYPYHMYFPFDTSLSHGYYWVGYFYQLYAYCGIIITFLPVDTIVINSIIHVISQTTLVGEAFNQLGSNIDYSQSLDEVLMLHEIRIVKCITELQNIYRVLYLLEDLYNVQLLIQYGASVFILCSLCYIIPLVDNVGELICSFIYLSATLGEIFVFTYCSQTLSLKLQNVKNAVYNLDWPCYPPRLRRNLVFLISKLQKPCLLTAGKIFTLDLLFFIQVTQKSYSFYTLISNTGNNK

>AglaOR70

MNFKALLFPENYFRWLLGFFKLFGLYPPKQHKYKIVYYIFSLIHVTLTMISIILELLNVLLSQDTLQRRMLNLGVTFMHITAGFRVLKWIWTKNRFEEVISIIQRPSFEFTVYIYNGGSKTTKPNRTPIETFKDARQRRTNFICCGFFWIAFFFLFANIFVTYIYNLFNATYEKFNPHAGKISVYRDFPYPMWFPFNVSLSDGYYLVAFLYQPYAYFSLMASLWFVDWSVLSSIIQLQNQLTVVSFALKEIDRNVPRVGAKKISLIKKIRLTKCVEELDAILRATRLMEELCNIDLLVQNGLAAFFSCTLLHVLSTLTVPIEIFSISTATAVILLESFVQCWICQNLTVEFSKILEKIYELDWADYPPDLRKFLVIIMVRLQNPTVITLGKWKAIDVSFYIHLLKMTYSFYTLISRQANKNK

>AglaOR71

MFKGISKKVLNTSDSIKICVWFPKLLLNSVCMWPGQINDILQGFAFWSIMAVCTFVEYGLYNFIANNFENFNESFTAISFFSTTLQVIVKGSVFYYYLNTAKRISDVILYKFWPSNLLGKEVESSLKRYYQILLIGMMSIYFNGSFFSIVFILTPLFKETRQTPYKTVYPFEYSDGPKFEIMYLVQSFTNFYVVLGVVIGVDTLFMAVCCNITAQYRLLKNAFLKLGTDEVKELNSKLSTLCAESDTSERNTTEEKKFLIRCIKHHQTIEDVETVYSVIGFFQLGFSIVSICLSSFVLTTKNLEFSQLVNITIFLSGNIVQLFCYCSVATEVNFEMDNLSQYIFSSYWYQADFVNFKRDILFVIKKSQEVQKITALKLFPLNYNTFIQVLRVSFSFYTLLSNITVK

>AglaOR72

MFEGIPKRILNTSDCAKICLWFPKLLLNLVWLWPDQMNDILQGFAFWSVMGICTFTEYGLYSFISNNFKNFNESFTAITSFSTLMQVIVKESIFYYYLKTAKRILDVILYKFWPSNLVGREVESSLNRYYHILLIGMISIYFHGSIFSIIYILIPLFKETRETPYKTVYPFEYSSSPKFEIMYLVQSFTNFYVVLGVVIGVDTLFMAACYNIIAQFRLLKGVVLKLGTDEVKEINSKLIILSSESDNIGRNVTEEKEFLIRCIKHHQTTEDIETVYSVIGLFQLGFSIIAICMSSFVVTTEEIEYVQLVNISIFISGHIVQLFCYCSVANEIGLEMDNLSQHIFSSYWYQTDFVNIKQDILVIMKKSQEVKRITALKLVPLNYDTFIQVLRISFSFHTLLSNITVK

>AglaOR73

MYFTLLNFSIIILNAMGVNPRKGYTIPQVLLYGTTLSFQMSIIYLGMLLLVYNKSITIIDITYALETVLIMCHGITKCTLLFVKRNKVRDLLDRIGHFWKIEDIQDEEEKKEHMKYLKFMKTVFLLYTILCIWTTITFTCRPLYINGEITFNTYRPEWIPFYVLQVYESIAFIVGIYLPLPGLDKFILTLLLLTKLQFKLLNQEIEKVFNEMASSEGKWKHFDKKIKKIVEYHDFLLDFVNRINDVFSEGLLVYVIVIVLSMCVQMYIVSTQHSLMELIKPLFYLTNALVQYIFCYCLPAQALSDEADRTTEYAYFSRWYENPSSAKVAQLMILGRSQKKTHILAGGIVKIDLETCLKTIRTMVSYAMFIRTMGIGQDQTR

>AglaOR74

MYFTLFNICIILFNRMGVNPMKGYTIPQVLMYVTTLSLQMSIIYLGMLLLVYKESVTITDVTYVLDSFMLISHGLTKCTLVFVKRNNIRDLLDRIDKFWKIEDVQDEAERKEHEKYLKFIKTMSFLYNFLCICTTLTFSCKPLSGELSFNTYRPERIPFHLLQVYESIAFVVGIYLPLTGLDIFVLTLLLLTKMQFKLLNQEMERLFNEMASSDGKSKHFDKKIKKLVEYHDFLLDFVDRINKGFSEGLLVYVIVLVVGMSVQMYDVSTEPTLIGLIEPIARLITGLTQFTFCYCLPAQAVTDEADRTTEYAYFNKWYDNPSSAKAAQLMILGRSQRKTHILAGGIVNIDLDNCLKTIKTMVSYAMFIRTMGIGQD

>AglaOR75PSE

MYFTLFNICIVMFNGMGVNPRKGYTIPQVLVYVFTVNCHMIIFYLSMWLLVYNKSATIIDVTNTLKTFFFFLNRINNVFSEGLFVYVIIIVLSMCVQMYIVSTHMTNGLAQYIFCCCADRTTQ*AYFSRWYENSSSAKVAQLMILGRSQRKTHILGGGIVNIDLDNCLKTIRTMVSYAIFIRTMGIRQDQTR*

>AglaOR76

MYLSLFNICLAMLEYVGTNPKKGYSIKQISLFIITFVSQWSIICLVIMYLIYDEKQITILDVTNALESLLLTFHAIVKLTFAFVKRRELHDLLERTKFFWAVENTEDKQQKNEHLKYLKFLKMAVLMYNALCLTTTLAFTCKPFFVKGGYLTFTTYKPDWIPFYVMVFYEGLVFIFGLYFAIAGFDIFIVVVLMLLKMQFKLLNQEIRRAFQEIGKPGKNLKNIDAQIKKLVDYHSFLLDYVNRINSAFSEGMMAYIITIVLSMCVEMYIVSTQNSLTTSFKAVVYMINGLSQYILCYCLPAQAMCDEAERTIDYVYFGKWYKHPVSSVKVAQIMMIRRAQKKTLILAGGFMKVDLETSLITVKTMVSYAMFIRTMGIREN

>AglaOR77

MYLSLLYIPLTMCRFMGVYPEEKYRTGQIFIFVIIFFWQWSIIYLAILHLIYKDHITISDLTTVLETLFLIFQAMMKLTMFFVKERQFYDLLERINYFWKVDDVQDEVEKKKHLNYLKLIKNRSAVYNIWASATTVAFLLKPLFVKGDNLIFTTATPTWIPFGVMPFYEEVLFVFGVHGPIVGMDLFTLALFLLVKLQFDMLNQEIQRMFQNMKESEINQRIKKIVDHHNFILDYVNRINNTLSEGMLLYVVNVLLAMCVEMYIASVQKSIMAAIKAIMYASTGVLQYCICYCLPAQAVTDEAELTSNYVYFNNWDNHPVSSVKVAQTMMLARAQQQTLILAGGFIKFDLETYLKTLKTMVSYAMFIRTMGIGQD

>AglaOR78

MYLSRLHIPLTMCRYMGVYPEEKYRTGQIIIFLIIFISQWIIIYLAILHLIYKDHITISDLTNALETLFLIFHAMIKLSMFFVKESEFHDLLERINYFWKVNDVEDEVERKKHQKYLKLIKIRSSMYNFWASATSVAFMLKPFLLKGDNSIFTTASPTWIPSGVMTFYEEVFFAFGVYGPIAGMDLFTLALLLLTRMQFDMLNQEIQRVFQNMTNSEENVEETNERIKKIVDYHNFLLDYVNRINNALSEGMFLYIVTILLSMCVEMYIASVQKSVMVAIQAIMYASNGLLQYCICYCLPAQSVTDEAELTAKYVYFNNWNEHPLSSVKVAQIMIIARAQQRTLILAGGFIKLDLETYLKTLKTMISYAMFLRTMGIGQD

>AglaOR79

MSFYNINFCLKLFNLMGTHPDKRGSVLQVMIYILGAVLNLSIVILSILLLFIKERVTITDVTDSIETIGLLLHGFLKLTNLFIKRSKISNLLRTMEAHFWKIEDVTDTDARTSHQKFLNSLKNVLKFFTSLCAFLAVLFIIKGFVGQTVFEIYLPNWFPLYLSVFYQSFTCVLTISFPVISTDLFIFTIFMLTSLQFKLLNEEARNIFGKTGECGIDDEVIKARIKKCVDHHIFLKNFVKMLNDTFSQVLFIYNGDIVLSLCVEMYIVSTQNSFQTAAKAAVYVFTGMFQYTVCYCIAAQAITDEASKISKSVYFGNWYEHPEKYIRNATTLMIAVGQRPVTIIALSFIKVNLETCLKTTQTILSYCMFLRTMGIN

>AglaOR80CTE

MNSYYNVVLYCTGVHPSYSVGNFRVIANLSVTVCGTILVLMNIYKETKLEGVESLSLLLIVLIKYLTLVFGRRLLKKITYATENFWEVNITDEGIEFFKQHLDRVDKFHKFLSFTGMVAYAAKPLMQNNKSSILGCYVPPAIPYPVFFIFELYFLGFVSSTFISFNILICSLIISVVIQFRLLNSKIRTINFLGIETKHDMDICFRKLKHRIKYHQFLIRYVQDLNKLLSTPVAILIVFSVSLVCLNMYNLSVNKSSIVNSYKVMITVSSVIAEFFSVYGFPAQLMMDESAATADTIYYECNWYLPKVRPLRKDFLMIIMRSQKNVCIKAGNYHVVSSQTILL

>AglaOR81CTE

MISNFCNISLYCTGVHPRHPFGIIRIILNVLVVSCGTVLVLLKIFKENDMQGVESLSIFFLVITKYLTLIFGRRLVQQILKATENFWPIDITDDQTIFFKKHLDRVQNFHLFVMYTGMLTFAVKPFVLTDASSMFGCYVPPQIPYPLFYIFEFYLLSFLSLGFVSFNVMICTLIMSVVTQFRFLNYKIKAINFSDIENDHDLETCFSKLKDRVRYHQYLIGYVQELNKLLSTSLALMMIFSVSLMCMDMYILSVHKGTIVDHCKVILTSSAVISEFFSVYGLPAQLMMDESAATADTIYYECNWYLPKVRPLQKDFLMVIMRSQKSVSIRAGNYYVVSNQTILM

>AglaOR82CTE

MKDSYFYKHFMFLGIHPDKQFNKCRFIFDVITITCIFILALIKLFQDKNLINVELVSSVFLAAIKYLTLVNWRKMTRQFEYTIEEFWKIVVADNQITFLLRHLKAVETFYRYVFIFGPFVFMAKPLVTQDSSIFNCYVPPVIPFPLFYILECYLVFLGSSAFIALNLFACSLIVLVAVQFRLLNLKIKYLNLEDIENSQDFNRFVRELKCIVNYQQFLMRYVKDLNKLFCVPVAFLMIISVIILCSNMYILSTNQGTVVDRSRIMVTSSCVIVEYFLVYGLPAQLLMDE

>AglaOR83PSE

MISSYCYKYFLFLGVHPQQPFSKCRVFFNILITTFFFLMACLKCYQDKNLDYVESLNSFVLASIKYLGMVLSRIIQQILQRMEDFWKVNVADDKIMLLMQYLKRIETSFNCLLIFGIFIFALKPFIVKGSSIFNCYTAYDSISFVLYFEFYYLLLAAISFISFSNFTCALITLVVVQFRLLNLKIGSINLNAENTDGLDEFIRDLKTIVEYQQFLIRYVKDLNKLLSVPTALLMVSSVTEMCINMYILSLNGDIIDKTRIILVSLCILMEYSLVYGLPAQLLMDELAATADMIYHECEWYLPILRPLRNDFLIIIARSQKSVRLRAGNYHVINNQTILLMIKTAYSFYTFLHNIVQRANN

>AglaOR84

MISSYCYKYFLFIGVHPQQPFSKCRFIFNITIIIFFFLMACVKCYQDKNLYFAEILNSFFFVTIKYLGMVLGRNKIQQILQSMEHFWKVNVADDKIKYLMQHLKRIERSLSCLHIFGVLIYAGKPFIAKGSSIFNCYVPSIIPFPLFYVFEFYYLLLASTSYICFSSFTCALIILVVVQFRLLNLRIRSPDLNDVENTDGLEKFIRDLRSIVEYQQFLMRYVEVLNKLLSTPIALLMVSSVTELCINMYILSLNGEIINKSRAIIVSFCTLLDYTMVYGLPAQLLMSESAATADMIYHECKWYLPILRPLRNDFLIIIARSQKGVRLRAANYHVINNQTILLMVKTAYSFYTFLQNIGQRPRN

>AglaOR85

MENISYFKHFSFLGIHPSQPMDKLRIIFNCSLVSCICLLALLNSYFEKDLGVVDVCNGLIIVTVKYLGLIFGRAPIKRILEAMRHFWNTDVEDDQVKYYVQYFKTIEKVYKHAIFFGIILHVAKPFFVRGSSICNCYIPPRIPFPIFYAFEIYVIIVGAASAFCFNVFVCSMIVSVAAQFRLVNLKIEDLNARKIENDHDLRVYKVNLKSIIKYQQFLIRFVDDLNKLLTVPIALLMVSSVTMICSSLYVRTTHQGSVFEEGRFFIIIFIVTAEFFLIYGLPAQLLMNESEATADTLYSECKWYSPQLRSLRKNFLIMMIRSQKSICIRAGHYHVINNRTILLMMKTAYTFHTFLQEVT

>AglaOR86

MENNFCFKSFILLGIHPHLSACKWRVTFNNFLTACVIVLAFLKCYLDRDLQIVETISALVVIMVKYVPLALGRKKIKQILEANELFWKVNVADDQITYLLQHFNGVQKIYKNILILAILIYVIGPFVSNASSIFNCYIPPFIPFPLFYTVEFYVIINAAFAFVSFDIFICSLIVVVAVQFRLVNLRIVDVNTMEIENDHDVEIYIRKLKSIIKYQQFLMRLVDYLNELLSVPIALLMVTSVALKCISMYNMTINQGSISDKGRTIVTILSVIAEFFLAYGLPAQILMDESTATADTIYSECKWYLPKLRCLRCHFLIMMTRNQRGVCIRAGNYHVINNGTVLLMVKTAYSFYAFLQNVT

>AglaOR87

MENSFCFKILFLLGVHPYYTMNKLRVTFSNLLTACIMSLAIFKCYLDRDLEIVEAISALNIVIVKYLTLAFGRKTIKRILEAMELFWKVNAVDEQVTYLIRHFKGVEKIYKYIFIFAIVIYAVGPFISKASSIFNCYIPPHVPFPLFYLVEFYVLLICSFVFTCFNILVCFLIVFVTVQFRLTNLKIKDLNFGEVESDQDVDVYVRNLRSMIQHQQFLMRCVDDLNKLLGVPIALLMVTNVSLMCISMYNITMNQGSISDKVRMFVIIFTMVAEFFMVYGLPAQLLMDESAAIADTVYSECKWYSSKLQHIWKDFFIVIIRSQRSVCIRAGNYHIINNATVLLMVKSAYTFYAFLQKVT

>AglaOR88PSE

MENKFCFKSFLLLGVDPHHPKSKWRVTFNNLLAVCLMFL*LYKCCLDRNLQDVELISALISRLLLDSAEHFWKVDTGDDQANYLI*YFKALERVYKNMVILAVSAYTVAPLF*KNSSVFIPFHIPLFYIAELYVTVHKGLLSVSYNIFICSLIVLVTVQFRIVNFKISDLNLKEVATDHDAEVLRNLKSIIKHQQFLL

>AglaOR89

MENNFCLKAFVFLGVDPNRPIRKWRVTFNSALAVWIISLSLCKCYLDRNLQNIEPISALNIIYVKYLALAIGRKTVKQILESKEQFWEVDIVDDEITYLIQYLKAIDRLYKSSIFLAVSAYTVPPLLSIKSSVFNCFIPEYIPFALFYIVEIYVIIVGSFVFIYFNIFVCSLIVLAAIQFRLVNFKIRDLSLKEIENDQDVEMYVRNMKKIIKHQQFLMRYVDELNTILSVPVALLMVTNIPMICITMYNATLTGSVLLDQFRTLATILCVLTESFLVYGFPAQVMMDQSEATAETIYSECKWHLPKLRHLRNDFLIMMIRSQEGVSIRAANYHIINNRTVLIIMKTAYTFYTFMQKVA

>AglaOR90

MENNFCLKAFIFLGVDPYRPIRKWRVTFNNLLSVWIISLSLCKCYLDRNLQNIEPISALNIICVKYLALALGTKTVKQILELKEQFWEVDIVDDEITYLIQYLKAIDRLYKSAIFLAVSAYTVPPLLSINSSVFNCFIPFHIPFPLFYIMEIYVIIFSAFVFIYFNIFVCSLIVFAAIQFRLVNFKIRDINLNDIENDQDVEVYVRNVKKIIKHQQLLMRYVDDLNTILSVPVALLMVTNISMICITMYNATVTEWVLLDQIRMFATVLCVVTESFLVYGFPAQVMMDQSEIAAEATYSECKWYLPKLRHLRYDFLIMMIRGQEGVSIRAAKYHIINNRTVLLIMKTAYTFYTFMQKVT

>AglaOR91CTE

MENNFCLKAFIFLGVDPNRPIRKWRVIFNNLLSVWIISLSLCKCYLDRNLQNIEPISALTMICVKYLALALGTKTVKQILELKEQFWEVDIVDDEITYLIQYLKAIDRLYKSAILLAVSAYTVPPLLSMNSSVYNCFIPFHIPFPLFYIIEIYVTIINAFVFIYFNIFVCSLIVFAAIQFRLVNFKIRDINLKDIENDQDVEVYVRNVKKIIKHQQLLMRYVDDLNTILSVPVALLMVTNISMICITMYNATVSEWVLLDQIRMFATVLCVVTESFLVYGFPAQVMMDQSEVAAEATYSECKWYLPILRHLRYDFLIMMIRGQEGVSIRAAKYHIINNRTVLL

>AglaOR92NTE

IMFVKYLALALGTKTVKQILEYKQQFWEVDIVDDEITYLIQYLKAVEKFYKIFAIPAASAYIVPPLLSINSSVFNCFIPHYIPFPLFYIAEVYVIITVGAFLFISFNIFVCPLIVLAVIQFKLVNFKIRDLNLMDIENDQDVEVYVRNMKKIIKHQQLLMSYVDDLNTLLSVPIALLMVTNISMICITMYNSTVGESGLLDQIRIFATILCVLTESFLVYGFPAQVMMDQSEAAAETIYSECKWYLPQLRHLRNDFLIMMMRSQEGVHIRAANYHIINNRTVLLIMKTAYSFYTFMQKVA

>AglaOR93

MENNFCLKAFLLLGVDPNRPIRKWRVTFNNSLAAWIMSLSIFKCYLDRNLLSIEYISALNIIFVKYLALAFGTKTVKQILEFKEQFWEVDIVDKEITYLIRYLKAIERLYKSFVVLAVSAYIVPPLFSTNPSVFNCFIPYYMPFPLLYIAEVYVTICSAFLFISFNIFVSSVILLAAIQFRLVNFKVRDLNLKDIENDQDVEVYVRNMKKIVKHQQLLMRYVDELNTILSVPVALLMVTNISMICITMYNATVTESVLLDQLRTFATILCVLTESFLVYGFPAQVMMNQSEATAETIYSECKWCLPKLQHLRNDFLIMMMRSQEGVSIRAANYHIINNRTVLLIMKTAYTFYTFMQKVA

>AglaOR94

MESTFCLKAFPFLGVDPNRPIRIWLVTFNNLLAVWIMSLSLYKCYLDRNLQNIEPINALIIIFIKYLALALGTKTIKQILESKEQFWEVDIVDDKITYLIKYMKAIERWYKSFVILAVSAYIVPPLLSINSSVFNCFIPFHIPFPLFYIAEVYVCIYSGLLFISLTIFVCSLIILATVQFRLVNLKIRDLNLKDIENSQDVEVYVRNMKKIIKHQQLLMRYVDDLNAILSVPFALLMVTNISMICIIMYNTTITESVLSDQIRMFATILCVLTESFLVYGFPAQLMMDQSEATAEAIYSECKWCLPELGHLRNDFLIMMIRSQEGVSIRAANYHIINNRTVLLIMKTAYTFYTFIQKVA

>AglaOR95CTE

MENNFYLKGFLLLGVDPNRPIRKWLVTFNNLLTAWIMSLSLFKCYLDRNLENIEPISALNIIFVKYFPLALGTKTVKQILEYKQQFWEVDIVDDEITYLIQYLKAIERLYKSAIFFGVSAYTVPPLLSINSSVFNCFIPNYIPFPLFYIAEIYVATIGGLLFLSFNIFVCSLIVSATIQFRLVNLKIRDLNLKDIENDQDVEVYVRNMKKIIKHQQLLMRYVDDLNTILSVPVALLMVTYISIMCISMYNATVSEGNLFVQVRLTVTIVCILTESFFVYGFPAQVMMDQSEATAETVYSECKWYLPKLRHLRNDFLIMIMRCQEGVSIRAANYHIINNRTVLL

>AglaOR96NTE

VEVYVRNMKKIIKHQQLLMRYVDDLNTILCVPVALLIVTNISIMCIAMYNTTVNEGNLFVQVRLIVTIVCILTESFFVYGFPAQVMMDQSEATAETIYSECKWCLPKLRHLRNDFLIMMMRSQEGVSIRAANYHIINNRTVLLIMKTAYSFYTFM

>AglaOR97

MGNNFYLKAFLLLGVDPNRPIKKWLVTFNNLLAAWIMSLSLFKCYLDRNLENIEPISAINIIFVKYLALAFGTKTVKQILESKEQFWQIDIVDDEITYLIQYLKAIERLYKSSIFFSVSAYTVPPLLSINSSVFNCFIPHYIPFPLFYIAEVYIVIFSSFVFVYFNIFVCSLIVFAAIQFRLVNFKIRDLNLKDIENDQDVEVYVRNMKRIIKHQQLLMRFVDDLNTILCVPVALLIVTNISIMCIAMYNATVNEGNLFVQVRLIVTIVCILTESFFVYGFPAQVMTDQSEATAETIYSECKWYLPKLQHLRNDFLIMMMRSQEGVSIRAANYHIINNRTVLLIMKTAYTFYTFMQKVA

>AglaOR98NC

KTAKQILESKEQFWEVDIVDDEITYLIQYMKAIEKWYESSVIVGACIYTVLPLLSIHSSVFNCIIPHYIPFPLFYIVEIYIIIVSSFVFVYFNIFVCSLIVSATIQFRLVNLKIRDHNLKDIENNRDVEVYVRNMKKIIKHQQLLMRYVDDLNTVFSAPIALLMMTSITILCINMYTTTVTEWKLSNQVRLFLTILCILTESFFVYGFPAQVMMEQ

>AglaOR99

MENNFCHKHFLFLGAHPFHSLRKWLCIFNVTLIIYFLFMALYKFYRDNDPEALEIVMCMILVLAKYLTLIFRPKLIRQIVEATENFWELNVMEDPVTFFLEHLKRVENICKYTFVLALLLLGVKPLVVKGSPVFNCYVLPGIWYPFHYVAEMYTLTVAASAFLSWNVFVCFLIILVVVQFRLLNLKIEALNCEDVENEHDFDRNVRGLKSMIQYQQYLMRYVKDLNELLYVPLALMMITSVFLMCLNMYTLTTSEGTMVDSGRVMLTSFALVVEFLIVYGLSAQLLMDESEATADMIYFECEWYLPNLRPLRKYFLIMMTRSQKDVCIRAGNYHMINNRTILVIMRTAYSFYTFLQKVAYVKE

>AglaOR100

MKNKFCHKNFLFLGAHPFHSLRKWLCIFNIALLIYFLFMALYQFYRDKDLEALETLSCIILVMAKYLTLIFRPKLIKQIVEATENFWELNIIDERVTVFLKHLKRVENIYKYTFVLVLLIFAAKPLVVRGSSVFNCYVPPEIWYPSYYVAEMYTLTVATSAFLSWNVFVCFLIILVAVQFRLLNLKIKALNCEDVQNEHYFDRYVRDLKSVIQYQQNLMRYIKVLNELLHVPVALLMITSVFLMCLNMYALTTSKGSMVDNGRIMISCSTLIVEFFMVYGLPAQLLMDESEATADMVYFECKWYLPNLRPLRKHFLNMMTRSQKGVCIRAGNYHIINNRTILIMMKTAYSFYTFLQKVA

>AglaOR101

MYEEKAEIIFPEENEHLLKEELFWKINLLLKFRRFDDGRKYIWFSAIVLKAIIAVSVTIFAYKVKNEPDKLAACVATCPAILMAVFKLFYMYFQSEGIKYLFDAINQEFWDLGIAGSLVRKRIMRRFLLINLTLTSQVCLSFVCIYILNVYPLMEMPKGVKRLPNPVWTPFDTNVSPLYEILYFLLVCSQVLTVFGNGYYDFAYGSATQHLCAQLLLLKEQLKNITVGIMPHASDLEKFHSGYFQKRVMERLKICVRHHCRLLKYGKNLDKYSSSILLLQLLMSYLAMVINGYILTSNKADTLQTVEICSYTMTIFTEFVVFSVQASDLKDQSITVVDAVALSEWYLFKAPIKKALALLTMNAEKSIVITVGGMLEADNSLIIEVVLKAFSAITLLKALTIEEG

>AglaOR102

MVIKFPKPGEFILRDDHMASMRKFLKLHDFDGKTKYFWIGVISLEILLLVSRVTVIFQCLDRPRRVVDLSATIPITITALLKAIYLYFDKNRVKYLYNTIESEFWDFRICGPEVQKQAITRYKYSSLVLWSVVTNVIICYLLLQIFPMTPMQSESDRLLPKVVWSPIDLNPSPLHEIAFVIILLNGLLTALTNIIYDYFYVYCAQHLVVQLMILQDLLRNITEDVMVDLSDVEKFNSEDFQDTVMDRMKICAENHSKLLKYGENLGRFCSLVLVPQLALTYAGLVVSGFILATDGSSFYQDVMYFSLFLNTLVELSIFAIPSSHMNTQSLLMLDAIYDSKWYLFNSRMKRACTFMMMNGQEGISVKAGGITKIDNSLLVDMMQKVFSAITLMTALIDANQTK

>AglaOR103

MVQFPEAGEFHLRDDPMIMLKKILKMHGFDGKKKYFWTVATIIKIVLLFDRIAFVILCLDRPRRVAELTASLPVAMTGVLKMIYLYVDKNRVNYLYDTITSEFWDYRIYGPKLEKEAMTIFKYTNRILWTYCANSVFCLTLFLIFPVAEMPSENDRILPNVVWSPVDLNPSPLYEIVFTVLLVNGIMTYLGNVAYDFFYSYCVQHLIVQFVILKELLRNITDGIMDDSSDVEKFNSKYFQDTVMERLKICAEHHSRLLKYGKNIESFCSLVLVPQLVLTYAALVVNGFMLSTDRSDISKTIALFNLSFTTFIQLVQFAIPSSQLNAQSISVLDALYGSKWYLFNARLKKACTFMMMNGQEGIVVKAGGTTRIDNPLLVDMLQKVFSAVTLLRALIGVDGNTAK

>AglaOR104PSE

DCLKFVRILFIDFYGHKYTKICTFIYLVFHVPIWIEQYYQYFKTFDLLMVAQYGPGSFILLFGNDVCCRHLYQCYYPQYVQRTCDCSFGIRKNFSGLSTQ*ARNLEELCSMRYITYIATINLTLGLLTYVAVIMKGTRDNNWEDIYQITFIKKHFPRYSGILIVTFYASISYLILSMLMPIYAMMYVTAHIFFQSAILCEYIKRIIENYPEDNELFYKESYQKRVFQKFRFCIMRGMTIKRITNQQIAALQPLMVLFIVLGMLLVVCFVGSWQLNINKYYPAVFLLIITSGLACLIIVEVGQWIEEKSLEEYYILCALPWYTFDLTNQKILLCVLLNAGIPITLSPFREFPINRALIVKFYKVACSVYAVAVHMEKKIPLTDGIMK*

>AglaOR105NTE

SLKLLLAFIKLSTNRYVKILSYFLLVFHTSSFIWQIYLLSKIWENSLISQYSPCLLISFTGIFNISMSLSFEKELRKFMDLVENNFYSLDMAGEETRQRIARESKQIEYFLCINFLLVLTTCITEVHNEGFNQEHFLFTTYFKETFPTQAHVLNYSFYFLYLILYMYLLNGAYIVVYLSKQSIFQLYILQYCIFYMLGRTSNVCEKCIQFNENYQKKVFKRLKFCIKYQELLMRNGRVIIKISNKFLGVSVILLYILWVSALANIIWSPVTNYPYFICLAITLAVTGGMAAEIGDNVSEETTNLHYAFTNLPWYTWNQKNKKMFLFILIHVGKGIHVGPLKMFIVNRTLMIRWVKFLWSISSLIGKIRDT

>AglaOR106

MEKKEVVNDMFENIRFMKDDPFTHLLYLLDFGYKERIFQINIVKCLIVLYIQIFNVNSIVSIIKDTTGTYFKQYMSNVLYLQIGIINYMFHIVYGSYIVQFPDLTQKNFLSLDAAGRKVHEKLLRECSLIKRGIRLLLISIFISFIIILPIVGDEENVMFEVKFIKQNFGKWQKLFLGIYFLYVYSFSYSCAVTYLSFFYVMTHLRYQFILLTELLKKISHGYSPDVDNEFYQAKIKKRLKVCVQIHNRIIKVSSIFLELGRYAIIILYISVSLDCASAVDYIVERISPPSDFRAAGILVMVGTSLVVFSRYGQILQDTSIEFYDAVCNSDWPYWNIQNRRTLMIIMMNSVKVTKVSSFDIVDLGHLTVLQVAKVVYSIVLLFVNVKH

>AglaOR107CTE

MDGAHKTIETSRLLYTGNYMKDDPLIYLIGIIHFNGKHYTNYFNTTRILIIIYLQTFNILGIVYIYKDESGAMLRQYVSVILSQQFGIVCHILLIYYSRSILRLPNLAKLYFLPLNATSKKLHQRILRQCATIRTLHPYVFIVSIVTYLIFLPFVSDQEDIEFTAMFINKYTGTCSNFLLVLYYLCIISLGHSLASMCSICLYITIHLKFQFELLTEYLIQISCDFKPSNENVFYQDVIRRRLKSFVQSHNNIKAVSPMFLEIAQGAVLMMAVAGALAYTALVYFIASGISPPSNFRFIVSLLGAVVTTYLLVVSGQDLQDKSIDVYTAACRCQWVYWNARNRKTLLMIIMNSSKVTKLTSFGILDLGNEIVLR

>AglaOR108INT

MSKNKNLKICGLLGETFMKGICGYILSIFYVKRVLRLSDFIKMSFLPLDATGVEVQRKILHQCVLIKKLFPLILISIMIMVCITSILSDMEDINFAVMFIRKYFVFPNFFLGLYYLCFLSLGYGIISLYIATFYLTVHLKFQYCLLSEYLKQISHGFKSNTEDHLYQDVIQKILRSFIQIHNKIKIFSFELQEMGNWTVLTVSVSAVLLYTAFAYPIASGASPKTNFVCISNLILSAIVALIMAGTGQSLRDESLNIYSAACQCDWVYWNAQNRKTLQMIIINTSNEVKLSGFGLMDLGHEIIITSLKVSYSIVLLFINLNN

>AglaOR109INT

MSKTENEKICGLLGETSMKDDPLIFLLNFLRIYMKNQIPYVNIVKSFIVLYLQTFNVIGIVYICRDETITSLRQYITFILAPQIGIFGYILTICYLKRVLYLSDYIKMSFLPLDATGAEVQRKLLRQCALIKKLFPLIFIVIMVMMFKTSVLGDVEEFHFAVVFIRKYFVFPNFFLGLYCLCFLSLGYGITSSCTATFYLLAHLKFQFYLLSEYLKQISHGFKPHIEDHLYQDVIQKRLRSFIQVHNKIKIFSFGTLDMGKWAVLAVAVSTILFYTAFAYFIASSVNMYSAACQCEWVYWNARNRKTLQMIIINTANEVKVSGFGILDLGHEIIIVILKVSYSIVLLFVNVNN

>AglaOR110

MCKLDKSANKHKLFAKIFMKGDPLGFLAYPMHFNMRNHITLINMVKALIVLYLQTFNIIGMVYILKDESGVYLQRYTSVILAQQIGIISLVIHIYYTKDIMLLRDVIEASFLPLNAVGKELHQNLLRQCSLIRKLYLLVFILAIPILCNVLPLTAGREDVQFTVMFIKKHFGVWSYLFQGLYYFCLMALGYGLASVCTVCLYLMTHLKFQFRLSSEYLKQISSDCKLDYEDHYYQEVIKQKLRSFVQAHNKIKATSVFLEIAKWPLLVLTMASILSYTAFGYFAVSEDIPSKFVRFGLGTVIAAIFTVILITSGQGLQDEAADLYNAACHCDWNHWNIQNRKTLLMIIINTSIEVKMTSFGILDLGHQILLLLLKVSYSMVLLFVNLKK

>AglaOR111PSE

MSAKEENARERNLLGDTFMKDDPLFFLIYPVLFNMRNHIFLINMIKGFIVLYLEVFNVIGIVYIWKDEIETYMQRYISVLLAQQIDILVPRVFIKEHFSVWPNFFQGIYYFCLLALGYGIASTSNSCLYLMTHLKFQLHLLSEYLKRISSGFTPDCEDAIYQEVIKERLRSIVQAHNNINRTAAVFLTVGKWSLIISAMSTTMSYTAFGYIAVSEDIPSKYYRCGLGVMTAGIISFIIIRSGQQLQDASANLSSAGYQCEWVHWNVQNRKTLLMIITNTSKKVKMTSFGL

>AglaOR112INT

MDDTCKISKTHKCICKMFMKEDPLHFLIKPLYFGMKKHIFVINVIKSLIILYLQIFNAICVVYICSDVTYLRRYISGILIQQVGISSYALMIYYARDILHLSDAVGTSFLPFNATGTKIHQELLRQCALTRNFFPLTFILLTAVHCGLLPVVSDFAHYQFAVTFLKEHLGVWSYFFLGLYYLCVLSAGYSIGCICCVSLYLGLHLKFQFQLLVSYLKQISLDTDANIEDGFYQDLICKRLTSFIVAHVKIKRLSFMFLEIGKWSVLVTTVSCSFLYTSLVHFIAMSSNLYSAACECDWADWNVQNRRILLMIIINTSKTVKMTSFGLLDLGNVIMLRMLKLTYSLVLLFINVNK

>AglaOR113

MDAKEVRDTFRLLDERPMKDDPLFFLLYPLYFNMRDRILLINTAKCLNILYLQTFNVIGLFYIFKDETGVYWKKYLSVIFIQQIGISSYLWQIYYARHFLYFSELTGKYFLPFDATGTKVYQRILGQCALTKKLLLAMFTGLIGTISILLPIIGNEEDIEFTVMFIKTHFNVWSKFFLVLYYLCVVSLGHSIACSTSMILYLAMHLKFQFYLLVEYLTQISCIEPNIDDVFHQDALHKRLRSFVRAHGKIKTTAAIFLELSKLSVLVSVILLSLLLSGLAYLVMMDVSPSIYFKCILALSISATVGYMITKGCQDLRDESTNLYNVACQCKWFLWNVQNRKTLLMIIANSSKEVKVADYANLDLGYEIIIAIWKITYSIVLLCLNLNK

>AglaOR114INT

MDAKEVRKTFRLLDERPMKDDPLFFMLYPLYFNMRDRILLINIAKCLIILYLQTFNVIGLFYIFKDKTGVYWKKYLSVIFIQQIGISNYLWQIYYARHFLYFSELTGKFFLPFDATGTKSASLYNVACQCKWFLWNVQNRKTLLMIIANSSKEVKASGFGNVDLGYEIIIAILKITYSTVLLCLNLNK

>AglaOR115CTE

MDAKEVRKTFRLLDERPMKDDPLFFMLYPLYFNMRDRILLINTAKCLIILYLQTFNVIGLFYIFKDKTGVYWKKYLSVIFIQQIGISNYLWQIYYARHFLYFSELTGKFFLPFDATGTKVYQRILGQCALTKKLLLAIFTGLIGTISILLPIIGNEEDIEFTVMFIKTHFNVWSKFFLVLYYLCIASLGYSIACSSSMIIYLTMHLKFQFYLLVEYLTQISCLAPNNDDVLQQDALHKRLRSFVHAHGKIKTTAAIFLELSKLSVLASVILLALLLSGLAYLVMMDVTPSTYFRSILGLGTSLSVGSIITKGGQDLRDE

>AglaOR116

MFTFKKIVQLLFGECITNEEDILHPIINLSLLLFRTRIHKMIGFIILILAGLENILYVKYVIRRPPMEEVIKFLPMQIVTVAGMVAYTYTIIYFNLCKGLIETMMHFLKPISEASEKTIQRIKKETKYIKMFMIIPLVATVVSPLPWFRTYEEVHFIIMYINQRYAVRYTVLLFQMIYLFCLSYSCFTATSIHFYWLYYVYHIKFQLYILRENVQELMTERDMFYDDVYQNEIYDKLIKCVQHHQAIKRCHAVFNHTMYWGLLVGQGSAIVIMTISVIFIIARTEPLPDVPVLVLFLITNMLLWEYCYFGQQYEYEWETLHLTLNNLPWYNWSIKNRKFLQIFIINTQHALSINSFGRIKANHEIITKTYQAVYTLITFFCTVSTRN

>AglaOR117

MSTLKKIVQLFFGECITFEGDILHPLINLSSLLFRTRIHKTIGFIILILAGLDNILYVKYVISRPPMQEVIKFLPMQIVIVAAMLAYTYAIIYFGLNNGLIETMKHSLKPISEASEKTIQRIKKETKYIKMFMIIPLVAVVVASSPGFGTYEKIHFIIMYINQRYERVLLFQITYLFCLSYSCITVTSIHYYLLYYVFHIKFQLYMLRENVQELMTERDMFYDDAYQNEIYDKLIKCVQHHQAIKRCHAVFNHTMYWGLLLSQGAAIGVITISVIFIIRRTEPLPDVQVLVVFLITNVLLWEYCYFGQQYENEWEMLHLTLNNLPWYNWSIKNRKFLQIFIINTQHALTINSFGSIKTNHEIITKSYQAIYTLITFFCTVTKRN

>AglaOR118

MSTLEKIVQLFFGECITFDGDILHPVGNLFSLLFRTAIHKTIGFIILILTGLENILFVKYVISRPPMEEVIKFLPVQIVTVNAMLAYTYSIIYFGDELIETMKHSLKPIGEASEKTIPRIKKEIKYIKMFMIIPLVAVVASLTGFGTYKKIHFVIMYINQRYERVLLLQMIYLFCLSYSCVTVTSIHYYWLYYIFHIKFQLYILRENVRELMTERDMFYDDAYQNEIYDKLIKCVQHHQDMKRCHAVFNHTVYWGLLLGQGAAIGVITISVIFIIRRIEPLTDVQVLVLFLITNVLLWEYCYFGQQYENEWEMLHLTLNNLPWYNWSIKNRKLLQIVMINTQHALTIKTFGSIKVNNEIITKSYQAVYTLIAFFCTVTRN

>AglaOR119PSE

MSTLKKTVQLFFGECITFEGDILHPLINLSSLLFRTGVHKTIGFIILILAGLDNILYVKYVISRPPMEEVIKLLPIQIVAVACDLLYLTLNNLPWYNWSIRNRKTLHMIMLNAQHALAIRSIGSKRANSQIITMLTRKCTYTFVTFFCTVTRRT

>AglaOR120

MRAFQKVFNWFFGECETFDGDVLHPLFNLVTLLHRNSANKIINLIIMVLSGLANILYVKYANSGPPVQDIIIFLPIQFLIFGGMVGYMFVFALFNLYQEVIKTMRHSLKPINDGSEKTIQRIKRVSKNLRKIVIIPALTALFTLSGLGGSDEIHFILVYINHRYGRKLMFRLIYYFCLSQSSFVVMSMHLLWLYHIFHIKFQLYILTDNIQVLMTGTDMFYDAAYQNDIHVKLKEYIQHHQAIKRCHAGFNQCVYWGLLIGQSTAVAVITISILFIIWTIQFDANVIIALFVANLLLWEYCYFAQQYENEFDLIYLTLNDLPWYNWSIRNRKILHMIMMNTQHALTMRTIGSHRVNNRIITMTYREVYTLVTFFWTVTRWT

>AglaOR121NTE

DVLHPLFNLVSLLHRNSANKIINLIIIVLAGVDNILYVKYVNSGPPGQDIIIFLPIQIYIFAGMLGNMYVFAFFNLYQEVIKTMRHSLKPISNGSEETIRRIKRIAKNLRKTLIIPALAMLIILSGLGGSDEIHFILMHINQRYGRKLMIRLIYYFCLSRTTFSVMSMHLLWLYHIFHIKFQLYILTDNIQVLMTGIDMFYDAAYQNDIHVKLKEYIQHHQAIKRCHTGFNKCLYWGLLTGQLAAVAVITISIIFIIWTIQFDVNVMALLLVSNLLLWEYCYFAQQYENECDLLYLTLNDLPWYNWDIRNQKILHMILMNTQHALAIKSIGSHSANNQIITMTYREVYAFVTFFCTVTRRT

>AglaOR122PSE

MSTLEKIAQLFFGECITFEGDVLHPLFNLVTLLHRNGANKIINLIIMVLAGLDTILYVKYVNSGPPGHDIIIFLPIQMYIFGGMLGNMYVFAFFNLYQEVIKTMRHSLKPISNANEKTIRRIKRIAKNLRKTLIIPALAMASESSTIQFDVNVMALLLVSNLLLWEYCYFAQQYENECDLLYLTLDDLPWYNWSFRNQKILHMILMNTQHALAIKSIGSQQPDYNNGELIP

>AglaOR123

MINFYRSSYLLNDVYIKLFGPLVTFEGDTISLLMHLSSLLCRNKLQIRLSMISSSIMVFNIVYYIYYSLFSSNARDEALKFLTGQITSFHFFVSYCLMYIFATSYLKVRNLYLKNYKPIFTGSDEVTNSIQKESRLAIKFAWVLVAAVVVNVTLFLPWTGDEEDFIFILNRFKLHFGNDSLGILLQLGIFFTIYHAAYVAITPMFIVIYYMLQIKFQLALLIEKVERLNLIESKNLSKFVTNDVNHQRKIKRRLVSCIRHHQFIKRFQRDLGERIYWPTFILASSVLLFETSLLIFYLSNVCPSSNYRILYSFAVTILQHVVFGFVGETVVEESERLYVAIKCCNWYNWDKKNCRLLEIFLNHTQEPMAFTSSGIIGVSKELIAKSFKVLYSAITFYHSMSNNRSMKQ

>AglaOR124

MWFGPLTTFEGDTIAVLMHFSTLLIRNRLQRFICTLSSMVIGFSLLYFLCYVVFSRPSRMEVMGFMATQLNTLHFYISYCIILRYKGTYLEIIDAFLKISKPIATGSDELVAIIKKDSKFVVKLVFVFLSAVAVNTSLFLPWTGDEDTVLFVLNRIHLYFGESSFRSLLVIVEYIFFYHVSFTATIPMIATIYYMLHIKFQLALLIENVERLNLLAHENVPELKIYDVNFQRKIKRRLVSCIRHHHGIKTFKEKLNDEIIYWPTFILSASAVVVETSLIIFYLSKINPSSNYRMIYTWFLMTLGHTVFAMAGQTIVDESERLYIALKCSNWYNFDKNNCRLLTIFLIQTQEPMVITSSGIIGVSKELILKSYKVLYSLITFYNSMSSKDPKK

>AglaOR125

MTRLFAFSRTLVIKLIDNLLAKLIVRCERLRYIYNEWFGPLATFEGDTIAALMHISTLMGRNKLQRRICMLFSVFAGVCVLDFLCYIIYARPSRREIIGFLTLQLAALHYCVAYFLVQKYTGMYSEVMESFLKNCKLISTGSEKIAGIIKKESKVTIRLVFVFLAVLAVNLTLFLPWTGDEDKINFAVYRIEKYLGDSFFGILLQILLYFCCYHGAYVASIPMFTISYYMLHIKFQFVLLTEKVEKLNLLGSKNSSDLIIYDVNFQKKVKRRLVSCIRHHQGIKRFKQKLDKIVYWPTFMLASSVVLFETSLIIFYLSNIYPSSNYRMIFTWSVTTLGHTVYAMVGQTIVDESEKLYTAVKCSNWYKWDKKNCCLLTMFLMNTQEPMQITSSGIIVVSKELILKSYKVLYSLITFYKSMSTKND

>AglaOR126

MMKFVLSNFPYKKLNITDDPLYFIPSLLDAILRRKLIKIFIATSVLYFQLLNSFSFFFIMTGQIAFQEYIALSLLCQVEIFGVISLLDFMRKIPTVLKTTVEYSWPVNIAGHKVENKIVYRLNLLNTIFLWLTSVSMVTHILIGLSYIGDESHNPYVRIAVEYLPYWEGLIVLIYFIYLPCFCFGCTFPVMLVFYLLTQLNNQMELLNNRLLIISRKFKRNEALLHNENYQNFIFDSLKSCCQHHCDILRMKKKYLSICRLWVLFLPVTSLYPAVVAYMLLNGTDVASSFEMISVMIVTMVLLAVASLLGQIITNKSEEIFKIAYECPWIYWNKRNRQTLLFLLTNSLRPMEFKSYTNIKLNLSFLFTIYRCIYALITYLY

>AglaOR127

MTRISLINRSHNSMRDDSDPLSFIEKLLNMNLWRSSIFRIITVVVAVTIQSSNIACLVTDKNNIHRYISLVLTTQPGIIATITTMFFSSKLDQSLKPSTRNFWPIRAAGKDVENKILQTLKIINRCIVLIILVFIGGYFITFLPLVGDELKINALEMISVNYFGRWSPLFCFTIYIYCPVFAFGLLYPFLLVSYLTALATIQLKLLEEKIKTMANDCNDEEGNHLLYNQNYQNDVYQRLIMCIEHHIRLKKFRNTWVKHSRFAMLYIPVASLAPVGAGYFLIHQISPSSNFRMVLVLLIFATSVTGLCFIGQHFQDSFAQIFTVACNCPWIYWNEKNKKALLLLLINSVKPVEIASYTTIAVNLRLLTTFQKWIYSLITLMH

>AglaOR128NTE

MLKTILGKKLLQHRLTRLFIISSVIIHMGYIVIGLSLALEKGNFHRYVPYFLLYLPCICAMVSTIFMSSSLKKIDVTINMKFKSPIIQKKVSQECRFLYRASIVLYVVACMAFLIGQVPLFGDEYMFIPIQLILQNYSFPKEVGIVSTILSSISSLAVAYPSVSGLYLTTLIKIQVYLVSDKLISISDGFGNLKGDELLDDKLYHDIVYKRLRICVKDHVKLIKFYRKAVEYNQIGITVAPVSSLTPVAILYFILNDISARNNFRMVPIMILFLYQLVIYCKLGQDVEDVGNYLYTVVTKTPWVFWTKKNRQALLIMLMNAKPMEVKAYNLISLNHRLLLLVYKVVYSLITVLI

>AglaOR129NTE

IICALPITMIMSRNINKLKVTTNMEFNDPNIEKIVALNCRIFYRTSVICYVLVFITIVITGVPLFGDEHTFIPVQLILQEYSFPRVVGIATTIHSAFSFMGIIHAFICGLYVIVLGRIQVYLIIDKLKSISRGFSNLKEDELMDDKLYQNMVYKRLIVCIKDHIEFIKFCKKAMEYNAIGIVFIPFATLYPVAVLYFILNDISAKNNFRMVLLLFVFIYVLVSICKIGQEAENETNYLRRVITETPWVWWDRRNRQALLIMLMNAEPVEVKAYNLITLNLRLLLTIFKWVYSIITVLV

>AglaOR130NC

DLFQFALTMLGYKMRRHIYIIKTFIISAVIYIQIFTGLYILEIIFFDGLDNALQFLPLIIIHQTFCCINVILCLNYNRMMRAQEDIIFKYGWCVKFANAEVMNKVEREVKNLKLTIVFLEIVIMSTYFSFVPLIGDEDQFSFCVMYIETHFGPWTPIFSVLYYLCISILAYGSTGYVIFMLYLSFYGLVQYHQLNVYLKEFAEDLTFRKHADNSQDMIHRLLSNFVDHQNDLRCATKKIHDFQKDVVFFVYFFSAVLILTTAMYFILW

>AglaOR131NTE

FLEIVTMSTYFSFVPLIGDEDQFNFYVMYIETHFGSGASIFSVVYYLCMSILGYGSTLYLALTMYLTFHLLIQYHQLNVYLKEFPDDLTLRKHADNCQDMIHRGLSNFVNHQNALRCAIKKMYEFNKDLIMFIYFFNGVLVLIAALYFILWNVSPPSNFRMVLVIVTMLMQSIAFTTIGEKLSDETEQIYYTIINCPWVDWNLKNKKSLLILLTNNMVPYEINALGILTLNYKIILSEFKMVYTVVVALYSSQK

>AglaOR132NTE

DPFQFVLTMLGFKLRRHIYIIKTFIISAIIYVQIFTGLYILEIIFFDGLDNALQFLPMITIHQTTCCFNMILYVNYNRIMRAQEEIIFKYCWCVKFANTEVMNKVEREVKNLKLIIVFLEIVTMSTYFSFVPLIGDEDRFNFCVMYIETHFGPGAPIFSVVYYLCMSILGYGNTLYFALIMYLTFHGLIQYHQLNVYLKEFAEDLTLRKHAENSQDMIHRGLSNFVDHQNDLRCGTKNIYEFHKDLIMLVYFFNGVLLLTAALCFILWNVSPPSNFRMGLVIVTVLMQSIVITAVGERLSDETEQIYYTIINCPWVDWNLKNKKSLLILLTNNMVPYELNAFGILSLNYKILLSEFKMVYTVVVALYSSQK

>DponGR1

MSKPPMAAQMDGQFLSPYPPEGPDVSRRKSDNIRIVTPETVRPEHVPDNELLEKLHTYDN

FYQTTKSLLILFQIMGVMPIQRLRGKTIFRWFSANTCWAYFVYTVETIFVSIVFKERLVL

ILKPGKRFDEYIYGVIFLSILIPHFLLPLGAWRNGQEVARFKNMWTKFQLKYFKMTGTVI

KFHRLTLTTYSLCVLSWLVGILIMLAQYYLQPDMLLWHTFGYYHILAMLNCLCTLWYINC

TAKGRVAGWIAEKLQEALQTKGSAKKLLTIGKLWVDLSHMMQQLGTAYSGMYALYCLLVL

LTTI

>DponGR2

AYFQLFAVVQFSLFLQKWIIYSFLLLLAVAGFIGYLKYYNINVTRTAEGRFEEAVIDYLF

TVYLVPIFVNILALYEAKKQANVLTQMVSFERIYTRMFKKRIGFDLGSKPLVMTVVLLIL

GCGVMVITHFSMANFIIYQVVPYCYINVITFIIGGAWYIYCDIIGNIATCLAEEFQFALR

NIEYSNRVCDYRSLWMILSKIIRNVGNSFGYTLTFLCLYLFFVITLTVYGLLSQIQEGMG

VKDIGLTITGVSATLMLYFICDEAHYASTCVRTYFQKKILLVELSFMNEDAQQEISMFLR

ATEMNPTDMCLCGFFDVNRNLFKSLLATMVTYLVVLLQFQISIPSSGDDVTNNTTNTTPS

APK

>DponOR1

EVIEYICLRIRHLKTVLLEALACPNRATRRGLFGRAVQYHEEILRMGRLADEFFGTELVL

HVVLTGAILGVSAFVMLESATLETLMIFVGWLNAIIMGCAAGQRLINESATISDVLHEVD

WFEFDNGLKKDLSFFLGAQQEVHV

>DponOR11

MNLSKFIEFPRKTLVLTGSWPQQHSSWPYLSRRIIVMTSIALLLLALVYNASFHFDDPIK

LSESLFILISVVNVFLKLIIMVVNEKVFLNLIARLETSTFIKGGILYQPIYVKFMEIVRP

VYVTYFILVCGCVTFRSSFPVFSLSSVAYDNMAIDLLVVGVVSIAAVQLQVLNSKLRDTK

QNVQFLPNYSISNHEALTVGYLKDCCIHYSDIEEYIKCLLDMFSIIILVQLGSSIVVICS

SGMVLLSLKPLSIEAISLYFYLITMFTELGMYCWFGNFVYVESLEVINSCYLSHWEERGP

AVRKTLFMLMERAKRPLEIKAVRFFTLSFDTFIVILKWSYSYFALLRNWMAD

>DponOR12

MEKDKFKILSLHINVLIFLQFWPNPLFNNVVNNHIMSIVCFITVTSCIPCIWTIYKVFEG

MYDIGILFESFICFVNIMAYLTAYWTIFRNKAVIENLINDICIFLPYCPTNLIRDTDASS

IRYTKYLIVYVTLGVFVNLAWPAISPEGCMRQRQSEYLLKHDPCGMPTHNYYPFDASKPI

PFWIAFACEALLTCNICILFSMVTAIILGLLMQITEQIKHCCDKFEHINFKGDVETARKE

FLECVRYHRAILEYAERVFTVFAPVMSAYLVVTSFATALIGYQIVETDNTQDRFRYAMLL

LAWGCLFFMICLYAQILQDESVLIADALYNSDWTCNSIYFRHYIIRVIARAHKPLYFNIS

FLGKISLTRFVSVMKTAYTVFTVLVTVVDRK

>DponOR13

LSAALFPWKIHDTKSYMFTLFIQTWTSIIGASVCVTLDCTFAIFSTIVKMELHLLKEKFK

TMDFSSTNKQIKYELKKNMELYDNIVKITFAIQKIFNYVLLSTFINSIILICLTGFLSTH

TVIDEQADLSEKINYVYTLGYFGYLLFQVSVYCYYGHQIMTESSDINEAIYLSN

>DponOR14

MLDISVSAVSGNCVIFAYTLVILLQCVIEVTGQLYLLTTKPSIGKILLLAPMFFTSFMVI

TTIIALLFQRSSHGRIYSQYEALSMEQLRRANKPILAEVRQLFDAGIYQFAAFLAAVALG

SIGYLPLNEFDYEHNVWPAIQALKLIEDGPKAVIYTIATLNYLVMPGKGCILLLYGLHIM

HLCSLYCVSSILLRGKLKGIAVNRPAAADLMLSDQAWVTQELKSCIKQDVRLKTFCSAII

DHFKWILLFHVALAVIILSLLYYINLFLNGGYSPLGSRCFVAIANILSFTYSYNSENFRE

QVENIRQEICNLPWYSFNKCNQKLVHVFLSNMLQPRYLSVGGLFDANHEFLVLVMHKVFN

VLTILSNLDPDRAGN

>DponOR15

MGGSGEFSSNILNLGISALHLCATNRVSRWFLIKNEFEVILANLRKINTDFSLFDYQSHQ

SGFKMDANEPEIHNEEYIGQMAWRNLNCTQATGFKLRYADDTASSAISNAIIKYKTTKLL

ETKRYCLGIFLTFLINVALNISISYTINYGNPLYEKWNPLLNKTSVYRDYPYPLLYPFDT

SVSDGHYLLGFFYQPYAFFCLMCAFFCIEYLCVGTIIHLTTHVNILGYAFSYVDENIDPM

LDYSKVIMLKEKRIIKLSGELKEIYNCAKELNAVFSGQLLMQEFLMSTVMCCCVYRVTTN

ISTAEIGYLSTMVAVCVAEMFTVSWFNQCFTLELFKIQQRIYELEWIDYPPKLRRVLLFL

MCRVQKPFNFTMGFGFPLDVNVFLSMIKTSYSFYTLITRSGSKFSNEDV

>DponOR16

MDFIQLFAPFKLILNISGFWPQKNPKPIVEVRKVFTLLVNLFFCLSLSIQCLFLRSQIEE

FLDVLTVITPPVAYLFKQIVFFGHSGAFLTLMDFLKDDDLVSIPLQLRKQISDSLQVAKI

IGVGYQACCTMTILFIVIWPMFTEHQLPVQFTLFDLGDFYAFMYLLQIFALANAAANSSS

LDLIALTLMCIVKGQICVLNDKIRSLGEINASKGHGAQRVKYVSGCVLHHTKIIELVALI

ENVYSQIVLIEYLTSMVVICNIGFQLVIVELASFAFLLMLTFLVTMLCQLGMYCWFGNEI

MLHSAAIRDACYESDWIHSCPQVRKMLLMIMERSKRPLYLTAGKFSILSLNSFTSVIHSA

YSFFALMQRMYGKSTTF

>DponOR17

LNYQAAHAISIESQNLAGSIFKGDWHSYSPKILRILQLVCMRAQKPLAMTLHSFANVQVN

LLIKIFKALYSYICLILKT

>DponOR19

MYPIRKDLPFYASLRMLESIGFYSENTNGFKKRSIVRTLIFCILCWSIILLSAVLLIYEN

LNDKNYASVFFNIAVAVASTSTYCCTLLFVKYQEKWSDILTALVNYEKFGKPRRYNQLKE

RGDRVAMACWGGILTGVFLYMLFAILHENDCELEKTGGGVCGLIIPTWLPAPYDNSLLAR

RLVLLYDIPNAAAVSSFVLVTHLNIQVNEFNIARIDHLSLLFNDIEFCKDPQAQLNKMKH

CIEYHQDIIRVSLQFKNLSKRTMGHMTLTFTIVTASMGCQLLQTSKNFYQENAFFFEIIY

VINMFIMCYCGQRLEYKMKTVGDFLYSTHWYNLNPKLQSLIPLVILNSQKTIRMDAVPIG

YLNYELFVTLLKTTFSYFSVLTQLT

>DponOR2

MKFFKKTEENVFFGFNIMVLKACGLWPDLIDYRYDKWRLMKDCLMISSLMPCAVPIMADF

IMQLYDGVPNLTAAVENMIALNCIIGMIYMVICFIANRRTIIKLMVNLKYFNKYGNSRKT

KEVDEKANLFSKIFMFYGILGNFVYMLMPQLSIDKCHNNRTAKMIDDGVPCGLVVRSVFP

FKFDYKPVFEIIFVHQIYTCTMVSIVVLALTMLLCGFLMHIVNQLKNLREFIAKLRHCPQ

DKPGERLFFIIQYHIDVIEYSQNTAKAFSTMLLFYITLTSLVLSVLCFEVIMVDAFEDSV

RFALHLVGWLAILLSVCYNGQLMIDESVEVANDIYSLNWFNFPVGIQKKIQMIIMRSQKP

LILDAAGMGLVSLPAFLKVLSSAYSFFTLLLKLK

>DponOR20

MLYPVRKGLPFYHNLLVLKLAGYYPKSSNYNKKLFFYCLVCWMSLWTGTLWNLIILLYLS

IQNKSSHGITEAMGYLIGNSSLALICLHFALKHQDWSHLMDALIDFQKYGKPPKFNTVQT

NASKVGITFFKVLFCAAVMYCVLQVLLEEECEKKLTFGKTSCGMLLPTWFPASHAESKLA

KRLLLIYQLLACWAIAPFTVIVSLVLQANEFIAYRIDHLKSLLRKVGSNENPDLQLHQFL

AYVQYHHHIIRLCRKLNYVAKYTTGHVALTFVTVVACFGHHSIQEKSLQSLTYQATYVIS

MCILCYAGQNMQDQMRSIGDALYSSTWYNCSLKVQKMIPLVLLRTQQPIGLDAVPLGVFN

YMLMVMVLKTTYSYMSFLSRTI

>DponOR21

LKEYSIHPMLHLIGYAMAVFLVCQNGQKIRDQTYDIQDAVYKARWYDNITSTTKDSQLIM

LRCQKPLCMDAIPFGIFNFSLLLVIIKTSYSYLTLINKTS

>DponOR24

MSVLGETTQIKKKWRSSIAITEKVLVITEIWPNDDTSLYRTMKVVFITIVCIVFNLTVID

ELKMLAIRQDYKTLSMHLSTFGLYIGFSVKIILFQFTKHGPLKNMLDSMDSPIFHAYPPE

MQKHQDNCIRVSNLIGKFFVYLVGGTILFYLNKPFYSSYPLPITFSHPLTTTTFYLLLTL

QCVCSFYLIMIGICFDMLVMGLANVATAQLDMLIEEITTFTPTSIETLEKEEHRFIKRCA

ERHNAIISYVNSIEDVFTYIFLAQCVVSVTCICNGLFQLTHVAPVFSIHFYYNCIFTFNV

LFEIGICCWFATLMTNKGNDVADACYNYNWLHSSSATRKLLLIMLCRSQKPLFITVGKII

QLSIGSFLSVLKTAYSYYALMQHLYDKTSQ

>DponOR29

VDLVSFPFLLMAAFLLCMFCQLGIYCWFGNEIILQSLLTREACYEFDWINADLQVKNMLL

IIMERSKRPLHLTAGKFSILSLDSFTSVINSA

>DponOR3

MMENKSYRIMQLHTNLLKCLFLWPVDTFSPGLNSLLMYGSFCISMCCGIPIISAAGYQFY

VGIEDVNILLEALIGVYDIIGNTVTYVCFLRKQRQIQEIIDDIKDFFQYCGYETVRKIDS

EIMCHTKYFLFYVTVSVILNLAWPMLSVNNCLKSRRSDFYIKHDPCGMPTQNLYPFEASE

GIVFWVLYIIEAIFCYHTCCFFSLATVIVIGFLKHITAQLKCCAYKFEHICDYMGSCKND

ENVIIREFVHLIKYHQRIIKYAEKVFGIFDVMIIVYIGVTSFTLAIIGYQIAIPKTNLED

RIRYTMLLIGWVLLFYSICFYGQQVRDESMKVGEAIYKSEWYQHQTLLGMKTDIMFVMRR

TQKPLDFKATLLGEVSLIVFVAVMKRAYQLFTLLLTVTEDGP

>DponOR33

MKNDFFGFCIPLARFIYIMPDKTPQNVYGWRNKLWAVFMYGLAVFCHLTEIIKLFQIVTA

KYFLLGEFIRNFVITSLHFTSLGKAMFIGGKTGKKAFEKILDFEKHVYKNLGDDIRLIYK

NKVTSIQKVKKYYLIGIILVVIFYVAAPIFREPIHIQDGNQTIRFRQVPLSSWSPFEQYY

WLTFIWTGLTGIYLSIFFVTTDLICYSYVQMINEGMKNLMVLDFLPGSVQLAGMIYQMMT

NLSVIQCILLGQFICSLIARVFIYSNSANNLSQLSKQLAVDWFEIDWTELPKDVTNNLNF

CIMRSQKNLQITVGDLSVITMESFLTILKGTYSYLMLLMTI

>DponOR34

EFICSLIARVFIYAHNAHNLSQISQQLADDWFEIDWNDLPKGVKTNLHICIMRSQKSLCI

TVGDLDVITMKTFLVILKGTYSYLTLLMTI

>DponOR35

MKNDFFGFCIPLAKYIYILPDISTQDRTKPLLHKICAVIIYVLALFCYFSEVVKLYQIVT

GEYFVYDELIRNYTVSYFHFTSLIKAINIKGAISARAFKTIIGFEDNIYNGEDEDIRKVY

KASVTPIQRVRKYYMAGMVMVVICYACAPAFRDPIEIQRENETIRIRQLPVSAWSPVEEY

FWLDFVWKSLVGAYLAYFFVTTDLILYSFIAFGACQVRILQHYIHNFNRYCEEIMHTEGV

PKNESARLLQKQLIAMHQDVISYVNMINGSIKQLMMLEFIPGSVQLAGMLYQLMTNLNAI

QCIFLGQFISCLIARIFIYTNSANDLSQLSQQLAADWFEIDWIELPKDIKMNLNICILRC

QKNLCITVGDLNAIDMTTFLTILKGSYSFLTLLTTI

>DponOR36

MKPIEDQSLFRACKILVLCGGMWRGNIPNWPIAHQKLYKVFLRGAQFAYFFCLPSLVLSL

WVNVDQDNEKAISVLKNITFVVVIFCKMIIIQSRPVTMLIEAASEKEQQAILSEDPQISE

IHRRHVVYTEFVVKSIMLCTFLAGLAYVVGDLYLANEFYKLHPNAAPTDPKPHSIYFWFP

FNPDEYYKIALTYEFVHIVQTVIYNGASHAVVNSAIIFVKVELKILEYEIRHMMSKPNLS

NLTPAQLMKIHIRKHQELIKWVCKFNDSFKYIILLEYSVVSLTLASTLTEILQGIKMVFN

GIFFLLSTLSLFILSWNANEIIVTSVFDLSDALYHFPWYELDKEAQELVLFMMLRCKRSL

NISNGPFGFLTLRGAVSRLKLAYSVVSVLSR

>DponOR37

MFSASKWVIMSSGSWGLEVDSKYRILYKIYVLYIRFIYITSTVAVFAMFLVNLGSNNDKA

IEALSLTLCSVSCIIRLAVCLKQKVVNLLKIVMEDQFNYAVNDPKIKVMLQEYKSYVTFL

CVFVVCYTYSLVILFNIFNGIIEFQSFRKLHPNATEYPQYLVSIWLPFNVQTHFTLALIC

QTVLFLQSCVLNYSSTVLFNTLMIYVVIKLKILQHLFQNFNTYPKNLENFHMELRDVLAI

DNLKHLIRQHQDIISFVKELDKNIKIGVLIEYTITSLMLATISIQVLTGNKVASFSFYGL

ILIYQLFLLSWNAAEIKTQSEKIAGAIYATDWYVYGPGVKQIIHFIIMRCSKGLSLDIGP

FGPNDLGAASARLKLAYSYVSVMGNNK

>DponOR4

EALKVGDSIFKSNWHKHGMALKVKPELIFTLARTQKPLEFKTQMIGSISLMQFMRVMKCA

YSGLTLLLAVTDDE

>DponOR5

MDTLGPALTKVRLRLPSLDIPDSKGTDYPKNLYSAIDRISFLCGQAKLSKNRSWILRFAY

SLYSYTLIIIAIMFIISEIITFRKSLTELTTVLSEIGMMFTHLVGMVKFWILIHKRDEIE

QVKNKLRDVQFEYVGIDDFQPGLKMRKEKLFIIIISTFIFALYNFVGISAHISAASMMYK

YTANGNFLGNTTCETFVPYYFYYPFDVSSPSSCHYLLFYMDLSLDIYASYIATFDSVFVI

LLNLLATQLNILGDALRTIRKRCVKRLQMKVDSSSLYDADNPLLENEMYNELTHCTKHLY

LLLEVGNDIESIFTFLTLLQTIASLLIFASCLFVAARVKPTTPIFYSQLEYFSAVLSQLT

VYCWFGNEITLASSAIPYSIYSSDWFSSSERFKKSMLLTMARLQRPLYVSIGKFTPLALT

TLLSVIKGSFSYFTLFQSAGTD

>DponOR6

MIKQPPYEVYATDFFSVNRWILKCAGLWPPSTPNRVVRRLYQLYTIGVFLFVNLWFTGTE

FVSLFYTYKSQYELIKNVNFFLTHFMGAVKVILWYFYGHLLRDIMNALESPQLHYEGYAD

FSPHRISHLHRAIGRRYSLLFLCLAHATLISSYIPPLIAVAEYLNQPQGGLQKLPSRLPY

FCWMPFSYDTPGKYLLAVAYQAGPMFSYAYSVVGMDALFMNILNCIAENMVLIQGAFKTV

RERSTLHYCVGALAPPCDAIREHPLVLRQMDLETKKIIKHLQITLRACKHLEGIYHIITL

SQVTATLFILCTSLYLISTASPFSKQFFAELVYMMAMLFELFLYCWFGNEVTLKYEQLPM

HIWESQWLATDDCFKKQMIFTMLRTNRPVYFTAGKLARLTLPTFMSILKTSYSIFALIKN

FSK

>DponOR7

MANLKQALQIYDICSFLEGEHIRLGIGGFYPRRIKRTFIVNLVTVFAYIITIAQMAVVIN

FVLSITDIVTITEVLLFSMTQVGFVNKLVNFHRNSRKVATLDELISQEIFTRVTVAEMDI

MKTSFQRCQKVLNIFLLSCFGVTLLYGVVPAVNGIMTGTKMYPFPGKFPFNPDDYFVLIY

GGEVATVAVSAWNNGAMDCLFTKHTVIATTLFRILRKKIKDLHYNTNEGERPLENRIKHC

VRYYNEIIKYVSAIENIFAYGILVQFMCSAIVICLTGFQLLVVASESGQSGLLVVYLFCM

MFQLVLYCWYGHMLMEESNRITEACYAINWHEMKIGQQKMLITIMERAKKPIALKALGIF

RLNLSTLMTILRSSYSYFAVLQQIYRNDKIMALVTN

>DponORco

MINKFKVVGLVADLMPNIRLIQASGHFMFNYYADNSGSLHILRLGYCCMHLFFVLVQYGC

IFGNLVKEKDNVSHLAANTITILFFTHCLSKFIYFAARSKLFYRTLGIWNQANSHPIFLE

SSNRYHALALKKMRSLLYIILFGTIFSASAWTAITFVGESVHFIKDPDNDNETITEEIPR

LLIKSWYPFDAMSGMTYYVALVFQIYYVFFSLFQANLLDNLFCSWLIFACEQLQHLKEIM

KPLMELSATLDTFVPKSADLFKSPGSATSQDHLIENDFNAKNDDLKGVYNIRQELGNLNF

RSGALQTFGQGGGGVGPNGLTKKQELMVRSAIKYWVERHKHVVRLVTAIGDAYGVALLLH

MLTATVMLTLLAYEATKIDGLNTYAATTLGYLLYSLAQVFHFCIFGNRLIEESSSVMEAA

YSCHWYDGSEEAKTFVQIVCQQCQKSLFISGAKFFTISLDLFASVLGATVTYFMVLVQLK

>ItypGR2

LMMTNIIISVYGFTSEVVDHGVKFTFKEMGLLVDAVYCLVLLYIFCDRSHKASENXAEXV

QWTLMEINLRQVDEATVREVQFF

>ItypGR3

MVETVHPITKVAPAPPPRYSNGDLMEAFDDSGELNSRIFSCLKPAYATLRLFGLMPVTQS

GPVFHVTAKWIIYSFMLLCSLAGFLGYLKYFNIAITRNAEGRFEEAVIDYLFTVYLLPVA

LNVIAMYEASKQAGVLTQIVAFERIYTRTFRKRLTLDMGSKQLILISVLLILGCVVMVVT

HFTMANFIVYQVVPYCYVNIVTYIIGGSWYIYCDVIGKVATSIAEEFQFALKNAEHSSRV

ADYRSLWMMLSKIIRNVGNSFGYQLTFLCLYLFFVITLTVYGLLSQIQEGMGIKDIGLTI

TGVSAVTMLYLICDEAHYASSCVRTYFQKKILLMELSLLNEEAQQEINMFLRATEMNPTD

MCLCGFFDVNRNLFKSLLATMVTYLVVLLQFQISIPAGNDNLTNSTLNS

>ItypGR4

MQFVNIVELNALEFHLEGVLLNLIFLYMAKIWAAFVREWSLIEKSMKYYEGPKNAKIKIV

VAMVSIMGIALFEHALVTSQIAWTSIRNNPLSFFNASREYFVHHEFVEVFHFVPYSIWTG

LFFKLLIIQKTFIWSFIDVFISTVSICFHCKMVQISRKVARLSAYEEKNCMIWRSTREDY

TKLSKLCQIVNSKLRWLIILSFFNNVYHILSQLFNSLKPNDDAMHKIYFCISFTLLILRM

TTVCVYAGSICDEQDRLITVLTTAPSGVYNIEVERFIMHLDTFEMALSGSNFFKITKGLL

LKISSAIVTLRISVNSV

>ItypGR5

MSVLFWKGIREDYDRLASFXXELDSHISVLVLLSYFLNIFFLLIQLYHGLE

>ItypGR6

MSYNGDVIKYPREITVFAAVCAVVFCVLGIVGNSITILALFRCPKLKSHATTAFVISLCV

SDLLFCGFNLPLTAARYIAEEWIFGDTLCQLFPVFFYGNVALSLLNMVAITLNRYVLITL

YDYYTKFYSKLSICLQLFCTWLIAFLIMMPPLTGIWGQLGLDPSTFSCTILEKDGRSPKK

TLFLFGIGFPCLVIILAYSCIYWTVRNSKLRLRSHEPLPNQRTSKRDRDDRRLTRLMALI

FICFVLCFLPLMLVNVFDDKVSYPTLHVLASIMAWASSVINPFIYAATNKQYRSAYKRLL

ALVRSSIGPESMHSNSLRSKQEKFQGVSYKAK

>ItypOR1

MWITMFCTKNRHVLISLVQGLSDFTGFPPNFDKFMQQLNFYSKIHLCYLTGGSLMYFVLF

APLHKRNCDELKREKNLTETCSLLLPLNAPFVEYQSFGKFPTLQLLNIIIFLSLMYMYMC

AGTIVWLNVELVEHIRIRIRHLKHMILRALKSNDKQFRREKFRKAVRYHEYICSMSRLAD

EFFGTELFLHVVLTGAILGISAYLIGDGSLETVMIFVGWLNAIIMGSVAGQRLINESLGI

SDIIYEVDWYNFETALKKDILFFLVYAARNLCLLGLGMW

>ItypOR11

MDVIKDILRNFKENSLLQAKFERHLKVAIKTKKRGDFLFLYGTFLGFGTQLFWSIYPFSQ

NKKLLPVNGWYPYNPMNSPSYELTSFFQIFASAFNISHTMNIDSFTINLMMQTGMQCDFL

QLTLKNIMQFHTVDGILCQSLQQNTEPLEDILTPNLKTCIRHYIEIKRIARKLEDIYRTS

MAVVFLGGAFIFCAIFYQMLHSQRDPTEIFYLLFFLFSMLTEQFIFCWFGNEITFKSGQI

HGALYTIPWVDCSVKFRKML

>ItypOR12

PFNPDDYYWPIFFGEYFATGLSALCNGCVDRLFAKHVSIATGLLKILRNRIKQIMDDDDH

NVIEAKIKHCVLYYNEVIKYGKVIERQFSFGILFQFLVSCLVICLTEFQFLVATSSGSFG

LLFAYLACMITQISIYCWYGHQLMEESDSVSMEFYNLNWLDMSIKNQKTMLTSKERAKNS

ITLKASGVFQLNLTTLMTILRASYSCFAVLHQIYTK

>ItypOR13

MSNYQRYNIQTFLKEERLFLGITGFVSGNLKLHLPTGVTYVLTTMQVLASIYYGLSTTDL

AEITAAFMITLSHINTLNKLFGLHLKSLTQLDRILVKQIFALADDRELTILKRTLTACHN

MLTMYLVTVLGSILLYGATPLVANYTTMERNYPTLAKFPFNPDDYYWAVFAGEFFIVALS

ALSNGCMDRLFAKHVAIATGLLKILRHKIKQIMDVDDQKVIEAKMKHCVLYYNEVMGYAN

QIENQFSFGIFIQFLCSCIVICLIEFQVLLATSSETIGLLLTYLTCMITQVTIYCWYGHQ

LMEESNSISMEFYDLNWIEMSVKNRKTMLTSKERAKYPIVLKASGVFPLNLATLMKILRT

SYSYFAVLHQVYTK

>ItypOR14

MEYSFHYHDGFRSHMFLYVMLQGKIAVIILIDYVCSINHMKNCSETTMKHLKIIIKKHLE

IIRYNQFVLKSNQYFYLLYLFNEVFFGLLPMFEYFSPNRSIIAVSSATVYFFCLVYAVND

AAEEYITLVEYFRTEIFNLKWYEWDVSCRKVYSILITYLNEPMKVDFVVVNINRALFCQI

LKLCIQYLISFIQPTELVDSSFCNSALYVLSLIISHYW

>ItypOR15

MLNNLVSSQLLLQEFCMTCVVCCSIYRSTSPGVSSTEMGYVSTMGIIAAIEMLTVSWFNQ

CFMLEVFELLNRIYELDWLNYNTKMRKMLVFLMQRVQRPYHFTMGLGFPLNLGVFFTMIK

TSYSLYALLTNTGSKVST

>ItypOR16

MYNIPENERKNYFLKFSRVTMLMLGIWPVRRGGDLLEKLYESYFLTTFLYYIAFNLSGLA

LAIRTWSNNYLTTASSMGIVIEYMSNAYKVWLFKTSVFKSLIKEIQDREREIFEGPDEAF

KEIYIRNAESNKKVVLFYTIMGTSGISLYFITPLVSNVLMPLGYNNVTGVYEHYFIVFNW

FPFDPNRYYWAAYLIQFTGCLIGYSYIVHCGAFYISILNFIRTQLKILRHVIVNMSEYSL

LYKNTYKLTEEQSQFVLLRAVVLEHQRIISFVTKTNHTIQLFTLINFVISSFQLALLVYQ

IFQVAILQQVTVLSYFITLSTQLFLTYYAAHMILFESSNIASSIFEGNWDTYPPQTLKLL

QMICMRAQKPLAMTIGPMAAVKVTALFQIFKALYSYICLIIKF

>ItypOR18

MYTLSKNKPFYYALVLLRAFFWYPSSPKCSVTFILCSLVLRLLSTLAALGTLAHLVLNLT

GETKAEISEDIGDLTGFVGCMSACLNFLWHRSRWSSFINRLLTFKQFGTPPGYVKVVRRG

NLITLACILYTIPGMLWYSHLTHLDIPRCEALNREFGMKEACGMVNPTWIPPGYDRRNGW

RFWVLYVLQCTGIFVYLPSTFVISNIPLEAVGVIVTRIBHLKYHLKRCGSDLGRLYHCVK

YHQDIIEVSKELSDLVQATLGTLLLTGAVVIGSLGSQVIKASTPKAVTFILGYVTTIFMV

CHAGQKLINQSLTLADQVYWMEWYEQQPKIRKDLRFVLARCQKPLGLVGPPSMGXAGYSL

FLIMLKTSYSYLTLLNEVIS

>ItypOR19

MLIFGNIISANVIVLFGSSLINGDYFLVTSSFPFALAIIVVNGSTLSFAVNHKQWSNLFK

SLTDCQKFGKPPNYDLLKKNGDRKGMICMTCYISTCTLFAIIEAVEEQRCLKNVSKHEIC

GFIIPVWWPTNYHPSTFLKTMVQVYEVGTIIILSNFTIIATLQYQVCEYIAAKAAHLGLN

FNAIDPNSDAKTQFEQFKLFVDYHQHIISLCAEFDSSCKRTVGHVTFTTAAISALFSYHG

MQGNYKLLAFLALYILNLGFMCHTGQNLEDAMLGISNSIYSSKWYELNIRVRQRIPFLLA

RTQKRIGLDAVPIGYLNYALFMTVLKTTCTYLNLLNHTI

>ItypOR2

MKVLQRETEITFFKFNIWVLKTCLLWPEDLNYKYDKKRFIKDLTMVASLMPCFLPILADF

LQQLYEEVPDLTEAVENMIALNCLIGMFYMVICFVRNRRMIIQLMIDIRTFNKYGNDSIT

QEVDNKANLFSKMFMFYGILGNFVYMAMPQIRVSKCHLNRTEDMIEKGVPCGLVVRSYFP

FKFDYSPVFEIVFVHQIYTCTMVSVVVLVLTMLFCGFLMHIVNQLKHLRVLIARLKNVPP

EKFERKLIFVVRYHVAIIQYSQNTAGAFSTMLLFYITLTSVVLSVLCFEILMVDAFADSV

RFTLHLLGWLIILLSICYNAQLVLDQSQEVANDVYSLDWVSILSVDVQKKSKXVIMRSQK

ALVMEAGGMGVVSLSAFLKVLSSAYSFFTLLLKFK

>ItypOR3

MPTRNLYPFDASQGWSFWILFVIEAIFCYHTCCVFTLATVTLIGFLKHILAQLRYCGHEF

ETIFDGVNEESGGKHLTLQHFIRVVKYHQEILRYTEKVFSTFNVMIVVYTGVTSFILAIT

GFQITSPETGGEDKIRYTMLIIGWALLFYWICYYGQQIQDEASQIADAIYNSKWYENTNT

VVLVRRDIIIIYLRTKRVLDFKVQFLGAVNMEVFVAVMRRAYQIFTLLLSVT

>ItypOR33

MLIDIVPGSIQIACSLFQMVKNLNLVQCIILCEFTLTLIFRIFMFTSTIHNMADLSQKIG

SAWYNMDWIELPRDVRNNLMFCIMRSQRPLWITLGDIDTISMGSFLAVLKGAYSYLMVLL

TV

>ItypOR34

MKFRELIHNDFLGICIQLGYYFCIIPEKAVTTDKIESRNYFFYTCIVRTLILYCHICQWV

KMYQIITADIFIFDELVRNCAITSIHFQSFVKTSIFRQNYQLFENVIDFENVLYKNNDQK

VLLIYRDTLQAIKNSRLVYVFGILIVIVFYIAAPLFRGPYYVEMGNETVTIIQLPLSAWS

PTNNYFSNFAVTGAMGAYLAMVFVQTDLLYYCFLYFSICQLNILEHYIVHFHHYSNELVN

DHKCSHVMALSLTQKIYIKYHQNIIKNVKQLNDALKNSLLIDLVPSSIQFANQFYIIATN

LNIMQCVCIGFFTIMLMSRVMAYCYLANQISVQSQKIGSAWFQMDWSDFPNEMKKMISFC

IMRAQKPLVITLGNFGNITLMTFVGILQASYSYVMLFITL

>ItypOR35

MDNQPKFLLFTKRLAFALGIFPSKLTFQDNNWRNQCYTLYTKFCLGLFNLYLLTSFVQLF

VIMSSNPIDFTELSKNLIITPLFTVTVIRQICMSQPGFIKLIQHLISHEMYLDTXTDKYV

AYIIQSSNIVFCSTFDTVGEILLTTILVYPTVRLKILKHVFENFQKYERNFTDPHSADRL

MKVCLKIHTDIIRYVEQFNSVMGTCMFLDFIQSSVHMACVLAEILTGDVSLIELVSTSAY

LVILNFRLFILFTTTANEVIVLSQGIGVAILNTNWYKKNSSV

>ItypOR37

TSSLVVMQTHTTHTGAVPTSIKESGRISWAIYEGNWFLHGPEAGYLTGLIILRSRKVLSL

NIGPFGAIGLDAAMDRVKLAYSYLAVLR

>ItypOR38

XGNRVELFTPYFIMLIFQLFLLSYNAEQIKTESGRISWAIYESNWFLHGPEAGYLTGLIT

LRSRKV

>ItypOR4

MDKQSQILKFHVKVLKFLMIWPFDGLDPNQNYYLMRGCFAYACFCSIPVFSGAAFQFCVG

IDNVKVLLEVLVGVGNITGYNIAYVCFLKNQEKIQFLIRDFQEFVQFSGPEIIQNTEEKT

TRYTKYLLGYASIGLVITFSWQMLSTESCVAQRGGDYYVRHDPCWLPVRNWYPFDASQPK

LFWIVFPIEAIYSIHICLFFSLATSTIIGFLMQITSQLQYCSNRFEHVFDEVDLKPFQQI

KPDFLFLIKYHKKILDYSKKLFNVFDALIVVYISLTSFIMAIICYQIVDPKISAQDRIKY

AILLIAWCLLVYLICYYGQKVQDEALKIGQSIFKSHWYGGTTAVELKPYILFTLARTQIP

LEFKAQLFGTISLLQFMKVMKWSYSGLTLLLAVTDED

>ItypOR42

QVRDKHQQIIQWVNDFNNNFRFIILFEYSMISLTLATILIDIFTAWDANQISYESSISLS

DALYACSWYEFDKNPRKISFYL

>ItypOR43

MDAALALMKNITFVAVVVFKTVVVQSDAIVKLVKAASVEEEKIRNLTDQAIRKIYKSNVD

YCNRVTKVIITYLYGSGTIYVLDGLYKSYTYYENHPNVKPEDPKPHTVLFWFPFDHNRYY

KIAIAYESFHIFQTLNYNGVAQSVVSSVMVFLKIELKVLQHHIRAIQGGSRDYQKVLIKC

AIKHQQIIQWVNDFNNNFRFIILFEYSMISLTLATILIDILQGTKICFNATFFALNFTQL

FVLAWNANQISDESSISISDALYACSWYEFDKTTQDFVLFMTLRCKKPLNISNGPFGYIN

MDAALSRVKLAYTVVSVLSTSTK

>ItypOR5

MYREVTHCNQHLNLLIEVRNDIEHVFSLITLIQTSASLLICASCLFVAAQVQGNSPTFFS

QLEYTAAILSQISLYCWFGDKITISSSEIPMALYKSDWLSCSQRFKKSMLMAMTRMRKPL

YVSIGKFTPLALNTLLAVLKGSFSYFTLFQ

>ItypOR6

RYATDFFFGKPMDVKKGWFTEALKQKQGSKYSLMFLMLAHATLTSSYVFSTITTIQHMKG

NSTVALPDRLPYYSWMPFSYDTGPKYLLAIGRIKQVPMFSYAYSIVGMDSLFMNIMNCIA

ANVTIIQGAFKTIRERALPGQPNNVPHESKADMDVLRVELRKIVNHLQTIFKACDKLENV

HRMVTLCQVTATLFILCTCLYLVSIAPPLSKQFLVEFVYMLAMSFQLYLYCWFGNEVTIK

FQELPRYIWASSWLATDTQFKKALLFTIMRTKRPVFLTAGKFSRLILPTFMSILKTSYSI

FALIRNTSK

>ItypOR7

VFFDQLRVCYFNVWGGTLNGQRLQWKEKLSFSRKFPFNPDDYYVAIFLGEVIAVAVSAWN

NGSMDCLFAKHAVIATTLCKILRKRISTMLQITEDDRTIEDKLKHCVIYYDEIISYVTKI

QRIYSYGVLIQFLCSAIVICLTGFQLLVVSSKNGKIGLLLVYLTCMTIQLVLYCWYGHIL

TEESNGITMACYVVDWHELKVTNQKMLIMLMERAKKPLGLQAMGVFRLNLFTLMKILRSS

YSYFAVLQQIYKN

>TcasGR19

MTSKARTFTIKCPVKKRKCTVSNVSLHSLKNKRSGASLQVGILDSLVFKCYNLRRIYYFY

CVSITFNVHLLFLLCSGYFTVHLLFYCPFIIFTVHFLLCTYYFYYAFIILLCVYYFYYAF

IIFTVHLLFLLCIYYFVVPLFFLLCIYYFYCAFIIFTVHLLFLLCIYHFFCAFIIFTMHL

LFCCAFIFFNMHLLFLLCLDYFTLHLLFLPCIYYFYSAFIIFTIHLLFYCVLIILLCIYY

FYYAFILFTVHLLLLVCIYYFYYMHLLFCCAFIIFTMHLLFLLCIYYFYCALIILLCIYY

FCRAFIIFPTHFYCAFSLYPLKSTFYLNVVRPFPERSMGITPMIWNSTRRLFVVW

>TcasGR1

MQHEVVKKFLHSVRIVFVQSEIFCLVNFNHRESYFRLSKAKSFCTFVAALVYCSVTFFAL

SELLTDLATSILLKVSSLLIGFCASIYVGTVWINTSINGTKFIEFINKLIEFDVKLQNVS

LIINYENQRTRSRIHLFVRYVFVTSYLLFDYFVQRNQEYKYYQILSHLSGIFFTVFNVAQ

CYLTTELVLMLQTRFVILNKQLTKITVKNFATKTQSAVLGKICTLHHHLSKLVTRFNEIF

GLGLLLMFAVSFLIITQVIFVICVLVQSEKIVWLHLVYISFLGIICAADVFYICHVCYAT

IQEVRAKIYFFYLVKFYKVSKSGELIHKIETNEHEIIDKIEMFSLQILNERAEFNAAGFF

PIDYTLVFSVSFG

>TcasGR20

MSAMSTVIVSGEMFDLFSPVLYLSRIFCLQPLKWIKTDYSYVISKSGPYMAYSILTSLFL

ITASIYGLTQVYSMEAVYLIRLSSNTDRFVTFSDVVVVLIPCIIGVPIASHNIEKTIKYF

SFLRQFDCCLQKQPPKTWKSFFVPIITIVFTATILLFDAVMWLTLIARNQSIFILSLPYY

VCYCFTMIIEIMFWQFVNSIKIRLILLNERLEQIGGEDFTIVRIDRKKINITNEQVRDLI

IAYQKLTDAANLVNDSFGLLILIVISGCLVHLLATPYALYAIIFTTGNTMFIITQSIWMT

GHVLRLLLIVEPCHGCILVAKTTTQLVCKLLCLDLDKEVKKSLEFFMTYLAECQIRFTAY

GFTKLHRGLLTTITGAVTTYLVILFQFN

>TcasGR2

MEISDLAQLYGNELHIKQISKWLRGSARAQEIQKRSELDSKDGHVIDEHDQFFRDHKLLL

VLFRVLGVMPIQRGEIGRITFGWTSIPMLYAYVFYVVTTVLVVLVGYERFDILLNKSKKF

DEYIYSIIFIIYLIPHFFIPFVGWGVAYEVCDYKNSWGGFQLHYYKITGKNLQFPLLSTL

IIIISLGCLILAVVFLLTLSALLEGFTLYHTTAYLHIITMINMNCALWYINCRAVGNAST

ALAESFQNDVDRNCSAYIIAHYRVLWLSLSDLLQKMGNAYARTYSTYSLFMMANITVAVY

GFTSEIVDHGIRFSFKEIGLLVDSTYCLFLLFVFCDCSHQASLNIARRVQVTLLQVNLSQ

VDPATRKEIDIFLVAIQMNPPKVSLKGYTVVNRELVTASVATIAIYLIVLLQFKISLLNM

RG

>TcasGR3

MNNKLNMVLVKSMLQRIKMEEKLVVEKFLNSLQIYLQHNQIFGFVTFTCTRSNFRSSKLL

ILYNIILQVLFVSFVSYWLYLVLEADDMLPIYKNTYLIILFADFAYLETTWICTLLKKDK

LLELFKRLIHFDTKCQENSTVIDYKRHKKRLLCYLLARYVALALVILFSEILVIVSEQEW

SFSTGLLVMIFNSALSYKASEIVVMLRSRFAILNKQIRFLNQYLRLKPEGRISNRRVFIS

FSKICYLHQHLSKSVKLFNEVFGVSLLVLFGNSFLSIVLALFRTAAELQASQIKWTRIAY

MALASVPFIFDSIHLCDVCYSTIGTVSWCELNFDWSSQVSKAGELIHQIQTEDHDIIDEI

EMFSLQIANEQVEFNAAGFFPINYTLVFSVRSVQVERI

>TcasGR4

MVRPELDLNSLKCITKVLTILGLLSCSFPKKKLVYCIIFGAIATLTCIEGLRDCPRKYST

PLAKITTILQRYCSVLLVFLTYFFNILFRKKLLNAVKILSKIDETLNSKPLKPVKIRTKV

YLVYCSSALVLAAMTTFHLIYEAHGFSYKCGIQYHICNTVISATVCFMMLLMLEIWRRFT

ILNNYLTIVLGETWTSLSVYHLVEISEIHFDLCQAADDLNRYFEVQVLTIFGVSFYFFIS

TFFYFFTTGNIIATYDKQFYIHNALIVRALFLFFQLWATVYTSSQVTREVQSRSNRMTPL

KLQPQNFSI

>TcasOR1

MMKFKVTGLVADLMPNIRLIQASGHFMLNYHADNSGALHTLRLGYCCMHLVFVLVQTFSC

NFVNLVLERGDVNDLAANTITVLFFTHCVTKFVYFAVRSKLFYRTLGIWNQPNSHPLFVE

SNNRYHGIALKKMRRLLYIIIIWTSFSAIAWTGITFVGDSVHNIKDPENENLTITEPIPR

LLVKAWYPWDAMSGMPYYITLVFQVYYVFFSLAHANLLDSLFCSWLIFACEQLQHLKEIM

KPLMELSATLDTYVPKSADLFRAPSATSQDQLIENGTNPAKKNEDLKGVYSTRQELGGHF

RGGALQNFGSGGVGPNGLTKKQELMVRSAIKYWVERHKHVVRLVTAIGDAYGVALLLHML

TSTIMLTLLAYQATKITGVDKYAATVLGYLLFALAQVFHFCIFGNRLIEESSSVMEAAYS

CHWYDGSEEAKTFVQIVCQQCQKAMSISGAKFFTISLDLFASVLGAVVTYFMVLVQLK

>TcasOR102

MQNQSKPCQLDMMDETYLQFFVKSFTYLNMLPEKTTFCTTIQQYYVSVIITITTFPILAD

LVSQFYEESISFTSVNENFVALSALFAVIYVSVCFINRKHKIRALIADLALFETFSSKAV

ITETDKSVKFYTKLFIVYGIVGNLCYGLLPILGYKKCHESKSVHMTRYGIPCGLVVRFLF

PFKFDYSPLAELVALYEILVCILGTSVVIVVTTLICGVLIHITVQLQCLRKIILDLSQVN

DLEILEHKMKFCVKYHTAILDYGIRTDLAFNQMMLLHITWTGFIISVLGFEISTTDDYVE

AFRFFMHLLGWLGMLFVVCYYGQKILDESLAIADAVYTFLWYKKSVIVQRYVLLILLRSQ

KPLTLRACGVKVMSLATFLGVLYSAYSYFTLLLKLKPZ

>TcasOR113

MLDAWERLTFYPFKWISLGGLHPQNDRLVAKFLFLYNFAGFGTILGLAITQIYLSYENIY

YTIDSILTIVLYLHIASKYVNLHLHKDTLAMLIQERSKFWPIDTFEMTVRQKCVRILTKS

LTIIKSYLGYSLLVVISFLIQPIITGQLPVFMYVPRGTYYIFFVIFMTITPGIMSSIWGV

DTLFFSITTPVSIQFKLLAHKFETIDLKMDSKRVRHEFRKLVDYHNFLINYCQNINRMSS

GIFLTQYLVAIATSCMQLFITSQPEFGLLNKIKCLTYFIMQIIETGIYCFTAQLISESSE

NVGNAVYKAPWYDFNCGTRRDIALVIVRSQKKVVFNGLGLVWIKMETFTKIFKTALSFYT

YLNTMVYQNZ

>TcasOR115

MSNKQLDPTYYILKIFKAAGVHPDVKVTPFLVFFFWMNCTIVSAVIVLATIGAIFGALDN

DINTVVECLQSTFIYIHILGKHFVLFYSKPILSHLLAQRTHFLQLETFDLSTIEQFQQLL

KKTSKFVNTFMFCTSCVVFSFYMQPYLTHGDLPVSVYVPDGWYYYIHFGFWPLAPCIVAS

IYGSDALFCAISVPVIVQFRLLARKIQNWKIENTKLNNQKSRKIFKKNLKELVDHQNFLF

EYCNEMNKFNNGIFLNQFLLSVGIICVQLFVVSQKGFKLPNKIKCVGYSFMEIIETAIFC

FNAELISDASEDVGNAAYDSLWYESDDPEVRHAITLIIARSQNRIVFSGFGFVWINLKTF

TQIFKTALSFYSYLHNVVLNZ

>TcasOR116

MPETLSFYPELMLKSAGLHPYTKLKIVKFFYHHYLNLFFFIFLLFLAILEVGVSVKCDIY

RAIEALSSVLFMTLTLFRYVVNYRNKPSLAWLLEKRSNFWLLEHFEGQIRTDCAKIMHTS

SNFIRNYKNYAIVLAAVFYVQPFIFHELQMKIYVPEGWFYYLYLVYWYMTPPLFVSVYGV

TSMFCAICIPVTIQFKLLAHRIQNLDFKSEKFQRDLKHLVDYHNFLIDYCTRINRYSNGV

LLFEFFITISVCCILIFIAANDYPFVDKIKYAGFIVSQFLDTAIFCYNCELISDASENVG

KAAYDSLWYESESKIRRSLILIIVRAQKKVTFSGYGLVRINMMTFTQVFKATLSFTSYLN

TVTVDEKMLNNZ

>TcasOR117

MDKNLDPDDLSAYPLKFLWYGRLHPGLNPWWTKILVPVNVSVAFLYLVLAIKGIFSSYNH

DTFFTAECVQTCILVVHAIGKFSNFLVHKNSLLRLVAKKSQFWKLESFDGDLYNECVWIS

TFVKKITRFYYFLNLFVLISFDLQPFTTGYLPTGCYVPEGWFNFLTGLLWYLSCAVLFGL

PGTDGFFCSLATSLIIQFKLLGYKFKNTKLYKNEPDITLWNNLKQLVDYHNYLLSYSKEL

DATFKTIFLLQFMISIGSASVSVFIFMQPGDWSNRIKFLLYFVATMVQTAFYCIPLEFVV

SSAKQIGDFVYESNWYQVKDIKFEKCFTLILARTQKNVVFSAYGLIWINLGTFLVICKTV

FSFYTYLNSVNKITSZ

>TcasOR120

MAKKFLLDISADSLRLLWLGQMHPLSPFRRFVTFLILNLAACWLMIALAIKGITISYKSD

IFFVAECLQTCNLMFHGVGKFLNLYFQKNNLKSLLENRSKFWQLDDFRSEKLYSQMQGIT

FVIKKVLRYYYLLVLCVVFLFDLQPFATGLLPTGCYLPEGWFKGLTLTLWFLSVSFFLNI

QGTNGFFYSQSVSLIVQFKLLSHRFKTTQFDKKELKELVDYHNFLTSYCKQLNQAFAAIF

LLQFFTSITSASLSIFIFMQPGAWTNRIKFILYYSYTLVETSFYCIPAEILVNAASEIGN

SVYDLDWHKIRINRVKKCIVIILARTQKTMVFTGYGLVNMNLQTFVVYVKTVFSFYTYLN

SVRKIZ

>TcasOR123

MAKKFRSDDISADPLRLLWLGQMHPFSPFRRSVAFLVMNVSACWLLAALAIKGIITSYKN

DIFFVAECLQTCNLMFHGIGKFVNLFLHRDNLKKLLKNRSKFWKIDDFQSEEIYQELSEI

TSTVKKGLRYYYCGVVVVMLLFDLQPFATGSLPSGCYVPEGWFKSLTVMTWLLSLSFLNG

VQGMDGFFCSISISIVIQFKMLTHRFKNMRLFHNESERKMWKELKELVDYHNFLTRYCKL

LNTIFASIFLLQFLVSIISASVSIFIFMQPGAWSNRIKFILYYLAVVAETSFYCVPAEII

VNSASEIGYAVSELDWYKIRINQIKKCFVIILARTQRTMVFTGYGLVNMNLQTFVVYVKT

VFSFYTYINSVRKIEKZ

>TcasOR126FIX

MYNNKDSLLYDPLRFFGFIGFHPIFNSLILKISFYSTTTLGLFIYTMAIIGIVKPEETNS

FFTLECLQTCILLSHTIGKQVNYYMNSNKIVKFLQMTTEFWEFETFQGTIHPESNFLFHT

VRKMIRYYFLVTTFGFIFFLINPPVCYVPEGWELFLRIVRALTFWSYYTSTMATDAFFVA

CGTLLLIQFKLLGHKFKNLDTQSQEKWNNLRQNVKHQIFLHSCKLLNQIFAVVFLIQFLN

SIAALAISIFIFSKPGSWNNRFKTLFYLVVVLFENAFYCVPAELVSAEALKICDQLFASK

WYESNVAQFRKSLVIVLCCTQKVIKFSSFGLVEMNLQTFVLISKTALSFYAFLNQLKRZ

>TcasOR127

MSPRTLEMIADLPKDIITDSLKMLRLGRHHPTGSIWWTIFFIPVNTFFCSLLIILSIVGI

VRYHEDDVFLAVDCLGTCTLMLHAISKQIFLHAQKNAINVLLKMKSQFWNLDDFDGEISK

ECEMILTSGKIAVRIYFSFTCAAATFYFLQPFTAHHLPSDCYVPEGWFPFLAISYMYLIP

TLVPSVVGLDALFWALGLSLAVQFKLLAQKFKLLGTCHENETAILWNQLKELINYHRFLI

DFCKKLNKLFSFIFFVQSFITITSASVAVFIVMQPGNLSTRVKCLLTFVSYILEMAFYCL

PAEMSVNAAIDVADSVYNSKWFRIKSTEFKKCLILIIGRAQIPFTFSGFGLIHINIRMFQ

LVCKTTFTFYTYLNTVQNRQZ

>TcasOR155

MTEPLYNPFAYTILLLKILGMWKYKTESNFYKCYKHLMSASILLTCVFSLLYAYKNYKDP

EAIFFIAYLPATLTVPMKFVMFPLNLAKIKRLLDILGPERAIIRTQKQKVILENSLQLSR

QIYRIFTACFVSASAGIVATPLSMRRQILLFLEWLPVDYSKDCVHYGVFVGLLYFIFHLI

LVNAAGDFFFYICAIQIEGRFDLINDTLLNLEEISAQNEDKYERMHRIVIECVQQYNIII

ECSKLLVDCFKEILINQLICSCLSLMFTLYQLNAAELFSLNFFRVVFIAMALGSEIFLFC

FFGNRLIVKGEILYYSSFESGWYKAPLKIQRDVLIFMQQLQKPVMINVGNIFPLNYETFK

GIMQKSWSFFVALKNTQELRTRNZ

>TcasOR160

MSGKTKRITTKTIHLSNPYSSFKKVFSDFAYSKIMIFYTIATLAFHMLSLFLQIYYVATN

YSVELICRYGPMMCLAIYVVTAKVVGVFYYKTFTMLENQCLFVLWKTCNSSPTTQRLILN

KSLKMNQKLHLALMSYFLLAIVMLPTWGDLNELFIFSQVYERYFKFWAPVLYYFYISTFL

WCSYYSFHLPGCILYLTLLLDVQIKLINDKITEIDQNFSQNEISETLRLCISHHIALKRW

MSTLAKMVNSVMPVFVLLGALSTVAVSFFVLNTLQNTTMILKIRLAILTVCNFVIVSTFA

ELGQIFSDQNNSLFEHLIDCPWYLWNVKNRKILLMFMANCMKPKTFSWGGITLDYSFAIS

ILKTSFSYALILFKLRGETIRNZ

>TcasOR165

MSDNTKKATTKSLDLTNPYSSLKKVFINFAYSKIMIVYTSATLIFHILSLMLEIYYLATN

FSVELICRYGCMMCLITYMVTAKFFGMLFSNQFKFLEEQCLLDFWKAFNSGPTTQRLILK

ESSKMNRKIHLALTFYVILAIIMLPIWEDVNDFFMFSQVYENYFANWAPVLYYFYISTFV

WCSYYSFHFAGVIMYLTLLLDLQFRLINDKITEIDQNSTQNEICGTLRLCISHHIALKRW

MNKLANSVDTAMPVFILLGALSTIAVSFFVLNTLQSTSVILKIRLATITVCNLIVVATFA

ELGQIFSDQNNSLLEHLMDSPWYLWDVENRKTLLMFMANCMKPKTFSWGGITLDYSFALS

IFKTSFSYALVLYQLRGNTFZ

>TcasOR173

MSNVTFDEPFMFFKKVFFDFGYCTSIRFYHLFCFTFHICCQIIENYFFLTEYLSADFVTR

YGCPMIVIGYTIVCEFFLMKWEEPIKELLDERETIFWEIDSNSKPQILKYSSKVNRIYKF

FLFWVVILAIFLLPFWGDLDETFFIIRIQKIYFGKWSTLFYTLYVSTLPFMVYSGIRFPI

VTLYLIMQSHLQILILSQKIGQISQNNNHMDDVSKFHDVGYQKKIRTSLHVCMCRHVTLK

QWISKILQIVQKAIPVYFSLAIIVLVTVMFCILYNVESASTTTIFKIRLVLVGICGAVVL

FTFSETGQLLSDDTSQVFDTLAASPWHEWDPKNRKTLLMFLLNSLKPVKIYWGGFALDYQ

LGGSVIKTTFSYALVLFNLRKDZ

>TcasOR175

MRNFQDSDDPFIFIRKVFVGFGCSTIIMYYSRLIFIFHTLSLLLESYHVITNFSLDIITQ

YGSAMSLMLYSITSQFLLICEQNLITEVVEECKSFFWTMDFLSFIKQTQILKDMTKIKRK

MYLSWIWFVVFGIALLPVWGDYNEMFLFPFIYQTYFGNWSPLFYYFHASSFPFLAYIAIR

IPAFILYLTLALHFQTLLLNQKILQIPQNKSGNQEDIFRNLCSCISHHVALKKFVTKTQQ

SIQKMIPVYFVLAILCLVAVMYSCLNSLAMSTSNHFKVRGFFGGVCGVVVLYTFAEAGQL

QADTTGEVFNTLMQCSWYNWNNRNQKILLLFMVNSLKPSYIDWGGVIVGYSFGSSVIKTC

YSYALVLYKLKISKEQNVTFZ

>TcasOR198

MPNVTNKRQKRLFSKTRTKSEDPFVMIKDVFVDGGYHPVTKMLNYICLVIHSCSLLLELN

YFVHNYHFDLMMKYCCAMSLMGYIIATMLFAIFQEHSAIDLTKDILSLFWPIDYCGPRVK

EEIVKKATKINRIHYIVLLFAGALGITMFPIWGDQKEWFLCVQVYQHYFGKWSKIPYYVY

FFTYPMLAFSSVRLPFMTMYAIVQIRMQVYLLHQHISEISGEYVYDMKNLQILCDQNYQN

EIYDKMRLIISHHIMLKRWMRKLVHTVQISMPVFVLLGTMTSISVLFYAIYSFHNINFIL

KVRLISVSVCTVLVVYMFSEAGQALSTETTGVFDLLMTCPWYVWNIKNRRILLIFMANSL

EPMTFSLAGVTLDYRFALGMLRTSCSYSLILYKLKTGIZ

>TcasOR199

MSMTRSKYFQDSDDPFSFIRKIFIDYGYSKKINYYNRVTFTFNTCSILLESYYMITNFSL

DLFVRYGGALSLMLYHVVTQFLVIAKQKSLEQLLEESKSYFWKADIFNSSVKNQILKSCN

HMQRKFCLLWTPFVACGIVLLPVWGDFTESHIFPQVYKAYFGHWSPIFYYFCISSYPFAV

YTSIRLPAIALYLFLQAHFQIVLLNQKILQISKNNDLDETTIFENMEYQKTIYRNLRSCI

SQHVALQKYITRILVSIQKAIPVYFCLAVLCLIAVIFFVLNNLNMSASNHFKARIFVSGV

CGSLILYTFTEAGQLLADTTGDIFNTLMQCPWYYWNIKNRTVFMIFMLHSLNPLKIDWGG

FTLGYSFGGAVIRTCCSYAVGLYNLRESKYZ

>TcasOR229

MSARPLHLRNFPYYFLKVLVFDFEQYSAGKVLSYFCAIVHSISIFLQMHYLVKNFTKETM

FQYGCVLTVLTYCVVALFFAIASGNFVEKLESEISSFVWPLDICGEDVKAAILKRAFYTS

LVAYITIIAFPIFSVIMFPVLGDQSDMFLCVRVFNEYFTKWSQIPISLYFYSFPVIAFSG

IRLPGMLLYAILITHIQMFLLNRRIEQISELSNQRRVFETLCSCIELQAKLKRLIRNVFQ

LVYIAMPIFILLGAVSSVFVLFFVVNSLETASYFLVLRMGCFFGANVLVVFIFSQSGQSF

SDETGRIFDTLVMCSWYNWDKRNKKVLLMFLANSLEPMSITIAGITLDYKFALAMLRTSC

SYALVLYQMKNZ

>TcasOR27

MLETKQIVFHSFKLNVTVLSLIGLYPPKNYSILYKIYAVILFLAVHTPQLVLGLLHYFLM

GDFTSIDYSDFVTVGMMFYAFKLLPFVTSVTKIQKCINYFDTLGYKILKSEEKIIEDCVG

SCRRNTNVFFVGCCLSWMGFVAQVFLRDEPQQLPLKVWFPYSRDESPVLFYCIYILLIFG

PGYSVLACGTIDPMIGGLAYHAAAQLQRLKRNLQYLDEYIKEKNVGKSKENKRGVIYEEI

ISCVQRYQEIATFVDLFKDSFSQVVFSQFMGSVFLIGLCCFQIITATEVDINFVITANYI

WVILFQIFFYCYYGTMLIEENYTLTNAIYLSNWYEYSIPEQKALFMLMERSKKPMIVTAG

KILDLSLDTFTMILRRSYSLLCCLKZ

>TcasOR275FIX

MVHFPSQKRPLKDDPFQYLRRCLEPWGQKPSLLPLCLLILLIKIFFFTARTVFILKWMKE

IDRFGNFSHMACTTFGKRNRKFWDLGVGGPGLEKELEKRFKLINRFLFGHVSLGVFYVGL

YAFFADVPIPKGRTRWLPVLASMPFDQDQSPQYEILYVLMYWNLVVSILGHGVFDMVFIY

SSQHLVGQFILLKALLRKLDYGFEGLEIVAKARSRQFQKEIRKRIAICVQHHNLLLAYGN

ELKKIASMIFGVHVLSTSLTLILVGYILSKNLEKILQYSMLLSGVVSEALQFIIFAVQSG

EIYHKSVSVAQAAYQSNWYVFNAKAKRDLTLLILNSQKGISMYGAGLVTINNEILVSMIQ

KIFSSITLLRSLGEQKZ

>TcasOR283

MLKFEPKPTTKDELLWVVRTIYVDLFRNKLIQFALKMLFYGSIIMAIYQGVLFLYEFEIH

YFVKYSSMYCFTCFILLAAYSVPIIAEVATTAFTTIKCWKIDSGGALVENKIKQEAHFTN

IITAINCIFGLMVLVLFIVPFEDDNDFYFLFIAFEKYFPQWQQLLKWGFKAFFPCITILL

QAPFYIVIYACLRIKFELYMWMEFLKNLNIVYEKSDICELVHDSEYQTEISKRLRFCIER

QEHIYRSLLYGKKYVQQLDIYIFAYAILGSLGGISIIFVCISFEGNFFQGTYLRLSALTF

VTLTTFMHVIWAGQSVETTSSDSYDILKQCDWFLWNLKNRKTYLMCLNYTQRPLKAQFTQ

NVSINYVLGFSVVRTVYSTLTALNSLRKASKZ

>TcasOR286

MSVQKVSPDNLWLSAKICLHIFQYKAIKIILKILSLSIIILTCIQTFLYLKRFDSAYFMK

YLPVYAGSLFILASIFCIEHISHVILSTVEEFEFWDYADSKPEIRNWIKWEALYINTFMV

VDAFVAYLSGIFHAIPLDEDYEIFYPLPIFQEFFPDWTNVLGWLYRSSFLIVPVVMTAPS

LMIIYFTSRLRFQMFLFMDILENISEGYDISEANDLIENSTYQKEIKERLKTCIKRHNEF

LSAGGQVMKNGQLFILIMSAAGVILGVSIIFFLFSFEGSFEKRYPRLVTLVISTGLTFTH

VIIAGQLVENIATRLYEILHFMDWHSWNQENRKILLIFMHNAQQELQIKFLDEVAVNYQL

GISIGKAVYSMISVLSSFKNLEESYNZ

>TcasOR287

MISFEEKHLTNVRESDVLWLGRIMSLEIVQYKPMRFILNIIAVSIIALTLIQTYLFLQKF

DGLYLIKYASVYTASLFILFSIIAAPFLTKFSTEALNNLEYWPIESAGAQIEKQIQREAI

YINTFFVVNMVVSLISGVAHMIPLDDDKELFYPLAIFEEFAPKWKNWLEWGYRLSFLVVP

VVMLNSSYVGIYTLSNFRFQISLFNHLLKNINFPLNDNEQTIELMDDQKYQNEINKRLKF

CIKRQTHLYKVAHYVTGKVKHLSFFVAILTILLLIAVIVFLFSFQGTFENRYFRIITLVL

TAGNTFIHVIIMGNRIEEETEKIFENLKSLNWSSWNLQNRQVYLIFLHNNEEHFKVPISE

NASVNYELGISMAKTICSMVSVMSQLKNIDYSKNZ

>TcasOR288

MFSFEVKLDENLFKNDVLWLSRKLCLDYYNTKLVKIILFVLSIGVAILTIIQTFLFLQRF

DGRYFIKYAPVYAGSFLIFLSVEHIPFALMLINSFKTITFWRIDSCGPEIEQKIKKHAMW

TNICLISCTVVGLVSAIFHAMPLEDDDELFYPLAMFEEFMPQWKNLLSWIYRSSFLIVPF

SMPIPVYIAIYVITKSYFQILLFLSCLENLNTGFDTTSNHLLIYNNRYQNTVKKRLVFCI

KRHAYFSRAMNVHIKKMYVTIATFSIMGVILSVSVIAFLFSFQGNFENRYIRITTLVFTM

VTVSTHILYVGQLIEDAAFQVYTTLKTVDWNNWNLENRKLYLIYLQNAQIIFSIKFTQDV

SINYRLGFSMAKAIYSMISVMSKLRNVDYSKIZ

>TcasOR292

MQPNLQKNDILWLIRKLTFDLFQLKITKMFLIITSVSIILLTIIQTFLFLKKFNGYYFIM

YSAVYTGSLFILVSSLSVLPISKLIKTAWVKFSFWEINSATPKIERKIRKEIFYINCVVF

FNTIVAIISGIFHAIPLQDDEELFYPLAIFETYTPEWKDWFSGIYRASFLPMPIIMVAPA

YTVVYLCAHMRFQFCLLLHFLENINPDNENISDKKYQAQIKERLHFCIKRHIHLFSKSRP

VLEDLKKFVFVLTLCGTIFCISIIIFHFSFQGTYEGRYPRIITIIIAASITFFLSILPGQ

LIENTSSEIFEVLRNTNWVSWNEQNKKLFIILLLNTRQIYKIKITENVSLNYELGVTMAK

AMYSMISVMKQLZ

>TcasOR293

MISFEENIDHDIYKDDVLWIMRKISIDYFHYKVVKILLTLLSIGITILTVIQTFLFLERF

EGRYFVKYAPAYIATFLMVVAMQYISFSIRLAALIKRITFWTINSARVETERKIKKHAMY

TNIFFLGTVIMGVISALFHIMPLDDDNELFFPLILFEEFVPNWKNFFSWMYRLNFLAVPF

TLPIPIYITTYHLIKSYYQILLYLDFLKNINTGFDTTSSNNIESAEYQQVTRDRLVFCIK

RHSYFYTQMREVNRKMSKFIAIFALISVLLGGSVLTFLFSFQGTFENRYPRIVTLILTAG

CIFAHVIYAGQLIEEAATQVCENLKVLDWYHWNCHNRKLYLIFLQNTQKPYKTQFSQNVS

INYELGLSIIKTVYSLISVLRNLQDINZ

>TcasOR294

MIVYEEKIDQDVNGNDILWVMRKVCIDCFQYKIVKILLLLLAIFIAILTLVQGFRFLERF

DGPYFIKYAPAFIRTFVILVAIAFISFGMETAIYVKDTTFWLIDSAGLESEKRIKKHAMY

TNIFFVSYIVVGVISAIFHIIPLDDDNDVLYPLALFEEYVPDWKNLFSSIYRFTFLTVPF

TLGIPLYTAIYIITTVYYQILLFVVYVKNINTDLDVENVKYQETIEKRLIFCIQRHSSFL

KRMKESNRKMSATVLVFSIVGILLGASVLMFLFSFHMSFKIWYYRIITMVLPTGAIFIHI

IFIGQSLENAISQLEANLKMVEWYHWNIPNRKLYLIFLINTQEQKGVKFSQNVSVNYKLG

VSIAKAVYSLISLMSNLRSIDZ

>TcasOR314

MEQLPKNDPLLVLRALPEILMQHKIIKYVVLFIICYMTVTMILCSYVLATVRGLWDLFWS

QYSLLAFGSSIGFSCYFVAFWKGSEFIKLRRRVFANYWPLTSLGEESFQKIKKLSIFANV

FMVATILASLATSTAGLPWVGDEYDIMFPVRVYTDYFGERAVPLLVPFYLAMYCTGFVMI

STGFIFVHFALHLKFQFFLLNKRLDGLRTEPLVNDFLYQNHVKEELTCCIEYHQKLLKVA

KEMNDIVYYPIFIVVSCGIMFSVCLVFYMKNFKNSFVRGTTMAMTGTLTTFGFGFTGQLM

ENESGRLFDTSVMLPWHLWCLSNRKLYHIFLTKCQYHVSFSSSGIINLNHTLFISLYTKI

TSILSFLLNVSKKNHTKZ

>TcasOR315

MTLVRKLQAAATNAFEIRIKDDILAELFNWPFLVLDSKWSTKFAVFLTVYCVFETLACAL

VYSTLDVNMMGTYAIVIARFATTFCSFFSFFTKRKQYFEIINENFPHFWPLQSLGKSTFN

RIKMRASSVKFYSFLNVVVMLIGAVILISFTQDESEVYLSVKIYKDYVNKWTTGFVMFFY

VSFIYIGLVVAAISFVLTYTAFHLIFQCFLLNQKLKQINDSIVENEQKQAKFDEKYQSFI

YKELISCVKLHQRLILFGKRINHLVYAPLLVYIFGGIVVGVALIYYLKSSVQHIFTSLIL

LLIALINSTTFVINGQMLENEAENIYISLTNLPWYSLNVQNRRVVYVMLMQSQKIIHMSA

SGLVSLNYQLTIVFFRCIYTGMTFLVNVGLZ

>TcasOR316

MTLMRKLQTAIRNLFEIQIKDDILAELLDWPTLVLFSKWPKNFAIFSTIYCVFDTLVCTL

VYSTLDVEMLGKYAIFIAKSTIALCSFFSFFAKRKQYHKIINENFPHFWQLQSMGESTFD

QMKKIATTVKFYSCLSVVAMLIGAVILILFTEDESEIYLSVKIYKDYVNKWTTGYIMFFY

ASFLYIGIVTAAVVFGLTYIVFHLIFQCFLLNQKLKLINSYIVKNGQKLVKLEERNQNFI

YKELISCVKLHQRLIYFSNQINDLLYAPIFMYTFSGIVVGVALIYFLKTSIQYILTSLVL

SIVSLIITTTFVINGQLLEDETENIIISLTNLPWYSLNVQNRRVVYVMLMQSQKIIHMSA

SGIVSLNYQLTIVLFRCIYTAMTFLVNMGLZ

>TcasOR32

MCLSTSEQSFSINLKIMKLCRLFPPTEGKKFYKIQAYLLQFLLLLPIPILGNLHLLLDEN

LDMEKVNYNAVFLAQVTCFVIKLMAIIANSEKIKKCITELDSPKFAAVRENHKIILQHCI

KVCKRNTLIFVVFVICGASSWATKPLFWSRRNLPLDVWFPLDTTSTPVYCSLYIYLLIGV

YFTSFANMVIDPLIAGLAYHATSQIKILKDNLQHLNNVYANEEITSSKNKIIYMKIKRCV

QHYDDILSFVKEFEECFSLAIFSQISASVFVICFSCLQLSKIKTFGYYFIQLVFYFGVIL

AQIYFYCFYGSTLFEESSSIINAVYSSKWYDFDVPCRKALLILMERAQTPITVAAGKIMD

LSLVTFATILRRSYSLVAVLNNYQZ

>TcasOR36

MKGLVEKSFRVNLLVMQVMGFYPPQKYKSLYKIYTYVVYCAFTTLIPVLATLELFLAENI

NLEQISDNAFIVCEAGCFIIKYLPFVRNADKIKKSLFLIERPMFHIYTKRQEHIIEECVA

ICRRNCRLFLTFCTITVINWSITPFFLPGNNLPVEIWSPFEHKASRKFYFLSFVYIVAGV

GNAAVSSGVIDPLLAGLISHATSQLKVLKNNLQFLDEHAEERIASRNISFIERKRFKADF

IYQQIKLCVNHHIAITEFIDVYEDTYSSSVFIQFAASVVVICISCLRLSMVEPFTFTFFV

MALFLWTMLCEIFLYCYYGTILYEENHSLTNAIYMGKWYNYDIKSMKALVILMERSKRPM

IVTAGKILDLSLETFTTILRRAYSLLLVLKNYESTPTEZ

>TcasOR39

MSNQHEIDLTEFVKLNIKNMHFFGYFFPRFGHNKTRKTLYTMYSTLFVGSTFVLTALSQI

ANMINSFGDMERMTEASFILFTNVVQCFKIYSFLTYGPRVWNLIDGLNRNIFKPINTDQH

RILVNDIYMSKKISKIFLLACTLTCMSWAISPFFDKRGDVLRLPLSGWYPFNTDKSPAFE

LVYIYQILTTWIGGMGNISMDTFISGIIMAISSQLSILNNALKNITKNNELVRCVFHYRI

IIKFSDEVIYLFNTCLTTQFIVGVIIVCISMFQMSLVPVLSFQFVAMLLYQMCILLEIFL

WCFYGNEVMLKSDQLTQAAYMSDWTKSPNHFKQNLLFFMTRTQFPLKLYASGYFTLSLET

FKAIVKSSWSYFAVLNQVHSRQTQZ

>TcasOR40

MSSLIQESLHINLRVLEFFLLYTPGEPTNFQKLRSSILFFALMFHVPVLSGINLIVGKHD

NPMKLVDNSFGFVGLSCYIAKLWPLIGNRSKIKVCINYLDKPIVELRENQKGILQACSKI

CRRNSNIFLYYMIISVTGFVTKPFLFEERGFPVDVWLPTSLKDRLDVYWGFYIYVSIGVA

YPVIASGVLDPLIPSLLCLATGHLKVLNDNLEHLDEYSSEENGSKDSNLYKNIQKCIKHH

IEILNFVYNHQKCFSLMVFSQFLGSPMILCFTCWNVSMREPFSLEWFQSLAYFLGLLLQL

FFYCYYGTRLSEEFEHVTTAVYMGKWYKYDVKSRKALIILMERSKKPTIVTAGKILDLSL

ETFTIILKRSYSLLAVLKNQNZ

>TcasOR41

MDNTLDIDLTEFVRFNVNSIHFFGYFLPEFGKHPKKKIIYVIYAVIFVGTTFGLSLVSEI

ANMINAFGDIEKMTDASFLLLTNLVQCFKMYSFLTHGPRVWKLIHSMNNSDFKPKNLEQR

NILVEEIKMSKRISKTFFMACTIVCSLWGISPFIDRGNSEKLRLPLSGWYPYSTDTSPGY

EITYAHQTLTTWIDGLADVGMDTFLSGVIMVIAAQLSLLNNSLKNLTKNCKNDGKKANTN

LIECVIHYRTIISFADEVTYLFTSCITAQFIIGVIIVCVSLFQMTLVSLRSFQFFSMFLY

QGCVLMEIFLWCYYGNEIILKSDELTRSAYMCEWIEESREFKKNLIFFMTRTQFPLKLYA

SRYFTLSLETFTAVVKSSWSYFAVLNQVHTKZ

>TcasOR46

MSKSEKIHTLATYFDSNIAFLKLTAFWIYDDETTRRKKYLQHAYNIFWIFYLFVAYQPAE

LLYVYYSFNDLSVFLRALRDIGNHVSLAYKAFNYFIMRRDILKLMETLQHGNYHYEDCGD

FQPKLIVDEEKKEALKWTKYFLNFCNAICLSMFANGVFTFIFLSDKQYVERNGQRVYHQE

QPVNTVSPFGSGTKLRFFVTFIYTMIALTFYAWTIVALDSLFITIMSCISSHLKILQGAF

KTVRARFIKLCASLSKLLISVSGKLESIYSTQTFVQTFISLGEMCFSLYLLSETADQNIG

NEITYLIATGFELLMYCWFGNRITEASLKISYALYESDWFPTSLSFKKQIIFTMTRMQKP

INVTIGKITPLAFSTFLTIARGAYSFFTFLKQRHGINHZ

>TcasOR48

MPHATLSKMVVQKIDLLEPFDNVTRLLKILGLWYSPNETIVYKIYKNFVMATCFLYTLTC

TVYGFKFMSFETLEIAFGAVEGVLKSLMFRLKFQKIAESWQQIRQQEFQPRNEHQRTVLK

WYIEVTKSLFLVYFFGVYIGCISALTVSSWLRHKDFPTDHWFPFNYRRPFLYQYIYVHIT

VGFYLTAFLNCASDSCFYLSLLHITAQCEILADTLKNVHDLHKLNAAKKNSGQKGEDEVM

NQILIECMKHYNLIKKYTSLVADCFKEIITLQFVPTIVMICIAMYKISTLEPSNTQFWFF

AFTELGAITQIFIYCFVGNLVTSTSQKLFYATFESQWYNASQKFKKNLITVMMAVQRPVI

FYGWNIFAINYATFKSIVQTSWSMCVAFRSTQDLZ

>TcasOR49

MVVEKINLREPFENVTRLLKILGCWYFPNESLVYKMYKNFALITCCMYTVTSIIYSFKYM

SIDYDKAYESLEIGVGTAEGVLKGIIFRMKFQKITESWQQIQQPEFQPRNEKQKMLLRRY

IYVTKFLFKVYFFVVYIVCVTGLIVSSLLRHKDLPTDHWLPFDYRKPFLHQYIYLHLTAG

LYLNSLTNCAVDSCFYLSLLHITAQCDVLADTLKNIHDLDKLNAKNAPERENKDQVMNKI

LVECMKHFNLIKKFTNQITDCFKEILTLQFVPTVAMICMGMYKISTLQASSSQFWFFVCT

DLGATTQIFIYCFVGNLVTTTSEKLFYATFKSQWYNASQKFKKNLLTFMMAVQHPIIFYG

WDVFAINYETFKSIMRTSWSICVALKSTQDLZ

>TcasOR50

MVVEKINLLEPFENVTRLLKILGCWYSPNETAVYKIYKNFIIATCFIYTVTCNIYVFQKM

FTDSDKAYETLEIAVGSAEGVLKGIIFRTKFQKITESWQQIQQPEFQPRNEKQKSVLRRY

IEVTKTFFKVYFSLVYVGCVTGIVVSSWLRHKDLPTDHWLPFDFRRPFLYPYVYVHVTVG

LYLNSFTNCVLDSCFYLSLLHITAQCDVLADTLKNIHDLDKLNGKNVPERENVDEVMNKI

LTECMKHFKLIQKFTNVITDSFKEILTLQFVPTIAMICISMYKISTLHPSNTQFWFFIFT

DIGATTQIFIYCFVGNLVTTTSEKLFYAAFESQWYNASQKFKKNVITVMMAVQQPIIFYG

WNVFAINYETFKSIMRTSWSICVALKSTQDLZ

>TcasOR52

MSQIDLKEAFKQNIVLLKAMGLWFFQNERFYKLFKCFVQGSLVFDSTSLIIYVALNIRIK

NVTDTIYSLPGSLEVVLQAILFRKNFHLIRKSLNNLKQKEFQPKNDTQEKILKDSIALSR

RVFYSFFWLVFVMIGMWMVLPLTKKGKYLPTKYWIPFDYRLPVVYELLYVFECSCIIFHA

FSNVALDTFFSIAMIQIGAQCDVLCDTIRNMDEQEKTNTMDRILIECVHHYRLIEDFAKS

IATSFKEILMVQFVCSSLMLCVSMYELSLSEPMSGHFFQVLLFQISATNEIFLYCWFGNE

VIIKSERLFYAMFESKWYDSAATHRKNLMIFAHQVQKPISLLVWNIFPVDLKTFGGLLQK

CWSFFVAMKNIQEIQEZ

>TcasOR54

MNLQKLDPLEGFKPTISMLKIFSVWNSSNMFYKIYKNVTTLSLAITYTCVMICVVVNFNV

SEINENFYYIPALSTAPFKLVIFQKSFKKIQNLLFLLQSQYTKIRSEKQAKMVEDSVVLS

KRVVKVFAVLVVPTCVGLFGMPLLKDEIKLPLIIWIPFDYHEPVVFGLVYFVISFSGSFT

AYINIGTDTFFYNCLIQIETQCNILSDTLRNLHEFGRFEAEIHTILIECIEQYKTILKFT

KILSKTYQGILSVQFICSLLSLCLTMYRMSLADPGSEEFLRYFVFQWGVLPEIFLYCYFG

HRVLDSTKNLYYSTYELQWYNTSAKFKTNLLIFMGQIQNPIVIYVAGIFSLDLETFKKIM

QKAWSFFTALRNIHEQZ

>TcasOR55

MKAFSFNIKLFQIFGQWCYENESFYKIYKYTATVLLFLDWLFTMIFVLVNFQESEVVDSL

YISPSMTTSMLKYVIFRVNFSQVERMLKIVEEQYLKIDSRRVGFLVERGTKSSVFVIKAC

FYLVMATVVSLITQPLLQEDIDIPLVIWLPFDYHRSGIFELIYVYVSVSYLYFAYVNVAT

DCFFYISAIQIGVQCEIVGFMLENLNEIAKQEEENVRRLFLSCVTYYNNILECVKIISDC

YREILIVQFFCSFVALCMTMYQLSIVEPFSDVFFKMCVFQSAVICEIFLYCFFGDLVLEK

SGKLFYASFSFGWYNGSAKFQKELLIFMNQLQKPIIFHVGNVIPVTCETFKSIMQKSWSF

FIALKNTQNRZ

>TcasOR56

MVSTPLTNPLFSVAKLNAFKNTTLLLKIVGMWKFKTESIFYKIYKHVMVLHASFACVLSF

LYALKKYKDPEAIFFISYLPAIFTVPIKLVMFPINSGKIKQLLELLAAEKAIIRSAQQEE

ILENAMKLSRQIFNLFGGSFVAAAIVILVTPLSVRSHVMMYYEWFPVDYSKDSVYYGIFG

GSLYFLLHLIAVNAAGDFFFYISAIQIEGRFDLIVDTFLNLDEISARDGNSTKYERMHEI

VIECVQQYNIIIECSKLLIDCFKEILINQLICSCLSLMFSMYQLNAAEPLSLNFFRIIFY

AIAMGSEIFLFCFFGNRLIVKGEILYYSTFASGWYDAPLKIQKDLLIFMQQLQKPVMINV

GNIFPLNYDTFKGIMQKSWSFFVALKNTQDLRSKNZ

>TcasOR57

MSHSNPLEAFKLNTFFLKALTVWHVENPTYRLYKIFVVFSFAVTFFSAWICALVNYNVSE

ISENFYYLPAMSTGPLKYAIFQKNFTNIVNLTHLLETQYAKIRTENQKKIFDESVIFERK

VMKNFAILIIPTCVAMFIVPYFQDRREMPLIVWFPFDYKQPVVFDLVYFILAFACISIAY

TNVSTDAFFYTCLIQIETQCEIVSDTLRNLDKIVTNGFRNVAESRKIFIECIEQYNVILR

YTKIVSDTYQGILVVQFFCSLVALCLTMYKLSLADPGSQDFIKYFVFKLGVISEIFMYCY

FGHRVLEKTEDLYFAIYEMHWYDASKQIQNEVFIFMGQLEKPIVFYVANIFSLDLDTFKK

IMQKAWSFFTALKNMHDIRNNZ

>TcasOR59

MDEEFLIGTFETEKKFLRYGSFYPCGKRIKFIFLGLFMFVYSWTEFLSMITVLFVERDNL

TKLSETLLFCMTQAAFLFKLVNFLYHNKTMLRIESILKNPILNCLDQFEKNIIEKYMIRV

KYLARLFRILCILTVSFYGLFPFIDEDPDHMLPLPGWFPFDVKTHQIELVIAQTCGIAIG

AFLNSTLDILPTILITLGSAQFDILKIRLENITSVDTSKSWLVKKAIKKCVIYHTILLNY

ITQIEILFHKGIFVQFTASVVVICLTGFQMLVISVRSIQFILLMIYFSTMTCQIALYCWY

GNELMYRSMGLSDACYMSEWNKCDTSVCKSLAIIMERGKRPVVLKAGNIFSLKLTTLMTV

LKSSYSYFAVLQRLYATSE

>TcasOR63

MGFMIQDYDLRNAFSLERKLMLVVGFYPKRDNKHEILYWLSAFFNLLISYGQLTTMIIQM

VFDRSDLSKLTESLLYFFTHFTFLCKLLNFQYYSKDLIEIENFLTDPIFYGYSFEQLDII

KAKIRSCAFISNAFRICCTFTCSFYCLVPFIDESRKKILPLPGWFPYDTTNYYYSTFFVQ

SLSLFISAYCNTAIDILTWKLITLASAQFEILKENLTKIDYEGGFNETKGALVRCITHHA

KIVNYTERVEAIFSKGIFLQLFGSVIVICTTGFQLIVVPIPSVQFAVLGTYLCGMTTQVA

TYCYYGHEVMTTSDAIGMSLYLSNWYASHVKIRKIVMIFLEKTKKPTIVKAGNFITLSLA

TLTQILRSAYSYFAVLQRLYKDSZ

>TcasOR65

MTATKSLKEIPPIYLRVHLTVLQILGIDILPVESVPQNLFYTYTALIISTMCLFTIAEFL

DMVLNYEDIYRLTFGLCYCVTHVLGTVKMFLMLYLRKKLWGNLTTLEEGIFKPNPTRGGP

EELQIVNDAITMCNRQGYVFYTLVFLIIGARLLYASLANWPYDKHNYFDGNVTVIVNTKE

MPYTTWMPFDYNDSPLYETIFAFQIFSTTVYGFYIGAADAVICGFMMLIKAQFLIVKREL

ETLIERAQKAAIAENPDNEDNFGREIERIELLDKRTQDYVAKYANECVYHHQELIALCDH

AEEDFCYLMLLQFISSLLIVCFQLFQVSTLSPDSVEFFSMVCYLLLMLFQLLCYCWHGNE

VQIVSGELSRYAFGINWIIMRESPKKTLLLLMMRAQRPCYFTAGKFSLLSLQTFMTIVRG

AGSYFMFLRQMNIZ

>TcasOR67

MDFTIRDFDLRNSFSLERKLLLVLGFYPIRDKEKHRILHQLSAFLNLLLYYGQLLTIIIQ

MVIDRNDLSKLTDSTLYFLTLFTFLCKLFNFQYYGKDLIEVEKSLTDPIFYGYSFHKLQI

IKAKVRSCTLVCLAFRISCTCSCFIYSVVPFIDRSGQKTLSIPGWFPYDTAKHFYITFFL

QSLSLFISAHCNSATDTLPCKLISLATAQFELLKDNLRTIDYENSFEETKHALVKCITHH

RKIVNYTKRVETIFSKGIFLQLFASVLVICTTGFQLVIVPFGSLKFAIHGIYLCAMTAQI

AIYCYYGHDVMITSDEIGTSLYMSNWYASHIKIRKIMVIFLEKTKKPTIVLAGNFITLSL

VTLTQILRSAYSYFAVLRRLYADDZ

>TcasOR70

MPSIIDISFKININVLCLAGLYLPDKFKSLYRVYTYLVYVFIVIPVPTLGCVYLLAQEKI

TFRQIADNLFLIAELGCFIPKYWPLVRHAERIKRCIHYFSAPIFKTDRKEHQEILDDCIK

VCHQWSAFYFASVTAGFVSWSIRPISWENHILPTDIWLPFDPHTASSAKVASVYFYLVLG

KGFGLGIKILKNNLQHLGEYVDEELASLEPCRKAQLTYQKIRQCVIHHEHILAFVEEYEE

CFSQVALSQFVGAVVIFCVSCLQLTIVEVVSLDFLAMMMYFIAMLCQVYLYCHFGTILYD

ESDTISDAIYLSKWYEFDKRSKKALCILMERLKRPMTVTCGKIFTMSLVTFTMILRRAYS

LLAVLENYNIELNZ

>TcasOR73FIX

MTRKHIFLNFTVTILKLSFLWPSNDNYDQWRLVKDASLIVSLMPCALPILAHFVLQITGD

VYNMVTITENLIALICIIGMIYMTICFVKNRKLVKTLVKNLPAFTKYSKTTDIILTDKKA

NLYTKIFVFYGVIGNVVYMIMPYLNIEKCQQRQNNDVPCGLVTRCWFPFKFDYSPVFEIV

FVHQFYTCLMVSVIILDLTMLICGFLMHITNQLKHLRGFIKRFDCSSQKIAEDVIYCVKF

HTAIITYSEKTNEAFGTMMMLHITLTSLVISALGFEILIVDNFNDSLRFTLHLLGWLVLL

LLICYYGQLLIDESIAVAEDIYYVPWHLAPVDVQKDIYMILMRSQKPLTLNAANIGVMSF

PTFLRVISSAYSYFTLLLNIKSZ

>TcasOR76

MMESTVTRLKRMYLWPTASVTSRKPAFFLITFSCFLLYGSVMHLIVNDISMEEVHVIETT

AGQFGVLYYLTLFTIYRKGILEIYADLSNFTKFGKPYNFDKRNKQLNQWSRWFSVVLYFF

VISVFAWPGIFTQSCEDLNVALNKTEVCGVVSPVWLPFRFDYKPMKQFVYFWQSFCCLYS

NGGAGTISFAMSETIEHLILRVEDLKILFPKIVAERSPEVRRKMLAKWVDYHLWLLSIGK

LMNDTYRYSFSVIVLCAGTLFGCIGYTVMKNASTNFNSSFIFFGWMESVFVICVCGQRLM

DAFHSVGTTVYNSEWCDTDVDFQKGVILITIRAQKPVRIYAGPFSYVSHLLILTVFQTSY

SYINLLNASSZ

>TcasOR77

MKYILMKKTIAFLSVTGFWPKTKESTKTRAFCILFSSSFLLFGSLGYLIVYRKFGSDDID

SIETATSHFGVLYFMFFWILKRDGLVHIVNLLSDFSKFGEPRFFNDRNRQLDYLLQYCIF

VLSVATGGVFLCPIIFVKNCEMVKQEKNLTKVCGLVSNVWAPFDYSEYPMKRVVSLWESY

CCFINFGCGGIMSFTMIKTMEHLHIRVEQLKDMFPDVVNEKNLAVRKQKLEKWVKYHLHL

YDIGELMNNTYRYCLSVIVLCVGILFGCIGISTMQPGSSHNSLFLFMGWFQSICILCMVG

QRLLDVFLSVGVMAYDSAWYEKDVDFQKAVLMIMIRARRPVLIYAGPFTNLSHLLILGVL

QTSYSYINLLNAKZ

>TcasOR78

MGHAIMTEILTYLTLMGFWPRSPKSSKASAFLIILSTSFLFFGILFYLIVNRQFGSSEID

SIETITSQFGVLYYLILFTWKRNDIVEIVELLSDFSKFGKPPFFDQRSTRLNYRLSCIVL

ILIVANIVVAALPVIYIDSCHKANEQLNLTKTCGLIAPVWLPFDYNEYPRKHLVFAWEVY

CCVMNYVGSGIGALTMVGTMEHVIIRIEQLKYIFPKILDQPNPRIREQMLKNWVRYHLAL

FEIGRLMNDAYKWSLSVIVLCVGALFACIGISMLQSTASQINSICLFFGWFPSIAFLCMW

GQRLLDSSLSVGTAVYSSRWYDMDVAFQKSVLMILIRSQKPIRISVGPFTHLSMLLLLGV

FQSAYSYINLLNATSZ

>TcasOR79

MGHVIMNEILTYVTLLGLWPRSRKSTKTISYLIILSSSFLFFGSLLYLVVHRKFGSNEID

SIETVTSQFAVLYYMTFFTLKREGTVRIIDQMSDFSKFGKPPLFDQHNKRLNYLLSYFVI

CLFVAIVGVVALPAIYTGSCHKANEQLNLTKTCGLVAPVWLPFDYNGYPLKFLVFAWEGY

CCIITYACSGISSLVLVGTMEHLIIRIEQLKLMFPEILNEANRHIREQKLKNWVQYHLAL

FGIGKLMTATYTYCLSVIVLCVGILFGCIGVSTMQSASSNNSVFLFLGWFQSLIVLSVCG

QRLIDTCLSVGIAVYNSRWYDMDVSFQKSVHMILIRSQKPILIYTGPFSYLSHLLILSVL

QTAYSYINLLSARGZ

>TcasOR84

MTEEKELRLCLWSCYYLKLSLMWPLKREEFKSSKGLYLRLLVFVIISGSTFTAMIFMHLY

KSLKVGSYDVSEDLAILASNIGYVLMMTMYVSRQKDLELLLLDLSDFKTYGKPPNFDKVR

KRMDLYAHLIFFYSMFGSFVYNMDKIILIDKCKEARRINEVCGSAIPFWTPFETEDLFTL

TLVITYVLINIFVVVKVAMTVSVQVLEISSHINLRIEQLKIFIAGCFDRDFKASRERLDF

CIRYHNVIIDFSERFSRCFSYVMFIHLAITGIIIGCLENQIVQEHQPEAMLHMGGWSTAT

FIACYGGQLLMDASTSIADEFYNCPWYEADVKMRKDLILIILRAQKALFVSTGPFNVLSF

ALFVSIMKLSYSIFTVLSZ

>TcasOR87

MKHVIMDELLIFLTFLGLWPRTPTSPKIISYLMIYSTSFLFFGSSIYLILHRKFGSDEID

TIEIITSQFGVLYYLTLLVVKRDGITKIVNLLSDFSKFGKPPLFDQRSRRLNLLLRLFVT

VLLAATVAIVSVPVVFINSCNKQNLQLNATKICGLAAPVWLPFDYTQNPRKYFVSAMEIY

CATMNYAGSGSGAFLVIGTMEHLVIRIEHLKNMFPEILNEPDKQIREKRLKKWIEYHLSI

FEIGELMNETYKWPLSVIVLCVGILFGCIGVSTMQSVSFQNSSVFLFFGWFQSIFVLCFW

GQRLLDSCLSIRKAVYNSKWHEMDVSFQKSVLMILIRSERPVLIHAGPFSYLSNLLVLGV

LQTAYSYINLLNARSZ

>TcasOR89

MKEAVLQQSKKEMHLLNLWPKGHVKHFRFRYVITLIIVSPFTLGTLTHFINVLKENLDVD

LSGDISVIAVVTGLHFMLITFVWGHKKIAYLWENLGPHEYFGKPDNFEKRCKQLNFYSRL

YAYYCYLGLTVYIIMKNRGGIECRRLNVERNLTEICGLVTTFWAPFDIDFFPFRQILFVD

QVFATYFIVKGGAAISFTTLEVGEYIILKIKHLKRLVKEVFDDPREEVQRKKLVFCIKYH

QYIISIQELYDGRYKHCNGCYILMVGIIIASLSNEIMKNHNIEALLHLVGWVFSFYICCF

SGQSLLSESLTIPDAAFESKWYEAPVYMQKDLLLMMLRSQKPLMLHATPIGVMSLSLFIT

LVKTSYSYFTLLNQSTZ

>TcasOR90

MAKDTSPVLRESIEVMKYLQLWPQNERTNLRRRYFIVIFLCSPLHLGLATHLVVCLKDNL

DVDLSANIAVLSAVTGLTYMLIVFVWSQDKLVHLLAKLDTHEIFGTPDNLTKRSRRLNFY

AKLYSYYCYFGIVIYSLVQIIEMPQCRKMNEEKGLSEICGMIVPFWAPFDIDWFPLKQIF

WLNQLLGIYIIIKGGAAVSITTFEVAQYICLKIKHLNRLLREAFDDPCDVVVEQKLLHCI

RYQQHIIRTNELFNVCFKHCNGCYVVMVGIIIASLLNQILKEKSVGALVHFAGWICSFFI

CCHAGQAVISESLTIPEAALDSHWYEAPVKYKKVLLLLLVRSQKAFNLQATPIGIMSFDL

FIALLKTSYSYFTLLHKSTZ

>TcasOR92

MKNQEIKICRATLTVLKYSLIWPSEADEMNPGKWYYIRVVTFILFTCPWVLSVFMHLIVS

IRNNADIHLSEDVALMVAFTGVYYMTIIYVKKQPKVAFLLRDLSYFQFGKPPGFDETERI

LGFLSKLTFCYSVMAVVIYNYIKYRQKPECERMNKLKGLKENCGMLTPTWWPFEINYSPA

FQLIFLYIFTSTQVMMKLSLMISFNVLEMAHHIILRINHLKTMILESLDEQDYEASKRKI

KTCILYHLEILGFAERMDDCFSNGMFAHLTITAAICGCLEKQFVDGDNQLGSLLHIFGWI

LALFLACLGGQHLINASETISDAIWSSKWYDADLRLRKDLIFMMARSQVGLYLNVGGFGI

LSYALFLSVIKMSYSILAMLTSZ

>TcasOR94

MAIKICKFTRKNMQISLIWPREFEEINPGKWYYIRIVIFLITYGVFPFCTFLHAVVVIHN

NLDIRISEDIGAVVSNIGISYMAIIYVQQQNQIAYLLKDLSDFKDFGKPPFFEEENKRLN

FWSICTFIYPTCGASLYNLSKILEKSECNKINEENGLPATCGFIFPIWVPFNINYFPLFH

IMLISTWFCTTMFVRLHLSISYNAFEIAHHIILRIKHLNGMIITCFDCQDYKISRQKFTT

CVLYYKQILDLSNRLNQSFSSIMFVHFTMTSAVCGCLEKQFVDGEYVGGFIHLVGWIISL

FIASVGGQDLVNASQSISEAIWSSKWYLADIRLKKDVLFMLMRSQKDLHMSVGSFGVLSY

AFFVSVLKMSYSILAMLTSZ

>TcasOR97

MNNQKIQISNMTRKVLRYSLLWPKTNEELNPGIEYQFSVLGFFLVTGVLVLCITIRFFIT

IKAVHEVDAEVLAILIASYGSYYMICAHLKNQHKVALLMRDLSVFNNFGKPPNFDKRNNQ

LNFVAKLLALYSFLATIFYNGEQLINKTECKRINKEKGLSDHYCGLLAPCWLPFEIDYFP

VFHLILIYAFTSGYLLIKMAIHISYNAFEIVSNIVLRIEHLKAMILETFENRNKQVCHKK

FLQCILYHIEILDFAARLDDSFFNSMFGHLALTGGICACLEKQIVSGVNVVAGTLHFIGW

ILALFIGCVAGQYLINASEILPSAIWTAKWYDADLELKKKVLFMLARSQKSLFIRAGPFG

ILCYPLFVTVLKTSYSILCMLTSZ

>TcasOR98FIX

MVKKESEIKISRVTRKLLQYSLLWPTEGEELNPGKWFYFRIFAFLSFTSLWCIAICMHFI

FVMKDKPDWDPTEEIAIIIAIYGTYYIVLAYVKNQRKAAGILRDLSNFDKFGVPPGFEEE

EQRLRVYIICVFIYGFITITFYNFYKMSQKKSCERFNIEHNLHENCGLLSPVWIPFRIDK

FPRYELVFLYLLTCCHLLMKLPLIVSYNALEMVHHIILRINHLKIMITECFDDPDYEISR

RKLTQCILYHTEILEFATRVDDCFSNCMFAHLTLTGTICACLEKQIVAGFSRFGAILHFF

GWILALFIACLGGQQFINASDTIPEALWASKWYNADLRLRGDLLLMMMRSQRDLHITAGP

FGVVSYALFVSVLKASYSILCVLTSZ

>DmelGR21a-PA

MSFWAVSRGLTPPSKVVPMLNPNQRQFLEDEVRYREKLKLMARGDAMEEVYVRKQETVDD

PLELDKHDSFYQTTKSLLVLFQIMGVMPIHRNPPEKNLPRTGYSWGSKQVMWAIFIYSCQ

TTIVVLVLRERVKKFVTSPDKRFDEAIYNVIFISLLFTNFLLPVASWRHGPQVAIFKNMW

TNYQYKFFKTTGSPIVFPNLYPLTWSLCVFSWLLSIAINLSQYFLQPDFRLWYTFAYYPI

IAMLNCFCSLWYINCNAFGTASRALSDALQTTIRGEKPAQKLTEYRHLWVDLSHMMQQLG

RAYSNMYGMYCLVIFFTTIIATYGSISEIIDHGATYKEVGLFVIVFYCMGLLYIICNEAH

YASRKVGLDFQTKLLNINLTAVDAATQKEVEMLLVAINKNPPIMNLDGYANINRELITTN

ISFMATYLVVLLQFKITEQRRIGQQQA

>DmelGR21a-PB

MSFWAVSRGLTPPSKVVPMLNPNQRQFLEDEVRYREKLKLMARGDAMEEVYVRKQETVDD

PLELDKHDSFYQTTKSLLVLFQIMGVMPIHRNPPEKNLPRTGYSWGSKQVMWAIFIYSCQ

TTIVVLVLRERVKKFVTSPDKRFDEAIYNVIFISLLFTNFLLPVASWRHGPQVAIFKNMW

TNYQYKFFKTTGSPIVFPNLYPLTWSLCVFSWLLSIAINLSQYFLQPDFRLWYTFAYYPI

IAMLNCFCSLWYINCNAFGTASRALSDALQTTIRGEKPAQKLTEYRHLWVDLSHMMQQLG

RAYSNMYGMYCLVIFFTTIIATYGSISEIIDHGATYKEVGLFVIVFYCMGLLYIICNEAH

YASRKVGLDFQTKLLNINLTAVDAATQKEVEMLLVAINKNPPIMNLDGYANINRELITTN

ISFMATYLVVLLQFKITEQRRIGQQQA

>DmelGR63a-PB

MANYYRRKKGDAVFLNAKPLNSANAQAYLYGVRKYSIGLAERLDADYEAPPLDRKKSSDS

TASNNPEFKPSVFYRNIDPINWFLRIIGVLPIVRHGPARAKFEMNSASFIYSVVFFVLLA

CYVGYVANNRIHIVRSLSGPFEEAVIAYLFLVNILPIMIIPILWYEARKIAKLFNDWDDF

EVLYYQISGHSLPLKLRQKAVYIAIVLPILSVLSVVITHVTMSDLNINQVVPYCILDNLT

AMLGAWWFLICEAMSITAHLLAERFQKALKHIGPAAMVADYRVLWLRLSKLTRDTGNALC

YTFVFMSLYLFFIITLSIYGLMSQLSEGFGIKDIGLTITALWNIGLLFYICDEAHYASVN

VRTNFQKKLLMVELNWMNSDAQTEINMFLRATEMNPSTINCGGFFDVNRTLFKGLLTTMV

TYLVVLLQFQISIPTDKGDSEGANNITVVDFVMDSLDNDMSLMGASTLSTTTVGTTLPPP

IMKLKGRKG

>DmelGR5a-PA

MRQLKGRNRCNRAVRHLKVQGKMWLKNLKSGLEQIRESQVRGTRKNFLHDGSFHEAVAPV

LAVAQCFCLMPVCGISAPTYRGLSFNRRSWRFWYSSLYLCSTSVDLAFSIRRVAHSVLDV

RSVEPIVFHVSILIASWQFLNLAQLWPGLMRHWAAVERRLPGYTCCLQRARPARRLKLVA

FVLLVVSLMEHLLSIISVVYYDFCPRRSDPVESYLLGASAQLFEVFPYSNWLAWLGKIQN

VLLTFGWSYMDIFLMMLGMGLSEMLARLNRSLEQQVRQPMPEAYWTWSRTLYRSIVELIR

EVDDAVSGIMLISFGSNLYFICLQLLKSINTMPSSAHAVYFYFSLLFLLSRSTAVLLFVS

AINDQAREPLRLLRLVPLKGYHPEVFRFAAELASDQVALTGLKFFNVTRKLFLAMAGTVA

TYELVLIQFHEDKKTWDCSPFNLD

>DmelGR64a-PA

MKGPNLNFRKTPSKDNGVKQVESLARPETPPPKFVEDSNLEFNVLASEKLPNYTNLDLFH

RAVFPFMFLAQCVAIMPLVGIRESNPRRVRFAYKSIPMFVTLIFMIATSILFLSMFTHLL

KIGITAKNFVGLVFFGCVLSAYVVFIRLAKKWPAVVRIWTRTEIPFTKPPYEIPKRNLSR

RVQLAALAIIGLSLGEHALYQVSAILSYTRRIQMCANITTVPSFNNYMQTNYDYVFQLLP

YSPIIAVLILLINGACTFVWNYMDLFIMMISKGLSYRFEQITTRIRKLEHEEVCESVFIQ

IREHYVKMCELLEFVDSAMSSLILLSCVNNLYFVCYQLLNVFNKLRWPINYIYFWYSLLY

LIGRTAFVFLTAADINEESKRGLGVLRRVSSRSWCVEVERLIFQMTTQTVALSGKKFYFL

TRRLLFGMAGTIVTYELVLLQFDEPNRRKGLQPLCA

>DmelGR43a-PA

MEISQPSIGIFYISKVLALAPYATVRNSKGRVEIGRSWLFTVYSATLTVVMVFLTYRGLL

FDANSEIPVRMKSATSKVVTALDVSVVVMAIVSGVYCGLFSLNDTLELNDRLNKIDNTLN

AYNNFRRDRWRALGMAAVSLLAISILVGLDVGTWMRIAQDMNIAQSDTELNVHWYIPFYS

LYFILTGLQVNIANTAYGLGRRFGRLNRMLSSSFLAENNATSAIKPQKVSTVKNVSVNRP

AMPSALHASLTKLNGETLPSEAAAKNKGLLLKSLADSHESLGKCVHLLSNSFGIAVLFIL

VSCLLHLVATAYFLFLELLSKRDNGYLWVQMLWICFHFLRLLMVVEPCHLAARESRKTIQ

IVCEIERKVHEPILAEAVKKFWQQLLVVDADFSACGLCRVNRTILTSFASAIATYLVILI

QFQRTNG

>DmelGR43a-PB

MEISQPSIGIFYISKVLALAPYATVRNSKGRVEIGRSWLFTVYSATLTVVMVFLTYRGLL

FDANSEIPVRMKSATSKVVTALDVSVVVMAIVSGVYCGLFSLNDTLELNDRLNKIDNTLN

AYNNFRRDRWRALGMAAVSLLAISILVGLDVGTWMRIAQDMNIAQSDTELNVHWYIPFYS

LYFILTGLQVNIANTAYGLGRRFGRLNRMLSSSFLAENNATSAIKPQKVSTVKNVSVNRP

AMPSALHASLTKLNGETLPSEAAGDKAAARSLILNVELLKLGYFPAKNKGLLLKSLADSH

ESLGKCVHLLSNSFGIAVLFILVSCLLHLVATAYFLFLELLSKRDNGYLWVQMLWICFHF

LRLLMVVEPCHLAARESRKTIQIVCEIERKVHEPILAEAVKKFWQQLLVVDADFSACGLC

RVNRTILTSFASAIATYLVILIQFQRTNG

>DmelGR43a-PC

MEISQPSIGIFYISKVLALAPYATVRNSKGRVEIGRSWLFTVYSATLTVVMVFLTYRGLL

FDANSEIPVRASFRMKSATSKVVTALDVSVVVMAIVSGVYCGLFSLNDTLELNDRLNKID

NTLNAYNNFRRDRWRALGMAAVSLLAISILVGLDVGTWMRIAQDMNIAQSDTELNVHWYI

PFYSLYFILTGLQVNIANTAYGLGRRFGRLNRMLSSSFLAENNATSAIKPQKVSTVKNVS

VNRPAMPSALHASLTKLNGETLPSEAAGDKAAARSLILNVELLKLGYFPAKNKGLLLKSL

ADSHESLGKCVHLLSNSFGIAVLFILVSCLLHLVATAYFLFLELLSKRDNGYLWVQMLWI

CFHFLRLLMVVEPCHLAARESRKTIQIVCEIERKVHEPILAEAVKKFWQQLLVVDADFSA

CGLCRVNRTILTSFASAIATYLVILIQFQRTNG

**IRs and iGluRs**

>AglaIR25a

MIFLVFYHLLAMLHLSHEQTTQNINVLYVNEEGNEVADRAIEVAMTYIKKNNKLGISVDLKRVVGNRTDSNTLLESLCSTYQTMLETQAFPHLVLDTTMTGIGSETVKSFTSALAIPTVSASFGQDGDLRQWRNIDENEKEYLIQICPPADIIPEIVRAIVLNQNITNAAILFDNSFVMDHKYKSLLQNVATRHVITPIRGNGEQKVIEDQLVQLRKLDIVNFFVLGSVINIGTVLNAANEISFFNRKFAWHAITQDDGEFKCNCKNASIIFVKPSPNTAFQDRLGTMQRTYQLNAEPIITAAFYLDLALRSFLEMISDGAWKKNNVTNYISCDDYDGKNSPKRMNLNLRQYLSKDSTETPTYGPISVSSNGQSFMEFQMQISAVGVREGASDKSTTLGSWTAGFDNNLTLVEPQVMANMTADVVYRVVTVVQKPFIFKDETAPKKYNGYCIDLIEKIADILKFDYEIVAVDHFGTMDESGKWDGLVKELMEKKADIGLGSMSVMAERENVIDFTVPYYDLVGITILMKLPETPTSLFKFLTVLENEVWLCILAAYFFTSFLMWVFDRWSPYSYQNNREKYKDDEEKREFNLKECLWFCMTSLTPQGGGEAPKNLSGRLVAATWWLFGFIIIASYTANLAAFLTVSRLDTPIESLDDLSKQYKIQYAPLNGSSTQVYFERMANIEARFYEIWKDMSLNDSLSEVERAKLAVWDYPVSDKYTKMWQAMKEAGLPNTLDEAVKKVRDSKSSSEGFAFLGDATDIRYLEATNCDLTVVGEEFSRKPYAIAVQQGSPLKDQFNTAILQLLNRRELERLKEKWWNKNPEKKDCQKADDQSDGISIQNIGGVFIVIFVGIGLACITLAFEYWWYKYRKGSKIIDVQEASHHHHQSKTTFPKEAGFPKGKEGAGNKNVASLYPRPRF

>AglaIR8a

MDDKMLLRIKLKRKKIWECILLKEEGQERIVEWYNKGISTLNTNLRFETTFIDIIDEKADVDNICNAISIGVTLILDITWSSNDVARNLASDVGIPYVEVDVSISPALLLLDKYLDFRNSTDVSLIFDNPQYVDQALYYWIDSTRLRMMILETLDKITAKKLREIRPYPNNFALVANTQNMNRLLKIVLDEELVTRPDRWNLVFLDFNYDNFEEKLMYNKSINLLTLDKNMCCNLLNRKDFCYCPDSFNLQEEFFKKVLSVIVKSLEALVKDGQKHTNDIRCNATAYDNQTKLSFEEIFENKISRNQLYKNNSIVSVKVEGTIKIGRNESYEVVLRYLNDVMELQQGKTVNPIRPFYRIGITHAIPWSYKEQDPETEEWYWTGYCVDFAKKIAEELDFDFEFVEPKNGDFGKKVNGTWNGVIGDLASGQTDLAVAAIIMTADREEVVDFVAPYYEQTGITIVIRKPVRKTSLFKFMTVLKLEVWLSIVAALIVTGFMVWFLDKYSPYSARNNKKAYPYPCREFTLKESFWFALTSFTPQGGGEAPKALSGRTLVAAYWLFVVLMLATFTANLAAFLTVERMQAPVQSLEQLARQSRINYTVVKNSNTHQYFINMKFAEDTLYRMWKELTLNASTDDTRYRVWDYPIKEQYGHILLAINDSNPVATPEAGFRNVNEHLDADYAFIHDSSEIKYEISRNCNLTEVGEVFAEKPYAVAVQQGSHLQDDLSKTILILQKQRFFEDLRTKYWNHSSKGQCPSTDDNEGITLESLGGVFIATLFGLALAMITLVGEVFYYRRKGKSNAIKSNKAHVFPEKQLKPVPPRLINASTYPVTIGTTFKPVNAKQKGEKDDLRLSHITLYPRARNTITKINN

>AglaIR21a

MKLHLLSVLIVVITAKCEGADKRALQKFHEKPPWIKWSETFFGKTMVDQQAYLVKLLKKVTNDYLSECTPVILYDTFTELYDNLLLEKLLTNFPFAYIHGQITDNYELKVKTTLDKKAQSSCVSYILFMKDVMRSKDVIGEQNCNKVIVVARSSQWRVIEFLSHKESQNFVNLLVIVKSGQVVPQNEESPYILYTHKLYVDAIGSSKPDVLTTWMKGKFTRHVNLFPKKMVYGFSGHRFLVAVSHQPPFIVNNSGEAVAQMVQKRKANLGLGGLYITQDRIDRIAISQWHSQDCAVFISLTSTALPRYRAIMGPFHWTVWLAVTIIYMLAIFPLTFSYKLTLKGLITNPEEMENMFWYVFGTFTNCFTFSGTEAWNKSDKFTTRILIGFYWLFSIIITACYTGSIIAFVTIPIYPAVVDTVRQLLDGRYQIGTLDKGGWPHLFANSSDEGTEKLLRHLDLVPDVESGLRNTTKSFIWNYAFLGSRAQLDYIVRTNFTTKSKRSILHISEDCLAPFNIGVGYPKNSIYSEILDVGILLALQAGILNKLKADVEWDMMRTATGRLLAASSKVGGIKALSYEDRALNLDDTQGMFLLLGAGFLVGAGVLMSEWLGGCFMFCKGKKRNDSVSSIESNPRSYEGRTPREKLNSIQYVRMRNRLDSTIDLEQNFDYEVPRAKRVQMKKQESNGENHVNCVVHEVADSHSSGGSKDSDDDDFNEEINKIFEEVFGEKNVDSDSSHEEVTDTCDKENVIETTT

>AglaIR40aNEW

MRKTFGGDLASAVTDIVNDFPLDKIIICFDNQTDVNFILLLMQRLSASGISSTLFNLTSPDVQEKYFDFLNYHTEYHLEAYTIFFANHHLYEHILLEIYDRNYIRRNLVYIFNWGRLPLERNFVRNIEYAMKIYVITNPRTDTLFYSQATSHREHHLDMVNWWNHGKGLFSHPTLPIMKSVYKDFKGKVLQVPVLHKPPWYFVRYNNSNNGNFSNINNTMEVLGGRDDRILSLLSQKLNFRYNYFDPPEHIQGTPDSESGVFSGVLGLIWRREADLFLGDVALTYERSNFVEFSFLTLADSGAFVTHAPSKLNEALALLRPFHWQVWPAILVTCLAVGPMLYIIIALPNAWQPRFRVRSHARLFFDCTWFTITILLKQTGRLPSSSHKARFFIILLSISATYVITDMYSANLTSLLARPGRERAVNNLYQLEKAMESRGYSLFVESHSSSYGLLENGTGIYGNLWDLMKRNQGGNVLVQSVEEGVKLVRDSDKIAVMAGRETLYFDIQRFGPSNFHLSEKLNTAYSAIALQLGCPYIEEINRILMATFEAGIITKMTENEYEKLGKQKEISMEVEENLVSEAERENPRKTKANEDSEKLKPISIKMLQGTFYLLCLGNAFSGLILLAEILFYKHQKKYRVKKKRLVAMRKFSKFVRFQINKLRLTIRRLYRNVMHDAFVATLEYIE

>AglaIR41aJOI

MNLHLNLLVSVIIQKYFLNSRCIIYFSEKFNSLDYTGPIPILQINVQNMNIHKDLIFRYFGCQGIIISNNLPIWTFEIFEKLIRLSSERFNTRRYLILSGNNPKENLTDIFNIKELTHISNMVIIEEYKRKRKKTTGKQYIMDQKHITYIVSTHGYVGLKNNNRRIILDFWSSNLKKFKFNNNLFPNKLLNQMGRELRMATFHYEPYSIIGSTEDTHKGSEMSVALTFARQYNMTPVPVINKEDYWGEIFTNWSGNGLLGNLVLDKADIGFGALYTWEVDYHYLDLSKPLVRTGITCLVPAPNYPIDNFSSCFIFRLYRLIINILYIFRNKTLRKDPQYLLDYSVFTIIKLFVLQVVTKKEIPEGIGGKYFMALLFLFSLFLTSTYSSGLSSVMTIPRYENPINTVQDFAESGLFWGATQEAWISSIENSEEPIHKMLVKRFVSTTEENLREYTKTGEFAFSLERLPHENFAIGSYIKGDIIGNFHLMDEDIYWEQCVIMTRKNSVLLPILDDFILRVFESGLIGYWQNEAVILYMDSGVQQAVKYYHHQNRIIIKLNWSHVQGAFGVLSLGYIISIVVYLLEVILNIMKRNSNIRN

>AglaIR68a

MWTEGDLQLLIQEIMIKSLEPYECVVVIADPLYNEVFAKSWYTRFGTEISFIMIYVEESEDLLSPYSHTQESLLTAKKVGCQLYITLVANGIQVARLLRFGDRFRILNSLAKYIFLYDDRLFEEKLFYLWRKIINVVFIRRFEGKSTANGKKRSPWFELSTVPFPSQIHRVLVPKRLDIWTHSKFRKDTDLFRDKTLDLNNQTVKVVAFPHLPGTAKISSTSTARAFLSLKENAPACFSGTEIEIIQTVAKAMNFRCEIYEPENADVELWGRKMVGGIYTGIIGEMVNARADIALGDLYYTPYLLELMDLSVPYNTECLTFLTPESLTDNSWKTLVLPFKPIMWAAVLICLMLCTLAFYFLARFHVHMTTMTNKRVKNKNRIHPDPVEELRGKKKKVVTLSLFPEVEKLDPNVKYTLMKEQYQPPRDKDEPVGLYQFSQPVNSTLYTYSMLLLVSLPKLPSGWSLRMLTGWYWLYCLLLVTSYRASMTAILAKPTPRKLTYGGWGDLTREFFTSSDDATLKMISENFVMVNDSDEAVDQVADASFAFYENTYFLKEALVKRQLRFQSLSKRNFTSNQTEEAKNLAKQDRSLHIMNDCIINMPISIGLQKNSPMKPRFDKFIRMVMEAGLIKKWLDDVMQNVLNAEVQTDNEHEIKAIMNMKKFSGGLVALGIGYFISIVTLLVEIIYFNSVVKKNPSFNKYSKTVQNNKKSK

>AglaIR75a

MSFVCRKIFVSGDLNVLSKRFSQNFISHGLLSTNPNKYRLYNGNKVFVLNMYCEGSVEQLTEALKSNLFDSTSVWIIFGELDVIKSYNFYFPINSLVYVVEKKDHLHFSVKIIYKNNKVDLGYVEIDVAEWHLQERFLYFNKFFLSTNRSDLLGSPIRVSYVVMYNNTLNHLWDYRNTHTDILTRANYGIISHVLELLNASKVDIWTNTWGYRNETSNIINGMYGDLILDRADIAGTAGFMQTTTQKHFKFIPPINTMPTMFIFQAPPLSYTADIFSLTFTRMVFVTYISLMIFSAILFYYVSIFEWKQSLKTNNRTCKPTVSDVLFLECLVAIQRSISWELKSLPGKLVLFFTFTLCTYMYIGFSAYILVLLQSTSESITDLASLYDSEMEMGVEDMAFSRYYFSTPNEYSNETLRRMILENRIVPKNNWFSARDGLERVRKGFFAFHIQTGKAFSLLRYSFTEKEKCKIRTTPTILHSTEVSLVVPKSSVYTEIFLVGRIFETGLQRRDHIKFYATYFAKPHCTGRHGNYDSVRLMDCYSPFLYFSFGLLLSCLVLICEILWVKFYSKLE

>AglaIR75b

MLLKRWIIFITVYLKFSFEKENYSEVINLVTDFVKVQNCPTKIIACVCWRKDVRLNLWKALSRNYIFGQIRRVGRPLYYPSSEEHQMFLLDARCEGSESMLRQAQELNLFNQPFRWLILGTGSDLIFGNVYFGVDSRVYVIEDDDLGYRINSVYKYAENARELVINLVGVWTNSQGFSHFNDISAPQNRTNLMGTFLKVSYVITNPESQNHLWDYRHTHIDGISKLNYILVHHLLEFTNATSEKIFRDTWGYKNATTSLYSGMIGDLQIGNAELGGTPAFFTIDRIDIIDYIAVTAPTFMKFIFKAPPLTYVTNVFTLPFDTYVWYCSFALIAAILAVVYVIVVWEWRDPVFKNKGITVYSLRPNIFDVFLMEVGAITQQGSDAEPRSNAGRIATIFTFIALMFMYTSYSANIVALLQSTTESIRTLEDLLASRISLGVEDIIYAHYYFENAQEPTRKAIYEQKIAPKGQKPNFMTAREGIEKVQQGFFAFHIELSTGYKIINEIFQESEKCTLKEIVYINLIEPWLAVKKNSSYKEIFKVGLEKIHESGIQSREISRIYTKKPKCHGTGGNFGSAGLIDCYAAFLIFGIGVGCSVILMILEILMKTYKQNSPSNIIEEIKDDQKFI

>AglaIR75c

MSCKHDIFKKFMGVEHMCKKGKTPTVEKITMSKQFMKYSLRFLFIDLNNCPANLESVLRRRNHHLAVVVNGDCENVTNVLNVQTDKKYFYETFHWLIVDNSKNAMLFKLDKVKLNINSEVHLAICENSSCVIYDVYNPASEHGGKLVATVLGHYKTDIGYKAEMSQNKYWLRRNMTGVNFKSAVVLPRLDKPLKKYLEDEENRQINSMHRFQTVCVNYCRDFFNFSLEIQRTNSWGYIQSDGHFDGLVSLLERRIVDFGSSPLIFKLDRLPVIDYSFGNWILRSTFIYRRPDVVAEYYEIFLRPLSKWVWICVLIAMIVIVIALKIVLSNESSIGNNVEKLDSSWSFLWLYTFGAFCQQGATFYPKFFSSRILSIFVYLFCILIYQFYSAGIVSYLLMDPPRIINNLEDLTESQLRVGIEDILIDRNYFVQTTDPNAIALFERKIKGSSNDSGFYSPIEGLEMVREGGFAFHVETSTAYPIIEATFTNELICELQEVQMYRTQPMHTNLQKNSPFKEMLNYCMFKLSENGNMDRLRKHWDARKPTCIESAKKTVIHVSLKEFSCGLISLLYGICFALLFLLMEIVIYKRYALANMVKVKIGLRPSYPYIN

>AglaIR75d

MMQMMQLSRLIAVLYFVLVISVSGFLDCEIIEGYIKKRFINHVTIIGCFSKKEQLKIVKKLTSKLMTSVLNLNKTNIHQTLETTLHLGVILDGDCNGVKHFLSMGGKHKYFDEKHHWLVLTSTTNFTHLFDNVEIYINGNIEIIYPEKNVSSVKNYTIQAVYNPSSSQGGYVKFYTVGFYNTMEGYTFGETYPKYSLRRNMTGVRLKTMVVLPIPFKGKLLDYLDNEENRDINTFNRYQYNLMSLCQRFYNFTKEVTISTTWGYKKEDGSFDGLVGALERKQVDYGSSPIFVRTDRAKVVQYGRKTWTLGAAFIFRNPKSLSSIEGFIKPLSSSIWALTAVSAVLLIILLRLSCVYEHQNIYQHVDMSWSYFVVCIIGALCQQGMSITPKFLSGRIVTLFLFLFALLIYQFYSASLVSNLLMEPPTKIKSLDDITRSNLKVGCEDIVYNREYFLNTIDKPSRKLYFQKLLGPNNNSHFLPPEKGLELVAKGQYAFHVETTIAYPIIRKTFKEDAICELRQIEMHRVQSLHTNFQKNSPYKEMFDICLQHLAEVGIMTREITFWRAAKPDCIRSSKTGAFNIGLDEFYPALALLGFGIACSVVILLFEMLFFYKQRCEEKQKEVLTYPFIK

>AglaIR75e

MKILELKLVTVWSVIHLINVNCLGIHKNETLLEDFFKNKNTRITISCCKVKLFHKTNMKSEKPKQIAFYRKIFENNMKLFFNDMSTENWFVMDFKNCNDYEILAKINGRKLFRYPLKYLFLVNVIDYSTFLKKLDSLSLLIMSEVVVASFGKESIDLYQPYKIQENESLIVENYGIWTENTGIRLYYDGNSFAKRRRDFRKLHITVSAVVHNIENVKLKDFDDIPAPQEDEPYFSQDMAIINNLMQYLNASYSLKIFDSYGYLNRTTGKFDGMVEDIYEERSDISIYVILSEERHAIVDFIKPAENFATKTKFILKKPAMSYIENIYYVTFTGKVWISSLAILGTLSVSLYVLLNWEAKHMNRKSSCCKYTFSDITLISLEAVCQQGTMTDSSTYSGRTIIFLFFTAFMFLYVAYSAYILVLLQSTKPITSIRHLVESRVECGGLNLSYTMAYYESTKDEALRDLYRKKIYPNRFYSLQDGLNKVQEGNFAFQVILGPAYNYILKTYTNYDICKIQELPGYMNTIMSPTVPKSSQYKKLFKIGLTMVEERGLQHRIKNRVSIRPRCYNEAGNFQSVRIYDCHSVFILFAMGVALAFGIFWLEIFVEKYYKRQKK

>AglaIR75f

MACCCYNIGTTRNITWSIHVVHPKFIVICTASAESFSLLKKLMLNGYQSKILQINSQDIEYPIPPNRQVFVLNLHCENSSRLLQRVDKLNFFRAPFRWIIHHSYDTETEALAKTFFSEINILVDSDVTLCELKTNGTVRIKKLYKNRSNYSINIEEMGYWTKNEGFSDSGFEKDTARRRQDLKGTKLNTCLVITQKNSLYHLTDKRDKHIDSISKVNYVLVEHLKDIINATFNYSVQQTWGYKNKKSEWSGMIGELTRNEADIGGTALFFTIDRINIIDYIAMATPTRSKFVFRQPKLSYVSNVYTLPFDYHVWLSTIFLVITVGIFLYVTVKWEWQKSKHDKMEDTSYPELRDSISDVAVLSFGALCQQGAAAIPACVSGRITTIIMFISLMFLYTSYSANIVALLQSSSNSIKTLEDLLHSRLQIGVDDTVFNRFYFPNSTEPTRRAIYLQKVSPPGKKDNFMPIEEGVRRLREGLFAFHMETGPGYKIVAEIFQEHEKCGLQEIQFLQVTDPWLAIQKNSSYKKLLKIGLRKIQESGLQSREVGLIYTKKPVCTSRGSSFISVGIVDCYPAAVVLAGGLTAAVTVLLMEIYIHYRSNFLKNINRHFFRNKIQERKTIS

>AglaIR75g

MRLFPYFVLVSRIHCLVSDETFNLITTFLEQNNSKLCYFFVCWKKADKVKMHKILSKENLRIAFAAVTGNVISNITDGSYERISFVLDLSCEKSDVILNESRKNNKFRFVHSWLMVSNSPIKRNDIKNMFENVTLRMDTDIKLAVPELDNRYSIFEIYHPGLEGDIKINEIGVHAYNKLTYFNKDISFYSARKDMSGILIRTGNRVNYSLGTPENYTEALRHRNRFTFGKFHFQLFLHLVYIHEFHYNTTIRAEWFGNSSSGIEAGLSRLLWDNEIDISSAGGILRAPRMHYYDYLLPYYQFRTSFFFRNPGNVSPGGEVLRPFSVTTWYCFLAATIVVSLAIKAAYWIERTFLKSRFDYSLVTSFVTTISLFAQQGSSTVPNHMGGRIIFLNILVLSILLYNYYTSALVSSLLNAKPDVLETVKELYESTLKVGTEYQPYTNTFIVQARGNEWVQKLNSSKIFTSGRANYYSPEEGLAKVKKGGFAYHTEVTTAYPIVSRTFEADPICDLAQIEFIDPASVGFMVPKRSQYRQLFLVSLVKMQQSGIFKRERDVWVASKPECLLSSRVLSVGVNELFMVYMILVGGMFSAAIIFCIEIIWQKNQGKTK

>AglaIR75h

MRFLFFLLLLGVNCQKRLETELILAYLKENDVKYCFLLVCWKDSDKVKLSKIVINEVENARITFLPPKKGSISKAITSGYSQITYILDLSCDQAFAILKEASESFKFSLTNSWLIIARPDMPVKNVKNLFGTLKLRLDMNVKIAVLKNNESRYHIYEIYDLGLEVDYKFHRLGDFKDGIFSSPVKEKQFFESRRNMTGVLVRIGNVVNFTDELLWKYRNVASYNEHGGLYGLYLYYLFQPFVELLKFRYNVTVRYPWFGYSDNGTDGGLGKLLYDEAIDIQIGGGFVTVGRTPYFEPLVVPYVNVKTYFFYRSPRIKKIGAQVLTTFSAEVWYCVLAIAVVMGAMIRAVYWVEDRYLACNHIYNFFTSVMTVLGVLTQQGSATFPRRTGGRILFLTTLIFSIWLYNYYTSGLVSSLLNSKPQMLDTFQDLYESPLEVGMEDQAYIHLFVMHTRDRYLKQLYQKKIYGRHGSRGPNIFSAQEGLERVSKGGFAFHTMVYTAYPIISRTFDQEAICDLGEIDVMDIIGAMFLRKKSHYKELFAISFTKLWESGVSKWVHDHVIEKRPECRSNLLLTAVGPQELFLIYAIFVIGVFLAIVILLAEIFYYKLQTRKGVWHQQESALVDSLQHSAPSNMRLKSR

>AglaIR76b

MGLIELVLAGLCLNATCPDESTIESLIISPRQRELTMLAEELKMETLRITTFNNGELSGYRKKGNELIGTGVAFDIIHILQDKYNFNYTIVAPEDNVFLSPTKKLGAKDLLINGRADLIAAFLPIINSFHEEISYSRRFDNAEWVVLMKRPSESATGSGLLAPFTTPVWILIILSLLVVGPIIYLLILIQARLTRDDNNRVYPLPACVWFVYGALLKQGTTINPTTDSSRLLFSTWWIFITILTAFYTANLTAFLTLSKFTLPINEPSDIKQRRYQWVSNRANGIKDYIKEENKEMLPRGAKLADIIGNGTTFPETDDMSILTHYVTDRDMMFIREKSVLDNIMYRDYKEKTKKGIDEVKRCTYVVAKFPITKFPRAFAFRNNFKYRKLFDSAIQHLVESGIIEHKFVENLPDTEICPLNLGNTERQLRNADLLLTYLIVAGGLTVAIVVFILELLIRRYWKKPRRSFRGLLGNNKHVPTPPFENNNNYNNFDYIKKITPPPPSYNSLFQPPFTYRNCQKKNINGRDYWIVKDGRGGRELIPHRTPSALLFQWSR

>AglaIR93a

MCRVMFVGIILYICVRTKAENFPSLITSNASLAIIIDREYVGDEYENIKADIENYLTYAKRELLKHGGVNHYFYSWTAINVRRDLSAVLSIASCSDTWKLFRSAEDEKLLHMAISETDCPRLPSNKAITIPLIKKGEEMPQLILDLRSAGIYNWKSVVIIYDRTLDRDMTTRVIKSITQQSNLNIKATGISLIKLERNITKYHLNEIMSTIRTKTVGGNFLVIVSHQLVETIMEYAKSLNLVDIKNQWLYIICDSIYNNSDITSYKRLLREGDNVAFVYNTSITSENCRVGKKCHSEEVLEGFMKALDEAIQDEYDIASQVSDEEWEAIRPTKLERRNYLLDRIKVKRNVSVKVTTKFLILIFKKHLARHGTCDNCTFWRMSTGETWGKEYQLANYFKNSEIQSVIEILPVGTWRPSDGPSMNDELFLHVAHGFRGKTLPVVSFHNPPWQILKINESGEVTEYTGLVFDIIKELSNNLNFTFTVEVVNKTVIKNSSVGNNSLEITNIATNSIPTEFIDMIRNKSVAFGACAFTVTEDNKHLINFTSPISIQTYTFLVARPKELSRALLFISPFTGDTWLCLSAAIISMGPILYYIHRFSPVYEYKGISMKGGLSSVQNCIWYMYGALLQQGGMHLPYADSARIIVGSWWLVVLVVATTYCGNLVAFLTFPKIDQPITTIDELIGHKDTVTWSFRRGSFLDMQLKTSTERRFRTLYDDAVKEHNISNLIKQIENGKHVFIDWKMRLQYIMKKQYLETDRCNLALGLDEFFDEQLALIVAQDIPYLPTINAEIKKLHQVGLIQKWLQDYLPKKDRCWKNRHIIEVNNHTVNMDDMQGSFFVLFFGFLVSILLLLLEKLWHKHAVKKKEKIIQPFMS

>AglaIR100a

MLPGYLVVTAVLIISFQNSHQLPAFSNTPECFPNADIWKIYSKHFTFATTITLKILTVRNHKKSEIKDATTDIINGLVSFTAKTVEVQNRDIEANANVNWTTMPCEIQRNEVVHNLPPSNTYKILRSFSDPEEVYLIVAWNQQVIYQHLQENHKFVFPKSRALYVVLLMFEQACGDEEIGYMLKMMWEKFDTLNIIVQVPCSSNSDDIYIYKPFKVMGGLVKISKIEEVLANEYLIINQLRSMNGYYGFDASVLAEMSNILNFKIKLVHGQYSDYGRVLKNGYVTGSLGKVVRRETHLSANGRFISDYGTHHIEFTRPFKSEQICFIVPKSKMKPHWMSILYCLKMSVWLLIMAVLLFAISVWYLYQMSVTRKQDLSKICLQICSICFSVPIKISTNGAGRLFLGSLMIFNIVFNGIFQGNLVMSFSKSVYFPDINSLEQLADSNLKISTSLSLFEDTTFKLFKVLQEKVVQPKDDDMTSMDKTAYLRIWAAVERKSEARVILETKYTNEEGEPLLHMINDCPRVEFLAYIVPKGSPLLPKFNSIIQMFLEAGFIEKWNVDIADAVIVQKRLNTKSRQNIPRKLTVTDFQAAFFILIGGYAVASIIFSIEISLHMYRRKYLEATETKLCFT

>AglaIR101

MYSVCILIIIMKLNIITRITCLRQTILLHQRQSSCTCGTNNRNSIIIFKRVLNTSQYLFKMTTTKLLVFFIICRSIYGFKTHFTNRCDETRGEFLKRCIVEVTDYFFTENTDITIFASPNAQKSFSSAGDIPRLFKSNTLQAVVALSVGEVSYGFKTYERKCNYIIETKNQTELNDVIEMLSSLPSWNPRAEFLIVCVEVVEKPHAVALNLSEVLWRKGIVKIAVLMPVANDQYQIYSRNPFKTPRHSLNTSPLGHCRLGTYHGKTPWSGPPLEECSNNCTLKVHYVDWPPLVLNAKMGLQPSNYELSQGFDMNILNIIANKLNLTVFYNTTGILWGKIYENGTTEGSFELLLRNTVDILIGGYSKTAERIKYFDITDSHIEVFQEWCVSNTPVMVGIRNISNIFRIEVWIAVVAVYFLVATCIYLSGRHNQHEFGTYRNIGNTLLTSFRVFLGMTVHALPRGTGTRYFISLLTIFGFYLTLYYSGYLTSIISAPKYVQKYNTLKDIYDYDLKTYFMPDDAKWILHDNSITSIVPSELIRKRWRNCRNMSWCFEIVATSEATSFYTDNLHKDYFLASHSRSMHCIRYNKIGTPVGIVLRKGFPFYTQFNLIINRIFEAGIILKWRRDIFKPKISALSKGDTDSSIKFKNLKPIFKFLLVSSCISLVVFLIEVVVHKLN

>AglaIR102

MKMLFSVTLLVIYQATLGHTKLQPLSRQYIRSKTSLSQCVTKTSRLLFDSGVVSVNTLYNNRSVLAFADAIIKTLEVENLQSLLIKRHSTSNSSSIRIAGTDNCIIHMTDQTDINWFFHVLKILSTQSIRTKLLVASSTVLPNSEDFVYKLFEEFGKGNIVKGVVLLPDKENCIKYKMYSWSLYQQSKRDKNNCCSFGKFHSNAPSFGNSIQCVNNCSIRIIYQKHPPYVMKAEMGFQSSNYFVSQGTEMNLLNMIAGHLNFDLEYNESRSYWGNIYENGTATDDIKLLLNNSVDILIGGYIRTLPRYKYLDLSDEFTYEFLVWCTKSIPVVKRAPNMVNIFTTDMWILVFLAYASASVSIWFLSRYNIYEFKCYKTLSATFFFNFAVLLGISVHTLPRSLKVQFAMALFIVFGFETTLLYTTYLTSIISTPRYIQKYSSLEDIYRDNLTTLLVPQAALYIYNDSDVLQGVPVRLIKERWQDCADMSQCLKIVAGSNRHAYCAPKLRVKHTVLENKYNMHCINHDVAVFPATTIMRKGFPHYVAFNKALNIILEAGFTFKWQNDVIKAKTSSSEPSDTHLEDISIRLKHLLPLCKVFLFCNLVSFMVLLCETLVYNKS

>AglaIR103PSE

MHVEPTWTAFQYNPDAKLVTAFSECVLQTSTRLFEDESQIVFLTFPDEMKCRWHVSDSAKCMLNFFHVRNKWTVLVMNSNITKYKFEKGINNYIIYICNEKDLTVVADTLKTKTFLHTYLKLLLISPSVYKNPESVSSNIISTLGMKGMFKTAILLGVNRNNSVTYKVYSZYLEHKFNFEKTIFASTCTLPAHHKKFVNYFKNYPVKVAYVDWPPLNVNAEMGYQSSNYYISQGLELNLLNMIAYLLEFNIQYYKGETWGNVYDNGTITGNFRFLLNNSVDILTGGYTYSADRLAYSDPSIPYIFDYLVWYVPNIPELIFPSLDVGVLVMVISVYLVVSVCVWLVSRLNVKELGVYRSISSTLFNVYAMLLGYGVQKLPRTTGNRYFIFLFIVFAFLVTIMYSSYLTSIISAPKYLQKYDSIVSIYDNNLITYFLPPTINRFPNNSDFIDTISIKLMKTKWIDCPNITWCLENVSTSTNKAFFSTLRQTQYIIARKKFSLHLLERNIFTLPIVFILRKGFPLRELLNGAIYRIAEAGFVNKWIKDSQTFRPINNGMITENSDDASIKFNNLLPVFGFFLVAQSFAFVICIVEIIVYKVRPNQLNYKKKNRILTYDHVFLNADPFPLSRDEASSNHHDGKRI

>AglaIR104

MTLIIKIILLVSVYKVNSSLKSLKSVSNLEHKISLVECLLEANRRHIEEGQIIYVALSQIDGISLSLVQDLVLPILSNELKWTFSTINVNEHIDKIGLYANKPERFLIYYRETSDIKKFFKHLKNTYLFTTNTYFVVVSSVKFTDPLHTATKIFQTFWQYKALFVVAMLEDPEKPGHFLVYTWYLFNDGNCGNSIDSVHLIDTCAHGKFKTNETWYDDRKNGTYKNCSVSVGITHYLPYVIDVNISSNRNYNYDPSLGLELNLLRIVSEVLNITLIYKNIFERGEIFLNGTEIGSFQSINDGAIDISVAGYAKSLFRCVNYDCSISYVQDGLRWCVPFQPLQTSNCFNIITNLSWILILSVCVITNILIWLTSKRNDDERELYTNPGNIFFYEYAVTVGISLRRLPKTSHVCLILMIFIIYSFYLRTMYQTFLISTLTNPTYSQKYQVMDDIYKYNLITYFPESAYKFFREEEVVLRKWKTCENGQCLRKIAEGHSAVCIRNAQVQYMVGFKRINHTNFYCFPNNIVRLHTNLVMRKGFFLRRDINRLIDILVRSGITAKFEKDVSGDKLRFGTDEPDEKVLKYEDLKFAFVLLLVGYIISIIVFIIELKRDVVLSLL

>AglaIR105

MSLLKLIIVSAICRSIFGSKINLTGQYGIDVESLSQCIVKATTCLFTKDAGIIMFTSSNDTSFTGKLLKLLEGSILKAVVVKSLITAPHVVKQHYEKYNYIIETESPRQLTQIIEILSSLPSWNSHAEFLIVSLETFERPIIAAIEFSETLWRKDVVRVAVLLPASKNSSLYKVYSWNPFQKPYQASNKQDIPKDYCSFGKFHKKTPWFDELFETCKNSCAFKVHYIDWPPLVVNAKLGLQPSNYILSQGFDINLLNMIANKLNLTVFYHTTGTLWGKIYENGTIYGNLGLLVNNTVDILIGGYRKTADRLQYFDVTDSYIQLFEVWCVSNTPVMVGFRSMSNIFRLEVWIAAVSAYFLVSTCIYISSNHKQHEFEAGRNFGDTLFSSLAVFLGIGVNALPRTTSARYFMTVLIVFGFYFTLYYSGYLTSIISAPKYVQKYNTLKDIYDYKLETYFMHDDIKWVLDDSSITSIVPNETIRKRWKDCYNMTWCFEKVATAKAVSFYTDNLHKDYFLASQNHSMHCIQHNKIGTPIALVLKKGFPFYRQFNIIINRIIEAGIISKWSKDIFKSKVSNQYNDNPDDASIKFVNLVQIFKFLLAGDSVSVLIFLMEIFVKRSSIRVM

>AglaIR106

MGNFKIALIVFLSILVFGQLKLAVPKLLVTSHDEIQPTSIGLCVFETSKRYFPKQSGTITFLTSDLGTTDISQSLVKVLMLHRNEYRWTFLINNSFNLVNKTGTNNYIIYVNDQKQLLVILDVLKYISWSIQVKFMVISTTTTDNVLLKTVQKISMLNLVFLCGTTDISKYMVFSWNSLRKEILKRNSCSSGIFESKAKLFEGKPLKCTRNCFVKVYYTKLPPTVMNDEKGMQHSNYFLNQGLDMNLLNMIATSLNLTLQYNRASDEWGNIYKNDTISGNLALLLNNTADVLIGNYMVTPKRLKYFDVTNSHNQQYQYWCVPCDPVLVSFRSMTNIFKKDLWILIIFVYVLISICIWITRLHILPETLYKSTSVRCFMALLAIFGFCITFTYSSFLTSIISTPKYIQKVNSIRDIYKYNMSTYFIRGINEKLVIELVKNDEVPENLIYERWRTCPNMSWCFEMIVTTKTHSVFTSNLRQSYFTAIKNYRMHCFQLNTVPTQLVLLMRKGFPYYIQINDIVKRIVEAGFISKWKRDLLESKNKNTEDVNDFDKSSIKLFNLMPIFYLLLIGNIVSFIIFLGEILTYKYNLQNYITTYMKH

>AglaIR107

MRSNQRRYYFKMDVTALLFILAFGQVKPVISKFQITNQSDVRPISMGLCVLETSKMYFVNKSGKVTYVPSNSVSQSFDDDILMLLINENRWTFLINKSIDCFDRTCTSNYIICVSDKSELLTVLDALNNISYSFQVKCIIISTITVDELFLNTLRKRNMFNILLLFRTEDISRYTVLSWSPVRKVPLMRYSCSFGRFENNQKLFNDKSFRCSRNCIVKVYYIKYPPMVINEERGMQRSNYFLNQGFDMNLLNMIATSLNMTLQYNRSSEVWGDVYENNTITGNLLLLLNNTADVLIGNYMVTSKRLEHFDMTDTHIQQYQYWCVPNDPVLVSFRNMANIFKKELWVLTIFVYASTSVCIWIASLHQKRELMTYKSLSSICLKTIAVLLGIGIHPLPKRSIVRYFMILSAIFGFYITSTYSGFLTSIISAPKSIQKVNSLRDIYKYNLSTFFTRGITKSVVIELAKRDVPESLIYEKWGTCHNLSWCLEMVSSTKTHTFFTTSLRQGYIARIRNYRMHCIRLNTVPIQLVIPMRKGFPYYTQFNNIVKRIVEGGFISKWKRDLLKSKDDCKEDVKDLEKSYIKFSNLEPIFRFLVIGNIISFVVFIHEVIIYRYNLHQRSFYFTK

>AglaIR108PSE

IFFVSVQLLVAIKGZIFCICIYFSSRGKFYGYSTIGSTLLKDLAVLLGISINLSRNTFSRYFMALFMIFGFKVTQLYTGYLTRIWPSISCPIPSHGXQDLAKYIMPDSESWVLGDMTAMEGVPVSVIRETLYXCTNSTWCYERVAIGKNSAXYIDNLLTDYVVATKGYSLNCLKLTSMGSYYSIIMRKGVPFYEHFNSIIDLTFEAGFLSKWSNSIMEPTVTFVNSNNAKIRYKNLQPIFYFFIVGNRFLLSFSLLNSL

>AglaIR109

MYLRKHLKLLFFLTLALFNQAISGKMKLEPAFQKNIRSVVSLSQCVTQVSRLYFNDVVTINSLYSNHSPLDLALKETMIKMLEMKNLQSLVIKQYKKNSSSVRVADGGSYIVHVTNQTEISLIFRILKDLSNRRANLLITSASIFSNPRNFTGKILEIFGKGNVVRGMILLADKESSVKYKMHSWSLYESASRQKNLCSFGRFRKNNTVSRSLRKCANFCNVSVIYQKNPPYVMNAEMGFQPSNYYLSQGTDINLLNLIAAHFNFNMQYKETKSYWGDIYENGTAVGDMILLLNNSVDILVGGYTKTLQRYKHFDLSNEYTYESLLWCTYSIPVIKRSPNMINIFSFETWTLIFFVYFLVSISTWVFGRYSKDESKCYKELSTVFLYNFVVLLGVILHSLPKTFKVRYIMALFIIFEYEVMILYTSYLTSIVSSPRYVQKYSSLRDIYNDNLTTLLVPPVSIYIYNDSETLEDVPVTLIKQRWHDCFNSSWCLEKIAASKRHAYCSPKFRMQFALLQKKYNVHCIEKDFGVFPVNMIMRKGFPHYAEFNRVLNRILGAGFILKWQNDIMKSKHQLSDPGVDDFSDFSIRLKHLLPICKAFLFFNTISFVVFLCETVIHNRIIFSNMFRSK

>AglaIR110

MRVQPLLCISIFLSIYQRYIIESKLQPLSANGITSATSLGQCINEISRYYYMNDSKIIAVTSSLEDYPEIIFKLVETDSNWSIKVIHLKNTNYLREHLSKVNNYLIYIEHPNDLVPILNDLKNVMSQSYRTKLIVVSTTIFKNPQGVASDIINILEREKVTKAIIFLTSESNSTRYAVHFWYSYTKLTNRYTEKPVNYCYFGALMRHGKRGEVVKKCKRNCEIKVYYEIHPPYVVDAKIKFQSTNYFTTQGFEINLLNMIANHLNLSIEYLKSGNLWGDIYDNGTVTSDFRFLLNKSADILVGSYTKNERREKHFDLSIPYIYEYMVWCVPHVPVVRFRSLGDIFTFEIWIFVILAYVSVAGCTWLLSMSSKKEHASYQRLGSVLFNDFGVLLGISVNVLPRTVKIRQIMASFTLFAFMVTLLYTSYLTSIISAPKYIQKYNTMKDIYENNLSTFLIYNSVVVFTDDTDEVYGVPVPLIRQKQVNCNNSTQCLEKVASSNNYAFCITELHKNYLIRNRHFLVHCMDQKIVTFYINMIMRRGFPHRQFFNIVLNRIISSGFLSKWKNDLLRPKYREDFEADESDIRFKHLKPLFKIFLMCTSFAFIVFLMELLF

>AglaIR111

MKLQVILHSLLFLNIYQTKNVKSKKLLPLSQDETMSTVSLGQCVTELSKIYFISDSRLLAITTSFDDPADVIFKLIEVKGYWSLMISHLRTTNYSSERFTRANNYLIYFKNVNELIPVLRALRKVMSKNYYTKLMIISTTVFRNPQEVASDVIDLLEAEEVTKALILLTGEHNSSKYAVHRWHSYSKLTSSHNDQPIGICSFGKLDQHGNEINGTVTKCKTDCDIKVYYKIHPPYVVNPEIGYQSSNYFKNQGFEINLLNMIARHLNLSVQYFESKEFWGDVYGNGTVTGDLRLLLNKSADVLLGSYSKTELREKYFDLTIPYVHEYMIWCVPHTPLFQFRSLVNIFTFKTWILISLIYLLVSICIWIISLLSKTEDVSYKSFSSVLFNDLVIFLSTPISILPKSTQIRYLMALLILFVLMTASVYTSYLTSIISSPRFVQKYNSMGDIYHYNLSTYMIPNSISRFTDDFDEVNDIPVSLIKKKWINCYNSTRCLEEVATSETYAFCILKLNKDYLIRTRELRIYCMNENIVIFYMNMVMRKGFPHGELFNVMINRIISAGFLRKWTKDFLEIKPKWNYFKSDKFSITFTHLKPFFKIYLTCNFFAFIVFLCEVLV

>AglaIR112PSE

MKHIIFRYLLSKLHAIMALHTFLCLSVFLNIYQGGIVETKLQSLSQADVTSTISLGQCITEISKRYFASDSQIFAITTSFEGYTDIIFKLIETDSYWALKIIYLNMINYLENHFTTINNCLIYIKHPKELVLILXLKNITTQNYRTKLIIISTNIFQNNQMVISDIVHILEEEEVTKAFIILARENNATIYAIHYWYSYSKLENNYTNLPVNYCHFGHMKTNGNKVDEAVAKCRTNCEIKVFYQSYPPYIINPELGFQQSNYFINQGLEVNLVNMVTFQLNLTVHYMKSGDVWGDFFENGTMTGDVRLLLNKSADVLTGSYAKTVQRYKYFDLTVPYVYVYMIWCVPHTPIFQFKSLAKHIQPZGVLTTYLSVSVYMWVLSINTKEEXDVLERKYNWNVFTVDNYSIRLKHLKLLFKIFVVCMLFVFMVFLGELLA

>AglaIR113

MYMMNVEIILNVSLLLNIYQTRTVETKLQPLSSGGIKSTTSLSQCVKEISKLYFRNDSKLFAIFASYNRHVDVILKIIETEGYWNLEVSHLTMINFSPEHFGKTNNYLIYAKDSLELVPVLRALRSVTSKNYRTKLMIVSATVFENPRNVASDITSLLEQEKVTKALIFLTGVKNTTRYTVHYWNLYSKLTHNITKLPVNYCSFGIMRRYRVDEVVAKCRTDCEIKVYYKSFPPYVIKAEMGQQSSNYFTSQGFEINLLNMIANHLNLRVKYVESIDFWGDIYENGTVTGDLRLLLNNSADVLIGSYSRTVLREKYFDLTIPYVHEHMVWCVPHTPIYQFRNIVDIFSFKIWMLIFMAYISISASMWVLSFRSLEEYVHYKSLSCILFQNLVILLGISVNVLPRSFRIRYLMALYLFFAFMASSLYTSYLTSILATPRFVQKYNSMGDVYRYNLSTYMMPNSITRFTDDFDTVNDVPVSLIKRKWINCYDVSECLEKVAASKTYAVCVAKLYKDYLIRTREIPLHCMNQNLVIFYMNIVMRKGFPHGQLFNILINRIVSAGFFSKWINDLLQPKPKWNFSETGNYSITFKHLKPFFTIFVVCMFIAFLIFLGELSASTICSFLF

>AglaIR114

MYLGVRLIFIFSLFLHIGTQMQYIRLNISKNEEIRRSLSMCIFQACRRFFHSNFETVTYSLPLIERDFASATVLNELVLPVLESDQRFALLIKDLKTAQQINHRYIGKSPSYIIQIKKEGELEANIRRLKRFTRWNPHAKFLVVSSTAFSYPLNVAIGVIKSLWNNNVVNAAVLLVDNENNTNFKVYSWKPYSSHSCGTNFSDVLPVGSCSFGVTDSNIDWFRNKIPKNLHGCPVKVKYMVWLPYVMSVKPIAQVPHYDADLGIDVNLLNVIANTLNLSLIYQKSSFDGWGGVGKDYSATKDLKLLKENNVDLVVGGYVNTPHRTVLFDSPQSHIQDSLLFCVPHTPIITGFRNFLNIFKFEVWILVCILYFIVSVCMWYASNFEGGEHLSYRSLPNCLLNNFLISIGLVVSILPKTMRVRCLACMFIIFSLKLNMIYTSYLTSVLSSPNYRQKYESMEEIYKHNLRTYFAPNTKLFFSGNDEKQLHSVPLNVITQRWRNCTNVPKCFNTISSKKDIAVCMPRSYKDYLLNNQARDKRRAIFCLKENVVSFSLTILMRKGFPLFSLFDKGICRIVEGGFINKWMRDIMMNKSERIGSHPEDVEKVQINFDTLVPVFYMLLMGYLGASLALVIELIVQNFNC

>AglaIR115

MCLKKFIVLPYFICCNGALPPLATHEEEEHSLNQCILNLIGSPFNQLTYMSCSQPVSKKRRSALRRLDLPVISHIHKENRWSIKREHVDKNHSFNTDSYNRHSGFIIVMESSQQIKPILLKMLMEHQTHKFLLIFQFPLENLEKVISKMIKSTSNRKEINFKIIAPDSTNSTRYIVHSWKQVCDKPIKRKLFYCGFGTFFNKTSLVDKQSPTKVCSLKAAIAENVPFVIVDKDTRAVRGIEIMVMDATASKLNLNISYIISKHTGKMHLNGQFTEMFLLLQNRTVDIVFGGQVMSEIKEKYFRSSVNYIDTAISWCAPNEPSFSYDKAVAGIINVLFIVSIITLCLCLSFVTWYISNLDVQELSVYQCFLTSFLNIFSILIGISVHLLPRTGKVRILMGSFITAALFLNTIYSSKLTSLLISAKFGMKYDDIDEIYRDNLTTYAKLPIINFLQDTKPIFASELTTEEILQHFVECKSETSCLEKATNRSIVFLPVLERNYLFKQGVSNRSSMHCSQQYLHRGHVAIYVRKEFPLMVRINNVIGKIASSGLITKWKNDVTGDFKTRKIYEQGIIELEHLLPVFKIYLLANCLAVLVFVMELIIGKLRK

>AglaIR116

MKERRRKLYVTWIIFVHCYAFTNLYLPVLYLYNTINLLVYFELHMYFVLPTLFICCSGILQPLPLRVEERSSLNQCSLKLIKRFNPKHIAIITQSSTLNYFNIYLHSPKFSLISRIHKETRWTVTVNNLTESSFNSDLYKKPIGVVVILESYDQLDILQKHLKYSGDNKLYVMLQTPVENPSNFLRQLLSPYFKKFYFNIKIVAPDSTNPTRFVVYSGIHKCGKPRKDKLVYCSFGRFYNKSSFTDNKAPSEGCLVKAVHGDRVPYTRGDDKTEGIETNLLNVMASVLHLTVVYSEMKDFGSVYFNGTSTGNFHLLQNGSADIAFGGYSMDEMRNRYFHSTLCYMEKELIWCAPSEAIFGYDIYFRGLFSAQLLIGVTIIYLGTSIIVWYMSTLEEQESRSFKNILNSLMNNFSVLIGISIRLPRTGRVRLLMGLFSSAALILNTLYSSYLTSTLLSSNYREKYETLDDIYKNNLTLYSTPLVTNYFVSTNSVRDTTIESILVKSQICVNITECFEKVAKNNSAFVYPKLSTEYLFKQDLIPYPSLIRCTQISIFKSLTTMYVRKEYPLLVQFNRIIGRIISSGLITKWENDATENTRHHIHHEDKVMIELKDLEPVFKIYLWITGFSVLVFLAELFIGRQRRNMN

>AglaIR117

MWNLILFITVSSIFGPSKCSGIVEYPEEPASKMSLSECVLAISKKYVYTKETITVASSVSEFPKYAIPLIVTSPMVVRRLTNEMRWTVLTANMNKYTTSSVTHYSLEKISRSYIIFIKSNGDISKNLMRLKTLNSWNTHSTFLIISSTVFQNPFQVASGILKVLWEEFLDGVVLLANPKNISVYDAYVSKPYARGNCGKNLGRVIVKDSCSMGTFRRNRYLFNNIVPKYIKNCNLLVGFTRIEPFTTVTQNDSTALGGIEVNLVNMIADILELNLVYREADHGGEIFFNGTATENLLLLQNGKIDIGISSYVKTFARSYYFDCSHSYIQDRLIWCVPHTPVLIRSYNFINIVDPISWMLLIILYVVTTNLIWFVSRVNGKELRPYHDYKNVSQYVFSISLGFPLRFHPRTYQIRFLFISIVFFSFCLNSAYVSFLTSVLTEPRYREKYDTEEDIYRNNLKTYFMLNSDRYFEEQKFQYVLDKMVNCMNMRQCMDDVAFRRNSAYCVSKIHVDYTQSRYISKNGEHLVHCFGNVVTMPVSMVMRKGFPLFPKINRLIHRILTSGFMAKWERDAIRTKAKVRRNKNNGIFVEFEDLVPVFVTLVVGYCISCLIFIVELKYGQK

>AglaIR118PSE

MWNVVIIISIIPIIYPTKCEAVLEPLEQPSYKISLCNCVLTAVKRYVHVKETITVASSITDYZKNIASVITSNQMVLVLLTNEAKWTVLTTNMNKCSTPPDHYSLEKISKSYIIFMRSVGEITTNLRKLKNLSSWNPHSYFLITSATIFQNPSEVAHGILQTLHEEELLDGVVLLANPKNISVINVYVSKPYANGNCSKHLEEIEVIDSCTMGTYRRNRDLFMNNIPKRLSNCTLYIGYSKVDPFVMDVKNNSMAINEYYQTDGLEVNLINVLANYLELKLLYFQENSGNVFLNGTATDNLLLLKEGKIDVAIGSYVKTFERSFYFDSSYIYIQDKLMWCVPHTPLLVNSQNFLNILDLTTWILLICLYLIAVSLIWFVSRVNGKEQRPYRNYKNVSQYVFSIFLGIPVPLQPSTFQIRIMFVFMVFFSFYLASTYLSFLTSVLTGSRYVEKYSTATDIYENNLKTYFVPNSGRYFQDKQFQHVLDKMIECENYRECMDNVAFQRDSAFCVSKMYVDYIYNSYVSKNSNPLLYCFGNIVSLSINMLMRKGFPLFSKINQLIGQIVSGGFIAKWKRDNLRTKAGNFVADIDISEDDHFLKLENLVPIFAALIFGHCLASLIFVIEMKYGKK

>AglaIR119

MNVINICAVLVLNILECKAILEPFKVPFHKISLCECILVASRRYVDTKETIIIASSITDHYRNITTVIPSNHMVLSLLFNETKWTVLTVNMGNFMKHATDYSFEKNCRSYVIFIRTPEEASINLKKLKTLHSWNPHSNFFIISTTVFHDPFEVASEILQSLWEEELLNSVVLLAQPENKTVFDAYVSRPYVNGNCGKNFGHVDKIDSCSMGLFRKNLNLFKNNIPKRLTNCSLSVGFVKVDPFVMAIENNSIPGKDYHVNRGIEINLLNVIANYLELKLVYYQGRTGRIFFNGTATGNLLPLKEGKIDLAIGYYVRTLVRSYYLDCSHIYIQDSLTWCVPHVPVLITSKHFFNILDTPLWILLILLYIVTTILLWVFSRMSEEELRSYNVYENVSQYVFSIFLGFSVRFQPRTLQIRIIFISLVLFSFYLDSAYLSCLTSVLTGSRYGEKYSTVKSIYENNLKTYFVQNSGRYFEDKQFQHVLDKMIECEDYKKCMDYVAFDRDSAFCVSKLYADYIRNSYVTRDNDALLYCFGKVVSMTINMLMRKGFPLYSTINKYISLIIEAGFMAKWEKDILNGKAGSFVADIVIGDSERHFLKLKNLLPLFGAIILGYGLSFLVFVFELKYAQS

>AglaIR120

MRMTILIVSTLCLSRCASKLEPLEINNFKISLCECVSTAIKRYVETKETITVASAITDFSKNVNSVIPINQMLLWRLVNNTKWTILTTNMIKYKEPISHYSLEKISRCYIIIIRSIGEISAHLKKLKSLNFWNPHSKFFVISSIKFHNPAQVANGILQTLWEEQLLNTVVLLASSKNGNIYDAYVSKPYRKGNCFRNVNEIEEIDSCTMGVYQKNNDFFKNNIPKRLNCTLSVGFVKVDPFIMIVRNNSGAREEYHHTYGLEINLLNVIAKYLDLELVYYESSVGEIFLNGTATDNLLLLREGKIDIAVGSYVKTYERAYYFECSNTYIQDKLVWCVPHTPVLINSQNFLSILDLTSWLLLVVLYLVTINIFWLVSGVKKNELDQYQDYKNVSQYVLSIFLGFPVLQPRTFQIRVVFILIVYFSFFLNSAYLSFLTSVLAGSRFKEKYGTMEDIYRNNLKTYFILNSARYFQDERYKYVLDKMIVCKKYGECMDNVVFRRDTAFCVPEMYVNYIYNSYISKNYNQLLHCFGNIVSLEQNFLMRKGFPLFSKINGIINQIVSGGLMVKWKRDVLRTKAGNVVADIDINENDGRFMKLKDLMPVFVTLIIGHCLTILIFAIELRRGRK

>AglaIR121

MFNSILLATLLVLKTEARLELLLEDELNAKWYLKECMVEATSRFFTSDYETITYCISGTDKNNTSLLLDDTELEILKALSSSRDILIKNTNNPQYHLQYSRKTKIYVIPIKTADEIKATIDELKKFATWNPHAKFLVISSTIFEDPHQIARETSKSLWQSNAMDVVVMLAKNLTYYKVYTWQPYHNYNCGNNFTYIRNIDSCSFGIIEKNISWFGGKLPERFNKCSIKARYVNRPPYAMRPSRKIEEEHGIEVNILNMIGDILNLTIHYAESTHLGRIKNNTLHGDFKLLENRTADLIIGSYVKTYQRSVLLDMSYSYTQKSLIWCVPHVVTLDNIENITNILKFETWVIVSLTNFVIISLIWCASWCEENERTSFKNFSRCLLNFFAVGLGTPLKFLPKTNRVRFLMGLYAVFSYDITTVCNTYLTSTLSSPAFKEKYSSIADIYRNDLKTYLLPEQIKYLGSIGDTINTGNVNFTEIKKKWITCSDIQKCLDDVAFRKDSSILLSKLHRDYLFNNNTQRKIHCLKEIMLTLPISMVMRKGFPLFSLFNELLLRIYSAGFISKWENEVLQGKYRKVKEVITFDAVKIKFKYLRLVFELLVISHCISTVVFILENIYYKYIQNKYYK

>AglaIR122PSE

MVLFIFFSICAGANLNFLEADGMSPTTSASTCIVEASKRYFAGHYSTIVYVTSSETHPFTSTTIALLQKKYPQLILMNNFFEKDHDHKFTKLHSNNNYIIQIRNKQELLNVVDILRMLSTWNTRAKFILVATETIENSSVVAIELIQSLWTVDVVNSVVLLPASGNTTLYGVYSLSLYGQTNRDTEILPRNYCSFGIFHNSTSWYDEPIKKCRSNCTIKVNYVEWSPIVMNPELGLQSSNYFTSQGIDMNLFNIIANQMEITVNYEKGTLWGNIEKNGTVTGNLKQLLNNTVDVLIGGYIMSPKRITLFDLSRSYMQVYPVCCAPTALIMTDFZSIQEVFPVEVWILVLSALVLTSVCLYLSSKRTGDFSGYSTIGGTVLKDLAVLLGVSVNVLPRNAISRYFMALFILFAFEITLFYNGYLTSILSAPKYKQRYNSLKDIYEENLETYIVPYSEPWLFDGNSVTKDVPIALVRKKLRSCVNLTWCYEKVAISKDSAFYVDDMITDYMIATRGYSLNCIKLASVGSYYSIAMRKGFPLYAQFNKIIDYTFEAGFLLKWKQDILQHRYTSTDFSNARIEFKHLETIFYFLIVGNFVSFLFFLVELVYSYFKR

>AglaIR123

MMLLLFIFFIIYTRVLGTNLNFLKTETMSSTMSASRCVVEASKRYFTSYSTIMYVTSPETYPLTCLTISLLQRNYPQLVLIKSFFKTTLGYSFTKLQGNNNYIIQVRNKKEFIEVVEILRTRPYWNVHSKFIIVASETFENPNASARELIKSLWKVNVANSVVLLPAPQNTTLYGIYSLSLHEQPHHGSDVSPKNYCSFGAFHNNSSWYDEPIKKCRNNCTVKVNYVEWPPVVMNSNLGFQVSNYIVNQGTDMNLINMIVRHLNITVSYIKGTLWGVIKETGNATENFKCLLNNSLDILIGGYFLTLERLTYFDVTYSNVQTFPICCVPIVPVMVHFRSMEDIFPIEVWILVVSVLFLTSTCIYLLSQRGNELSGYSTIGGTLVKDLAVLLGISINVLPRNSISRYFMALFMIFGFQVTQFYTGYLTSIVSAPKYTHKYNSLEDIYKYDLDTYIVPYSESWILDGKTFVNGVPVSVIEKRWRYCMNSTWCYERVAISKDSAFYTDDLITDYLVAARGYSLNCFRLSATGSYYYIIMRKGFPFYSEFSDMIQRIFEAGFVTKWKMLPPTPTFDNCDNARIGYKNLLPIFYFIAVGNAVSFVVFLVELIYNFFKK

>AglaIR124

MRLALFFLLSHFSQMKFAVTKYQLIIRDEIHSPSLSSCILEVSKNFESSSETIVLLQSDFDQMETVFMLLEENYRWTSLITKGIHVSSTFNEAHNYIIYLLSQKELFVVLKKFVSSWNSDRKLVIVTTAHMEEPYRFATLFINELWKISVRNFVLLLATKNTPNYKVFSGNPIKEDLCSFGKLGNSKQLKIGENSCINNCTLKIYYISVPPAVINEEVGMLRSNYFINQGFDMNLLNMVAVHLNFTLQYIKSEGLWGRVYENKTTTGTLTFLSNNSADLLIGNYIKTSSRIKHFDVSDSHIQEYRVWCVSRDLAPVGFGSMVSIFSSEIWFLIGLTYALFSVSIWILSLNQNNELKSYRAFHSVLLKSIGVLLGTGVAPLPRTTGVRYFMVMLSLFGFTITLIYSGYLTGVITAPKYIQKIDSVRDIYNYNLTTYFVPGDIWLLEDDTMKEIPASTIRERWRNCPDIDWCFRKVATSNSYSFFTNNLREEYYAATKNYRMHCIKHNTIPIQLALVMRKGFPFYKQFNVVINRVIEGGFISKWKKDLLRSKSTDNDSEDSTIKLDHLEALFRFLLISHIMSIIIFIIEILLHTRICVF

>AglaIR125

MDIRVYSFYIQLYKHIFIKNKTSLKPYNVYISVTQQNPIPTTGYIKSRMSCNVYKQITLLFLCVHLDLTFSKFLLEDETESMNLCLLETSKSYFANDTKSIIFVSSKGDEIENFPFHFEDISHETVYRYTFLINKTLEMSHHTNSYILRVENEKDLAAVLERLRNLSSWNLHAKFIVVSTTTLDIPKQAAMLLFKELWQVNIVESVLLLETKDSTLHYAVFYWNPRKMVELKEASCSFGKIKYVEKLFDEPLVKCAHNCTFKVYYMAQPPSVIDGELGLQRSNYFINQGFDMNVMNIIAISLNLTLNYIKSEENWGFIFENGTTTANLALLLNKSADVLIGSYISTAERLKYFEVTVSHIQEYMLWCIPRHPVLVSFQSMVSIFSGGLWILIVFTYVLLSICIWMLSLHQKELASFKTIGSVLTKTLAVFLGVGIDFLPKNTSVRYFLYLMTLLGVNVTMMYSSYLTGIVTAPKFAEKINSVAEIYENNLTTYFLPTDIWLVGKDNMYNVPRSLIRERWKNCPNISWCFERIATTKNHSFFTNNLRKNYIVAKKKYRIHCIRQNTIPIQLVLLVRKGFPFKVQFDNIITNLIEGGFVSKWKNDLVKNKYIDDNFDEATIRFTNLLPIFKFLVVSHTICFTAFLGEIYLYKHQRNTAIRSDVIII

>AglaIR126

MMFVKFLLMVFLFKFICGFKEITETKNNLRESQCVHLAVEKYIGGKHILFIFSAESSQDGSSSMKDVYILQRLQEELKWSIAVRNVKDNITETSEIYWSKIDMYLISFKHVYEIEIILKVLEKYLSWNPRATFLIISRNNFKENIKITKKIFNLLLNYNVINVVIMLANQDDSQSFSFLTWNPFENGNCGNSVNFIVLIGSCTVGTFESNTTLYKNESFGHFKNCRATAGTINLGEYDFRLNHPKSYPEFRFLEMKLVDIIAKFSNITISHKEIKISSPGDIYDNGIATGGFQYLKNNTIDILIGGYSLTPLRYIHFEASTPYIFDSIRWCVPNYPVFTKFQRMFDIVRPTVWILIFSIYFLVAILIWWASSHSQEEYSCYISISNIFLYQFTIALSMSLNKLPKVFQVRYLVMIFVIFSFYVNIAYQTYLTSILADDSNIQKYGSQEDIYKYNLKTYFRQNSKRFFAGDKLVLQKYKDCFDFQKCMDFVARRRDSAFCTSRIRVNYVFNNFISKKKGPMIYCFRHDVVSYPISIIIQKGSPLYSHINKAVSILRETGIIGKLKEDSLKPRRKQISDFDVTESRTLKFDNLLPVFLMFVVGHVAAICAFFLELMTSKLFTGYLIKYA

>AglaIR127

MKILQSLFVYLATNAVKLCAMEISDTKLISRSTMEISMNQCLVSLIGRYLSDNETILIGLANGISDALLQSLSENLNWTVLVEKIEDGNTLIKDYLYSLKKPTFFLLYMKNGENIRYKIRILRQLNSKAIFLIVPPKSSIQMVKELFDILWQEHIINAVILINDPETNQCNMYKRKIFKNCGHRNNDGKIDLVDTCSFGIFLKNVSLFLNEEPAFNKNCTVLVGTINVEPFVMNIKNNDLGIKDDYLSKNGLEINLMNLVAKLLKIKVLYKQYFKPDWGHVYINGTGSGNLRLLVESKIDIALSSYEVSWARAFSLDFSYPHLADSLIWCVPHVPISKQKMYPIFDLSSSILILFVYLTVTILIWYTTFYYKHGNKPYMKLIDVFQKTFSMLIGIPGLLLSKTFKLRILILTLIFLSFTLVTIYQTFLTSMLAKSEFVEKYDTIEDIYKHNLKTYFQMNCERYFTELYDKDHIIFKRWKVCDKLKKCLQFVAFRKDSTFCGPQWYITYSIGKFVPKDALYCFGRLVGFPLTLLMRKGFPLHKDIDLQVRRIIDAGLLVKWKRDILRTGTIINPETVRSFDTDENGNHFLRLKNLVLIFNILLVGLGLASVVFIIELVYFNRKR

>AglaIR128PSE

YVKTYERAYFECSDTYIQSKLVWCVRHTPVLIXTQNFLSILDLTSWLLLAVLYLVTINIFWFVSGVKKNKLDQYQDYKNVSQYVLSVFLGFPVLFQPRTFQIRIVFILLVYSSFFLNSAYLNFLTSSRFKEKYSTMEDIYRNNLKTYFLLNSARYFQDERYKYVLDKMIECESYGECMDNAVFRRDSAFCVPEMYVNYISNSYISKNYNQLLHCFGNIVSLEQNLLMRKGFPLFSKINGIINZIVSGGLMVKWKRNVLRTKAGNVVANIDINENDGRFMKLRDLMPVFVTLIIGHCSAIVIFAIELRCGKK

>AglaIR129PSE

MNIINICAVLVLNLLECKAILEPFESPLHKISLCKCILVASRRYIHTKETYKHATDYSFEKISRSYVIFIRTPEEASINLKKLKTLNSWNPXSNFFIISTTVFHDPFEVASEILQSLWEAELLNSVVLLAQPENKIVFDAYVSRPYANGNCGKTFGHVDKIDSCSMGVFRKNLNLFKNNIPKRLTNCSLSVGFVKADPFVMAIENNSVPGKGYHVNRGIEINLLNVIAKYLELKVVYYQGRNGRIFFNGTATGNLLPLKEGKIDLAVGYYIRTLLRSYYFDCSHIYIQDSLTWCVPHVPVLITSKHFFNILGTPLWILLILLYIVTTILLWVFSRMSEEELRSYTVYENVSQYVLSIFLGISVRFQPRTLQIRIIFISLVLFSFYLDSAYLSCLTSVLTDSLYGEKYSTVKSIYENNLKTYFVPNSGTYFEDKQFQHVLDKMIECEDYKKCMDYVAFDRDSAFCVSKLYAGYIHNSYVTRDNDALLYCFGNLVSMTTNMFMRKGFPFYSTINKYISLIVEAGFMAMWEKDLLNEKAGSFVADIVIGDSERHFLKLKNLLPLFVALILGYGLSFLIFVFELKYAQS

>AglaIR130

MLLINLWENSNVLFVSVPIWIISCRGVLQLPLVHTNEDNSLNRCVYKLVKRFKLDHIRIITNSYLLSTSNTNILSHPSDISLVRRLHDGERWTVTTDNFNKNDFTSTLNRPTVFILVATTYHRLTQVLKKLSIENRSHKFLVIFQLPLEDPLQIVWEIMKYFFNENGGNVKIVFKDSSNSTSYIIHTFVQSCVKPLQHISYCSYGIFFNKSLLVDNKPSEHCSVKAFHTKRPPLTTFVKDNEKVIGIEGMILNTVASNLHLTVTYTESRNLGVVYRNGTTAGNFHLLQKGAADILFGGYSMFEDRIKYFQGTISYMRKKITWCAPLAPIFSYDGAFESALTFRFVISVVSFYFGTSFAVWCISNVAQNESLSYKNLLNSLTKTFSVLIGVSVRVLPRTRSVRLLIISFSITSLILNGFCSSRLTSRLIVVKYNEKYENLDEILKNNLKIYTASTVLNIFDSPELNYLDSRSVQRCLNVTRCLERVAYKNSALFLSKLDKNFFLGKKEVPYKTKSMIYCSHRHIYESLLTMYVRKDFPLLEEINRVMGRIISSGLIIKWRRDTFDKFEPYVHSDSLVKMKHLVPVFKMLISAIGLSIFVFVVELIMGRRR

>AglaIR131

MLILFTIIFQILFKDVNNHLQMGSEDKDIEVMKHCLFNIVVEATQPGETTIIITNNSQDDLFAKLNVDSKRQFERWSFGTYIEPEIFELSKGNSMNMFLLQINGTEELGYILDSLKSSYIFNSHAKFLVASTSKFENTTVAISAIVEILWASDIYNILVVVPHEEDSPIHNIYTWYPYGSKNCGNNFTNFKRIRQCSNGELSDGKLLYPNKIPRKLDGCQVKVRTVVWPPFVMKPKKRISNSDRYIFKKGFEIILLNTISKAINVNLTYTLSEKELNWGELFENATATGLQAYILENEGDIGISSQAYTRNRLKYFDMGVPYFYEHLMWCVPHADTIPAWKKIIIVVSLESWILNFVTFIFFILSIWGFSKLSAFENNRFRNFYNCIESTFGVLMGVAIFPLPKSNIVRLAFSLWFIYTIHIGIVYKTTLISTLTNPVYQHQVSTVGEILLSNIRMQFLSNMKKYFSEDQEEHSQEILYKWIDCVDLSDCMDRVAYTRDSVIFLPKLYLEFIENRYQRKNRPSYIFCFNGNVMTYAGSMIMRKGFPLKGRFDDVTRKILSSGLLDFWIRNSLKQLSVNKSQIFYEDDEILIGLKELQPTFVCLVVGLLLATLVFIIELCVFNYFSSTYWYGRAFII

>AglaIR132

MICLKILCTILSTTWLISCNGALQPPLLYSKAEKSFSQCTYKLVKQFKINYINLIEFPHVTSKSSNSFLYSSLLRLQKEKQWTVTINKLNENHFVPVFKGPTCFIILVNTRQDLNIVLKNLTTENHDNKFLVILESNLNNSVEAVPDATKLFFNRDEVNLKIIVPDCSNVTQHVVYSWVQRCDRPRRDRFIHCAFGSFYNKSLFLDNNTPVERCILKAVHANRPPYTSFIEDNNKVQGIDETLLNTVASALHLTVVYTEVNELGTAYFNGTMTGHLLLLQNGSADVLLGCYGLSVKKMKHFRFTTSHIQKKLSWCAPSNPTFSHDKAFEKALSFRIVYAVCLFLVTSLAVWCISNFEQRELPFYNNFLNTLTSCCFAMTGSSNRVLPRTTRVRLLMGLFSVSALIICTSYSSHLTSRVIFAKYIEKYHNLDEIYKDNLTVYMKEGTLNLFNTSGLNISSSLTTEKILTKLKKCMNISECLERVAYNNSVLFLSMLERTYAFKNKGYSLKPLLYCSDQPVYKFLLTMYVRTNFPLLGTINRAIERIISSGLINKWQRVVAGQFIPHVPTDSLIQLIHLIPVFKMYICLNGISALVFAVELIVGRIMKKQI

>AglaIR133

MLSLTACTVLLSTWFIFCRGTLQLPSSHSKEEYSLNQCTYRLVKKFKVNHIAFIRFHITPKSDDSFYYHSDLSSVSGIHTEKYWTVTIDNLNQYDVIPMFKRPTCFIITVKMREELNWVLKNLKFNDNLGNKFLVIFQSSLNSTVEAISNAMKLFFNRNQLNFKIIVPDSSNVTQHTVYSWVQRCGKPRRDRFIYCAFGSFYNKSLYVDNKKPVDPCLVKAFHAKRPPYTSYVDHNNKLAGIEEKVLSTVASALSLTVTYTEFNHLNEAYSNDTTTENFQLLQNGSADILFGCYALSEGRLKHFGSTVSYLTKKLSWCAHHKPMFSFGKAFEVVPMSRFLICIVGLYLGISLVVWCISNLQQGESPFYKKFLNAFTNSCLSFIGTSIGSLPMTKTVRLLITLLSLSALIINTFYSSHITSRLISAKYIQKYDNLSEIYDDNLTVYAAPGVLGMFNNSGINISASIIAEKVLSRMEKCWDTTQCLKRVAGNKSVLFLSSLEIYYFLKQEEIPIKSLIYCSRQYAYRFLLTMYVRKDFPLLGNVNRIIGRIKTSGLIDKWISEVGDKFTSLVHSDIMIKLKHLIPVFKIYFCVIALSIFVFVVELIIVKRG

>AglaIR134

MNFSNFIKYRFSVLIIVIIILFSNICPLLSEEQDVMVPCIEKIVEKYLNPNDLIFLINSKVVLNFPVVRYDSTEHLHIFNVEMPDAYVISVDSNLDILENIFMDIEHVNPRAKFVFICKSIELPLLEMLNYFYVHHVVLLEKNKKGLISVYSYDLSRDNRLTTAGHCTQKMFLEESELFQYHVPDNWRNGQITALYIQYAPFIMNVTSTDDKGIEMNLVNIISRHLNLNITYVKSNYEYWGEIHDGNFSSAWGELNSRRFDFAIGMFHQKFNEFLYFDVSFPYMEAAVKFIAPKARLLEDSIGSLLRIFQMDVWIVFFFTLLASLLLFYVIRRSLNNPSKSLRISDDIIFLIQLLVETSVRYNRNIKSVRCLLTFFILFMFSMNSALRSRLLLLFSEKRFEHEINTMEDIVHSGIGISYHPFFNMSFENYESDSEKYIYENSFPCCFDGECTNRAANQRDIVTIRTEEHVKYYSPRRYVDGEGKALIHITDVTIRVISVHMFFVKGFPLFTRINRDLEILKESGIVQYYYNKMEFDNEISLQKAAYKNKKLLQKSLSVQKLINVFYFYLIGVSLSIIIFMVEILQSYFI

>AglaIR135PSE

MSSGNFTKFWFLTLFNIILFPEKCSLCVRDSIVISCIEKVVEKYLNPDDLIVQINMNSAMNLAVVQYDPRKYFYVFSTRRPDAYIIAVEFDVDIMESVFNDIIHFNPRAKFIFICNEIRHDFLELLNYFYIHNVVLFKLNQSNLISLYSYDPYHDNRLSLNGZCSLNGVLEGKNIFQHTLPTDWRNTKLTVLYVEYPPYVMDINSTDYHGIEMSLIDIIMSHLQLNVTYVKSDFDYWGEIENENFTNSWGELKSRQFDFAFGWFHIKFNEFLYFDMSVAYLDDSIKFVTPKAKLQESVRLIGAFQMNVWIMFWASLLIFFLIFHINRKSSSNLKEISNNILFFVQLIIETSVKFNANRKFIHFFLCLCIFLIFNMNAAFKSKLFLLFSAKKFEHQIDTLEDIVSSGMVIGYHPYFNNSFENYEPDFRNRIYEKSIPCSFGWECTNRTANQRDMVAVQSEGQFEYFAPKDYVDEEGKALLHLTSVVMRPFPGHAFFVKGFPLFTRINRYLMILQESGFVRFHHNKIKLENEISMQKAIYKGNKLLKKSLDFSKVIGVFYFYFSGIFTSSVVFLIECIWYHYNCRKFKFEMSV

>AglaIR136

MICEHSFFYIQIALCMLLMGNCMLVEEKQSAILVNCIEKIVGKYFNPEDLIFQINLELKLHFSVVRYDPSNYLHVFNVRKPDGYIIYVGSNIEMLEFFFMDVLHFNPTAKFIFICHKITQNFLRMLKYFYVYQVVLLEIVETDLIQLYGYDPSIHSSFTVGSCTNKYFLEEKRLFQQHLPTDWSTYKVTVLYVQYPPYVTYINSTNFEGIEISLMNIISSHLNINVTYVKSDFNYWGEIEDQNFTDAWGELKSRKYDFAIGWFHLKFNEFLYFDTSVSYLHESVKVIAPKAKLLKASGITLFRAFEVSVWTMYVISLLTFLLVFHALRKYGTTSKATRITDDILWVTRIIAETSVRLNPDAKFIHFFLTLGIFFTFCMNAAFKSQLFLLFSEKKFEYQINSLEDVVNSGLGIGYHPYFNSSFEYYESDTEKYIYENSFPCPFDWSCVARVAAQADIVTIQTAGALKYLAPRHYVDEEGRALIHITDVVIRDFPGQAFFVKGFPLFPKINRCLSILVENGFVQLYYGKINFENEITLQKAVYKSSKLLNKSLNIRQLLGVFYFYFTGLIISLVIFISEMVTFHLKLKFMKYKMGTRKRRNCV

>AglaIR137PSE

MNFAYFKYZNLIWFILVSKYCSPIKNDAVESCIKHVLEKHVNPEELIFQINTNYLLNLPVIRYDPKKDLHLMNVRRPNVYVISVNNSLDILEITFSDIIHINPRAKFIIICSKITRNLLELLSYFYIHNKANEKGVIRLYTYDPYDNNRLSVAGDCTEMNILQEGKLFQLHTPANSRNVNLTALCIPYSQYVINLNSTVLKGIEIDLINDISCHLNINVTYIRSESVFWGTMENENFTDAWRELKSRQFDFVIGGFHMKYNEFLYFDASVAYLEDSVKMIAPKARLRDEGITSFFRIFEKSLWIVYLMTLLTIFVVFYISRTYISNISKSLQMSANIYFLVKLITETSVRCNVTNKSVNFLLMLCIFFVFNINAAFKSQLLILFSEKMFEHQINTMEEMVTSKMKIGYNPYFNKSFENYESKDEKYVYENSIPCSLDWTCMNRTAHRRDLITAQLEGEIKYYGPRRFVDSLGRALLHITDVIVRPLHIHVFFIXYSKINFQNKITLEKATYKSRKLINKSLILKHFMEAFFSYFVGVLLSVAVFLVEILSWYIKL

>AglaIR138

MKRLLIVFCFLRVYFITCLARYETTAQKDDDDVPVDCINHLLKDIYNPGDLILVLGKITQLHYPVINYDWTRPLTTFSVIKNNIAIIHLEKNQEYEQIIQKISEEQFFNPTARFIVISKRISSELLVVSSKFYLTDIIFIETVNKSFQSIYTYEPHQEQNSSFIPKSFKYLTQCKNGTVAMLKCFGHYRPPKTWKNSTLNVIYNVIPPYFQCPTCENGIETYLLDIASKRLGFRVNYVQQEDFQYWGSKLSGNYTYILEKLFQRKCDMVLGAFHSKCEEHLDFDTTFSFIRDGLFWLVPKASKLSGYKRIALVLNFETYFVLPAVMIIVPLIGYLLYKKHVLEYFFVLYQTLLEVALPRPPVHKLMYFHIIIPFLIVSTVFKGCLIHVLSVDHYEHQINSLEDIVASNLTINLDHDIAQYFKNDDPLDRYVYHRYEYCTNPRDCINRTAFKRDVVTVDIQRAYEYQLPNFYVDSDGKPLLYLFSSPISPLYIHWYLVKGFPIFSQIDYILLRLKSSGIIHYFCDKVIFKAKVNMKRKIGITSNILGLKEVLPLFVLISLGYILGLIAFFIELCTTVNRKKCRVAC

>AglaIR139

MYLSACIFLALLIKRSTELCPVNETIAGIIPEHVKKGFICLVNTNLTFQFPTCRFYGKKRLFWNNPGNVHFMPDLYIIDLSQGDDLEEVLFDLHRNTLFDPRATHLLVANEVNKQSLLHALKKFYIVETFVVNPKGKTYHFNRKLSKMSPVRKQEVIFQQSTKARLSDTTNITLRVGYLKKPPFVICPECPTNKGIEAEVFDLAANYLDVKVEYIQYRTTYWGDKLDGSYNFLYKGIFDRTFDVGFGQMFDYDDYHLDFDLTPIHVIDPIQFVVPKAKRINQFNIFNYIFKKETVLIHLLALALVFLTMCILNRWKNKLDKFLEFLQINFEVPFDMRRVNRPSTRCLYFFLMFSIIAFNTAYKSKLFSTLTMEHFEYQIDSLEDIVHSNLVPMFNNRTEGYFDRDNPIDKFLDTNSPRTCGFELDCVDMVAFHRNVTTMKLKKTLEYYTPRKYVDKKGRALMHIINEDVQKFYFRFFFVKGHPVFPRFSKIVNTLLENGINEYISEKLREDNRKAQIKAEGNYHQIFVRKLKITQLLNVVYIYIGGIILSLLVFCAELLWH

>AglaIR140

MTVINVLLFLTTTCGFNSASILPLTYHDFNENYATCVDQLVERLMSTEETMDFAFTINAELNRTIPVINWDIHKPSYWNTFLKISLYVIRIAEDDINLEEILEILENYDCLYPRATFILISEEVNTTRYPQKWRNSTINSLYFSVAPYVSPCHECKDFTNMSGIEIEIYKIVKDRVGFKVNFTEYREDDWGPKINRKYIGGLGSLQKRKVHVMIGACNAHKEEHLDFDITTTYYMDTCNWLVPRIEASFTFSRFLKIFTLSVWVLIFCTCISITILLFMKINNFPVALLSIFQIVMESGVHNFHRIFRKVRFILILFTLWSLVISTIFKNQLFVILSSQKLRNSIETFDDLVKSDLRILLSDRMVKVMSITTDPREKQIFNRLYPTPNMMHYHQIHAVGFEKHTATVSIRGLILFLLTKIFIDKEGNPLLHILDKPHYALNVQMFFTKGFPIKEEVDKYLRYCVDNGLLAKKRRDTEYDLKRDLYRRKIYVFGAQKIGFARLKWYFYLLGASLVLSILIFFVELLIHRLSTE

>AglaIR141

MLRYLIIILLSSTTSSIKADLDFEESLKEYIPIYKRRAENYSRCNMFFKSSSAVSKNMANWFASQNGCGNIIISNVLDYKFAVTDSHANFHFFESVQHVKGNLLKLPETFLFNGKGDSHFIIGTKTKDFKFLLDTFEFFWKKRFINCVIVFVYEHLEIFAYDTFRQVALNLTACDRCPLFSRVLRNFWGYPLRVNMFEEYPLLRKVDDRWMGKDYGIMMEIAKRSNVSLQILDTSGGFGGTMESLIRNESDICFVSMFLLTKYKMKGIDYVYPEHINSIVLLVPRSPKIPQYKKLIYIFDKNVWICLSITILGVALVVKIIATCNNRRNTYFYYLLVVWRSFLQQGVCSSRQRYHKARPFLVLWLYFSLIFGTAFQTSLMSMFIKSKYWKDINTLSEVRQLGLPIYVPAVYGDVIPKRYKLHKQLKFVEGMNQLHDLISTSNTFSGYIMTEEAARVYVEFIRSKDDKPIYHIVGEKLIPGINTYMVQKKSPFLKRINKMMLLIRQFGLRDHWMKNSPVVNENSKNIRMSNLQGVLYLLVLGYIISIMCFISELLIHKFKK

>AglaIR142

MHLQKLFCMAIVCIMKLVNIMVSFVVVMKVCGSFFQESNNVQIELNILMDKLKFCPHLDSSTCYYTISNSDSSKDIANSFLKKNNDCGTLVLNSNILNDDLLYEVGTFFVFVDTIENVPVAAVNLTENPLFNQMAYSFFIVTSQVEDLTVLNGILSYIWSKNILNFLLVFVHRSYEVYTFRPFAEEKVFDVKRLKNFCQYYNLNKVTNLHRYPLRAAFYEYPPGVYKENGKWTGSDFEKYLLIAKILNATTEIIPCHDANETVAKLYSNEADIFLVKVFNVRYFHNVSYSYSYEMGDRVAIVPKPTKIPAYRYIFYIFDFSTWCVLLLAISVLQALYFFISNTCGINIIVIWSSLVNMGIPRHLINRTKKKWLLMLWIYSSVVLGVCFQTSLTKSFVKPRYEKSIKTIEELRNSQMKIYIHNFLAQSYNIPESFGLQKNFVYMNVSETIQCIEEKNTSGAFIASRQLVGYYINENKRKYGDPVYEVVDEVLIPGSRAYLFQKTSPFLLKINKCLLLIEQYALHKKLYYNTIPPSGENILQLSHFLVMFYLLFLGYLVGFIVFLMEKYFI

>AglaIR143

MYTSLCVIILTTVVVVLLCDINVPDNYGFINLQIKKYIDKLKDATIHRSTVFYFVNPATTPQCRTMSNWWIKYSSMSSVIIDNGNITGVSQTFENFILFNFIEKLERFNEQFRYLQNRPYFDNRVNTHFIVCSEVHDKGFTKTLLPRIWKSKVYNFVVSYLYKSKLEMIAYNPFEDDSFTDYMKEDNIFPNKLTDMKKTNLRVAISDDYLKTFNKSNTCHDKNYRHVNVIERGMNINVSYIKISTEKKTLEMVELVMKGSVDFCSVSTLYDDKIKTVRFSYPIIMESVVVLVPKPKTVDQIVYLLDAFTIKKTSIFVFTIITMSCLHEYLMYSRIKRDSFIVTIFDYFGSFFGMTLSNFKQKRSILLLFWITFSLIFVTIVKAYLLPAALNPNYEKKLLTVADLRQSGLKIFVHEWIYDMIPEEHDLNRLISITNTSNIFNMISNGTKAAFIVTNTIATKYMTFYWKTNKEFKYDILNQDLLQTYIAYIFPKDSPLVAQIRWCILISKEMGVSEMSERIPDSVPTTNFLQIQHFLGALYLLATGLCLSILVFISEIILYYK

>AglaIR144

MISRVHLLVMFSVLQISETKIDPLPPETIRKDQIKLYCNKIDTAFENVTVVYKFFNKGGPVCSDFSSWCLESCTLPKFVGDGALTSPANVVFYNNVNNVQRHIAVRDIGRVENLRAPHHIVFCAEVDDASAKTIAKKIWAMKNLAYFVIVYVTNNLLKVISYDPYAKGGPNINFTNNYENVFARRPKVLKKSLFLMTPTYPVTIQENGKLKGAYIELMEALEICFKTNINPINESFLENLDVTYIQSHFHTNSDVLICVKPITYEEANKYSVTDPIDIEDVVVLVPKEKVKNEFNYIFSILTYKIIIWFFVILITMSFLNKNLTLGYNSSSYLDVLFDYFGSFLGLPIERFEMKRSIMILSWLIFSLIITVQFKGVFFNEIFNMQQEHKINSLEELKATRMNIYISLEMRSIEQHDNIKYLQDQVHFMTTVFVIDAIKNNSNHAFAVTDTTAKGFLNLHVQHKINFNYRVIDEKLASFFLARVARAALLDDINACILRATDAAVQKSSFVSWPKDSYDDGDRMLTSVHFVGAFILLFIGYLIALVAFLVELIVGRLRGATEDNHVILPYVN

>AglaIR145

MQAATMIVIFLLIPVFAHAAKTDTTELLLNITNAVFKDKKTICYVHDSNILSHVLDQALLRSHKVIVSYEMDRLKNITTDIFCNGYILCLNDLLEFEDYFGNKNKYSFYFKPHHRILLVFEKRSKVTPDLQFMTNLYALDIIAVFNLKPNDVYRSKMNSLYKNRKGNDKGKNLLVYSVYKNETLLEWNYRDILVFDECKFKPHTWVPKFYFDNKQFVFKISLFDCPPFVTIAEDGKVEGTEFRFIESVVKDWPIKYNIPHSKHKGMWSLIWKQMEANSSDVAACSLWQSTSEGKNVDFTYPFQQTCVTFLVPKPQLLPDSTFVFQSFQYTLWLVITLVVFLVSGCLFWFRIYHEDVDGYADMFQVVSLHLLRLISGGSIKGFPSLRASAVRFFVISWIIASVILSTYYQAGLTSNLTKPRYVNLINSLNDMVEKKVTWLAPTNFFQKHFRSSGTETLVKLADLHEKGFIEDLKENKALIVKTLANIYVCDIDMLCPEKRKPLKVLGECFERYYVGFVMQKNSPFTKIFNKFSFLYAEHDLLKYWYKLSTKEARAQEILFQTYRQEEKIWIELSKLKGSFYFLLAAHFVSSLVFVFEIVLHKYCR

>AglaIR146

MLVTKLIMQAFLVKLFSGISFEKNSHKIQTIFQYGFSNEHTICHIAYYKTIYHDFWIDAMVPVIVMIVINPDGKPRNVKFCDGFIISARNVTDFLKILSNFQFPPHKKVLLFLDEEYQTTFEIEAKVYRMALSLIVIHGLTGTQDLLDATIKIYFTVENKTKILGKDKLDKEDFSSKWTPQLWSKIGRNIRVSLFHCPPYVMVNHTINHTRYNLEGIDFNIFQIVTRNWPIEFRLEEEQENTRDNLFIKIIQQVVSKESDIAMCGLWQRVILERGTDMSNYNSVQCVTFLVTKPRLLGDETFIFQPFSDCLWLHMLGMLSLIFLFRFFLASCYKKYRTSASATDPTSWKPTNSSIKIFSQSWAFFCVLILTYYSAGLTRSLRFPSFSRNINSFKDMVDNGIHWEEPVPDIQKWLLQTNDTLYQKVGKLFFVGNNTTAMNLRLRSRKYALLVKLADNRYVMNGEYLDDFGRYHMKVIPQCLGNFYTVFAFQRNSPFVEIFNKEIPKFLEHGFVDHWQAIYLAAGEVRYMENLFKNLNVEVRQEIDFSKMRGAFRFLIYSYAASTVVFILEIFVHKNRYLKKTLNAIFSTLY

>AglaIR147

MKRCLALLVLLFLKVQCFDDTVSLISGIIKEVFSNEILCYITDKNVFNNVVEKSFINSGTEVISLSYDHLKHVTENLPCSGYILTLQDGDDFIRYYMDQKKYTFAFKPWSRLLLMYEKPPLHLHDLYLPSIVFALDVVVIDLPKEDEDVTVNVTSIFKNETILTWRPKQGTVDWTKFRPVKWTPSYGSLESNFTVALFNCPPFVFLNEDGEVGRGLEVQVIDEVIREVPIKYVVINSSQDIDQWAISSEMVQTNVTDMAVCSQWYINTYGKSLEFSYPIRQICSTFLVKKPVLLPASSFVFQQLQGAVYLVTFLILLVLSFLLALQWYFYKNREQLDLAPDYVVYPFHLLRILSMGGVPKLTLYVLASSKCLLGFWCCYCLIFTTYYSAGMTSCLTNPRYENNINTLQDMIYYKIHWFSKVPTYQDYLLGFNSPLYTTLADLFAIKHDKSDSIENAARIVKTIERRYVTEVEQLPQDVKKYYKVLDECMSKYYTGFLFQKNSPYTRVFDDTIIKLVESGIYDNWIDYILYRAEEEQRGFFTNYVEDPSTVIDLKKIQGAFFLLAGGYVLSFISFVVEYCLGKIVI

>AglaIR148

MKRYFTLFIIISIFYSNTVSYHESVSTFSHIIKEVFRDRIICYVNDRSSFNYLIQTTLLNTEIDIRVISISYNFLKDLTTDIPCTGYIVVLSDGNDFIKYHLNKKNSFMFKSWTRLLLMYETPPDLEKLYHLVLNSAIDILVVEPKLTNYSNLSFSYDGFVVKSVHKNTTILSWHSNQSEIDWTKFRPAKWTPHQVFKSLRVAMFNCPPFMYVNKEGKVVGGIEYLVIKEVIKHIPIETVLITSESWLLISDTVERSINDVAACSQWYISSQDKNLDFTYPIRQIFLTFLVKKPRLLPDSTFIFQPLHSLVYTVTLLILVLVLFGFALINKVLGNANHVHGFTLQLLYLVRVLSMGGLENLPVLLRTSTKFLLGFWYLYCVILSTSYSAGMTSCLTNPRYTNDIDTLQKMVDSKIEWYSMDTSLRDYFISLNISIFNELAKLYVRKHRILDRSATSAIAVKTIAHRYVTDINKLPYEDRKYYKVLTESVGTYYIGFVLQKNSPLTKLFDQTISRLVESGIYQSWVVRVLYQNDKFQSRFFTNYIEDPHVNVNIKKIQGAFYLLAGGYVLSLTSFLVELLLRNRAQHHTEIC

>AglaIR149

MIFFILYLILVKLILVSSIFNTSENSSFNICFSSIANKYLHQHTNVFLGSKFKEINFLTSRPFTIGNSTDFLLDNDQQSAVSYSLSYNHIDELEAILADIGISNTVYFIIIANHRDDVLKISKTLWRFRLYKSVILLPNKSTVKLYAIDLQNSKCGIHIKPKKINECTSRMFKRELDLFRPNMKKCFYKCPIRIIWSKYTPWIFDNNDTESGIYRDILTVFEEKSNFIITYMPESEVYNEELAFYNFGSIIADFDNDYADVFVGLVGFSASSEYVWTTGTIYEDNMYFVIPNPKMIPHWKLIYKIFSPTMWIICSILIFIFCSFFTLLALNSVVDRGRYGNFVDNSFYLFSLIITVGYYQFPRSFKLRLLLGTYFITTIISVAVTQGKYFSIINNSIYERRIRNVDQLLDSDVQIKCSEGLGLLFRYAVSNPKYSKVLERMENLPSRELSVNLKKAYDENFATVTDTGLLIMNPHLKYAMQVFKIYSFVTCFLVQKNHVLHEYFSNNLTTIVESGFVDKFVSHMKFKYARKQYFKEENRLVRYTLKHVEGPFLILAGGYVAGLFVFVLEHTYFHCRTKSMNNNQTR

>AglaIR150

MRCLLLKFLILTNLRAVVNLIMHEKGPTHRKIAENCIEEIIKEHFHFQYGKFDTNHTMLLTVMVTNNLSTPAFEIQENVLRKIHDLTEWFQIETLSSGKRFDFQSYAECYEDDCQKSFIIDFKSNFYVIIVDTEDNLDTILEVLEDSKTFNPKALFLVYLQYIGTEYRDTARNILSKMWDKLLIRTALLIPKTLQVLNLYKMHSPTRGPYECESNVTLATIDQCVKGKMRNIKKHYYAEKDERDLLNCSVGVITSPQEPFVINETAGFEIQVLKLIAGIINVHFNITFDENLTDTWGREENGTWSGRLKFVYENYYLGVGNVETGMAYATDFSFSRFYHMEPLVMVVPIARFVPKWRVLIAIFTVEMWGICLAMIVFFAATFYFASKFGHEIQSFKNFGDCCYQSFQIIVYHAVPAQPLNDLTRVFFVSFAIISIIMASAYTCSLIYYLKNPIREHQAKEMSDIMDSFGEYKYAIGGVNRFRELFTLSSDDEGSQLYEMYQSATGDNDTLLYWISKVAKERTIWTVSNKLYVDFLISNRSCATVNQDGKLSIYVFKKKLLAYSISIIMRKGHPLLKNIDSAIQRLTWGGFIAHLTDRYAHSIRRNSLMAADDDDDTFAPLAIENLQGAFALLAMGCCAGLVTLFLEFFVKKYRDEKSEKVDRKH

>AglaIR151

MVQLVSIFVYLALYLSCEASANKNLVSFIETFMRKSLAVVFGNSTNLTPILKIGHPKVLINLYRNNSSQVVLEKISSYIFMVDDLEDLELAVKTSVSTKNWDNRKSYLTIFQKPASKNTIQKSFSLLFTRKIFNSVAYFEKDKFYTWYPYSHESRCGTKVHLVRVRKWNPFINKVPRILPGCPVNVTWTKLDIFFWDPFNETFPGAIFLFFNTLAKKMNITVTYLENNTNFVKSHIKTGTYEVLEKDMVDRNIDLVLYWAVNGDSLHQNLEISQRFYQENFYFLAPSRRPYFRKNYVVNMYTTGVLCSLTASLVTSCFIWKLLTKRSIGDSIFNTFQLSVQLFINKTPSTTRTRIFFITLLLFLMRFNIAFQARLSSILTRPSYTPPINTIRQLLFNSDIKVIRAFNNSLRTMYAVDDIQEALRKKWIVFPGHRRNVDLIEHFVKNQSVAIVLSQVHFHHFKDPYQLHVVYYDPVGVTGRRYPFFVEVTRKYLVSQSDTRHVQNGFSLSNIPENVNFFQVGDLILLAGARKGFPLLEVMNYWINTLSERGFMAKYRDVSASIIKKFNFRNPVNERFIKLDMEHVEGPLVLWVAGVVISSIAFLGEIVALRIKKKYW

>AglaIR152

MCVIKNQSRLEYHYIPHFFPLNKKCLIIIKHFYSNWYRHILDDHLYYTKCSRPIDMNLKALSITLYLNLSAVFGLLPLAQPDCEGREQFLKVLKKNNPTDVVYVFDETECEDWSADIYRMFQPVYTINLECKGRLFREYSIIKDNDTVLNNENLYIISMRNFSLFEGVIDDIRHLVFWKARHRLVFITENDTGTVENFTERFKYCFKYWIINVVVVFGHNLEVAYTYNPFESDYFIEKDISEAFFDKTLNLHGFQLNITMFPEISNAREVNGSYEGIDGWMSTIIMDAINATYRYVVPSGGAVFGAFNETSADGALADIIPARTQAELTYSHRRNDLVVLVPIERANTLRNVFALTSRLTWALVLGLAVLIFLYIYIFNKHLDFGTIVTVIILVITCKPLHDLTFSKFRILFMSAMFLMFYIIAEFNCRLTSVLTVSFVPPSPRTLEELWETSRYRIKSRPSFARHLTTYVDSELGRKLMSNAEGLDRDIQIQQIMKCNKEIIISKRDIAKNAVKKSLIKDRNICYRIMKEIFMPNVQVYIVPYGSPFLGRFDDIIQRLKSNGIFAFWERGPKASRKEEISKKPYKISLDTLFVAFAVLGLGLIISTLVFLYEYKSVRGHKS

>AglaIR153

MYPIILIIILLCQWTAGSITLSKNVTLGICLESFTKFILKNHEFGNDKASFEAKNMLHSILTLCDYVSLSNNLHIVNLYGSDGTNYKIFEDYGYDRKLLMVQRGTYFNPTQLLVEGWVKKRARIGVLRVQSTNVTFVTNFNLEAENLCTKHFYEKFTCNKIVKITKHYLSKKNLDKMCRVKVSWTRGCPFVDDVHRTINPGLMVSFLKLYGEKSHLKVKFLKDSKYNHELLNNGTFYKLRRDLLDGKHDVAIGPLFMNATEELPFSYGPVFFNDMYLLTARKRNRITNYRKHAIVFSFYLWISCFITFLLVVLSYFLLNLALETRRLDFIATVFDMYRVGLSYSVTILPQSISLRLLFACYSFSYMILDSVYLGILSSICTNAPYEILDMRNVMHSARYDYYLIINWLAERLLKINFVAERNFYTFTDNRITNESDEALLKRVAYDQKDMTLTLLSTQKSHPYEALLVDSFLTLKADLNLFVTFYIRSDFHLKDSLQYWSQEMIEKGFVSKWMNDIIEQNRNGTHIIKERKEAIPLNFQHFEANFVVLGLGYALATVALILEVAYKWIDHRLHLTRKIIFMFSLLKPPHIYD

>AglaIR154

MMLQIALIVILLNQGYAENNFSRNNIILNNSTSGNCLRTTAKYILKDYKFENIDEANFEAESLLNSIVTFSDYVSHLNNLHIIVLNRSKAFGTYKYDKKFLVVQKGTSYINPVQLLVEGWVRKRAKIGVLQIQDDSVAFLTSLNLDTTKLCTKHFYEKLSCNNVVTITKHYLSKENLNKLCRVRVSWVRSYPFVDDVQGKTNPGIMVSFLNTYGEKGHLQVKFQKNNSEYNHELLHNGTFYKLRKDLFEGKYDVAIGPLFMNATEEVPFDYGPLFFKDQYHFTTTKRDRIPSYKKLTVIFSFDIWKSFLITFLLVTCSYFLLSLAREPRKLNFMAIVFDVYRMSLSSSINIIPQSVSLRILFIFYSLFSINIDSAYLGTLSSIFANTPYDIQQLEVWILMERGIPFNTNWLAERLMKLNFVAKRSFSPFSNIGNAIVNESDEDLLKRVAYTQKKLTFTLLSTLVSRPYEASLVDYFEVLNAQLSLFVTFYIRSDFHLKDSLQYWSQEVIEKGFVSKWMNDIIEQNRNGSYVIKEKKETIALKVQHFEVIFKLLSFGYALATMALILEFAYKWIDYHLGVTKKIKFIFSLLKPPSMYD

>AglaIR155

MMHQILLITVLLCQAHAMIPLEQDLNLPGGLRTIVRNILENFQFEMDYDFNFEAGSLLNSIVTLSDYKSYSRNLHIIVLSEIIATDCKFIDEYGCDKKFLVVQKEASYFDPEALLMEGWIRKRAKMGILQFLSDNITFTSNLNLENTKVCTRYFYEKYTSCDTNFTVSRHHLSKKYLKKLCRVRVSWVRGYPYVDDVRRKINPGLMVSFMNIYSEKRYIKVVFKKNSSEYNYEFIFNGTVHKLSKDLLEGKYDASIGLLFMNSTEDVPFNYGTIFINDNYLLVTKKRDKITSYKKLTIVFTVEVWKSFLITYLVATFSYFLLSVVIESRGFGFMGATFDVYRLSLSSGVSILPQSVSLRLLFVFYSIFSINIDSAYLGTLSSIFTNAPYDVRLIDIADLMATGGAFNVDWLSERLLKLGYVHNRSFSRFSKHGNMARNESEEDLLKRIAYAQKNMTITRHSTLLSHPYEASLVDYFSPPQTAYLTMFITFYIRRDFHLKDSLQYWSQQVLEKGFVKKWLHDIIEQNRNGSHIVKEKKGAISLKLQHFEEMFYLLIFGYTLAIVTLILEFVYKWIDNRLGITKKIIFLFNLLKPPPMYD

>TcasIR40a

MRRDHGGDLVSASFDIVAGFLFEEICICFDKNTNINFLQHLLVRFVSNNIAIKLFNITTV

EVQDKYFAFLNYQVTNHLGANTIFFSSHKFYEHVLLEINERDFIRRNLIYIFNWGRRPFS

RYFVRNIINVMKVFVITNPRNDTFRIFYNQAVPYKKHHLEMVNWWQHGVGLFNHPTLPAK

YNNVFKDFKENVFKIPVIHKPPWHFVQYGNDSIKVTGGRDDRILSLLSKKLNFRYDYFDP

PERIQGSSASENGTFKGVLGLIWKRQAEFFIGDVALSHERANYVEFSFITLADSGAFITH

APSKLNEALALLRPFQWQVWPAIGVTFVVVGPVLYAIIALPNAWRPRFRVRSHARLFFDC

TWFTTTVLLKQTGKEPSSSHKARFFIIILSISSTYVINDMYSANLTSLLAKPGREKAINN

LNQLEKAMATRGYDLYVERHSSSYSLFENGTGIYSRLWQMMNRRQTHFLLESVEEGVQLV

RDSTNKAVIAGRETLFFDIQRFGASNFHLSEKLNTAYSAIALQLGCPYIEEINKILMAIF

EAGIITKMTENEYEQLGKKKQTTSETEKELIPGVKKENRRVAKVSEDNEKLQPISIKMLQ

GTFYLLCIGNIFSGFILLAEILVYKHRKTYKHKKRRHRFVYLRKIRHSVASKFGAVVDAV

RRVYRRAMHDAFVATLEYLE

>TcasIR21a

MQRGLIVLKLCLTALALKSLDKRALQKSHEKSQLEKWEDKFLNRDPSFDQTASLVNLISK

VALDELSGCSATILYDKFTETSSDLLLEKLFRTFPIPYLHGQITDKYHMKVPKLQTSQDT

CTGYILFLKDVMRSKDVVGPQTNNKVVLVSRSSQWRVYEFLASEQSQSFMNLLVIAKSEK

IVSSSIARLICLALHLKFGTALAIYAPNGGKSAVYPSVIANVPKLGFRSAESVTSVITQN

GANLGIGGLYITDTRLKATDMSHIHSQDCAAFISLASTALPRYRAIMGPFHWTVWLSLTL

VYLFAIFPLAFSDKHTLRHLLDKPEEVENMFWYVFGTFTNAFSFFGKDSWSKTDKFATRL

LIGFYWIFTIIVTACYTGSIIAFVTLPVFPATVDTPEQLVRGKYTVGTLDKGGWQYWFEN

STDPITQKLLTRIDFVPDIESGLKNTTKAFFWPYAFLGSRAQLDYIVRTNFTTINKRSLL

HISSECFVPFGVSIIYNKNALYSKIIDQGVLQAVQSGIVDKIKNDVEWETMRSASGKLLA

ANSYGKSLKALTVDDRALTLDDTQGMFLLLGIGFLLGGASLLSEWMGGCLHLCKGNRNQS

ATSIQSNYRSHEVPTPREKLDSMQFNSFENHKIEEEIVEERNCIIHRQDDDDIEEHINRL

FDFEGVFGEANPDSRTGPEEELSFKNTTKAFFSLYAFLDSRAQLDYIVRTYFTSMNKRSL

LHISSECFVPFGVSIIYNKNALYSKIIDQGVLQAVQSGIVDKIKNDVEWETMRSASGKLL

AANSYGKSLKALTVDDRALTLDDTQGMFLLLGIGFLLGGASLLSEWMGGCLHLCKGKRNQ

SATSIQSNYRSHEVPTPREKLDSMQFNSFENHKIEEEIVEERNCIIHRQDDDDIEEHINR

LFDFEGVFGEANPDSRTGPEEELSEENGKK

>TcasIR76b

MGLFEIALAALCLNATCPGEEEPPEFPEVQYLAPDSNDRKTLFAQLTEQLKNENLIITTL

KNDRLSGTEKRNNTILGKGIAFDLLNILQDKFQFNYTLIEPKANVWGAEKFGVLDLLKDK

KANLSAAFLPVLTQYSNHISYSPSLDTGEWVVLMKRPKESATGSGLLAPFNLPVWLLILL

SLVVVGPVIYFIIYLQAKLCKDDNNKVFPLPACIWFVYGALLKQGTTLNPMTDSSRLLFA

TWWIFITILTAFYTANLTAFLTLSKFTLPITEPKDIGEKRYKWVTTKGNALEDTVTVNES

LTELGKILGQPQRYLYVSDSDILRNYVHKRNWMFIREKPIVEYVMYDDYKEKTRNQIEEA

KRCTYVITKFSVVSFSRAFAYSKDFKYKPLFDSTLVQIVKCHKCFSLLSRIQYLVESGII

KFKLREELPDTEICPHNLGNKERQLRNSDLLMTYEIVGGGFIISAIVFIIEVIIRRQKKP

KTKSLPLQNPNKHTFEINLNNNYEKFGHFPYSSKFVTPPPPYHTLFNPPHKSDNMKKRNF

NGREYWVYDSISGETKMIPMRTPSALLFQYTN

>TcasIR93a

MLLELVLSSAFVCVIRGDSFPSLLTTNATLAVIIDREFLSNEYEVIKHAIESYLVFAKRE

ILKHGGVNVQYYSWTTINIKKDVTAIFSIASCPDTWRLFRQARDANLLHMAISESDCPRL

PPDEAITVPLITRGEELPQLLLDLRTRQTYNWNSAFILYDDTLSRDQVTRVVKSITAQYS

NLRVNAAAISFVKLETRLPMDEIRRQVKEILSSVSIKTVGGNFLAIIGYELVELLMEYAK

MFGLVNTRTQWLYIISNTHFRHKDINRFRQLLSEGDNIAFLYNNTVNNDTCTGGIQCHCE

EILSGFTRALDEAILFEWETSSQVSDEEWEAIRPSKLDRRNSLLQGIKTFLLQRGQCDNC

TSWLMKTGDTWGREYQQNGTDSGGLISVGNWRPSDGPSMSDELFPHIVHGFRKRNLPIVT

FHNPPWQIIRSNESGAVSEYAGVIFELIKELSKNLNFTYTVELAKIGQEFSANLTKNEAQ

VVTNFIPDSILDMIRNKSVAFGACAFTVTEESKRLINFTSPISTQTYTFLVSRPRELSRA

LLFMSPFTGDTWLCLSASIVSMGPILYYIHKYSPVYEYKGLSKRGLSSVQNCIWYMYGAL

LQQGGMHLPQADSARIIVGAWWLVVLVLATTYCGNLVAFLTFPKIDIPITTIDELLAHSG

TVTWSMPKGSYLERTLKYTTEPRFRYLFDKKVEVGNFKNMIEDIENGKHVHIDWKIKLQY

IMKQQYLDSDRCDLALGLDEFLNEQLAMVVSQDTPYLEIINDEIKKLHQVGLIQKWLTDY

LPKKDRCWKNNRHIVEVNNHTVNMDDMQGSFFVLFLGFLLSFFITIGEKLWHKYVTKKKM

KIIQPFTT

>TcasIR64a.1

NKISLILVILSKTETYIIKSCLSNAIVDFAILANVAFSLRISCYKLFMHIKLIANVFYNQ

LDQVLNRNHYHLAVIIDSGCIDYADFAIQDKKYFYETYHWLVPTTPQNLNNSLNFLQKSP

LNINSDVNVAILNGEGTKWSILDVYNPASSHHGQFTVTKLGLCDETNGYQAKIAGNKYWS

RKNMTGVQFKSAVVVPDPSIKLNDYLTSDKNRQLHSMHRFQSVTVNYCREMYNFSLEIQR

TNSWGYLTPNGHFDGLVGLLERRLVDFGSSPLIYKLDRMPVIDYSYGNWVLRSTFIYRRP

KIIEASYKIFLRPLSRTVWICIVLMMVLLMLFLKVVFSREKRLLQKRNLVDSSWSFLFLF

TLGAFCQQGATCHPQLLSSRTLSIFVFLFCILTYQFYSASIVSYLLIDPPRKINNLKDLS

DSNLRAGIEDILIDRNYFVQTTDPVAIELFNKKIKFSNNNSGFYEPWDGLDLVKQGGFAF

HVETSTAYPIIEETFTNEEICELEEVQMYRTQPMHTNLQKNSPFREMMNYCMLHLVENGL

MYRLRKYWDARKPMCIESAKKFTFNVGLKEFSSGLIVLSYGILISLGLLLREVIVHKK

>TcasIR64a.2

MSPPLPFMILLSVLTQTHALLDINLIENYFTEKSIKSATVFGCFRKTEQLNLVKIFSRGS

SPISVLNLNQAGVYQSIKSNHQQIGVVLDGDCPESESFLITVSPGFTHIAPNVVFISVRS

TETXFDVKHHWLILSKSIQFLEKIKNAVVNINADIHVAVQSGTNWTIFDVYNPASEHGGS

LKYTRVGFYSRGRGYNAQTNEAKYWRRKDMTGVTFKTMVVLLVPFEGPLEDYLHNDDNRN

INTFNRFQNKLLRFCRDYYNYSMIVELGSSWGYPFPNGSFDGMVGAMEKKLIDFGSSPIF

VREDRARVIDYGRNTWSWKAGFLFRSPKSRTSIEIFLKPLSTSIWLITGVLATASIVILK

MVTTFERNRYHSTSETSWSLSFLFTLGALCQQGSPWVPKMACGRITAISIFLLSLIIYQF

YSASIVSHLLMKPTNKIRNLKDLTDSSLKVGCEDIIYNKDLFAHTTDKVLKDLYAKKIYG

KGNTSHFFPPEKGLDLVRQGGYAFHIEVARAYPIIETTFPDNAICELREVKLFKNTDLYN

TMQKGTPFRDMLESCFQRLAEQGILDREKKHWHPRKPECIQSSQAFVTFHVGLDEFYPAL

LVLLIGIVISLTVLVVEKQIHIAREKMEREKGVVF

>TcasIR64a.3

FQLRVLMERLFFLSVLAVIIYTTNCTDNHDIITSYIKEKSVKYATVFGCFTKKEKINLVK

IISHICPISVFDINRLNIENRMESRHFHTGIILDGDCPSAEKFLINCGRSYLFDVKHHWL

IVASSEKIREKFNNVILNINADINVIIPEKPSNWSIIDVYNPASQHGGVLNFTRVGFYNK

HDGYKIKYTGVKYWNRKNLTGVTFKSMVVVTYSKTXKNSAYTIFQLPVPFEGTLQHYLDS

DDNRDVNTFNRFHSRLISFCRDYYNFSLDIEVSKSWGYTNEDGTFDGMVGALERKIIDFG

SSPLFLREDRARVIDYGRNTWILRSLIKQQFRIISNWGFSAAFIFRNPKVRTSLEIFLRP

LPSSVWLITGLLAIVSIIILKLATSFERRRYVYDVETSWSISVIFTLGAFCQQGSPSTPK

MACGRIATFFIFLLSVLIYQFYSASLVSHLLNKPLTKIKNVRDLLLSPLKAGCEDILYDR

DYFLHTTDKVAKELYAKKILGKSNSSNFHTPEAGLKLVAEGGYAFHVETATAYPIIESTF

QDQAVCELREVPLFRTQPMHANFQKKSPFRDMFDTCFQRLAEHGLLVRERKHWHPRKPEC

IQSSKSIRFNVGLDDFYPALVILLVGIVASLLILVIEKEFRILTENPA

>TcasIR75q.1

SFLGTILTVYKQLAEKKIVLNVLTNHWKINQTKLSQHTFLVGDTLCPQFNSLLSHVSKFF

CYQNSQQTLGQIITSSXKWLVFDQNSTVNTNDLLLDSNFAVASQISNGRFHLKLCYKRAP

NETIKFNEIGVFSNGFEYYNHFIPTRNRSDLSGVNITVSYVVTKPDYPFDVEDYRFRHLE

AFSKLSYAMVYPMLEMLNCTKKFIQRSSWGYKGANETQFVGGMFGDIQNGTAEIGGTVSF

YTVDRMSVVDYLSVTTPSDLKFILRAPPLSYVNNLFTLPFDTKVWYCLYFIVGVTVLILY

VIVRCESTYENALERRNNIDNIKPKFFDVVMLQIEAITQQGSENEPKTMSGRIAVFIVFL

VLMFLYTSYSANIVVLLQSTSANINTLQDLLNSKITLGVEDVVYSHHYFETQTEFTRKSI

YEKKVAPKNQKSNFMTTEMGIEKMKDEFFAFHVETTAGYKQIMDTFQEHEKCGLIEIDYL

NVLYPSITIRKNSPYKEIVKVNFRKIYESGIRHRQLNRIYYKKPHCVGKGGSFKSVGIVD

IYFSVEIFAIGCFMALWLLLLEVLFKKKIKFLVQ

>TcasIR75q.2

MKILIVFICLLINETTQNNFTDNLIVNTFNFIKILNVPVKISAHICWTRGKFDSLLMKLY

XTVLANTIHFIKSISDKYNTNLIKNVSPKYANPEHQLFIIDLKCNDSLSVLQQAEKFKLF

KSPFKWLLLGNSESLPNLYFGTDSQIFVTEPRSQLDDIKTIYKYSPMVPRFVQHSFDRFY

TNTKRTNLMGTTIKISYVITNLDSLNHLWDYRLQELKKKLYHFLICRNSHIDAINKLNYI

LVHNLMDFLNASRQFTMQPTWGYKNSTTGLYSGMAGDLQKGLADLGGTPLFFTPDRIDII

DYIAATTPTYMKFIFRAPPLSYVTNVFTLPFDSAVWHYCFVMVAVVVVCIYVIVVWEWKE

TKFEEKDTHSHIDTLRPNIFDVVMFEIGAITQQGTNAEPKSNSGRIITIFSFLTLMFLYT

SYSANIVALLQSTSDSIKNLEDLLNSRIKLGVEDIVYAHYYFENAQEPVRKAIYQQKVAP

KGQKPNFMTAEEGIRKVQQGFFAFHVELSTGYKIIGEVFQEGEKCGLKEIEYVNLIEPWL

ATQKKSPYKEVMKIGMRKMHETGVQNREIRKIYTRKPQCHSGGSNFGSVGLIDCYSAFLT

FGVGIAFAFLLFVMELIVRRYFIRREKERLK

>TcasIR75s

IVLPMINDLIEHFNKTQIILAYLCDKNGTNLLLIRNNNNTNFRRLSGSEPLFXKKLYQVN

VLSPNSRDMPYPTPPAFLTYVLDAGCSNTKQLLLLVPVITHXLIFGNNILKASEQKQFAT

PFKWIVYYNNPVELSFFIDEYFTKTNILVDSDVTLATINPTSGTFDLNKIYKRKINGSII

IENIGIWGRGLGVTDTGYEKITYKRRRNLTKTVLKSCIVITNNDSLNHLTDKRDIHIDSI

AKVNYVLVQHLSDTINASLEYSVRGTWGYKDNKSQWSGMIGELTRNEADIGGTALFLTSD

RIRVIDYIAMTTPTRSKFIFRQPKLSYVANVFTLPFDASVWASVCGLLVIIAGLLYVVVR

WEWKKKDYVQVVVFFAFWVDFPSSVFCRTNRTSRKFTILGSXVFITFGALCQQGSSSVPF

SIPGRITLIFLLVSLMFLYTSYSANIVALLQSSSSSIQTLQDILNSRLDVGVDNTVFNHF

YFPNATEPIRRAIYQQKVAPPGQKPKFYPIEEGIRKMRQGLFAFHVETGPGYKFVSEIFR

EDEKCGLQEIQYLQVPDPWLAIQKNSSYKKMLKVGLRLLQENGIQEREVGLIYTKKPQCL

ARGSSFISVGLVDCYPAAVVLAGGIGAALAVLILEIYVHQRFVGFLL

>Tcas971730.2

MLRAILLVLILFMSVHSYQNFQDHKDSLNVGLILPYTNFGVREYTRAINNAVSGLHRSRG

QRLNWLKKYNFTPKNVHYVLITLTPSPTAILKSLCKEFLSVNVSAILYLMNYEKYGRSTA

SAQYFLQLAGYLGIPVIAWNADNSGLERRASQSSLQLQLAPSLEHQTAAMLSILERYKWH

QFSVVTSPIAGHDDFIQAVRERVSAMQDRFKFTILNAVLVSHHRDLAALVDSEARVMLLY

CTSQEAIDILTAAKDFHLTGENYVWVVTQSVIANPLEAPGQFPVGMLGVHFDTSSSSLVN

EITTAIKVYAYGVEDFTNDLANAGRSLNTQLSCEGEGAARWNTGDRFFRVLRNVSVEGEA

GKPNLEFTQDGVLKAAELKIMNLRPGVSKQLVWEEIGVWKSWQKEGLDIKDIVWPGNSHT

PPQGVPEKFHLKITFLEEPPYISLAPPDPVTGKCSMDRGVLCRIASDADITEVDTTLAHR

NGSFYQCCSGFCIDLLQKFSEELGFTYELVRVEDGRWGTNENGKWNGLIADLVNRKTDMV

LTSLMINAEREAVVDFSVPFMETGIAIVVAKRTGIISPTAFLEPFDTASWMLVGVVAIQA

ATFTIFLFEWLSPSGFNMRLSLNQSNDTSHRFSLFRTYWLVWAVLFQAAVHVDSPRGFTA

RFMTNVWAMFAVVFLAIYTANLAAFMITREEFFEFSGLDDHRLSRPYSQKPLIKFGTIPW

SHTDSTIAKYFKEMHAYMRQFNKSTVHEGVDAVLSAEMDAFIYDGTVLDYLTSQDEDCRL

LTVGSWYAMTGYGLAFPRNSKYLKMFNKRLLDFRENGDLERLRRYWMTGVCKPGKQEHKS

SDPLALEQFLSAFLLLMAGILLAALLLFLEHLYFKYVRKHLAKTDRGGCCALISLSMGKS

LTFRGAVYEAQDILRHHRCRDPICDTHLWKVKRELDISQMRCKQLEKELEAHGIKPPPPC

KR

>Tcas969654.1

MFVFVIFALNWLTIRADLWSSNNPTVFNIGGVLSSNESEYYFKETIAHLNFDSQYVPKGV

TYYDTAILMDPNPIKTALNVCKYLITSRVYAVVVSHPLTGDLSPAAVSYTSGFYHIPVIG

ISSRDSAFSDKNIHVSFLRTVPPYSHQADVWVEMLKHFNYKKVIFIHSSDTDGRALLGRF

QTTSQSLEDDVEIKVQVESIIEFEPGLETFKEQLSDMKNAQSRVYLMYASKTDAQVIFRD

AAEFNMTDAGYAWIVTEQALVANNIPEGILGLRLVNATNEKAHIKDSIYVLASALRDLNQ

TKEITEAPKDCDDSGQIWETGRDLFDFIKKQVLMNGETGKVAFDDQGDRINAEYNIVNIQ

RKRKQVTVGKFFFNRTSNKMRLAVDENNILWPGRQHVKPEGFMIPTHLKVLTIEEKPFVY

VRKLVEPQDVCTAEEIPCPHFNATQDLAGSYCCKGYCMDLLKELSKKINFTYSLALSPDG

QFGNYIIRNSSGSGKKEWTGLIGELVGERADMIVAPLTINPERAEFIEFSKPFKYQGITI

LEKKPSRSSTLVSFLQPFSNTLWILVMVSVHVVALVLYLLDRFSPFGRFKLANTDGTEED

ALNLSSAIWFAWGVLLNSGIGEGTPRSFSARVLGMVWAGFAMIIVASYTANLAAFLVLER

PKTKLTGINDARLRNTMENLTCATVKGSAVDMYFRRQVELSNMYRTMEANNYNTAEDAIE

DVKVGKLMAFIWDSSRLEFEAAQDCELVTAGELFGRSGYGIGLQKGSPWADDITLAILDF

HESGFMESLDNKWILQGNVQQCEQFEKTPNTLGLKNMAGVFILVAAGIVGGIGLIVIEMA

YKKHQIKKQKRMELARHAADKWRGCVEKRKTLRASATTQRRIKSNGVNDPATISLAVDKY

QRIGGPERAWPGDSDIRQRRVEDSGGVQPVPRYLPAYTSDVSHLIV

>Tcas968786.2

MSVTNVKNIVLLTFFTISVSATGDKIPLGAIFEQGTDEVQTAFKFAMLNHNQNVTARRFE

LQAYVDVINTADAFKLSRLICNQFQRGVYSMLGAVSPDSFDTLHSYSNTFQMPFVTPWFP

EKVLAPSSGFLDYAISMRPEYHQAIIDTVRYYGWPKIIYLYDSNDGLLRLQQIYQGLVPG

SESFQVSTVRRISNVTEALQFLRGLEEQSRWEHKYVVLDCSADMAKEIVVSHVRDIALGK

RTYHYLLSGLVMDDRWESEVIEYGAINITGFRIVDSSRKHVKDFLDNWKKLDSTGSQNTG

RESISAQAALMYDAVFVLVEAFNKLLRKKQDIFRNNMRRGQIFNNGSKGLDCNASGGWVI

PWEHGDKISRYLRKVEIEGLTGEIRFSEDGRRQNYTLHVVEMTINSAMVKVAEWSDETGF

TPVAAKYIRLKSNAQIERNRTYIVTTIVEEPYIMLRSPEPGETLSGNDRFEGYCKDLADL

IAKHLGITYELRVVKDGNYGSENHEVKGNWDGMVGELVRNEADIAIAPMTITSERERVID

FSKPFMSLGISIMIKKPMKQKPGVFSFLNPLSKEIWVCVIFSYIGVSIVLFTVSRFSPYE

WRLLHLTGEHRDPSGQHSTHNSMANDFTMLNSLWFSLGAFMQQGCDIAPRSISGRIVGAV

WWFFTLILISSYTANLAAFLTVERMVAPINSPEDLASQTEVEYGTLYHGATWDFFKRSQI

TLYSKMWEYMNSRKHVFVKSYDEGIRRVRTSKGKYALLIESPKNDYINEREPCDTMKVGR

NLDAKGFGVATPLGSPLRDAINLAVLNLKENGELTKLMNRWWYDRTECIHDKQDAARNEL

SLSNVAGIFYILIGGLMIALAVALIEFCYKSHTEAVRAKIPLSDAMKAKARLTIGVGRDI

DNGRYYTPANQIAGANEQEQAHSNTHTQV

>Tcas966884.2

WGSWGATLGLAGLLAVAVALPPVVKIGAIFTEDQRDSATELAFKYAVYKINKDKTLLPYT

SLVYDIQYVPRDDSFHASKKACNQVQHGVHAVFGPSDPLLGAHIHSICDALDIPHLEARL

DLDTDIREFSINLHPAQHLLNTAFQDVMAFLNWTKVAIIYEEDYGLIKLRELVRSPHNGD

LEIHLRQADPESYRAVLKEIKSKEIHNIVIDTKPSNMQHFLKGILQLQMNDYKYHYLFTT

FDMETFDLEDFKYNFVNMTAFRVVDVTDLSVQEVLRDMARFQANINADSKLNSTYLQAEA

ALIYDSVFVFAIGLQTLEQSHTLKLSNVSCDKEQPWLEGLSLINYINAVEFKGLSGPIEF

KEGRRIQFKLDLLKLKQHALVKVGEWRPGAGVNITDRAAFFDPGTMNVTLVVTTILEQPY

VMLRTQTNVVGNERYEGFCIDLLKEIASMVGFEYRIELVPDSKYGVIDLETGEWNGIVRQ

LMDKKADLAVGSMTINYARESVIDFTKPFMNLGISILFKVPTDKESAFFSFFSPLGFDIW

IFVGGAFFMSSFTLFTLARFTPYEWVYPQPWKRSKYLVNQLSMSNSFWFIAGTLLRQPSG

VNPQVPTSQQARLFSFMNPLAMDIWMYVFSAYVLVSITMFVVARFSPYEWHNPHPCDMEN

ELVENQFSLANSFWFTIGTLMQQGSDLNPKATSTRIVGGIWWFFTLIIISSYTANLAAFL

TVERMITPIENAEDLAGQTEIPYGTLESGSTMTFFRDSMIETYKKMWRFMENRKPSVFVP

TYEEGIQRVLEGNYAFLMESTMLDYTVQRDCNLTQIGGLLDSKGYGIATPMGSPWRDKIS

LAILELQEKGEIQMLYDKWWKNTGETCSRNEKGKESKANSLGVDNIGGVFVVLLCGLAFA

VIIAICEFCYNSKKNALTEKRSASAPHQSLCSEMGGELCFALRCRGSRQRPALRRQCSKC

LPGATYVPAMLDIPPHPPQPPSRPPPTNGLSAHVCPEDTSLRDRMMIPLELQHHMQPQHP

LHQQLDN

>Tcas966711.2

MSHGAANPPPAISAPIFAEQITLKTTCAIFTEDQKDSSVELAFKYAVYKINKDRVLLSNT

TLVYDIQYVPRDDSFRTSKKVCRQMEFGVQAIFGPSDPILGAHIQSICEALDVPHLEARI

DFEPLSKDLSINLHPSQEHMNKAFKDLMTFLNWTKVAIIYEEDYGLFKLQELVKAPAAAR

TEMYIRQAGPTSYRQVLKEVRQKEIYKLIVDTNPRNIQKFFRAILQLQMNDYRYHYMFTT

FDLETFDLEDFKYNSVNITAFRIVDVDDPQVKESLEVMEKFQPIGHAILNKSGIIQAEPA

LMFDSVYVFAKGLAAMGSIKPMNLSCDVEKPWDDGSSLYNYLGDDDLRGLTGNIEFNGGK

RSNFKLDLLKLKKEEIRKVGQWTPSGGVNITDPNAFYESHAPNITLVVMTREERPYVMVK

DEKNLTGNARYEGFCIDLLKWIAGQVGFQYTIRLVPDHMYGVYDPDTKEWNGIVRELMEK

RADLAVASMTINYARESVIDFTKPFMNLGIGILFKQSSKSEPSRLFSFLNPLAMNIWLYM

AGAYVLVSITIWIVARFSPLEWKEPELHEHADGRTLEILENGFTIGNSFWFAIGSLMQQG

SDLNPKATSTRIVGGIWWFFTLIIISSYTANLAAFLTVERMITPIESAQDLADQTDIAYG

TLEGGSTMTFFRDSKIGIYQKMWRFMESRKPSVFVKTYEEGVQRVLEGNYAFLMESTMLD

YAVQRDCNLTQIGGLLDSKGYGIATPKGSPWRDKISLAILELQEKGVIQILYDKWWKNTG

DVCNRDDKSKESKANALGVENIGGVFVVLLCGLALAILVAILEFCWNSKKNAQTDRQSLC

SEMAEELRFAVRCHGSRQRPALRRSCTRCSPATTYVPAALDLPHINGRRLYAGGTSTFAC

TSSAIRDCCGGPSNSRPQRAATLNYRATPQCPDLAKKSATLGRNSGFCRDTCDIVQSGLQ

NDVPDKILWKFDCDIGSPNNPDVVTRTLPDAIVSRTTTL

>TcasGluRK1

IWLLIFVAYNYLFCLADDKNKITVGAFFEREDVQSKAALNYAIDTTNMMQQHLKYALKTQ

ILAQNDSFYCCKLWSGLAAIFASKPIFESLSNRLEIPFILTKWRPASYSNKQTTVNFFPD

SYLFSHGLAIIVKNLQWKNFVLLYDSDKGLVKLQQILKLNNFNSGSVIVRQLGPGPDHRP

LLKEIRALNHNRIILDCDTENIIEILKQAKEVNLMESSYFNYFLTSVDAHTLDFSVLNTT

ANITTIRILDFTQXNQITRLGYKLXRFFGITLIVIXTETALIQDGIHSFITSVNTLHVTE

PIVPSPMACDQKWSHGFRISSFMRVVIVKFWVCGPIGFDSSGRRLNFTIFVVEGNRENVV

AKWRPENPEILIYMRGENDSFDALVKNMQKSVLIISSRLGPPYLMERKPRFEGEILTGNS

RYEGFSMDLIDAIAGILGFKYEFRLAKDGKYGNYDPETKSWNGLIKDLLDRKADLAICDL

TITHQRREVVDFSMPFMRLGISILYKKAEEKDVNIFAFLEPFSPEIWIYTATLYLVVSVI

LYLVARMAPGDWENPHACNPKPEKLENIWNLKNCLWLTLGSIMTQGCDILPKGISSRLAT

SMWWFFSLIMTSSYTANLAAFLTMERLEPTIDSAEALAKQTKIKYGTVEGGATQAFFRES

NYSTYQKMWTTMIQAKPGVFEKNNADGVKRVQTTKNRLYAFLMESSQIEYEIETKCDLKQ

VGNWLDNKEYGIAMPIDYPYRSAINTAILKLQEEAKLTELKDKWWKKMRDEPSCPVRTLG

KSSTELALDNVGGVFLVLGVGMAVAFVLAILEFLWNVRNISVEEHMTYFEALKVELIFAL

NVWVTKKRTKPKINSLNCVLXRVIKNQTRKSCLLIKNIVFINNMNKYDKK

>Tcas968606.2

MKIVYQNVIWWVFFAFYNYLSLAQDEPLTVDVVGFFDEKNGLSEIAFQTAISNLNIMKNS

IRFNPLSTVVNTSDSFENSKFLCETAEAGKVGGVFCATSAKIAPIIESVSDNLNIPAVQV

AWRPSATYTDMLVNVYPLPKLLFQGLGAIVRNLQWRSVVVFYESAENLIPLQDVLKTQDY

NGGNKYNSLMLKELGPGPDYRSALKQIQNKSEYRIILDCKTENIVTILRQAKELKLLEPH

FSYFLTSLDAHTVDFKLLNTTANITTVRIFDPASDNFQYAISNWNNYVKKMNIPGVNLDP

YSVKTETALMHDAVHMFLKCITDLHATGKSVKPTKLSCENVDKWTPGFDIASFIKAYTHD

TDGLYSTTAPISFDNLGRRTNFSIFVVEGNRDDVVAKWNPSDPEVLQFLKSEEDRNKELE

RKWSEGIVTTRIGPPYLMVKEQKSETDLLEGNNRYEGFSMDLIALLAKDLNIKFRFEVLK

SGQRGAYDKTTKSWNGLIREILDRRAELAICDLTITPDRREVVDFSTPFMRLGISILYRK

AEAKEADMYAFLDPFSLKLWMYSATLYLALTVVLFFISRISPQDWENPHPCEQEPEELEN

IWDMKNCLWLTLGSIMNQGCDILPKGMAPRLAASMWWFFTIIVTNSYMANLAAFLTNERS

QSEINSAEDLAKQTKIKYGTLDGGSTQGFFRESNYSLYQRMWTAMEQAKPSVFEQSNDAG

VARVQNEKNRLYAFLMESSTLEYQIQTKCDLKQVGNWLDSKGYGIAMPLDYPHRSRINEA

LLRLQEQGEINRLKDKWWKEERKDPLCPKESEDQDANKLALQNVGGVFIVLGVGVALAYI

VAVLEFLWNVRSVSVDEHISYMQALKVELLFALDVRKTKKRAKPEVPESSSSSRSPSMAR

SFLQSAGSFLRLDKMNQMETPGSSRHTSRPLE

>Tcas974911.2

MEWLTQLLLVLPLMKFSNTLPDVIRIGGLFHPADDKQEIAFRYAVEKINSDRMILPRSKL

SAQIEKMSPQDSFHASKKVCHLLRSGVAAIFGPQSAHTASHVQSICDTMEIPHLETRWDY

RLRRESCLVNLYPHPTTLSKAYVDLVKAWGWKSFTIIYENNEGLVRLQELLKAHGPYEFP

ITVRQLGESSDYRPLLKQIKNSAESHIVLDCSTERIYDVLKQAQQIGMMSDYHSYLITSL

DLHGVDLEEFKYGGTNITAFRLVDPDGPEVRKVVREWNLSEAKNKKGEISSIIRAETALM

YDAVHLFAKALHDLDTSQQIDIKPLSCDAVDTWPHGYSLINYMKIVEMRGLTGVIKFDHQ

GFRSDFVLDIIELNKEGLKKIGTWNSTEGVNFTRTYGEAYTQIVEIIQNKTFVVTTILSS

PYVMRKEASEKLTGNAQFEGYAVDLIHEISRVLGFNYTIRLAPDGRYGSLNRETKEWDGM

IRELLDQKADLAIADLTITYDREQAVDFTMPFMNLGISILYRKPIKQPPNLFSFLSPLSL

DVWIYMATAYLGVSVLLFILARFTPYEWQNPHPCNPNPDHLENQFTLFNCMWFAIGSLMQ

QGCDFLPKFSPYEWDNPHPCNSDPDVLENQFTLLNSLWFTIGSLMQQGSDIAPKAVSTRM

VAGMWWFFTLIMISSYTANLAAFLTVERMDSPIESADDLAKQTKIKYGALRGGSTAAFFR

DSNFSTYQRMWSFMESQRPSVFTASNVEGVERVVKGKGSYAFLMESTSIEYVIERNCELT

QVGGMLDSKGYGIAMPPNSPFRTAISGTILKLQEEGKLHILKTRWWKEKRGGGACRDDTT

KTSSTANELGLANVGGVFVVLMGGMGVACVIAVCEFVWKSRKVAVEERSSLCAEMANELK

FAMRCQGSTKPIRKKGRPCDASTGVDDARFHPLGSYTSYGFVVNKEPIN

>Tcas974933.1

MATFSILFLILLEACASPENPKALKVAFFLNENAALDELAITSATNYINNYAATQYALNF

VLAPRIYRIKKSEIYNVGNLACDALREGIAAIFGPENGEANEIIQSMALSLEIPQFQTFW

NPNFATYAGLGTANKKEIFNFNLYPSPSVLSKAFATLVRENDWRSYTIIYENDDGLVRLQ

EVLKALSPNNPLVTYRKLGPEPDHRPVFKEIVASGALHIILDCEADHTIDILSQAKEVKL

FEEYHTYLLTSLDAYTIDFRQLGEIKTNVSIVRMLDQKVVDTVIGNWELVDSERKLKIPN

KLIKVKTAFLFDALNLFITAYSNLDQEQEMDVRPQSCDTNEISSHGYRLSAFIPLLNMTK

GMLGEPISGSLNFNSLGQRVSLKLEVLELRKDEFRVTGIWDSGTPHSIYSTITSADREKE

LEQQLKGRTFRVVSRIYPPYLSRKPGIDSSVMSGNNAFEGYAMDLMKGICELYECNYVFE

LVPDNNYGKYDPKTKEWNGLIRHLLDRKADLAICDLTTTYERRKAVDFSNPFMTLGISIL

YTKIVKEPPDLLAFTNPLSLHVWLYMVTAYMVISMIIFLVARLNPNEWENPHPCNPNPEE

LENIWNIKNCFWLTLGSIMQQGCDILPKGISTRMVAGMWWFFTLIMISCYTANLAAFLTQ

SRMGPTIQSAEDLAAQTKIKYGCLKDGATASFFRDTNVTTYHKMWVAMETADPSVFETSN

DDGVKRVISKKGKYAFLMESSSIEYEVEKHCELVQVGNRLDTKGYGIAMPTNAPYRTSIN

QAILKMQEMGRLQRLKEKWWKEKNKANTCKKDEDSKTDSANELSLAHVGGVFVVLVVGMS

IAMVIAVCEFLWHVRKIAVTQHVALKEVFLKELRFAMDIWCRQKPANPAANLNLSRETLR

QGD

>Tcas974901.2

MPKPRHETEQKAFHLATDLINAKYKDSSIRLIPDSHLIDNYNAYTTYLTTCELLQKGVIA

IFGPSSIHSSPAIQTILDRKEIPHVETYFDRKLSRHDCLLNLHPHPSVMSQAYLEIVNKW

GWRSLVVIYDSEESLAKLGLFAASCKQRVTLSRLELDMYDTFRTSLTSIKKTGETNFILE

CSVDILEAVLKQAQQVGMMTERHSYIITKLDLQTIDLAPFQYSEANITGFRIFNPENAEI

MSLADQIYTQEKYKGIPSGWLLRHQTALLIDSVDLLHQAVLDLTLSEQVVIQSQTLYCNT

SNNWDSGHTIVNYMKGQTIKGLTGVVHFDNEGFRRDFTLDILELSLGGLLRIGAWSFFSG

LSLNRPPNLSKVKIVDDANLVNKTFTVITCLTTPYGMLKETTQQLFGNDRFEGFGIDLMD

ELSKMLGFNYTIIIQEDGYNGNYNQTTGEWNGLIGAILSGKADLAIADLTVTAEREAVVD

FTLQFMNLGISILYKKPKPVPPSLFMFVSPFSYTVWILLVVTYFLVSMCFFVMGRLSPSE

WTNPFPCVEEPEYLINQFSIRNSLWFTIGSLMQQGTELAPIGISTRTGAGVWWFFTLIMV

SSYTANLAAFLTVETLVTPFSNVKELSEQTEIKYGAKRGGATANFFKNAGNDSVRSRIWH

FMATHDEEMTESNDEGVERTEEKHYAFFMESTTIEYVIERHCSLASVGAPLDDKGYAIAM

KKNSSYRNDLSAAILRLQETGKIAQLKEKWWKEKRGASNCGAQKSESAATPLNLQNVGGV

FLVLFLGTGLGFCISFVELALRVYSTTKKTDQQFRKELIEEIKFFIRFKKNVKSVKPETH

>Tcas966620.1

MRCLGLTVFLLIFPNFLGQEQDRKEIFLGGIFTEPPDVDDSVLSDEEAFNFAIDIANREY

SDVKFTSVSEESDLRTNGPFDSRLQACSLVNQQALVIFGPKNAEEIDIVQSICDNKDLAH

VITRWVYSSADFRSVINFYPHSAYLTSAYFSVLKLWNWKTLTVFYEDNESMLRLGDLLNL

AKNEGIIVTVKQLYEGLDETPIYRTTLKEAVRSGQKNFIIDCKIESLEEVLKQAQQVGLM

TKDYNFFITNLDLQTINLEPFQYSEANITGIRILDPLNEMFHVKAGAIMRQKPNFNLTKM

RTETALLIDAVSVITQVISRKLSIKEMEMTEISCNSPKSSRHGYTIANHVKTSKFDEMTG

RIEFDGNGVRSNFDLDVIELTQNGISKIGTWNMSKGLVITPHKDEDIVEDPLSLRNKTFK

VITCLTDPYCMLKENSGQLFGNDRFEGFAIDLIHELAQMEGFNYTFIIREDKSNGDKNKV

TGEWSGMIGDVMHGVADLAITDLTITAEREEAVDFTSPFMNLGISILAKKPGNAPPSFFS

FADPFALDTWIMLALAYIAVSVSFFVLGRICPDEWTNPYPCVEEPEFLINQFSLSNSFWY

AVGSLMQQGTELAPIGVPTRMVAGMWWFFVLIMVSSYTASLAAFLANENTITLFTDVESL

VQNYEEKGIRMGAKRKGATEGFFRGKDSETYKIIAKYMEEHPDDMVGDNKDGVKLANKET

YAFFMESISIEYETQRHCDLQQYGGLLDDKGYGIAMRKNSTYRKTLSTAILKLQSSGQLD

NLKRTWWEEKRGGGQCLDSGDDATPALDVRNVEGVFYVTIGGTLCAIVLIFFELFLSLLK

ISKKYKISMREALNNEKKAFLDFNSNVKPAPKAKSKSGSKSSGESGKSNNNTGAPTYGFI

PTITKDTLDE

>Tcas966528.1

MLLLVITISLYFHKFSQAETLKIGAIFDTDDPIKERAFHHAIHQIEPIHGRTIEGLVKNV

PPNDPFEAMLAACHLIESGAVAILGPTTHENAHMVQTVCDNKDIPLLDVRSVAHPQNSIN

FYPLQQILTQIYIKLLEAWNFENFVILYENDDSLIRLAELLKFYGNGHRMVVRQLDKYQN

GNYRPTLKEVWRSGATHFVLDCSTDILEEVLHQAQQVGLVTNKQFYIITNLDFHTLDLTS

FQYSETNITGMRFIDPDSDEIQNLGLTLYRNDFTNTEFGFIEAWKVNLEMALIIDAVTMF

GEVLNRLPKDFAIPSIDCASDKAWTYGTTLTNLVKSVKYPGYTGLIQFDNFGLRSAFGLE

IIELKEGGIIKIGNWNYSDGLNINRVYPPDPPPLVEGSLVNRTFIVITCLTEPYGMRRDS

EVPLYGNERYEGFGIDLIAELSKKLGFNYTFIIREDKKNGEFDESSGEWTGMIGDVISGK

ADLAITDLTITSERESAVDFSTTFMSLGISILYQKPKKALPSFFSFADPFSLTVWKLLAA

AFFGASIALFILGRISPSEWQNPYPCVEDEFLVNQLSLRNCVWFMVGSLMQQGSEIAPIA

FSTRMVAGMWWFFTLIMVSSYTANLAAFLTTESPDLPFKDVFELVQVAEKKGIKFGAKIN

GSTEKFFLDSKHVDEYQQIYKYMKNHEDEVMVNDNKDGVHKAEHEDYAFFMETTSIEYET

QRRCGLTSVGHSLDEKGYGIAMRKNSSYRMALSTAILKLQEEGVLAKLKRKWWEEQRGGG

LCPQGEKSTEGTPLNLKNVEGVFCVTIIGTVLSCVLVFVEMAVHTFKKSLRVKKPFKVLL

MDEMRFYFRTSAMLKPVTAPKPEPYGFITS

>TcasIR8a

MVISENLDKTTANRLKAIRPIPNNFAIVATSSNMEELLQTALDENLVTLPERWNLVFLDF

QYQQFDKKRLKNMPINLLHMDEEICCRFLQSEKCECPHDFNLQENFLSLATNTLAKILKT

LTMENLLRADLNCDDSRYSEATRTRFYELLQQEVDSNDLVFKENFGLHVNINGVIETGDE

KVAEYNYKTGVTVLDGKKVEPITPFFRIGITHALPWSYKETDSSGNTYWTGYCVDFTEEL

SKLMGFGYEFVEPKSGTFGKKRDGVWDGVVGDLATGETDLAITALIMTADREEVIDYVAP

YFEQTGITIVMRKPVRKTSLFKFMTVLKLEVWLSIVGALIVTGFMVWFLDKYSPYSARNN

KKAYPYPTREFTLKESFWFALTSFTPQGGGEAPKALSGRTLVAAYWLFVVLMLATFTANL

AAFLTVERMQTPVQSLEQLAKQSRINYTVVKDSDTHKYFINMKHAEDTLYRMWKELTLNA

STDDTQYRVWDYPIREQYGHILLAINDSNPVANASEGFRIVNEHTDADFAFIHDSSEIKY

EISKNCNLTEVGEVFAERPYAVAVQQGSHLQDEISKTILNLQKDRFFEQLQAKYWNHSGK

GSCPTTDDNEGITLESLGGVFIATLFGLALAMITLVGEVLYYRRKSKIQNSETKKPKTVQ

TSENWKTDTLMPVSLINKDKQSVTIGTEFKPVNRNRDLSEFGHITLYPRARNRITQTSNE

>TcasIR25a

MASSSAIIYRIAIYSRIATAHLNYSDFLNNVLTETHKMLKLVAFILYCTNLANGQTTQNI

NVLFVNEEGNLVAEKAVDVATNYIKKNNKLGVNADPVKVVGNRTDASGLLDSLCSSYNEM

IANSMNPHLVLDTTMTGLASETVKSFTAALGLPTISASFGQEGDLRQWRNIDENEKEYLV

QISPPADVIPEIIRSLVLSKNVTNAAILFDDSFVMDHKYKSLLQNVATRHVIAPIKEADK

IGDQLRQLRKLDIVNFFILGSFENIKRVLDAADSVGFFNRKFSWHAITQDKGELKCNCRN

ATITLAKPLIDAQYQDRLGLIKTSYQLNAEPEIAAAFYFDLALYSFLAVKEMIADGVWKR

NNATNYITCDDFDGKNTPRRAGLNLKKYFSKEVSETPTYGPISIVSNGYSFMEFTMQISA

VGVRESSSDKSVPLGSWKAGYDNNLTLVDPQIMKNYTADVVYRVVTVEQKPFIIKDETAP

KGYKGYCIDLIQRISEILNFDYEITPVGDQKFGNMDENGKWNGVVRELMEKRADIGLGSM

SVMAERENVIDFTVPYYDLVGITILMKLPKTPTSLFKFLTVLENEVWLCILAAYFFTSFL

MWVFDRWSPYSYQNNREKYKDDEEKREFNLKECLWFCMTSLTPQGGGEAPKNLSGRLVAA

TWWLFGFIIIASYTANLAAFLTVSRLDTPIESLDDLSKQYKIQYAPLNGSSTMTYFERMA

NIEAKFYEIWKDMSLNDSLSEVERAKLAVWDYPVSDKYTKMWQAMKEAGLPNTLDEAVKR

VKDSRSSSEGFAYLGDATDIRYLEITSCDLQMVGEEFSRKPYAIAVQQGSPLKDQFNTAI

LQLLNRRELERLKEKWWSKNPEAKKCDKQEDQSDGISIQNIGGVFIVIFVGIGLACITLA

FEYWWYKYRKGGKVVDVQAKHSDVATKINDGFHAKINKLYPRSRF

>TcasIR144

MQVSKILLLSSLLLNRDETSKCLDAIFKQPVVVLRGVPKNLQNFDAWKPETYLILAPNAT

VLEQMLEKWSTIESFNPRAKFWLLTHWHEIKPKTLTILAKFYIVNVAIVTRTGQVFTYYP

YKYENIAQPDTKPVLLGQCDNVPSFPDKLPKFWRNTTVQVLTKCLLPYVDCSDLDQGLET

QIFDLVQEFLKFKVRRIFDKSFKFGLAKINGSYSASFRFLQEREVDMAMGSFRSVGSTQF

RDFEFSTNHMEDKLVWVVPKARPMVHWVRLVKIFEPSFWGLLVVLTVAMARVFEKMARFT

DEPMGIYRKSGFRVAVLILIGSYLKKTPKRFEMRIIFIFWIYFCMVLNIVFNSNLTNVFF

GTFNTFQVNSFDDIIKSNLEMGLTDDVMHILSQEQNWPEITSTKVISSCAFGPACLNRTI

FQRNLVCCWGERSIKFRMAKFYTTQVHYVDDHLLFFYLLFYFVKGYPIVPQISKMIVQLK

SAGFVQFIKSKVDKLEPRQGNELTTKILTLKRLEGPFYFLLVGWVGGIMIFGYEVVTYER

KRRKKVRQEVTKILKKKKMRQNEKVKILEI

>TcasIR41a.1

TKMLFNNFCINILVNFIINNYHKNSRCLLIFTDGDFDYKGEIPTVRIKATNGSFNSYLIF

NYHGCQSVIIYTSNVTALLIKFETEIRLKMERFNERKFLIVPQNPSEDFDKFFNLKQLYF

ISDLLLVLPTHNDTIFDLKTHKYVGVIDNNEPVLLDRWFSQNQSFLFGKNLYPNKLQNQL

GRPLKMATFTYEPYSIIGNVFEQFFENDFILQGKSVGEHHGSELMSAVQFALKYNMTPVP

VINEKDYWGDIFPNWSGNGLLGNLVDDKADVGFSALYTWEFCYHFLELSKPLVRTGITCL

VPAPKLSERWLTPLFSYSSYLWFCIILTLVIAIFVLSLVLFCYNHNKTLNLNYPLKRKTT

YIHFLESAVTIVLKPVFQQSLTLRELPIEIASKLLMGLVLLLALFLTSSYGSGLATVMTI

PTYENAINTVEDFANSGLDWGATQDAWIMSIQNAEEQRYVKIVSKFHPISEEELFQFSKS

GKFGFSIERLPFEDYAIGDYIKEDVIDNFHLMKEDLYWEQCVIMLRKNSVLLPALDLFIL

KIFEAGLISHWQNEAVDLYMNPKVQRAVKFYRQGQEHTVVKLQWSHVKGPFALLLIGLCI

SFIIFILELTLKKKRNQF

>TcasIR41a.2

TLGCLTMTNLNVLLQILLKTYFLNTRCIFLFTDSTIDLQVETPIVYFKVSNTLNPSLIFQ

HHGCQNILIHHENASDIFVQFENLIRLNNERFNERKYIVTGHNSLKILLTKQLEYVSDLL

LVVPKQTGHYELITHVYRHQNRSKINEPVLLDVWYSQNHSFRQENDLFPNKLTNQNQRVL

KIGTLSYEPYSVIGKLTVNXSPYYLNLGKDDYSFDGTETSLVYEFVHKYNLTPSFTIMGD

DLWGDVYANWTGIGLFGSVLNDEIDIGYAAVYTWEEYYKFMDYTKTLIRSGVTCLVPAPQ

LAAGWVTPLRSFSLGMWIALVIVLLSNTIVLNLLFYRNQKYHXNQLFQILLFNAFSKRFF

IDSLTTAIKLYVQQPLTLTLKRGLLKYFIVTNMIMVLFISSSYSSGLSSVMTVPRYGKSI

QTVKDLASSHLNWTGTTDAWIFSLRQVEEANYENIKNRFVVKTQNDLVTASKQYNFGFSV

ERLPYGHYAVGPYIQRDVICNYRIMQEDLYWGQCTFLLRKNSVLLPLLDKLILRVFEAGL

EAYWENQVKCFGRKNMNLRDFLGCLPIHGHVCPKRHYVLYTTYXEHDTIKLTWEHVEGAF

AVLVLGYAASIFTFVIELILDKVRS

>TcasIR68a

MIKNLLPYKCVVLISDDIYGGTFTKSWYRRFGPFITFVVIRVDEYEDLLSPFEETQACLD

TAKNEGCQMYLILLSNALQVSRLLRFGDKYRVINTRAKFVLLYDNRLFDKPLFYLWKRII

NVIFIRRYSGQKSDTKKNMPWYEITTVPFPTQITSILIPRRLDIWTKSKFRKGIDLFRDK

TSDLRNQTLKVAAFSHIPGTTKSLQEKTARTVIGNFSGTEVEILQTVSAAMNFHCELYEP

VNVDVDLWGGKQSSGKYTGLVGEMVSTNADIALGDLYYTPYILDLMDLSIPYNTECLTFL

TPESLTDNSWKTLILPFKYFRPAMWAAVLVCLLICGAVFHALARFHETISQNKSQVLEIH

TKRKKIIILSICPEIEKLDSNLKYTKMREQYKPPRFEGQSIGLYQFSEPFNSVLYTYSML

LLVSLPKLPTGWSLRMLTGWYWLYCLLLVVAYRASMTAILARPTPRVTIDTLQELVNSRL

KCGGWGEINRQFFKSSLDPITKLIGENFELVNDSNEAVDRVAQGVFAFYENSYYLKEALV

KRQLRFQIARTTQNQSEREMRDIAREDRNLHIMTDCVIKMPISIGLQKNSPIKPRVDKYI

RRVLEAGLIKKWLQDVMASILNAEVQSTQEEMKAIMNMKKFFGAIVALFIGYFISVVVLI

VENVYFHFFVKRNPHYNKYTRSIHHVKKAE

>TcasIR100l

MPRKLFLWIFFLLVSCYGNLSETHLQFLKRYFVSANSVAISMLQTHHQEVKIRDLAEVIS

RKLNSIGTPVVVHENHKSGSLNIIMIVWSLKILRQFLDSLVVPEEKGTYYIIILEQDCAT

VHSDFAQILEQFWCEHNVLNVVVQNPCSGGTFYLFLPFEHRDNFWGSCKSWDFNEQMPNK

LRNLNQFPLKISLFLYNPTLIAKLPKGLKTNPRYHNLSASKGYGGLDGFLLRELVDYFNF

DPVIVENLEEYGRVLPNGTAFGSLGDVVNQRVHFSINSRFLMDYGTKEIEYTFPYISDEI

CMLVPKSLKVPTWKTLLKCFNTLSWVLIFVSCLCSTFAWYFVGPSKNLHKLIWQIYCFIV

GIPQKIEPSFSQFVFLLSCFFFNVTIFGIIQGSYFTEFATTSFYPDIDTLEELYESNLPV

ATHFWFLLDGDTSDLMTKLKTHKIEATGDCLEQTARQRNIATLGRKSESDLIIRTKYTSR

DGTPLVHIVEECHTSLYLCGIVPKGSHFLAPFNQIITRLFEGGFTTKWYRDVFDGIISEE

KPQLDETVSFNSLNMNDLQTAFHILTIGHLFSIMVLIGEVVIKGKHNKKLLT

>TcasIR100k

VTIIILIMMCLSLPKIQTCPIKINHLKEHFKQVKSARIMILQNEIIVTDWLIMELIKDNK

ITVTVQKAIRNFEPFNTSNLTRFEALEFNDTIPTLQTDSTCGHLIIVKNEERLYQYLKSD

PGFLILNPRHFYAIVAMELFKTNVLREFWSLQVSNILLLDCDTSYTVLPFNGTTIRINAY

TQRKLLRNFHNYFLQVSMQPKPPTAIVKFPKPLRENPIYKDLVPFKDYAGLDGCLLKVLT

QRLNMKYVIVGNGQKYGTVLKNGTTTGTLAWIASNKVQISTNGRFLMTYGTNKLEFTVPY

SSDQVCAVVPKALKIPKIIMLAKSLTPSSWFMIFLIYVICVLIYTLMGSTGSTWTLYAIF

HGFPVKIVPTSRQSFFLTSCMLFSIIIMTIIEGSFFKTFTTTTYYKDINTLEELDESELP

IAETFFSFTNDKSRIMTSLKRKKLVINRDDILEQVARKRNIAKLERKRDIKVRLKTEFLD

EEGESRLHVVEECFTTFYIGFIVPKNSIFLPTFNNVIRRIFESGLTQKWYGDVEFSIFLE

KIFKLENNIKHHSFSFDNIVSALCVLFIGLSLALLVFFWEVTKXKQITLIYVSLIYCIIS

RH

>TcasIR100j

LTLVQVVICLLEVSHYDNEKFVNVYQHFTLVRYLTLTFLNDGVHRIDLNNLVVDLMSRLN

FSMMIKEKRLGKNSTTFQESDPFQGHIMVVYDVKVLLAFLEESTEVVPKARGSFAILFTS

LKCPTHYETNHALKQLWTNHGTANLIAFCDNIYVYHPFSKNDSTWGATLDYSPATETPNL

FRNFNGYLLRVSLFKRPPTALKQVPSYISNNPIYRDLKPGDFAGLDGTLLRFLSNYLNFT

VVIDESHPTHGRVLKNGTITGSLSDVVSHRVDFSANDWFLIDYQTPEIEPTVPFSYDQVC

PVVSKALKVPQWKAFFFIFDLTSWVLIFFMWLCCVFVWHVLNPFRDLSTIIWEICSVLFG

NPVNVVPLSNQHMFLGSCMVLNIIIMGIIQGSVFTDFTTTTFHKDINTLEELDEAGLKIA

SSAWYLDFDTTDLIKRLKTKQIRNYIGSYKDTAFKRGMAVLGRKQDVEHMVKVEFVAEDG

SPLLHVTSECLQTFLLVSLFPKGSPFLPTFNNVITRLFEAGLTVKWYQDVTSTGTMLQQM

KNFANRRPTGLFSLNDAKLAFYALFVGYIASFVTFLTEILTKNHHNNVHNHVDVLKAQHH

GQVQVDQ

>TcasIR100n

DTFWIVYQTHFLLTDYLTLHILETEDHKFELRQFTQNILKRVNKYGYFLSVRITKSSLNK

RNKSYHFPSTAYAPSQNLAKLSDDQEFYKAKRLSTDSKHGFALIVWDLTTLHLFLDQDYR

TIVPEGRGTYAIQVVSKQCDVKNEIAFTLQRLWTEYQVINVVAQTPCSCDKTHIFIYHPF

VKREGFWGLATSHTLDQIKGDSRLISNTLSDFNGFPLRISIFPRTPTAMQTLPKLLHYNP

IYRNLTWSKGFAGLDGLVLATLAEYFNFEVVLVGSLLEDDFGKVLPNGTTVGSLADITER

RAVYNANERLVAYFNLDQIDFTVPYTREDICLVVPKAAKIPKWKILFQSLDPQSWCFTLF

AYVSCFMFWYNIGPSRSLPKVSWQMFSFFLGIPTKSFARKLDQVLFLIPCMIFSVVMLGV

VQGSFFTKLTLFSFYQDVNTLEEMADLELPIGAFIWNLIRDDSDVIRRLKSKSVKPPDNI

FDMIAAHRNIATIETRARAQLLIGSKYVDDDGFPLLHIVNECLTTFLNANIVPKGSALLT

VFNAVLGKLFESGLTRKWNNDVVDSLIAEKMISVNRKRVRTKSFSLYDAQGAFFVILVGY

ACSVFVFLCEIVLKXDKICYLALIINKT

>TcasIR100e

DDFWRVTKNHFLLVNSLTIQVLQTEEHQYDLNQYTVTLLKRLNSLNLLVALRMQEKFLSG

RNFPKHSVTNHTFSTTKPKFDPIGGEELTQLKRLSSDSSKGYFIVIWDVESLHNFLDEDF

QVVVPEARAXYMIHFAFTYSTEACKIVKLQVSSVLTRLWIDNNVFNIIAQTSCLCDLEVY

VHRPFVKRGGFWGLTNSYQMSEIVENPRIIANPLINFNQFPLKIGIFPRPPTVIETLPKL

LTDSPIYKNLSFSKGFAGVDGLVLGTLAECLNFDTTVITSKPNSYGYIYKNGTATGAIAD

VIDRRMVFSANSRFLLIYNTDQLEFTVPYTAEKMCLAVPKALKVYKWSSMFRCFNKLTWV

SIICSFGICTIFWYLLKWQKLVTALATIAQFLLGVPANVRPNVPQMLFLNSCMGFNIVIM

GIIQGFLFQSFTTTSFYPDINTIEEMVDSELPLRSSIFYFLRIDNSSLIHKLKSRTMAAP

PNVYDLVAFHRNIATTDIKSHVDFMVRSRYLDEDGWPLIHTVDECFETFLIANIVPKGSA

FLTVFNNVITKLLEGGLTQKWYEDVINSLILENWINLNRNKSKTHAFSLYDLQVAFYVII

MGCAVAILVFVAEIVHKRRNXNNCCNNHHKNIIFAA

>TcasIR100f

DDFWVIFSTHFLLATSLTFITVQTNSKQYDLRLLAQAIIQSMDKDQVMTTRHVILHNYAE

NINFNVVFKTGTKKNARDFVTDLLAKTKKLASDSREGFVIITWNVNVLQKFLAQHISEIN

PRTRATYLFILISSSDSLRKIKHCLHFLWHKYDILNIVVHVLGCGTTTTLIYRPFCKTKN

SWGEITAHQIEEIVQQPLLLTNSLQDLNQYPLQVSLFARDPTALTQLPKLLQNNPIYKNL

ASFYGLDGSMLSTMAKILNFEVVIVENHDRLPFGRVWPNGTASGTLGDVVNRRVALSSNS

RILADYNTQEIEFTVAYNGDSICVAVPKSLKVPKWRVLFECFDAASWLLTLSVFIVCLCF

WYCVALKNFARILWDVYSFLMGIPTRIVPSRQYFFLSSCMVFNVIILQLLQGWLFTAFTK

TVFYPDLDTLEVLEKTNLPVATNMWFLFKDNSEVIQKLSSRGIGKTPNSLDLVAYSRNIC

VLDKRQDLELYSQAKFVGPDGLSLLHIVNQCLTSVLLVNIVPKGSPFLPVFNDIMSRLFE

SGFTKKWYSDVVTSRVTEKMVSLGRKERNFSFKIKDLQAAFYVMMAGCVFSLFVFVGELV

THXVFVMNKSSQSKSHRFLLNCNYGV

>TcasIR100g

TLFKIAEVTFFMVTMHEEFLSLLFGNYYHTNLYQTVKIQEKFARTNNKTGAWYENVALDQ

KLDPPIDQNWQRVKLRTSDSFEGFIIIVWDPQTLDQFLNQNFSLVVPRARATYFLLFVFS

IYENCKLVNHILKRFWSEFSVLNIIAQTPYCCNKVYIHRPFVKTTNSWGVTQSYTLTEVT

QNLALITNPLLDLNQFPLRIALFEKNPTAIRKLPKALQNNPIYRNLSRSKGFAGSDGFLL

SAMVEYLNFDPLIDETLEPMNFGHVLPNGTVCGVLAEVVHKRTDYAGNCRLMTYFGTDGY

EFTAPYSSEKIAMVVPKAGKVPRWRSLFNCFNALSWSLIFSIAIVSTVFWCFLRRSQHLK

RASWEMFAHFVGIPCRVVPSRGQFMFLTACMMFNIIILGIIQGSFFTDFTTTSYYPDLNT

LEQVLDSNLPIMAFAWRLLRTNSSPILQKLEQRSIPYEDNVYELVALYRNVAALDRRLDL

ELEIKTKYSGRDGVSPLHIVDESLVTFLTTSLVPKGSPFLVVFNHVIRSMFEAGLTAKWY

DDVVTSLIIEHKHKTPSFGVKYRPFTLQDVQAAFYVIAFGYSCSVFVFWCEIIVKFSGKI

KHFHYYFVLI

>DponIR21a.1

RKVDYVPDVESGLRNVTKAFFWPYALLGSREELQFIVKTNFSLGSKKSMLHISQQCFVPF

KVGIALPHHLVYSEILAGGIQMILQSGLNIKMKNDIEWEMLRSSTGKLLAANSRSGTLTI

LSRDDRALTLDDTQGMFLLLAIGFLAGGGVLISEIFGGCFNLCKKIDNSRATSSNSSIPS

NPRFHERQTIRERNRSISLASFQQRHNSIQSEIAFEKAQAEEHHQGGLVECQIHGTTPDQ

NSQGVVLEETNADIDYNEQISKLFEQALGEETCGSRPLRA

>DponIR25a

MKNNNIVAGGFFSIFLLNVADICGQTTQNINVIFANEEGNFVADKAVTVALNYIKKTSKL

GLSVDLRRVVGNKTDSQNVLDSLCAAYQQMLDDNNPPHLVLDATRAGLASETVKSFTAAL

GIPTVSASYGQQGDLRQWRNLQPNEEEYLVQISPPGDIIPEMVRTLVLNQNITNAAILFD

DSFVMDHKYKALLQNVATRHLIDEINEDVNKIPDHLESLVKLDLKNFFVLGSLQTIKNVL

EAAEKKSLFNRMFAWHVLTKDPDDLKASIKNATIIFAKPIVNNLYQDRLRNIQTTYQLSS

VTPEIEAAFYFDVALKGFLAVKEMLLDGSWKKNNVTNYVTCDDYEPKYSPKRFNLNLRSY

LQKESSEPPTYGPFAIESNGMSFMEFSMALSAVYVRSGASDKSLPLGTWHGGFNNNMTLL

TPKDMKNYTADVVYKVVTVVQKPFIYRDDTAPKGFKGYCIDLIDEIAKILHFDYEIDAVA

DGMFGNMDENGKWNGIIKDLIEKRADIGLGSLSVMAERENVIDFTVPYYDLVGITILMKM

PETPTSLFKFLTVLENEVWLCILAAYFFTSFLMWVFDRWSPYSYQNNREKYKDDEEKREF

NLKECLWFCMTSLTPQGGGEAPKNLSGRLVAATWWLFGFIIIASYTANLAAFLTVSRLDT

PIESLDDLSKQYKIQYAPVNGSSTMTYFQRMADIEAQFYEIWKDMSLNDSLSDVERAKLA

VWDYPVSDKYTKMWQAMKEAGLPPDLDTAIERVKKSKSSSEGFAYLGDATDIKYLEITNC

DMAIVGEEFSRKPYAIAVQQGSPLKDQFNTAILQLLNRRELERLKERWWNKNPEKKQCEK

ADDQADGISIQNIGGVFIVIFVGIGLACITLAFEYWWYKYRKNTRVTNVAEAPNSRHHKV

GGVQKGFPRQFEGESDMKITKLYPKTKF

>DponIR41a

MGSEMKILETFSQYVNASISPVINQADYWGEIWNNWSGSGLMGNLVEDKADIGAAALYTW

EFAYEYLDLSKPTVRTGITCLVPAPKLSAGWLTPFRVYSLEAWMALIGTLALSFLALYAL

NKLQISVKPQLKSKHHINQLKGKLLSKTLMSVSKPFVMQSITNKEMAQGNLAKYLMGLVF

LSTLVLSTTFDSGLATIMTVPRYDNPINTIEELAESGLPWGGTQDAWILSINNSLEPNLM

KLVARFVAHSEANLRKYSLGDQFAFGVERLPNDNYAIG

>DponIR56e.1

MAYNYNQGPYAGQQQPGYGPQPGVYPPPPQGYPQGGYPQPGGYPSAPQPGFQPPPYGDPY

GGANQGYGGYDAEDPEVKGFDFSDQSIRRGFIRKVYSILMVQLAITMGFIALLCYEPKTK

AFVHNTPSLFIVALVVMIVAMITLACCGEVRRKAPINYVMLFIFTIAEGFLLGVSASTYK

QDAVLMAVGITAAVCLALTLFAFQTKYDFTMMGGVLLVAVIILLVFGIVAMFVHNKIVQL

VYASLGALIFSIYLVYDTQLMMGGKHKYSISPEEYVFAALNLYLDIVNIFMYILAIIGHA

RD

>DponIR64

MDNGIDVQNHRERTSDTSWSLSTICTFGVFCQQGIISVPRCLGGRTSAIVALWCGLVIYQ

FYSASLVSFLLNVPVNVLSTVQDILDNGFDVGYERVLYAMSLLKAATNPAAQEVYRRVSN

KNQTGYLRREQGLELVKNGRYAFHVELVTGYPFIEQHFDESMICELKEISLFPAMYMYSG

YQKWSPFREFLDACLHRLEENGVVSRELYFWHPRKPQCMRSRSTI

>DponIR56e.2

RADLTSITSARNMWKKVVLLAAMLTITSQVDFSSIEFIEAVGRNRRATVFGCSRKDLVET

SKLLSEKNFESNWISEWSSSDGRIFHKSRHIFLVNLNCPETMRFLKQAQQWKLFASPFRW

IAFHQATKTPLAIEKTFRNLSILVDSDFNLVTHQNNREISYRKIYKKRIDESTFEVEDFG

LWRQNSYFDRQTSSLNDRKNLGTVLKACIVITHNDSLNHLEDKRDKHIDSIAKVNYVLVL

TMASIYNISINFSVTNTWGYKDNNSQ

>DponIR75p.1

MSWTILCFLKMFSVCLSSSPAHADTDISFLFDFLQQTNRPNYAVLENVCWSKEETQKLHR

NLTMQNFKCKSVDGNSTIMTERYYEHSYIILVHVDCDFDTIWKQAAKGSLTFYPHIWILL

GDFEKIKKKNVHIPMNSLLLSLVRTANYTKLETAYKIKKTVDWYIERTVGNWSPKVAMFD

LEKVNIFKDRGNFMKVPLRVTYIITDNSTVNHFIDYRNKHVDNLTKINYVLYILIFDILN

ATQIRLFSRGWGFESRENNGSFASGMFQDLANDRSDIAGTLAFTPSSRLKYFRYIYPPAK

DMDICLVFRAPSLAYYTNVFGLPFNNWVWVALGLQLLCGCVLIFIIFKWEWKVAQGRKEN

GPSFLDTAMMQIAIACQQDFFHEPKSISGKMATLSILIFFTFIYTAFSAKIVLFLQLSTN

KINDVGSVYAAGFDFAVEDQPFNQYYFKGPSDRAEEQLRKQIYEKKIRRSHGDQFVSAEK

GIQLVRDTFCAFHVERTVAHYLVDKTFSNNQKCSLRFVRSIFKSDMPYLSIPWNSSYIKY

FIISFRRLAETGLQDRECKRCYAKKPSCEGKANTFVSVGLIEAYFPLLIFGVGISLCISI

LILEKLVHKYIKLH

>DponIR75p.2

MANFRQHHNFKGQTRLFPHQQLRSGYQRINRQNLEDSSPVPAKELLELFGKLHSRLVRRS

GLYLLQPANFDKNRTNFHKMPLKVTYVVSDNHSLDHGIYDNRNKHIDKATKINYIMYEYF

VDIWNMSHEVVVTNEWGQEFHPSKLYYKGLLGDLCHEKADAAGTVMFTPTERLKYFKFLV

STTKEMSLHFIFRAPPLPYSHNLFALPFDTNIWISCAIVLSLCGLVIWIIMSWEAKVASF

AANRQMHNENAASFLDIVMMQIGVVCQMDYFHEPRSTAGKIATFSLLLGFSYLYNAFCAR

IVVLLQATANNLHDYKALYEAKMDMGVEATSYNIYYFSHPNSRTNEDYRKLIYQKKIAPN

NKFLPATDGMRLVQNSYFAFHVELTTASDLILATFNNQEKCAVRKVNSIFKEDKPYLASP

INSTVTEYLMIGFHRLFETGVHSRESRRRFNKLPPCRGRNSAFISVGLIECYFAIEIFLI

GFTLCLAFFLLELASGVYRKNQST

>DponIR75q

MNTLYFGFFMWFALICGGHCGVHADDVLLLIEDLLDFYRFSKKVYTHVCWDKELQLHLPR

FALHAKYHEFQSTKSQVVILMDMSCKGADFFLENMQKRNLLTPRVILLLINPAALTPHYF

PVNSNALFLKPSGEGFAISKIYSGVSNVTIGTWTKTHRYIERPIAKKKLRATNLKVCYLV

GDKHEGLESSEYQPVAEATTKLNRVLLEDGIRMINSTKTDAFQIGSGEPELVNDLIAGKC

DIGGTPLALTAEKIGRLDVLAKTLREDKTFVFRAAPHSYISNVFTLPFDSYVWCSCFGLM

GVIFLIVHLVVCWEWKDPVFKLNLQPNISLRPNPVDILLMEVGSAAQQGFEAEPRSNSGR

IVFMSTLISFMFLYTSFSASIVALLQSTTDSLNTFDNLFQSRINVGFKQNISLDFFNDLK

HQPNRADYHGKLKEPQFFTLEEGVKRLQNDFFAFYAETSEVYRYINRWFQESEKCSLREI

PFKNTHINHWLFMGKNSQYRDALRIGMNSIQERGIRSREYKRLFPLKPFCDSIGGNFESV

GLVDSYGAFLIMVYGVALSSLLFLLEHMSLQYNLSTKAGRIRNRIFNSIHRGDE

>DponIR75s

LVVLYFLRKWEFAKIKQQELLHKTAYPDVVFITIGAVCQQGAATLPHSVPGRIATLWLFV

SLMFIYVSYSANIVALLQTSSNSIRTLEDLLKSRIPIGVDDTIFNHFFFTTTQEPTRRAL

YEKKVAPPGKRPNFLSIEEGVRRMRQGLFAFHMETGAGYKIVGETFTEDEKCGLQEIQFL

QVVDPWLAIQKNSSYQELLKIGFHLLRETGIQQRENNLIYTKKPSCSSKSSTFFSVGLVD

CYPAVLIFAEGLAAGLALFLLELYIHKKFSNQ

>DponIR75x

PAAKSIRNVIYSRRRAFLDKVRLHKAILEENLNMRVVFAKARVDAFENINTKRYEKFTYF

LDMQCPDSADILDKASNVNRFGKFEFIYSWYLLSKVNQELEDIFHIFTRFKTRMDMDVKV

LAVDDFGRFEVSEIFNPGINVGLTTRRIGKLQNGSVIIDKNWSYYESRMNMTGVLIRSAN

VIRYPFTTSFDEYMTDPKLMKYDIYSKFHYQLFQGLVHIHGFEYNTSLPFSWFGNTSSGE

DGGLAKMLWDDTIDISSAGCILRLLDSDRIDFYDYIMPYYKFRSCFFPQSRSRKAELLRS

IKAV

>DponIR76b

MGLMEVVLTTLATLCFNSTCVDQDLINASKQRLAHLKEELKHETLTVTTLKNGPLSGYEI

VNNTVIGTGVAFEILNIVQREYGFKYNVIVPDHDSFEPVNGGEGGVRNMLLNETIDVAVA

FLPQQYTDVVSYSRSLDTAQWVVLMKRPKESASGSGLLAPFTATVWSLIIISLLGVGPIL

WLTILLRARMCKEDHDIVFSLPSCMWFVYGALLKQGSTLNPRTDSSRILFSTWWIFITIL

TAFYTANLTAFLTLSKFTLPISEPKDISRKHNKWITNRGNGIVEQLYLSKKYANGDGNSL

FEEIGMPQWEPDVDEDTMLSTYVIKQNMMYIREKTVLESIMYEDYKVKTKADVEESKRCT

YVITKFAVCVFPRAFAFRPGFKYKELFDFTIQHLSESGITDFQQRKSLPDTTICPLDLGS

KERRLRNSDLAMTYMIVGGGLIISTIIFAVELIIYYAKMHCFNKKSHVNNNNTLVTQSNN

GLFVKNHQHQGNFRASKQFVSPPPSYHTLFHPPNLTNGEYKNKTINGRQYWVFNDKQGMT

SLIPQRTPSALLFQFTN

>DponIR8a

MHVLGLFGAVACVLSVRGQQFKIVTLHQPDQAAEVKYFENAFLKVNKDEEIAFLDVLLNE

DESGHYKQICDALSTGFSLILDFAWSGTEVAQDLTSNMSLPYLHVDVSVAPFLVLLDSYL

DSRNSTDVVVVFDKEEYIDQSLYYWLDSVRLRLVMADALNRSTASKIESIRPIPHSFAIV

ASAKNMNKLVSQALNEDLMSLSDRWNLVFTDFETGIFDKSLFQNQTPSLMYLKPELCLDL

SIQSRCPSNFVLKEQFLYWLAWGLSRLAKMAAEESLEFPEKEFQCGKTTFSEDTKERLGD

MLDSIVADNSNVLSLTGRSVKVAVRGNVEKMINGSFQTIAQYTNGKLTPEPGKQIDPIRA

FYRIGITHAIPWSFKAQNLQTGEFYWTGYCADFAQKISEVMNFDYVFVEPATGTFGEKVN

GTWDGIVGDLAVGETDIAITAVIMTADKEEVIDFVAPYYEQTGITIVMRKPVRKTSLFKF

MTVLKLEVWLSIVGALIVTGFMIWFLDKYSPYSARNNRKAYPYPCREFTLKESFWFALTS

FTPQGGGEAPKALSGRTLVAAYWLFVVLMLATFTANLAAFLTVERMQAPVQSLEQLARQS

RINYTVVQDSETHMYFINMKFAEDTLYRMWKELTLNASTDDTRYRVWDYPIREQYGHILL

AINDSNPVANASEGFRITNEHLDADFAFIHDSSEIKYEISKNCNLTEVGEVFAEKPYAVA

VQQGSHLQDDLSKVILDLQKDRFFEQLQAKYWNDSAKGDCPSTDDNEGITLESLGGVFIA

TLFGLALAMITLAGEVLYYRRKGKKIKPKKRKAKSKNLPLNTAGLKLSKLDGQSVDMFNV

NKTVTIGTTFKPVNLKENLTKEMETVHISHISLYPKARNRIPRVE

>DponIR93a.1

TKWTIEAGETWGREYQMLDEATNAELLAVGTWRPSDGPNMIDALFPHVAHGFRRKLLPLV

TFHNPPWQILKTNSTGDVVEYGGIVFNIIKELSKNLNFTFNVATVKPQSLLNASTLQSPK

GDTDSSANFNGNSYITTYRVPHSILEMVHNKSAALGACAFTVTEENQRVINFTDPISIQA

YTFLAARPRELSRALLFISPFRGDTWLCLSATIISMGPVLFYIHKLSPVYEYKGVRCKGG

LATIQNCIWYMYGALLQQGGMHLPYADSARIIVGSWWLVVLVIGTTYCGNLVAYLTFPKI

EVPMTTIDDVLAHKEMVSWSYAKNTLFEARLHNSVDKSFNIIFKDAKNIWDRKAMMGEIK

SGKHVYIDWKIKLQYMIKEHFIDSGECSFALGVEEFCEEQIALIVAPDTPYLHKINEEIK

KLHQVGLIQKWLSDYLPKKDKCWKKKRTIEVNNHTVNLDDMQGSFFVLFIGFLIAVIVIS

LEMLWSRKVTNNRKRKVVHQFVT

>DponIR93a.2

MWVRLVICLGVFCKVTNSDIFPSLLTTNASIAIVIDRNYVVEEYEPIKSKIEDYLVYAKR

EILKHGGVNTHLFAWSAINLKRDLTFLLSITSCTETWKLFESADTESLLHIAISEQDCPR

LPQHSAITIPIIDRGQDTPQLLLDLRTVGIYKWKQVVIIYDNTITNDLLTRVIKSMTKQV

NRIDASGVSLVQLAKKVSTTRDVIIANLRTELSKIDPRIMGNNFLVIVSYELAATIMKCA

KELNMVNTQTQWLYVISDTNSSTKSMNRFKTFLNEGDNIAFIYNTTDVKNVCLGGTICHT

EESITGLMKALDSAIMEEFQMASQISEEEWEAIRPTKNERRKYLLRKNSEYLSDYGTCDN

CT

>ItypIR76b

QSTGVSYTKLFDFTIQHLRQAGIIQFKHRELLPDAKICPLDLGSKERRLRNSDLAMTYQI

VGGGLIISS

>ItypIR25a

RGNREKYKDDEEKREFNLKECLWFCMTSLTPQGGGEAPKNLSGRLVAATWWLFGFIIIAS

YTANLAAFLTVSRLDTPIESLDDLSKQYKIQYAPVNGSSTMTYFQRMADIEARFYEIWKD

MSLNDSLTDVERAKLAVWDYPVSDKYTKMWQAMKEATLPPDLETAVERVRKSKSSSEGFA

YLGDATDIKYIHMTSCDFVVVGEEVSRKPYAIAVQQGSPLKDQFNSAILQLLNRRELERL

KEQWWNRNEESKQCETSDDQQDGISIQNIGGVFIVIFVGIGLACVTLAFEYWWYKYRKNS

NITNVIVSDPKHRRVAGFPKDVGGKANEGELALRPGKLYVKPKY

>ItypIR64a

LVKLGLNLVKQGHYAFHVELVTGYPFIRKHYSESMVCELKSVSLFPSMFMHANYQKWSPF

KDLLDVCLHRLGENGVINRELIFWHPKKPECIRSSSTININTGLESFYPALVVLLLGILA

SLNILLLEILWFKYQKRQILPYTE

>ItypIR68a

VRGDTTQFKDEGNQSVSNKIRANIMLSNATELFSGLEIEILDTLSKVMNFHCELYEPDRA

DTELWGRKQYGGVFTGLLGELFTSKADMALGDLYYIPFILDVMDLSIPYNTECLTFLTPE

SLTDISWKTLVLPFS

>ItypIR75p

QSRFIAEREMYEENAPDFLDIVLMQIGVVCQISYSFKPRSTAAKIATLSLLVGFVYIYNA

FCARIVILLQSTANNLNNYKDLYYSKIDMGVEEAPYNKYYFSNPNNRANEAWRKLIYETK

IMSKNKHPVFYSTAEGLKLVKQSYFALHVEYTTATDVILATFTNEEMCAVRVIESIYKED

VPYISCPVNSTFTEYLLIGFHRLFETGLHSREARRKFSKLPKCIGRNSIFVSVGIIGCYF

AVEVFLVGVILSLVIFAVELV

>ItypIR75s

SKFVFRQPKLSYVTNVYTLPFVSKVWYSTVILTVLMALALYGLMKWEHAKNHFLLEKSLA

GRGRQNQTELRDSIKDVAFVTIGAV

>ItypIR75s.2

STGGAKCLSVTLQCAWTICHVDFIHHINVYVRVLLGQYSGIIADIVKQYKNSGRPFKLQN

SFRSRRHGFQSLFLYYYYRTNQEGNLPKKGGATGQKPNFMPIEDGIKRMRQGLFAFHMET

GSGYKLVGETFEEAEKCGLQEIQFLQVVDPWLAIQKNSSYEEHLKIGLRLLHETGIQQRE

NNLIYTKKTDVL

>DmelCG11155

MVRKKREIVIKENIQGRSYLKKICCSYIILSILVISNALPPVIRVGAIFTEDERESSIES

AFKYAIYRINKEKTLLPNTQLVYDIEYVPRDDSFRTTKKVCSQLEAGVQAIFGPTDALLA

SHVQSICEAYDIPHIEGRIDLEYNSKEFSINLYPSHTLLTLAYRDIMVYLNWTKVAIIYE

EDYGLFNLMHSSTETKAEMYIRQASPDSYRQVLRAIRQKEIYKIIVDTNPSHIKSFFRSI

LQLQMNDHRYHYMFTTFDLETYDLEDFRYNSVNITAFRLVDVDSKRYLEVINQMQKLQHN

GLDTINGSPYIQTESALMFDSVYAFANGLHFLNLDNHQNFYIKNLSCTSDQTWNDGISLY

NQINAAITDGLTGTVQFVEGRRNIFKLDILKLKQEKIQKVGYWHPDDGVNISDPTAFYDS

NIANITLVVMTREERPYVMVKEDKNLTGNLRFEGFCIDLLKAIATQVGFQYKIELVPDNM

YGVYIPETNSWNGIVQELMERRADLAVASMTINYARESVIDFTKPFMNLGIGILFKVPTS

QPTRLFSFMNPLAIEIWLYVLAAYILVSFALFVMARFSPYEWKNPHPCYKETDIVENQFS

ISNSFWFITGTFLRQGSGLNPKATSTRIVGGCWFFFCLIIISSYTANLAAFLTVERMISP

IESASDLAEQTEISYGTLEGGSTMTFFRDSKIGIYQKMWRYMENRKTAVFVKTYEDGIKR

VMEGSYAFLMESTMLDYAVQRDCNLTQIGGLLDSKGYGIATPKGSPWRDKISLAILELQE

KGIIQILYDKWWKNTGDVCNRDDKSKESKANALGVENIGGVFVVLLCGLALAVVVAIFEF

CWNSRKNLNTENQSLCSEMAEELRFAMHCHGSKSRHRPRKRSCLNCSSVPTYVPSNVSTS

NVGVYYNYFN

>DmelCG3822

MRSSGVLVLPLLLLQLILNCRKAQSLPDIIKIGGLFHPADDHQELAFRQAVDRINADRSI

LPRSKLVAQIERISPFDSFHAGKRVCGLLNIGVAAIFGPQSSHTASHVQSICDNMEIPHL

ENRWDYRLRRESCLVNLYPHPNTLSKAYVDIVRHWGWKTFTIIYENNDGIVRLQELLKAH

GMTPFPITVRQLSDSGDYRPLLKQIKNSAEAHIVLDCSTERIHEVLKQAQQIGMMSDYHS

YLVTSLDLHTVNLDEFRYGGTNITGFRLINEKIVSDVVRQWSIDEKGLLRSANLTTVRSE

TALMYDAVHLFAKALHDLDTSQQIDIHPISCDGQSTWQHGFSLINYMKIVEMKGLTNVIK

FDHQGFRTDFMLDIVELTPAGIRKIGTWNSTLPDGINFTRTFSQKQQEIEANLKNKTLVV

TTILSNPYCMRKESAIPLSGNDQFEGYAVDLIHEISKSLGFNYKIQLVPDGSYGSLNKLT

GEWNGMIRELLEQRADLAIADLTITFEREQAVDFTTPFMNLGVSILYRKPIKQPPNLFSF

LSPLSLDVWIYMATAYLGVSVLLFILAKFTPYEWPAYTDAHGEKVESQFTLLNCMWFAIG

SLMQQGCDFLPKALSTRMVAGIWWFFTLIMISSYTANLAAFLTVERMDSPIESAEDLAKQ

TRIKYGALKGGSTAAFFRDSKISTYQRMWSFMESARPSVFTASNGEGVERVAKGKGSYAF

LMESTSIEYVTERNCELTQVGGMLDTKSYGIATPPNSPYRTAINSVILKLQEEGKLHILK

TKWWKEKRGGGKCRVETSKSSSAANELGLANVGGVFVVLMGGMGVACVIAVCEFVWKSRK

VAVEERLSAILNE

>DmelCG5621

MISTEASFPLGFILTSLLLAFPGCRGERTNVGLVYENTDPDLEKIFHLAISKANEENEDL

QLHGVSVSIEPGNSFETSKKLCKMLRQNLVAVFGPTSNLAARHAMSICDAKELPFLDTRW

DFGAQLPTINLHPHPATLGVALRDMVVALGWESFTIIYESGEYLPTVRELLQMYGTAGPT

VTVRRYELDLNGNYRNVLRRIRNADDFSFVVVGSMATLPEFFKQAQQVGLVTSDYRYIIG

NLDWHTMDLEPYQHAGTNITGLRLVSPDSEQVQEVAKALYESEEPFQNVSCPLTNSMALV

YDGVQLLAETYKHVNFRPVALSCNDDSAWDKGYTLVNYMKSLTLNGLTGPIRFDYEGLRT

DFKLEVIELAVSGMQKIGQWSGEDGFQENRPAPAHSLEPDMRSLVNKSFVVITAISEPYG

MLKETSEKLEGNDQFEGFGIELIDELSKKLGFSYTWRLQEDNKYGGIDPKTGEWNGMLRE

IIDSRADMGITDLTMTSERESGVDFTIPFMSLGIGILFRKPMKEPPKLFSFMSPFSGEVW

LWLGLAYMGVSISMFVLGRLSPAEWDNPYPCIEEPTELENQFSFANCLWFSIGALLQQGS

ELAPKAYSTRAVAASWWFFTLILVSSYTANLAAFLTVESLVTPINDADDLSKNKGGVNYG

AKIGGATFNFFKESNYPTYQRMYEFMRDNPQYMTNTNQEGVDRVENSNYAFLMESTTIEY

ITERRCTLTQVGALLDEKGYGIAMRKNWPYRDTLSQAVLEMQEQGLLTKMKTKWWQEKRG

GGACSDADEDSGAVALEISNLGGVFLVMGVGSFFGIFVSLLEMVLGVKERSDENQEAPDS

DASSLGFANLGGVYLVMFVGSCFGSIYGLVNCVVSVYLRARENKVSFKTELLDEIRFILQ

CSGNTKAVKYPKNSSRSNASSKSKGSSMSVDSLPEDTSEADASGKHNHGKK

>DmelCG9935

MLIASGFLLFQFLSYGLGVPPLVRIGAIFSNQPGMYNSELAFRYAIHRLNMDKSLLPETT

VDYYVEYVNRFDSFETVQKVCKLIRVGVQAVFSPTDSVLATHINSICDALDIPNIGRSAH

DFSINVYPSKQLVNYAFNDVIQYLNWTRFGILHEKENGIINLHQLSRSFHGEVHMRQVSR

DSYVSALNEFKGKEIHNIIIDTNSNGISILLKNILQQQMNEYKYHYLFTSFDLETYDLED

FKYNFVNITSFRLVDTADVGVKQILKDIGLYSHHIFKKPYLNLHIKKSTILESEPALMFD

SVYVFAIGLQTLEQSHSLTLLNISCEEENSWDGGLSLINYLNAVEWKGLTGPIQFKDGQR

VQFKLDLIKLKQHSIVKVGEWTPHGHLNITEPSMFFDAGSMNVTLVVITILETPYVMMHY

GKNFTGNERFYGFCVDILETISREVGFDYILDLVPDRKYGAKDPETGEWNGMVAQLMKYK

ADLAVGSMTITYARESVIDFTKPFMNLGISILFKVPTSEPTRLFSFMNPLAIEIWIYVLI

AYFLVSLCIYIVGKLSPIEWKCINACDLENISIGNQFSLTDSFWFTIGTFMQQSPDIYPR

AMSTRIISSTWGFFSLIIVASYTANLAAFLTTERMINPIENAEDLASQTEISYGTLDSGS

TMTFFRDSVIETYKKIWRSMDNKKPSAFTTTYEDGIKRVNQGNYAFLMESTMLDYIVQRD

CNLTQIGGLLDTKGYGIATPKGSPWRDKISLAILELQERGDIQMLYDKWWKNTDETCTRK

NTSKQSKANSLGLESIGGVFVVLIAGIIVAAVVAFFEFWYNFRYNYEATPSQSVVNNKYN

QDGILESERNYTPPDRSFWIEIAEELRYASWCMNKQKRPALTRTCSKCTIPKGQRINKL

>DmelClumsy

MYSLFLTHFLLIALPVLADIDRSQFMVGSIFTSDKDESEIAFRTAVDRANILERNVELVP

IVVYANTDDSFIMEKMVCNLISQGVIAIFGPSTGSSSDIIASICDTLDIPHIVYDWIPNE

SIPDREHSTMTLNVHPDNLLLSQGLAEIVQSFAWRSFTVVYETDKELQQLQDILQVGEPI

SNPTTVKQLGPGDDHRPFLKEIKLSTDNCLILHCAPDNLLKILQQANELKMLGEYQSVFI

PLLDTHSIDFGELSGVEANITTVRLMDPSDFHVKNVVHDWEEREKREGRYFKVDPNRVKS

QMILLNDAVWLFSKGLTELGIFEELTAPDLECRRKKPWPFGKRIIEFIKARSEETSTGRI

DFNENGQRSFFTLRFMELNSDGFLDLATWDPVNGLDVLNDDEESEKRVGQKLSNKTFIVS

SRLGAPFLTLREPQEGEILTGNSRYEGYSIDLINEIAKMLNFKFEFRMSPDGKYGALNKV

TQTWDGIVRQLIDGNADLGICDLTMTSSRRQAVDFTPPFMTLGISILFSKPPTPPTDLFS

FLSPFSLDVWIYMGSAYLFISLLLFALARMAPDDWENPHPCKEPEEVENIWSIMNTTWLS

IGSLMGQGCDILPKAASTRLVTGMWWFFALMMLNSYTANLAAFLTNSRQANSINSAEDLA

AQSKIKYGAMAGGSTMGFFRDSNFSTYQKMWTAMESASPSVFTKTNDEGVERVQKGKNLY

AFLMESTTLEYNVERKCDLVQIGGWLDYKSYGIAMPFNSPYRKQISAAVLKLGELGQLAE

LKRKWWKEMHGGGNCEKSDEDGGDTPELGLENVGGVFLVLGLGLLSAMVLGCTEFLWNVK

SVAIEEKISLKEAFKSEALFAARIWITTKPVHTSSESGSSNSSSSSSSRSKHSFKSQGLS

MKSLKSSGYQDVEASVHSKLKKIGSMFSLKSQKTVTPPPEIGWKLDKSTQIDVVPTSDVD

QELIPEVEPHLPHRHHHHHHHRHHHHHHQPDQEHDRNPSPPE

>DmelGlu-R1

MHSRLKFLAYLHFICASSIFWPEFSSAQQQQQTVSLTEKIPLGAIFEQGTDDVQSAFKYA

MLNHNLNVSSRRFELQAYVDVINTADAFKLSRLICNQFSRGVYSMLGAVSPDSFDTLHSY

SNTFQMPFVTPWFPEKVLAPSSGLLDFAISMRPDYHQAIIDTIQYYGWQSIIYLYDSHDG

LLRLQQIYQELKPGNETFRVQMVKRIANVTMAIEFLHTLEDLGRFSKKRIVLDCPAEMAK

EIIVQHVRDIKLGRRTYHYLLSGLVMDNHWPSDVVEFGAINITGFRIVDSNRRAVRDFHD

SRKRLEPSGQSQSQNAGGPNSLPAISAQAALMYDAVFVLVEAFNRILRKKPDQFRSNHLQ

RRSHGGSSSSSATGTNESSALLDCNTSKGWVTPWEQGEKISRVLRKVEIDGLSGEIRFDE

DGRRINYTLHVVEMSVNSTLQQVAEWRDDAGLLPLHSHNYASSSRSASASTGDYDRNHTY

IVSSLLEEPYLSLKQYTYGESLVGNDRFEGYCKDLADMLAAQLGIKYEIRLVQDGNYGAE

NQYAPGGWDGMVGELIRKEADIAISAMTITAERERVIDFSKPFMTLGISIMIKKPVKQTP

GVFSFLNPLSQEIWISVILSYVGVSFVLYFVTRFPPYEWRIVRRPQADSTAQQPPGIIGG

ATLSEPQAHVPPVPPNEFTMLNSFWYSLAAFMQQGCDITPPSIAGRIAAAVWWFFTIILI

SSYTANLAAFLTVERMVAPIKTPEDLTMQTDVNYGTLLYGSTWEFFRRSQIGLHNKMWEY

MNANQHHSVHTYDEGIRRVRQSKGKYALLVESPKNEYVNARPPCDTMKVGRNIDTKGFGV

ATPIGSPLRKRLNEAVLTLKENGELLRIRNKWWFDKTECNLDQETSTPNELSLSNVAGIY

YILIGGLLLAVIVAIMEFFCRNKTPQLKSPGSNGSAGGVPGMLASSTYQRDSLSDAIMHS

QAKLAMQASSEYDERLVGVELASNVRYQYSM

>DmelGlu-R1B

MRFGLKLSCLWPSFLLWLTWSSGGGGGSGVGVSAQPSLTEKIPLGAIFEQGTDEVQSAFK

YAMLNHNLNVSSRRFELQAYVDVINTADAFKLSRLICNQFSRGVYSMLGAVSPDSFDTLH

SYSNTFQMPFVTPWFPEKVLTPSSGFLDFALSMRPDYHQAIIDTIQFYGWRKIIYLYDSH

DGLLRLQQIYQGLRPGNESFQVELVKRISNVSMAIEFLHTLEQIGRFENKHIVLDCPTEM

AKQILIQHVRDLRLGRRTYHYLLSGLVMDDRWESEIIEFGAINITGFRIVDTNRRLVREF

YDSWKRLDPQMSVGAGRESISAQAALMYDAVFVLVEAFNKILRKKPDQFRNNVQRRSQTL

MVAQAAASTSSDGYNYSASGGGGGNGGAGGGFAGSDSGGSGGMASRALDCNTAKGWVNAW

EHGDKISRYLRKVEIEGLTGDIKFNDDGRRVNYTLHVVEMTVNSAMVKVAEWNDDAGLQP

LNAKYVRLRPHVEFEKNRTYIVTTVLEEPYIMLKQVAFGEKLHGNNRFEGYCKDLADLLA

KELGINYELRLVKDGNYGSEKSSAHGGWDGMVGELVRKEADIAIAAMTITAERERVIDFS

KPFMSLGISIMIKKPVKQTPGVFSFMNPLSQEIWVSVIFSYIGVSIVLFFVSRFSPHEWR

LVQQQPQQSQSPDPHAHHEQLANQQPPGIIGGAPLPAPPGPPTPGAQTAAGAAALQAALS

AGSPGSGGSSSAVVNEFSVWNSFWFSLAAFMQQGCDLSPRSVSGRIAAASWFFFTLILIS

SYTANLAAFLTVERMVTPINSPEDLAMQTEVQYGTLLHGSTWDFFRRSQIGLHNKMWEYM

NSRKHVFVPTYDEGIKRVRNSKGKYALLVESPKNEYVNAREPCDTMKVGRNLDTKGFGIA

TPLGSALKDPINLAVLTLKENGELIKLRNKWWYEKAECSTHKDGETSHSELSLSNVAGIF

YILIGGLLVSVFVAILEYCFRSRDSRSASSGSGMGLGMGLGGGMSGGSLGKANGSMMLGP

SSAVPGGMPSSHQRSTLTDTMHAKAKLTIQASRDYDNGRVGYLNCASLQYYPPAQLSATP

PDAGDSLHMNAHGQV

>DmelGluRIIA

MRLCPVVIYAFIIIIGFLEGIIALGGDDRNEITVGAIFYENEKEIELSFDQAFREVNNMK

FSELRFVTIKRYMPTNDSFLLQQITCELISNGVAAIFGPSSKAASDIVAQIANATGIPHI

EYDLKLEATRQEQLNHQMSINVAPSLSVLSRAYFEIIKSNYEWRTFTLIYETPEGLARLQ

DLMNIQALNSDYVKLRNLADYADDYRILWKETDETFHEQRIILDCEPKTLKELLKVSIDF

KLQGPFRNWFLTHLDTHNSGLRDIYNEDFKANITSVRLKVVDANPFERKKTRLTKVDQIL

GNQTMLPILIYDAVVLFASSARNVIAAMQPFHPPNRHCGSSSPWMLGAFIVNEMKTISED

DVEPHFKTENMKLDEYGQRIHFNLEIYKPTVNEPMMVWTPDNGIKKRLLNLELESAGTTQ

DFSEQRKVYTVVTHYEEPYFMMKEDHENFRGREKYEGYAVDLISKLSELMEFDYEFMIVN

GNGKYNPETKQWDGIIRKLIDHHAQIGVCDLTITQMRRSVVDFTVPFMQLGISILHYKSP

PEPKNQFAFLEPFAVEVWIYMIFAQLIMTLAFVFIARLSYREWLPPNPAIQDPDELENIW

NVNNSTWLMVGSIMQQGCDILPRGPHMRILTGMWWFFALMMLSTYTANLAAFLTSNKWQS

SIKSLQDLIEQDKVHFGSMRGGSTSLFFSESNDTDYQRAWNQMKDFNPSAFTSTNKEGVA

RVRKEKGGYAFLMETTSLTYNIERNCDLTQIGEQIGEKHYGLAVPLGSDYRTNLSVSILQ

LSERGELQKMKNKWWKNHNVTCDSYHEVDGDELSIIELGGVFLVLAGGVLIGVILGIFEF

LWNVQNVAVEERVTPWQAFKAELIFALKFWVRKKPMRISSSSDKSSSRRSSGSRRSSKEK

SRSKTVS

>DmelGluRIIB

MHGLQFLVLLALAIASGANEDTLVIKIGAIFFDTEMKLADAFSAALEEVNAINPALKLDA

IKRYVTVDDSIVLQDISCDLIGSGVAAIFGPSSKTNSDIVEVLCNMTGIPHLQFDWHPQQ

SNRERMNHQLTVNVAPMELFLSAAFSDILASKTFDWKSFTIAYERSSHLIRLQHILAWKQ

LHKAGIKMQEFERGDDYRILWKRINNAREKFVLLDCPSDILVDVINASIGYNMTGSFNHL

FLTNLDTHLSGIDGFYSRDFTVAVAAVRIRTYVPPPVHDEIDVFDNSVDTRFSSLGSQLV

YDSIVLFYNALLEISQRPGFYIPNFSCGRGFWQPGPRLVEQMKQITPKMVKPPFKTQRLQ

INADGQREDFNLEVYNPIIDRVTHIWNKEFQLVDFEKLRENSTQALKQKRLQNKEDFSQK

PIRYTVATRVGKPYFSWREEPEGVHYEGNERFEGYAVDLIYMLAQECKFDFNFEPVRDNK

YGSYDANTDEWDGIIRQLIDNNAQIGICDLTITQARRSVVDFTVPFMQLGISILSYKEPP

PKADIYAFLNPYNAEVWLFVMIAMMITAFALIFTGRIDQYEWDQPVENVNREMERQNIWH

LSNALWLVLGSMLNQGCDLLPRGLPMRLLTAFWWIFALLISQTYIAKLAAFITSSKIAGD

IGSLHDLVDQNKVQFGTIRGGATSVYFSESNDTDNRMAWNKMLSFKPDAFTKNNEEGVDR

VKLSKGTYAFLMETTNLQYYVQRNCELTQIGESFGEKHYGIAVPLNADFRSNLSVGILRL

SERGELFKLRNKWFNSNESTCDSNVPTIDDGQFDMDSVGGLFVVLIVGVVVGLVIGVAEF

LWHVQRISVKEKIPPMLALKAEFYFVIRFWLTRKPLHTYRQSRDSTSTGYSSLEQITSAS

SAKKKKKTRRIEK

>DmelGluRIIC

MWQRILLLGCMWSAFFMCRSRGQQINIGAFFYDDELELEKEFMTVVNAINGPESEQTMRF

YPLIKRLKPEDGSVTMQEHACDLIDNGVAAIFGPSSKAASDIVALVCNSTGIPHIEFDIS

DEGIQAEKPNHQMTLNLYPAQAILSKAYADIVQNFGWRKFTIVYDADDARAAARLQDLLQ

LREVHNDVVRVRKFHKDDDFRVMWKSIRGERRVVLDCEPNMLVELLNSSTEFGLTGQYNH

IFLTNLETYTDHLEELAADNETFAVNITAARLLVNPDPPPYSLPYGYVTQRDNIVYESSD

PPRTLIHDLIHDALQLFAQSWRNASFFYPDRMVVPRITCDFAASGGRTWAMGRYLARLMK

GTSGVNNTNFRTSILQFDEDGQRITFNIEVYDPLDGIGIAIWDPRGQITQLNVDVKAQKK

MIYRVATRIGPPYFSYNETARELNLTGNALYQGYAVDLIDAIARHVGFEYVFVPVADQQY

GKLDKETKQWNGIIGEIINNDAHMGICDLTITQARKTAVDFTVPFMQLGVSILAYKSPHV

EKTLDAYLAPFGGEVWIWILISVFVMTFLKTIVARISKMDWENPHPCNRDPEVLENQWRI

HNTGWLTVASIMTAGCDILPRSPQVRMFEATWWIFAIIIANSYTANLAAFLTSSKMEGSI

ANLKDLSAQKKVKFGTIYGGSTYNLLADSNETVYRLAFNLMNNDDPSAYTKDNLEGVDRV

RKNRGDYMFLMETTTLEYHREQNCDLRSVGEKFGEKHYAIAVPFGAEYRSNLSVAILKLS

ERGELYDLKQKWWKNPNASCFEEPDPDATPDMTFEELRGIFYTLYAGILIAFLIGITEFL

VYVQQVALEERLTFKDAFKKEIRFVLCVWNNRKPIVAGTPISSVRTTPRRSLDKSLDRTP

KSSRRVVIGRSSEEMREMAQGSGSSSGSNNAGRGEKEARV

>DmelGluRIID

MHFCWISLIILSLSRVQAQFYGGNAYEASSGQSIRLGLITDDATDRIRQTFEHAISVVNN

ELGVPLVGETEQVAYGNSVQAFAQLCRLMQSGVGAVFGPAARHTASHLLNACDSKDIPFI

YPHLSWGSNPDGFNLHPSPEDIANALYDIVNQFEWSRFIFCYESAEYLKILDHLMTRYGI

KGPVIKVMRYDLNLNGNYKSVLRRIRKSEDSRIVVVGSTTGVAELLRQAQQVGIMNEDYT

YIIGNLNLHTFDLEEYKYSEANITGIRMFSPDQEEVRDLMEKLHQELGESEPVNSGSTFI

TMEMALTYDAVRVIAETTKHLPYQPQMLNCSERHDNVQPDGSTFRNYMRSLEIKEKTITG

RIYFEGNVRKGFTFDVIELQTSGLVKVGTWEEGKDFEFQRPPQAVNFNDIDDGSLVNKTF

IVLISVATKPYASLVESIDTLIGNNQFQGYGVDLIKELADKLGFNFTFRDGGNDYGSFNK

TTNSTSGMLKEIVEGRADLAITDLTITSEREEVIDFSIPFMNLGIAILYVKPQKAPPALF

SFMDPFSSEVWLYLGIAYLGVSLCFFIIGRLSPIEWDNPYPCIEEPEELENQFTINNSLW

FTTGALLQQGSEIAPKALSTRTISAIWWFFTLIMVSSYTANLAAFLTIENPTSPINSVKD

LADNKDDVQYGAKRTGSTRNFFSTSEEPIYIKMNEYLNAHPEMLMENNQQGVDKVKSGTK

YAFLMESTSIEFNTVRECNLTKVGDPLDEKGYGIAMVKNWPYRDKFNKALLELQEQGVLA

RLKNKWWNEVGAGVCSAKSDDDGPSELGVDNLSGIYVVLVIGSIISIIISILCWCYFVYK

KAKNYEVPFCDALAEEFRIVIRFSENERPLKSAQSIYSRSRNSSQSIESLKTDSEENMPV

ED

>DmelGluRIIE

MFFNHFVILWSLFSIHISVNWAQYENFGGYDNYQSLESVPIGLLTDQNTEQMNIVFDHAI

DVANQEVGTSLTSLKEEVNYGDAYQSYGKLCRMLETGIAGVFGPSSRHTAVHLMSICDAM

DIPHIYSYMSENAEGFNLHPHPADLAKALYSLITEFNWTRFIFLYESAEYLNILNELTTM

LGKSGTVITVLRYDMQLNGNYKQVLRRVRKSVDNRIVVVGSSETMPEFLNQAQQVGIINE

DYKYIIGNLDFHSFDLEEYKYSEANITGLRLFSPEKMAVKELLMKLGYPTDQDEFRNGSC

PITVEMALTYDAVQLFAQTLKNLPFKPMPQNCSQRTESVRDDGSSFKNYMRTLRLTDRLL

TGPIYFEGNVRKGYHLDVIELQPSGIVKVGTWDEDRQYRPQRLAPTTAQFDSVDNSLANK

TFIILLSVPNKPYAQLVETYKQLEGNSQYEGYGVDLIKELADKLGFNFTFVNGGNDYGSY

NKSTNESTGMLREIMTGRADLAITDLTITSEREQALDFTIPFMNLGIAILYLKPQKATPE

LFTFMDPFSEEVWWFLGFSFLGVSLSFFILGRLSPSEWDNPYPCIEEPEELENQFTLGNS

IWFTTGALLQQGSEIGPKALSTRTVASFWWFFTLIVVSSYTANLAAFLTIEKPQSLINSV

DDLADNKDGVVYGAKKTGSTRNFFMTSAEERYKKMNKFMSENPQYLTEDNMEGVNRVKTN

THYAFLMESTSIEYNTKRECNLKKIGDALDEKGYGIAMRKDWPHRGKFNNALLELQEQGV

LEKMKNKWWNEVGTGICATKEDAPDATPLDMNNLEGVFFVLLVGSCCALLYGIISWVLFV

MKKAHHYRVPLRDALKEEFQFVIDFNNYVRVLKNSASIYSRSRQSSMSVASVAQESQ

>DmelIR100a

MATTLQLIMLALVGGTLGQANNTDHKQVLTSIVKQLEGGLELHLRTSEDGGNDLVQFLMQ

EKSSIIISAKQEEVPSRAKIMRHHFFIFDGVHQMQEIRTSLFNTDGFYILALENNTIEDD

VLLMEFAADVWLQHGHSRIYYVQLSKKSVLLFNPFLQRLVVVQDSKTYSRIYKDLEGYHL

RIYIFDSVYSSVIGDGENKVLSVTGADAKLAKTVARQLNFTADFVWPDDEFFGGRLANGE

YSGGVGRAHRGEVDIIFAGFFIKDYLTTHIQFSAAVYMDELCLYVKKAQRIPQSILPLFA

VHMDVWLCFLLVGLLGALVWLILRAVNLILGIEGVPDGSRATRISYFGAARRIFVDTWVI

WVRVNVGRFPPFHSERIFVASLCLVSVIFGALLESSLATVYIRPLYYRDVNTLRELDESG

QPIYIKHPAFKDDLFYGHNSEVYRRLDAKMMLVAEGEERLIEMVSKRGGFAGVTRSASLQ

LSDIRYVMTKKVHKIPECPKNYHIAYVLPRPSPYLEEVNRIVLRLVAGGIVGLWTGEAKE

RAKWSIQRFPEYLAELDVGRWKVLTLSDVQLAFYALTIGCLLSAIVCMAEILLGRQRRLH

SPK

>DmelIR10a

MAVLGTVFLLFMLDLKTLNLTRLNGLLVEPTRDLPQLELWLRAGSDHQDAENPYVQWFLL

RTEIPLSIVTYQENRYWMDDPFGRRNLVLVMSLDQLLTNRGAAAPIQKASTFFYILADQD

KDLSADEQLRLEGSCRQLWTQHKVYNRFFLTRDGVWIYDPFKRRDSAFGRLVRYYGSETL

DKLLFRDMAGYPLRIQMFRSVYTRPEFDKETGLLTRVTGVDFLVAQMLRERLNFTMLLQQ

PEKKYFGERSANGSYNGAIGSIIKDGLDICLTGFFVKDYLVQQYMDFTVAVYDDELCIYV

PKASRIPQSILPIFAVGYDIWLGFVLTAFACALIWLTLRVINLKLRIVSLGNQHIVGQAL

GIMVDTWVVWVRLNLSHLPASYAERMFIGTLCLVSVIFGAIFESSLATVYIHPLYYKDIN

TMQELDESGLKVVYKYSSMADDLFFSETSPXWNRDLRADVIDEVARFRNKAGVSRYTSLI

LESSHFTLLRKIWVVPECPKYYTISYVMPRDSPWEDAVNALLLRFLNAGLIVKWIQDEKS

WVDIKMRSNILEADAESELVRVLTIGDLQLAFYVVIGGNLLAFLGFLAEHFRWKLQKKGV

>DmelIR11a

MRFAILWLFSGCLLPGIQVGIWVVVRAQPTGRDVLLSRLGNQQNELNTRRLANASSYLTR

NYIANRINTLVVREICVECPYELSERQRQLVDQILASLAPELSVLLHKGTAEETTWEYTL

FVVNDHTAFTGQVFIFPDELLEREFFCIVVVSEIQSRQFVRQTVGSIVKSNLQMHFVNVV

VVAQLEDGTVGTYSYKLFKANCTPGITVRQINHFDRITGKPQQSMPDLYPVRNGHLGDCP

FNVGAAHMPPHLIYKRHKDPPPASNVSIPAEDLAGIDWDLLQLLAKALKFRIQLYMPQEP

SQIFGEGNVSGCFRQLADGTVSIAIGGLSGSDKRRSLFSKSTVYHQSNFVMVVRRDRYLG

RLGPLILPFRGKLWGVIIVILLLAVLSTCWLRSRLGLSHPIEDLLTVIVGNPIPDHRLPG

KGFLRYLLASWMLLTLVLRCAYQARLFDVLRLSRHRPLPKDLSGLIKDNYTMVANGYHDF

YPLELTCRQPLDFSARFERVQRAAPDERLTTIALISNLAYWNHKHPNISRLTFVRQPIYM

YHLVIYFPRRFFLRPAIDRKIKQLLSAGVMAHIERRYMQYENKRKVASNDPVLLRRITKS

IMNGAYRIHGLVIVLATGMFILELLAGRSNGRLRRWMEWVHQ

>DmelIR20a

MLASLNRSTGLSAELLDLYGLVVHFLLSGEHTTLVYFNPAGLDCSWGVLWQRNLTAHPQI

VWQRNYSYPDLYYQFNAKLLVLACLPMDSRAAIQLEILANSLSHLRTVVRLLIEVAGPDQ

VTLARQYLSFCLRRSMLHVELYFRDYHHSLILYSFRAFPSFELVMRWISVGQGVKLFLHK

LDDLRGHRLRVIPDLSPPNTFFYRDARGDNQVTGYLWDFLATFAGRLNAGLEVVRPSWRA

GSASDSSYMLEYSAKGLIDVGLTTTLITKWNLWAIHQYTYPLLVSSWCTMLPVEKPLATP

DLFGRIVCPTLAMTLLLIILVTWLVFRQLRCLTRLKNSRPARIVPHLLTLLLLTTCSAQL

LSLLIFPPYHVRIASFEDLLRGDQKILGMRNEFYNFDGAFRARYAGVFYLIDDPNELYDL

RNHFNTTWAYTMPYIKWLVIKTQQRHFSKPLFRWSKDLCFFDFMPTSVIVAPDSIYWESI

KDFTFRIHQAGLMKHWIRKSFYDMIKAGKMSIKDYSDLETLKPLNIGDLEIVWRVCGAAI

AVASAIFIMELLYFYINVFFNSL

>DmelIR21a

MSYYWVALVLFTAQAFSIEGDRSASYQEKCISRRLINHYQLNKEIFGVGMCDGNNENEFR

QKRRIVPTFQGNPRPRGELLASKFHVNSYNFEQTNSLVGLVNKIAQEYLNKCPPVIYYDS

FVEKSDGLILENLFKTIPITFYHGEINADYEAKNKRFTSHIDCNCKSYILFLSDPLMTRK

ILGPQTESRVVLVSRSTQWRLRDFLSSELSSNIVNLLVIGESLMADPMRERPYVLYTHKL

YADGLGSNTPVVLTSWIKGALSRPHINLFPSKFQFGFAGHRFQISAANQPPFIFRIRTLD

SSGMGQLRWDGVEFRLLTMISKRLNFSIDITETPTRSNTRGVVDTIQEQIIERTVDIGMS

GIYITQERLMDSAMSVGHSPDCAAFITLASKALPKYRAIMGPFQWPVWVALICVYLGGIF

PIVFTDRLTLSHLMGNWGEVENMFWYVFGMFTNAFSFTGKYSWSNTRKNSTRLLIGAYWL

FTIIITSCYTGSIIAFVTLPAFPDTVDSVLDLLGLFFRVGTLNNGGWETWFQNSTHIPTS

RLYKKMEFVGSVDEGIGNVTQSFFWNYAFLGSKAQLEYLVQSNFSDENISRRSALHLSEE

CFALFQIGFLFPRESVYKIKIDSMILLAQQSGLIAKINNEVSWVMQRSSSGRLLQASSSN

SLREIIQEERQLTTADTEGMFLLMALGYFLGATALVSEIVGGITNKCRQIIKRSRKSAAS

SWSSASSGSMLRTNAEQLSHDKRKANRREAAEVAQKMSFGMRELNLTRATLREIYGSYGA

PETDHGQLDIVHTEFPNSSAKLNNIEDEESREALESLQRLDEFMDQMDNDGNPSSHTFRI

DN

>DmelIR25a

MILMNPKTSKILWLLGFLSLLSSFSLEIAAQTTQNINVLFINEVDNEPAAKAVEVVLTYL

KKNIRYGLSVQLDSIEANKSDAKVLLEAICNKYATSIEKKQTPHLILDTTKSGIASETVK

SFTQALGLPTISASYGQQGDLRQWRDLDEAKQKYLLQVMPPADIIPEAIRSIVIHMNITN

AAILYDDSFVMDHKYKSLLQNIQTRHVITAIAKDGKREREEQIEKLRNLDINNFFILGTL

QSIRMVLESVKPAYFERNFAWHAITQNEGEISSQRDNATIMFMKPMAYTQYRDRLGLLRT

TYNLNEEPQLSSAFYFDLALRSFLTIKEMLQSGAWPKDMEYLNCDDFQGGNTPQRNLDLR

DYFTKITEPTSYGTFDLVTQSTQPFNGHSFMKFEMDINVLQIRGGSSVNSKSIGKWISGL

NSELIVKDEEQMKNLTADTVYRIFTVVQAPFIMRDETAPKGYKGYCIDLINEIAAIVHFD

YTIQEVEDGKFGNMDENGQWNGIVKKLMDKQADIGLGSMSVMAEREIVIDFTVPYYDLVG

ITIMMQRPSSPSSLFKFLTVLETNVWLCILAAYFFTSFLMWIFDRWSPYSYQNNREKYKD

DEEKREFNLKECLWFCMTSLTPQGGGEAPKNLSGRLVAATWWLFGFIIIASYTANLAAFL

TVSRLDTPVESLDDLAKQYKILYAPLNGSSAMTYFERMSNIEQMFYEIWKDLSLNDSLTA

VERSKLAVWDYPVSDKYTKMWQAMQEAKLPATLDEAVARVRNSTAATGFAFLGDATDIRY

LQLTNCDLQVVGEEFSRKPYAIAVQQGSHLKDQFNNAILTLLNKRQLEKLKEKWWKNDEA

LAKCDKPEDQSDGISIQNIGGVFIVIFVGIGMACITLVFEYWWYRYRKNPRIIDVAEANA

ERSNAADHPGKLVDGVILGHSGEKFEKSKAALRPRFNQYPATFKPRF

>DmelIR31a

MNLLISMFILILAAGEGEIIPSMEESVVTNFVKSLVKTKQAIVFSCLFKDFKEISLALMR

INQFVSVVNLNQSYSLTSILTRENYARTSVMVNARCSGSSELLFEASENRYFNKTYQWFL

WGVDLEVQSLFPLNLNYVGPNAQITYVNETADGYAYWDIHSKGRHLKSNLEINLIATLIN

DTLNIARDIFHLQSIDFRGQFNGLTLRGASVIDKEDIISNEQIESILSRPTKDAGVAAFI

KYHYELLGLLRERFNFTVNFRNSRGWAGRLGNTTFRLGLLGIVMRNEADIAASGAFNRIN

RFAEFDTIHQSWKFETAFLYRYTSDLDTHGKSGNFLSPFSDRVWLFCLLTLGAFSIIWVL

FEIIDYKILRIRVNSQKLEHLNQKSSVICIKTTCIERILQTFGACCQQGLDPNPVDRSVR

FLVMTLFLFSLVMYNYYTSSVVGGLLSSSDQGPSTVDEITASPLKISFEDIGYYKVLFRE

SQNRSITRLIEKKLSSSRSLNELPIFSHIEDAVPYLKAGGFAFHCEVVDAYPVISEYFDA

NEICDLREVSGLMEVEILNWILHKNSQYTEIFKTAMCNAQEKGFVERILRRRQIKKPACQ

SLYTVYPVSLSGVLPGFVILICKSINKFS

>DmelIR40a

MHKFLALGLLPYLLGLLNSTRLTFIGNDESDTAIALTQIVRGLQQSSLAILALPSLALSD

GVCQKERNVYLDDFLQRLHRSNYKSVVFSQTELFFQHIEENLQGANECISLILDEPNQLL

NSLHDRHLGHRLSLFIFYWGARWPPSSRVIRFREPLRVVVVTRPRKKAFRIYYNQARPCS

DSQLQLVNWYDGDNLGLQRIPLLPTALSVYANFKGRTFRVPVFHSPPWFWVTYCNNSFEE

DEEFNSLDSIEKRKVRVTGGRDHRLLMLLSKHMNFRFKYIEAPGRTQGSMRSEDGKDSND

SFTGGIGLLQSGQQADFFLGDVGLSWERRKAIEFSFFTLADSGAFATHAPRRLNEALAIM

RPFKQDIWPHLILTIIFSGPIFYGIIALPYIWRRRWANSDVEHLGELYIHMTYLKEITPR

LLKLKPRTVLSAHQMPHQLFQKCIWFTLRLFLKQSCNELHNGYRAKFLTIVYWIAATYVL

ADVYSAQLTSQFARPAREPPINTLQRLQAAMIHDGYRLYVEKESSSLEMLENGTELFRQL

YALMRQQVINDPQGFFIDSVEAGIKLIAEGGEDKAVLGGRETLFFNVQQYGSNNFQLSQK

LYTRYSAVAVQIGCPFLGSLNNVLMQLFESGILDKMTAAEYAKQYQEVEATRIYKGSVQA

KNSEAYSRTESYDSTVISPLNLRMLQGAFIALGVGSLAAAALNNTINVRSLNSRDKFICG

GPVKIWYYLVLLLWYYFNRGLVGIYQLWHKTSIRNTGKGMPFLGE

>DmelIR41a

MFIDLSWSLVLSAIVGKYLNESTICIFWNDKFEFQLLHKSDYISFVGINIKSFDDNGGHY

IIDTGLKKKELQNKHLFLDELVIKIIISIEVTHCETFVVFDKDIDRFVNAFNKASVYSIW

RSLHNKFVFAHIANESPESRNHFFEDQPNILFVVRDHSSASSFDIKTNKFVGRKAENPSQ

MILVDRYLASEQRFQFGKSLFADKLNNLQGREVIIAGFDYPPYTVIKHNMSTNAQDMGVS

GESDFKNVYIDGTETRIVLNFCEQFNCTIQIDSSAANDWGKVYPNMSGDGALGMLINRKA

DICIGAMYSWYEDYTYLDLSMYLVRSGITCLVPAPLRLTSWYLPLEPFKETLWAAILLCL

CAEATGLVLAYKSEQALYVLPGYREGWWTCTSFGVCTTFKLFISQSGNSKAYSLTVRVLL

FACFLNDLIITSIYGGGLASILTIPSMDEAADTVTRLRFHRLQWAANSEAWVSAIRASDE

ALVKDILYNFHIYSDDELLRLAQDQHMRIGFTVERLPFGHFAIGNYLGPQAIDQLVIMKD

DIYFQYTVAFVPRLWPLLDKLNTLIYSWHSSGFDKYWEYRVVADNLNLKIQQQVQETMTG

TKDIGPVPLGMSNFAGFIIVWILGSAIATLTFLLELSLTYILKQSNLK

>DmelIR47a

MRQIKLLVWLLVVGVVSSTEQLQFLKNFLEAVHKERSISTILLIQRKVHKNDFLHGLYPI

FWPIICLDETKRVELVNNFNKDFLALVYMESEADTLLLSALAADLNHIRDARIMIWLQMS

PSENFLDRIVFQASKQKFLNLVVIENTLKTRRFYPFPQPKVQVIDKPFEEKEIYPALWRN

FMGKNAIAVPDLVPPRSFNSFDPKTGHRRESGSIYNVFKAFTQRYNITMLLKWPLIRNTT

QEEIIGKSVRGEIDLPITGQLISFRHPNGSRSQPLLGMTALSIAVPCGPELPMFDRFFLF

YGLATPITITGYYVLLNTIEIILGTLSDRIKRHPRRKKILNLVLNLRVFSCILSLPTPQG

NRLRSVKGQLTMVMSITGLILSCIVAAQTSTILTMKPQYRHIKNFQELSDSNITVVCNHL

NYLTIKQQMDPKFMAKFMQNIWIVNSIEQMKMIFDLNTSYAYQTFSYKKDPFTLLQMHTT

RKAFCRTPGLDLVSGLAYTAVLEKNSIYALALQDYTLKAFSAGLVYYWAEESIRDLISTV

GRTQFEKLPIVIGYQSLKLQDYNVCWKILLIGGALAFCVFIVEVVVGLINRRI

>DmelIR47b

MREAQIIIFLLTSAAAVTLKQYEFLXSFLKAGEQEQTITTLLMMQKHVHTKNLLQGLYPX

PWPIIHFVETQRIKFIALLYMSSEKDIFLSSLAANLKFERLDKPFGKSNIFPVLWRNYMG

XIALTLDHLVEPRSFYWTDPRTNIKRRTGYIYMLITNFAEQHNITLQLXSPPNEDMSQMV

IIERTHKGPRSTHNWADDQLETFERXQDSLLPWHGSMAIVVPCGQEMSAYERFHAAHAFR

APIIFFGFHIFLSLIDFLLRTISDRIRCNPRRIQLLQTVLSLCVLRCILSTSLPNSNXLR

SRLRDNSPXXXVLQAXSYSALWXLTGTAXQXHNRDFQSHKLHDYXTTDGSXHSIEVPGLL

KARNXXIXLFHIFSSLGTKFDLRIGSAGSHTSGVEFRYYELLDRXSSLENNIVSQVFTIL

KLPYSRFRVLKLEDCRGCWQTLFVGFSIATFVFIVNVLMGFFRNINQKK

>DmelIR48a

MHLLITETYMIIGKTLHDILNELNERLIISTNIIFCKQFDNLIHFEAQTSRFVYSSLEAF

NITSLWNHVGNDNKLFVIVGNVPPYELFAKLELSSPENCTQFILNNTVDMCADALVKNSK

AFSVSRELRIAPANVIVPHGKPLLSYRYLAAPFNTKVWIALGTYVFLISGFLCLIHWLRS

GKWDFSQNLLEVYSSLLFTVFHLKATNGIERYILFGVLFISGFVYSTSYLRLLKSMLIAE

TFEKQIQTFEELAESNIPLLINPYDRMIFQHHHIPKSLWTAVRTVSSETLLNHRSHGYVR

LCPAILTASKIPSHTHRHLFSVCRFSHEQEVVPKGSSXXSLVPCIRKRNREXNHLGCLSG

VSWPGISXFFHYGALGGEAFGSILLHDANYFPSPRLFRRLAELHYGSY

>DmelIR48b

MILQQSSNLLKLLLLLAISSVRTQGLNDIIIELNQRLLISNNFLYCNQSDKLNEYEIKYL

QHMPPISLMIFTSIESMNFTQVEYNLGADNKLFLIMGNEEPPYDFLHALNLHFQFAEYII

VIDEPVDLKKSTKWLDFVNHLWQQGYVQLLIYTSYDEKLYHKIIFPETVIEETLVEQYIS

IRGSFNNLYGYPVRVAAYNNAPRSMLYVNRWGKHIFAGFYMRFLRAFIDARNGSFVPVLT

PSNSPGNCTLNLVNETVDVCADALAANPAAFSLTHGFRIASANVLVTHAKPLHSYRYLTA

PFQWSVWACLVIYVLLVVNFLSFIGWLRSGKWEFSKYLLEVFSSLLFSGFYLKEIRGRER

YILFGVLFIAGFVYSTEYLGLLKSMLISEVFEKQIDTFEALVESNITLMVDPYDKILFAK

YNMPEILSPIMELVSFETLLKHRNRFDQDYAYILFSDRMALYDYAQQFLKHPKLLRIPID

FSFLYTGIPMRKRWFLKHHLGRAWYWAFESGLTRKLALDADFEAVRVGYLSFLITEHVEA

QPLNVDYFVMPAIALAIGYILALLSFVIEMTAWRIREFLGCRKATMTSTGCSEGGHVDVD

>DmelIR48c

MSLLRIILIIIFLRIVSSIPDTIISHLSAELQIKIQIYFGLGNDLYDFSRLDGNYQKIII

SHNISEEFKTYHDEPVLIIIRLERDLNLNLATLDVLRSYLTDRQYNDILLIDNDEENLNS

YVDIRKAYWNAGFSQVLIYNSQQRTWSIKPYPYLQIRPTSLKEYIENRNTRNLMGYPLRV

LVTNDPPHCFVDKDELPGSPNRYKGSIVTMLKIFADQLNATFQANPFREFRRYSTADCVQ

MVSDDEIDACGSIFIRTYTYATSQPVRLNRVVIMAPFGNPIEKFYYFFRPFDLYVWIGTG

IIVVYIAVMGSLLHRWHFKEWNVGQYLLLAVQTLLNRELSLPQSSSGSKFMLLLLLFAIG

FILSNLYVALLSMMLTTKLYQRPIENLADLKAANVNILLQTHNIRPNSVYGSSEELRERF

LLVEESQHLEKRNGLDPSYAYVDSEDRMDFYLYQQKFLRRRRMKKLSNPVGYTWAVQVIK

QNWVLEKHYNDHVQRFFETGLQNKLVDDVHELAVKAGFLHFFPTQTQTIEPLRLEDIVMA

AMVLGGGHALAVICFLVELFA

>DmelIR51a

MYNVLVLFLLLFTRAQMEPHRRGHNMTLLRSVLTVIRGRENWKNTPIFLGGHCNSDDLNN

LMSWLQNTMEVTCHTVDTSTSAKNENALGHFNINADNSLGLLFCQSSHELIWFNMDKRLR

RLRGIRLIVILSDKRSSSSKAIMSTFKRLWHFQFQXNFQGYVVSTPVENDIPRVFFVKDK

KTGRKQIRGFGYRTFVEYLHRYNASLHVSNSQQEHAINSSVNMGRIINQIVDGQLEISLH

PYVDVPENMGDNSYPLLIASNCLIVPVRNEISRYMYLLLPLNQSSWILLLGSVIYISGVL

YYIQPGLLHRTWDQRIGLNILDSISRIINICSPSRIYNPSLRYFIVSVHLSILGFVVTNL

YSIMLGSFFTTLVVGEQVDSMQQLIQXQQKVLVKYYEVSTFLRHVEPDLVDGVAQLLVGV

NASEQVSALLGFNRSYAYPFTLERWEFFSLQQQYAFKPIFRFSSACLGSPIIGYPMKSDC

HLQSSLNMFIMRIQAAGLLRHWVVSDFNDAMRAGYVRLLENFLGFHSLDVDSLRLRWAVL

LCGWLLSTLIFLCER

>DmelIR51b

MCKVLTLLVVILLLALTNAAYNVTLLKSVLSLISTREPWINTPIFVGHNTQGGDLNDLII

WLHQTMGVTSLTMNLFLQPEHIRPLGHFKITRYNGIALFFCHDKHDIMWLTLDRNLRKLR

RIRLIIILRNQRSGSQGAIKSIFNALWQYQFLNVLVLQRDQLYSYTPYPAMRFFKLDIHT

EPLFPHAARNFHGYVVSTPAENDIPRVFHVHDPLTKSRKVLGYAYRTFVEYLDHYNASLR

LTNPDENLDPTTSVNMNHIVQLIIDGQLEISLHPYVFTPPTATKSYPLLIYPNCLIVPMR

NEIPRHMYLLRPFQLYSWYILLFAVFYITGILYCISPKLNKSSWPQRLGLNFLDAISKIL

FISPPITIYRPTWRHLIIFLQLSVLGFMSTSWYNIELDSFFTTIVVGEQVNSMDQLVHQQ

QRVLVKEYEINTFLRHVEPRLVEKVSRLLVPVNASEQVSALLSFNRSFAYPFTEERWQFF

AMQQQYAFKPIFRFSSACLGSPHIGYPMRVDSHLETSLNHFILKIQDTGLLNHWVVSDFN

DAMRAGYVRFVDNVLGYQSIDVDTLRLGWCVLGIGWILSALVFSCEYWHLYPWRFIA

>DmelIR52a

MALGWSVIILGFIGQLSAQILNYTQSRDLELLEGSLFRVLSRLNLEEEYNTLLIYGKECV

FHSLLRKLEISAVTVPSGSTDYDWSFSTAILILSCGYDAENEENSYTLMKLQRTRRLIYL

EDNSEPESVCMRYSLKEQHNIAMVKSDFDQSDTFYSCRLFQTPNYVEGHFFKDQPIYIEN

FQNMRGATIRTVADSLVPRTILYRDEKSGETKMMGYLGHMINTYAQKLNAKLHFIDTSKL

GAKKPSVLDIMNWVNEDIVDIGTALASSLQFKNMDSVWYPYLLTGYCLMVPVPAKMPYNL

VYSMIVDPLVLSIIFVMLCLFSVLIIYTQHLSWKNLTLANILLNDKSLRGLLGQSFPFPP

NPSKHLKLIIFVLCFASVMITTMYEAYLQSYFTQPPSEPYIRSFRDIGNSSLKMAISRLE

VNVLTSLNNSHFREISEDHLLIFDDLSEYLVLRDSFNTSFIFPVSVDRWNGYEEQQKLFA

EPAFYLATNLCFNQFMLFSPPLRRYLPHRHLFEDHMMRQHEFGLVTFWKSQSFIEMVRLG

LASMEDLSRKRNEEVSLLLDDISWILKLYLGAMFISSFCFILEILRCGERCKRLWRCRW

>DmelIR52b

MTWLVILLCFLGYMAAHIADISVQNQSLMDNELINLLLKLRNEEFYDTLLVYGKDCEFHS

VIKNVDVAVVLVSDSMNFEWNFSSLTLILSCGPDIDNGGPNSTSIKLQRNRRLVLLKEDF

QPSNICNIYTQKEQYNIALVRENFTKSKSIYTCRYFQDPNVDEVNLSGTKPIFIEQFQNM

KGKAIRIVPDLLPPRVMLYQDANDGELKMIGYVANLITNFAQKVNATLQLDFLKPSTSIT

EISRMAKDDELDMGITLEASLNTSNLETSSYPYLLTSYCLMVQVPAKFPYNLVYALIVDP

LVLGIIFVLFLLLSVLLIYSQKMSWQDLSVANILLNDKSLRGLLGQSFPFPLNASKKLRL

IFTILCFASIMLTTMYEAYLQSFFTNPPSEPEICSFQDVGSYNRRIAMSALEVNGLIKTN

NSHFREIRMDDLEIFDNMPECYELRDAFNLSYNYVVTGDRWRSYAEQQTLFKEPVFYFAR

DLCFSRLIFLSVPLRRHLPYRHLFDEHMMQQHEFGFVNYWMSHSFFDMVRLGLTSLKDLS

RPLAYTPSLLMDDISWIMKIYLAAIVLCVFCFLLEIGVDKWKRWMKFRNLQILNTC

>DmelIR52c

MVWLIIILFCLGNSSSQILDVTNNSHLDFDYRLFGLLQRLQVEKSYDTLLVYGEDCAIPS

LFERLQVPAVLVSSGSTNFDWNFSSLTLILSCNFQDEREENYRTLMKLQTSRRLILLKGH

IKPESVCDFYSKKEQHNVAMVKENFYQLEVVYSCRLFQDQNYEKLNLFDGKSIYKDQFRN

MHGAPIRTLSDKEPPRTIPYIDSKTGEEKFKGYVGMLISQFVKKVNATMQIREDLIKDDE

EVSFVDITNFTSNDILDIGICEARTLEMSNYDAISYPYLMSSYCFMAPLPDSLPFSDVYM

AIVAPSILIMFLIIFCICSVLIIYIQERSYRSLTIRSVLMNDICLRGFLAQPFPFPRQYN

RKLKLIFMLVCFSSLISTTMYTAYLQAFLWGPPIEPRLTSFDDVKKSRYTMAINIYEREF

LEALNVSLEDVEIYDYGKFSKLRSTFNTNYLFPVTALQWFTINEEQKLFKYKIFYYCDAF

CLNQFDILSIPLRRHLPYRDIFEEHMLLQKEFGLTKYWIDQSYRDMIRANLTTFKDFSPL

LENDYIEVHNLYWVFTMYFVGMGMGLCFFILEILRPLRYWRNCKIKCEYCYAFLKNFAK

>DmelIR52d

MVRIIIILLCLGYTKARILDATNTNHTDLEERLLSLLLRLQQEQFFNTLLIYGEDCAFSS

LSRRLQVPTILVSSGSTSFEWNYSSLALILTCEFKAEREENYQTLKKLQMNRRLILLNGN

IKPDSVCDFYSKKDQYNIAMVNNNFHQVGIIYACRLFQERNYEKVYLSEGNPIYVDQFRN

MQGALLKSITFNLIPGSMAYRDPKTGQEKHIGYVANLLNNFVEKVNATLDMQVKLHKAGK

KTSFYNITKWASEDLVDIGMSYAAYFEMTNFDTISYPYLMTSTCFMVPLPDMMPNSEIYM

GIVDPPVLVVLIAIFCIFSVMLNYIKQRSWRSLSLVNVLLNDICLRGFLAQPFPFPRQSN

RKLKLISMLVCFFSVITTTMYTSYLQSFMWGPPIDPKMCSFADLENSRYKLAIRRYDIEM

LRPFNVSMDHVVVFDESSQLEYLRDSFDDNYMYPMSALSWSAFKEQQKLFAFPLFYYSEK

LCLKPISFFSFPIRRHLPYRDLFEEHMLQQNEFGLSTYWIDRSFSDMVRLKLATMNDFSP

PRLEDYIEVSDLSWVFGMYFTGLGISCCCFGLELLGLPSWTRRLRLTNWLRVRN

>DmelIR54a

MWTVITGIVLWAPVLVAGSAVDFIFRAAAEHSLSVIMIRIDYCPYNWAKDIFENQTIPVV

VLSDSETFINIRMFSRPLHVACLPGHELQKDLALLENFTSSLMDFPSQKKIVYISNNFSD

PTRMDYIFETCYHRRIWNIVGLLASDEHRYFYRYHLYPSFRTEYRSLESSTIFDKDFPNM

HGHPLTVMPDQWLPRSVLYVDRRTGKQILAGSVGRFFHVLSWKLNATLQLSKKVTTGRFL

NATALKELSESFSVDVPASLTIMERVEQLASTSYPMEVTHVCLMVPVARRIPIKDIYFIL

SSASNMFLAIVIVSSYGLALNLLRNMTHRDVRLVDFVLNDKALRGILGQSFNLPLSRSFS

TRLIFLMLGIVGLNVSSIFGAGLDTLMAHPPRQFQARSFAGLRRTKIPLVTTEEDFPTWM

KLRVPMLVVNVSEYNHLRNGRNTSNAYFASRLYWNLFSEQQKRFTRELFIYSTDDCLWSL

ALLSFQWPQNSLFTEPVSQLILEVNANGLYDFWVGMHYYDMTAAGLSGLEDPSLQLKERE

HPTSLRIVDFQWMWQAYGTFMVIAILVFLLEVSWHRITSLFVSLVY

>DmelIR56a

MGSRFFIRNLILFGLLASSNMQIPFGELEKKFELDVDFLLGVTELVGHIQGLYSITVYAD

CIDIHPSIQQRIMDKFMVPVNTIGSNLSRPNYHKLDNSRIRIVLFTGLNDTILVNLNKTD

VPYSDNFYMLAYASAIKNKCIELDFIEEVFTLLWKMSIQNAILLIRGEFMMEMWSYLYMG

KIHKIKLTKPNSYLESLRKYNYRFSLEVINDPPAIFWYNSSEQADVTGGGNLSVSGPLGL

IIINFLRHLNVTIDIVPIPGKQTSQYELFQQPDNLRAENGVNMVGSALLKYSPMVTQSRM

CLLVSNRRMIPFSRFLDRLVSPGVHKLTFVSSIGIFVIKYFSHRPRSFVDAIFCTIRFFF

AIPLPSIILNRLPVVDRFIEVFIIIFVQILLSSNISITTSALTTGFWEPPIINVETMRAS

GLHILTEDPTILQAFKENILPSSLADLVILVDEDTYFHHVTTLNNSYVYVVQAHNWQIFR

LYQQQMTNEPFEIASEELCSKWRILGIPLNPKSPLRFMFKDYFYRILESGLREQWVHSGF

KKFCEFNNLKKLPVDSVDSWQPLSIEFYSNVIRAYIIGLVIATLAFVAELLHNGYRRKNV

KKT

>DmelIR56b

MLLDTDLASGVIRSPYSFDIPHAFIFNETQFVVPKFCGPYMEIVKHFAEVYHYQLFLDSL

ESLPKKSVVEQDIISGKYNLSLHGVIIRPEETSDFFNATQHSYPLELMTNCVMVPLAPEL

PKWMYMVWPLGKYIWTCLFLGTFYVALLLRYVHWREPGNATRSYTRNVLHAMALLMFSAN

MNMSVKLKHASIRVIIFYTLLYIFGFILTNYHLSHMTAFDMKPVFLRPIDTWSDLIHSRL

RIVIHDSLLEELRWLPVEYQALLASPSRSYAYVVTQDAWLFFNRQQKVLIQPYFHLSKVC

FGGLFNALPMASNASFADSLNKFILNVWQAGLWNYWEELAFRYAEQAGYAKVFLDTYPVE

PLNLEFFTTAWIVLSAGIPISSLAFCLELFIHRRKQRRPQYERFECYDY

>DmelIR56c

MRSSFRICLFLLTTYHPSHGWNMQHLLNLLAPFGRMNVFQEIVWFVSPHQRLDQLDEFIM

RIDEAFGKSATQTVVNNNTEMRMIYSSARRNHMSFVFTTGAEDPIMKVFSKVLLGRHFYV

SMVIYVDKVGDMHPIYDLLTFAYNQQFFNSMVHFESMEGVNQLFGVSKFPVMSFENRTDF

LKYMGKIWKQVQNARSDVGGFGFTTPLRQDLPHLFQSQGHYDGSTYRIIETFVRFINGSF

KELIMPPDSLGGQVINMKDALQLIRERKMEFCAHAYALFMSDEELEKSYPLLVVQWCLMV

PLYNSVSTYFYPLQPFDWNVWFFALGALLALVLLELMWLRMFGGWSGYRGAVLNSFCYII

NVPIEGQLQQPCLLRFLLLATVFFHGFFLSAYYTSNLGSILTVNLFHAQINTMNDIVSAQ

LPVMIIDYEMEFLLNLNKELPQEFLELLRPVDSAVFSEHQTSFNSSFAYFVTEDHWEFLD

EQQKHLKQRLFKLSSICFGSYHLAFPLQMDSSLWRDIEYFTFRIHSSGLLNFYARSSFGS

ALHAGLVQRMPDTQEYTSAGLQHLAIAFILLLVMSFLAGIVFVLETLSR

>DmelIR56d

MDNRAAELILRERNIFPTNGSDNITLLNNMFVLEMFYRITQLYHFKNFIFYISERLDLNN

KDSQEFFHNFWTYFPMAPNLIITREHHLGIPMMQFISTPSLVMVFTTGKDDPIMELASHN

QQGIHWLKTIFVLFPSLQSRDFETNPESLAQFTAEIKDVYDWVWRKQFINTFLITIKDNV

FILDPYPTPSIVNKTGVWQAEEFFHKYAKNMKGYLVRTPILYDMPRVFKSDRPTNRYEKN

FIHGTSGNLFLGFLEFVNATLMDTSANVTADYLNMTNLLDLVSQGVYETLIHSFTEITTK

FVVSYSYPIGINDCCIMVPYRNQSPADQYMHEALQENVWVLISLFTLYITVAIYLCSPLR

PRDLSAAFLQSICTLTYSVPTFIIRTPTLRMRYLYILLAIWGIVTSNLYISRMTSYFTTA

PPVRQINTVQDVVEANLRIKMLAIEYERMAKSPLQYPESYLNQVDLVDKHMLDLHRDPFN

TSFGYTVSSDRWRFLNLQQLHLRKPIFRLTEICEGPFYHVFPLHKDSHMRSVMTEYIMIA

QQAGLMNHWERETFWEAVHLHRIHVHLFDDEPMALSLDFFSSLLRTWTLGLILAGLAFAA

EMKWHEHVTFKRRPVIRITRKPRSFLRRFMKL

>DmelIR56e

ERXAFRNQWAFCFPRTXAIEVVLSAWSPXCPGQRSKPQPISXPHHXGSCWRKRKWKXKPR

LLVVDKRTLVEHLNSLNDGYAYCIIAGHWQVGMM

>DmelIR60a

MWCNNPGLIIIIFLGQILNLCQGIVNLSNETANTVIFMLPEKDLGPDVWKAGVGCLDSFA

QIFFFRNPKERFTRAYNLMLVHAFHLSSPADQIQEGFSKLINEAVTNPGPPDREELFQMR

VASDYNITNGTEDKGELILADNYVIVVDSVDRLKELMKKKIVEMRSWNPGARFLVLFHNA

TCRNRPLGVASNIFKDLMEMFYVHRVALLYANSTMNYNLLVNDYYSNVNCRILNVQSVGQ

CHDGKLYPNNAVVKASMQDYVSGFSPRNCTFFACSSISAPFVEADCILGLEMRILGFMKN

RLKFDVNQTCSLESRGEMDGPANWTGLLGKVQNNECDFVFGGYYPDNEVADHFWGSDTYL

QDAHTWYIKMADRRPAWQALVGIFEAYTWIGFILILIISWLFWFTLVMILPEPKYYQQLS

LTAINALAVTISIAVQERPICETTRLFFMALTLYGLNVVATYTSKMIATFQDPGYLHQLD

ELTEVVAAGIPFGGHEESRDWFENDDDMWIFNGYNISPEFIPQSKNLEAVKWGQRCILSN

RMYTMQSPLADVIYAFPNNVFSSPVQMIMKAGFPFLFEMNSIIRLMRDVGIFQKIDADFR

YNNTYLNRINKMRPQFPETAIVLTTEHLKGPFFILVVGSCWAALTFIGELIIHRWRTQLV

STSEQQDRRSDKRRRRRRRRKPEKDNRWQRQVQVAPVVRFTPVKRRKVFQGQTSQK

>DmelIR60b

MRRSLYLIIAIGLVDVHCVSLRYILNALENELQYRAILLVESASEIESCWEQKYIQGAVP

ILNFNANQSLYLKDALNTNILALVCLNENVESTMQALYENLEDMRDTPTILFVLSDSKVQ

DVFLECLRRKMLNVLAFKGLDRGFVYSFRAFPTFRVIERNVMDILQYFEQQLEDLGGHTL

TTLPDNIIPRTVVYKSPDGSRQLAGYLYPFLRNYVSTINATLKVCWHLVPEDGMIQLGEV

VRLSEIHDVDFPLGMHGIEHGSTSQNVPLEVSSWFLMLPMEPSLSRAQFFIMLGFEKVTP

VLLLLTILLSTAHRIEMGLRPSWRCYVLGDRVLQGTLGQAFFLPRRLSVKLMLVYSLILL

NGFTFSNYSITSLETWLVHPPSGHPIHSWEQMRTLNLKVLIVPSELDSMTKALGKQFTES

NSDLFELSKSGNFQDKRLAMDQSYAYPVTCTLWPLLEHAQIRLPKPEFRRSREMVLIPLL

IMAMPLPKNSMFHKSLNRYRALTHQSGLYEFWFKRSFNELVALRKIHYKVNGDHQIYRDF

EWQDFSYVWLGFVGGTIASILVLLAEIGYHRWQLNQN

>DmelIR60c

MEMRLALFFTFACLAGAHDGSLRNMLKSLEDELGYRTILLLEGFVYSFKAFPTLRVVKRR

VKDVRRYFEPQLEDLGGCVLKVVPDGIMPRTMVYQGEDGELQMGGYLSHFIRNYVSTINA

SLQIRWDLFPEDGDFDMDSLTGSNHVDFPLGLGSLSFQTLHKDVAMEISSWFLMLPMEPS

LPRARFFIRFGISLYLIPLIILLAIVLSNAHRFEAGLTPSWRCCSMGNTVLRGVLAQAFV

LPKGLSPKLMFVYWLLLVSGFFVSNYVIVYLTAWLIQPPTSDPVTDFDQMRRAKLKILMV

PTDMDYLKSIRGAEYVDAHSDVFQTADSTDFQTQRMSMELHFAFSVTGTLWPLLRQAQVK

LHRPIFRRSKEMVFLPFVIMGMTMPNNSIFLSSLKQYRLRTSEAGLYLLWFKKSFSELVA

IHKISYKEDWVHDSYSDLKWEDFLFAWLGFLGGTTVSCLALLAEIGYHRWLWKRTHQ

>DmelIR60d

MRLAIYVAFLSSIGNRSGFLSSLLMSLGKELHYKTILLVGGSSTCWSLEPFETGVPILNL

RGENNAYPQDTFNSQMLALACLQTESEDAVKLLYRSLKDMRDTPTLLFASSEEHIHDTLF

LGCFRENMLNVLALTASSKEFIYSYQAFPTFRVIKRKLVEIHRYFEPQLKDLGGHIVSAL

PGNIMPRTMCYRNAEGERQLAGYLNTFIRNYVESINGTLRISWGLVPEDDMRHLTISRLS

KIQHVDFPLGIIPLYNKTDKQHVYMEISSWFLMLPMETSVPRAHLFVKLGLERLLPIIVV

VGAVLGNAHRIEVGLGPSWRCYYLADKVLRGALAQPIVLPRRLSPKLMLIYSLLLLSGFF

LSNYYMASLTTWLVHPPASDRILEWDQLRYLHLKVLTIPEEFKYMSLILGTDFMTAYGSI

FQLTNSTDFQRRRISMDPSYAYPVTTSLWPFLELSQVRLRRPLFRRSYDMVLQPFQVMSL

PLPRNSIFHKSLLRYAALTRETGLYYYWFRRSYYELVALGKISYKEEEGNPYCDLKWNDF

RIVWLAFLGGTIISCLALLLEVAHYRWHLGNSSL

>DmelIR60e

MVIKMISFLLVSVLLCLVGASDSESMQVQVLQDLNLALQTELNVFIDFECCATSEILHKL

DSPRILLSSNSREARDLRIRGNFTESTLIIVSVMDSDLNPLVASLLPRLLDELHELHIVF

LSNEEPGFPKQDLYTYCFKEGFVNVILMSGKGLYSYLPYPSIQPISLSNVSEYFDRARII

RNFQGFPVRILRSTLAPRDFEYSNEQGGLVRAGYLFTAVKELTYRYNATIESVPIPDLPE

YDVYLAVAEMLHTKKIDIVCYFKDFSLEVAYTAPLSIIREYFMAPHARPISSYLYYSKPF

GWTLWAVVISTVLYGTVMLHLAARGARVEIGKCLLYSLSHILYNCHQKIRVAGWRDVAIH

GILTIGGFILTNVYLATLSSILTSGLYDEEYNTLEDLARAPYPSLHDEYYRSQMKAKTFL

PERLRRNSLSLNATLLKAYRDGLNQSYIYILYEDRLELILMQQYLLKTPRFNMIRQAVGF

TLESYCVSNSLPYLAMTSEFMRRLQEHGISIKMKADTFRELIHQGIYTLMRDDEPPAKAF

DLDYYFFAFVLXTVGLISSLLVFFAELVSGHL

>DmelIR60f

MRFHLNIANSGLLGLHLCPTRSALPEQNPCFSKAGAVIXNLTLPWRRWRERCLLGALRPX

TLPTPELQCXSKYLPXRKSQQENASSGLPGFCXGDXQTELHRGSRAIALPRSPYHDLYYV

WIAYLGGTMIGIGMLAVEIACFKWDLLRRPPIXMY

>DmelIR62a

MYLQFLFALFLSRYQIVATENFDRAFELALFLDRIGRVHRLHAITIVNSLGSVDPSYLDD

LHRGLMCNSSNHFYMLPQMTATDKDSSHVHFSSLQDEETIYLVFARDSKDAVIYLQAERA

RGRRYTRTMFLLRKQESQKDIKYFFELLWKLQFRSALVVVAARNFYQMDPYPTVRVIRMR

RLSSYDPHHVFPPANRKNFRGYRMRLPVQQDVPNTFWYKNRRTKAWELAGLGGILINQLM

MHLNVTMDLFRFEVNGSSLLNMAALTDLIVKGKVELSPHLYDTLQSNTSVDYSYPTQVAP

RCFMIPLDNEISRSLYVFLPFSLTMWLCLLFVLLVVHFVYVRRLIPDGHFWAILGVPGAG

QVRYGNRKPVRRFSTFLILFGIFILGQTYSTKLTSSLTVTLIRRPDNSLEELFLLPYRIL

VLPTDVYAIVDSLGHAEQFSTKFSCTDAENFSQKRISMHPEYIYPISTIRWRFFDMQQRF

LRKKRFYFSKICHGSFPYQYQLRVDSHLKDALHRFLLHVQQAGLHDLWLDTCYRKAHRMG

YLKDFSTLAELEEKLRLRPLALNLLVPAFSLFLCGMLGSGIAFLVEIRHSFGCRQKPPSI

NRNPGD

>DmelIR64a

MHWWLLVFLPLSCQGLPEHELLELELDYGLAEPQRTSLLQSSLILQFSQDYKHIPRITYF

TCQKPHLQTPNQIPNAAEHRDAFAAKNFQLIKSLYESELFVRIVLLDVLAQSPTSGRPNR

PGNGPTGGFSQTPSQAQSNSEWLEGVLRMEALRQIAVVDLACGAVSRRFLELASAKMLYS

EKFHWLLIEDFAWHGRTQTAEGSGKRDDGEMEEEEPPGQQIQATDDEDLPSIESFLGGMN

LYMNTELTLAKRMSEAAHYTLFDVWNPGLNYGGHVNLTEIGSFTPTEGIQLHTWFRTTST

VRRRMDMQHARVRCMVVVTNKNMTGTLMYYLTHTMSGHIDTMNRFNFNLLMAVRDMFNWT

FVLSRTTSWGYVKNGRFDGMIGALIRNETDIGGAPIFYWLERHKWIDVAGRSWSSRPCFI

FRHPRSTQKDRIVFLQPFTNDVWILIVGCGVLTVFILWFLTTIEWKLVPHDGSALIKPKG

GAPPRHHYQQQQQQEQVEAPVRPITAVSVVVSKEKVEEKQEEYEDSTPIDAGTLWQRCYQ

KLNKYIKDRKAKQKKAPERVGLFLESVLFFVGIICQQGLGFSTSFVSGRCIVITSLLFSF

CIYQFYSASIVGTLLMEKPKTIKTLSDLVHSSLKVGMEDILYNRDYFLHTKDPVSMELYA

KKITSVPTTKENEADEDEPVDPNPVSTDPAKSYRDIVHSHETGAHAKDNAASNWLDPETG

LLRVKHERFAFHVDVAAAYKIIAETFSEQDICDLTEVSMFPPQKTVSIMQKNSPMRKVIS

YGLRRVTETGILTYHFNVWHSRKPPCVKKIETSDLHVDMDTVSSALLILLFSYAITLMIL

GTEILYSKWHNRIQLKWVGAT

>DmelIR67a

MLPILVPVLLLFNETSWINPILTSIYKDRHHETVLLLQHSQHGNASGLERFPWPVFSFNE

QMDFYVRGKYNSEMLVLIWQTGNSDWDLDLWQALDRSLLNMRKVRVLLLRKWEKIPTADV

AATAEHLLFLHVAVIGQGNRIYRLQPYAPQSWLQVDPIESPIFIKIRNYFGRYIVTLPDQ

FPPRSIVYRNPKTDEIQMTGYVYKFLLEFIRIYNFTFRWQRPIVQGERMNLILLRNMTLN

GTINLAISLCGFETPSXLGVFSDVYDMEEWYIMVPRAQEISIADVYVVMVSGNFLIVLII

FYFIFTILDTCFGPLLLKERVDWSNLMLNERMISGIMGQSFNMSARNTISSKVTNATLFL

LGLVLSTLYAAHLKTLLTKRPTSQQISNFKQLRDSPVTVFFEEAERFYLKHAWDRPIRYI

KDQLNFRETIEYNALRMGLNRSNAFSALTSEWMIVAKRQELFKQPIFTVQPELRVIQTSV

LLSLVMQSNSIYEDHINDLIHRVQSAGIVEYWKHQTLREMITMGMISQKDPFPYVAFREF

KVGDLFWIWLLWVSFLFMSFVIFLCELLVDCFISKTLIRNKRPH

>DmelIR67b

MELLYLNTLQSLSLLEGNRLVQTVQELNNIYQTELNVFLEFGNGADILESAQGTFVPTLW

IKNPQNQKVMKGNFTSCTLTILYLEDEHLDRGLYYLANWLWEYHHLEVLIFFNGGSYDKL

IQIFSRCFNEGFVNVLVMLPGSDELYTFMPYQDLKILNLKSIKEFYSLSRKKMDLNGYNI

TSGLVIAGAPRWFSFRDRQNRLILTGYMLRMIVDFTNHFNGSVRLMNVLTVNDGLELLAN

RTIDFFPFLIRPLKSFSMSNILYLENCGLIVPTSRPLPNWVYLLRPYAFDTWIAWLIMLI

YCSLALRILSKGQISISAAFLKVLRLVMYLSGSRDMGTRPTTRRLFLFVILTTSGFILTN

LYVAQLSSNSAAGLYEKQINTWEDLDKSDSIWPLIDVDIKTMEKLIPDRTKLLKKIVPTL

EADVDTYRRNLNTSCIHSGFFDRIDFALYQQKFLRFPIFRKFPHLLYQQPLQISAAFGRP

YLQLFNWFVRKIFESGIYLKMKDDAYRHGIQSGLLNLAFRDRHLEVKSNDVEYYYLIAGL

WFGGLTLATVCFLLELLIGYAKIKVTISCKMNIM

>DmelIR67c

MFCWLIFLNIILLSDRSESWSAREVIHQFNHDQQLQLNIYLDCNDVELQIGQEVSNLFVN

STADKMKILGRFSSHSLIIACFKDSTRNRTLNGVKELLWGLQYLPILFVVDSNMDFYFQQ

ALRHGFIHVLALNFMNGSLYTYKPYPKVEVHQIKDMQKFYKLTKLRNLQGQAVRTTVETM

TPRCFRYRNRHGQLVYAGYMYRMVKEFISTYNGTEEHVFGNVDTVPYKEGLAALKNGEID

MMPRIIHALEWYYFYRSHILYNIKTYIMVPWAEPLPKSLYFIQPFRGTVWITIMVSFVYA

SIVIWWIRYRQQGNSSLTQSFMDVLQLLFQLPLSKIWHFNMGTHQVVSFIVLFVFGFMLT

NLYTAQLSSYLTTGLFKSQINTFDDLFREKRTLLVESFDAEVLHNMTKEKIIQKEFESII

LITSIEEVFKHRKSLNTSYAYEAYEDRIAFELSQQRYLRVPIFKILKEVYDQRPVFVALR

HGLPYVELFNNYLRRIFESGIWIKLQEDSFLEGIASGEISFRKSKSREIKIFDKDFYFFA

YILLGMGWCVSTIALFLELWSFKYSVTNVLHEG

>DmelIR68a

MRCLWILIVAFISLAMATSIPIPIANPAPLSGYEMQLKILLQKILWVANVKRCFAVITDD

LHYPIYDRIFFESVGRRVIPFFVMRTNESDDLQRPSRQVELFVKAIKSSDCELNVITILN

GWQVQRFLGYIYDNRSLNMQKKFVLLHDLRLFESDMIHLWSVFIDAIFLKRQLDNKYTIS

TIAFPGILSGVLVMKNIANWELGKGLNGRILFADKTSNLFGTSLPVAISEHVPMVLWANA

TKSFQGVEVEIMNALGKALNFKPVYYKPNQTENMDWTELDGGASVAYGSGNPDGYAQNGT

HIDSMLVDEVAAHSARFAIGDLHLFQVYLKLVELSAPHNFECLTFLTPESSTDNSWQTFI

LPFSAGMWVGVLLSLFVVGTVFYAISFLNAIINGNVSSEFFRCLRPNRNVPMDPKIYRRI

SFRIAISRYRSSKGDRMPRDLFDGYTNCILLTYSMLLYVALPRMPRNWPLRVLTGWYWIY

CILLVATYRASFTAILANPAARVTIDTLEDLLRSHIPPSTGATENRQFFLEANDEVARKV

GEKMEVFGYSDDLTSRIAKGQCAYYDNEFYLRYLRVADESGSALHIMKECVLYMPVVLAM

EKNSALKPRVDASIQHLAEGGLIAKWLKDAIEHLPAEALAQQEALMNIQKFWSSFVALLI

GYVISMLTLLAERWHFKHIVMKHPMYDVYNPSLYYNFKRIYPQH

>DmelIR68b

MKFLVGLLLQWYLPGIYALAEIACRIAVEQNVQVTYLYRCASCPASFDADYSALELDLYR

CVGSRLPVITRNMEAHELEPFRRTDSLSIFQIPAAEKGDSLVRRILDMLNPHQRRKHMHK

YLFVWPNAGRHQLLRLFRGSWAKKLLYGLAITGRENGTFDFDPFAWGGLQVIQRLDGEVP

YARKVKDLRGYPLRFSMFTDPLMAMPRSPVETAGYQAVDGVAARVVGEMLNASVTYVFPE

DNESYGRCLPNGNYTGVVSDIVGGHTHFAPNSRFVLDCIWPAVEVLYPYTRRNLHLVVPA

SAIQPEYLIFVRVFRRTVWYLLLVTLLVVVLVFWVMQRLQRRIPRRGVIQFQATWYEILE

MFGKTHVGEPAGRLSSFSSMRTFLMGWILFSYVLSTIYFAKLESGFVRPSYEEQVDRVDD

LVHLDVHIYAVTTMYDAVRSALTEHQYGLLENRSRQLPLGIATSYYQPVVRRRDRRAAFI

MRDFHARDFLAITYDSQAERPAYHIAREYLRSMICTYILPRGSPFLHRLESLYSGFLEHG

FFEHWRQMDLITRVGASPDAEEFLEDLGDQTDTDSGSNELAIRNKKVVLTLDILQGAFYL

WSVGIGISCLGFAVEHAHWFWRRQTLRNAVEARTS

>DmelIR75a

MQLVQLANFVLDNLVQSRIGFIVLFHCWQSDESLKFAQQFMKPIHPILVYHQFVQMRGVL

NWSHLELSYMGHTQPTLAIYVDIKCDQTQDLLEEASREQIYNQHYHWLLVGNQSKLEFYD

LFGLFNISIDADVSYVKEQIQDNNDSVAYAVHDVYNNGKIIGGQLNVTGSHEMSCDPFVC

RRTRHLSSLQKRSKYGNREQLTDVVLRVATVVTQRPLTLSDDELIRFLSQENDTHIDSLA

RFGFHLTLILRDLLHCKMKFIFSDSWSKSDVVGGSVGAVVDQTADLTATPSLATEGRLKY

LSAIIETGFFRSVCIFRTPHNAGLRGDVFLQPFSPLVWYLFGGVLSLIGVLLWITFYMEC

KRMQKRWRLDYLPSLLSTFLISFGAACIQSSSLIPRSAGGRLIYFALFLISFIMYNYYTS

VVVSSLLSSPVKSKIKTMRQLAESSLTVGLEPLPFTKSYLNYSRLPEIHLFIKRKIESQT

QNPELWLPAEQGVLRVRDNPGYVYVFETSSGYAYVERYFTAQEICDLNEVLFRPEQLFYT

HLHRNSTYKELFRLRFLRILETGVYRKQRSYWVHMKLHCVAQNFVITVGMEYVAPLLLML

ICADILVVVILLVELAWKRFFTRHLTFHP

>DmelIR75b

MNFSVLESHFKEAQIFVDADVTYVTHDPFSKNFLLYDVYNKGRQLGGELNITADREIFCN

KTNCRVERYLSELYTRSALQHRKSFTGLTMRATAVVTALPLNVSIKEIFDFMNSKYRIQL

DTYARLGYQARQPLRDMLDCKFKYIFRDRWSDGNATGGMIGDLILDKADLAIAPFIYSFD

RALFLQPITKFSVFREICMFRNPRSVSAGLSATEFLQPFSGGVWLTFALLLLLAGCLLWV

TFILERRKQWKPSLLTSCLLSFGAGCIQGAWLTPRSMGGRMAFFALMVTSYLMYNYYTSI

VVSKLLGQPIKSNIRTLQQLADSNLDVGIEPTVYTRIYVETSEEPDVRDLYRKKVLGSKR

SPDKIWIPTEAGVLSVRDQEGFVYITGVATGYEFVRKHFLAHQICELNEIPLRDASHTHT

VLAKRSPYAELIKLSELRMLETGVHFKHERSWMETKLHCYQHNHTVAVGLEYAAPLFIIL

LGAIILCMGILGLEVIWHRHCTLH

>DmelIR75c

MTSWPLYRLIVFNLLEINLSNLMVFHCWSIKEAFPLVEMLNQNGIFSQYIDVQNPDNLAN

VHKEYLDSDLVSLNADVTYVSREDEERFILHDVYNKGSHLGGKLNITVDQTLQCNRSHCQ

VKEYLSELHLRPRLQHRMDLSSVTFRLAALVSVLPINSSEEELLEFLNSDRDSHMDSISR

IGNRLIMHTQEILGFNVQDAFGGAIGMLTNESAELCTTPFVPSWNRLHYLHPMTEQAQFR

AVCMFRTPHNAGIKAAVFLEPFMPSVWFAFAGLLIFAGVLLWMIFHLERHWMQRCLDFIP

SLLSSCLISFGAACIQGSYLMPKSAGGRLAFIAVMLTSFLMYNYYTSIVVSTLLGSPVRS

NIRTIQQLADSSLDVGFDTVPFTKTYLVSSPRPDIRSLYKQKVESKRDPNSVWLSPEEGV

IRVRDQPGFVYTSEASFMYHFVEKHYLPREISDLNEIILRPESAVYGMVHLNSTYRQLLT

QLQVRMLETGITSKQSRFFSKTKLHTFSNSFVIQVGMEYAAPLFISLLVAYFLALLILIL

EICWARYAKKKFSTIIPQNQ

>DmelIR75d

MKVQVAHWLPLIFFLLVSGTPRVAGSWRSEYSRQDPDPKTRWGNQLPDMLVAYYRHHGVH

SLMLVVCHTDIADFRLWKLWQHFNLNNFYVQVSTESSLRDLQHVDALDEHKDAPPPKSFH

ANNSTHWETSFLLPALPYKMGILLLEFSSECALNLLRWSAASEHNYFTTNRFWLLLTEDP

GDIDLLEDPEIFIPPDSELRVLHYENVGNFSCSLIDLYKVAAWKPLKRTLVGHNIRNSRH

VIHALQHFGSAITYRQDLEGIVFNSAIVIAFPDLFTNIEDLSLRHIDTISKVNHRLMLEL

ANRLNMSYNTYQTVNYGWRQPNGSFDGLMGRFQRYELDLAQLAIFMRLDRIALVDFVAET

YRVRAGIMFRQPPLSAVANIFAMPFENDVWVSILMLLIITTVVLVLELFFSPHNHDMSYM

DTLNFVWGAMCQQGFYVEVRNRSARIIVFTTFVAALFLFTSFSANIVALLQSPSDAIQSL

SDLGQSPLEIGVQDTQYNKIYFTESTDPVTKNLYHKKIASKGENIYMRPLLGMEKMRTGL

FAYQVELQAGYQIVSDTFSEPEKCGLMELEPFQLPMLAIPTRKNFPYKELIRRQLRWQRE

VSLVNREERKWIPQKPKCEGGVGGFVSIGITECRYALGIFGCGAAVSFVLFLFEFIFRHF

KQVYRIIKGYREVQR

>DmelIR76a

MENLLVESYYFSTVLSFFAQQFFADSHATCIFWHPAFDFRLETVHPMPLIIMDWHRWANR

SDQDVYDYKIKEDEFEGKGIPYNDWTLRLTVAIERSHCETFIAFQEQIPEFARYFYHASI

YSIWRSLRNRFMFVYTKEFEDKKDSYLSGYIFQDQPNILVITSQYLNSSTFEIKTNRFVG

PRNFNKNPEPVEFYILQRFDAKGTKATWETQSAMSSKMRNLKGREVVIGIFDYKPFMLLD

YEKPPLYYDRFMNTTDVTIDGTDIQLMLIFCELYNCTIQVDTSEPYDWGDIYLNASGYGL

VGMILDRRNDYGVGGMYLWYEAYEYMDMTHFLGRSGVTCLVPAPNRLISWTLLLRPFQFV

LWMCVMLCLLLESLALGITRRWEHSSVAAGNSWISSLRFGCISTLKLFVNQSTNYVTSSY

ALRTVLVASYMIDIILTTVYSGGLAAILTLPTLEEAADSRQRLFDHKLIWTGTSQAWITT

IDERSADPVLLGLMEHYRVYDANLISAFSHTEQMGFVVERLQFGHLGNTELIENDALKRL

KLMVDDIYFAFTVAFVPRLWPHLNAYNDFILAWHSSGFDKFWEWKIAAEYMNAHRQNRIV

ASEKTNLDIGPVKLGIDNFIGLILLWCFGMICSLLTFLGELWRGQG

>DmelIR76b

MATGIELLVAAALCVACPPLNDSPPTNLIQMGENGTLSPVTELPMDVDASEAGFDADAPV

ETLETINRKKPKLREMLDWIGGKHLRIATLEDFPLSYTEVLENGTRVGHGVSFQIIDFLK

KKFNFTYEVVVPQDNIIGSPSDFDRSLIEMVNSSTVDLAAAFIPSLSDQRSFVYYSTTTL

DEGEWIMVMQRPRESASGSGLLAPFEFWVWILILVSLLAVGPIIYALIILRNRLTGDGQQ

TPYSLGHCAWFVYGALMKQGSTLSPIADSTRLLFATWWIFITILTSFYTANLTAFLTLSK

FTLPYNTVNDILTKNKHFVSMRGGGVEYAIRTTNESLSMLNRMIQNNYAVFSDETNDTYN

LQNYVEKNGYVFVRDRPAINIMLYRDYLYRKTVSFSDEKVHCPFAMAKEPFLKKKRTFAY

PIGSNLSQLFDPELLHLVESGIVKHLSKRNLPSAEICPQDLGGTERQLRNGDLMMTYYIM

LAGFATALAVFSTELMFRYVNSRQEANKWARHGIGRTPNGQSVAPSRWLRGWRRLNSGHG

QLLGASTHGQNVTPPPPYQSIFNGGSHGDPLNRWRRPLANGNALGNGVLLGGDSEGGVRR

LINGRDYMVFRNPNGQSQLVPVRSPSAALFQYSYTE

>DmelIR7a

MFHHLWLLMGLRSLAMGALHPPQPEAMTPLVAAALEILAEQVSPSQSTLAVMDLTQDAEH

RDERQEQLMTIILRSVGSEMALRTFQKPPAEVPASFVVFLVNSAQAFNTLGFHFTDIHST

REFNFLILLTHRMSSRAERLQVLRDISRTCVRFHTSNVILLTEKRDGVVLVYAYRLLNMD

CDLSVNLELIDIYKNGLFRHGHEARSFNRVLSLSGCPLQVSWYPLPPFVSFIGNSSDPEE

RAQIWRLTGIDGELIKLLASIFDFRILLEEPCNKCLSPDIKDDCSGCFDQVIISNSSILI

GAMSGSHQHRSHFSFTSSYHQSSLVFIMHMSSQFGAVAQLAVPFTVIVWLALVVSSLLLV

LVLWMRNRLVCGRSDLASHALQVLTTLMGNPLEARSLPRSSRLRILYAGWLLLVLVLRVV

YQGKLFDSFRLPYHKPLPTEISELIRSNYTLINQEYLDYYPRELTVLTRNGSKDRFDYIQ

GLGKEGKFTTTSLIATMEYYNMMHWSTSRLTHIKEHIFLYQMVIYLRRHSLLKFAFDRKI

KQLLSAGIIGYFVREFDACQYRKPFEEDYEVTPIPLDSFCGLYYISLIWLSAAVVAFILE

LLSQRIVWLRRIFE

>DmelIR7b

MKYWLYILSCCSLVASTMESSSDWDLAEALAQVVANSEMGRFKTLYIYTHTNSQSTGGHL

EELLDQVLMIVPNNLQARRLLLQQSMEYKPYVHAVLALVDGLPSLSAIYARIRATQDLSH

TLIYMSMPTDAYGEEMQATLRFLWRLSVLNVGVVLRPPGDHILMVSYFPFSALHGCQVIS

ANVVNRYQVGTKRWASQDYFPSKLGNFYGCLLTCATWEDMPYLVWRPDGSGSFVGIEGAL

LQFMAENLNFTVGLYWMNKEEVLATFDESGRIFDEIFGHHADFSLGGFHFKPSAGSEIPY

SQSTYYFMSHIMLVTNLQSAYSAYEKLSFPFTPLLWRAIGLVLILACLLLMLLVRWRHHH

ELPRNPYYELLVLTMGGNLEDRWVPQRFPSRLVLLTWLFATLVLRSGYQSGMYQLLRQDT

QRNPPQTISEVLAQHFTIQLAEVNEARILASLPELRPEQLVYLEGSELQSFPALAQQSGS

SARVAILTPYEYFGYFRKVHPMSRRLHLVRERIYTQQLAFYVRRHSHLVGVLNKQIQHAH

THGFLEHWTRQYVSAVDEKDESVARIASTSYSTLDGIDGDPSLSESEEDQQVAPVRQNVL

SMRELAALFWLILWANLGAVVVFVLELLLPRIKLRKILRKMKKSTRASATTTSTLSSPST

TKDIPFSCKDGFQDSWPKCSLLVS

>DmelIR7c

MLHSAVHNVSLVYALVWAIDNYYGMATSTPLAVVQFPTSRESRRLHNDLIDAALGRSSGT

GRIQFLLEDDRVEMTETDTDPPPPSGLTGRPIAIWFLDSLRSYFRLEMYLNQLGSPYKRN

GFFLVIYTGLEDQPMESLKIMFRRLLNMYVLNVNVFLQRDGTVHLYTYYPYGPHHCQSSL

PVYYTAFQDLAAPANGFGLTKPLFPRKLTNMHGCEMVVATFEHRPYVIIEDDPKTPGGRS

IHGIEGLIFRSLAERMNFTIKLVEQKDKNRGEILPDGNFTGILKMMVDGEVNLTFVCFMY

SKARSDLMLPSTSYTSFPIVLVVPSGGSISPMGRLTRPFRYIIWSCILVSLIFGFVLICL

LKITALPGLRNLVLGRRNRLPFMGMWASLLGGLALYNPQRNFARYILVMWLLQTLILRAA

YTGQLYLLLQDVEMRSPIKSLSEVLAKDYEFRILPALRTIFKDSMPTTNFHAVLSLEESL

YRLRDEDDPGITVALLQPTVNQFDFRSGPNKRHLTVLPDPLMTAPLTFYMRPHSYFKRRI

DRLIMAMMSSGIVARYRKMYMDRIKRVSKRRNLEPKPLSIWRLSGIFVCCAGLYLVALIV

FILEILTTNHRRLRRAFNVINRYAA

>DmelIR7d

MDIRCVVALLLGLCKVQAVVWPHQHLLEEQLASQISATLQKIFINGLAVYNFGVFISTSY

EEMDRDRVILVHQVLNRNLYPPNFPVAVVLASKMNRKITAQVFTQLLFVQNAEQAIAIAE

GVNRNGLCVIVLLTSQPERPIMTKIFTYFMQERYNINVVILVPRLHGVQAFNVRPYTPTS

CSSLEPVEIDIKDGDLWDVFPRRLKNLHGCPLSVIVWDIPPYMRINWKSSDPMDGLDGLD

GLLLRIVARKMNFTLKLIPNEPNGLIGGSSFMNGTFTGAYKMLRERRANITIGCAACTPE

RSTFLEATSPYSQMSYIIVLQARGGYSIYEVMLFPFEKYTWLLLSTILGLHWIVGSRWRM

PSPILAGWMLWIFVIRASYEASVFNFIQNSPVKPSPRTLDQALSGGFRFITDHASYRMTL

KIPSFQGKTLISAGQPVDVFDALLKAPWKTGAFTSRAFLADHLVRHRKHRNQLVILAEKI

VDNMLCMYFPHGSYFAWEINKLLFNMRSFGIFQHHSQILAWDNLPTTTDTDTPGKRIHSS

TESVATGFAESMSFVVAALNCLMGALCISIVVFGLELLSRRRHWTGLEWLFERV

>DmelIR7e

MNHINEFVARAVLHVVHHYILSVTPSLVLTLCCRSNHTCNFYNKMMSTLFREWGLAPLQI

VNVLRGVPWHPVPGRRHFNVIFTDSFAAFEEIRMEYYSREYNYNEHYFIFLQARDRLLQG

EMRLIFDYCWRYRLIHCSIQVQKSNGDILFYSYYPFGEHGCSDMEPQLINRYNGSMLVEP

DLFPRKLRNFFGCPLRCALWDVPPFLTLDEDQEEVLRVNGGYEGRLLLALAEKMNFTIAV

RKVHVNMRDEALEMLRRDEVDLTLGGIRQTVARGMVATSSHNYHQTREVFGVLASSYELS

SFDILFYPYRLQIWMGILGVVALSALIQLIVGRMLRERMGSRFWLNLELVFVGMPLLECP

RSHTARLYCVMLMMYTLIIRTIYQGLLYHLIRTHQLNRWPQTIESLVQKNFTVVLTPIVQ

EVLDEIPSVQHMRFRLLEANSELDPLYFLEANHQLRQHVTASALDIFIHFNRLSADKVHQ

RGEQGSGAHFEIVPEDIISMQLTMYLAKHSFLIDQLNEEIMWMRSVGLLSVWSRWELSES

YLRNEQSFQVLGTMELYAIFLMVLVGLIVGLLVFILELVSMRSIYLRKLFT

>DmelIR7f

MQGEDANLYVARALRLVIENVLAQLSTTLVVTISTRHLGTAHWFEYMMNILMDSWRMVAV

QLLRIRPDLVVNPVPGRKRVSLLMVDSYQGLLDTNITASNANFDDPDYYFIFLQARDHLI

PKELQLILDHCLAHFWLHCNVMIQTAQVEVLVYTYYPYTADACQKAYPIPVNTFDGRKWK

ASQMFPDKLSQMHGCPLTVLTWHQPPFVELVWDPKHNRSRGSGFEIQLVEHLARRMNFSL

ELVNIALLRPNAYRLAEGSSEGPIEKLLQRNVNISMGYFRKTARRNQLLTTPMSYYSANL

VAVLQLERYRIGSLALLVFPFELSVWMLLLLALLIHLGIHLPSARRGNEEDGGGGLQVVA

LLLGAALARLPRSWRHRFIAAHWLWASIPLRISYQSLLFHLIRLQLYNTPSFSLDQLLAE

GFQGICTANTQRLLLEMPQLARDPDSIQSVDTPFDWDVLNVLTRNRNRKIFAVANQDVTL

SFLHSSAHPNAFHVVKQPVNVEYAGMYMPKHSFLYEKMDDDIRRLDASGFIHAWRRASFA

SVHRKEQVHMTSRRYINHAKLSGIYMVMAGLYLLAGLLFAGEVLLRQRN

>DmelIR7g

MNVTSLLNFESMKYIGAQTQAASINHHVAQALRVFIEDFYQRIAPAFIVVLSCRRPSPMN

FYRNIMQLLYESVDTMIVQLVLVELGRPRRIAGPRTHNLLLVDSLDALLDIEIHTYTAQS

DTSEYYFIFLQQRDALIPHDMQGVFAYCWRHQLINCNVMTQSSGGQVLLHTYFPYAPGQC

NDSQPTRINMFLGESWKHRDYFPSKLHNLNGCPLIVLARKVSPFLDLDEGQRELRGLEGR

LLQELSRRMNFSIQFSGLQDQLKNRTTWTEKQLLQKLVQERIAHLAIGYVRKRIQYATNL

TPVFPHYSNRVVGCLLLNAHNLTSLEIWSFPFQALTWICLVAGDRLALVLAVYAASLGLP

IDPPERPSLQLLFASWLIFGLIVRSMYSALLFFILRYHLHQRLPGNLQDLTHGDYAAVMG

RTTLQDLREVPSLQDLLGLKSVIVTSEREEEVLRTLDRCTLREGAGSHPLFFGLISQDAL

LHLTQRGHRAGAYHIIPQDVLEQQLAIYLQKHSHLASHLDHLVMSIRSVGLVHHWAGQMA

SERYFRSRFLYREKRIRQPDLWAVYILTAGLYLLSLVVFICELLASRRAGL

>DmelIR84a

MIKLQVKVISWPLIILTAFLRVLQIESINTNFLELAAFEDFLRSEHLSHVLVVRGDDADG

DWKIECHQKLLANYRVQFYRPEMSANFEDLMFYGSPRTAVLVLNSEHVLVRRQVFGVASE

AGYFNNSLAWFILGSGRESLPVEQLIDQLLSGYRMGIDADITVALRGPDNASMLFYDVYR

ISRQANTPLIIEKKGLWTHSGGYQKFGNFKNTWVIRRRNFLNVTLIGSTVLTEKPPGFGD

MEYLADDKQLQQLDPMQRKTYQLFQLVERMFNLSLAISLTDKWGELLDNGSWSGVMGQVT

SREADFAVCPIRFVLDRQPYVQYSAVLHTQNIHFLFRHPRRSHIKNIFFEPLSNQVWWCV

LALVTGSTILLLFHVRLERMLSNMENRFSFVWFTMLETYLQQGPANEIFRLFSTRLLISL

SCIFSFMLMQFYGAFIVGSLLSESARSIVNLQALYDSNLAIGMENISYNFPIFTNTSNQL

VRDVYVKKICKSGEHNIMSLQQGAERIIQGRFAFHTAIDRMYRLLLELQMDEAEFCDLQE

VMFNLPYDSGSVMPKGSPWREHLAHALLHFRATGLLQYNDKKWMVRRPDCSLFKTSQAEV

DLEHFAPALFALALAMVASALVFLLELFLHWLPDFRRRLGTMST

>DmelIR85a

MSIQWLKHILLLAILVNLAGTRENHIPLDLKKSSIVMVKMSQILCKARIKVLFVYFENQT

SHEHTGQILKEVTKCDISNQNTPLEAVKDDGILMYMVMITTNISQPLELSLIRKKSAAKH

RSHVFLLVRDADTVSDAWMRASFRQFWKIWLLNIVILYWRDGRLNAYRYNPFMDNYLIPV

DNKPNEVPTLEQLFPKTIPNMQRKPLRMCIYKDDVRAIFWRQGTILGTDGLLAAYVAERL

NATMMITRPHSYNNHNLSSDICFLEVAKEYVDVAMNIRFLVPDTFRKQAESTVSHTRDDL

CVIVPKAKTAPTFWNIFRSFGSLVWALILVSVLVANVFCYILKSEVGRVPMQLFAGALTM

PMTQIPPNHSIRLFLIFWLYFGLLICSAFKGNLTSMMVFQPYLPDINQLGALARSHYHII

IRPRHVKHIQHFLTLGHKHESRIREQMLEVSDTQMYEMMRNNDIRFAYLEKYHIARFQVN

SRVHMHLGRPLFHLMNSCLVPFHAVYIVPYGSPYLGFLDSLIRSSHEFGFERYWDRIMNS

AFIKSGVKVVNRRRGSGNDEPVVLKLQHFHAVFALWLVGIGMACIVLAWEHLTHNYNLAV

TKRRD

>DmelIR87a

MSTPEQRFWLAALLFLLSQHSEVRGFGINLMKVQTEDKGQEACILALLRKYFDSGDGLSG

SVLCINRNYQLPNIEEQLLRGVNNYENYPWSLLITNSREGPSPAKFLMNEKPQCYFLIVD

NLEDEDLDEVFEHWKGMVNWNPLAQFVVYLASLEETDEEMNDLMVELLLTFINKKIFNVN

VIGQSEENQFYYGKTVFPYHPDNNCGNRVISVELLDACDYPSEETDSEDENDEDEGDGAQ

EEDDGPQEEGDGEQEEEDGPQEQEDGDQAKGDEGQENDDGGLENKVENEFRIGASDDDEL

ENDLSSNSSEPEAIIEEFFRAKFEDKFPRDLSGCPLTASFRPWEPYIFRNSEEQPVDDYY

YGLQGDEDDYNDTSPNYGESDDESYADPGEDGDGAIPDTETQSGGKLKLSGIEYEMVQTI

AERLHVSIEMQGENSNLYHLFQQLIDGEIEMIVGGIDEDPSISQFVSSSIPYHQDELTWC

VARAKRRHGFFNFVATFNADAGFLIGIFVVTCSLVVWLAQRVSGFQLRNLNGYFPTCLRV

LGILLNQAIPAQDFPITLRQLFALSFLMGFFFSNTYQSFLISTLTTPRSSYQIHTLQEIY

SNKMTVMGTSEHVRHLNKDGEIFKYIREKFQMCYNLVDCLNDAAQNEHIAVAVSRQHSFY

NPRIQRDRLYCFDRRESLYVYLVTMLLPKKYHLLHQINPVIQHIIESGHMQKWARDLDMR

RMIHEEITRVREDPFKALTFDQFRGAIAFSGGLLLVASCVFAFELCYVKYVYRTEKRERK

TKKITKKVHNIKIQHD

>DmelIR8a

MELPLLVLLLALRFAGSEVLKITFWIEPVQRAEFDTDIAMVLKELDALRLDVKVDDTTLT

LTRSEDGLDMQRFCEILSTVGASAVIDLTYSHWEEGYNLVRSLGIGYVRLERIMRPFLDM

FGDFMRQKRANNVAMVFMNARDAVEAMQQMLVGYPFRTLIMDASQTDPGQHFLERIRSLR

PAPTYIALFARAAAMNGIFEKVQKADLFQRPLEWHFVFLDTRDRVFKYRRQAELCTRFTL

NPRAICRSMPMPDLYCGSGFTMQRAMLLNVLRSLINAAQVSPGYPLAIYQDCNATASSSE

VSDPLEKDDYNWLDMVHWSNFLAYAPPLPHIQDQFQSPVPGLTFAVNISAGYYSSEHEAK

TDLAAWSSVGEMRLLNETISPARRFFRIGTAESIPWSYLRREEGTGELIRDRSGLPIWEG

YCIDFIIRLSQKLNFEFEIVAPEVGHMGELNELGEWDGVVGDLVRGETDFAIAALKMYSE

REEVIDFLPPYYEQTGISIAIRKPVRRTSLFKFMTVLRLEVWLSIVAALVGTAIMIWFMD

KYSPYSSRNNRQAYPYACREFTLRESFWFALTSFTPQGGGEAPKAISGRMLVAAYWLFVV

LMLATFTANLAAFLTVERMQTPVQSLEQLARQSRINYTVVKDSDTHQYFVNMKFAEDTLY

RMWKELALNASKDFKKFRIWDYPIKEQYGHILLAINSSQPVADAKEGFANVDAHENADYA

FIHDSAEIKYEITRNCNLTEVGEVFAEQPYAVAVQQGSHLGDELSYAILELQKDRFFEEL

KAKYWNQSNLPNCPLSEDQEGITLESLGGVFIATLFGLVLAMMTLGMEVLYYKKKQNALE

ITQVRPVNDSSGSGGNSSTAPPTATSTTKQAWHIPVLEAEEKPAKVSPPPSFETATFRGK

KLPARITLGDGKFKPRHGLYARRNLGASDSHSGYME

>DmelIR92a

MLLQPLVMHLSQLLRIIVGQYFAEFPSILIVYNNSASTTPLQLEYLSALELVLRELSKPI

RLQWINVAFLKDLNDLEDQVMGALNSSVTEGFITILSQTHHFIHARYYATRNANVRLKDK

RYLFLCEDESPAELLCMDILQFYPHHLMVRPGTETAPTGPTGPHPDPRRGGGASVSTKNK

DDGEGGAGNKTTSPYRDINFELWTQKFVGAVGNLDALLLDAFLPNETFANRVELYPNKLL

NLQRRSLLVGSITYVPYTITNYVPAGQGDVDPIHPQWPNRSLTFDGAEANVMKTFCQVHN

CHLRVEAYGADNWGGIYDNESSDGMLGDIYEQRVEMAIGCIYNWYDGITETSHTIARSSV

TILGPAPAPLPSWRTNIMPFNNRAWLVLISTLVICGTFLYFMKYVSYRLRYSGTQVKFHH

SRKLEKSMLDIFALFIQQPSAPLSFDRFAPRFFLATILCATITLENIYSGQLKSMLTFPF

YSAPVDTIEKWAQSGWKWSAPSIIWVHTVQSSDLETEQILARNFEVHDYSYLSNVSFMPN

YGFGIERLSSGSLSVGDYVSTEALENRIVLHDDLYFDYTRAVSIRGWILMPELNKHIRTC

QETGLYFHWELEFIDKYMDKKKQEVLMDLANGHKVKGAPQALDVRNIAGALFVLAFGVAF

AGCALVAELLIHRMDLSK

>DmelIR93a

MNPGEMRPSACLLLLAGLQLSILVPTEANDFSSFLSANASLAVVVDHEYMTVHGENILAH

FEKILSDVIRENLRNGGINVKYFSWNAVRLKKDFLAAITVTDCENTWNFYKNTQETSILL

IAITDSDCPRLPLNRALMTVECRINAVVFVDQTILEENALLVKSIVHESITNHITPISLI

LYEINDSLRGQQKRVALRQALSQFAPKKHEEMRQQFLVISAFHEDIIEIAETLNMFHVGN

QWMIFVLDMVARDFDAGTVTINLDEGANIAFALNETDPNCQDSLNCTISEISLALVNAIS

KITVEEESIYGEISDEEWEAIRFTKQEKQAEILEYMKEFLKTNAKCSSCARWRVETAITW

GKSQENRKFRSTPQRDAKNRNFEFINIGYWTPVLGFVCQELAFPHIEHHFRNITMDILTV

HNPPWQILTKNSNGVIVEHKGIVMEIVKELSRALNFSYYLHEASAWKEEDSLSTSAGGNE

SDELVGSMTFRIPYRVVEMVQGNQFFIAAVAATVEDPDQKPFNYTQPISVQKYSFITRKP

DEVSRIYLFTAPFTVETWFCLMGIILLTAPTLYAINRLAPLKEMRIVGLSTVKSCFWYIF

GALLQQGGMYLPTADSGRLVVGFWWIVVIVLVTTYCGNLVAFLTFPKFQPGVDYLNQLED

HKDIVQYGLRNGTFFERYVQSTTREDFKHYLERAKIYGSAQEEDIEAVKRGERINIDWRI

NLQLIVQRHFEREKECHFALGRESFVDEQIAMIVPAQSAYLHLVNRHIKSMFRMGFIERW

HQMNLPSAGKCNGKSAQRQVTNHKVNMDDMQGCFLVLLLGFTLALLIVCGEFWYRRFRAS

RKRRQFTN

>DmelIR94a

MALPKQLKFINIFLVLLIIYGSSDGTENQHEIFLNRLLQAVHNERSVETLFLLHHSNLAN

CSLQDWNPPRIPTIRSNELTVFNVEKTFNHNALALVCLMKNSYREILNTLAKSFDCMRQE

RIILMIHRKSDSKFIEDITHEVKNLQFLHLIVLIVQEKYNGQVFASTLRLQSFPEPHFKR

IRNVFAIQRIFYRPINFHGKVLNAIPNDIPILFVALNEMFTEYARRYNSTLRIQNRTIKE

DIEITEDNYDIDMKIQLHNSQNFLHHMNIAMDIGSNSLIILVPCATELRGLDIFKELGVR

TLTWLALLFYIIFVLVEMLFVFISNRFNGRNFTMRYTNPLINLRAVRAILGQTSPISNRY

SLSIQHFFVFMSLFGTLFGGFFDCKLRSFLTKRPYYSQIENFSELRKSGVTVVVDHTTRQ

FIEQEINANFFRDEVPNVRTTTIQELINHVYSYDRKFAFVANSIPWRTFREEMKSINQKI

LCDSKNLTILENVPLTFSIRRNAIFSHHLRNFIINAADSGMITCWFKMAGKVIRKHIKTT

LRESEQQPSHLPLSFDHFKWLWAVLCIAYVMSFMVFVMEILWSKYQRRTRSVSIV

>DmelIR94b

MSLIFNLLFILILSQAVSQETEFLQLKYLNNIVRSMIKLHKMETLVIVKHHLDNNCSLQN

WNAHGMGIIRTNDQGKLIMKDTFNSRTLAIICIGQNSHITLLRNVFETFGKVQQKKIILW

TQMELKEKFFQEISKKSRDLKLLNLLVLKAVTKDKLLIYRLNPFPSPHFKRIENIWTPND

TLFMDTKFNFHGMTAVVKHDYNWTIQMGNIRKFPISRIEDKEVIEFALKYNLTLQFFNDV

ERFDIELRKRIILKSNSTQPIDSGIPMVFSSLLIVVPCGNYLSIQDVIKVSGIEKWIFYI

ILVYVIFVLIEITFLGVTILISRQSRHQMIPNTLVNLCAFRAILGLPFPETRRTSLSLRQ

LFLAIALFGMIFSIFINCKLSSMLTNPCPRPQVNNFEELKTSGLTVVMDHDAENFIEKEI

GVDFFNQYMPRKVTLTFTERAKLLFSLKGNHAFTLFSESFAIIESYQRSKGLRAHCTSED

LIVAERVPRIYILENNSILDRPLRRFIRQMQESGITNHWLKNIPSSLEKNLMQITIPYDR

ERVHPLSIEHLTWLWCILILGYSISMIVFFVEMSLKRRKKNLENRAPNICIC

>DmelIR94c

MSKVFKLLVLPLIYLSLTKGSKNPQLKFLRELINVIEEGREIRTIMVIKHSRDEYCHLDQ

WNPRGSPILRTNEMGSIRISGYFNDQAVILACMGENSDYGLLKSLANAMDNMRQERIILW

SEREPTKMLMDYISQQADRYNFAQIIIVTMNEDVDAVPSLHQLNPYPTPRFRQITNISNI

RRTSFFGCGLSFQGKTAILKESVVSNIRFKVWSPSGPIPLSELKDYEIVQFAVKYNLSLK

LYDQNESKSDHFDIQLGPLFITKDFPTQMAFVSPNTACSLIVIVPCSPKWRFMDVLHKLG

VLKLIGCLLIAYAVFVLIETLILWLTHRISGREVRLTSLNQLLNPRAFRGILGLPFPEFR

RSSISLRQLFLVISVFGLVYSNFVSCTLSALLTKPAQNPQVRNFKELRDSGLITIMDKYT

HSFIEKHIDPEFFDHVLPHYLILQKKEALRMIWNFNDSYSYVMYTTTWKSLNTVQKSFDE

RVFCESESLTIAWNLPRMYVLGNNSVLKWMLSRYITYMPQTGIPDSWTEQLPKVLKLLYN

VTSPRRIKEGAVPLSIQHLSWIWHLLFIGESIATLVFIVEILLQKSNQHTSNMRERSSED

DDFV

>DmelIR94d

MGQLHLLLVALVLLSPGGDSFYHSLIHHLNRELKIEYVLLLGNFDTTWLDILWQLPVSVL

QIKEHSRETYSLLENPSHNVLTIAFVNDSPEDILEILYRNLRMLNTQPVLLVIRKSTIRV

NSLLEWCWHHQLLKVVAIAQDFMESLIVYSYNPFPVLQFIERRLDNSTVIFEKRLENLHG

YEVPIALGGSSPRLIVYRDLEGKLIFSGPVGNFMKSFEQRYNCRLVQPYPFDESAISPAR

DLIASVQNGSVQIALGAIYPQVPYTGYSYPIELMSWCLMMPVPEEVPHSQLYSMVFSPMA

FGITIVAMVLISLTLSMALRLHGYRVSFSEYFLHDSCLRGVLSQSFYEVLRAPALIKAMY

LVICLLGLLITSWYNSYFSTFVTSAPRFPQLTSYESIRHSNIKIVIWKPEYEMLLFFSEN

MEKYSSIFQLQEDYKEFLHLRDSFDTRYGYMMPMEKWSLMKEQQRVFSSPLFSLQDDLCV

FHTVPIVFPMVKNSIFKEPFDRLILDVTATGLLSRWRDMSFTEMIKAGQLGLEDRGHPKE

FRAMKVGDLIQIWRFVGWMLGLATIVFLLELICFWRHKMWQNMKYMFCRNKNI

>DmelIR94e

MDCPKWILSGLCLISLVSGATVIELLGTLKLELDFEYVLLMKNRNFSLSDQVWNGTSLTK

DVMDEVQVPVLQFNENVSYFLHNSISRRLVTLGFMSDANLDEHRGLLTALVANLRHMTTS

RVIFLVQSKASTDFLYELFRNCWRKKLLNVIVIFQDFETTSTFYSYSNFPILQIEERIYE

TSLQTLPIFPDRLRNLHGYEMPVILGGTAPRMIAYRNKKGNVVYDGTVGHFMTAFQQKYN

VKFVQPLQAKNPLDFAPSMQTVGAVRNETVEISISLTFPTIPPFGFSYPYEQMNWCVMLP

VEADVPPFEYYTRVFELAAFLLTLGTLVLISCLLASALSLHGYATNISEFLLHDSCLRGV

LGQSFVEVFRAPTLVRGIYLEICVLGILITAWYNSYFSSYVTSAPKQPPFRTYDDILASK

LKVVAWKPEYAELVGRLLEFRKYETMFLVEPDFNRYLALRDTLDTRYGYMITTNRWVLIN

EQQKVFSRPLFQKRDDFCFFNNIPFGFPLHENSVFMEPVQKLIMELAETGLYYHWITTGF

SELIDAGEMHFVDLSPHREFRAMQIQDLQYVWYGYAFMVVLSSLVWLLENLAYTVKSKTI

FPTHFMQRNKK

>DmelIR94f

MSGMWQQVLLAETSNWFRSDVLQRFWTHLRVEIRFRTMLNYRLESCDCWFDNVLGSDNST

ALLWNDQTYPHYLRRRQDTDILVVSCLRFHQYQEVLLALSLMLDQMRSMPVVLQLCGDED

SMQELNSARLLLKHSQDLKMPNVVLLSSTFFTSATLYSYEMFPEFNVQKLVYQAYLTLFP

YKLGNLKGHPIRTVPDNSEPLTIVRKTLNGSIAIDGLVWQFMIEFAKHINATLQLPIEPH

PEKSIKLVQILDLVRNQTVDIAASLRPYSLNVQRSSTHIYGSPMMVGNWCMMLPTERVIG

SHEALTRLMKSPWTWLILLLFYSVHRFLAQKTRLRSSLIHLIKLLINLSLICFLQAQLSA

YFIGPQKVNHISNMQQVEESGLKIRGMRGEFMEYPIDMRSRYASSFLLHDLFFDLAQYRN

SLNTSYGYTVTSVKWELYKEAQRHFRRPLFRYSEEICVQKLSLFSLIQQSNCIYCYRSRI

FILRMHEAGLIRLWYRRSYYVMVTAGRFPIGDLSTVHRAQPIRWTEWQNVVLLHGVGLLF

SVVVFVIELTVHYANVCLNNL

>DmelIR94g

MSTAVNSVHSKLVSLISRGQELTSIFFYAPAKEKCHLEDTISSATWGLPLVIWRTDRTVI

LNGFIGEGLLVLACLPGFHWRALLGSLARSLKYLRQARILIELMQDRDEFLVSEVLQFCL

SQDMINVNAIFDDFPETENLSSFEAYPSFEVVNQTFTPDTQVSDLYPNKMLNLRGGVIRT

MPDYSEPNTILYQDKEGNKEILGYLWDLLEAYAHKHNAQLQVVNKYADDRPLNFIELLDA

AQSGIIDVGASIQPMSMGSLSRMHEMSYPVNQASWCTMLPVERQLHVSELLTRVIPYPTL

ALLLLLWIFYEVLRGRWRRHSRLQSIGWLVLATLVSSNYVGKLLNLFTDPPSLPPVNSLA

ALMESPVRIISIRSEYSAIEFTQRTKYSAAFHLALHASILIGLRNAFNTSYGYTITSEKW

KIYEEQQKRSSKPVFRYSKDLCFYEMIPFGLVIPENSPHRAPLHSYTLLLRQAGLHDFWV

NRGFSYMVKAGKINFTAVGERYEAKTLTITDLRNVFIIYVSVLLISLILFTCELFVSWVN

YWLGF

>DmelIR94h

MLSNISFSSAPELVDLYGLVLKFLVSSETTLFYFNPTGQKCSWETLPRTILSNHPQIIWF

REETYPGLYKRHSSNLFVMACLSSTSYDGQLQLLAESLTRYRSVRVLIEVQDKEGSFLAS

QILLLCQQHSMLNVVLYFSRWTRTLNVFSYLAFPYFKLLKQRLSGSLRPKIFINQLKDLQ

GYKIRVQPDLSPPNSFSYRDRHGECQVGGFLWRIVENFSKSLKGDTQVLYPTWAKAKVSA

AEYMIQFTRNGSSDIGVTTTMITFKHEERYRDYSYPMYDISWCTMLPVEKPLSVEILFSH

VLSPGSALLLILAFILFFLIVPQLIKCLGITFRGRLIGMASRIFALVMLCSSSAQLLSLL

MSPPLHTRIKSFDDLLTSGLKIFGIRSELYFLDGGFRAKYASAFHLTENPNELYDNRNYF

NTSWAYTITSVKWNVIEAQQRHFAHPVFRYSTDLCFSSETPWGLLIAPESFYREPLQHFT

LKINQAGLITQWMTQSFHEMVRAGRMTIKDYSRTNLMKPLRIQDLRKCWVIFAVGLGTST

VVFTIELLLIYTNVFLNSL

>DmelNmdar1

MAMAEFVFCRPLFGLAIVLLVAPIDAAQRHTASDNPSTYNIGGVLSNSDSEEHFSTTIKH

LNFDQQYVPRKVTYYDKTIRMDKNPIKTVFNVCDKLIENRVYAVVVSHEQTSGDLSPAAV

SYTSGFYSIPVIGISSRDAAFSDKNIHVSFLRTVPPYYHQADVWLEMLSHFAYTKVIIIH

SSDTDGRAILGRFQTTSQTYYDDVDVRATVELIVEFEPKLESFTEHLIDMKTAQSRVYLM

YASTEDAQVIFRDAGEYNMTGEGHVWIVTEQALFSNNTPDGVLGLQLEHAHSDKGHIRDS

VYVLASAIKEMISNETIAEAPKDCGDSAVNWESGKRLFQYLKSRNITGETGQVAFDDNGD

RIYAGYDVINIREQQKKHVVGKFSYDSMRAKMRMRINDSEIIWPGKQRRKPEGIMIPTHL

RLLTIEEKPFVYVRRMGDDEFRCEPDERPCPLFNNSDATANEFCCRGYCIDLLIELSKRI

NFTYDLALSPDGQFGHYILRNNTGAMTLRKEWTGLIGELVNERADMIVAPLTINPERAEY

IEFSKPFKYQGITILEKKPSRSSTLVSFLQPFSNTLWILVMVSVHVVALVLYLLDRFSPF

GRFKLSHSDSNEEKALNLSSAVWFAWGVLLNSGIGEGTPRSFSARVLGMVWAGFAMIIVA

SYTANLAAFLVLERPKTKLSGINDARLRNTMENLTCATVKGSSVDMYFRRQVELSNMYRT

MEANNYATAEQAIQDVKKGKLMAFIWDSSRLEYEASKDCELVTAGELFGRSGYGIGLQKG

SPWTDAVTLAILEFHESGFMEKLDKQWIFHGHVQQNCELFEKTPNTLGLKNMAGVFILVG

VGIAGGVGLIIIEVIYKKHQVKKQKRLDIARHAADKWRGTIEKRKTIRASLAMQRQYNVG

LNSTHAPGTISLAVDKRRYPRLGQRLGPERAWPGDAADVLRIRRPYELGNPGQSPKVMAA

NQPGMPMPMLGKTRPQQSVLPPRYSPGYTSDVSHLVV

>DmelNmdar2

MMPSRVKLKRGTDGPTPTPTPMPTTMRKHTPIATLNTASCQHNSTTSRRKRILTPPSGPI

SLLLLTVLTLLILDTRSCQGLRLTNGGGSLSKGAAANKEQLNIGLIAPHTNFGKREYLRS

INNAVTGLTKTRGAKLTFLKDYSFEQKNIHFDMMSLTPSPTAILSTLCKEFLRVNVSAIL

YMMNNEQFGHSTASAQYFLQLAGYLGIPVISWNADNSGLERRASQSTLQLQLAPSIEHQS

AAMLSILERYKWHQFSVVTSQIAGHDDFVQAVRERVAEMQEHFKFTILNSIVVTRTSDLM

ELVNSEARVMLLYATQTEAITILRAAEEMKLTGENYVWVVSQSVIEKKDAHSQFPVGMLG

VHFDTSSAALMNEISNAIKIYSYGVEAYLTDPANRDRRLTTQSLSCEDEGRGRWDNGEIF

FKYLRNVSIEGDLNKPNIEFTADGDLRSAELKIMNLRPSANNKNLVWEEIGVWKSWETQK

LDIRDIAWPGNSHAPPQGVPEKFHLKITFLEEAPYINLSPADPVSGKCLMDRGVLCRVAA

DHEMAADIDVGQAHRNESFYQCCSGFCIDLLEKFAEELGFTYELVRVEDGKWGTLENGKW

NGLIADLVNRKTDMVLTSLMINTEREAVVDFSEPFMETGIAIVVAKRTGIISPTAFLEPF

DTASWMLVGIVAIQAATFMIFLFEWLSPSGYDMKLYLQNTNVTPYRFSLFRTYWLVWAVL

FQAAVHVDSPRGFTSRFMTNVWALFAVVFLAIYTANLAAFMITREEFHEFSGLNDSRLVH

PFSHKPSFKFGTIPYSHTDSTIHKYFNVMHNYMRQYNKTSVADGVAAVLNGNLDSFIYDG

TVLDYLVAQDEDCRLMTVGSWYAMTGYGLAFSRNSKYVQMFNKRLLEFRANGDLERLRRY

WMTGTCRPGKQEHKSSDPLALEQFLSAFLLLMAGILLAALLLLLEHVYFKYIRKRLAKKD

GGHCCALISLSMGKSLTFRGAVFEATEILKKHRCNDPICDTHLWKVKHELDMSRLRVRQL

EKVMDKHGIKAPQLRLASSSDLLNHHHLKERPPLLGNLSLAASAQDLYRWSYKTEIAEME

TVL
