# Supplementary material for: A subset of chemosensory genes differs between two populations of a specialized leaf beetle after host plant shift
Source: Ecol Evol. 2018 Jul 20;8(16):8055–75. doi: 10.1002/ece3.4246 (PMC6145003; doi:10.1002/ece3.4246)
Supplement: Supplementary file 13 [file ECE3-8-8055-s013.docx]

**Chrysomela lapponica CSPs**

>ClapCSP04

MRVCTIHLVCLLGLVLAKPEEKKYTTKYDNVDIDTILKSERLLKNYVHCLLEKGNCTPDG

AELKAHLPDALLTDCSKCSAVQKKGSKKIIRYLIDNKPDWYKELETKYDPEGTYKAKYEK

EIKDQASE

>ClapCSP07

MKRLLCASILIVVLADVSVDGREERSKVSGYTTKYDNVDLNAILKNERILRNYVDCCLGK

KKCTSDGEVLKGHIKDAIENECDKCSEVQKKSVRKVGKAIYQKYPQWWKELCDHFDPQGQ

YQKRYEKFIKDALAAED

>ClapCSP06

MYNFSLFIFVFLIAFAIGEKYTTKYDNIDLDEILKSERLLKNYIDCLMDRGNCTPDGKEL

KNTLPDAMKTECSKCSDKQKEGSRKVIRFLLKNKRANFDELATKYDPDGSYIKKYKDELA

KEGIMV

>ClapCSP12

MIRPYHLLCVLVVVSLARSQKYQSKYDNIDVDSILTNRRVLKSYLRCILDEGPCPPPARE

FRTHIPDAISNNCARCTDAQVNIIRKTSRFIMRNNPDDWERISKKFDPQRKYRANFIKFL

NAQ

>ClapCSP11

MLPLLPLFVCGLSGLVAAGPLQYYATKYDHIDVETILNNRRMVNYYAACLLNKGPCPPEG

VEFKRILPEALRTNCLRCTEKQKTVTLRTIKRLKKEYPKIWDQLRNEWDPDDTYVTKFEN

TFVNSYVGPTPVVIIDRFGGENGDIISDYAVGSVSVSTPTTTIKQISYTSTTKPSTISKT

STIKKITPTTQKTTKKPITSSESTQTISNKPIATSTISYSYISPYAVPYRPLTNFGAGIQ

ATVSLGTNIVDEFVRSLGAIGNRVVETGAEIAEVVLKNIARPL

>ClapCSP01

MTFAVNTGSKMNCFCFSIFSILVISSVIDGDDVDERLVKRIKRTAVDKYSTKYDNFDILS

VFVSTGLVERYGDCLMERGSCSPEGKFLKQVIPDAIATKCSKCNAKQKKLAGVMLQQLLL

NYRPLFIELCDKYDPTGEARKMYRIDSNSKEADYENYDEA

>ClapCSP10

MLFLIILSCLVIVSLSAVTEKTKYTTKYDNINVEDIVKNDRLLKNYVDCLLDRGKCTPDG

VELKKNMPDAIETDCSKCSEKQKEGSEIMIRYLIDNKPAYWNPLQEKYDPSGSYKKRYLE

AKKTEVNIEPIVKS

>ClapCSP09

MKSVGALTFLAIVALVAGQNSYTGKYDNVDVDKILKNDRVLSNYIKCLLEQGPCTPEGRE

LKKTLPDALANGCEKCNPKQKDTSEKVIKHLVAKKAREWERLVKKYDPQGLYKIRYEQHL

ARLNPSAN

>ClapCSP02

MKTIVLLALLGMCAAKYTTMYDNIDVEQILKTQRLLKRYVDCLKGVPNTCTREGLKLREL

LPNALLNKCEECSEEQKKSAKRVSNYVIECYPKWWSELEKIYDPRGIYRANYQNELKAQG

KMICYH

>ClapCSP05

MKCVIVLCFVMVCASVMARPEDSKYTSKYDNVDLDAIVKNDRLLRNYVDCLLGKKKCSKD

GEQLKGILPDALKTKCSKCSDVQKNGAKKMINHLIKNKRTWWDELEAVYDSDGTYRKEYE

AEAKKEGIQL

>ClapCSP03

MQIKSVDRVIMSSPGLFFLMLLGTILCTEGQVLRGNSYMEKQLLCALDRAPCDILGSQIK

DALPEIIGNNCRSCDNRQVSNARRIAVFVQSKYPDVWNALVQKYSPSSTXLLMYTSNSSS

XTRIINDTX

>ClapCSP08

MHLQNSRALVVVAIFVLSIISIAKARPATSTERPTVSDDALDNTLKDKRYLMRQLKCALG

EAPCDPVGRRLKSLAPLVLQGSCSQCSPQEQRQIRKVLGYMQVNFPKEWNKILKQYSGXE

QHQYVTEYGSTTTARRTEITRISQQLPNRINTVILV

**Chrysomela lapponica Glus**

>ClapGlu02

VTCGAVFCACFLMFFAKTRDFLLGVLVISIYFGYHQAEIYRIGVISDLPNSHQHKALFKS

VSKAVRDINENQETIFLDVINIEIPKDNSFEAVRTTCSLLEQSVVGIFGPFTEDNSNVVQ

SICDLKEIPHIEVRWDDHPLDGTIINVHPYPDMLTRTYYDIILNWGWKDFVILYENNESL

QRVGELLKLFEPSKQRVVVRQLELTKTTEGYREILKEVRKSGATHFVLDCSIEILEEVLK

EAQQVGLMTSKHNFIITNLDLHTIDLKPFMYSETNITGMRFVNVEKLLENTDQPLEAYKM

KLKEALIHDAIKMFAEALSASDTMVRPFSIDCYNRSHKLSSGTTIINLMRSIEYPGLTGP

VKFDIKGYRSNFALDIFELMEGGQTTVGHWNSTMVPALNISRKIPPSEDDNGDIRNRTFK

VEITLTEPYGMRVESAEPLHGNEQYEGFAIDLIKELAAMRGFNYTFTVREDKANGKFDND

TSRWTGIIGDLIDGHADLAICDLTITMERAAAVDFTVPFMMLGVSILYKKPTKAPPSFFS

FADPFAFEVWKLLMASWVGVSVIIFVVGRISPAEWENPYPCIEEPEYLVNQLDFRNSLWF

VTGSIMQQGSEIELKSVGTRMVAGMWWFFTLLMVSSYTANLAAFLATENPDPHFTNLKEL

VDNAETKGIKIGAKALGATETFFRDKFIADPTSDFGKAWQIMEKSKQVIEIPDNKDGVAQ

AQKGYYAFFMEDTSIEYETQRKCDLNQVGGKLDEKGYGIAMRRNSTYRHSLNTAILVLQN

SGKIAEIKRKWWQERKGGGQCLADGEAADATPLNLSGVEGVFWVTIGGTIVAIMMAILEN

LLQIVKKSIKTKTPFTSVLMEEIKFYFRFGEMEKHMVYRENTEESPGTEVPYGFVVEKQM

SPDRHSYRSKRSKSRISRSRAGRSRSRSRRSGSRKFDKQLSANTTX

>ClapGlu01

TIRALKTSGQQNIVLDCHIQNLNEILKQLQQAGMINENFHYFITNLDTHTEDLTPFMFSD

VKMTGIRIIDPYAENIQTISTDLFAGSELAPSWKIKTEAALMIDAVQMLSKTLSDRQKLS

PIPIIADDDIFSCTESKSWEHGLSIINMLKTSTRDGLTGLISFNNEGFRSDFNLHVFELR

EGGITDIGNWNSSKGLNITATSQSQYVSDGDSLRNLSFNVLITLTEPYGMLKMTTEPQTG

NDRFEGYGIDLIQKLAEMEGFNYTFLVREDKKNGEYDDKTEKWTGMIGDLLEYKADLAIC

DFTITSDRQEAVDFTVPFMTLGVSILFKEPVNAPPSFFSFADPFGLDTWIALGVSFFVVS

FALYFMGRLCGDEWTNPYPCIEEPEHLTNQFSLANAIWFATGSLLAQGSEIGPIAISTRM

GAGIWWYFCLIMSASYTANLAAFLATQNPVELFTDLQSLYDNPHGIKYGGKDGGATLKFF

TEAEEGTILRKIGDHMKDNPANVKGNDEGVLRAESEDEKYAFFMESTSIEYATQRHCTLK

QYGGLLDEKGYGIAMRKNSTYRTRLNSALLQLQMSHVLDDLKKKWWEDRRGGGACGGPVA

SSEADPLGLMNVEGCFFVTVYGTMLALILVIIEHLCFVRKISRSTKIPFGKVFMSELRAF

LDFNNNTKPNIVQGFKDGSGVTVEECPRENGDSKDSKMESKRDSRSKSRPLNDSGSRCCS

SVHSRSRSRSISRPKSRKSKTSKSSDRKAPIPYGFVVSCSDNDKCX

>ClapGlu03

HEGFRRDFKIDILELYPQGLIKIGEWNSSLRQITDLQRPAAVNFQKNLGDNLFNKTFKVL

ISITPPYGMLKETTTQLIGNDRFEGFGIDVIKELSYILGFNYTFVLQEDGLYGNINKTSG

EWNGMIREIIDGRADLAITDLTVTSERESAVDFTMPFMNLGISILYRKPEPVPPSLFMFA

SPFSFNVWLLLGVSYFIVSLSFFVMGRLSPSEWQNPYPCAEEPSYLVNQFTIRNSFWFTM

GALMQQGSELAPIAISTRTASGVWWLFVLIMVSSYTANLAAFLTVETLVTPFNDIDELAA

QQEIKFGSKKNGATANYFRDSNLTKYKKVWSYMLHHPDLMMDDNDAAVVKVENENYAFLM

ESTTIEYVTERHCTLARVGGLLDDKGYGIAMRKYSPYRNDLSTAVLQLQEKGILTGLKIK

WWKEKRGGGKCASKSEGSEATPLDLKNVGGVFLVLYIGTILGTLGSFAEMTKCIYRRAKR

ENISFKEEFLKELRFLVQFKQNVKEVNNTPLDGVSDRDLMDSTKTLNYVKMDWKX

>ClapGlu04

RKFANLRTSTAATRSNWCHMKADLGVCDLTITYERRKAVDFTMPFMTLGISILYAKAVKE

PPELLSFSHPLSFEVWIYIATSYLIISITMFLVARMNPNDWENPHPCNPYPQELENIWNI

RNCCWLTLGSFMAQGCDLLPKGISTRMVAAMWWFFTLIMGACYTANMTAFLTMSRMGPTI

ESADDLATQTKIKYGCLEGGATSSFFKDNNFSTYHRMWVQMESAEPSVFEKNNKDGVKRV

LTGKRKYAFLMESSSIEYEMERNCELMQVGNNLDSKGYGIAMPTNAPYRKSINEAILKMQ

EMGILHKLKDKWWKEMNGGGQCTKDKHSTDATAAEMGMDNVGGVFVVLAGGVMLAFFIAI

CEFLWNIRKVAVVEHLTPKEALMKEFRFAMNIWVRKKEVRSNSSRDISLETLEAKX

>ClapGlu06

KPSRSSTLVSFLQPFSNTLWILVMVSVHVVALVLYLLDRFSPFGRFKIANTDGTEEDALN

LSSAIWFAWGVLLNSGIGEGTPRSFSARVLGMVWAGFAMIIVASYTANLAAFLVLERPKT

KLTGINDARLRNTMENLTCATVKGSAVDMYFKRQVELSNMYRTMEANNYDTAEAAINDVK

HRKLMAFIWDSSRLEFEAAQDCELVTAGELFGRSGYGIGLQKGSPWSDDVTLAILDFHES

GFMESLDNRWILQGNFQQCEQFEKTPNTLGLKNMAGVFILVGAGIVGGIGLIVIEMAYKK

HQIKKQKRMELARHAADKWRGAVEKRKSMRTSGAAQRRIKSNGVNEAVTISHVFDKFQRI

GQLYGAERTWPGDSDIRQRRMDDVGGGVQPVPRYLPAFTQDVSHLIVX

>ClapGlu07

IESVTMFLKIFSTGFIFLSIFLSATSMAVQLQIAGIFDSFQLHQSTFVYSDKLTDIEKGS

PDIELSPIVSGDIFPDDPLSALQGTCYFLRQGIVGIFGPQSSSNIDIVKSIADRKEIPQI

LTRWVDPIQMSPNTINFYPSPSKLAHAFVDIIRKLEWKSFTILYTNFGNLMRISDLIKKA

KDDGCLVYIENIDPNQSRNYRPTLREVMKYGQKNFILDSPIQELVNILSQIQQVGMLTKD

FNYILSNLDAHTKANSGDMDVFMHSEATIRGVHLVNPQGEQSTKVGNELCYLYKMTFNEE

CSVHPNHTREMDFETASIIDAVHVFKDALQSAGVTEEQIMDCNDTKSWEPGQNVVNILKS

GSYKGLTGTIEFDEDGFRSTFELTIYKLQSNLAQIGSWNTVSGLNMDIETTSVLVEGEED

FTNKTLVVMITLTEPYAMKIDSPDRLVGNKKYEGFAIDLIDEISKYIGFQFVLVVRGDNK

HGHFDSMSGKWTGMIGDIIEGKADLAISDLTITRDRVDPVEFTHPFMYTGIGILFHKPTE

FPTFFHFALPFSEQVWITLGLSYLGTSLTLFIIGRLSHSEWKSERNGRSLENQLTLCNSL

WFAAGSLFMGQNTGVRISSIPAKIVSICWSFVCFILIAMYIAYAASIDIHDQKEVLFNDV

KDLVESAENHGIRFGAVSGGSTQAFFKNSKSPIYEEISNFMEEHPEDMAMTTREGIERAA

NEEYAFFMESATMEYAIKRYCNLTTYGGLLNDKGFGIAVRKGSPLLTPLNRAILKLHTSG

DIMRIKRKWWEEKNIGETCEDSSDSVAIEKGLVNIDGLLWITIVGTLFTMFSAVVEFIVH

VRRLGKKSNQSFGSAFCQELKGIFKSRRIGGNSAESRVKSDDDRNTTTESLELIX

>ClapGlu08

QNLDAKGFGIATPLGSPLRDTINLAVLSLKENGDLTKLKNKWWYDRTECLRDKQDASRNE

LSLSNVAGIFYILIAGLFVAMAVAILEFCYKSQSEAKRAKIPLGDAMKAKARLTIGVGRD

YDNGRYYTPANQIAGSTEPEQPHSNTHTQVX

>ClapGlu09

APKAVSTRMVAGMWWFFTLIMISSYTANLAAFLTVERMDSPIETAEDLAKQTKIKYGALR

GGSTAAFFRDSNFSTYQRMWAFMESQRPPVFTSSNQEGVEQVVKGKGNYAFLMESTSIEY

VIERNCELTQVGGMLDSKGYGIAMPPNSPFRTAISGAILKLQEEGKLHILKTRWWKEKRG

GGRX

>ClapGlu10

MGIAALMEFNNKRGRTVILKQLESLPEQPGNFRPMLNNVKSLGETQFVLACSTNILENVL

KQLQQVGMMTEIYSYVITSLDFQTIDVEPFQYGGTNITGIRMINPDLTEVKRISMAVQDN

GDPHKLEIETALLMDAVQIFYETVYDLTVDRMMTVKAHPLNCKTADNWMYGNSIINLMKS

KSTLGLTGLIKFDHEGFR

>ClapGlu11

AGQTEIPYGTLESGSTMTFFRDSMIETYKKMWRFMENRKPSVFVPTYEEGIQKVLDGNYA

FLMESTMLDFVVQRDCNLTQIGGLLDSKGYGIATPMGSPWRDKISLAILEMQEKGEIQML

YDKWWKNTGETCQRNEKGKESKANSLGVDNIGGVFVVLLCGLAFAVVIA

>ClapGlu12

GIGVKMLPTVINSALMCVFLIRSCVDGLTVGILFGEYTSQEQLLINDTISRKYENGEIIF

SSNIQSVSGNDSYGVSQIMCKMISDGSGVAGIFGPEHSVSTVILEAISSEFQIPYILTSW

KPPSTMISEYTINFFPDASMFALGLADIVKSFGWKSFAILYEDDITLEKLQHLLVLQKYR

EKDTRNRIIIEKLGPGSDHRKILEKIQSSTLTNVILDCRLEYLISILHQAKSVNMLNAFN

NYFITNLDAHTLDYSELNTTANITTIKLIDDNYELLEDTMHKIGLAELIHHKKLKTDLML

FYDAFWFMNETLNELGPAVMTNPVSCNKKETSLSGRKLVHYMHNKLYSSPTLTGPISFDD

HGNRISFNLFVIDVLQKTKLATWSSQNMTLQVNRAFEKKLSAVSNIQGTKIIVSSRLGAP

YLMMRQTKEGETLTGNARFEGYSLDLVAGLAKIIGFEFEIQLSNDGKYGSWDENNGKWNG

IIGDVLEKRAHLGVADLTITHERREVVDFSLPFMSLGIGILYKKRASRETQILEFMTPLS

HSAWTYTGYLLVFMSIFFYLVMRLSPLDWKSSFNQEDTENIWNLKQCIWIVFRSLTGHSC

EIVPKGVSARMAMTVWWFFCTILACSYVANLAASRNLFSTETSVKNVEDLARQSRIKYGL

LEGGSTESFFRTSNFSTYQRMWSTMEQNPDNFVKSNSEGVEKVSGTGNAEYAFLMESTQI

EYVLETRCDLKQVGDWLDSKSYGIAMPPNAPYRTAVNGAILKMQESGQLADLKNKWWKEK

QEESICSVEEHTKMGVDELTLADIRGIFLILWMTICLTLILAILGFFWNIKKIAKNQKLP

YCEILMEELKFTTFVWTSKRRTASTGIESX

>ClapGlu13

GVSIVLFVVSRFSPYEWRLLHLTGDPRDHQPVHNTNHSGTMANDFTMMNSLWFALGAFMQ

QGGELSPRSISGRIVGACWWFFTLILISSYTANLAAFLTVERMVAPINSPEDLASQTEVE

YGTLYHGATWDFFRRSQITLYSKMWEFMNSRKHVFVKTYDEGIKKVRTSKGKFALLIESP

KNDYINEREPCDTMKVGQNLX

>ClapGlu14

KRNMKKLIQNVLSLLCFLSLQILCSSKTAVTIGVLLNDRTSQVRLPLNSVIYKKNVYDQN

VYFTTNISTVSNSDSFEASRTLCDMMDSYIGVAAVFAVDIPDIIPVLESVCTNFEIPFIM

TSWRPPVVRNPDQERALLSFYPEAERFAEGLAEIVKSLQWSSFVVVYEDEEGLIRMQDVL

KLQEYKRNTRKNNIIVKQLGPGPDYRPLLKEIRNTTEDNMILDCKTENVLPILLQAKSVN

MLNLHNRFLITSLNAHTIDFSVLNTTANITILRFHDPKTDNFKNAMHRWQLTEFENRNIQ

MQLDPKSIKTETLLFHDAILLLTDSISELYFTPGIETSAISCSGNQTTRDGYAIRNYMRI

KTPAMTLTGPLEFNKNGDRIHFNIYAVDIMDTVISTYFASNRSIMLARSGKESMDAAVLN

LQKIKVIVSSRLGPPYLMPREPTYEGEEFVGNRRYVGYSMDLIDGISKIIGFSYEFVITA

KYGNYDPEAKRWNGLVGELLEKRAHLAVCDLTITPERTEVVDFSMPFMTLGISILYKKPD

KKDISMFGFLDTFSNAVWIYTATLYLIISIVLFFISRMTPGDWENPHPCEDEPEELENIW

DIKNCLWLTLGSIMTQGCDILPKGISSRMAVAMWWFFSLIMTSSYTANLAAFLTKANLEP

EIDGAEALSKQTKIKYGVVIGGSTESFFRNSNFSVYQRMWQNMQQFKPSVFEDNNEDGVN

RVKTKNSFYAFLMESTQIEYVVETKCTLRQIGNWLDTKSYGIAMPMNSPYRTAINRAVLK

LQESGELGILKKKWWKDKRTEPKCDQNPADEGDAGKLALANVGGVFLVLGVGLSIACVVA

VLEFLWNVRNITVEEHVSYWDALKVELIFACKVWITKKRTRPMMSESSSSSDKSDRTDNR

SIINSILHSAESFLHLDNRPX

>ClapGlu05

LFHKVTHIRKDVMIKSYSIGKKIHRLGMKLSWFQVICAFISFHCIFCAKKNGRKNVIFGI

LFDGDAYQSELPLNNTIFSNNIGENNVVTLSTNIRHIPNNDIFDASEMVCDLLNAEEGLV

AVFGPSTSTASPLIESIFKNLRVPYILTSWKSTSYDGSETFLNFYPDAEKLSRGIAALIQ

SLEWNGFVIIYETEEGLVRLQEVLKLQGWKRKTAQDFIRIRQLNKGPDHKPFFKHFKNST

EANFILDCKTENILPILQQVQSLGMLDIHTNIFLTSLDAHTVDYSGLNTNANITTIRLFD

YKNDYFKNAVFRWTHNELQSYNRRKELSPHSIKTETVLFHDALLYFTDALNDMSEIPENE

RSSCYNTVSSRDGTELIKVMKNREYLEPLSGPIQFDDNGNRIDFNLHLYNNDQELLAVWS

GQNETVALARNYDETSNAAVSNLLKIKVIVSTRLGPPYLMEVVPENEGQILEGNYRYEGY

TKDLMDQIAKLIGFTYELYITHKNSYGKWDEDTKRWNGLVGDLLDKRAHLGICDLTITHE

RQQVVDFSMPFMTLGISILHKYPERSSNNLFTFLEPFATSVWIYSATLYLVVSLVLFFIA

RMTPGDWENPHPCEEYPSTLENIWGIKNCHWATLGTIMNQGCDILPKGISSRMALAMWWF

FALIITNSYIANLTAFLTKDKMELPIKSAEDLAKQSKIKYGLLEGGSTEAFFKTTNNSMY

QKMYMNMKSQRPTVFETENVDGVQRVNTTKDGLYAFFMESTQIEYEVEKKCKLKQVGSWL

DTKSYGIAMPMNAPYRSAINKAVLQLQEAGYLTTLKKKWWKDVRKGDSCEDLRNQKKDEK

EGDLDLAKTTGTFLVLGVGVSIGISIGILEFLWNVRNVSVEEHLSYWEALKAELLFAANI

WINRKKIKNSSAESSSERRDYKIDNENTIQNNMMHGADSFMNINSNSX

>ClapGlu-W05

HQLHEGGEFFERLLRISFAESEDKHSQVEMRGLTGIIKFDHQGFRSDFVLEIIELTREGL

KNIGTWNSSEGINFTRTYGEAYTQIVEIIQNKTFVVTTLLSAPYVMRKEASEKLTGNAQY

EG

>ClapGlu-W06

VLGFNYTIRLVPDGRYGSLNRATKEWDGLIRELLDQKADLAIADLTITYDREQAVDFTMP

FMNLGISILYRKPIKQPPNLFSFLSPLSLDVWIYMATAYLGVSVLLFILARFTPYEWQNP

HPCNPX

>ClapGlu-W04

HFFNRETNKMHLTVDEKSIIWPGRQNVKPEGFMIPTHLKVLTIEEKPFVYVRKLVEPNEG

CTVEEIPCPHFNTTGDLTDNLCCKGYCMDLLKELSRKINFTYSLALSPDGQFGNYVIRNH

SGSIRKEWTGLIGELVX

>ClapGlu-W01

KVGEWRPSTGVNITDREAFFDPGTMNVTLVVTTILEQPYVMLKSNADLVGNDRLEGFCID

LLKAIAAMVNFEYKILLVPDGKYGAFDFETLEWNGMVRQLMEKKADLAVGSMTINYARES

VIDFTKPFMNLGISILFKVPTDKESAFFTFIDFTKPF

>ClapGlu-W02

CDMEKPWDDGLSLYNYIDAVTGLHGLTGNLEFNEGKRANFKVDLLKLKKEEIRKVGQWSP

NEGINITDPNAFYENNSPNITLVVMTREERPYVMVKNDKNLTGNARYEGFCIDLLKWIAG

QVGFQYSIRLVPDHMYGVYDPHTKEWNGIVRELMEKRADLAVASMTINYARESVIDFTKP

FMNLGIGILFKVPTSQPTRLFSFMNPLAFEIWLYVLAAYVLVSFTLFVMARFSPYEWNNP

HPCHQESDIVENQFSVSNSFWFITGTFLRQGSGLNPKVRPVHALFDTSIMHFFSSFVF

>ClapGlu-W03

FKKAIECSCVSIQKIVYKRTKMHKEIFLLAVLSSAVQFGEQRKQINIGILLEDDQYELST

IAVSSIVRRINLFTKTDYLLRPHIFRVPKGDLLETGQFVCQLIEKGIAAIFGPESPEINE

MIQSVSSGLEIPQFQTFWNPKLASLNSSFQGIFNLHPSPRSLSKALATLVRENDWGSYTV

LYESEEAVLRLQE

**C. lapponica GRs**

>ClapGR03

MHDPIPNVISPSEGHVHVLAVSPRRRSVYLEGAHTGRRDPNRVTQVAPYRPSGPALPSRA

FLDAQGQRGFVYDSLRPIVFLMRVTGVLPVGIVDGVFQVTVQMMAYSVAVFLVVLAYIGY

IRWDKVEMFRTAEGRFEEAVIDYLFTVYLVPIVINPIAWYESRKQAKVLTTMVSFETIYR

RVAKKNLVLSLGNTPLLIAIGLPVLAISAMLVTHVTMVHFKLFQVIPYCYINTTTYLIGG

SWYIYSNLIGRLAQTVAADFQQALKHIGPSSRIADYRALWLTLARLVRDVGDACAYAVTF

LCLYLFLTITLTIYGLLSRIQDGVGVKDVGLTLTALLSGLMLFFVCDEAHYASNCVKVQF

QKKLLLVELNWMNDDAQQEINMFLRATEMNPTDMSLGGFFDVNRNLFKSLIATMVTYLVV

LLQFQISIPGDRSDANGNVTNTNVTRVNX

>ClapGR05

LAIPSIFTSINYILRVGVFLGVAPTPSVSKLHKLYVLLLLVVSIPLFLTSSYLKMSIKTG

IVALIEITSDFFLTLATSSMGLYIIFIHPSKLGQVFIHIKYFDGKMSFPFEERTKLYVLS

FVLHSSCHLLFSALEIWKYYQYDVLHISNYLIHTQRILRVFQKNVFMFLWLLLAIEMYKR

FKFLNNSLLRIVQDSCRAATSPMNVYRVEKCETKHYPIKQLRKISEMHNHLCELLDEINQ

IFGIPILFYVLFSIMYIVYNTTFAIQEVFLESEKEVQLKGSSNYFSLCYIIFAFVEVICL

SAAGDYVSGEAKRIKDICQNILVSLNIYDPSKVELMQLAQQVAYRNPQLAASSFFEIDFT

ILGFTLANITSYIIVAVQFLKX

>ClapGR07

QSMRMILAVYCTFTFVMVFDIFYWTKCSGEKSGGQYILRYMGFYLLYYIMITLELYFSHL

VYSIHIKLALINEHLVVGNTGNNSKNNISIMNSQIDNFHREMYGFMAPKDSEILFKIKIP

KARITNGNIFIAWXSILRSTCITIIFVAYSVFYSFIAAERIEILRKLHKQLNFGVKIINE

TIANGIMLIMLSCLVHLVVTPYFLVAELIKEKTNYLFIFLQIIWIITHICRLLIIVEPCQ

SCSLEGQRTNMLLCDLLSLSGADEEFQKAVKLFGIYLSQNQIKFSCSGLFVIDRSVITSI

AGSVTTY

>ClapGR04

QFFMTLFALEIIGVVRSRFMELQRILDEVSLKRVIIISQSDNQYVLGKLLKLRKLYGILY

DMQQNVNLIFRYSIFYGTLSYILIILGDLNYIVEFAHTSDQNFNIGVSFVNFLYAFYYSS

ASVSIVMSCDRMESISPKMVKTCYLYRDILEKSIISNEFIELAEYMKKLAPTFSAAGFFQ

VNQQLLSILISAIITYX

>ClapGR01

AQYYLQSDMLLWHTFAYYHILAMLNCLCTLWFINCTAKGRTAGWLAENLHKTLQSSDPAI

RLWEYRDLWVDLSHMMQQLGKAYSGMYGMYCILILLTTIVASYGCLTEILDHGLSFKEAG

LFLISFYCLTLLYIICNEAHYASQKMGPEFRERLLNVNIGELVIQXVXNIICSYSYSLLL

GVVDIRTRQEVIMFLTAIDKNPPIMNLNX

>ClapGR06

ISQCFAVFSSFIYPGTMFTRILAVVYVSLVLTEINEGSPAAVSSRQFLSGHPGFIPVYIR

AGDTPLEDINPDLADAFDSYAQRHGRLTYRRSLGDESGKPGGHEEFPDLAEGLTPEEDLK

PEEDSNNISDSQVPISGSHIQKIPRNX

>ClapGR02

GISLSFKELGLLVAAVYCMVLLYIFCDCSHQASENIAGKVQMSLMNIKLDSVDLDTTREV

RRVFHKSNENRYYXLNFLPFQIELFLKAIHLNPPTVSLQGYSDVNRKLVTSVSFKFSINF

YXMXYFRXKVITKIVLLLFQSIATMAIYLIVLLQFKISLVNVRSX

>ClapTR

TVNSIKYPREATIFAACCAIIFCFVGVIGNSVTVLALIRCPKLRVHATTAFVLSLCLSDL

IFCSINLPLTAARYIYEAWILGEALCKLFPVLFYGNVAVSVLNMVAITLNRYVLISCQHY

YNILFSKSAICIQLFCIWGIAFLLMVPPLLGMWGQLGLNPSTFSCTILTKNGKSPKNMIF

LIGFILPCVVIILAYSCIYYSVRKSRKKLRSHQPISDKRNGKREKDDSRLTKLMAIIFVC

FLTCFLPLMLVNVFDDHEIRYPTLHVLASVMAWASSVINPFIYAASNRQYRSAYSKLFRI

VKSSVVFSDSKQLSNNLRPDRNSVTYKPSVASDLX

**C. lapponica IRs**

>ClapIR75b

RMLLKILLSVMIFEVSSGEENHTEIMELVNAFIMKENVPTKITAFICWPRNLQLKFLKLF

SRSGFLTRLGKVVEDIQCSFPPEHHLIMMDLSCENTKAILNQVKRPDIVPSRWMFWGNKN

DSEMLTLLNKLYFNVDSQVYLISKLSTTYHIKSLYKLQENSDSFIENHIANWSPNLGFTS

FNIFSLPRNRTDLMGATLKLSFVITNNDTLNHLEDYRDIHIDPISKLSWIMVHHLMKMVN

ATPTPIFRATWGYKNTSTNLFSGMIGDLQTGEAELGGTASFFTVDRIDVVDFVSPSAPTY

MRFIFRAPPLSYTTNIFTLPFDSWVWYCCFALIAIILVVVYLIVLWEWRNPVFRKHIMDK

HDGNMAALKPGVSNVCLMEIGAITQQGTDSEPKSIAGRIATIFTFIALMFLYTSYSANIV

ALLQSTTESIRTLEDLLSSRISLGVEDIVYAHYYFENAREPVRKSIYEQKIAPKGQKPNF

MSVQEGIKRVQSGFFGFHAELSTSYKMVADTFQEHEKCGLKEISFVSLVQPWLPMKKKSP

FKEIFKVGMQKMYESGIQRREVNRIYTKKPVCTSKGSYFGSAGLLDCYAAFLIFGIGVGC

SFILLLVETFMRKYVEEKHQLEARKTX

>ClapIR25a

YVTHDRILLHLYLSMFLAYARLHRKYRKMGVEKIGVIFLALLFEYCDGQTTQNINVMFVN

EEGNGVAEKALDVAMTYLKKNNKLGINVDLRRVVGNRTESNAFLESLCSTYNSMLTSETS

PHLVLDTTMTGLGSETVKSFTQALALPTVSASFGQEGDLRQWRNINESETDYLIQISPPA

DIIPEIIRTIVLNQNITNAAILYDDSFVMDHKYKSLLQNVATRHIITPIKETNQIPEQLT

QLRKLDLVNYFILASLKNIKKVLDAADGLNYFNRKFAWHAITQDDGDIKCICRNATIMFA

KPLPNAAYQDRLGTMQRTYQLNTEPIIASAFYFDLILHSFIAIKEIISDGTWKNAGTNYI

TCDDYNGNNVPKRTGLDLKRYLNKENLESPTYGPLSVVSNGLSYMEFQMQLTSVGVRGGA

SDKSLNLGTWSSGFDNNLTLIDTQVMANLTADVVYRVVTVVQKPFIFRDETARAGFSGYC

VDLLEKIAEILKFDYEITEVDNFGVMDENGKWNGVVKELMEKRADIGLGSMSVMAERENV

IDFTVPYYDLVGITILMKLPETPTSLFKFLTVLENEVWLCILAAYFFTSFLMWIFDRWSP

YSYQNNREKYKDDEEKREFNLKECLWFCMTSLTPQGGGEAPKNLSGRLVAATWWLFGFII

IASYTANLAAFLTVSRLDTPIESLDDLSKQYKIQYAPLNSSSTQTYFERMANIESRFYEI

WKDMSLNDSLSEVERAKLAVWDYPVSDKYTKMWQAMKEAGLPSNMADAVDRVRASKSSSE

GFAFLGDATDIRYLELTNCDLTRVGEEFSRKPYAIAVQQGSPLKDQFNTAILQLLNRREL

ERLKEKWWNKNPEKKNCDKVEDQSDGISIQNIGGVFIVIFVGIGLACVTLAFEYWWYKYR

KNSKIIDIRETPNLHQGEKAKMNKKHINLLDPRSLDNPTHKNKSVYPRSRFX

>ClapIR8a

FHPTNANNYTFLYFSVCQAISDSGTIILDLTWCGNDDGKNLANDIGVPYVKIDVSISIFL

DILDKYLDFRNSSDVVIIFENPSYIDQALYYWINTARMRLLMSETLDVSTAKKLREVRPT

PNNFALLATTKNMQRLFEVAIRENLIELPERWNLIFLDFNYKSFDRSLMLNQPINYLTLD

TNICCQIQNITSSCECTDPFIMQMEFLRIALKVLLISSQEMTRNGFLSTSLNCNDNGSIQ

NDEVQMRFEDILKREMSSQNIMYLENSILRMKTTGFIEVGSNSTNTNRIIAKYEGGVIHL

EENTTIKPIKAFYRVGITHALPWSYKTTDPNSGASIWTGYCVDFTVKLAEMMNFDFEFVE

PKSGTFGVKIDGVWNGVVGDLASGQTDLAITAIIMTADKEEVIDFVAPYFEQSGITIVMR

KPVRKTSLFKFMTVLKLEVWLSIVAALIVTGFMVWFLDKYSPYSSRNNKNAYPYPCREFT

LKESFWFALTSFTPQGGGEAPKALSGRTLVAAYWLFVVLMLATFTANLAAFLTVERMQAP

VQSLEQLARQSRINYTVIKESETHQYFINMKYAEDTLYRMWKELTLNASTDDTRYRVWDY

PIREQYGHILLAINDSNPVANAEEGFRIVEEHLDADYAFIHDSSEIKYEISRNCNLTEVG

EVFAEKPYAVAVQQGSHIQDDISKTILKLQKDRFFEGLQAKYWNHSSKGNCPSMDDNEGI

TLESLGGVFIATLFGLALAMITLAGEVIYYRQKRKDGVFKDKQAKIPPRELYFKENRSVT

IGTTFQPIQLHNQIIEDQKELRLSHISLYPRARNRITQVDX

>ClapIR76b

AITTHIQFMTTCEVEVEKGAMGLIEIVLASLCLNATCDIQPDYGKISATARIQRMTQLAE

ELKGQTLIITTLQNGVLSGYKKENGTLIGTGLAFDILRMFQQKFGFNYKIVLPEDNIFTP

GSINKGAKNLLQDKVVDMAVAFLPIVDSFRHDITYSRSFDVAEWSVLMVRPRDSATGSGL

LAPFTTEVWIIIIFSVLVVGPIMHLLVLVRARFSETLEEGIVYPLTDCMWFLYGALLKQG

SVLNPLTDSSRIIFSTWWLFILIITAFYTANLTAFLTLSKFTLPIKSASDIGDKGYKWVS

NKGNGIRDLIYSESRDRSLIASRLADRIGRGQFYSDLRDVEILEEYVAGGKMMFIREQSV

ARHVLYEDYKVKTRQGVRESERCTFVIAKFPIVQIARAFAYRRGFKYKELFDDSIQYLVE

SGIIPYKLRENLPDAEICPLDLGSTERKLRNSDLSLTYFIVAGGLAFAVCVFLIEILWRL

GSRRGEKFRRNWEQRARVRKWVDKNVNVMKRTQSSAFANATPPPSYQALFRPPFFFSDPD

SHRRNINGRDYWVIQKDGFREIIPLRTPSALLFQYSHX

>ClapIR75e

GNWTPEEGISRFYKNIPTTVRRKNFKKSEIIVDVVVKNPKSLEVKNFDDFSKPDENEPYF

FQDFPNVHSLFQFINASFSFRIFDSYGYLNYSSGKFDGMVQDLYEERGDISGSALFLSEE

RHNVIEYIKTFNSWGTKFLLKKPSASYIENIYFITFAKNVWIASLVVLFIFGVVLYILLN

WEIEKNIVSQRSQSRFYNKKNKYSLSDITLISFEAVCQQGTFADSRTYSGRILLFIMFAA

FMFLYVAYSSYIIVLLQSTKPITSVRGLVDSRIQCGGLNISFMMAYYTATKDNALRDLYK

KKIFPDRFFTLEEGLKRVQDGNFAFQTILGPAYNYILKEFTNQDICKLQELPGYMNTGMY

IAVPKTSQYKKFFKIGLMRVDESGLQIRTKNRVSMKPKCYNEAGNFQSIRLYDCHLVFIW

YLYGILFSIFILFIEKAVEKYKTIKITSMSNTNTSLKINTX

>ClapIR64a.1

FLICLLLWESHCILDCNIIVEFSRFKNVTQGTVFGCFKNNEILRLSKCLQRYFLRFFVVN

IDKGSEIWEKALKRKQHHFAISVDGDCGNITGFLNEHIDKTYFYETYHWMIVSETESIFG

NFGPLRLNINSNFHAAIRNGEDNNFTIFDVYNPASEHRGELKIKRLGFYNKIDGYRARCK

RNKYEERRNMTGVEFKSAVVIPKIQKPNLMEYLQSDENRQVDSMHRFQSVTVNHCKEFYN

FSLRIQRTDSWGYIQKNGRFDGLVSLLENRLVDFGNSPLLFKLDRLPYVDYSYGNWILRS

TFIFRRPKVVSRSYEIFLRPLERSVWISISAILVVILILVRIIFCHELRFFRQNPICLES

SWSFLILFTLGAFCQQGATCYPTFYSSRIISISVFLFCILVYQFYSASIVSYLLMDPPRT

IFSVNDLLKSQLRVGVEDILIDRNYFIQTTDPDVIGLYESKIRGKTNSSGFYQPPEGLEL

VRKGGFAYHVETSTAYPIIEETFLNQDICELDEVQMYRTQPMYTNLQKGSPFREMMNFCM

LKLVENGNMQRLRHHWDARKPTCIESAKKKEIHVGLGEFSCSPIALTFGVIASVIFLIIE

LISCFKNNILNFFKLHIRLKKNAKVYPYLKX

>ClapIR21a

QSHNRVVVFARSSQWRVLDFLMHKDSRMFLDILVIVQSERMSSHEEAPYILYTHELYVDA

LGSSRPIVLTSFHKGRFTRHVNLFPRKISAGFSGHRFIVAVAHQPPFVISRERSFDGDKT

FIGAEIRLVDMLSRKFNFTTDYREATEDIFVGSSEAVTRTVEKGEANLGIGGLYVTPNKL

NRVGMSRWHSQDCAAFVSLSSTALPRYRAIMGPFHWTVWMSLVIIYLFGIIPLSFSEKHT

LKHLIKNPEEIENMFWYVFGTFTNCFTFTGDKSWSRADKVATRLLVVFYWLFTIIVTACY

TGSIIAFVTLPVYPDVVDTISQLKSGKFQIGILDKGEWPEWFVNYSDPSTEKLFNDLDLV

PNVQSGLKNATTIFFWNYAFMGSKAELEFVLRTNITLKNKKSIFHVSEQCFIPFSLALIF

PKKSIYRDILNGGIERITESGLLDKIKFDVEWETMKTSTGKLLAANSRPSTR

>ClapIR75f

LLQSSSSSIQTLEDLLKSRLRVGVDDTVFNRFYFPNSTETVRRAIYLQKVKSGKKEKFMT

MEEGVREMRGGLFAFHMETGPGYKLVGEIFEESEKCGLKEIQYLQVSDPWLAIQKRSSYK

KFLKIGLRKIQESGLQNREVNLIYTKKPICISRGSSFISVGLVDCYPAVVVLAGGLILAF

LIWLLELFIYNRKNVWFNMKRVFPSNNKNTSTSKVQYLNX

>ClapIR64a.2

PFQFQGLTTTPLSYSGRILIMSFLIMSLMVYQFYAANLLSTLLMKPETRIRTVEDLVTSE

MRVGCEDTLYNRDYFKHAVDKSSRDLYAKKILAGQNKTSFLAPDDGLALVKDGGYAFHVE

LSTAYPIIEKTFPVQSICRMREVKMYRTQPMHANLQKRSAFRDMFDTCLQRLAEHGILHR

ELTFWHPRKPECIESSSVSLNMGLDYFYPSLMFLAIGMILSLLILAVEVYIDFKERAMRV

GKCSGVGPVYAFSPX

>ClapIR40a

NKILMSIFEAGIITKMTENEYEMLGKQKELTSDIAESVKEEAKKETRLPKKDTEGNEKLK

PINLKMLQGSFYLLCFGNAFSGLILFAEIFFHKQHIRYNSKKKRFRMARRFIKQIRFKVT

QLRLAIRRFHRNLMHEAFISTLEYMEX

>ClapIR93a

YVHNFSPVYEYKGIPMRGGLSSIQNCVWYMYGALLQQGGMHLPHADSARIVVGSWWLVVL

VIATTYCGNLVAFLTFPKIDIAITTVEELVAHRDTVSWTVREGSFLENKLENSSAPRYQM

LYKQRSRRRNTSIDSLIESIEEGKHVYVDWKMRLQFIMKRRFLKTGRCEFTLSTEDFFEE

ELSLILPQGSPYLPRINGEX

>ClapIR56e

WISYISCFKANKLDLFASPLQWILFSSALSRMAIFEKYFSEIDILIDSDVTLLYPKNNAT

IYIKKIYQRHRKSFIVVESIGEWNDISGYEEYMNETVIWRRRNDMKGTQLNACIVITNNN

SMNHLTDKRX

>ClapIR75g-B

SIFAQQGSAILPAHLGCRLIVLNLLILSILLYNYYTSSLVSSLLSSKPTMLTTIRELYES

NLKVGIEQQPYTITYIIQQKNDVYIQKLNSTKIYESEVPNFLAPEVGIDMVKNGGFAYHT

TVETAYPLLARTFDQESICDLAEIDFVTPGVVGLTLQKRSEYTELFQISLVMMRVRGIIK

RAKNFWLPKKPECLLGSRVVSVGANELFL

>ClapIR93a-B

RRLLGSVMKSAKSSGLVDTRSQWLYIISNAKNSSSNVEFTRRLLKEGDNVAFIYNTSKRS

NDCVGGQMCQIKEVLTAFSLALDQAIQEEYEAASQIAEEEWEAIRPTKLERRDFLLKIIK

TH

>ClapIR68a-B

NNHTDLLSDAELFRDKTSDLQNQTLKVILFAHTPGITKTNTTDQSGRATLTKNGDNAIKF

TGTEAEILQSVAKAMNFRYEILEPKKASRGLYTSLIWEMVGTGADIALADLYYTSYILDV

MDLSVPYNTECLTFLTPESLTDNSWKTLILPFSPLLWTIVLISLCLCIVSFHYLARFHIK

ITEMKKQLGKAKSQSYPTNKMKVITLSMHPEVEKLDPDVKYAMMKEKYQTVRAEGDPVGL

YQFSEPVNSALYTYSMILLVSLPKLPTGWSLRILTGWYWIYCLLVVTSYRASMTAILAKP

APKVKIDTLEELVKSSLTFSGWGEINGEFFKSSSDVAMQRIAEDFVLFNDSEKAVDLVAE

ASYAFYENSNFLKEALVKRQYRFQKQSENESTGLNHPETIARADRTLHIMKECVINMPVS

IGLQKNSPIKPRVDKYVRKVLEAGLIHKWLGDVMQNILNAEVQTDDSGNSKALMNMRKFS

GAMVALCVGYFISVMALLIEIVYFKKVISKNRDFNKYSRRIETTTRMX

>ClapIR41a-W

VKRNPMQMLRQSIFSIVKIFFLQAVTKADQLRSLIGKYLMGGFFVFSLFLCSSFSSGLST

IMTIPRYENPINSVREFSASGIHWGGIQDAWTISIENVEDPIYREIVKRFVATSEENLRD

YSKTSNFAFPLERLPNSNYAIGTYIQDDVIDSFHLMQEX

>ClapIR40a-W

LSSSHKARFFIALLSVSATYVISDMYSANLTSLLARPGKEKAIHNLYHLENAMQTRNVKL

FVEQHSSYYGLLENGTGVYTKLWDLMTNRQDGNFLIKSVEEGVX

**C. lapponica OBPs**

>ClapOBP32

MTKTFSQLALVA

ALTAVIAAYDFQDLEFNQILAGDLEDVYTDTFSHPRVRREENSPEEDKCRPKRGRPLCCG

EELFKKPTDDDKDMKRACFKEITGKDKPEGRPDKHHGHFGGPLDPFNCDRVQQFRKDMIC

IEQCVGQKQNIIDSEGNLKQEDFQKFVQNQLAKDPALAKIADKVAAGCLEEVKNATALAQ

KLETGDGCKPVGLALHHCIFKHVQLNCPEDQIKDKGACVKFQERLKKGREGMPGGPPGPP

GPPGPPGPEGDEE

>ClapOBP28

MFSSIVVGISLVFSISAFTEEEQQMMNALHAECVSETGCSEDLISKASSGDFAEDDKLKC

YMKCIFDELGVIDDDGKIDASGLLTIFPEDIQAIAKPMITKCGTVAGKDLCDSIYQTNKC

YYAENPEAYLLPX

>ClapOBP26

MEVWIVIVGLLLSSDAVMGLSEEMQELADMLHATCVEETGAKEEDIENARKGEFADEENF

KCYIKCIMAQMACIDDDGIIDEEATIAVLPEEYRAKSAPVIKKCGTVKGSSACENAWLTN

KCYQHEAGSEYFLVX

>ClapOBP30

MKWLIVFINLLIITHYGYSEMTEKQLKATQKLVRNTCQNKVKASSDELDAMRSGNFDQGK

TAQCYMLCILNTYKLFTKEGSFDWEAGVKSLKSVAPEKIAGPGVASIKNCKDANKATDKC

MGALEIAECLYNDNPQNYFLPX

>ClapOBP01

MRTLKPMLIILLSFIVLNVTGMNEKQMQAAVKVVRNVCMPKTKATAEDIDKMHQGNWDID

HNTMCYMFCSLNMYKLMNKDNTLNYESAMKQLKMLPDTYQDATKVCMDNCKDSVVTLDDK

CIAAYELAKCMYICNPEKYFLPX

>ClapOBP21

MLWRSILYFLVLLGFSSCIELPPDLQEFVDDLHKICVSKSGITESDHAAYDVKSNPHDSK

LQCYMKCLMMEAKWMNSAGVIQYDFIIDTAHPAIKDPLEAAMNKCREIDDGANLCEKASN

FNFCMYGADPENWFLIX

>ClapOBP11

MKSTYASIFLAIALFSASLAARNNRKTLLDAGDHKKVLTDCRTKSGATSADMEAIRMKRL

PDTKTGRCLVQCIFDSARIMDAGRFSKGGMVVAFTPALKGDLTKLGKLRQLAEVCEKEIG

TGVVGNCEGPRRIVECVTKHGEAYGFSYASTNMX

>ClapOBP23

MRSLIVFVALAVAVQSVDQELVNEFTEKITEIGMKCAQETNANNEDIAALVGHQIPQSKE

GKCMISCVYKAFKLQNEDGSLNPNGISDWMERLEKSDEDMYGKLKQVIDECLKPENVLPD

HCDTSVKVSSCASETAKKVGLGTAVMNLX

>ClapOBP10

MSTVRHCLLYGVCLVFLSLRVQAAEIYKNFTNKCDIPPTAPKKIEAVINKCQDEIKLAIL

SEALESLNVNEHTHSRAKRAAFSDDERRIAGCLLQCVYRKMDAVNDKGFPTADGLVSLYT

DGISQKDYILATVDAVKYCLGFTQKKFRVTPNSIDVQGMSCDIAYDVFDCVSDEIANYCG

QTPX

>ClapOBP24

MKYNMLNIAGIMMVATVLPSILGYKMNAVHEDCVKSSGINPEELERQPPQTTKELLCYFK

CAMEKDGFIDGKGDIDLTTMDKYAPNGASDSMKNEVKQCFEKIGKVSECKDVEKVIACIP

MX

>ClapOBP13

MFILSIGLLIVLSHVTTAEKIDIKLDEKAKEIFEECMKSSDVTMDDFKEVLASGNFTRNI

YCMMKCQFEKNKFLRDGIIDISQLDDEDKLLIYFNKEQLPEFKKCLAHVGKIEECEDVEK

VSNCFPHKTEX

>ClapOBP29

MMKRLIVLFLSCYVVHSKAIECGIDKSNRDEIKQALAMCVKNNDTMNKIWEMTTSSQTAP

SSAEQEVDSTEENDDESVSRSNAKNQRGGKASRIKRAKSAKSFNNQKVSTTTMRSTNNNN

KMKSTDDDDEEGNIGSNDVSEETSNSSQDSSDKCIIHCVLEKLSMTDDNGLPDHSKILEE

LLKSGPKRELKNFFQDSTDECFQEVDEANESDSCEYSNKLVFCLAEKGRSNCADWPAGNL

PFX

>ClapOBP27

MYKTLVYTVLLISLMEFSLTWKTDDFGKEFVELMTILHNTCINRTGTDDAAIESIKHGQF

IEDDKIKKYITCLWQESTVVESDGTMNMDILMNLCPPKLKDTVPKLISDCWKEVNGVTDL

AEKIYEMEKCLYRTSPETFIMFX

>ClapOBP07

MLKVPLICAVLLSISISHSSALLDKKDYGTKALELANHLHNTCINRSKTDDATIQKIQVG

NFVEDDKIKKYITCLWTESNVVDADGTMKIEVLGEIMPPKVKESHPKFVAQCWKRVQGVT

PLDEKIYELTKCLFEVNPEEFVMFX

>ClapOBP12

PATKVDKDTMWRFYNGEQVDEVQFAKHFLCMNIKLGIMNEHGDINKGTLTPSLQNNVKEK

QMFDECAEKKGNTAEEVALAMAKCIRKYKLARKQDHAHHHX

>ClapOBP15

MKTIVLIALTMALVAMVAAKPHGADVEAFRRIHDECQADPATKVDKESIEKFNKGEEVDE

VQFGKHSLCISIKMGVQNEHGDIDKEALKKGFEGSHNDKAQQIIDECGEKKGHTAEEAAL

ALAKCMRKYRPAPPKQDHHGDHHX

>ClapOBP16

MKTIALIASILAIAAMVAAETKEEKIGRIKSVHEECQADPATKVDENVMEKFFKGEPVDE

VQFAKHSACMNKKTGLQKADGDVDKDDLKKTLGNHEKVDEIVEECGQRKGDAEETALAVL

KCLRKYLPKPAPGHEHHGHX

>ClapOBP03

MKNIAFIACALAFLTLSVSGRNDRLRRIHAECQLDPAYYIDESLIKRAMRGEQVDESALG

PHVICINVRAGIQSRNGDIIEEGLRTILKERIRNSSDVEDVIRRCGRRNGGTPEEAAVAL

TRCLHKYARGHHX

>ClapOBP05

MKCIILLYIVSLLIVLGNTQSELGGTFETARQVCQTDLLSKIIEDMKRRPGSQHNYQMSK

ELVNDQLLCMYVNSGIMSREGVINSAVLEQRLREVIRDQDVLNNILRDCSASRRHGSPPE

TAAGLYGCITRNLKX

>ClapOBP08

MHNKLLFFCITTAVICLGGTQKNLTGEFRKAHEFCQIRSDTRIEEDLMDRAAAGQTVDEY

KLGNHLFCMYIKMGIMSRDGSIKAATLRQQLVEPVKDEAVIDGILRNCVPSRVQHGTSQE

VAAELFRCVRRNLRNAX

>ClapOBP02

MKTTIFLFCSASALITLVSTQSRLFGPFLDAHNACQEDPSTRIVGNLMDMAAAGEAVDET

ALGNHMLCMYLRLGIMSRSGRVNASRTIDLLTDVLNDRPGVSRAVHSCCGPRRPNETAQQ

VATKLYLCVTRNLPGLSTVTPPAX

>ClapOBP14

MKFAVAVVLAVAVCFVQGDLSPEQVEKVKQHHKECMQSSGITPELLAKTKKGEFPDNQKL

KEHMFCFAQKADLMDAQGKIKKDVLLAKASTALGDRALAQKLIDECAVQKKDGPETAFQC

VKCYYEKTPSHLSLVX

>ClapOBP20

MKSTLVVVLCVAVVVLANRLPESEKVKLAIAHAECQADPKTRCDENLLRNLGANANNAQV

GIHMLCMSQKEGLQKANGELDRDFIRKAIELVSDDKSKVDEHLQRCAVKKESPEKTAVDL

VLCFVQNGIPYYYKLX

>ClapOBP22

MNTTLILVICLISIVKAGMLTDEQKKKILEYGQECLKESGAELDVVLDAAKGKYVDDPKL

KKQILCFNKRIGVQGPDGKLVMDMVKTRLTAVTKDQKKTEEIVKQCVIERNTPEDTAFET

AKCLHQMVPDEKIVX

>ClapOBP17

MKIIALVFIVLALITDGKCLTDEQKQMMMNIHTECLAVSGVTDAMIEQAKTGNFPEDSEF

KEHLLCFAKKVGIMSDSGELQTDMITKMVQMHVTDAAKADEIVGNCLVNKATPQDTIFES

AKCMFQVYHFFX

>ClapOBP31

MHIFLFDENKMYLKFILVLFIFISKTHTQFPGGQIGNVVRFHRECQSRTGVSDATALGVT

SGRFPNDAAFKRHLLCMNQKMGFQDNNGNINRNVISRTLRTAMPNANIEGVMRNCAIQRG

SPEETAFAMDQCFFNNLRSSAGGMGX

>ClapOBP09

MGMSLALLVLLGISSIYAEIDINAQGPALGKVHMPLHDKCVEKYPVTDDDIAKVRSGIFD

DNDVQMKEYILCLWITSGMLDISTFRLNNILLEVYVPEILNRGKGSDMYLECATRGRNLP

NGTPLRERTWVLMKCVQETDPEHFIMFX

>ClapOBP04

MEIVNILLAIIGATLINAQLEEHEFPPAYLHLISKVRHHCLELHPISEAERQNARNGIFD

DNDEEMKKYVTCLFTKSGFADSNLHMNKAVLKAFIPEAVMDGEGVKMIINCSEEARNKLA

SSPLQDKVWDMLKCVYHEDPVHFILPX

>ClapOBP18

MGRVIFVLFFTFAVAFCKPTSEDAKAELLKFHEECAVTSGVNAKDILDKEFDDIGDEDQV

KTHVLCIGQKLKIIDDDNKIDRDVLKTHLGEIITEDGKVDEIVTKCAEEKDDARETAYSM

TKCIHEIMRENX

>ClapOBP25

MTKKPISMKITIFCFFLVFVSYGAVPIPTSNIDDMKERERMGLECLKEVNINRKIIDNAV

KTLDFPRQNQKYKDFLSCSYKKQGFQSEEGHILYDKITEFLTRYYRRSDLKIMENCKSTT

GNDHGEMAFNALQCIISDLKKMDDIKNNX

>ClapOBP06

MKTSAVTVCVINIVALVLVSGKDIRTYFQECQSDPSTHVDEDLLRKGLRGELADETAVGP

HALCMNIKRGFQKPNGDVDVATLKKYLDQAEDRNVTLIDQVLKECGQRNGTDAQRAALAL

LDCMHRIIPHKEEHHQASVSAYRFHDECQSDPATLIDEQIFQKFLRGEEMDEPALGPHTF

CINVKAGLQSQNGDVVRDGLKAFLSGKTKDTSAVERMVKDCGTRNGGTPEEAAVGLAKCL

ISNAKGVIDFISVFEKTEEKHX

>ClapOBP19

MRFSLAVVATAFVVAIYEISANPVNPNEGVVPLFSHSDCQARSGVDEYQILHPDEGIRSE

DPKFKEYLLCFLQKSGYINELGEFHQEAIRKQVKDLHDLEDNLVVDEFLRECAATNGQPL

EDAFGFYKCYHRHYPQEGFLYRSSIGNKVKEALTQTHSECRKESPLEDALLEQFKGGNFD

HPQAKKHLHCLYKNTGYIDEFGEINQENFRKRLAQYYPESSLVDQYLEKCLIKKESPEET

AFYLNSCFYENTPHKFNIFSVLX

**C. lapponica ORs**

>ClapOR-B02

FNMYVLSFFGAIPPDFRNSNLVLKILHFLRVSLMGFICGGLIICQMVYLYLTLMGGPIHE

IVKAAYLTMTNLLSGVKLYRVYQTRGRIMSLVQTMNDTVFQPKCQHQVGVLESYMRLSKV

VTIVLIIISNMVISLMSIYPCTV

>ClapOR-W02

IGITSSFLMMEILHLNIKALKLFFLWPNVEDTIFRRVICSILFLLMVTLSYLENAAAIVY

QIIVNFEDEAVLTDCLIATGNYFGYIFIYLCFQVNARKVQKLTVDFNNFLRYCDVEVIDD

AEKEIRLFMKILVIYFFSGITLNDLMPVLDF

>ClapOR-W04

VPFDEQKYHLPNFWWNVLDGCIGASFLAYSEILSFSLIIFPLGKIHILKHILTNFDDYVM

KIGNQIGVAKEEASFITLRECILNHKEIIRWVYXKKNFRFIGDLNEAMRNVX

>ClapOR-W01

MIYKTHFDLRSSLVEATRPEMPCKIILKWTRYFMVASGALQIRTSSRISKIIYTFWAHLS

HAYYFFFCVSLSIGAIYADDQEKLDYAKETILLCLLLLWKLAISCSNQILRVVEEVQEVE

AKITAGEITRIYDGNCSHNYKIFFFLTSLYFSTCYLYSSNAIQQWRLMQTGAERKSLLFP

GWYPFDSDRYFDLAYFYQITIGFFIVLYSSTCDSLFISLVNFATARLAILGYELEHLGTS

STRGKLPVYDCLRKIVKEHKEIIRYVENLNAMLRWYFFGDFLVGSYHVSLAIINVRHHLY

EESIFFHSFYLMYILSQVCCLYFHINELILESTNLSRKIYEGEWYDQSLEVKRSLLIVMM

RCRKPLILTIGNFGVINNNLFVKILKAAYTFLLYQMLDILRTX

>ClapOR-W03

LEEKYEIKNTLVYRGICENVEHYDFIFRFVNDIQDTYGFVILTQFVVSIIVICMACLQLS

IAEPLTPLFFAMLIYIVALLIEVLFYCYYGTILYEESNSIVHAIYMSEWYDYDEKSKRAL

ITLMERTKRPMIMTTGKLLDLSLQSWTWIIRRSYSL

>ClapOR-W07

LLELSLASGIEKHSIENTHKFFXVVPFTFKFFSTIIFALTMLIQIFLYCYYGSLLREESA

TINSAIYMSEWYKYDKKSKDILITLMERAKKPIKITAGKFFELSLDTFTSILRRSYSL

>ClapOR-W05

TYAFSISFVIRIKSIAKLYSFFSNFVTFGKPRDFDSDNKYFNTLSKLHFVYLECLICLSL

FFSNILKKKTCQLENEEYGLQEICGLFTYTWMPFNIDFTPVRQLYTLGQVVGGQYIYMLA

GILAWQVLETMQHLIIRIRDVKYKFEDAFQQNDRRVRKKKLISAIKYHKAVVRLQDDLND

CFGVFMFTHVIFTAAIVGTGIYCFLHRKSLSSFLVCIGWFIGLLMDCFSGQRLQDESLLI

AETIYNSPWYDFEKELKQQVLFVLANSQKPMRLYAGPFGIMDRATFLAVMKATYSYITLL

>ClapOR-B01

LISMTGISYFNLCFVMNRSAITEACKMLSDLQTFGKPNHFNRTNANLNRSSTVHFFAMLL

ALLVFALTSLPLWLPSDLDFSPAKQAVFVVQVLACRFSYVTASMITVFEWECFEHLVVRL

RHVGDMFVRALEQDSYEKRREHLGRAIEYHNFVX

>ClapOR-W06

AQYYFVLILNTYASLNIACVVLVITLTLVGLLIHLNAQVKHLKKKLLDIFINNSDQSKKS

RNDVEEEIHLCVQYHNDIISHVGEIFRCFGVLLVVHVTLTPFVFGVLGYRIVSVENFTDK

>ClapOR-B03

GDWKLFFLTAYYLLFSIYMYSMITILNTSNYIISHINFQYHILNHYVIRLAKLSVHNDRV

GYQEAMYKEIVNLVQMHDKILKFSNKMLAYADIGMLALLFSIIQMFLCIAGFVIFNVQPA

DNLRMASSLIPMIVFAVNICSNGQRVKDESERVYYNLLECGWYNX

>ClapOR17

LLYLNVFALYTLTASFSNDNSWNVLFALREYSKEWKSLVTILDYSVAIIGAYAITVNLNA

SNYIICQIIFQYHILNHYVIRLARTSMKNKDRFGYQEDIYNQITTVAKMHAQIKKFRNMM

LLYGDYATLAFTIAGIQLCLCVSAFIVLNVHPESNLRISSTMVLVVMFAANLCSNGQRAK

DESERVYYNALECGWYNWNTKNRRAYLMFLINNMGTTTFSNTGVYDVDHPLFMFICRTGY

ALLTLFMGVREKSMX

>ClapOR30

VYLKENIRKISKIQDYHLKNHICHCVRYHTAILQISKDLNDAFSVMMLIHITWTSFIISV

LGFGIIMENNYWNMLRFIMHLGGWLLMLFLVSFYGQILMDKSSDISKEVYDSEWYRTLPK

IRQHLVLILLRSQRPIALKAAQLNTMSLSTFLGVLYSAYTY

>ClapOR05

MKEDPIKVMKNSLNFLRIPYLFPKSEDIDNPKKNLYIRFCLLYLTTYYVPLGAAFHLAMT

IKQGTYANIDRDVSAIISYHGASYFNFRCLISLKNMIKLYKEYSDFESYGIPTKFDKMNN

LLNKISTLYFCYHTLIVTGMTTSTLLTIGKCEQENISRNISEVCGLVAATWLPFDYNYFP

LKQFVYGYQLYSYFLIFQCAGLLSYTLMETIEHLIVRFEHVADVFVMAVAEKDPQLRRKK

FNAAVKYHKAVIEMGELLNTCFSPGMVVHISLTGPVLGVAGYRFLTEIPLDSTCLFIGWM

ISTSVVCSGGQRLSDASTALGHVIYSVEWYNLETDLQRDLGIVMMRCQKPVMVSAGPFGT

MTYATMMVILKTSYSYVTLLKQTMX

>ClapOR12

MSDLDQEKNTEKPKDIICMPTSIKVFRLYGMFPSANRLLNPGKKFYIKFALIALYSSLVL

VGCTMHLVKNIKDNTYNHVELDITYIVSMCAGYGLICSYIAKVKFAVQLYLFLSDFSEFK

KPIDFEETNKKYNRYSIYHYCYLESIVIFILLGSNIFKGDQCRKENEENNLNEICGLFTY

TWMPFNIDFVPVKQMYLFEQLFGAHYVYMVAGLAAWMVLESVEHIASRIRHVTYLFNDAL

NEEDSEKRRKKFNFAVRYHIAVLELEDKLNKTFSVFMFTHMVMTGAIMGYGVYAYIKGRN

LSSFLLAIGWLVGLLMDCHSGQRIRDESDSIAIGLYQANWAHCDSGLKKDILFVLMRCQR

QMILKAASFGIMDHPMFLAVLKASYSYITLMTQNEKRDHX

>ClapOR26

CLANYIITSSAVCCFLPFETAELLISHIGYLKENFLRVFQVDHERMRMKKLRFCIRYHIF

ILGMADQLNFLVKFTLGHMSLICAMVFGCIGNQIFRAKPLGAAIFLLGYMVSLFLLCYAG

QRVINESLSVVDVVYESAWYEGSIEMKKSLKFVMARCQIPSRLSAWPFGFFSFPLFLMIV

RTS

>ClapOR23

LIMCHLCLECTEFLISHLNAVKHIFIEAFSLDDEELIRQTLRLGLRYHNFTISLGDQFQD

LVKKTFSVIILMCALVIGCIGNQVVKDKSIGSCLFLLGYSSGLSMLCHAGQRLHDESVSL

VDSIYGSKWYEADVKFWKDIPFILARCQQPMKLPSFPVGYLSYALFAVIMKTSYSYLTVL

NNSSEKX

>ClapOR10

KCFCSTEQLVYISTYNFIYLSMISFISRCASLMIESFKASTFIHILQTGMFLGCLVYQIL

RSPYIGNFVIIGGYMNSFILLSIVGEKLAQECLVIGNHIYRHTKWYNLEIDLQKDLILVL

LRSRKPVHIKAGPYAVMTHRSILSILKTAYSVLSLLKALSLGX

>ClapORco

MMKFKVSGLVADLMPNIRLIQASGHFMFNYYADNSGALHTLRLGYSCMHLIFCLLQYGAT

FGNLVMERDDVNYLAANTITVLFFTHCITKFVYFALRSKLFYRTLGIWNQSNSHPLFVES

NNRYHALSLKKMRTLLICVMSTTIFSAIAWTAITFMGDSVHRVKDPDDKNVTIIEEIPRL

LIKSWYPWNAMSGMTYYVCLIFQIYYVLFSLTHANLLDSLFCSWLIFACEQLQHLKEIMK

PLMELSATLDTYVPKSADLFRAPSANYQDNLIENDYNTKNEELNLKGIYSTRQELGANFR

SGALQTFGQGGGGVGPNGLSKKQELMVRSAIKYWVERHKHVVRLVTAIGDAYGVALLLHM

LTSTVLLTLLAYQATQINGVNTYAASVIGYLVYSLAQVFHFCIFGNRLIEESSSVMEAAY

SCHWYDGSEEAKTFVQIVCQQCQKAMSISGAKFFTISLDLFASVLGAVVTYFMVLVQLKX

>ClapOR28

SDTLFVILEVGVCILKFLPFKNRPDAIKRTIYAVDNNEFNRATRDQSHLIEGTVKSCRRM

FVIFLILCLGSLFTWPIKVLFYEERKFPIDVWLPFEPFEDIRVYLGVFLCIFVATGNAPI

GNAAVDTLIPGLIHHAATQIKIIKDNLENLGQRVEKYITEQYTYRSLEEKYEX

>ClapOR03

KIKEIMDTLQSEIYVAVDDFNPGEFMQKQKIINSVVSIMMFSLYCSTTLGTPIATIMMMD

SGLEDDSFEGTNLTCYDFLPCPFYIPFPSNTKSGCQITAVFMGIGFLWSSAISAGCDTLF

LGLLSCLKTQLVIVCHVFKTIRERSLLKLELPENYDVLDDSENPVLEKELYCQLKQTTKH

LITLLRIKDDMEEIFTFATLGQCITSLFVLASCLFMTSSVPVGTPTFYIQILYFASMSVE

FSLFCWYGNEMTIASEAVASSLYESYWYSGTAQYKNSMIITMTRMQRPAYLTVGKFAPLT

LNLMIAVYKASFSYYTVFKEFQX

>ClapOR22

GTSAHISTIFSLNIGLAGESLENINATCYDIMPYVFHIPFPTDQKWECNIAAAFMNIGLQ

LFAGVIASNDAIFVGLLGCLKTQMIIVGHVFKTLRARTLKKLDLPTNYALMYDSENPILE

KELYTQLNKAIEHLMILLRARDDIESMFTYVTLSQSIASLFIIASCLFVASTVPIGSPNF

FSQLEYFVCVLVQFSLICWCGNEITIASATITTALYESDWFSCSARYKTSMIITMTRMQR

PVYLTIGKFTPLTLTALITIFRGSFSYFTVFKSIQX

>ClapOR27

ATFLFVNIFFTGTEFLSLISTFSNEYDFIKNISFLLTHFMGAVKVVFFYFHGSKLKRIMS

TLESPQLRYGSCPEKGFFPGATSERFKKTGIKYTMLFFILAHATLISSYVPX

>ClapOR15

MEDDGLLDLRDIVRINIDVLYFFGNMYPRFRTSVVKLFYIARLSIFFGFFFMGIVSAELA

NWYASMGNLQAMVNASFLTLSNMVSMIKFYAIFRHQDRIFALLESTNRKEFRPKNRQQRS

ILRHFIRTMRTISLLMYGGCLFTCAFWAISPFTDEDGPYLPIAAWVPFEPSKGTIYFEIV

FAYEILATIVGGITDMSADCLIAGFLIVVCAQLKILNNSLSNIRQLSLQEERSGLLVHDS

DDDSNEPEIMWMKLQAVMDDKLVECVVHHRCVLEFAEELTSLFSYSILGQFVVSVIIICT

TLFEITMVSFMSVKFFSLILYQYCMLMEIFILCYFGNEVILESNKLTNSAYHCNWRPCSM

HFKENLLFFMTRSQRVLKLYAGGFFTLSLDTFVRILKSSWSYFAVLIQVNKRKLDLYEIS

QGAX

>ClapOR29

IYPCTVAKKPFLPLAAHIPLNTDYSPTFEITYAFEVVGVLLGAWAGVNTDGLLIGFIIFI

CAQLTLLNDSLEHLREHSMDVTDANEDASRGVLDEKMKATLIDCINQHGQISEFKKEVSS

IFGTITLSQFMVSVLTLCTNLFELTTVSELNVQLFALLFYMMFLLTEIYCMCYFGNEIIL

KSFQLTNSAYHSDWPNCSLDFKKNLIVFMTRSHIDMKYLAGNLFPLSLETFVKIIKSSVS

YFTVLNQLGDSSEYETRNNLX

>ClapOR13

TCTLDLIKMLAIYRNMDLIKSLLKKLNQPLFQPKCKEHLVLAASVKKFHKTLFYFCLYFG

VQTYVFFSAIPFFRKDLVQLTQGWFPMDWRRSPNYECVYIFQNVVILWNTLIFLNLDTFT

SGLLMQVGLQCDYLRVTLNYLGRFSMVESVLCQNDENVCEMDVQMFNETMSQHLVTCIEH

YKEIKKLSKDIEDIHHTSVFILFLGGSMVICFALFQLSMVELGGIQFCLFVSFLLCMLME

QFMYCWFGNEIIYKSGRILTSSYNTPWLDCDKKFQKILLIFMTQVKNPINLRAGGLFTMS

ISVFVSVLKSSYSYFTLLQTMQENETTEX

>ClapOR02

MFPKESKMLINKQDFHFRSTIYLEEKVLNLSGLHPEKLPQLFSWHTLKPLTSLAMVLYLT

VSTSMTVFHYDFDVVVESIMLGFGVLRFLCKIAVFLYHNDSFRCMMSDFEDPVFYSLTPE

DLAHFRAKAKQTRILGLLHFSSVMVYTTFLAVSPFIFDKQFQLVLIWEPLDLGVITYLCH

LYGLCFAVFNHVSMDNVFMMMSTFAGCQFDVIISGLGRMDFGRHGESEVVLRANIVRHLH

ILRFIKRMNKLVSYMALIQYLMSIMAICVTLFQIMKNPQGGSNYAFLLCMMVEIILYCWY

GEAIKDKNDDLSRTLYLTNWEESHVKIRKMILIIRERCKKPIRLEVGGFTSMTLNSFVNI

MKSAYSSFTVLRQVYNX

>ClapOR07

MMPRTKILKWTDHLLLAAGASHPTPRGPYSIPYHAWTYLSQANFLFFCVSLGAGAMNTKA

AKDELDRNEAFLLLCLLLLWKLAICRGSRMREIVGEARRVEVGIPAGDHEMGRMYDRSAS

SNHGVFFFLAAIYFTSCSVCIFNGLQHWQLMRKAGAEMEKSLVIVAWFPFDYSGCFDLVY

FYQICSIALIPLYCTAFDSLFISLMNSATARLAILGYKMESFKEQSTLEGQSTYHYLRDV

VMEHKNVMRYVEDLNESLKWFLFVDFLSRSYHVSLTIMNIVRHRSEGPIFFDFSTIIYLL

SQIICFYYHANRLILESTSLSMKIFHSEWLDQSPEVKRSLLIVMARSQKPLVLTIGNFRV

MDNNLLVQIVKAGYTFFLYQLLDILHX

>ClapOR11

VAGWFPFDAQRNFAAAYFFQATAALLLGFYSLTFDSLFISLMNFPTAILDVLGHKLEGFD

VYGERRRRQSVGACLRSIVVDHQNIIRYIKDLNDSLKWFFLGDFLIKSYHISLALTNVVH

ATKETFGLDAFILMMIFTQVLSLYYHANEIVLKSGTLCRKVFESNWCDQTPELKRSLTIV

MMRSQKSLTLTIGFLGVIDNELIVKIMKAAYTFVLFQLDIX

>ClapOR25

NENWQIIYKAYSSIILAEFLIFVVTLLIGWKDLVHGSDLQLANYLQYLITYVFTAWKIFL

VRSAPVKKLVVEILSLERAKMASNEDIEKIHHDVSRHNFKIFGSLLFVTTMAVIQFIIRT

SENLLMWKKAESQGIETEKALIFPQWFPFGTEKVFDVIYVYQICNGTLGGGLIVATDTLF

VSLILFTSFRLRALGYELKNFGTEMEDECKQNTKX

>ClapOR16

MPTKIFPPNEHFRVTMYATSLIGLWPCIFEDNDFLNRIYKIYSIFIFTVAVCVTYCTDFK

LFSLVTDEVLFLDEAVENLCISCLHTMILIRAHRLKSKSVTKIVRRILTIEEEIYKSEDE

AIIRIYNSYASQSRLSNMFYIIIVYLATLFYIIHPVFLGDIVKYYPSKNLTVYERPLPLK

TWFPFEKQNHYVLSYVLEVCGGCVVATFVIFVDIFNFSIIIFPLGQIQILKYTLQNFNTY

ASRLQSREDCSRDRASFLTLRGCILKHKDIISYVSEVNDAMRNVMVLDVLQSSVQLAASM

LQLVMAEQKLENDLKAGQFIASMLTRLTIYYWYANEIAEQSSDIASSVYDNEWYNQSEEV

KKMMCMVMLRCKKGIGLEIGTFRIMTLKTLLSILKGAYSYMTVVYKX

>ClapOR20

TQGAMVYISRGNFMEMAENLGVLIVYVAYIYKIRITKSKSIRDMVQKLKEQEGHILENAD

EEIRKIYMEYAESTGKIVFFYFIVGTIATVVYFARPPIENYLRQPETKYLIFPSWFPFDT

NEHYWIAYSIQAAAGLLGYSYIVFQGTLYCSFLIFFVGQLHIMRHIFKNITHYISKFSDE

TGIPYVKAQEYFVKICIKQHQQIIRSVRSLDESVKTFLLLDFATSSLQLSLLVVQLIDGT

EIIERCAQLWFLLTLCYQLVFFYWNAHEIIVQSTGIADAVFESDWYKLELPVQKLLTMVI

MRAQKPMSLSIGPFYEARVDAVVSLYKAIYS

>ClapOR24

KYYFTAFWIEVWCMISATTSNSVTQTFFVTMMNFMIGRLIVLQQHFKNFDSYETGENERK

TITDEKSVLVALRGLIMEHLEIIGLVENFDKSMRSLMLLEFMTNSLQIASLVTELLMSDS

ILVIFHMITYIVIVTCQLFLX

>ClapOR19

LKTTMYEKEFRNVFRLLNVVGMNPVKGYKKALIVFNTTLTLYVTVLILMNLFLKKELVTV

ESIGVFSQIWLKLVILLTKRRQIKEVIEHTQLFWHQDPEGSRNSSMLRSLRKLERTFLVY

IFCSTCMFLFKPLLVQGSTIYYYYKVPEIPFLVSYAVEFYVTLVTMSMVIAVNIFVSISI

VIGAGQFSNLNANIRKLDLEEAKTKGGFEVAVREINGNVAYHNNLIQYVTKLDDIFSLLF

VVLVGIITSLLCMNMYVLSLPNTTVVDYIRCGTMVCAFTTEFLFLYGVPAQKLVDEAEKV

ADSAFYHCDWYLPNILPIRKSLTFMIHRSQKTVGLSAMGFIDINRQTIVAMIKTAYSFFT

FLQTVESTGTEKX

>ClapOR04

KMYFHNIDHCLKMMSIIGVHPGIEKGLYHYFRYILLVLSICIIGYLVMLYPYHREEKMTL

LNMTPVVTSMCLFAHSIFKLTNLFFKGRQIRSLLKDFECSFWKIEDISESDDKAYSVAQI

NRIKQMYHIYLFMSTNTATFFYCGRIFIDGQPADRNLPFESHVPFDVPFPVIFVFELIVG

YILVIPIVAMDIFISTTVLLTALQCRLLNREFQNIFCDDNDDSIRSRFRKCNDHHSFLIR

FREELNQIFANGILAYMLVIVPTLCIEMYILSTEKSLKEDIRAFSYSATLFFEFFICYCL

PAQYLNDEAELFLNSVYSSNWHLHSKHSKDILLLLGKSHLKMEMSAGGYTKVDLKAGFAA

LKSMVSYCIFLRSLGVSQDX

>ClapOR31

RHRHSRENRIKLWVNKHNFLLDIVARMNRAFSTSLLLYFGTVSLTICVELFNLTNQGKLS

LDLIMKLIMYLIAGIFEFSTFYCIPAQMLIDEASRISFNTFSSYDWDMSNRPLGFSAGII

IMKGHQSVKLWIGRIAIINLETCLETIRSTFSIYMFLNTVVSKKX

>ClapOR18

IRSVVEHHHFLMKYTQLINDCFSSCLLAYMIMIVTSMCVEMYNISSSTDVGAIIKSAVYI

NVALSGFVIFFCIPAQHLSDEAEKVSTSAFSSGWCDRPQEFGTAVLMMMANGRPVKISAG

SFVDINLETSLATIKTMVSYCTFLRTMSIDDX

>ClapOR06

RGIKTIIIQEMRIVKCIRELQIVIGLAAKLDSLINIMMLLSFLCSLFILCCLSYLASVLS

TAFQISLFASIFGATLTEVFFICWFIQEFTSEFSIVTRNIYDLEWTSYSPRLVRMLVFFM

ARVQKPPTFKLYRWAKIDMLLFINVVKMASSFYTVISNVNTNX

>ClapOR14

KTKLLRTMHYVAVFTYAGFLGVSPFIFHSRIQVVPIWEPLDLGIFTFFCHLFATCFTAYN

HVSLDTIYTMVTTFACCQFDIIINSFKRMDFGNCKESEEVLRAHIIRHLYILRFITMINN

LSSYMALIQYLISIFVICITLFQIMKTSHGGTNIAFLICMIVEIIRYCWFGEAIKDKNEE

LNRALYLSNWEESNLETRKMMLIMRERCQRPILLDVGGFTSMSLNSFVNIM

>ClapOR21

MNGEMKKICRHLRIIYEMCEKLENVYKFLTLAQILATLFILCSCLYLVSTTPESSKQLAE

IVYMVAMGFQLILYCWFGNEVTLKADRIPFYIWECDWISADAQFKKSMIFTMVRAKRPLY

LTAGKFVPLTLSTFIAIIKASYSAYAVIKNTSRSIQIRX

>ClapOR09

VDTSEDLFVLFGGLGS

TAICALFARSSHNWHDYLQRLLDFEPFSLDVEFEEEMIRRNFLGYFYAVYYVLGTVIYAI

LTYLDSSTCHSIIEEDNVHLICDTLFPVWLPFDIPYSSVRWMIFFLQFLSSLCAVHPAAL

VCFLIWEASEIICIHIYMLKKHFKKIIEEGNIRTRPDGIAFCVKYHNHVLQLAENLRDLA

KETVGYVSLFSALVLGCTEHSIMKTKPIGGILFLLGWLASLMMICHAGERLMEETFSIGA

GISESRWYKADEETKKDFVLILLRSQKPIYLKASPLGLLNYTLFVMILRTSYSYMTLLNQ

TSX

>ClapOR08

NLMMYFFAGIILNDLMPVLDFKNCNDHRLSDYYRAHDPCGLPVRVWWPFDARNAAQYYLL

LMFNTYASLVCACVVLIITLTLVGLLIHLNAQMKHLKQKLVDIFIDFSEESTSTYSTDVE

EKLNLCVQYHDDIISQADEIFRCFGVLLVVHVTLTPFVFGILGFRIVSMENFTDKLRYTM

QLGGWIAMLFLTCYYGQLVLDESLSVSDAAYQSKWYNGNSRLGKYILLILMRSQRPLELK

AASITTLSLNTFVSIIKTAYSCFTLLITISTEX

**C. lapponica SNMPs**

>ClapSNMP2a

MMLACSKFFSVKVLIVITSILTLVFLAVLVLAFYGVPVIIDGSIQNSVHLEHGTIQWDRF

VDLPVDIMMKVFLYHVSNPVAVLNGSKPMIEERGPYCYRQKIHKNILSTNSSEDTVTFEQ

NFQIAFDQEASGDLTEADKVVIVNPAIMTLYRITSPLERLVVLGCLDKIFPSEYLGLFIE

VDIKTIMLDGFPFAQQSEDLGPACNIVRNRVLDKTLPMKNVQRVIPDDDDDGILELRFAF

LQYKMRNPDGIYTVNRGINDITKLGQIIRWNNESELPYWGRIQSINNDTCKKVRGSDSTL

YPPQVEKTRSFDIYSTDICRVVEISFQKTDSYKGIDAYRFGITKDTFRSATPNPQNDCYC

IQQTAGVHGEPSCFLDGVLDMFPCFGAPIVLSFPHFLYADESYSDAIEGIGPSHPEIHEL

FLLIEPNTGTPLQGMKRIQLNTVLMPIKNIPVTSNIIPLVMPILWLEEGVSLPQNLIDEL

NSHYFHTVKLVRGIIYGLIAVTAASALISSGFIIRKKYC

>ClapSNMP1b

MQLPVKLAIGSLSALLLIVIVGFVAFPKLITSKVKGMINLGPGTEIRDMFLKVPFPLTFR

VYIFNVTNPDRVQMGDMPILNEVGPFCYEEWKEKLNVEDMEADDTISYDPIDTFLKKRWP

GCKTGRELVTIPHPMILGLVNTVIRQKPGALSLANKAIKSIYDNPTSIFITTEADNLLFD

GVNINCGVNDFAGKAICSQLKTSGNLRKINEEQLLFSLLGPKNATLNQRIKAYRGTKKFQ

DVGRILEFAGATKLDVWPTDECNEIKGTDGTIFPPFLEKEQGLVSYSPDLCRSLRATFVK

KSEYNGIPCGEYTATFGDMSTNEDEKCYCLTSDTCMKKGIMDLYKCAGVPVYASLPHFYG

TDESYSNGVKGLAPNKTKHEIKILFESTTGSPLYARKRLQLSMPLEPIQKVELFNNFTAT

VIPIIWIEEGVDLNRTYTGQLKSLFQLKKAVGVIKWMILLSSLGGLAAAGYIFYKNNGKT

EITPVHEIKRDGISRIHSLEGQVNHGMSDNNLDKF

>ClapSNMP1a

MQLAIKIIVSGVVLTLSSVIFALVAYGPLIRYAIRDQTSLRKKNQIRDIYLKIPFPLDFR

IYFFNISNPMEVQNGAKPVLKEIGPYCYDEYEEKVDIIDNEMEDSLTYNSYDIFEFNAEK

SRGLSEDDYVTILHPLLVGMAFQVNRDTPALLSFLNQAIVPIFKDPKSIYLTDTVKNILF

DGFEINCNVSEFAAKAVCTQLMNSNIPGLKNDPSRDNMLVFSLIGVRNATLGHTMKVSRG

IRHSESVGKVLEVDGKKEINKWVSKACNRYRGTDGWIIPPLLTPEEGVWTHSVDLCRNVK

AKYIRDTLVKGVNVRLYEADLGNMQKNEEDKCYCPTPTTCSRKGVFDLSKCMGAPIVASL

PHFLEADEIYRKQVEGMQPIHEKHIIRIYLEVITSAPLIGKKRMQMNFPIAPISKITLMS

KLPVALHPIFWLEEGVELEGDFLKIITNKLMLLEVANVSRWLAVFGGLTATGIGIYLYIK

NKKSITISSIDSMSNDSEITRSTNELMDQMKTLERTEKGQINPVLSGHEFDRYM

>ClapSNMP2b

DMFKKMPFPFTFKVFLFDIKNPEEIMQGAKPIVKETGPFVYKVHKWNSDIKWEGPDDISY

YAYTRFEFDEDASGRYTEDYKLTILNTPYLGMLLKVAETQAAALPMVEGVLADIFKENDG

LFIKVKVKDYLFDGLRMCENEGKGGDFAAGLVCKQVMAQAGDSKNLRVENNSVLFANLHY

KNDTHLGRFTIKAGVKNREESAHLALYNNQSYSTVWEGEKSICNRIEGLSTTVFPVNIEK

DMIFESFAEDICRRMKLTYKMDETVKGLKGYKFTAANDSFSLKNKNNTCYCNNKSTLLDG

NLGCVKDGITDLSTCTGSPVMVS
